# The ^1^H NMR Spectroscopic Effect of Steric Compression Is Found in [3.3.1]Oxa- and Azabicycles and Their Analogues

**DOI:** 10.1021/acsomega.1c01093

**Published:** 2021-05-07

**Authors:** Ziyu Zeng, Gabriele Kociok-Köhn, Timothy J. Woodman, Michael G. Rowan, Ian S. Blagbrough

**Affiliations:** † Department of Pharmacy and Pharmacology, University of Bath, Bath BA2 7AY, U.K.; ‡ Material and Chemical Characterisation Facility, University of Bath, Bath BA2 7AY, U.K.

## Abstract

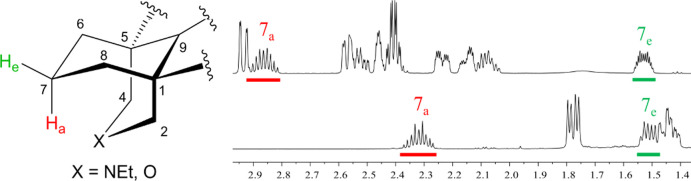

The through-space ^1^H NMR effect of steric compression by the lone-pair electrons of O- and N-atoms is shown in synthetic [3.3.1]oxa- and azabicycles. The electrons of the compressed proton bond are pushed away by the repulsive force generated by the lone-pair electrons of the heteroatom. There is a corresponding significant increase in the chemical shift of the compressed proton. The intensity of this deshielding effect is related to the proximity and overlap of the lone-pair or compressing atom. The steric compression decreases when the lone-pair electrons of the heteroatom and the compressed proton are not directly overlapped, for example, in [4.3.1]- and [3.2.1]azabicycles. Steric compression is also caused by a proton, deuterium, or an ethyl group close in space to the compressed proton. The protonated [3.3.1]azabicycle adopts a true-boat/true-chair conformation in its crystal lattice, but in solution the conformation is true-chair/true-chair.

## 1 Introduction

**Figure 1:**
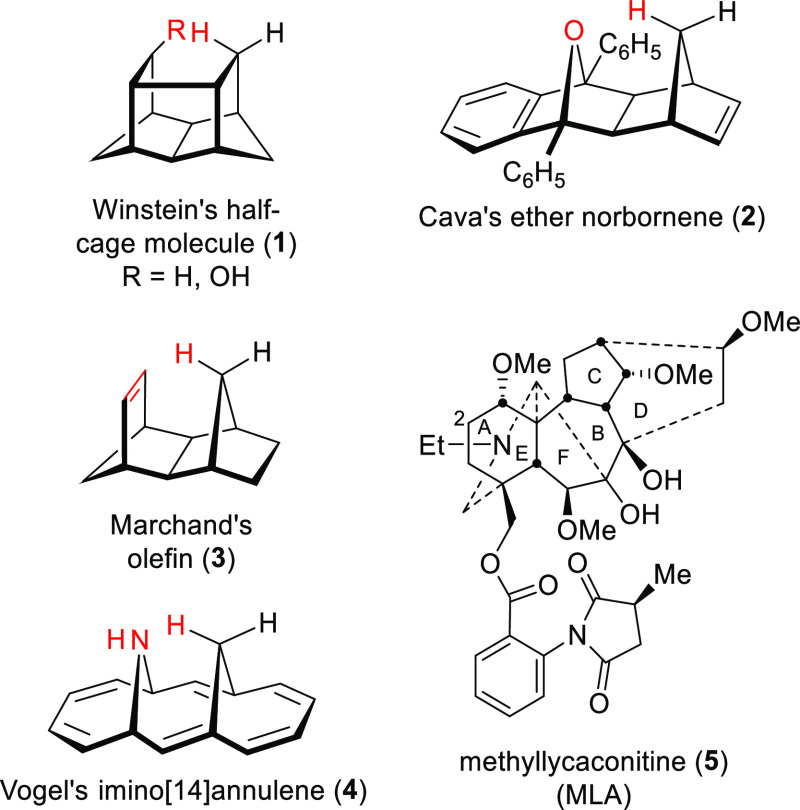
Designed reporter molecules (**1–4**) and the natural product methyllycaconitine (**5**, MLA).

The intramolecular through-space interaction that causes steric compression is detectable in ^1^H NMR studies. The chemical shifts of the compressed proton and its neighbor on the methylene group shift significantly.^1^ Some inflexible C-skeleta (Figure 1), for example, half-cage cyclopentyl (**1**), norbornenes (**2** and **3**), and imino[14]annulene (**4**), have been designed and synthesized to demonstrate elegantly that the compressed proton must be close in space to the source of the repulsive force, for example, H, OH,^1,2^ ether oxygen,^3^ alkene π-cloud,^4−6^ and NH.^7^

For two protons attached to the same carbon of a cyclohexane ring in a chair conformation, it is known that the chemical shift of the equatorial proton (∼1.6 ppm) is larger than that of its geminal axial proton (1.1 ppm) by Δδ ∼ 0.5 ppm (at −103 °C in CS_2_, 60 MHz),^8^ and this is the result of the magnetic anisotropic effect.^9^ For 4-H_a_ and 4-H_e_ of cyclohexanone, the difference between their chemical shifts (0.24 ppm, at <−185 °C in a 5:1 CHClF_2_/CHCl_2_F mixture, 251 MHz) is relatively small.^10^

C_19_-Norditerpenoid alkaloid methyllycaconitine (**5**, MLA) is one of the most potent competitive antagonists of α7-nicotinic acetylcholine receptors (α7-nAChRs) with a highly selective targeting of the snake venom toxin α-bungarotoxin (α-BgTx) binding sites.^11^ In terms of structure, norditerpenoid alkaloids are hexacyclic with bridged structures leading to well-defined conformations. The synthesis of cyclic analogues mimicking MLA (**5**)^12−16^ has been reported where the C2 axial and equatorial methylene group protons show a significant difference (∼1 ppm) in their chemical shifts.^17−22^

In this study, the large ^1^H NMR separations of the two signals on the methylene groups of different synthetic bridged [3.3.1]oxa- and azabicycles are reported. In order to explain this observation, comprehensive 1D/2D NMR spectroscopy and single-crystal X-ray analyses have been undertaken, which demonstrate that the effect of steric compression is found in these [3.3.1]bicycles and compared with the effect in analogues of various ring sizes.

## 2 Results and Discussion

### 2.1 Conformational Analysis of [3.3.1]Azabicycles

**Scheme 1:**
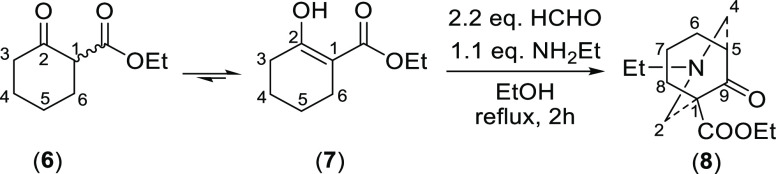
Synthesis of [3.3.1]Azabicycle (**8**)

An A/E-bicyclic [3.3.1]analogue (**8**) of norditerpenoid alkaloids was prepared *via* a double-Mannich reaction (Scheme 1). ^1^H, ^13^C, heteronuclear single-quantum coherence (HSQC), heteronuclear multiple bond correlation (HMBC), correlation spectroscopy (COSY), and nuclear Overhauser enhancement spectroscopy (NOESY) were used to assign the product (**8**).^12−22^ One of the protons attached to C7 is significantly deshielded and resonates at 2.86 ppm; the geminal methylene proton resonates at 1.53 ppm. The chemical shifts of these 7-H are separated by 1.33 ppm. This deshielding must be through space, as there are no significantly electronegative functional groups nearby.

**Figure 2:**
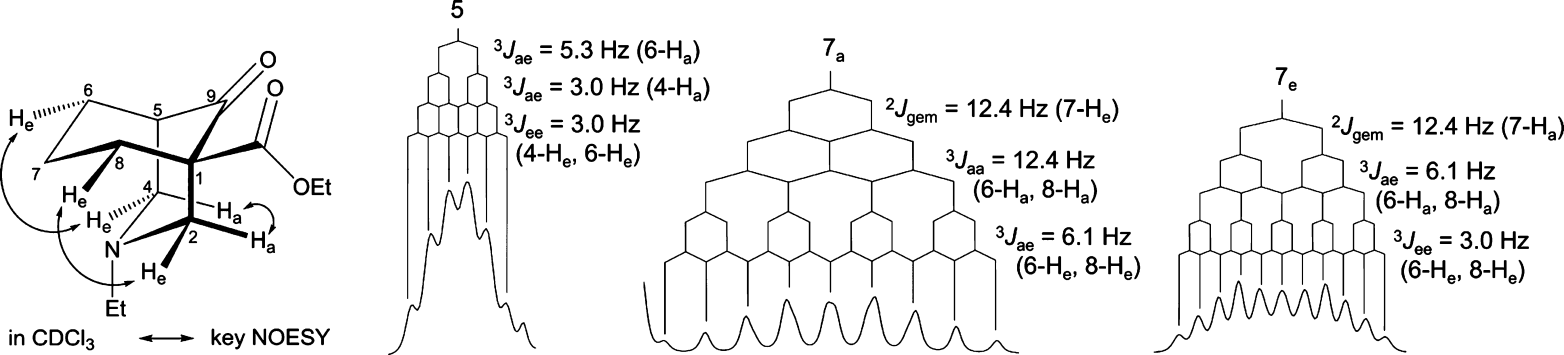
Key NOESY correlations and the coupling patterns of key ^1^H NMR signals of [3.3.1]azabicycle (**8**).

The tertiary amine nitrogen is assumed to be responsible for the observed shift as it should be close to 7-H_a_, if the [3.3.1]azabicycle (**8**) adopts a chair/chair conformation. The deshielding was observed for 7-H_a_. This is an example of steric compression, where the lone-pair electrons of the N-atom generate a repulsive force pushing the electron cloud surrounding 7-H_a_ away and thus decreasing its electron density, leading to 7-H_a_ resonating at a low field. This explanation holds if the [3.3.1]azabicycle (**8**) adopts a chair/chair conformation with the *N*-ethyl group in the equatorial position and therefore the N-atom lone-pair electrons are close to 7-H_a_. Due to the intramolecular hindrance in the “half-cage” structure, it is certainly possible that the AE-[3.3.1]bicycle (**8**) can adopt a boat/chair or a chair/boat conformation rather than an “obvious” chair/chair conformation. NOESY data (Figure 2) of the [3.3.1]azabicycle (**8**) were obtained. 2-H_a_ is correlated with 4-H_a_. 2-H_e_ and 4-H_e_ are correlated with 8-H_e_ and 6-H_e_, respectively. Therefore, the piperidine ring of [3.3.1]azabicycle (**8**) has adopted a chair conformation.

**Figure 3:**
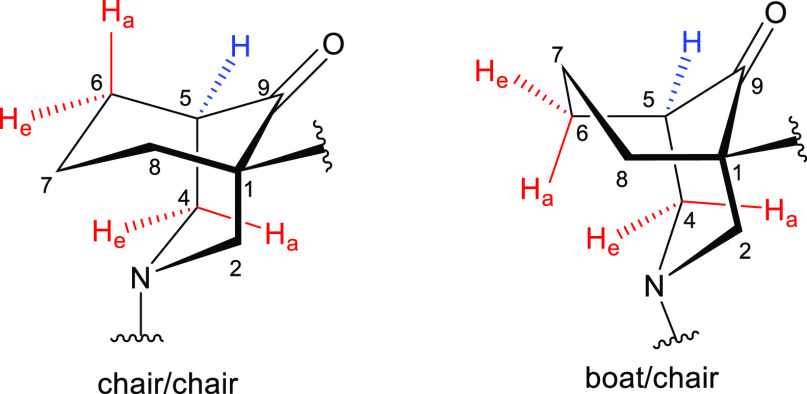
[3.3.1]Azabicycles in two different conformations.

The coupling pattern of 5-H of the compound (**8**) is shown in Figure 2, which is displayed as a dq peak (5.3, 3.0 Hz). The lack of a large coupling constant (caused by 6-H_e_ in the eclipsed position of a boat/chair conformation, Figure 3) for 5-H strongly suggests that the cyclohexane ring of this [3.3.1]azabicycle (**8**) is in a chair conformer. 5-H couples with 4-H_a_, 4-H_e_, 6-H_a_, and 6-H_e_. If the [3.3.1]azabicycle (**8**) adopts a chair/chair conformation, dihedral angles ∠(4-H_a_)–C4–C5–(5-H), ∠(4-H_e_)–C4–C5–(5-H), ∠(6-H_a_)–C6–C5–(5-H), and ∠(6-H_e_)–C6–C5–(5-H) are the same and equal to ∼60° (Figure 3). Therefore, two small ^3^*J*_ae_ (typically 5–7 Hz, 4-H_a_, and 6-H_a_) and two even smaller ^3^*J*_ee_ (typically 2–4 Hz, 4-H_e_ and 6-H_e_) are supposed to be displayed in the coupling pattern of the equatorial 5-H, according to the Karplus relationship. If the bicycle (**8**) adopts a boat/chair conformation, dihedral angles ∠(4-H_a_)–C4–C5–(5-H), ∠(4-H_e_)–C4–C5–(5-H), and ∠(6-H_a_)–C6–C5–(5-H) are still the same ∼60° (6-H_a_ in the cyclohexane ring adopting a boat conformation is 6-H_e_ in the cyclohexane ring adopting a chair conformation), but 6-H_e_ is now in the eclipsed position of the equatorial 5-H, so ∠(6-H_e_)–C6–C5–(5-H) is ∼0° which generates a large coupling constant (∼10 Hz), so the signal of 5-H should be a “doublet” when the conformation is a boat/chair.

The coupling patterns of 7-H_a_ and 7-H_e_ are different (Figure 2), which were used to assign their orientations. 7-H_a_ couples with 7-H_e_, 6-H_a_, 8-H_a_ (^2^*J*_gem_ = ^3^*J*_aa_ = 12.4 Hz) and 6-H_e_ and 8-H_e_ (^3^*J*_ae_ = 6.1 Hz); therefore, it shows as a qt. 7-H_e_ couples with 7-H_a_ (^2^*J*_gem_ = 12.4 Hz); 6-H_a_, 8-H_a_ (^3^*J*_ae_ = 6.1 Hz); and 6-H_e_ and 8-H_e_ (^3^*J*_ae_ = 3.0 Hz); therefore, it resonates as a dtt. These assignments confirmed that the 7-H_a_ is deshielded rather than shielded by the lone-pair electrons of the N-atom.

The coupling constants of 6-H_a_ and 8-H_a_ that are caused by 7-H_a_ are equal (12.4 Hz); therefore, the dihedral angles ∠(6-H_a_)–C6–C7–(7-H_a_) and ∠(8-H_a_)–C8–C7–(7-H_a_) are the same or highly similar, indicating this chair conformation of the cyclohexane ring is true (not twisted).^23,24^

NMR spectra of azabicycle (**8**) are also obtained in CDCl_3_, CD_3_OD, and *d*_6_-DMSO, and the chemical shifts for 7-H_a_ and 7-H_e_ are given in Table S8. In all three solvents, the 7-H_a_ of azabicycle (**8**) is significantly deshielded; therefore, this effect cannot be attributed to solvent effects. In addition, variable temperature ^1^H NMR experiments of azabicycle (**8**) in *d*_6_-DMSO were used to investigate the stability of the chair/chair conformation (Figure S1). It is clear that the chemical shifts of both 7-H_a_ and 7-H_e_ barely change (∼0.2 ppm) when the azabicycle (**8**) solution was heated, so 7-H_a_ still experiences steric compression; therefore, the molecule adopts a nearly inflexible chair/chair conformation at 25–125 °C.

**Figure 4:**
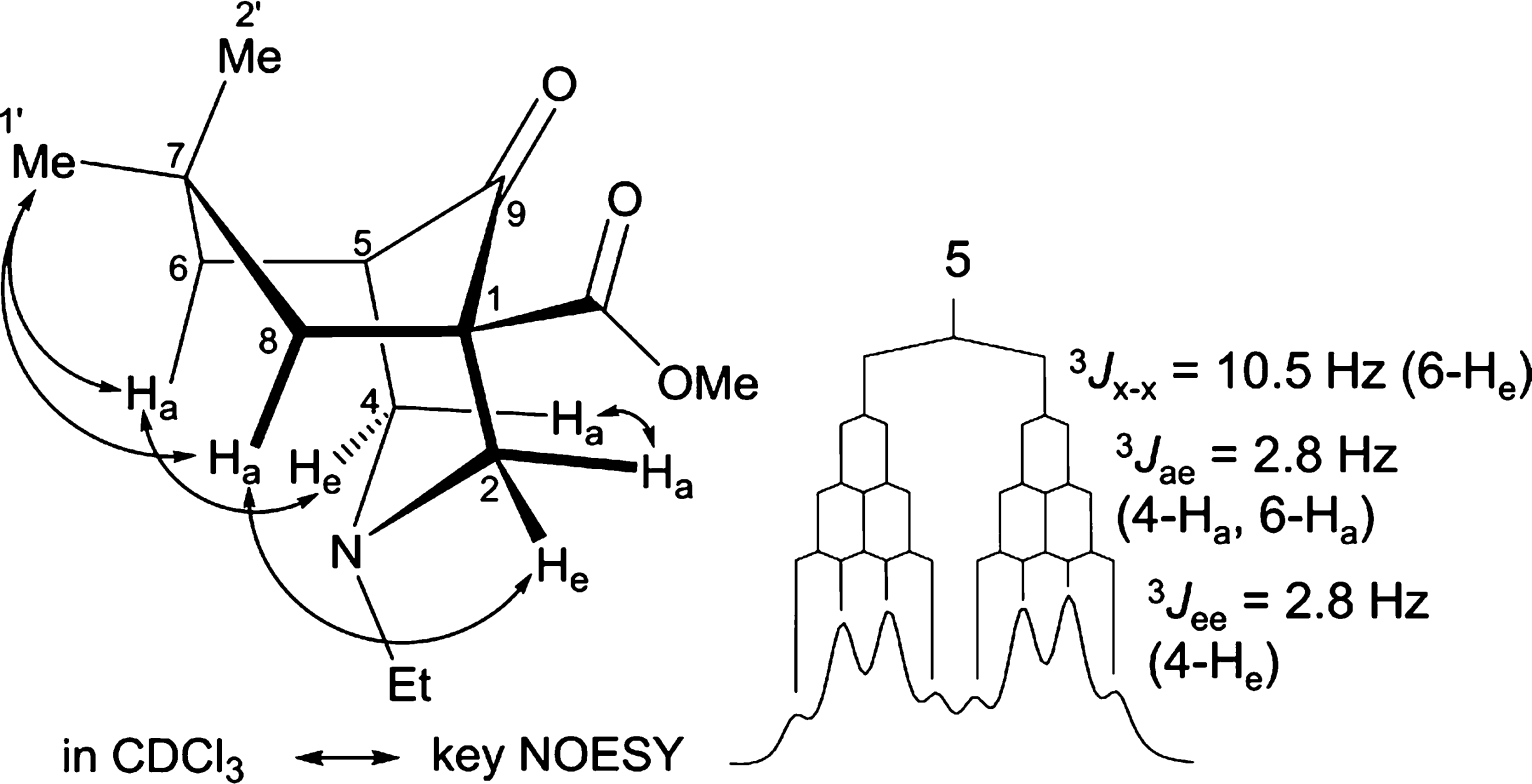
NOESY correlations and the coupling pattern of 5-H of boat/chair [3.3.1]azabicycle (**10**).

**Scheme 2:**
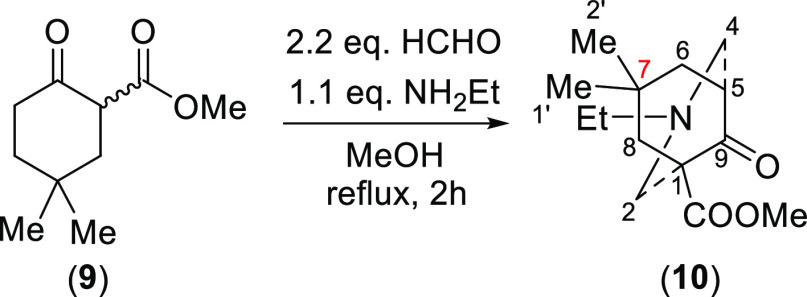
Synthesis of 7,7-Dimethyl[3.3.1]azabicycle (**10**)

The 7,7-dimethyl[3.3.1]azabicycle (**10**) was prepared (Scheme 2) as an example of adopting a boat/chair conformer. As two methyl groups are introduced at C7, the cyclohexane or piperidine ring flips into a boat conformation. The piperidine ring is in a chair conformation as shown by the NOESY correlations of 2-H_a_/4-H_a_, 2-H_e_/8-H_e_, and 4-H_e_/6-H_e_ (Figure 4). In contrast to the coupling pattern of 5-H in [3.3.1]azabicycle (**8**, Figure 2), 5-H of 7,7-dimethyl[3.3.1]azabicycle (**10**) displayed a dq with a large coupling constant (^3^*J*_x–x_ = 10.5 Hz) that originates from eclipsed 6-H_e_ (Figure 4), confirming that the cyclohexane ring of 7,7-dimethyl[3.3.1]azabicycle (**10**) adopts a boat conformation.

**Scheme 3:**
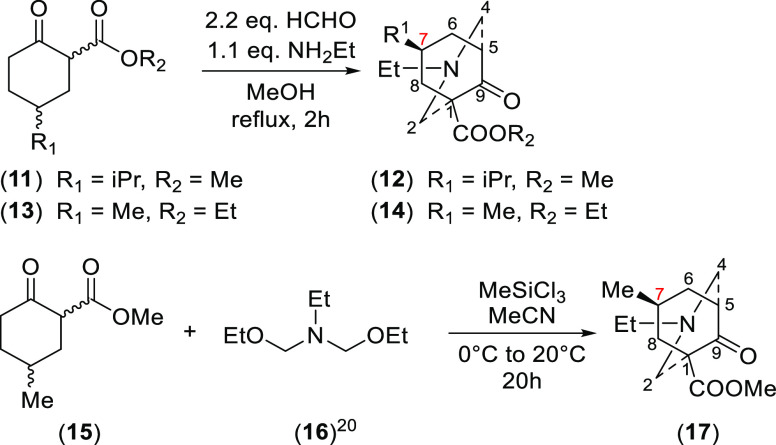
Synthesis of 7-Alkyl-Substituted [3.3.1]Azabicycles

To support the ^1^H NMR assignment of 7-H_a_ and 7-H_e_ of the azabicycle (**8**), 7-alkyl substituted [3.3.1]azabicycle (**12**), (**14**), and (**17**) were synthesized (Scheme 3). In these [3.3.1]azabicycles (**12**), (**14**), and (**17**), the bulky 7-alkyl groups will preferentially adopt the equatorial positions and therefore the δ (7-H_a_) can be demonstrated unequivocally. The δ (7-H_a_) of products (**12**), (**14**), and (**17**) resonate (in CDCl_3_) at 3.02, 3.42, and 3.42 ppm, respectively, which are similar values to the δ (7-H_a_) of azabicycle (**8**) as these 7-H_a_ are experiencing the same steric compression. Therefore, assignments of 7-H_a_ at 2.86 ppm and of 7-H_e_ at 1.53 ppm of the [3.3.1]azabicycle (**8**) are confirmed.

**Figure 5:**
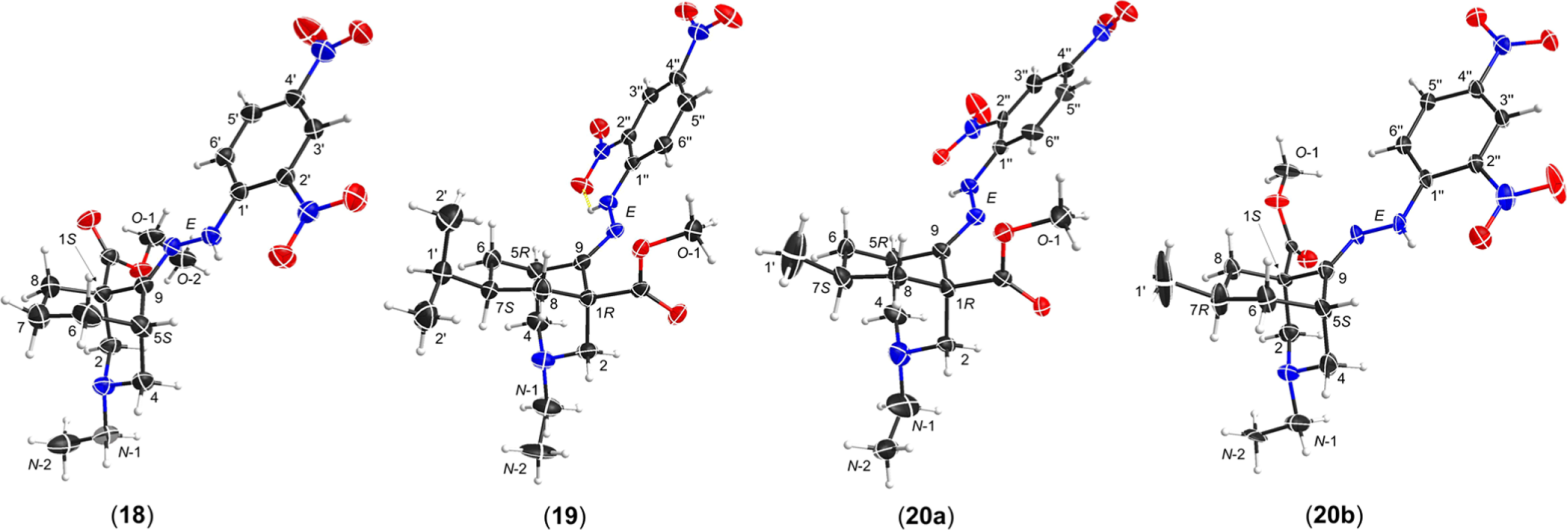
SXRD data of 2,4-DNP derivatives (**18–20**), ORTEP presentations of the crystal structures show the atom position with a 50% probability for each ellipsoid.

**Scheme 4:**
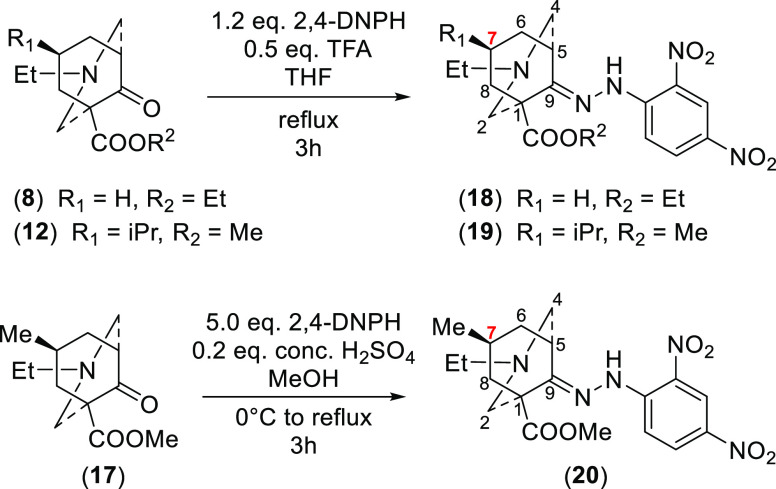
2,4-DNP Derivatization of [3.3.1]Azabicycles (**8**), (**12**), and (**17**)

2,4-Dinitrophenylhydrazine (2,4-DNPH) was used to derivatize these oily ketones (**8**), (**12**), and (**17**) in order to obtain crystalline derivatives (**18–20**, respectively Scheme 4) for single-crystal X-ray diffraction (SXRD). NMR data of dinitrophenylhydrazone (DNP) derivatives (**18–20**) also show that each 7-H_a_ resonates at a low field (Table S9), suggesting that both the ketone starting materials (**8**), (**12**), and (**17**) and their DNP derivatives (**18–20**) adopt the same solution conformations. SXRD data (**18–20**) were obtained (Figure 5). The [3.3.1]azabicyclic DNP derivatives (**18**), (**19**), (**20a**), and (**20b**) adopt true-chair/true-chair conformations with the *N*-ethyl groups in the equatorial positions and 9-imine hydrazinyl groups adopt E-configurations. 7-*i*Pr and 7-Me groups of DNP derivatives (**19**), (**20a**), and (**20b**) are equatorial. These conclusions, based on the SXRD data, are consistent with the NMR studies of the synthetic [3.3.1]azabicycles (**8**), (**12**), and (**17–20**). The single crystals of 7-Me derivatives (**20a**) and (**20b**) are packed in the same unit cell; they are enantiomers.

### 2.2 Impacts of Change in Ring Size on Steric Compression

**Figure 6:**
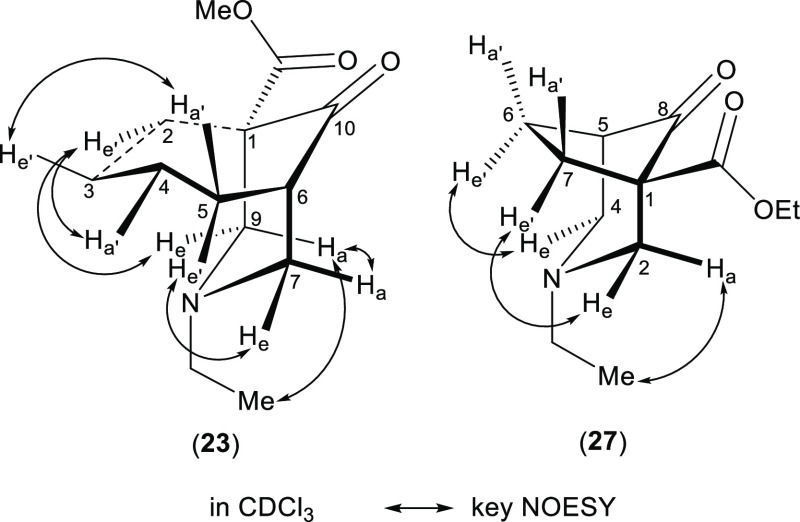
Key NOESY correlations of [4.3.1]- and [3.2.1]azabicyclic analogues (**23**) and (**27**).

**Scheme 5:**
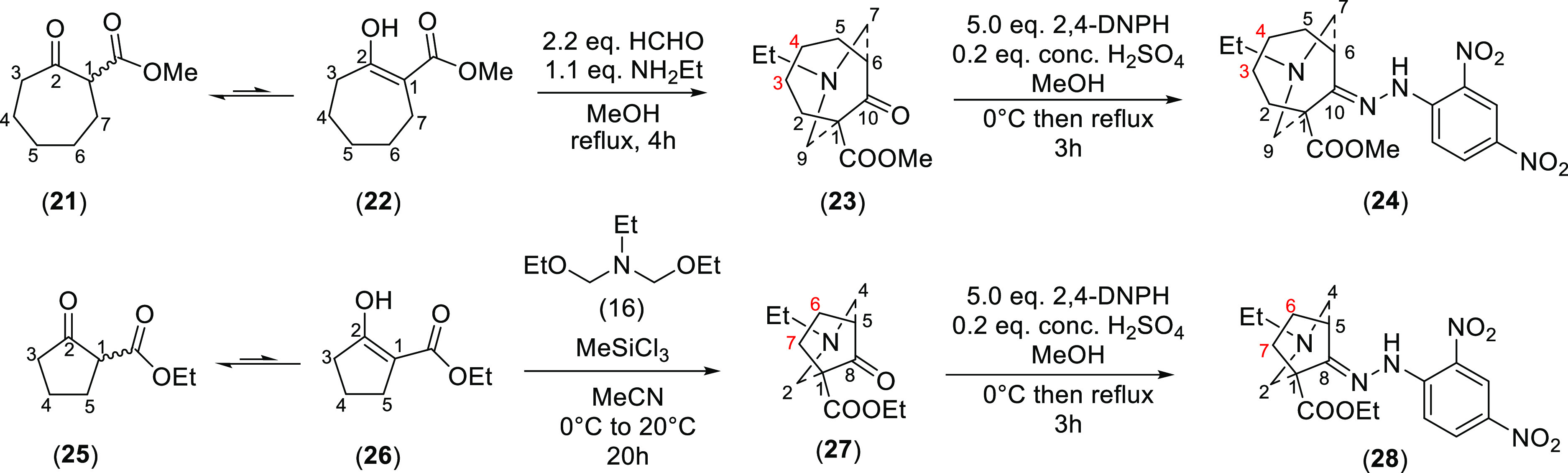
Synthesis of [4.3.1]- and [3.2.1]Azabicyclic Analogues

To investigate whether the ring size influences the ^1^H NMR effect of steric compression, [4.3.1]- and [3.2.1]azabicyclic analogues (**23**) and (**27**) were prepared (Scheme 5). According to key NOESY correlations of [4.3.1]- and [3.2.1]azabicycles (**23**) and (**27**) (Figure 6), all the orientation assignments are confirmed and the piperidine rings of both analogues (**23**) and (**27**) are proven to adopt chair conformations. As δ (3-H_a′_) and δ (4-H_a′_) of [4.3.1]azabicycle (**23**) are larger than δ (3-H_e′_) and δ (4-H_e′_), thus these 3-H_a′_ and 4-H_a′_ are experiencing steric compression (Δδ_3-H_ = 0.68 ppm, Δδ_4-H_ = 0.45 ppm, Table S10), and the cycloheptane ring adopts a chair conformation.^24^ Axial and equatorial are suitable for describing the orientations of protons attached to a six-membered ring, for example, cyclohexane in a true-chair conformation. For cyclopentane and cycloheptane rings, protons attached to the ring are better described as pseudo-axial (a′) and pseudo-axial (e′).^25^ In this study, a (a′) and a (e′) are preferred rather than exo and endo for retaining consistency with labeling used in the monoring system. Δδ_6-H_ and Δδ_7-H_ of [3.2.1]azabicycle (**27**) are only small values (<0.2 ppm). The steric compression decreases if the N-atom and the compressed proton are in a staggered relationship.

**Figure 7:**
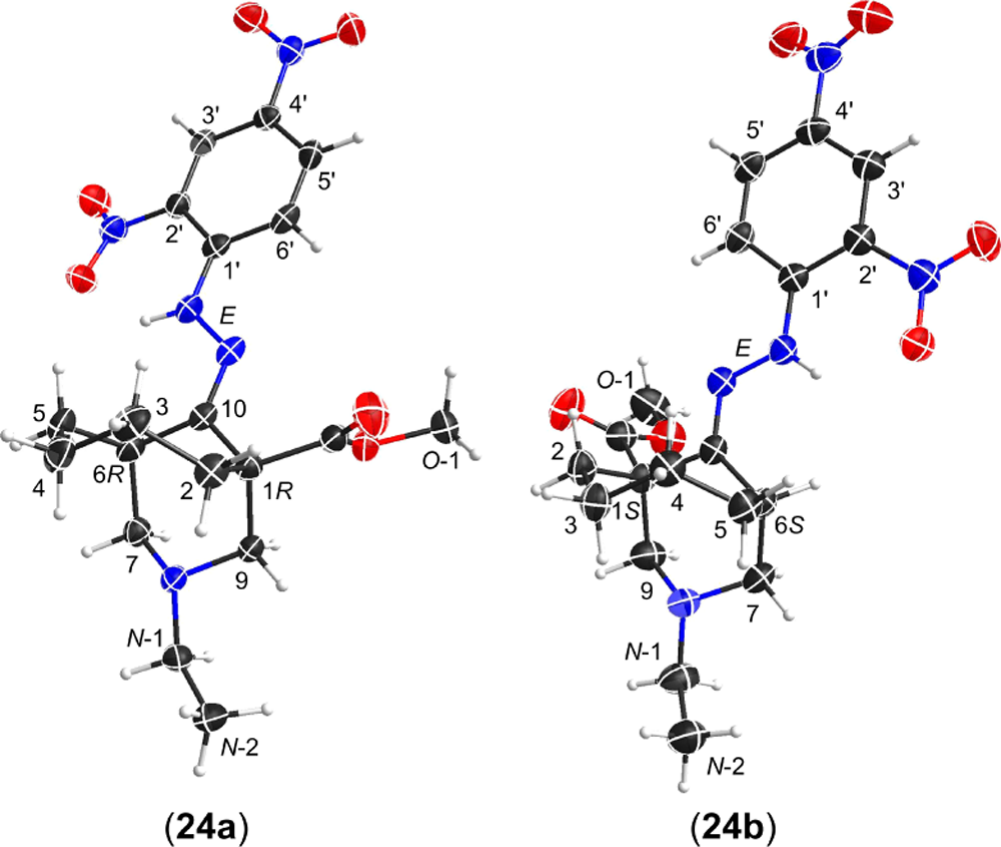
SXRD data of [4.3.1]azabicyclic DNP derivatives (**24a**) and (**24b**).

**Figure 8:**
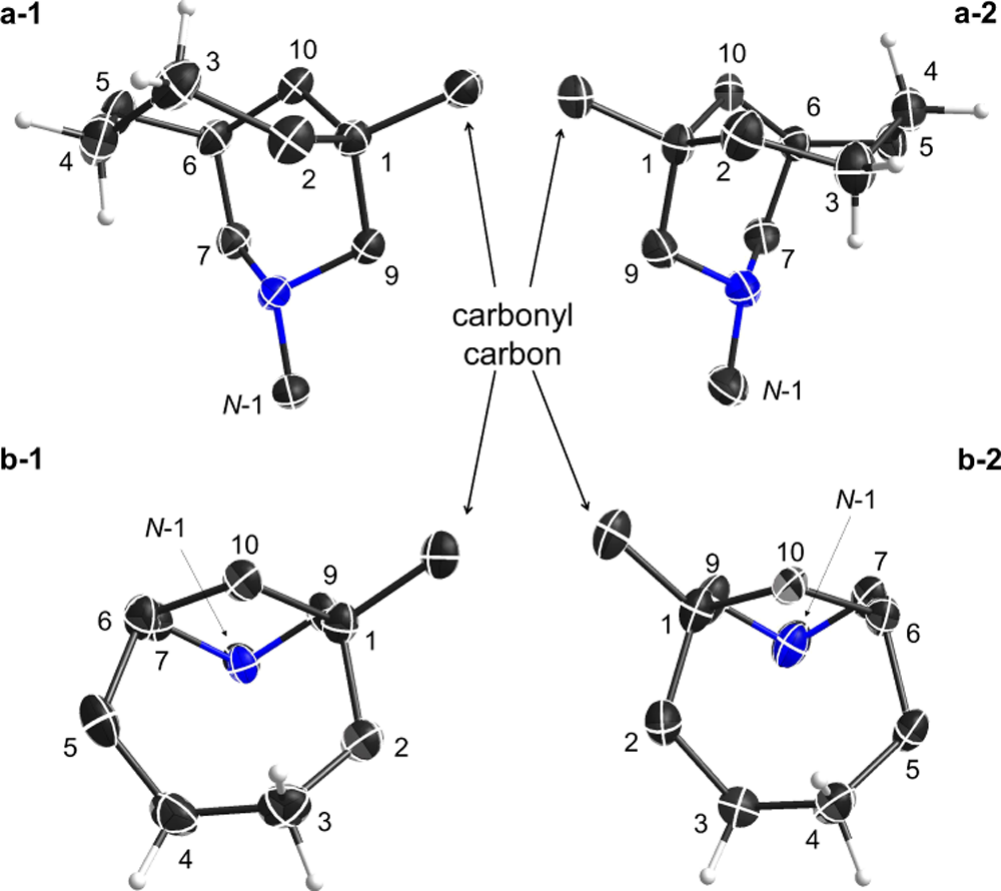
Comparison between bicyclic carbon skeleta in different stereoisomers (**24a**, left and **24b**, right).

Both [4.3.1]azabicycle (**23**) and [3.2.1]azabicycle (**27**) were converted into 2,4-DNP derivatives in order to produce crystalline products (**24**) and (**28**), and crystals of [4.3.1]azabicyclic derivative (**24**) suitable for X-ray analysis were obtained. Twin-packed crystal structures in one unit cell in this crystal are stereoisomers (**24a**) and (**24b**), and these stereoisomers are extracted from the original SXRD data and shown separately, as shown in Figure 7. Chair conformations of piperidine rings with equatorial *N*-ethyls are displayed in both isomers with hydrazone imines in the *E*-configuration, and these two stereoisomers (**24a**) and (**24b**) can be distinguished as 1*R*- and 1*S*-esters. Unlike the twin-packed crystal structures of enantiomers 7-Me [3.3.1]azabicyclic DNP derivative (**20a**) and (**20b**), the two crystal stereoisomers (**24a**) and (**24b**) are not mirror images on the basis of comparison between them in different view angles (Figure 8). A cycloheptane ring is more flexible and its conformations are more various than that of a cyclohexane ring.^26^

The NMR spectroscopic study of the [4.3.1]azabicycles (**23** and **24**, Table S10) showed that the Δδ_3-H_ is slightly larger than Δδ_4-H_, suggesting that 3-H_a′_ is experiencing more steric compression than 4-H_a′_, and thus, it may, on average, sit closer to the N-atom. This theoretically preferred conformation of [4.3.1]azabicycle revealed by NMR is similar to 1*S*,6*S*-isomer **24b** (Figure 8, right).

**Figure 9:**
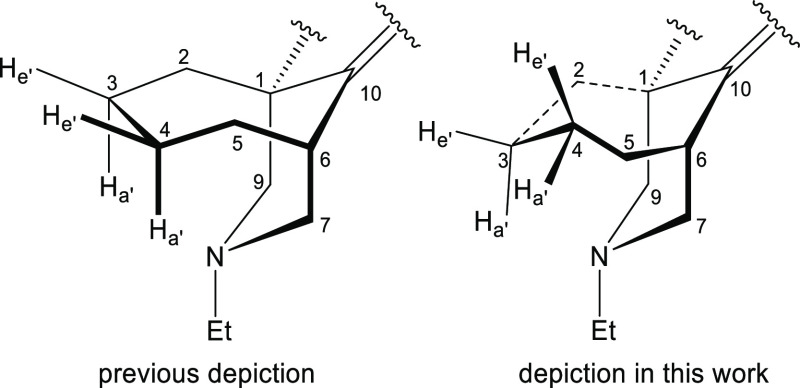
3D depictions of [4.3.1]azabicycle (**24b**).

Computer projections of the cycloheptane boat and chair conformations were reported by Bocian *et al.*(27) In their paper, they especially drew attention to the eclipsed hydrogens at the “stern”, the left side of the projection (Figure 9) of the boat, and the chair conformations. In the twist-chair conformation, these previously eclipsed hydrogen atoms are now shown on the basis of SXRD data and NMR analysis to be staggered.

### 2.3 No Steric Compression in a Mono-Mannich Product

**Scheme 6:**
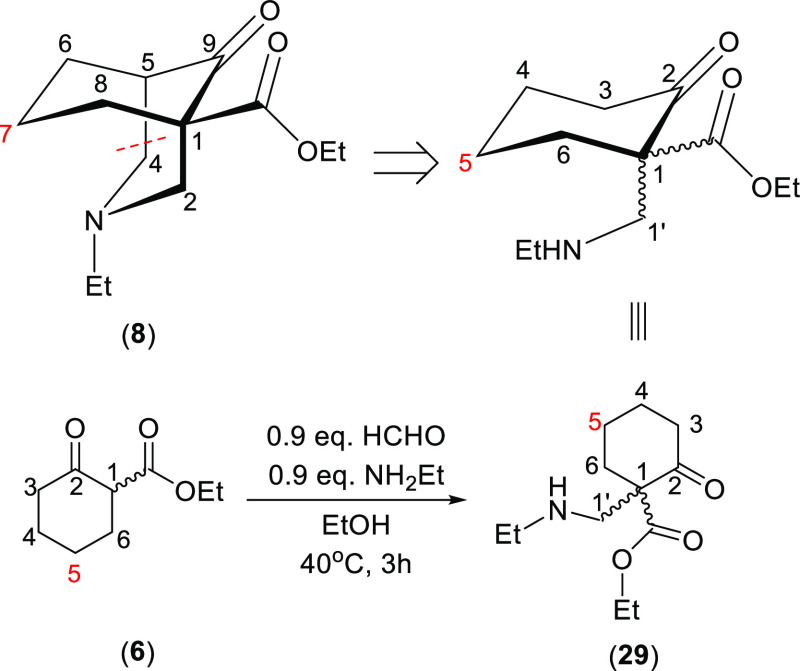
Synthesis of Mono-Mannich Product (**29**)

To prove that 7-H_a_ of the synthetic [3.3.1]azabicycles is being sterically compressed by the lone-pair electrons of the N-atoms, a mono-Mannich reaction was designed and carried out to give the monocyclic product (**29**). β-Keto ester (**6**) was treated with 0.9 equiv. formaldehyde and 0.9 equiv. ethyl amine, and the reaction was heated at 40 °C, rather than under reflux, for 3 h giving the target product (**29**) (Scheme 6). In the mono-Mannich product (**29**), there is no piperidine ring; thus, the lone-pair electrons of the N-atom are away from 5-H that correlates to 7-H of the double-Mannich products (**8**).

The ^13^C signal assignments of the mono-Mannich product (**29**) are assigned (Table S11) compared with those of β-keto ester (**6**, keto tautomer). Compared with Δ_7-H_ (1.33 ppm) of the double-Mannich product (**8**), Δ_5-H_ (0.09 ppm) of the mono-Mannich product (**29**) is significantly smaller, demonstrating that a significant ^1^H NMR steric compression of 7-H_a_ of [3.3.1]azabicycles requires the N-atom to be close in space to 7-H_a_.

### 2.4 Reducing 9-Ketone

**Scheme 7:**
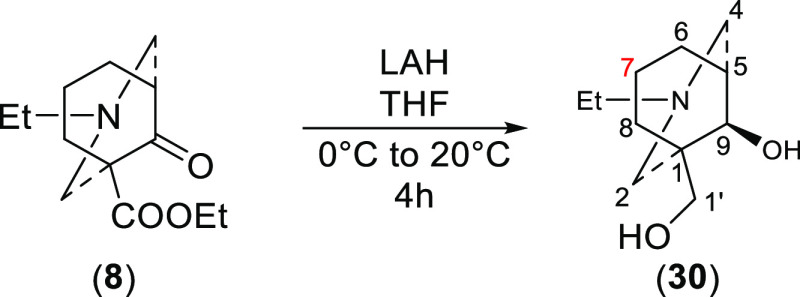
Reduction of Azabicycle (**8**) Providing Diol (**30**)

An alternative explanation for the observed chemical shifts may possibly be attributed to the 9-ketone group of [3.3.1]azabicycle (**8**) displaying a long-range anisotropic effect on axial or equatorial protons. To investigate this, the ketone (**8**) was treated with lithium aluminum hydride to reduce both the 1-ester and the 9-ketone functional groups affording a diol (**30**, Scheme 7).

**Figure 10:**
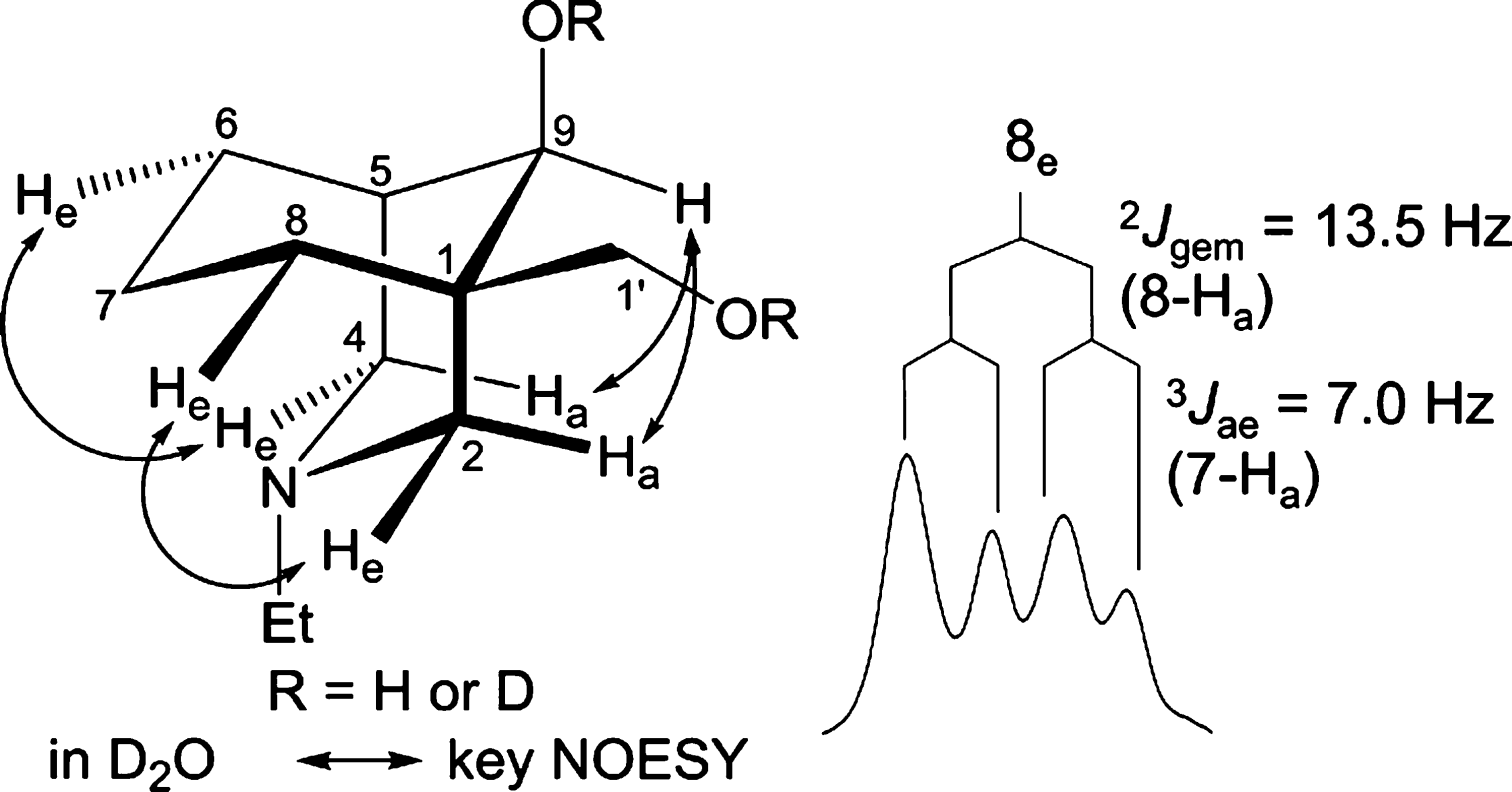
Key NOE correlations and the coupling pattern of 8-H_e_ of diol (**30**) in D_2_O.

**Figure 11:**
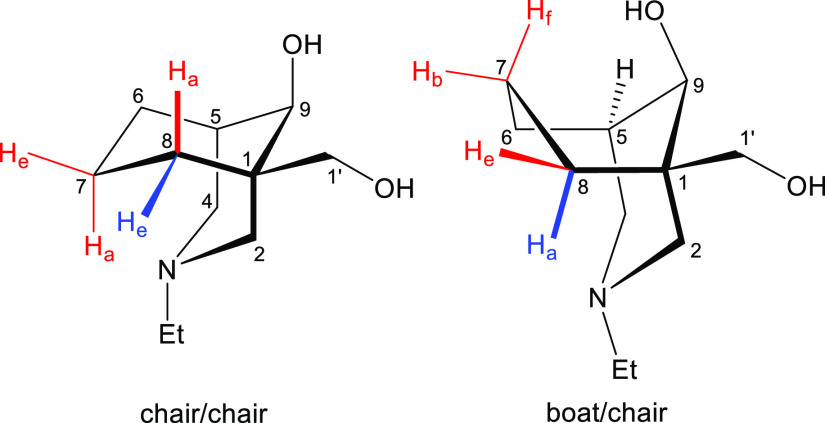
Diol (**30**) in two different conformations.

In CDCl_3_, CD_3_OD, and *d*_6_-DMSO, 9-ketone of azabicycle (**8**) has no obvious magnetic effect on 7-H_a_ or 7-H_e_ as chemical shifts of 7-H_a_ and 7-H_e_ of the diol (**30**) remain at similar values compared to those of 7-H_a_ and 7-H_e_ of ketone (**8**) when 9-ketone is reduced (Table S12). This is also consistent with diol (**30**) adopting true-chair/true-chair conformations in these three solvents. Interestingly, the relative difference in the shifts for 7-H_a_ of the diol (**30**) obtained in D_2_O is reduced by ∼0.7 ppm to Δδ_7-H_ = 0.45 ppm. The intensity of the effect of steric compression in diol (**30**) decreases in D_2_O, which is consistent with the solution conformation of the cyclohexane ring adopting a boat conformation. This makes the N-atom away from 7-H_a_. The orientation of 8-H_e_ of diol (**30**) has been confirmed by NOESY correlation 2-H_e_/8-H_e_, and this signal is a dd peak (Figure 10) as it couples with 8-H_a_ (^2^*J*_gem_ = 13.5 Hz) and 7-H_a_ (^3^*J*_ae_ = 7.0 Hz). Due to the absence of a large ^3^*J*_aa_ ∼ 14 Hz (7-H_f_, dihedral angle ∼ 180°, Figure 11), the diol (**30**) adopts a chair/chair conformation in D_2_O. When the cyclohexane ring of the diol (**30**) adopts a chair conformation (Figure 11), the coupling pattern of 8-H_e_, the proton that is close to 2-H_e_, should contain only one large coupling constant of ^2^*J*_gem_ ∼ 14 Hz (8-H_a_), and it also couples with 7-H_a_ and 7-H_e_ (typically ^3^*J*_ae_ ∼ 6 Hz and ^3^*J*_ee_ ∼ 3 Hz, both dihedral angles ∼ 60°). If the cyclohexane ring of this diol (**30**) adopts a boat conformation, 8-H_a_, which is 8-H_e_ of the chair-like cyclohexane ring, the proton that is close to 2-H_e_, then its coupling pattern should consist of two large coupling constants, ^2^*J*_gem_ ∼ 14 Hz (8-H_a_) and ^3^*J*_aa_ ∼ 14 Hz (7-H_f_, dihedral angle ∼ 180°), and it also couples with 7-H_b_, which results in a small coupling constant (dihedral angle ∼ 60°).

### 2.5 Protonation

**Scheme 8:**
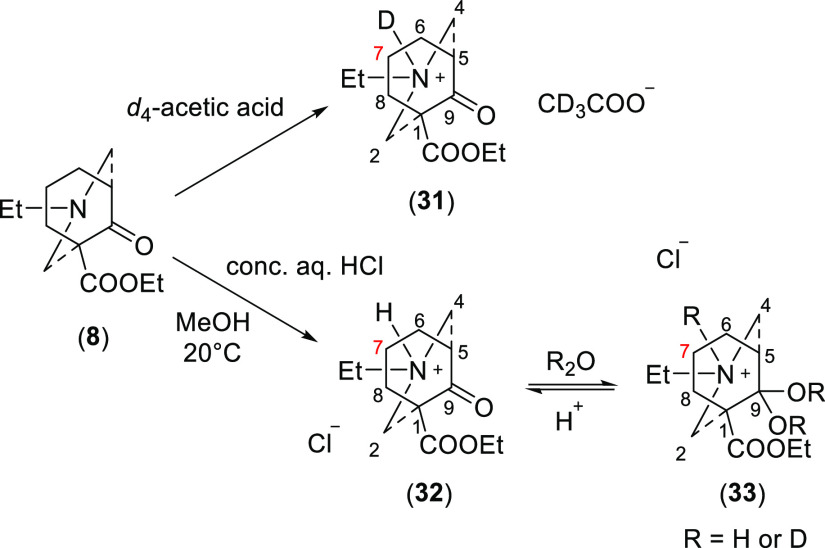
Acidification of [3.3.1]Azabicycle (**8**)

Norditerpenoid alkaloids and their synthetic analogues are bases. They are typically partly protonated in the aqueous components of body fluids even at neutral pH. Thus, it is valuable to understand the conformation of the protonated form of [3.3.1]azabicycle (**8**) that is synthesized for mimicking the A/E-rings of bioactive norditerpenoid alkaloids especially MLA (**5**). The [3.3.1]azabicycle (**8**) was separately dissolved in *d*_4_-acetic acid and concd HCl aq solutions in order to obtain protonated compounds (**31–33**, Scheme 8), which are the ketone salts (**31**) and (**32**) and the ketal (hydrate) salt (**33**), respectively.

**Figure 12:**
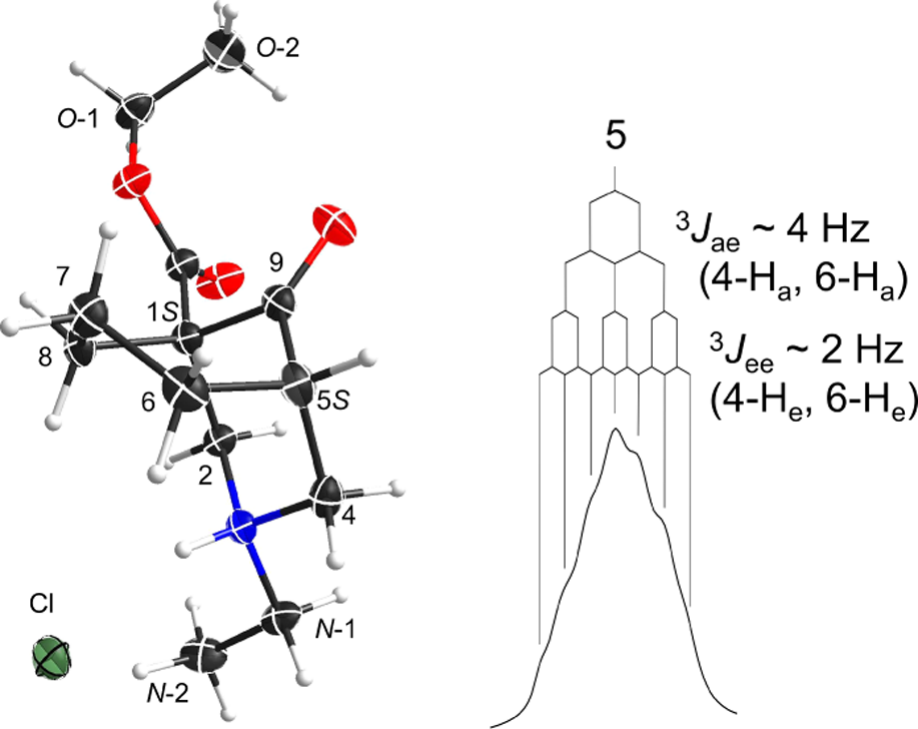
SXRD data of HCl salt (**32**) and the coupling pattern of 5-H of ketal salt (**33**).

The acetate salt (**31**) shows δ (7-H_a_) = 2.42 ppm > δ (7-H_e_) = 1.72 ppm in *d*_4_-acetic acid, Δδ_7-H_ = 0.70 ppm (Table S13), which means the NH(D) is able to provide steric compression acting on 7-H_a_. Therefore, the conformation of this salt (**31**) is determined to be true-chair/true-chair as significant compression is displayed. The compression is caused by the electrons in the new bond of NH/D, as there are no lone-pair electrons available. SXRD data of the chloride salt (**32**) were determined, which shows that this salt (**32**) adopts a true-boat/true-chair crystal conformation with the *N*-ethyl in the equatorial position (Figure 12).

NMR spectroscopic analyses of the crystalline chloride salt (**32**) in *d*_4_-acetic acid gave similar results to those of the acetate salt (**31**) in *d*_4_-acetic acid. NMR data of the chloride salt (**32**) show a significant Δδ_7-H_ = 0.87 ppm (Table S13), suggesting that the solution conformation of this chloride salt (**32**) in *d*_4_-acetic acid is true-chair/true-chair. Therefore, the crystal conformation of the synthetic [3.3.1]azabicyclic chloride salt (**32**) is different from its solution conformation.

When the crystalline ketone chloride salt (**32**) was dissolved in D_2_O (or wet solvents, e.g., CD_3_OD and *d*_6_-DMSO), the ketal (hydrate) salt (**33**) was obtained. To determine the conformation of this salt (**33**), the ^1^H signal of 5-H was employed (Figure 12). On the basis of the shape (even though broad) of this 5-H, it does not contain a large coupling constant such as ^3^*J*_x–x_ (10.5 Hz) of the 5-H of 7,7-dimethyl azabicycle (**10**), *cf.*Figure 4, this ketal salt (**33**) adopts a chair/chair conformation.

### 2.6 Methylation

**Figure 13:**
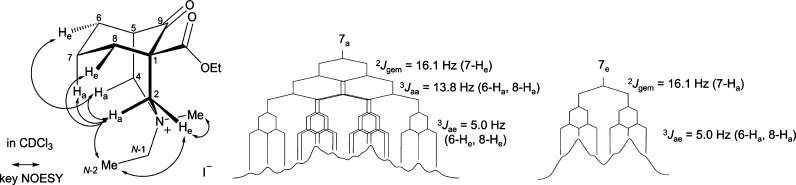
Key NOESY correlations and coupling patterns of key ^1^H NMR signals methylated [3.3.1]azabicycle (**34**).

**Scheme 9:**
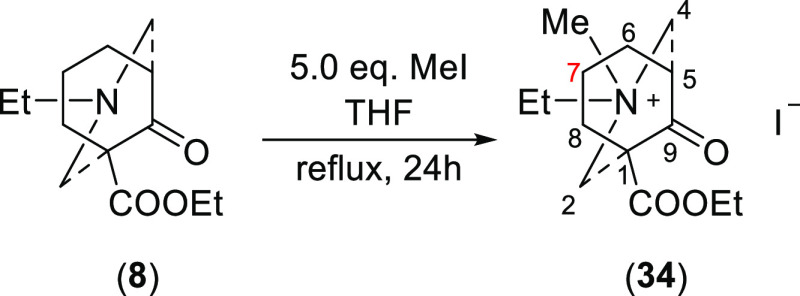
N-Methylation of [3.3.1]Azabicycle (**8**)

[3.3.1]Azabicycle (**8**) was methylated with MeI (5.0 equiv) heated under reflux for 24 h (Scheme 9). The key NOESY data of this methylated product (**34**) are given in Figure 13. The 2-H_a_ of the compound (**34**) has a NOESY correlation with 4-H_a_, and 2-H_a_ is also NOESY correlated with 8-H_e_, so the piperidine ring adopts a boat conformation. The 7-H_a_ is close to the 2-H_a_ in the space determined by NOESY correlation 2-H_a_/7-H_a_, thus the cyclohexane ring is in a chair conformation.

The N-2 has NOESY correlations with both 2-H_a_ and 2-H_e_, but the N–Me only show NOESY correlation with the 2-H_e_; therefore, the N–Me is in the flagpole position and the N–Et is determined to be in the bowsprit position.

The 7-H_a_ experiences the effect of steric compression presented by δ (7-H_a_) = 2.84 ppm > δ (7-H_e_) = 1.87 ppm, Δδ_7-H_ = 0.97 ppm. It is notable that the steric compression acting on the 7-H_a_ is caused by the methyl group of the N–Et rather than the lone-pair electrons of the N-atom, as the lone-pair electrons are no longer available.

If the piperidine ring adopts a true-boat conformation, the methyl group of the N–Et is far away from 7-H_a_ in space; therefore, the boat-like piperidine ring has to be mono-flattened (only the N-atom is flattened rather than both the N-atom and the C9 are flattened) allowing the methyl group of the N–Et to be close to the 7-H_a_ showing a significant steric effect on the 7-H_a_.

Both 6-H_a_ and 8-H_a_ of methylated [3.3.1]azabicycle (**34**) (Figure 13) contribute equal coupling constants of ^3^*J*_aa_ = 13.8 Hz to the 7-H_a_, which suggests that the cyclohexane ring adopts a true-chair conformation. The value of the ^2^*J*_gem_ between 7-H_a_ and 7-H_e_ of the methylated derivative (**34**) is 16.1 Hz, which is significantly larger than that of the ^2^*J*_gem_ (12.4 Hz) of the 7-H_a_ of azabicycle (**8**). This suggests that the ∠(7-H_a_)–C7–(7-H_e_) of the methylated derivative (**34**) becomes smaller than that of the azabicycle (**8**), as 7-H_a_ experiences a strong compression through space changing the geminal bond angle of ∠(7-H_a_)–C7–(7-H_e_).

### 2.7 Synthesis and Analysis of [3.3.1]Oxabicycle

**Scheme 10:**
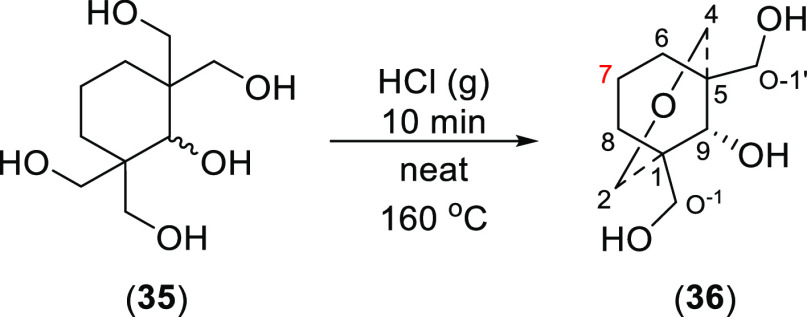
Synthesis of [3.3.1]Oxabicycle (**36**)

To add further data about the effect of steric compression on ^1^H NMR signals, a [3.3.1]oxabicyclic tetrahydropyranyl ether (**36**) was designed and then synthesized by intramolecular dehydration (Scheme 10).^28^

**Figure 14:**
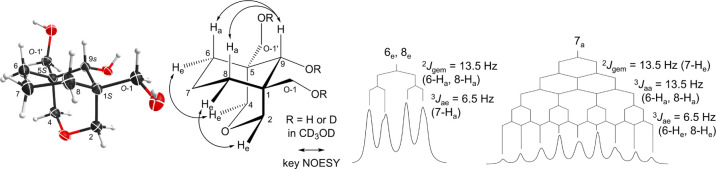
SXRD data, key NOESY correlations, and the coupling patterns of key ^1^H NMR signals of [3.3.1]oxabicycle (**36**).

The product was recrystallized from EtOAc (∼14 h). SXRD data of this [3.3.1]bicyclic ether (**36**) show a true-chair/true-chair conformation with 9-OH in the equatorial position (Figure 14), supported by the related NOESY data.

The signal of 6-H_e_ (8-H_e_) resonates as a dd (Figure 14), coupled with 6-H_a_ (8-H_a_, ^2^*J*_gem_ = 13.5 Hz) and 7-H_a_ (^3^*J*_ae_ = 6.5 Hz), determining that in solution the [3.3.1]oxabicycle (**36**) adopts a chair/chair conformation. The 7-H_a_ signal at 2.32 ppm is sterically compressed [δ (7-H_e_) = 1.51 ppm, Δδ_7-H_ = 0.81 ppm] by the lone-pair electrons of the ether O-atom. Hence, the solution conformation of this ether (**36**) is true-chair/true-chair as found in its crystal lattice (Figure 14). The solution data are supported by the coupling pattern of 7-H_a_ resonating as a typical qt peak.

NMR data of [3.3.1]oxabicycle (**36**) in *d*_6_-DMSO, *d*_6_-acetone, and D_2_O are also obtained, and key ^1^H NMR data are given in Table S14. Compared with Δδ_7-H_ (0.81 ppm) of this ether (**36**) in CD_3_OD, Δδ_7-H_ acquired from *d*_6_-DMSO and *d*_6_-acetone are similar, 0.80 and 0.92 ppm, respectively. However, Δδ_7-H_ measured in the D_2_O solution is smaller (0.59 ppm). D_2_O (H_2_O) may form H-bonds with the ether O-atom, resulting in the oxygen lone-pair electrons perhaps being less available for compressing 7-H_a_.

## 3 Conclusions

**Figure 15:**
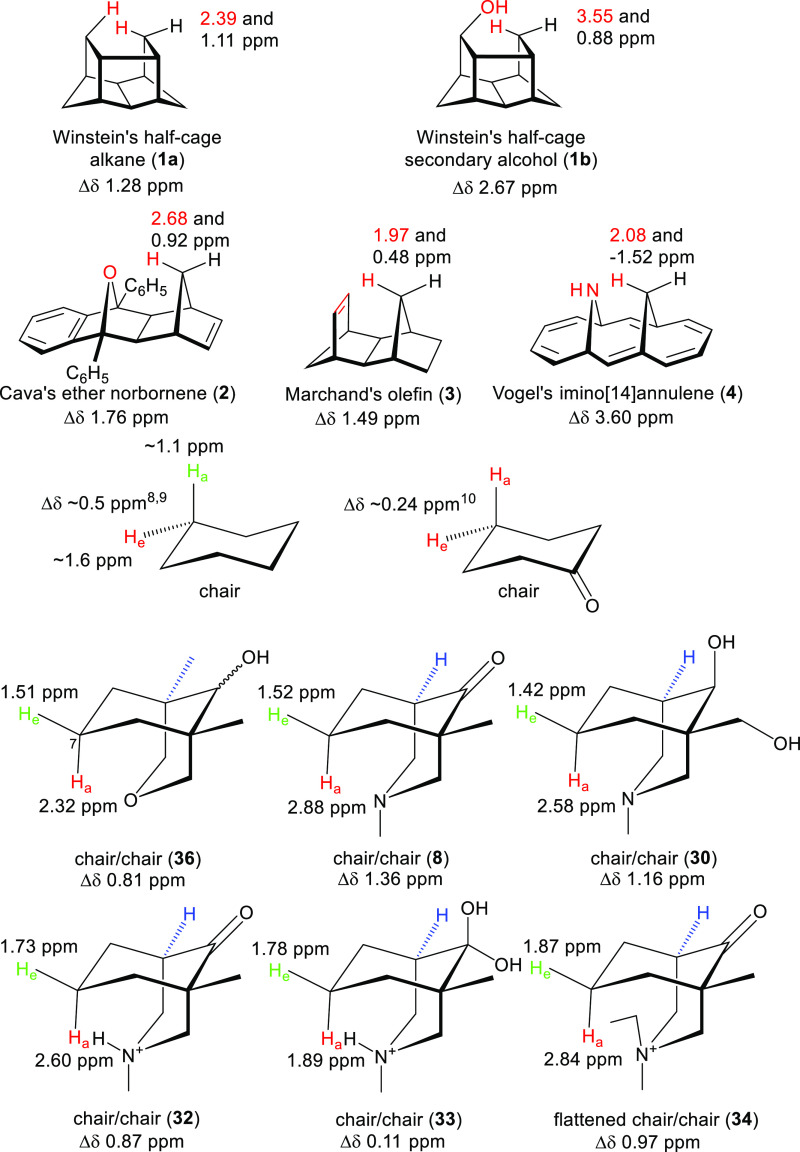
Intramolecular through-space interactions that cause steric compression are reported by the key ^1^H NMR signals across a range of inflexible half-cage-type molecules where the chemical shifts of the compressed proton and its methylene group neighbor shift significantly compared with those in unsubstituted chair conformers of cyclohexane^8^ and cyclohexanone^10^ (δ in ppm).

A through-space ^1^H NMR effect of steric compression displayed in [3.3.1]azabicycles is demonstrated and fully discussed. It is caused by the lone-pair electrons of the N-atom generating an intramolecular repulsive force acting on 7-H_a_ leading to this proton being significantly deshielded. By comprehensive conformational analysis on these bicyclic compounds and their analogues *via* NOESY, coupling pattern analysis of key ^1^H NMR signals, and SXRD the conformation of the bicycles and the configuration of protons of different methylene groups that experience steric compression are unambiguously assigned. The intensity of this compression is also proven to be related to the distance between the N-atom, especially its lone-pair electrons and the compressed proton: smaller distance and larger intensity. The key NMR data of several typical [3.3.1]bicycles in this work are summarized in Table 1 and shown compared to the literature data in Figure 15.

**Table 1:** ^1^H NMR Data of Key Methylenes in [3.3.1]Bicycles (δ in ppm)

compound	solvent	δ (7-H_a_)	δ (7-H_e_)	Δδ_7-H_
[3.3.1]azabicycle (**8**)	CDCl_3_	2.86	1.53	1.33
	CD_3_OD	2.88	1.52	1.36
7-*i*Pr [3.3.1]azabicycle (**12**)	CDCl_3_	3.02		
[3.3.1]azabicyclic diol (**30**)	CDCl_3_	2.59	1.48	1.11
	CD_3_OD	2.58	1.42	1.16
	D_2_O^a^	2.04	1.59	0.45
protonated [3.3.1]azabicycle ketone chloride salt (**32**)	*d*_4_-acetic acid	2.60	1.73	0.87
protonated [3.3.1]azabicycle ketal chloride salt (**33**)	CD_3_OD	1.78; 1.89^b^		0.11
	D_2_O	1.72; 1.83^b^		0.11
methylated [3.3.1]azabicycle (**34**)	CDCl_3_	2.84	1.87	0.97
[3.3.1]oxabicyclic ether (**36**)	CD_3_OD	2.32	1.51	0.81
	D_2_O	2.16	1.57	0.59

a With the addition of 2 drops of *d*_6_-DMSO.

b No reliable evidence was obtained to identify the orientation of these protons.

Half-cage cyclopentyl (**1**), norbornenes (**2** and **3**), and imino[14]annulene (**4**) elegantly demonstrate that the compressed proton must be close in space to the source of the repulsive force, for example, H, OH, ether oxygen, alkene π-cloud, and the only literature example of a secondary amine. Winstein and colleagues reported (in 1965) a new kind of steric compression on one proton of a methylene pair when the other proton is strongly compressed, for example, by an oxygen functional group. This was found together with unusually large deshielding effects in their half-cage or endo,endo-fused skeleta.^1,2^ Such rigid geometries and enormous H–H or H–O steric oppositions are ideally suited for the study of effects of steric compression on chemical shifts, where the inside protons are strongly deshielded. Cava and Scheel (in 1967) concluded that the ether oxygen bridge exerts considerable shielding and deshielding effects on the methylene bridge protons, which are separated from each other by Δδ = 1.76 ppm, where such a large difference in the chemical shifts of the methylene bridge protons of a norbornene or norbornane was then unprecedented.^3^ Marchand and Rose (in 1968) reported that of particular interest is the effect of the alkene electron π-cloud causing the unusually large value of Δδ = 1.49 ppm between the bridge norbornene protons.^4^ A comparable value had been noted only once before in the literature by Cava and Scheel.

The expected aromatic character was found in *syn*-1,6-imino-8,13-methano[14]annulene (**4**), first synthesized by Vogel and colleagues.^7^ This is the first, and outside of the [3.3.1]azabicycle of MLA (**5**) and related natural products and their analogues, and essentially the only (secondary) amine to show such a strong steric compression effect. The NH proton is exo-orientated, the bridge methylene protons are so magnetically different; they are an AX system, −1.52 (d, H_exo_) and 2.08 (d, H_endo_) (*J*_AX_ gem = 10.2 Hz). The chemical shifts of the two CH_2_ protons therefore differ by 3.6 ppm! The chemical shifts of the *exo*-CH_2_- and the NH-bridge protons are observed at a relatively high field, the *endo*-CH_2_-bridge proton is strongly deshielded. Such a large Δδ might be due in a considerable part to its steric interaction with the spatially very close (H)N-group, possibly due to the van der Waals effect. The NH-bridge proton is assigned to the exo-position with a high degree of certainty on the basis of its resonance at a relatively high field, NH_exo_ [CCl_4_, tetramethylsilane (TMS)] −2.07 (br s) ppm. If this proton were to be in the endo-position, then it would not only have to be markedly deshielded as a result of the H–H (steric) compression but also show a not-present nuclear Overhauser effect.^7^

**Figure 16:**
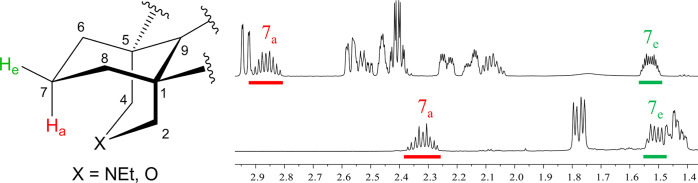
Steric compression reported by the 7-methylene key ^1^H NMR signals of [3.3.1]azabicycle (**8**) (upper) and [3.3.1]oxabicycle (**36**) (lower).

This effect of steric compression can not only be caused by the lone-pair electrons of the N- or another heteroatom (e.g., O-atom) (Figure 16) but also by a proton (deuterium) or alkyl, for example, ethyl group that is close in space to the compressed proton. This conclusion can help in understanding the conformations of molecules related to [3.3.1]bicycles as a true-chair/true-chair conformation allows a significant steric compression to be demonstrated.

## 4 Experimental Section

### 4.1 Materials and General Methods

2,4-Dinitrophenylhydrazine (∼70%, wet with ∼30% water) was purchased from Fluorochem (U.K.). All other chemicals were purchased from Sigma-Aldrich (U.K.) and used as received. Deuterated solvents including *d*_4_-acetic acid, *d*_6_-acetone, *d*-chloroform, *d*_4_-methanol, *d*_6_-DMSO, and deuterium oxide (D_2_O) were used for NMR experiments (99.8% D atom, Cambridge Isotope Laboratories, Inc., USA). All other solvents were of high-performance liquid chromatography grade, ≥99.9% purity (Fisher Scientific, U.K. and VWR, U.K.) including anhydrous solvents (Sigma-Aldrich, U.K. and Acros Organics, U.K.). Petroleum ether (Fisher Scientific, U.K.) specifically refers to the 40–60 °C distillate.

#### 4.1.1 Instrumentation

^1^H NMR spectra were recorded on Bruker Avance III spectrometers (^1^H Larmor precession frequency, 400 and 500 MHz) at 25 °C. Chemical shifts are expressed in parts per million (ppm) downfield from TMS or 3-(trimethylsilyl)-propionic-2,2,3,3-*d*_4_ acid sodium salt (TMSP) as internal or external standards, and residual (protio) solvent peaks were also used as internal standards if required. Chemical shifts (δ_H_) are reported as the position (accurate δ_H_ of overlapping signals were extracted from 2D NMR spectra, e.g., HSQC, COSY, and NOESY), relative integral, multiplicity, and assignment. Multiplicity is abbreviated: s = singlet, d = doublet, t = triplet, q = quartet, quin = quintet, m = multiplet; br = broad. Coupling constants (*J*) are line separations (absolute values expressed in hertz) rounded and rationalized to 0.1 Hz.

^13^C NMR spectra were recorded with complete proton decoupling on Bruker Avance III spectrometers (^13^C Larmor precession frequency 100 and 125 MHz) at 25 °C as well as 2D NMR experiments including HSQC and HMBC. Chemical shifts are expressed in a ppm downfield shift from TMS or TMSP as internal or external standards, and solvent peaks were also used as internal standards if required. Data are reported as the position (δ_C_), number of attached protons (CH_3_, CH_2_, CH, quat = quaternary), and assignment.

Positive-ion [M + H]^+^ mode-mass spectrometry was performed on samples dissolved in methanol, using Bruker micrOTOF and Agilent Q-TOF mass spectrometers equipped with electrospray ionization (ESI) sources. Negative-ion [M – H]^−^ mode-mass spectrometry was performed on samples dissolved in methanol, on an Agilent ESI-Q-TOF mass spectrometer. High-resolution mass spectra were within 5 ppm error unless otherwise stated.

Intensity SXRD data were collected at 150 ± 2 K on a Rigaku SuperNova Dual, EosS2 system using monochromated Cu Kα radiation (λ = 1.54184 Å). Unit cell determination, data collection, and data reduction were performed using CrysAlisPro software CrysAlisPro 1.171.39.46 (Rigaku Oxford Diffraction, 2018). An empirical absorption correction using spherical harmonics was employed. The structures were solved with SHELXT and refined by a full-matrix least-squares procedure based on *F*^2^ (SHELXL-2018/3).^29^ All nonhydrogen atoms were refined anisotropically. Hydrogen atoms were placed onto calculated positions and refined using a riding model.

The removal of solvents by evaporation in the procedures specifically refers to the use of a Buchi R-114 rotary evaporator with warming samples to 40 °C on a Buchi B-480 water bath and *in vacuo* (50–500 mbar).

#### 4.1.2 Chromatography

Flash chromatography^30^ was performed using silica gel 60A 35–70 μm (Fluorochem Ltd., U.K. and Sigma-Aldrich, U.K.) with the indicated solvents. Thin-layer chromatography (TLC) was performed using 0.2 mm thick precoated silica gel plates (Merck KGaA 60 F_254_). Compounds were visualized under ultraviolet light (UV, λ = 254 nm) and by staining with different reagents including iodine vapor, potassium permanganate aq solution (0.05 M), *p*-anisaldehyde solution (*p*-anisaldehyde/concd aq H_2_SO_4_/H_2_O/acetic acid = 3:2:50:40, v/v), ninhydrin solution (0.2% w/v ninhydrin in ethanol), or Dragendorff’s reagent: bismuth subnitrate (1.7 g), acetic acid (20 mL), water (80 mL), and 50% w/v solution of potassium iodide in water (100 mL) were mixed and stored as a stock solution. The stock solution (10 mL) and acetic acid (20 mL) were mixed and made up to 100 mL with water to give Dragendorff’s reagent.

All the products after purification by chromatography were detected by TLC (UV, λ = 254 nm, and staining with at least two reagents) showing a single spot, and the residual solvents were removed under high vacuum for ∼14 h, then the NMR data of the products were recorded.

#### 4.1.3 Synthesis and Structural Identification

##### 4.1.3.1 Ethyl 2-Oxocyclohexane-1-carboxylate (**6**, Keto)

δ_H_ (500 MHz; CDCl_3_; calibrated with TMS): 1.28 (3H, t, *J* = 7.2 Hz, OCH_2_*CH*_*3*_), 1.68 (1H, m, 5-H_A_), 1.84 (1H, m, 4-H_A_), 1.87 (1H, m, 5-H_B_), 1.97 (1H, m, 4-H_B_), 2.11 (1H, m, 6-H_A_), 2.16 (1H, m, 6-H_B_), 2.37 (1H, ddd, *J* = 14.9, 10.3, 5.5 Hz, 3-H_A_), 2.51 (1H, dt, *J* = 12.1, 5.5 Hz, 3-H_B_), 3.37 (1H, dd, *J* = 9.7, 5.8 Hz, 1-H) and 4.21 (2H, m, O*CH*_*2*_CH_3_); δ_C_ (125 MHz; CDCl_3_; calibrated with TMS): 14.17 (CH_3_, OCH_2_*CH*_*3*_), 23.31 (CH_2_, C5), 27.12 (CH_2_, C4), 29.98 (CH_2_, C6), 41.57 (CH_2_, C3), 57.25 (CH, C1), 61.09 (CH_2_, O*CH*_*2*_CH_3_), 170.02 (quat, *C*OOEt) and 206.32 (quat, C2).

##### 4.1.3.2 Ethyl 2-Hydroxycyclohex-1-ene-1-carboxylate (**7**, Enol)

δ_H_ (500 MHz; CDCl_3_; calibrated with TMS): 1.0 (3H, t, *J* = 7.1 Hz, OCH_2_*CH*_*3*_), 1.60 (2H, quin, *J* = 6.0 Hz, 5-H), 1.68 (2H, quin, *J* = 6.0 Hz, 4-H), 2.22 (2H, t, *J* = 6.3 Hz, 6-H), 2.27 (2H, t, *J* = 6.3 Hz, 3-H), 4.21 (2H, q, *J* = 7.1 Hz, O*CH*_*2*_CH_3_) and 12.25 (1H, s, O*H*); δ_C_ (125 MHz; CDCl_3_; calibrated with TMS): 14.32 (CH_3_, OCH_2_*CH*_*3*_), 21.97 (CH_2_, C4), 22.42 (CH_2_, C5 or C6), 22.44 (CH_2_, C5 or C6), 29.11 (CH_2_, C3), 60.15 (CH_2_, O*CH*_*2*_CH_3_), 97.79 (quat, C1), 172.01 (quat, C2) and 172.80 (quat, *C*OOEt).

##### 4.1.3.3 Ethyl 3-Ethyl-9-oxo-3-azabicyclo[3.3.1]nonane-1-carboxylate (**8**)^12^

Ethyl 2-oxocyclohexane-1-carboxylate (**6**, 50 μL, 0.30 mmol), ethylamine aq solution (66–72% w/w, 28 μL, 0.33 mmol), formaldehyde aq solution (37–40% w/w, 54 μL, 0.66 mmol), and EtOH (2 mL) were mixed with stirring at 20 °C. The solution was then heated under reflux for 2 h when TLC monitoring showed the reaction was complete (petroleum ether/ethyl acetate = 20:1 v/v, target compound *R*_f_ = 0.35, stained with *p*-anisaldehyde solution, or iodine vapor). After the solvent was removed by evaporation, the crude product was purified by chromatography over silica gel (petroleum ether/ethyl acetate = 20:1 v/v; mobile phase was basified concd aq 0.880 ammonia, 0.5% v/v of the prepared mobile phase) giving pale yellow oil (**8**, 60 mg, 79%). MS (*m*/*z*): [M + H]^+^ found 240.1603, C_13_H_22_NO_3_ requires 240.1600 and [M + Na]^+^ found 262.1410, C_13_H_21_NO_3_Na requires 262.1419 and [2M + Na]^+^ found 501.3066, C_26_H_42_N_2_O_6_Na requires 501.2941. δ_H_ (500 MHz; CDCl_3_; calibrated with TMS): 1.10 (3H, t, *J* = 7.2 Hz, NCH_2_*CH*_*3*_), 1.29 (3H, t, *J* = 7.2 Hz, OCH_2_*CH*_*3*_), 1.53 (1H, dtt, *J* = 12.4, 6.1, 3.0 Hz, 7-H_e_), 2.08 (1H, ddddd, *J* = 13.9, 12.4, 6.1, 5.3, 1.7 Hz, 6-H_a_), 2.14 (1H, ddt, *J* = 13.9, 5.8, 3.0 Hz, 6-H_e_), 2.24 (1H, ddt, *J* = 14.0, 6.1, 3.0 Hz, 8-H_e_), 2.41 (2H, qd, *J* = 7.2, 2.4 Hz, N*CH*_*2*_CH_3_), 2.46 (1H, dq, *J* = 5.3, 3.0 Hz, 5-H), 2.53 (1H, dddd, *J* = 14.0, 12.4, 6.1, 1.7 Hz, 8-H_a_), 2.57 (1H, br ddd, *J* = 11.1, 3.0, 1.3 Hz, 4-H_a_), 2.86 (1H, qt, *J* = 12.4, 6.1 Hz, 7-H_a_), 2.93 (1H, dd, *J* = 11.4, 1.7 Hz, 2-H_a_), 3.15 (1H, dt, *J* = 11.1, 3.0 Hz, 4-H_e_), 3.22 (1H, dd, *J* = 11.4, 2.4 Hz, 2-H_e_) and 4.21 (2H, q, *J* = 7.2 Hz, O*CH*_*2*_CH_3_). δ_C_ (125 MHz; CDCl_3_; calibrated with TMS): 12.73 (CH_3_, NCH_2_*CH*_*3*_), 14.15 (CH_3_, OCH_2_*CH*_*3*_), 20.56 (CH_2_, C7), 34.19 (CH_2_, C6), 36.84 (CH_2_, C8), 47.23 (CH, C5), 51.12 (CH_2_, N*CH*_*2*_CH_3_), 58.84 (quat, C1), 59.94 (CH_2_, C4), 61.10 (CH_2_, O*CH*_*2*_CH_3_), 61.67 (CH_2_, C2), 171.24 (quat, *C*OOEt) and 212.83 (quat, C9). δ_H_ (500 MHz; CD_3_OD; calibrated with TMS): 1.11 (3H, t, *J* = 7.2 Hz, NCH_2_*CH*_*3*_), 1.26 (3H, t, *J* = 7.1 Hz, OCH_2_*CH*_*3*_), 1.52 (1H, dtt, *J* = 12.4, 6.1, 3.1 Hz, 7-H_e_), 2.03 (1H, m, 6-H_a_), 2.18 (1H, m, 6-H_e_), 2.24 (1H, m, 8-H_e_), 2.40 (1H, m, 5-H), 2.41 (2H, m, N*CH*_*2*_CH_3_), 2.48 (1H, m, 8-H_a_), 2.52 (1H, m, 4-H_a_), 2.88 (1H, m, 7-H_a_), 2.90 (1H, m, 2-H_a_), 3.23 (1H, m, 4-H_e_), 3.23 (1H, m, 2-H_e_) and 4.16 (2H, q, *J* = 7.2 Hz, O*CH*_*2*_CH_3_). δ_C_ (125 MHz; CD_3_OD; calibrated with TMS): 13.05 (CH_3_, NCH_2_*CH*_*3*_), 14.45 (CH_3_, OCH_2_*CH*_*3*_), 21.52 (CH_2_, C7), 35.32 (CH_2_, C6), 37.99 (CH_2_, C8), 48.71 (CH, C5), 52.20 (CH_2_, N*CH*_*2*_CH_3_), 60.26 (quat, C1), 61.00 (CH_2_, C4), 62.20 (CH_2_, O*CH*_*2*_CH_3_), 62.88 (CH_2_, C2), 172.66 (quat, *C*OOEt) and 214.64 (quat, C9). δ_H_ [400 MHz; *d*_6_-DMSO; calibrated with residual DMSO (2.50 ppm)]: 1.04 (3H, t, *J* = 7.2 Hz, NCH_2_*CH*_*3*_), 1.18 (3H, t, *J* = 7.2 Hz, OCH_2_*CH*_*3*_), 1.45 (1H, m, 1H, dtt, *J* = 12.1, 5.9, 2.7 Hz, 7-H_e_), 1.95 (1H, m, 6-H_a_), 2.10 (1H, ddq, *J* = 13.7, 5.5, 2.7 Hz, 6-H_e_), 2.18 (1H, dq, *J* = 13.7, 2.7 Hz, 8-H_e_), 2.35 (2H, m, N*CH*_*2*_CH_3_), 2.37 (1H, m, 8-H_a_), 2.37 (1H, m, 5-H), 2.45 (1H, br dd, *J* = 11.1, 3.2 Hz, 4-H_a_), 2.77 (1H, qt, *J* = 12.1, 5.9 Hz, 7-H_a_), 2.81 (1H, dd, *J* = 11.5, 1.5 Hz, 2-H_a_), 3.16 (1H, dt, *J* = 11.2, 2.2 Hz, 4-H_e_), 3.22 (1H, dd, *J* = 2.2, 11.4 Hz, 2-H_e_) and 4.21 (2H, qd, *J* = 7.2, 1.1 Hz, O*CH*_*2*_CH_3_). δ_C_ [100 MHz; *d*_6_-DMSO; calibrated with *d*_6_-DMSO (39.52 ppm)]: 12.51 (CH_3_, NCH_2_*CH*_*3*_), 14.01 (CH_3_, OCH_2_*CH*_*3*_), 19.97 (CH_2_, C7), 33.57 (CH_2_, C6), 36.12 (CH_2_, C8), 46.46 (CH, C5), 50.49 (CH_2_, N*CH*_*2*_CH_3_), 58.27 (quat, C1), 59.21 (CH_2_, C4), 60.48 (CH_2_, O*CH*_*2*_CH_3_), 61.10 (CH_2_, C2), 170.46 (quat, *C*OOEt) and 212.16 (quat, C9).

##### 4.1.3.4 Ethyl 3-Ethyl-7,7-dimethyl-9-oxo-3-azabicyclo[3.3.1]nonane-1-carboxylate (**10**)

Methyl 5,5-dimethyl-2-oxocyclohexane-1-carboxylate (**9**, 50 μL, 0.29 mmol), ethylamine aq solution (66–72% w/w, 27 μL, 0.32 mmol), formaldehyde aq solution (37–40% w/w, 52 μL, 0.64 mmol), and MeOH (2 mL) were mixed together successively with stirring. The round-bottomed flask with reactants was heated and stirred under reflux on a heating mantle for 2 h under an atmosphere of anhydrous nitrogen. TLC showed the reaction was complete (petroleum spirit/ethyl acetate = 2:1 v/v; the mobile phase was basified with concd aq 0.880 ammonia, 0.5% v/v of the prepared mobile phase; target compound *R*_f_ = 0.24, stained with potassium permanganate solution). After the solvents were removed by evaporation, the products were acidified with aq HCl (1 M) to pH = 2 followed by washing with dichloromethane (DCM) (3 × 5 mL), then the aqueous layer was basified with aq NaHCO_3_ (pH = 8) and extracted with DCM (3 × 5 mL). The combined organic phase was dried (Na_2_SO_4_), filtered, and then the solvents were removed by evaporation. The crude product was purified by chromatography over silica gel (petroleum ether/ethyl acetate = 30:1 → petroleum ether/ethyl acetate = 20:1 v/v; the mobile phase was basified with concd aq 0.880 ammonia, 0.5% v/v of the prepared mobile phase), and finally pure colorless oil (**10**, 10 mg, 14%) was obtained. MS (*m*/*z*): [M + Na]^+^ found 276.1559, C_14_H_23_NO_3_Na requires 276.1576. δ_H_ [500 MHz; CDCl_3_; calibrated with residual CHCl_3_ (7.26 ppm)]: 0.94 (3H, s, 2′-H), 1.00 (3H, s, 1′-H), 1.08 (3H, t, *J* = 7.2 Hz, NCH_2_*CH*_*3*_), 1.89 (1H, ddd, *J* = 13.3, 10.5, 2.8 Hz, 6-H_e_), 2.12 (1H, dd, *J* = 13.3, 2.2 Hz, 6-H_a_), 2.17 (1H, dd, *J* = 13.1, 2.8 Hz, 8-H_e_), 2.37 (1H, m, 4-H_a_), 2.40 (1H, m, 8-H_a_), 2.45 (1H, dq, *J* = 10.5, 2.8 Hz, 5-H), 2.52 (2H, q, *J* = 7.2 Hz, N*CH*_*2*_CH_3_), 2.62 (1H, d, *J* = 11.0 Hz, 2-H_a_), 2.93 (1H, dd, *J* = 10.5, 2.8 Hz, 4-H_e_), 3.02 (1H, dd, *J* = 11.0, 2.8 Hz, 2-H_e_) and 3.74 (3H, s, OCH_3_). δ_C_ [125 MHz; CDCl_3_; calibrated with CDCl_3_ (77.16 ppm)]: 12.68 (CH_3_, NCH_2_*CH*_*3*_), 27.83 (CH_3_, C2′), 29.23 (quat, C7), 31.83 (CH_3_, C2′), 44.53 (CH_2_, C6), 44.80 (CH, C5), 47.26 (CH_2_, C8), 50.31 (CH_2_, N*CH*_*2*_CH_3_), 52.34 (CH_3_, OCH_3_), 57.84 (quat, C1), 62.59 (CH_2_, C4), 63.74 (CH_2_, C2), 172.75 (quat, *C*OOMe) and 216.33 (quat, C9).

##### 4.1.3.5 Methyl 3-Ethyl-7-isopropyl-9-oxo-3-azabicyclo[3.3.1]nonane-1-carboxylate (**12**)

A solution of methyl 5-isopropyl-2-oxocyclohexane-1-carboxylate (**11**, 420 mg, 2.12 mmol), ethylamine aq solution (66–72% w/w, 0.20 mL, 2.33 mmol), and paraformaldehyde (140 mg, 4.66 mmol) in MeOH (16 mL) was stirred and heated under reflux for 2 h under an atmosphere of anhydrous nitrogen. TLC showed the reaction was complete (petroleum ether/ethyl acetate = 20:1 v/v, *R*_f_ = 0.3, stained with *p*-anisaldehyde solution). After the solvents were removed by evaporation, the crude products were acidified with aq HCl (1 M) to pH = 2 followed by washing with DCM (3 × 5 mL), then the aqueous layer was basified with a sat. NaHCO_3_ aq solution (pH = 8) and extracted with DCM (3 × 5 mL). The combined organic phase was dried (Na_2_SO_4_), filtered, and then the solvents were removed by evaporation. The crude product was purified by chromatography over silica gel (petroleum ether/ethyl acetate = 20:1 v/v; the mobile phase was basified with concd aq 0.880 ammonia, 0.5% v/v of the prepared mobile phase) producing colorless oil (**12**, 63 mg, 11%). MS (*m*/*z*): [M + H]^+^ found 268.1913, C_15_H_26_NO_3_ requires 268.1913 and [M + Na]^+^ found 290.1723, C_15_H_25_NO_3_Na requires 290.1732. δ_H_ (500 MHz; CDCl_3_; calibrated with TMS): 0.89 (3H, d, *J* = 6.1 Hz, 2′-H_A_), 0.90 (3H, d, *J* = 6.1 Hz, 2′-H_B_), 1.10 (3H, t, *J* = 7.2 Hz, NCH_2_*CH*_*3*_), 1.28 (1H, m, 1′-H), 1.72 (1H, td, *J* = 12.9, 6.0 Hz, 6-H_a_), 2.13–2.29 (3H, m, 6-H_e_, 8-H_a_ and 8-H_e_), 2.41 (2H, q, *J* = 7.2 Hz, N*CH*_*2*_CH_3_), 2.45 (1H, br quin, *J* = 2.6 Hz, 5-H), 2.55 (1H, br dd, *J* = 11.2, 2.4 Hz, 4-H_a_), 2.93 (1H, br dd, *J* = 11.2, 2.5 Hz, 2-H_a_), 2.93 (1H, br dd, *J* = 11.4, 1.9 Hz, 2-H_a_), 3.02 (1H, tq, *J* = 12.4, 6.0 Hz, 7-H_a_), 3.14 (1H, br td, *J* = 11.2, 2.4 Hz, 4-H_e_), 3.22 (1H, dd, *J* = 11.4, 2.5 Hz, 2-H_e_) and 3.75 (3H, s, OCH_3_). δ_C_ (125 MHz; CDCl_3_; calibrated with TMS): 12.69 (CH_3_, NCH_2_*CH*_*3*_), 20.24 (CH_3_, C2_A_^′^), 22.38 (CH3, C2_B_^′^), 33.76 (CH, C1′), 37.01 (CH, C7), 38.35 (CH_2_, C6), 41.17 (CH_2_, C8), 47.08 (CH, C5), 51.04 (CH_2_, N*CH*_*2*_CH_3_), 52.25 (CH_3_, OCH_3_), 58.66 (quat, C1), 59.75 (CH_2_, C4), 61.59 (CH_2_, C2), 171.67 (quat, *C*OOMe) and 213.10 (quat, C9).

##### 4.1.3.6 Ethyl 3-Ethyl-7-methyl-9-oxo-3-azabicyclo[3.3.1]nonane-1-carboxylate (**14**)

A solution of methyl 5-isopropyl-2-oxocyclohexane-1-carboxylate (**13**, 200 mg, 1.09 mmol), ethylamine aq solution (66–72% w/w, 0.10 mL, 1.20 mmol), and paraformaldehyde (173 mg, 2.40 mmol) in EtOH (8 mL) was stirred and heated under reflux for 2 h under an atmosphere of anhydrous nitrogen. TLC showed the reaction was complete (petroleum ether/ethyl acetate = 20:1 v/v; the mobile phase was basified with concd aq 0.880 ammonia, 0.5% v/v of the prepared mobile phase; target compound *R*_f_ = 0.25, stained with potassium permanganate solution). After the solvents were removed by evaporation, the products were acidified with aq HCl (1 M) to pH = 2 followed by washing with DCM (3 × 20 mL), then aqueous layer was basified with a sat. NaHCO_3_ aq solution (pH = 8) and extracted with DCM (3 × 20 mL). The combined organic phase was dried (Na_2_SO_4_), filtered, and then the solvents were removed by evaporation. The crude product was purified by chromatography over silica gel (petroleum ether/ethyl acetate, 20:1 v/v; the mobile phase was basified with concd aq 0.880 ammonia, 0.5% v/v of the prepared mobile phase) producing yellow oil (**14**, 11 mg, 4%). MS (*m*/*z*): [M + H]^+^ found 254.1752, C_14_H_24_NO_3_ requires 254.1757 and [M + Na]^+^ found 276.1573, C_14_H_23_NO_3_Na requires 276.1576. δ_H_ (500 MHz; CDCl_3_; calibrated with TMS): 0.88 (3H, d, *J* = 6.7 Hz, 1′-H), 1.10 (3H, t, *J* = 7.2 Hz, NCH_2_*CH*_*3*_), 1.29 (3H, t, *J* = 7.2 Hz, OCH_2_*CH*_*3*_), 1.68 (1H, m, 6-H_a_), 2.09–2.24 (3H, m, 6-H_e_, 8-H_a_ and 8-H_e_), 2.40 (2H, m, N*CH*_*2*_CH_3_), 2.42 (1H, m, 5-H), 2.54 (1H, d, *J* = 11.2 Hz, 4-H_a_), 2.93 (1H, dd, *J* = 11.5, 2.0 Hz, 2-H_a_), 3.16 (1H, dt, *J* = 11.2, 2.0 Hz, 4-H_e_), 3.23 (1H, dt, *J* = 11.5, 2.0 Hz, 2-H_e_), 3.42 (1H, tq, *J* = 12.3, 6.1 Hz, 7-H_a_) and 4.21 (2H, q, *J* = 7.2 Hz, O*CH*_*2*_CH_3_). δ_C_ (125 MHz; CDCl_3_; calibrated with TMS): 12.73 (CH_3_, NCH_2_*CH*_*3*_), 14.15 (CH_3_, OCH_2_*CH*_*3*_), 22.01 (CH_3_, C1′), 26.28 (CH, C7), 42.56 (CH_2_, C6), 44.99 (CH_2_, C8), 47.30 (CH, C5), 51.13 (CH_2_, N*CH2*CH3), 58.54 (quat, C1), 59.65 (CH_2_, C4), 61.14 (CH_2_, O*CH*_*2*_CH_3_), 61.46 (CH_2_, C2), 171.03 (quat, *C*OOEt) and 212.93 (quat, C9).

##### 4.1.3.7 *N*,*N*-Bis(ethoxymethyl)ethanamine (**16**)^19^

Freshly distilled ethylamine (14.2 g, 315 mmol) was slowly added to a solution of pre-dried paraformaldehyde (18.9 g, 630 mmol) and oven-dried potassium carbonate (43.5 g, 315 mmol) in anhydrous ethanol (120 mL) at 0 °C with magnetic stirring, and the mixture was stirred vigorously for 2 days at 20 °C. The suspension was filtered, and the residue was rinsed with anhydrous ethanol (20 mL). The crude product solution was first purified by fractional vacuum distillation through a Vigreux column at 90–130 °C, 400 mbar, mainly in order to remove the excess of alcohol and then the Vigreux column was removed and the products were further purified by fractional vacuum distillation (130–150 °C, 100–110 mbar) followed by collecting the fraction with a boiling point of 100–105 °C, forming the clear liquid product amine (**16**, 21.3 g, 41%). δ_H_ (500 MHz; CDCl_3_; calibrated with TMS): 1.11 (3H, t, *J* = 7.2 Hz, NCH_2_*CH*_*3*_), 1.19 (3H × 2, t, *J* = 7.1 Hz, OCH_2_*CH*_*3*_ × 2), 2.89 (2H, q, *J* = 7.2 Hz, N*CH*_*2*_CH_3_), 3.44 (2H × 2, q, *J* = 7.1 Hz, O*CH*_*2*_CH_3_ × 2) and 4.30 (2H × 2, s, NCH_2_O × 2). δ_C_ (125 MHz; CDCl_3_; calibrated with TMS): 13.69 (CH_3_, NCH_2_*CH*_*3*_), 15.25 (CH_3_ × 2, OCH_2_*CH*_*3*_ × 2), 43.81 (CH_2_, N*CH*_*2*_CH_3_), 62.58 (CH_2_ × 2, O*CH*_*2*_CH_3_ × 2) and 84.27 (CH_2_ × 2, N*CH*_*2*_O × 2).

##### 4.1.3.8 Methyl 3-Ethyl-7-methyl-9-oxo-3-azabicyclo[3.3.1]nonane-1-carboxylate (**17**)^19^

To a solution of methyl 2-oxocyclopentane-1-carboxylate (**15**, 94 mg, 0.55 mmol) and *N*,*N*-bis(ethoxymethyl)ethanamine (**16**, 179 mg, 1.10 mmol) in acetonitrile (2 mL), trichloromethylsilane (217 mg, 1.11 mmol) was added followed by stirring for 20 h at 20 °C. The reaction was quenched with sat. aq NaHCO_3_ (pH = 8) and extracted with ethyl acetate (3 × 10 mL). The combined organic extracts were washed with sat. aq brine (10 mL), dried (Na_2_SO_4_), and filtered, and then the solvents were removed by evaporation. The crude products were purified by chromatography over silica gel (petroleum ether/ethyl acetate = 20:1 v/v; the mobile phase was basified with concd aq 0.880 ammonia, 0.5% v/v of the prepared mobile phase; target compound TLC *R*_f_ = 0.25, stained with Dragendorff’s reagent and *p*-anisaldehyde solution) giving a yellow oil product (**17**, 26 mg, 73%). MS (*m*/*z*): [M + H]^+^ found 240.1618, C_13_H_22_NO_3_ requires 240.1594 and [M + Na]^+^ found 262.1448, C_13_H_21_NO_3_Na requires 262.1414. δ_H_ [500 MHz; CDCl_3_; calibrated with residual CHCl_3_ (7.26 ppm)]: 0.87 (3H, d, *J* = 6.7 Hz, 1′-H), 1.09 (3H, t, *J* = 7.2 Hz, NCH_2_*CH*_*3*_), 1.66 (1H, tdd, *J* = 13.8, 6.0, 1.5 Hz, 6-H_a_), 2.09–2.23 (3H, m, 6-H_e_, 8-H_a_ and 8-H_e_), 2.39 (2H, qd, *J* = 7.2, 2.0 Hz, N*CH*_*2*_CH), 2.42 (1H, m, 5-H), 2.54 (1H, m, 4-H_a_), 2.92 (1H, dd, *J* = 11.4, 2.1 Hz, 2-H_a_), 3.15 (1H, dt, *J* = 11.2, 2.1 Hz, 4-H_e_), 3.23 (1H, dt, *J* = 11.4, 2.1 Hz, 2-H_e_), 3.42 (1H, tt, *J* = 12.3, 6.0 Hz, 7-H_a_) and 3.74 (3H, s, OCH_3_). δ_C_ [125 MHz; CDCl_3_; calibrated with CDCl_3_ (77.16 ppm)]: 12.80 (CH_3_, NCH_2_*CH*_*3*_), 22.11 (CH_3_, C1′), 26.40 (CH, C7), 42.70 (CH_2_, C6), 45.18 (CH_2_, C8), 47.39 (CH, C5), 51.25 (CH_2_, N*CH2*CH3), 52.34 (CH_3_, OCH_3_), 58.90 (quat, C1), 59.75 (CH_2_, C4), 61.59 (CH_2_, C2), 171.60 (quat, *C*OOMe) and 212.93 (quat, C9).

##### 4.1.3.9 Ethyl (*E*)-9-(2-(2,4-Dinitrophenyl)hydrazinylidene)-3-ethyl-3-azabicyclo[3.3.1]nonane-1-carboxylate (**18**)^31^

2,4-Dinitrophenylhydrazine (412 mg, 2.08 mmol) and trifluoroacetic acid (TFA) (119 mg, 1.04 mmol) were added to a solution of ethyl 3-ethyl-9-oxo-3-azabicyclo[3.3.1]nonane-1-carboxylate (**8**, 415 mg, 1.73 mmol) in tetrahydrofuran (THF, 12 mL), and the resulting solution was stirred and heated under reflux for 3 h under an atmosphere of anhydrous nitrogen. After the solution was cooled to 20 °C, about 7 mL of the solvent was removed by evaporation. The residue was basified with sat. aq NaHCO_3_ (pH = 8) and extracted with DCM (3 × 15 mL), and then the combined organic layers were washed with sat. aq brine (10 mL), dried (Na_2_SO_4_), and filtered, and then the solvents were removed by evaporation. The crude product was purified by chromatography over silica gel (petroleum ether/ethyl acetate 10:1 v/v; the mobile phase was basified with concd aq 0.880 ammonia, 0.5% v/v of the prepared mobile phase; target compound TLC *R*_f_ = 0.3, stained with iodine vapor or ninhydrin solution) giving an orange solid product (**18**, 472 mg, 65%). MS (*m*/*z*): [M + H]^+^ found 420.1934, C_19_H_26_N_5_O_6_ requires 420.1878 and [M + Na]^+^ found 442.1729, C_19_H_25_N_5_O_6_Na requires 442.1697. δ_H_ (500 MHz; CDCl_3_; calibrated with TMS): 1.11 (3H, t, *J* = 7.2 Hz, NCH_2_*CH*_*3*_), 1.36 (3H, t, *J* = 7.2 Hz, OCH_2_*CH*_*3*_), 1.58 (1H, m, 7-H_e_), 1.93 (1H, tt, *J* = 12.2, 6.0 Hz, 6-H_a_), 2.14 (1H, m, 6-H_e_), 2.16 (1H, m, 8-H_e_), 2.40 (2H, q, *J* = 7.2 Hz, N*CH*_*2*_CH_3_), 2.42 (1H, m, 4-H_a_), 2.49 (1H, *J* = 12.9, 6.0, 1.1 Hz, 8-H_a_), 2.89 (2H, m, 7-H_a_ and 2-H_a_), 3.11 (1H, m, 5-H), 3.16 (1H, m, 4-H_e_), 3.19 (1H, m, 2-H_e_), 4.30 (2H, qd, *J* = 7.1, 1.9 Hz, O*CH*_*2*_CH_3_), 7.80 (1H, d, *J* = 9.5 Hz, 6′-H), 8.28 (1H, dd, *J* = 9.5, 2.6 Hz, 5′-H), 9.11 (1H, d, *J* = 2.6 Hz, 3′-H) and 11.17 (1H, s, NH). δ_C_ (125 MHz; CDCl_3_; calibrated with TMS): 12.54 (CH_3_, NCH_2_*CH*_*3*_), 14.37 (CH_3_, OCH_2_*CH*_*3*_), 20.85 (CH_2_, C7), 32.45 (CH_2_, C6), 33.22 (CH, C5), 36.38 (CH_2_, C8), 51.54 (CH_2_, N*CH*_*2*_CH_3_), 52.34 (quat, C1), 58.42 (CH_2_, C4), 61.13 (CH_2_, O*CH*_*2*_CH_3_), 61.82 (CH_2_, C2), 116.20 (CH, C6′), 123.48 (CH, C3′), 129.10 (quat, C2′), 129.97 (CH, C5′), 137.88 (quat, C4′), 145.40 (quat, C1′), 163.86 (quat, C9) and 172.12 (quat, *C*OOEt).

##### 4.1.3.10 Methyl (*E*)-9-(2-(2,4-Dinitrophenyl)hydrazinylidene)-3-ethyl-7-isopropyl-3-azabicyclo[3.3.1]nonane-1-carboxylate (**19**)

2,4-Dinitrophenylhydrazine (55 mg, 0.19 mmol) and TFA (11 mg, 0.10 mmol) were added to a solution of methyl 3-ethyl-7-isopropyl-9-oxo-3-azabicyclo[3.3.1]nonane-1-carboxylate (**12**, 43 mg, 0.16 mmol) in THF (3 mL), and the resulting solution was stirred and heated under reflux for 3 h under an atmosphere of anhydrous nitrogen. The solution was basified with sat. aq NaHCO_3_ (pH = 8) and extracted with DCM (3 × 10 mL), and then the combined organic layers were washed with sat. aq brine (5 mL), dried (Na_2_SO_4_), and filtered, and then the solvents were removed by evaporation. The crude product was purified by chromatography over silica gel (petroleum ether/ethyl acetate 10:1 v/v; the mobile phase was basified with concd aq 0.880 ammonia, 0.5% v/v of the prepared mobile phase; target compound TLC *R*_f_ = 0.2, stained with iodine vapor or ninhydrin solution) giving an orange solid product (**19**, 44 mg, 61%). MS (*m*/*z*): [M + H]^+^ found 448.2206, C_21_H_30_N_5_O_6_ requires 448.2191 and [M + Na]^+^ found 470.2026, C_21_H_29_N_5_O_6_Na requires 470.2010. δ_H_ (500 MHz; CDCl_3_; calibrated with TMS): 0.89 (3H, d, *J* = 6.1 Hz, 2′-H_A_), 0.91 (3H, d, *J* = 6.1 Hz, 2′-H_B_), 1.10 (3H, t, *J* = 7.2 Hz, NCH_2_*CH*_*3*_), 1.26 (1H, m, 1′-H), 1.93 (1H, td, *J* = 13.4, 4.1 Hz, 6-H_a_), 2.13 (1H, m, 8-H_a_), 2.19 (1H, m, 6-H_e_), 2.20 (1H, m, 8-H_e_), 2.40 (3H, m, 4-H_a_ and N*CH*_*2*_CH_3_), 2.89 (1H, br d, *J* = 11.3 Hz, 2-H_a_), 2.97 (1H, m, 7-H_a_), 3.12 (1H, m, 5-H), 3.14 (1H, m, 4-H_e_), 3.18 (1H, m, 2-H_e_), 3.84 (3H, s, OCH_3_), 7.77 (1H, d, *J* = 9.6 Hz, 6″-H), 8.29 (1H, dd, *J* = 9.6, 2.5 Hz, 5″-H), 9.12 (1H, d, *J* = 2.5 Hz, 3″-H) and 11.16 (1H, s, NH). δ_C_ (125 MHz; CDCl_3_; calibrated with TMS): 12.48 (CH_3_, NCH_2_*CH*_*3*_), 20.18 (CH_3_, C2_A_^′^), 20.28 (CH_3_, C2_B_^′^), 33.33 (CH, C5), 34.12 (CH, C1′), 36.67 (CH_2_, C6), 37.43 (CH, C7), 40.68 (CH_2_, C8), 51.35 (CH_2_, N*CH*_*2*_CH_3_), 52.22 (CH_3_, OCH_3_), 52.54 (quat, C1), 58.23 (CH_2_, C4), 61.65 (CH_2_, C2), 116.16 (CH, C6″), 123.46 (CH, C3″), 129.14 (quat, C2″), 130.05 (CH, C5″), 137.92 (quat, C4″), 145.34 (quat, C1″), 163.83 (quat, C9) and 172.57 (quat, *C*OOMe).

##### 4.1.3.11 Methyl (*E*)-9-(2-(2,4-Dinitrophenyl)hydrazinylidene)-3-ethyl-7-methyl-3-azabicyclo[3.3.1]nonane-1-carboxylate (**20**)^32^

2,4-Dinitrophenylhydrazine (91 mg, 0.46 mmol) and concd aq H_2_SO_4_ solution (15 μL, 0.10 mmol) were added to a solution of methyl 3-ethyl-7-methyl-9-oxo-3-azabicyclo[3.3.1]nonane-1-carboxylate (**17**, 22 mg, 0.09 mmol) in MeOH (3 mL) at 0 °C, and the resulting solution was stirred and heated under reflux for 3 h under an atmosphere of anhydrous nitrogen. The solution was basified with sat. aq NaHCO_3_ (pH = 8) and extracted with DCM (3 × 10 mL), and then the combined organic layers were washed with sat. aq brine (5 mL), dried (Na_2_SO_4_), and filtered, and then the solvents were removed by evaporation. The crude product was purified by chromatography over silica gel (petroleum ether/ethyl acetate = 20:1 v/v; the mobile phase was basified with concd aq 0.880 ammonia, 0.5% v/v of the prepared mobile phase; target compound TLC *R*_f_ = 0.32, stained with iodine vapor or ninhydrin solution) giving an orange solid product (**20**, 20 mg, 53%). MS (*m*/*z*): [M + H]^+^ found 420.1902, C_19_H_26_N_5_O_6_ requires 420.1878 and [M + Na]^+^ found 442.1720, C_19_H_25_N_5_O_6_Na requires 442.1697. δ_H_ (500 MHz; CDCl_3_; calibrated with TMS): 0.81 (3H, d, *J* = 6.6 Hz, 1′-H), 1.13 (3H, t, *J* = 7.1 Hz, NCH_2_*CH*_*3*_), 1.42 (1H, m, 6-H_a_), 2.01 (1H, m, 8-H_a_), 2.08 (1H, m, 6-H_e_), 2.10 (1H, m, 8-H_e_), 2.32 (1H, m, 4-H_a_), 2.33 (2H, m, N*CH*_*2*_CH_3_), 2.82 (1H, br d, *J* = 11.3 Hz, 2-H_a_), 3.03 (1H, m, 5-H), 3.09 (1H, d, *J* = 11.2 Hz, 4-H_e_), 3.13 (1H, d, *J* = 11.4 Hz, 2-H_e_), 3.28 (1H, tq, *J* = 12.2, 6.1 Hz, 7-H_a_), 3.76 (3H, s, OCH_3_), 7.70 (1H, d, *J* = 9.5 Hz, 6″-H), 8.22 (1H, d, *J* = 9.5 Hz, 5″-H), 9.03 (1H, d, *J* = 2.5 Hz, 3″-H) and 11.08 (1H, s, NH). δ_C_ [125 MHz; CDCl_3_; calibrated with CDCl_3_ (77.16 ppm)]: 12.60 (CH_3_, NCH_2_*CH*_*3*_), 22.49 (CH_3_, C1′), 26.71 (CH, C7), 33.57 (CH, C5), 40.94 (CH_2_, C6), 44.78 (CH_2_, C8), 51.56 (CH_2_, N*CH*_*2*_CH_3_), 52.32 (CH_3_, OCH_3_), 52.74 (quat, C1), 58.19 (CH_2_, C4), 61.59 (CH_2_, C2), 116.26 (CH, C6″), 123.56 (CH, C3″), 129.26 (quat, C2″), 130.17 (CH, C5″), 138.04 (quat, C4″), 145.45 (quat, C1″), 163.70 (quat, C9) and 172.54 (quat, *C*OOMe).

An orange solid (42 mg in total) was obtained after purification, and there were three main components in both ^1^H and ^13^C NMR spectra, which are the target product, the conformational isomer of the target product, and the byproduct, 1-(2,4-dinitrophenyl)-2-(propan-2-ylidene)hydrazine derived from acetone. The ratio of ^1^H integrals of these components was 20:4:17; therefore, the yield is 53%. The total content of the isomer was too low to be analyzed in detail using NMR spectroscopy. These products were therefore not purified further after the purification by flash chromatography over silica gel.

##### 4.1.3.12 1-(2,4-Dinitrophenyl)-2-(propan-2-ylidene)hydrazine

MS (*m*/*z*): [M – H]^−^ found 237.0614, C_9_H_9_N_4_O_4_ requires 237.0623, and MS (*m*/*z*): [M + HCOO]^−^ found 283.0669, C_10_H_11_N_4_O_6_ requires 283.0679. δ_H_ (500 MHz; CDCl_3_; calibrated with TMS): 2.02 (3H, s, 2′-H), 2.11 (3H, s, 3′-H), 7.89 (1H, d, *J* = 7.1 Hz, 6″-H), 8.22 (1H, m, 5″-H), 9.05 (1H, d, *J* = 2.4 Hz, 3″-H) and 10.95 (1H, br s, NH). δ_C_ [125 MHz; CDCl_3_; calibrated with CDCl_3_ (77.16 ppm)]: 17.17 (CH_2_, C2′), 25.64 (CH_2_, C3′), 116.49 (CH, C6), 123.69 (CH, C3), 129.07 (quat, C2), 130.12 (CH, C5), 137.76 (quat, C4), 145.27 (quat, C1) and 155.36 (quat, C1′).

##### 4.1.3.13 Methyl 8-Ethyl-10-oxo-8-azabicyclo[4.3.1]decane-1-carboxylate (**23**)

A solution of methyl 5-isopropyl-2-oxocyclohexane-1-carboxylate (**21**, 511 mg, 3.01 mmol), aq ethylamine (66–72% w/w, 0.28 mL, 3.31 mmol), and formaldehyde aq solution (37–40% w/w, 0.54 mL, 6.65 mmol) in MeOH (20 mL) was stirred and heated under reflux for 4 h under an atmosphere of anhydrous nitrogen. TLC showed the reaction was complete (petroleum ether/ethyl acetate = 20:1 v/v, *R*_f_ = 0.2, stained with *p*-anisaldehyde solution). After the solvents were removed by evaporation, the crude products were acidified with aq HCl (1 M) to pH = 2 followed by washing with DCM (3 × 20 mL), then the aqueous layer was basified with a sat. NaHCO_3_ aq solution (pH = 8) and extracted with DCM (3 × 20 mL). The combined organic phase was dried (Na_2_SO_4_), filtered, and then the solvents were removed by evaporation. The crude compound was purified by column chromatography over silica gel (petroleum ether/ethyl acetate = 20:1 v/v; the mobile phase was basified with concd aq 0.880 ammonia, 0.5% v/v of the prepared mobile phase) producing colorless oil (**23**, 459 mg, 64%). MS (*m*/*z*): [M + H]^+^ found 240.1624, C_13_H_22_NO_3_ requires 240.1594 and [M + Na]^+^ found 262.1428, C_13_H_21_NO_3_Na requires 262.1414. δ_H_ (500 MHz; CDCl_3_; calibrated with TMS): 1.11 (3H, t, *J* = 7.2 Hz, NCH_2_*CH*_*3*_), 1.36 (1H, dtdd, *J* = 15.0, 10.3, 3.6, 1.4 Hz, 3-H_e′_), 1.51 (1H, m, 4-H_e′_), 1.69 (1H, m, 5-H_e′_), 1.75 (1H, m, 5-H_a′_), 1.85 (1H, ddd, *J* = 13.5, 10.5, 3.1 Hz, 2-H_e′_), 1.96 (1H, m, 4-H_a′_), 2.04 (1H, m, 3-H_a′_), 2.41 (1H, ddd, *J* = 13.5, 10.5, 3.1 Hz, 2-H_a′_), 2.47 (2H, q, *J* = 7.2 Hz, N*CH*_*2*_CH_3_), 2.58 (1H, dd, *J* = 11.5, 4.4 Hz, 7-H_a_), 2.70 (1H, quin, *J* = 3.6 Hz, 6-H), 2.83 (1H, d, *J* = 11.5 Hz, 9-H_a_), 2.88 (1H, m, 9-H_e_), 2.89 (1H, m, 7-H_e_) and 3.74 (3H, s, OCH_3_). δ_C_ (125 MHz; CDCl_3_; calibrated with TMS): 12.62 (CH_3_, NCH_2_*CH*_*3*_), 26.00 (CH_2_, C4), 26.25 (CH_2_, C3), 32.45 (CH_2_, C5), 33.54 (CH_2_, C2), 48.47 (CH, C6), 51.50 (CH_2_, N*CH*_*2*_CH_3_), 52.34 (CH_3_, OCH_3_), 58.45 (CH_2_, C7), 61.50 (CH_2_, C9), 62.09 (quat, C1), 172.95 (quat, *C*OOMe) and 208.75 (quat, C10).

##### 4.1.3.14 Methyl (*E*)-10-(2-(2,4-Dinitrophenyl)hydrazinylidene)-8-ethyl-8-azabicyclo[4.3.1]decane-1-carboxylate (**24**)

2,4-Dinitrophenylhydrazine (425 mg, 1.46 mmol) and concd aq H_2_SO_4_ solution (40 μL, 0.73 mmol) were added to a solution of methyl 8-ethyl-10-oxo-8-azabicyclo[4.3.1]decane-1-carboxylate (**23**, 70 mg, 0.29 mmol) in MeOH (8 mL) at 0 °C, and the resulting solution was stirred and heated under reflux under an atmosphere of anhydrous nitrogen. After 4.5 days, TLC monitoring showed the reactant [4.3.1]azabicycle was fully reacted and the target compound was generated (petroleum ether/ethyl acetate = 3:1 v/v, target compound TLC *R*_f_ = 0.8, stained with *p*-anisaldehyde). After the solution was cooled to 20 °C, 4 mL of the solvent was removed by evaporation. The rest of the solution was basified with sat. aq NaHCO_3_ (pH = 8) and extracted with DCM (3 × 15 mL), and the combined organic layers were washed with sat. aq brine (15 mL), dried (Na_2_SO_4_), and filtered, and then the solvents were removed by evaporation. The residue was purified by chromatography over silica gel (petroleum ether/ethyl acetate = 30:1 v/v; the mobile phase was basified with concd aq 0.880 ammonia, 0.5% v/v of the prepared mobile phase; target compound TLC *R*_f_ = 0.25, stained with iodine vapor or ninhydrin solution) giving an orange solid product (**24**, 16 mg, 13%). MS (*m*/*z*): [M + H]^+^ found 420.1903, C_19_H_26_N_5_O_6_ requires 420.1878 and [M + Na]^+^ found 442.1706, C_19_H_25_N_5_O_6_Na requires 442.1697. δ_H_ (500 MHz; CDCl_3_; calibrated with TMS): 1.11 (3H, t, *J* = 7.1 Hz, NCH_2_*CH*_*3*_), 1.39 (1H, m, 4-H_e′_), 1.42 (1H, m, 3-H_e′_), 1.79 (1H, ddd, *J* = 10.1, 7.8, 2.9 Hz, 5-H_e_), 1.91 (1H, m, 2-H_e′_), 1.95 (2H, m, 5-H_a′_ and 4-H_a′_), 2.05 (1H, m, 3-H_a′_), 2.44 (1H, m, 7-H_a_), 2.45 (2H, m, N*CH*_*2*_CH_3_), 2.51 (1H, ddd, *J* = 13.1, 8.2, 3.8 Hz, 2-H_a_), 2.78 (1H, d, *J* = 11.0 Hz, 9-H_a_), 2.86 (1H, dd, *J* = 11.0, 2.3 Hz, 9-H_e_), 2.90 (1H, br dt, *J* = 11.4, 1.9 Hz, 7-H_e_), 3.19 (1H, m, 6-H), 3.80 (3H, s, OCH_3_), 7.75 (1H, d, *J* = 9.5 Hz, 6′-H), 8.29 (1H, dd, *J* = 9.5, 2.6 Hz, 5′-H), 9.12 (1H, d, *J* = 2.6 Hz, 3′-H) and 11.24 (1H, s, NH). δ_C_ (125 MHz; CDCl_3_; calibrated with TMS): 12.41 (CH_3_, NCH_2_*CH*_*3*_), 25.83 (CH_2_, C3), 26.07 (CH_2_, C4), 30.51 (CH_2_, C5), 34.50 (CH, C6), 26.80 (CH_2_, C2), 51.74 (CH_2_, N*CH*_*2*_CH_3_), 52.32 (CH_3_, OCH_3_), 55.55 (quat, C1), 58.05 (CH_2_, C7), 61.92 (CH_2_, C9), 116.26 (CH, C6′), 123.42 (CH, C3′), 129.36 (quat, C2′), 130.11 (CH, C5′), 138.05 (quat, C4′), 145.20 (quat, C1′), 160.08 (quat, C10) and 173.98 (quat, *C*OOMe).

##### 4.1.3.15 Ethyl 3-Ethyl-8-oxo-3-azabicyclo[3.2.1]octane-1-carboxylate (**27**)

To a solution of ethyl 2-oxocyclopentane-1-carboxylate (**25**, 253 mg, 1.64 mmol) and *N*,*N*-bis(ethoxymethyl)ethanamine (533 mg, 3.28 mmol) in acetonitrile (5 mL) was added trichloromethylsilane (642 mg, 3.28 mmol) at 0 °C followed by stirring for 20 h at 20 °C. The reaction was quenched with sat. aq NaHCO_3_ (pH = 8) and extracted with ethyl acetate (3 × 15 mL). The combined organic layers were washed with sat. aq brine (15 mL), dried (Na_2_SO_4_), and filtered, and then the solvents were removed by evaporation. The crude products were purified by chromatography over silica gel (petroleum ether/ethyl acetate = 7:1 v/v; the mobile phase was basified with concd aq 0.880 ammonia, 0.5% v/v of the prepared mobile phase; target compound TLC *R*_f_ = 0.25, stained with Dragendorff’s reagent and *p*-anisaldehyde solution) giving a pale yellow oil product (**27**, 183 mg, 50%). MS (*m*/*z*): [M + H]^+^ found 226.1448, C_12_H_20_NO_3_ requires 226.1438 and [M + Na]^+^ found 248.1265, C_12_H_19_NO_3_Na requires 248.1257. δ_H_ (500 MHz; CDCl_3_; calibrated with TMS): 1.09 (3H, t, *J* = 7.1 Hz, NCH_2_*CH*_*3*_), 1.28 (3H, t, *J* = 7.2 Hz, OCH_2_*CH*_*3*_), 1.95 (2H, m, 6-H_a′_ and 6-H_e′_), 2.25 (1H, ddd, *J* = 12.6, 10.1, 6.1 Hz, 7-H_a′_), 2.36 (1H, m, 5-H), 2.38 (1H, m, 7-H_e′_), 2.51 (1-H, m, 4-H_a_), 2.55 (2H, m, N*CH*_*2*_CH_3_), 2.70 (1H, d, *J* = 10.9 Hz, 2-H_a_), 3.00 (1H, ddd, *J* = 10.5, 4.0, 2.7 Hz, 4-H_e_), 3.15 (1H, dd, *J* = 11.0, 2.7 Hz, 2-H_e_) and 4.21 (2H, q, *J* = 7.2 Hz, O*CH*_*2*_CH_3_). δ_C_ (125 MHz; CDCl_3_; calibrated with TMS): 12.66 (CH_3_, NCH_2_*CH*_*3*_), 14.19 (CH_3_, OCH_2_*CH*_*3*_), 21.86 (CH_2_, C6), 27.61 (CH_2_, C7), 46.42 (CH, C5), 49.78 (CH_2_, N*CH*_*2*_CH_3_), 57.65 (quat, C1), 61.13 (CH_2_, C4), 61.17 (CH_2_, O*CH*_*2*_CH_3_), 62.24 (CH_2_, C2), 170.45 (quat, *C*OOEt) and 213.80 (quat, C8).

##### 4.1.3.16 Ethyl (*E*)-8-(2-(2,4-Dinitrophenyl)hydrazinylidene)-3-ethyl-3-azabicyclo[3.2.1]octane-1-carboxylate (**28**)

2,4-Dinitrophenylhydrazine (212 mg, 0.75 mmol) and concd aq H_2_SO_4_ solution (22 μL, 0.10 mmol) were added to a solution of ethyl 3-ethyl-8-oxo-3-azabicyclo[3.2.1]octane-1-carboxylate (**27**, 33 mg, 0.15 mmol) in MeOH (5 mL) at 0 °C, and the resulting solution was stirred and heated under reflux for 3 h under an atmosphere of anhydrous nitrogen. After the solution was cooled to 20 °C, about 2 mL of the solvent was removed by evaporation. The solution was basified with sat. aq NaHCO_3_ (pH = 8) and extracted with DCM (3 × 10 mL), and then the combined organic layers were washed with sat. aq brine (5 mL), dried (Na_2_SO_4_), and filtered, and then the solvents were removed by evaporation. The crude product was purified by chromatography over silica gel (petroleum ether/ethyl acetate = 5:1 v/v; the mobile phase was basified with concd aq 0.880 ammonia, 0.5% v/v of the prepared mobile phase; target compound TLC *R*_f_ = 0.35, stained with iodine vapor or ninhydrin solution) giving an orange solid product (22 mg, 37%). The product (**28**, 26 mg) was obtained after purification and the byproduct of 1-(2,4-dinitrophenyl)-2-(propan-2-ylidene)hydrazine was displayed. The ratio of ^1^H integrals between the target product and the byproduct was 5:1; thus, the yield is 37%. MS (*m*/*z*): [M + H]^+^ found 406.1755, C_18_H_24_N_5_O_6_ requires 406.1721. δ_H_ (500 MHz; CDCl_3_; calibrated with TMS): 1.10 (3H, t, *J* = 7.1 Hz, NCH_2_*CH*_*3*_), 1.36 (3H, t, *J* = 7.1 Hz, OCH_2_*CH*_*3*_), 1.94 (1H, tdd, *J* = 12.0, 6.4, 4.7 Hz, 6-H_a′_), 2.07 (1H, m, 6-H_e′_), 2.19 (1H, m, 7-H_a′_), 2.35 (1H, m, 4-H_a_), 2.37 (1H, m, 7-H_e′_), 2.55 (2H, q, *J* = 7.1 Hz, N*CH*_*2*_CH_3_), 2.64 (1H, d, *J* = 10.7 Hz, 2-H_a_), 2.99 (1H, ddd, *J* = 10.3, 4.1, 1.6 Hz, 4-H_e_), 3.18 (1H, m, 2-H_e_), 3.19 (1H, m, 5-H), 4.31 (2H, qd, *J* = 7.1, 4.0 Hz, O*CH*_*2*_CH_3_), 7.84 (1H, d, *J* = 9.5 Hz, 6′-H), 8.29 (1H, dd, *J* = 9.5, 2.6 Hz, 5′-H), 9.11 (1H, d, *J* = 2.6 Hz, 3′-H) and 11.02 (1H, s, NH). δ_C_ (125 MHz; CDCl_3_; calibrated with TMS): 12.44 (CH_3_, NCH_2_*CH*_*3*_), 14.42 (CH_3_, OCH_2_*CH*_*3*_), 25.40 (CH_2_, C6), 29.81 (CH_2_, C7), 36.62 (CH, C5), 50.18 (CH_2_, N*CH*_*2*_CH_3_), 54.58 (quat, C1), 58.70 (CH_2_, C4), 61.14 (CH_2_, O*CH*_*2*_CH_3_), 62.64 (CH_2_, C2), 116.20 (CH, C6′), 123.56 (CH, C3′), 129.07 (quat, C2′), 130.01 (CH, C5′), 137.86 (quat, C4′), 145.38 (quat, C1′), 168.39 (quat, C8) and 171.05 (quat, *C*OOEt).

##### 4.1.3.17 Ethyl 1-[(Ethylamino)methyl]-2-oxocyclohexane-1-carboxylate (**29**)

A solution of ethyl 2-oxocyclohexane-1-carboxylate (**6**, 532 mg, 3.13 mmol), ethylamine aq solution (0.24 mL, 2.82 mmol), and paraformaldehyde (85 mg, 2.82 mmol) in EtOH (10 mL) was stirred and heated for 3 h at 40 °C under an atmosphere of anhydrous nitrogen. TLC monitoring showed the reaction was complete (DCM/MeOH = 40:1 v/v, target compound *R*_f_ = 0.2, stained with *p*-anisaldehyde solution and iodine vapor). After the solvent was removed by evaporation, the crude product was purified by chromatography over silica gel (DCM/MeOH = 40:1 v/v; the mobile phase was basified with concd aq 0.880 ammonia, 0.5% v/v of the prepared mobile phase) producing the pale yellow oil product of the mono-Mannich reaction (**29**, 37 mg, 5%). MS (*m*/*z*): [M + H]^+^ found 228.1602, C_12_H_22_NO_3_ requires 228.1594. δ_H_ (500 MHz; CDCl_3_; calibrated with TMS): 1.04 (3H, t, *J* = 7.1 Hz, NCH_2_*CH*_*3*_), 1.27 (3H, t, *J* = 6.8 Hz, OCH_2_*CH*_*3*_), 1.68 (3H, m, 4-H_A_, 5-H_A_ and 6-H_A_), 1.77 (1H, m, 5-H_B_), 2.00 (1H, m, 4-H_B_), 2.42 (1H, m, 6-H_B_), 2.43 (1H, m, 3-H_A_), 2.58 (1H, m, 3-H_B_), 2.60 (2H, m, N*CH*_*2*_CH_3_), 2.73 (1H, d, *J* = 11.7 Hz, 1′-H_A_), 2.92 (1H, d, *J* = 11.7 Hz, 1′-H_B_), 4.23 (2H, m, O*CH*_*2*_CH_3_). δ_C_ (125 MHz; CDCl_3_; calibrated with TMS): 14.15 (CH_3_, OCH_2_*CH*_*3*_), 15.15 (CH_3_, NCH_2_*CH*_*3*_), 22.38 (CH_2_, C5), 27.26 (CH_2_, C4), 34.77 (CH_2_, C6), 41.12 (CH_2_, C3), 44.60 (CH_2_, N*CH*_*2*_CH_3_), 53.76 (CH_2_, C1′), 61.23 (CH_2_, O*CH*_*2*_CH_3_), 62.19 (quat, C1), 172.06 (quat, *C*OOEt) and 209.24 (quat, C2).

##### 4.1.3.18 3-Ethyl-1-(hydroxymethyl)-3-azabicyclo[3.3.1]nonan-9-ol (**30**)

Lithium aluminum hydride (410 mg, 10.75 mmol, 1.6 equiv) was added to a solution of the ethyl 3-ethyl-9-oxo-3-azabicyclo[3.3.1]nonane-1-carboxylate (**8**, 513 mg, 2.15 mmol) in anhydrous THF (50 mL) at 0 °C; then the mixture was warmed to 20 °C and stirred under an atmosphere of anhydrous nitrogen for 4 h. The reaction mixture was diluted with anhydrous THF (30 mL) and then quenched by the dropwise addition of water (20 mL) cooled in an ice-bath at 0 °C. The organic solvent (40 mL) was removed by evaporation and the remaining aq solution was extracted with DCM (3 × 30 mL). The combined organic layers were washed with sat. aq brine (50 mL), dried (Na_2_SO_4_), filtered, and then the solvents were removed by evaporation. The crude product was purified by chromatography over silica gel (DCM/MeOH = 11:1 v/v; the mobile phase was basified with concd aq 0.880 ammonia, 0.5% v/v of the prepared mobile phase; target compound TLC *R*_f_ = 0.25, stained with iodine vapor) to give the title compound as colorless oil (**30**, 78 mg, 18%). [M + Na]^+^ found 222.1450, C_11_H_21_NO_2_Na requires 222.1470. δ_H_ (500 MHz; CDCl_3_; calibrated with CDCl_3_): 1.04 (3H, t, *J* = 7.2 Hz, NCH_2_*CH*_*3*_), 1.28 (1H, dd, *J* = 13.4, 6.2 Hz, 8-H_e_), 1.48 (1H, m, 7-H_e_), 1.52 (1H, m, 6-H_e_), 1.83 (1H, br m, 2-H_a_), 1.86 (1H, br m, 5-H), 1.91 (1H, m, 6-H_a_), 2.01 (1H, dtd, *J* = 13.4, 6.2, 2.0 Hz, 8-H_a_), 2.19 (1H, br m, 4-H_a_), 2.25 (2H, br m, N*CH*_*2*_CH_3_), 2.59 (1H, m, 7-H_a_), 2.64 (1H, d, *J* = 11.1 Hz, 2-H_e_), 2.98 (1H, d, *J* = 10.8 Hz, 4-H_e_), 3.37 (1H, d, *J* = 10.8 Hz, 1′-H_A_), 3.43 (1H, d, *J* = 10.8 Hz, 1′-H_B_) and 3.72 (1H, br d, *J* = 2.0 Hz, 9-H). δ_C_ [125 MHz; CDCl_3_; calibrated with CDCl_3_ (77.16 ppm)]: 12.83 (CH_3_, NCH_2_*CH*_*3*_), 20.59 (CH_2_, C7), 24.02 (CH_2_, C6), 26.59 (CH_2_, C8), 26.27 (CH, C5), 28.29 (quat, C1), 52.56 (CH_2_, N*CH*_*2*_CH_3_), 58.43 (CH_2_, C4), 60.52 (CH_2_, C2), 71.30 (CH_2_, C1′), and 75.52 (CH, C9). δ_H_ (500 MHz; CD_3_OD; calibrated with TMS): 1.05 (3H, t, *J* = 7.1 Hz, NCH_2_*CH*_*3*_), 1.35 (1H, dd, *J* = 13.5, 6.5 Hz, 8-H_e_), 1.42 (1H, m, 7-H_e_), 1.48 (1H, dd, *J* = 13.5, 6.5 Hz, 6-H_e_), 1.75 (1H, tdd, *J* = 13.5, 6.5, 2.4 Hz, 8-H_a_), 1.81 (1H, br m, 5-H), 1.96 (1H, m, 2-H_a_), 1.99 (1H, br m, 6-H_a_), 2.19 (1H, m, 4-H_a_), 2.25 (2H, br m, N*CH*_*2*_CH_3_), 2.58 (1H, br m, 7-H_a_), 2.83 (1H, d, *J* = 9.5 Hz, 2-H_e_), 3.00 (1H, d, *J* = 8.5 Hz, 4-H_e_), 3.28 (1H, d, *J* = 11.0 Hz, 1′-H_A_), 3.34 (1H, d, *J* = 11.0 Hz, 1′-H_B_) and 3.56 (1H, br d, *J* = 3.6 Hz, 9-H). δ_C_ (125 MHz; CD_3_OD; calibrated with TMS): 13.13 (CH_3_, NCH_2_*CH*_*3*_), 21.77 (CH_2_, C7), 25.35 (CH_2_, C6), 28.03 (CH_2_, C8), 37.69 (CH, C5), 39.85 (quat, C1), 53.76 (CH_2_, N*CH*_*2*_CH_3_), 59.97 (CH_2_, C4), 62.02 (CH_2_, C2), 69.91 (CH_2_, C1′) and 73.98 (CH, C9). δ_H_ [500 MHz; *d*_6_-DMSO; calibrated with residual DMSO (2.50 ppm)]: 0.97 (3H, br t, *J* = 7.2 Hz, NCH_2_*CH*_*3*_), 1.23 (1H, dd, *J* = 13.0, 6.5 Hz, 8-H_e_), 1.29 (1H, m, 7-H_e_), 1.35 (1H, br dd, *J* = 12.6, 4.9 Hz, 6-H_e_), 1.58 (1H, tdd, *J* = 13.0, 6.5, 2.2 Hz, 8-H_a_), 1.69 (1H, br m, 5-H), 1.85 (1H, br m, 2-H_a_), 1.89 (1H, m, 6-H_a_), 2.04 (1H, br d, *J* = 10.0 Hz, 4-H_a_), 2.14 (2H, br s, N*CH*_*2*_CH_3_), 2.47 (1H, br m, 7-H_a_), 2.73 (1H, d, *J* = 10.1 Hz, 2-H_e_), 2.88 (1H, d, *J* = 10.0 Hz, 4-H_e_), 3.08 (1H, dd, *J* = 10.6, 5.5 Hz, 1′-H_A_), 3.17 (1H, dd, *J* = 10.6, 5.5 Hz, 1′-H_B_), 3.37 [1H, m (overlapped with HDO peak), 9-H], 4.29 (1H, br s, CH_2_O*H*) and 4.43 (1H, br s, 9-OH). δ_C_ [125 MHz; *d*_6_-DMSO; calibrated with *d*_6_-DMSO (39.52 ppm)]: 12.28 (CH_3_, NCH_2_*CH*_*3*_), 20.65 (CH_2_, C7), 24.32 (CH_2_, C6), 27.01 (CH_2_, C8), 35.91 (CH, C5), 38.50 (quat, C1), 52.16 (CH_2_, N*CH*_*2*_CH_3_), 58.62 (CH_2_, C4), 60.83 (CH_2_, C2), 67.64 (CH_2_, C1′) and 70.92 (CH, C9). δ_H_ [500 MHz; D_2_O/*d*_6_-DMSO (2 drops); calibrated with residual DMSO (2.71 ppm)]: 1.12 (3H, t, *J* = 7.2 Hz, NCH_2_*CH*_*3*_), 1.45 (1H, dd, *J* = 13.0, 7.0 Hz, 8-H_e_), 1.59 (1H, m, 7-H_e_), 1.60 (1H, m, 6-H_e_), 1.62 (1H, tdd, *J* = 13.0, 7.0, 2.5 Hz, 8-H_a_), 1.91 (1H, tt, *J* = 13.5, 7.0 Hz, 6-H_a_), 2.02 (1H, m, 5-H), 2.04 (1H, m, 7-H_a_), 2.25 (1H, br d, *J* = 12.0 Hz, 2-H_a_), 2.46 (3H, m, 4-H_a_ and N*CH*_*2*_CH_3_), 3.02 (1H, d, *J* = 12.0 Hz, 2-H_e_), 3.17(1H, d, *J* = 12.0 Hz, 4-H_e_), 3.32 (1H, d, *J* = 11.3 Hz, 1′-H_A_), 3.46 (1H, d, *J* = 11.3 Hz, 1′-H_B_) and 3.75 (1H, d, *J* = 2.5 Hz, 9-H). δ_C_ [125 MHz; D_2_O/*d*_6_-DMSO (2 drops); calibrated with *d*_6_-DMSO (39.52 ppm)]: 12.46 (CH_3_, NCH_2_*CH*_*3*_), 20.49 (CH_2_, C7), 24.14 (CH_2_, C6), 27.19 (CH_2_, C8), 36.33 (CH, C5), 39.86 (quat, C1), 55.47 (CH_2_, N*CH*_*2*_CH_3_), 59.65 (CH_2_, C4), 61.46 (CH_2_, C2), 68.81 (CH_2_, C1′) and 72.29 (CH, C9).

##### 4.1.3.19 1-(Ethoxycarbonyl)-3-ethyl-9-oxo-3-azabicyclo[3.3.1]nonan-3-ium Acetate Salt (**31**)

Ethyl 3-ethyl-9-oxo-3-azabicyclo[3.3.1]nonane-1-carboxylate (**8**, 20 mg) was dissolved in *d*_4_-acetic acid (0.8 mL) and the title ketone (**31**) was produced. δ_H_ [500 MHz; *d*_4_-acetic acid; calibrated with residual acetic acid (2.05 ppm)]: 1.29 (3H, t, *J* = 7.2 Hz, OCH_2_*CH*_*3*_), 1.38 (3H, t, *J* = 7.2 Hz, NCH_2_*CH*_*3*_), 1.72 (1H, m, 7-H_e_), 2.12 (1H, tdd, *J* = 14.3, 5.8, 5.2 Hz, 6-H_a_), 2.35 (1H, m, 6-H_e_), 2.41 (1H, m, 8-H_e_), 2.42 (1H, m, 7-H_a_), 2.58 (1H, td, *J* = 14.3, 5.8 Hz, 8-H_a_), 2.83 (1H, tt, *J* = 5.2, 2.4 Hz, 5-H), 3.35 (2H, qd, *J* = 7.2, 2.4 Hz, N*CH*_*2*_CH_3_), 3.66 (1H, dd, *J* = 13.2, 5.2 Hz, 4-H_a_), 4.01 (1H, d, *J* = 13.4 Hz, 2-H_a_), 4.12 (1H, m, 4-H_e_), 4.15 (1H, m, 2-H_e_) and 4.26 (2H, q, *J* = 7.2 Hz, O*CH*_*2*_CH_3_). δ_C_ [125 MHz; *d*_4_-acetic acid; calibrated with *d*_4_-acetic acid (20.00 ppm)]: 9.98 (CH_3_, NCH_2_*CH*_*3*_), 14.30 (CH_3_, OCH_2_*CH*_*3*_), 18.45 (CH_2_, C7), 33.93 (CH_2_, C6), 36.80 (CH_2_, C8), 45.11 (CH, C5), 55.30 (CH_2_, N*CH*_*2*_CH_3_), 57.03 (CH_2_, C4), 58.06 (quat, C1), 58.20 (CH_2_, C2), 63.24 (CH_2_, O*CH*_*2*_CH_3_), 170.15 (quat, *C*OOEt) and 208.66 (quat, C9).

##### 4.1.3.20 1-(Ethoxycarbonyl)-3-ethyl-9-oxo-3-azabicyclo[3.3.1]nonan-3-ium Chloride (**32**)

A solution of ethyl 3-ethyl-9-oxo-3-azabicyclo[3.3.1]nonane-1-carboxylate (**8**, 225 mg) in MeOH (2 mL) was titrated with concd aq HCl solution (37%, w/w) to pH = 2, then all the solvents and the excess of HCl were removed by evaporation to give the ketone as its HCl salt (**32**). δ_H_ [500 MHz; *d*_4_-acetic acid; calibrated with residual acetic acid (2.05 ppm)]: 1.30 (3H, t, *J* = 7.2 Hz, OCH_2_*CH*_*3*_), 1.47 (3H, t, *J* = 7.1 Hz, NCH_2_*CH*_*3*_), 1.74 (1H, m, 7-H_e_), 2.15 (1H, m, 6-H_a_), 2.48 (1H, br d, *J* = 13.3 Hz, 6-H_e_), 2.55 (1H, m, 8-H_e_), 2.60 (1H, m, 7-H_a_), 2.61 (1H, m, 8-H_a_), 2.82 (1H, m, 5-H), 3.44 (2H, q, *J* = 7.1 Hz, N*CH*_*2*_CH_3_), 3.64 (1H, br d, *J* = 9.7 Hz, 4-H_a_), 4.01 (1H, br d, *J* = 13.5 Hz, 2-H_a_), 4.16 (1H, m, 2-H_e_), 4.19 (1H, m, 4-H_e_) and 4.27 (2H, q, *J* = 7.2 Hz, O*CH*_*2*_CH_3_). δ_C_ [125 MHz; *d*_4_-acetic acid; calibrated with *d*_4_-acetic acid (20.00 ppm)]: 10.12 (CH_3_, NCH_2_*CH*_*3*_), 14.31 (CH_3_, OCH_2_*CH*_*3*_), 19.35 (CH_2_, C7), 33.52 (CH_2_, C6), 36.46 (CH_2_, C8), 45.28 (CH, C5), 55.62 (CH_2_, N*CH*_*2*_CH_3_), 57.47 (CH_2_, C4), 57.98 (quat, C1), 58.55 (CH_2_, C2), 63.34 (CH_2_, O*CH*_*2*_CH_3_), 169.91 (quat, *C*OOEt) and 208.26 (quat, C9).

##### 4.1.3.21 1-(Ethoxycarbonyl)-3-ethyl-9,9-dihydroxy-3-azabicyclo-[3.3.1]nonan-3-ium Chloride (**33**)

The ketone (**32**) HCl salt was dissolved in the indicated solvents and the title ketal (**33**, hydrate) salt was generated. δ_H_ (500 MHz; D_2_O; calibrated with TMSP): 1.29 (3H, t, *J* = 7.2 Hz, OCH_2_*CH*_*3*_), 1.37 (3H, t, *J* = 7.2 Hz, NCH_2_*CH*_*3*_), 1.72 (1H, qt, *J* = 14.1, 7.0 Hz, 7-H_A_), 1.83 (1H, m, 7-H_B_), 1.85 (1H, m, 6-H_e_), 2.00 (1H, dd, *J* = 14.1, 7.0 Hz, 8-H_e_), 2.15 (1H, ddddd, *J* = 14.1, 13.1, 7.0, 4.2, 1.8 Hz, 6-H_a_), 2.23 (1H, tt, *J* = 4.2, 2.2 Hz, 5-H), 2.39 (1H, tdd, *J* = 14.1, 7.0, 2.0 Hz, 8-H_a_), 3.26 (2H, q, *J* = 7.2 Hz, N*CH*_*2*_CH_3_), 3.50 (1H, br ddd, *J* = 13.2, 4.2, 1.8 Hz, 4-H_a_), 3.64 (2H, m, 4-H_e_ and 2-H_a_), 3.82 (1H, d, *J* = 13.4 Hz, 2-H_e_) and 4.26 (2H, qd, *J* = 7.2, 1.0 Hz, O*CH*_*2*_CH_3_). δ_C_ (125 MHz; D_2_O; calibrated with TMSP): 11.90 (CH_3_, NCH_2_*CH*_*3*_), 16.05 (CH_3_, OCH_2_*CH*_*3*_), 20.73 (CH_2_, C7), 27.97 (CH_2_, C6), 32.66 (CH_2_, C8), 41.39 (CH, C5), 51.96 (quat, C1), 57.47 (CH_2_, C4), 57.93 (CH_2_, C2), 58.73 (CH_2_, N*CH*_*2*_CH_3_), 66.03 (CH_2_, O*CH*_*2*_CH_3_), 95.55 (quat, C9) and 176.04 (quat, *C*OOEt). δ_H_ (500 MHz; CD_3_OD; calibrated with TMS): 1.30 (3H, t, *J* = 7.2 Hz, OCH_2_*CH*_*3*_), 1.10 (3H, t, *J* = 7.2 Hz, NCH_2_*CH*_*3*_), 1.78 (1H, m, 7-H_A_), 1.88 (1H, m, 6-H_e_), 1.89 (1H, m, 7-H_B_), 1.94 (1H, m, 8-H_e_), 2.20 (1H, m, 6-H_a_), 2.40 (1H, m, 8-H_a_), 2.45 (1H, m, 5-H), 3.23 (1H, m, 4-H_a_), 3.24 (2H, m, N*CH*_*2*_CH_3_), 3.55 (1H, m, 4-H_e_), 3.60 (1H, m, 2-H_a_), 3.75 (1H, m, 2-H_e_) and 4.24 (2H, qd, *J* = 7.2, 1.0 Hz, O*CH*_*2*_CH_3_). δ_C_ [125 MHz; CD_3_OD; calibrated with CD_3_OD (49.50 ppm)]: 10.53 (CH_3_, NCH_2_*CH*_*3*_), 14.86 (CH_3_, OCH_2_*CH*_*3*_), 19.95 (CH_2_, C7), 26.92 (CH_2_, C6), 32.93 (CH_2_, C8), 34.69 (CH, C5), 50.69 (quat, C1), 55.85 (CH_2_, C4), 56.50 (CH_2_, C2), 57.73 (CH_2_, N*CH*_*2*_CH_3_), 63.78 (CH_2_, O*CH*_*2*_CH_3_), 96.99 (quat, C9) and 174.77 (quat, *C*OOEt). δ_H_ [500 MHz; *d*_6_-DMSO/D_2_O (2 drops); calibrated with residual DMSO (2.50 ppm)]: 1.20 (3H, m, OCH_2_*CH*_*3*_), 1.24 (3H, m, NCH_2_*CH*_*3*_), 1.55 (1H, br m, 7-H_A_), 1.68 (1H, br m, 6-H_e_), 1.78 (1H, br dd, *J* = 13.7, 5.2 Hz, 8-H_e_), 1.90 (1H, m, 7-H_B_), 2.21 (1H, m, 6-H_a_), 2.03 (1H, br m, 5-H), 2.25 (1H, m, 8-H_a_), 3.11 (2H, m, N*CH*_*2*_CH_3_), 3.25 (1H, br d, *J* = 12.0 Hz, 4-H_a_), 3.45 (1H, m, 2-H_a_), 3.47 (1H, m, 4-H_e_), 3.55 (1H, br d, *J* = 13.2 Hz, 2-H_e_) and 4.14 (2H, m, O*CH*_*2*_CH_3_). δ_C_ [125 MHz; *d*_6_-DMSO/D_2_O (2 drops); calibrated with *d*_6_-DMSO (39.52 ppm)]: 9.72 (CH_3_, NCH_2_*CH*_*3*_), 14.25 (CH_3_, OCH_2_*CH*_*3*_), 17.87 (CH_2_, C7), 25.41 (CH_2_, C6), 29.88 (CH_2_, C8), 38.65 (CH, C5), 49.19 (quat, C1), 54.10 (CH_2_, C4), 55.02 (CH_2_, C2), 55.62 (CH_2_, N*CH*_*2*_CH_3_), 61.75 (CH_2_, O*CH*_*2*_CH_3_), 91.92 (quat, C9) and 172.29 (quat, *C*OOEt).

##### 4.1.3.22 1-(Ethoxycarbonyl)-3-ethyl-3-methyl-9-oxo-3-azabicyclo-[3.3.1]nonan-3-ium Iodide (**34**)

A solution of ethyl 3-ethyl-9-oxo-3-azabicyclo[3.3.1]nonane-1-carboxylate (**8**, 101 mg, 0.42 mmol) and methyl iodide (0.13 mL, 2.09 mmol, 5 equiv) in THF (3 mL) was heated under reflux under an atmosphere of anhydrous nitrogen for 24 h. After the solvent and the excess of methyl iodide were removed by evaporation, and the residue was washed with Et_2_O (5 × 2 mL) and EtOAc (5 × 2 mL). The crude product was purified by chromatography over silica gel (DCM/MeOH 10:1 v/v; the mobile phase was basified with concd aq 0.880 ammonia, 0.5% v/v of the prepared mobile phase; target compound TLC *R*_f_ = 0.2, stained with Dragendorff’s reagent) giving the title compound (**34**, 23 mg, 15%). [M]^+^ found 254.1733, C_14_H_24_NO_3_^+^ requires 254.1751 (254.17562 – 0.00055 ≈ 254.1751 Da, difference = 7 ppm). δ_H_ (500 MHz; CDCl_3_; calibrated with TMS): 1.35 (3H, t, *J* = 7.2 Hz, OCH_2_*CH*_*3*_), 1.54 (3H, t, *J* = 7.2 Hz, NCH_2_*CH*_*3*_), 1.87 (1H, dt, *J* = 16.1, 5.0 Hz, 7-H_e_), 2.07 (1H, tt, *J* = 13.8, 5.0 Hz, 6-H_a_), 2.35 (1H, m, 6-H_e_), 2.38 (1H, m, 8-H_e_), 2.47 (1H, td, *J* = 13.8, 5.0 Hz, 8-H_a_), 2.84 (1H, dtt, *J* = 16.1, 13.8, 5.0 Hz, 7-H_a_), 3.15 (4H, m, 5-H and N*CH*_*3*_), 4.24 (1H, d, *J* = 13.8 Hz, 2-H_e_), 4.31 (2H, m, O*CH*_*2*_CH_3_), 4.35 (2H, m, N*CH*_*2*_CH_3_), 4.43 (2H, d, *J* = 7.1 Hz, 4-H) and 4.56 (1H, d, *J* = 13.8 Hz, 2-H_a_). δ_C_ (125 MHz; CDCl_3_; calibrated with TMS): 8.96 (CH_3_, NCH_2_*CH*_*3*_), 14.12 (CH_3_, OCH_2_*CH*_*3*_), 16.68 (CH_2_, C7), 36.08 (CH_2_, C6), 38.50 (CH_2_, C8), 42.21 (CH, C5), 47.90 (CH_3_, N*CH*_*3*_), 57.19 (quat, C1), 63.22 (CH_2_, O*CH*_*2*_CH_3_), 63.98 (CH_2_, C4), 65.33 (CH_2_ × 2, C2 and N*CH*_*2*_CH_3_) and 169.55 (quat, *C*OOEt) and 207.19 (quat, C9).

##### 4.1.3.23 ((1*R*,5*S*,9*s*))-9-Hydroxy-3-oxabicyclo[3.3.1]nonane-1,5-diyl Dimethanol (**36**)^28^

Tetramethylolcyclohexanol (**35**, 1.0 g) was heated to melting at 160 °C, and then the anhydrous gaseous HCl (concd aq HCl in a dropping funnel was added dropwise into a three-necked round-bottomed flask with concd aq H_2_SO_4_, and the generated gaseous HCl was piped into a Drechsel gas washing bottle containing concd aq H_2_SO_4_, forming the anhydrous gaseous HCl) was slowly pumped into the round-bottomed flask with the melted reactant for 10 min. Then, the reaction was heated at 160 °C for 15 min to remove residual HCl. After cooling to 20 °C, water (10 mL) was added to the crude products. Aqueous solution was extracted with chloroform (3 × 10 mL), then extracted with ethyl acetate (5 × 15 mL), and the combined organic extracts were concentrated by evaporation forming a white solid ether (**36**, 142 mg, 16%). MS (*m*/*z*): [M + H]^+^ found 203.1281, C_10_H_19_O_4_ requires 203.1283 and [M + Na]^+^ found 225.1104, C_10_H_18_O_4_Na requires 225.1103. δ_H_ (500 MHz; CD_3_OD; calibrated with TMS): 1.44 (2H, tdd, *J* = 13.5, 6.5, 2.4 Hz, 6-H_a_ and 8-H_a_), 1.51 (1H, dt, *J* = 13.5, 6.5 Hz, 7-H_e_), 1.78 (2H, dd, *J* = 13.5, 6.5 Hz, 6-H_e_ and 8-H_e_), 2.32 (1H, qt, *J* = 13.5, 6.5 Hz, 7-H_a_), 3.33 (2H, d, *J* = 11.1 Hz, O-1_A_ and O-1_A_^′^), 3.37 (2H, d, *J* = 11.1 Hz, O-1_B_ and O-1_B_^′^), 3.52 (1H, s, 9-H), 3.53 (2H, d, *J* = 11.4 Hz, 2-H_e_ and 4-H_e_) and 3.84 (2H, d, *J* = 11.4, 2.4 Hz, 2-H_a_ and 4-H_a_). δ_C_ (125 MHz; CD_3_OD; calibrated with TMS): 21.66 (CH_2_, C7), 34.20 (CH_2_ × 2, C6 and C8), 40.71 (quat × 2, C1 and C5), 67.84 (CH_2_ × 2, O-1 and O-1′), 70.07 (CH_2_ × 2, C2 and C4) and 74.39 (CH, C9). δ_H_ [500 MHz; *d*_6_-DMSO; calibrated with residual DMSO (2.50 ppm)]: 1.35 (2H, m, 6-H_a_ and 8-H_a_), 1.38 (1H, m, 7-H_e_), 1.70 (2H, dd, *J* = 13.0, 5.7 Hz, 6-H_e_ and 8-H_e_), 2.18 (1H, qt, *J* = 13.0, 5.7 Hz, 7-H_a_), 3.15 (2H × 2, m, O-1 and O-1′), 3.29 (1H, d, *J* = 4.2 Hz, 9-H), 3.38 (2H, m, 2-H_e_ and 4-H_e_) and 3.59 (2H, dd, *J* = 11.1, 2.1 Hz, 2-H_a_ and 4-H_a_). δ_C_ [125 MHz; *d*_6_-DMSO; calibrated with *d*_6_-DMSO (39.52 ppm)]: 20.42 (CH_2_, C7), 32.91 (CH_2_ × 2, C6 and C8), 43.44 (quat × 2, C1 and C5), 65.48 (CH_2_ × 2, O-1 and O-1′), 68.76 (CH_2_ × 2, C2 and C4) and 71.15 (CH, C9). δ_H_ [500 MHz; *d*_6_-acetone; calibrated with residual acetone (2.05 ppm)]: 1.37 (2H, tdd, *J* = 13.5, 5.8, 2.5 Hz, 6-H_a_ and 8-H_a_), 1.43 (1H, dt, *J* = 13.5, 5.8 Hz, 7-H_e_), 1.66 (2H, dd, *J* = 13.5, 5.8, 2.5 Hz, 6-H_e_ and 8-H_e_), 2.35 (1H, qt, *J* = 13.5, 5.8 Hz, 7-H_a_), 3.36 (2H, d, *J* = 10.9 Hz, O-1_A_ and O-1_A_^′^), 3.39 (2H, d, *J* = 10.9 Hz, O-1_B_ and O-1_B_^′^), 3.45 (2H, d, *J* = 11.2 Hz, 2-H_e_ and 4-H_e_), 3.64 (1H, br s, 9-H) and 3.90 (2H, dd, *J* = 11.2, 2.5 Hz, 2-H_a_ and 4-H_a_). δ_C_ [125 MHz; *d*_6_-acetone; calibrated with *d*_6_-acetone (29.84 ppm)]: 21.38 (CH_2_, C7), 33.82 (CH_2_ × 2, C6 and C8), 39.94 (quat × 2, C1 and C5), 68.27 (CH_2_ × 2, O-1 and O-1′), 69.32 (CH_2_ × 2, C2 and C4) and 75.83 (CH, C9). δ_H_ (500 MHz; D_2_O; calibrated with TMSP): 1.52 (2H, m, 6-H_a_ and 8-H_a_), 1.57 (1H, m, 7-H_e_), 1.82 (2H, dd, *J* = 12.8, 5.8 Hz, 6-H_e_ and 8-H_e_), 2.16 (1H, tq, *J* = 12.8, 5.8 Hz, 7-H_a_), 3.37 (2H, d, *J* = 11.5 Hz, O-1_A_ and O-1_A_^′^), 3.42 (2H, d, *J* = 11.5 Hz, O-1_B_ and O-1_B_^′^), 3.58 (1H, s, 9-H), 3.63 (2H, d, *J* = 11.2 Hz, 2-H_e_ and 4-H_e_) and 3.73 (2H, br d, *J* = 11.2 Hz, 2-H_a_ and 4-H_a_). δ_C_ (125 MHz; D_2_O; calibrated with TMSP): 22.69 (CH_2_, C7), 35.21 (CH_2_ × 2, C6 and C8), 42.03 (quat × 2, C1 and C5), 68.34 (CH_2_ × 2, O-1 and O-1′), 71.13 (CH_2_ × 2, C2 and C4) and 74.32 (CH, C9).

#### 4.1.4 Recrystallization

The conditions of recrystallization obtaining single crystals for the X-ray diffraction studies are shown in Table 2. Generally, the selected hot solvent(s) were added dropwise to a glass vial (5–10 mL) containing the indicated sample (5–25 mg) heated on a water bath (40–60 °C) with slow shaking until the sample was fully dissolved, and then the glass vial was covered with foil, allowed to cool to 20 °C, and then partially evaporated at 20 °C for the required period. After the single crystals were formed (in solution), intensity data for compounds (**18**), (**19**), (**20**), (**24**), (**32**), and (**36**) were collected on a Rigaku Supernova Dual, EosS2 system using monochromated Cu Kα radiation (λ = 1.54184 Å) at 150 ± 2 K. The details of recrystallization and SXRD data are reported in Tables S1–S7.

**Table 2:** Conditions of Recrystallization

compound	solvent(s)	time
**18**	MeOH	3 days
**19**	MeOH/Et_2_O	2 days
**20**	MeOH/Et_2_O	4 days
**24**	MeOH/Et_2_O	1 week
**32**	EtOAc/CHCl_3_/MeOH	3 weeks
**36**	EtOAc	14 h

## Supporting Information Available

Key 2D NMR correlations (COSY, HMBC, and NOESY), ESI-MS spectra, and fully assigned 1D/2D NMR spectra (PDF)
Crystallographic data from the structural analysis with CCDC no. 1975722 (**18)** (CIF)
Crystallographic data from the structural analysis with CCDC no. 1975720 (**19**) (CIF)
Crystallographic data from the structural analysis with CCDC no. 1975723 (**20**) (CIF)
Crystallographic data from the structural analysis with CCDC no. 1975725 (**24**) (CIF)
Crystallographic data from the structural analysis with CCDC no. 1975721 (**32**) (CIF)
Crystallographic data from the structural analysis with CCDC no. 1975724 (**36**) (CIF)

## Supplementary Material

ao1c01093_si_001.pdf

ao1c01093_si_002.cif

ao1c01093_si_003.cif

ao1c01093_si_004.cif

ao1c01093_si_005.cif

ao1c01093_si_006.cif

ao1c01093_si_007.cif
