# Supplementary material for: The 1H NMR Spectroscopic Effect of Steric Compression Is Found in [3.3.1]Oxa- and Azabicycles and Their Analogues
Source: ACS Omega. 2021 May 7;6(19):12769–86. doi: 10.1021/acsomega.1c01093 (PMC8154237; doi:10.1021/acsomega.1c01093)
Supplement: Supplementary file 1 — ao1c01093_si_001.pdf [file ao1c01093_si_001.pdf]

The ^{1}H NMR Spectroscopic Effect of Steric Compression Is Found in [3.3.1]Oxa- and Azabicycles and Their Analogues

Ziyu Zeng,[a] Gabriele Kociok-Köhn,[b] Timothy J. Woodman,[a,b] Michael G. Rowan,[a] and Ian S. Blagbrough*,[a]

[a]Department of Pharmacy and Pharmacology and [b]Material and Chemical Characterisation Facility, University of Bath, Bath BA2 7AY, U.K.

Supporting Information

CONTENTS

1. Key 2D NMR correlations 2

2. Variable temperature (VT) ^{1}H NMR experiments 9

3. Recrystallization and data of single-crystal X-ray determinations 10

4. Key ^{1}H NMR data related to steric compression 15

5. NMR spectra and MS data of synthetic compounds 17

References 135

1. Key 2D NMR correlations

Tautomers of ethyl 2-oxocyclohexane-1-carboxylate (**6**, keto) and ethyl 2-hydroxycyclohex-1-ene-1-carboxylate (**7**, enol)

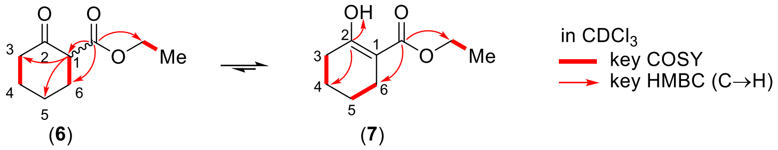

Ethyl 3-ethyl-9-oxo-3-azabicyclo[3.3.1]nonane-1-carboxylate (**8**)

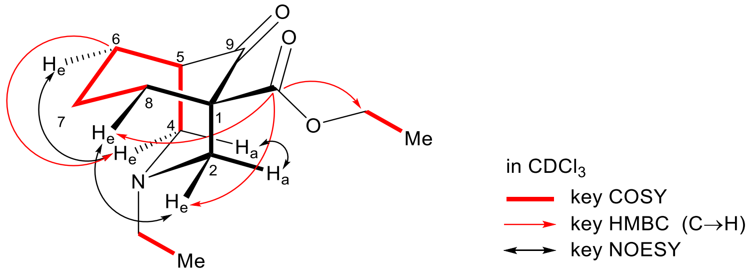

Ethyl 3-ethyl-7,7-dimethyl-9-oxo-3-azabicyclo[3.3.1]nonane-1-carboxylate (**10**)

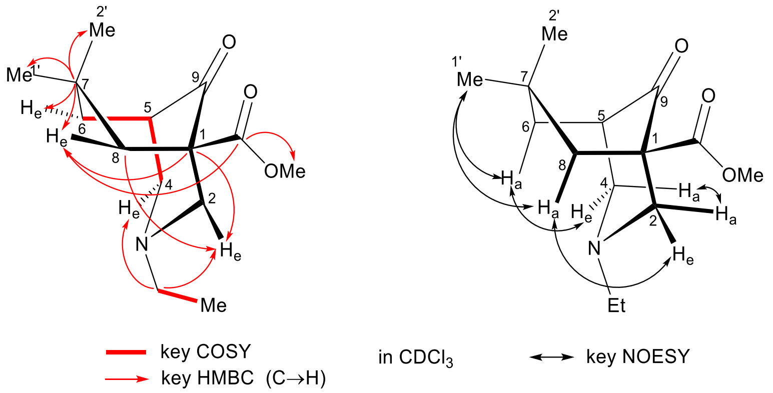

Methyl 3-ethyl-7-isopropyl-9-oxo-3-azabicyclo[3.3.1]nonane-1-carboxylate (**12**)

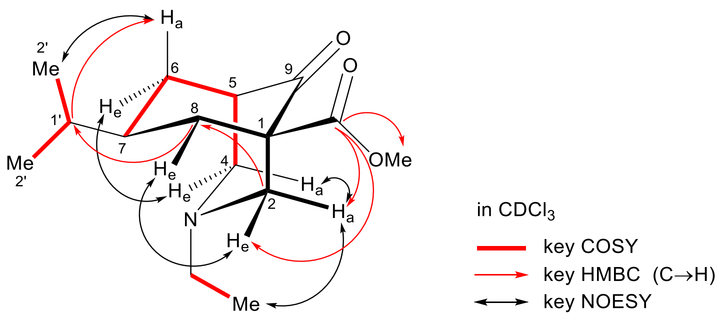

Ethyl 3-ethyl-7-methyl-9-oxo-3-azabicyclo[3.3.1]nonane-1-carboxylate (**14**)

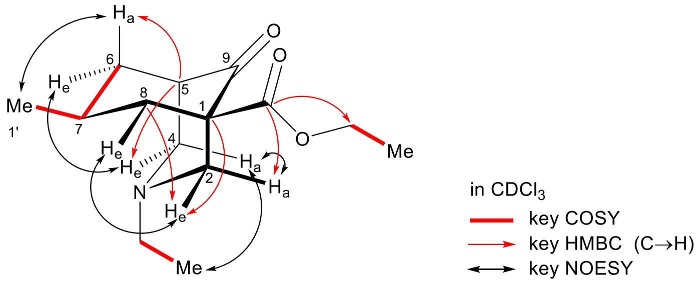

Methyl 3-ethyl-7-methyl-9-oxo-3-azabicyclo[3.3.1]nonane-1-carboxylate (**17**)

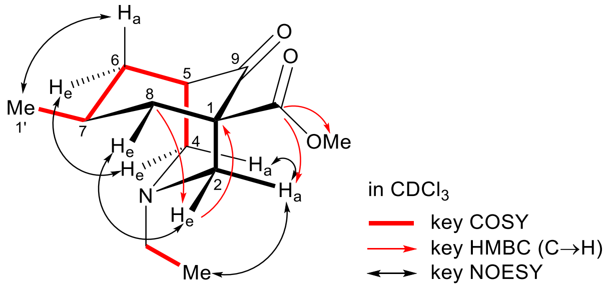

Ethyl (*E*)-9-(2-(2,4-dinitrophenyl)hydrazinylidene)-3-ethyl-3-azabicyclo[3.3.1]nonane-1-carboxylate (**18**)

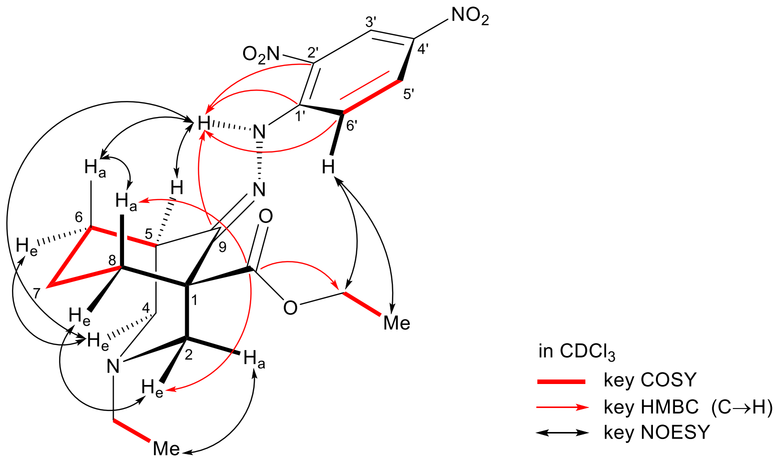

Methyl (*E*)-9-(2-(2,4-dinitrophenyl)hydrazinylidene)-3-ethyl-7-isopropyl-3-azabicyclo[3.3.1]nonane-1-carboxylate (**19**)

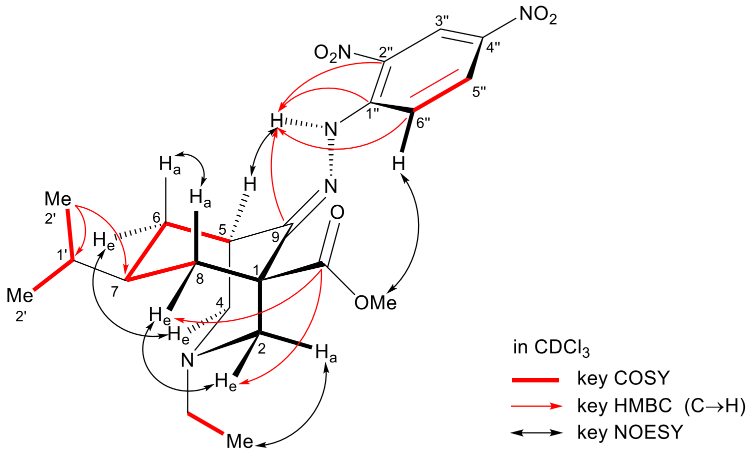

Methyl (*E*)-9-(2-(2,4-dinitrophenyl)hydrazinylidene)-3-ethyl-7-methyl-3-azabicyclo[3.3.1]nonane-1-carboxylate (**20**)

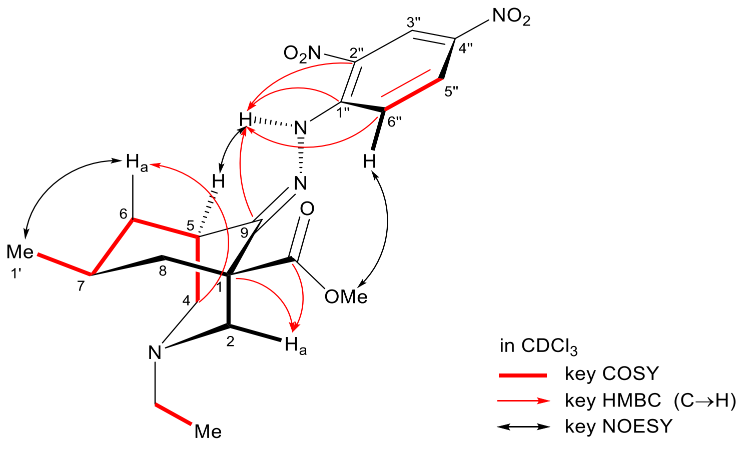

1-(2,4-dinitrophenyl)-2-(propan-2-ylidene)hydrazine

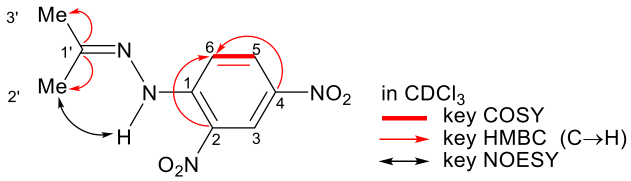

Methyl 8-ethyl-10-oxo-8-azabicyclo[4.3.1]decane-1-carboxylate (**23**)

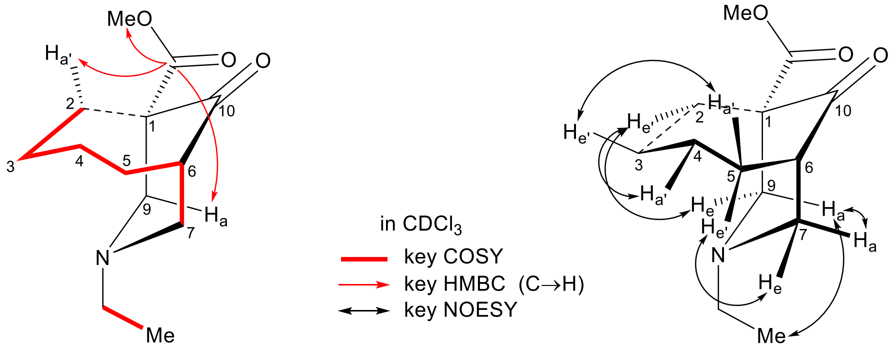

Methyl (*E*)-10-(2-(2,4-dinitrophenyl)hydrazinylidene)-8-ethyl-8-azabicyclo[4.3.1]decane-1-carboxylate (**24**)

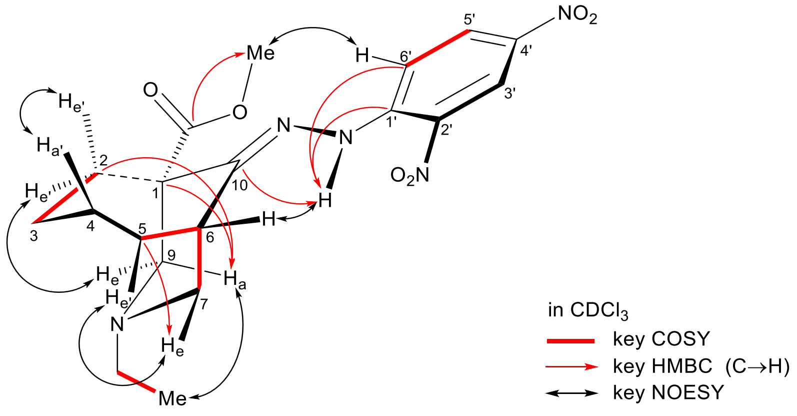

Ethyl 3-ethyl-8-oxo-3-azabicyclo[3.2.1]octane-1-carboxylate (**27**)

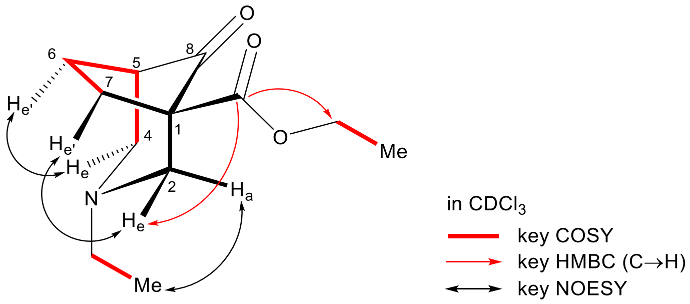

Ethyl (*E*)-8-(2-(2,4-dinitrophenyl)hydrazinylidene)-3-ethyl-3-azabicyclo[3.2.1]octane-1-carboxylate (**28**)

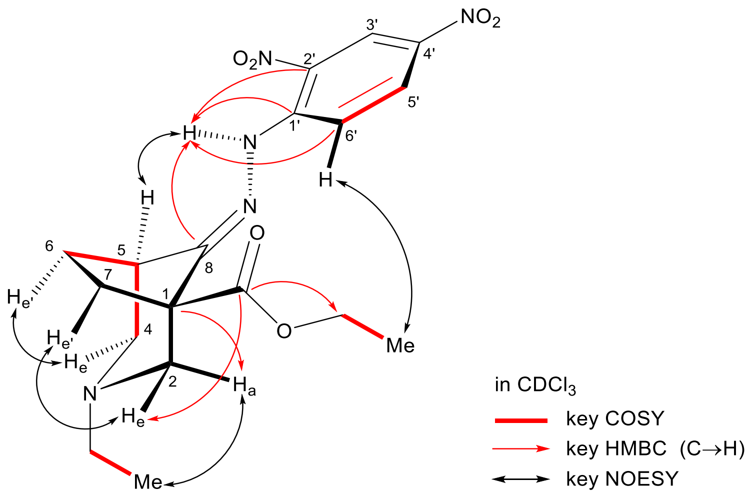

Ethyl 1-[(ethylamino)methyl]-2-oxocyclohexane-1-carboxylate (**29**)

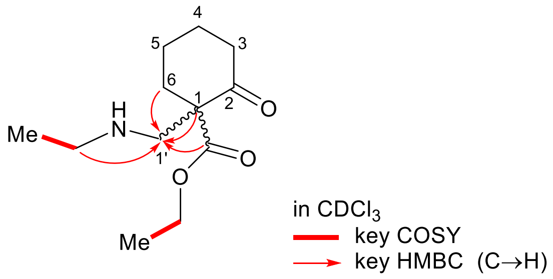

3-Ethyl-1-(hydroxymethyl)-3-azabicyclo[3.3.1]nonan-9-ol (**30**)

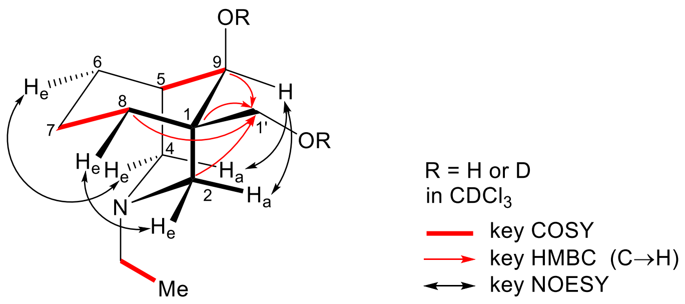

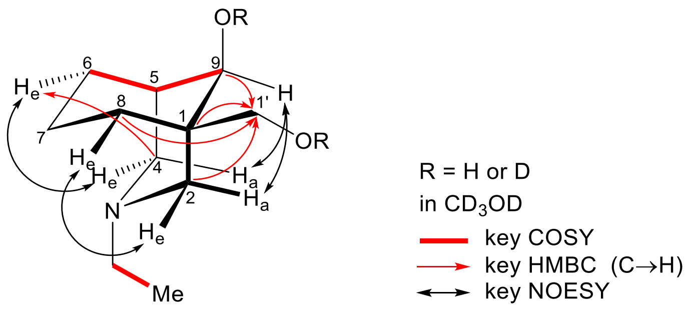

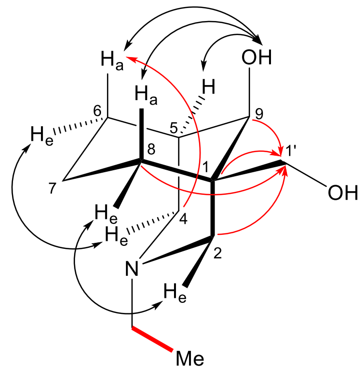

in d_6-DMSO

key COSY

key HMBC (C→H)

key NOESY

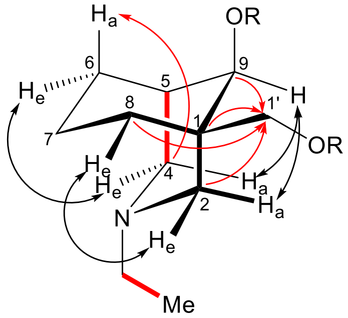

R = H or D

in D_2O (with 2drops of d_6-DMSO)

key COSY

key HMBC (C→H)

key NOESY

1-(Ethoxycarbonyl)-3-ethyl-9-oxo-3-azabicyclo[3.3.1]nonan-3-ium acetate salt (**31**)

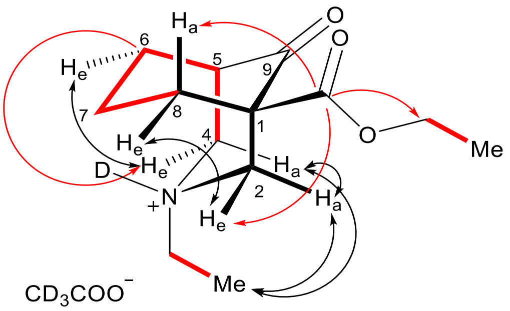

in d_4-acetic acid

key COSY

key HMBC (C→H)

key NOESY

1-(Ethoxycarbonyl)-3-ethyl-9-oxo-3-azabicyclo[3.3.1]nonan-3-ium chloride (**32**)

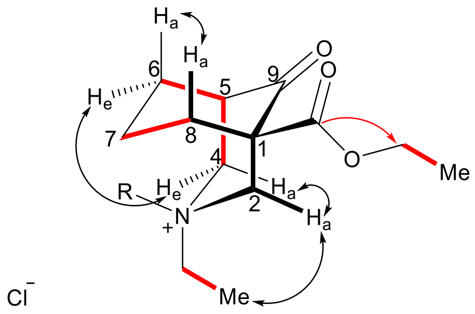

R = H or D

in d_4-acetic acid

key COSY

key HMBC (C→H)

key NOESY

1-(Ethoxycarbonyl)-3-ethyl-9,9-dihydroxy-3-azabicyclo[3.3.1]nonan-3-ium chloride (**33**)

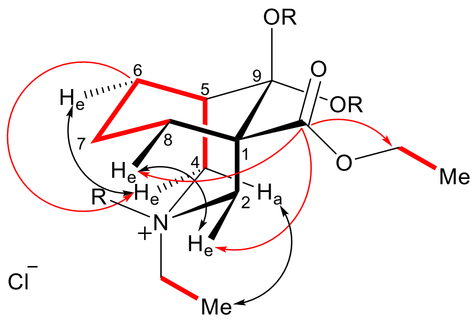

R = H or D
in D_2O
key COSY
key HMBC (C→H)
key NOESY

1-(Ethoxycarbonyl)-3-ethyl-3-methyl-9-oxo-3-azabicyclo[3.3.1]nonan-3-ium iodide (**34**)

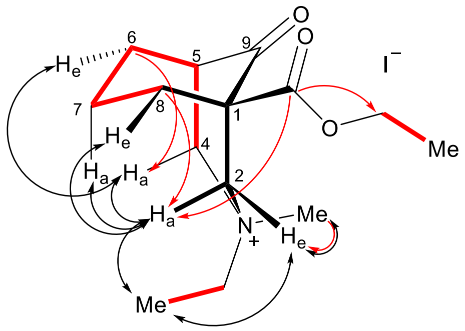

in $CDCl_3$
key COSY
key HMBC (C→H)
key NOESY

((1*R*,5*S*,9*s*))-9-Hydroxy-3-oxabicyclo[3.3.1]nonane-1,5-diyl) dimethanol (**36**)

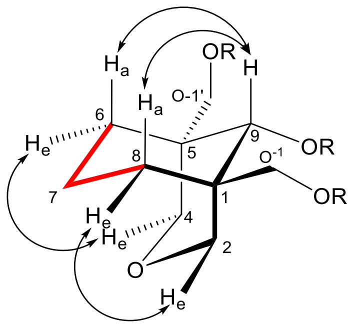

R = H or D
in CD_3OD
key COSY
key NOESY

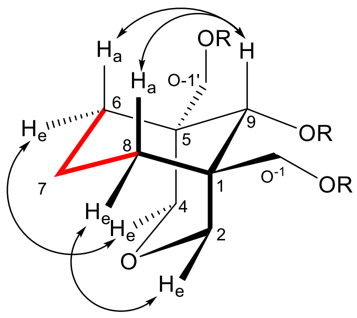

R = H or D
in D_2O
key COSY
key NOESY

2. Variable temperature (VT) ^{1}H NMR experiments

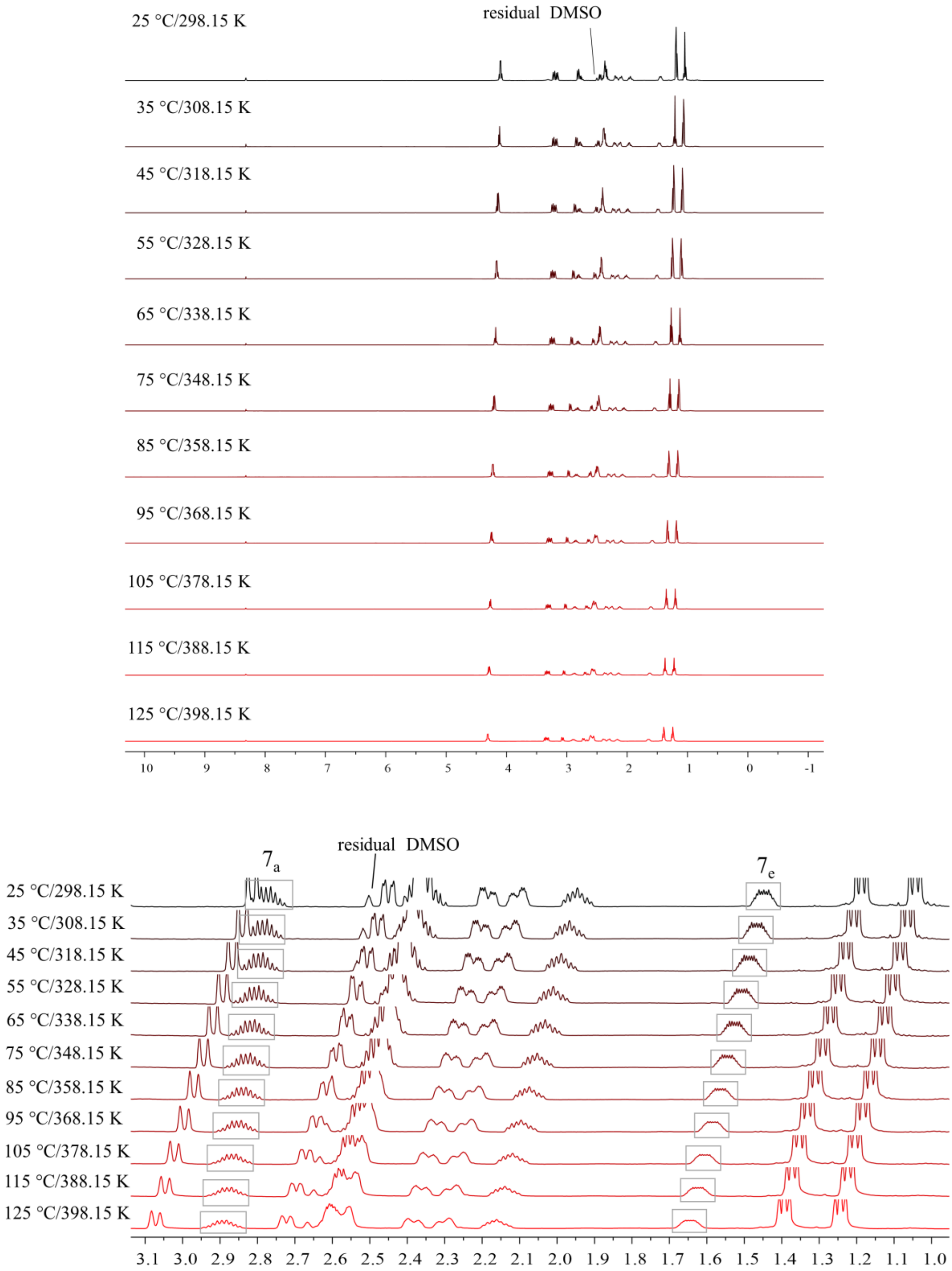

Figure S1. (*Upper*) VT ^{1}H NMR experiments of [3.3.1]azabicycle (**8**) in d_6-DMSO: the experiments were started at 25 °C (298.15 K, the first row) and finished at 125 °C (398.15 K, the last row) with temperature increases in steps of 10 °C between each experiment.

(*Lower*) Expansions of VT ^{1}H NMR experiments of [3.3.1]azabicycle (**8**) in d_6-DMSO.

3. Recrystallization and data of single-crystal X-ray determinations

Table S1. Conditions of recrystallization and SXRD data content

Code (CCDC no.)	Compound			Table
	Solvent system	Time (approximate)	Appearance and colour	
**s17phar5** (1975722)	Ethyl (1*S*,5*S*,*E*)-9-(2-(2,4-dinitrophenyl)hydrazinylidene)-3-ethyl-3-azabicyclo[3.3.1] nonane-1-carboxylate (**18**)			S2
	MeOH	3 days	block-shaped, yellow	
**s17phar6** (1975720)	Methyl (1*R*,5*R*,7*S*,*E*)-9-(2-(2,4-dinitrophenyl)hydrazinylidene)-3-ethyl-7-isopropyl-3-azabicyclo [3.3.1]nonane-1-carboxylate (**19**)			S3
	MeOH/Et_2O	2 days	needle-shaped, yellow	
**s18phar4** (1975723)	Methyl (1*R*,5*R*,7*S*,*E*)-9-(2-(2,4-dinitrophenyl)hydrazinylidene)-3-ethyl-7-methyl-3-azabicyclo [3.3.1]nonane-1-carboxylate (**20a**) and methyl (1*S*,5*S*,7*R*,*E*)-9-(2-(2,4-dinitrophenyl) hydrazinylidene)-3-ethyl-7-methyl-3-azabicyclo[3.3.1]nonane-1-carboxylate (**20b**) (twin-packed)			S4
	MeOH/Et_2O	4 days	needle-shaped, yellow	
**s17phar15** (1975725)	Methyl (1*R*,6*R*,*E*)-10-(2-(2,4-dinitrophenyl)hydrazinylidene)-8-ethyl-8-azabicyclo[4.3.1]decane-1-carboxylate (**24a**) and methyl (1*S*,6*S*,*E*)-10-(2-(2,4-dinitrophenyl)hydrazinylidene)-8-ethyl-8-azabicyclo [4.3.1]decane-1-carboxylate (**24b**) (twin-packed)			S5
	MeOH/Et_2O	a week	needle-shaped, yellow	
**s18phar3** (1975721)	(1*S*,5*S*)-1-(Ethoxycarbonyl)-3-ethyl-9-oxo-3-azabicyclo[3.3.1]nonan-3-ium chloride (**32**)			S6
	EtOAc/$CHCl_3$/MeOH	3 weeks	block-shaped, brown	
**s18phar11** (1975724)	((1*R*,5*S*,9*s*)-9-Hydroxy-3-oxabicyclo[3.3.1]nonane-1,5-diyl) dimethanol (**36**)			S7
	EtOAc	14 h	block-shaped, white	

Table S2. Crystal data and structure refinement for ethyl (1*S*,5*S*,*E*)-9-(2-(2,4-dinitrophenyl)hydrazinylidene)-3-ethyl-3-azabicyclo[3.3.1] nonane-1-carboxylate (**18**) (**s17phar5**).

Identification code	s17phar5	
Empirical formula	C19 H25 N5 O6	
Formula weight	419.44	
Temperature	150.00(10) K	
Wavelength	1.54184 Å	
Crystal system	Monoclinic	
Space group	I2/a	
Unit cell dimensions	a = 26.8708(18) Å	α= 90°.
	b = 7.3323(7) Å	β= 95.548(6)°.
	c = 20.6528(14) Å	γ = 90°.
Volume	4050.1(5) $Å^3$	
Z	8	
Density (calculated)	1.376 Mg/m^3	
Absorption coefficient	0.872 mm^{-1}	
F(000)	1776	
Crystal size	0.100 × 0.080 × 0.020 mm^3	
Theta range for data collection	3.305 to 73.477°.	
Index ranges	-33<=h<=31, -7<=k<=8, -22<=l<=25	
Reflections collected	13152	
Independent reflections	4023 [R(int) = 0.0446]	
Completeness to theta = 67.684°	100.0 %	
Absorption correction	Semi-empirical from equivalents	

Max. and min. transmission	1.00000 and 0.84944
Refinement method	Full-matrix least-squares on F^2
Data / restraints / parameters	4023 / 0 / 277
Goodness-of-fit on F^2	1.020
Final R indices [I>2sigma(I)]	R1 = 0.0537, wR2 = 0.1391
R indices (all data)	R1 = 0.0744, wR2 = 0.1534
Extinction coefficient	n/a
Largest diff. peak and hole	0.421 and -0.227 e.Å^{-3}

Table S3. Crystal data and structure refinement for methyl (1*R*,5*R*,7*S*,*E*)-9-(2-(2,4-dinitrophenyl)hydrazinylidene)-3-ethyl-7-isopropyl-3-azabicyclo [3.3.1]nonane-1-carboxylate (**19**) (**s17phar6**).

Identification code	s17phar6	
Empirical formula	C21 H29 N5 O6	
Formula weight	447.49	
Temperature	150.01(10) K	
Wavelength	1.54184 Å	
Crystal system	Monoclinic	
Space group	I2/a	
Unit cell dimensions	a = 7.74460(10) Å	α= 90°.
	b = 15.5385(2) Å	β= 91.337(2)°.
	c = 36.6455(7) Å	γ = 90°.
Volume	4408.70(12) Å^3	
Z	8	
Density (calculated)	1.348 Mg/m^3	
Absorption coefficient	0.834 mm^{-1}	
F(000)	1904	
Crystal size	0.200 × 0.100 × 0.040 mm^3	
Theta range for data collection	3.089 to 73.175°.	
Index ranges	-9<=h<=6, -19<=k<=19, -43<=l<=45	
Reflections collected	25235	
Independent reflections	4398 [R(int) = 0.0329]	
Completeness to theta = 67.684°	100.0 %	
Absorption correction	Semi-empirical from equivalents	
Max. and min. transmission	1.00000 and 0.83146	
Refinement method	Full-matrix least-squares on F^2	
Data / restraints / parameters	4398 / 0 / 308	
Goodness-of-fit on F^2	1.076	
Final R indices [I>2sigma(I)]	R1 = 0.0470, wR2 = 0.1337	
R indices (all data)	R1 = 0.0509, wR2 = 0.1367	
Extinction coefficient	n/a	
Largest diff. peak and hole	0.371 and -0.433 e.Å^{-3}	

Table S4. Crystal data and structure refinement for methyl (1*R*,5*R*,7*S*,*E*)-9-(2-(2,4-dinitrophenyl)hydrazinylidene)-3-ethyl-7-methyl-3-azabicyclo [3.3.1]nonane-1-carboxylate (**20a**) and methyl (1*S*,5*S*,7*R*,*E*)-9-(2-(2,4-dinitrophenyl) hydrazinylidene)-3-ethyl-7-methyl-3-azabicyclo[3.3.1]nonane-1-carboxylate (twin-packed) (**20b**) (**s18phar4**).

Identification code	s18phar4	
Empirical formula	C18.60 H23.80 N5 O6	
Formula weight	413.42	
Temperature	149.9(4) K	
Wavelength	1.54184 Å	
Crystal system	Triclinic	
Space group	P-1	
Unit cell dimensions	a = 7.7392(4) Å	α= 110.066(11)°.
	b = 15.8949(16) Å	β= 90.729(7)°.
	c = 17.396(2) Å	γ = 90.190(7)°.
Volume	2009.8(4) Å^3	
Z	4	
Density (calculated)	1.366 Mg/m^3	
Absorption coefficient	0.871 mm^{-1}	
F(000)	874	
Crystal size	0.425 × 0.039 × 0.025 mm^3	
Theta range for data collection	2.705 to 69.746°.	
Index ranges	-9<=h<=9, -18<=k<=19, -20<=l<=20	
Reflections collected	11165	
Independent reflections	11165 [R(int) = ?]	
Completeness to theta = 67.684°	100.0 %	
Absorption correction	Gaussian	
Max. and min. transmission	0.981 and 0.838	
Refinement method	Full-matrix least-squares on F^2	
Data / restraints / parameters	11165 / 2 / 556	
Goodness-of-fit on F^2	0.786	
Final R indices [I>2sigma(I)]	R1 = 0.0621, wR2 = 0.1311	
R indices (all data)	R1 = 0.1407, wR2 = 0.1536	
Extinction coefficient	n/a	
Largest diff. peak and hole	0.714 and -0.310 e.Å^{-3}	

Table S5. Crystal data and structure refinement for methyl (1*R*,6*R*,*E*)-10-(2-(2,4-dinitrophenyl)hydrazinylidene)-8-ethyl-8-azabicyclo[4.3.1]decane-1-carboxylate (**24a**) and methyl (1*S*,6*S*,*E*)-10-(2-(2,4-dinitrophenyl)hydrazinylidene)-8-ethyl-8-azabicyclo [4.3.1]decane-1-carboxylate (twin-packed) and (**24b**) (**s17phar15**).

Identification code	s17phar15	
Empirical formula	C19 H25 N5 O6	
Formula weight	419.44	
Temperature	150.01(10) K	
Wavelength	1.54184 Å	
Crystal system	Triclinic	
Space group	P-1	
Unit cell dimensions	a = 8.2535(2) Å	α= 100.206(3)°.
	b = 14.1548(4) Å	β= 99.075(3)°.

	c = 18.1606(7) Å	γ = 102.165(2)°.
Volume	1998.79(11) $Å^3$	
Z	4	
Density (calculated)	1.394 Mg/m^3	
Absorption coefficient	0.883 mm^{-1}	
F(000)	888	
Crystal size	0.200 × 0.150 × 0.020 mm^3	
Theta range for data collection	3.273 to 72.332°.	
Index ranges	-10<=h<=9, -17<=k<=17, -22<=l<=22	
Reflections collected	22201	
Independent reflections	7805 [R(int) = 0.0391]	
Completeness to theta = 67.684°	100.0 %	
Absorption correction	Semi-empirical from equivalents	
Max. and min. transmission	1.00000 and 0.73157	
Refinement method	Full-matrix least-squares on F^2	
Data / restraints / parameters	7805 / 0 / 553	
Goodness-of-fit on F^2	1.046	
Final R indices [I>2sigma(I)]	R1 = 0.0495, wR2 = 0.1240	
R indices (all data)	R1 = 0.0703, wR2 = 0.1369	
Extinction coefficient	n/a	
Largest diff. peak and hole	0.399 and -0.224 e.$Å^{-3}$	

Table S6. Crystal data and structure refinement for (1*S*,5*S*)-1-(Ethoxycarbonyl)-3-ethyl-9-oxo-3-azabicyclo[3.3.1]nonan-3-ium chloride (**32**) (**s18phar3**).

Identification code	s18phar3	
Empirical formula	C13 H22 Cl N O3	
Formula weight	275.76	
Temperature	150.00(10) K	
Wavelength	1.54184 Å	
Crystal system	Orthorhombic	
Space group	Pbca	
Unit cell dimensions	a = 14.0556(3) Å	α= 90°.
	b = 8.4265(2) Å	β= 90°.
	c = 23.4225(6) Å	γ = 90°.
Volume	2774.15(11) $Å^3$	
Z	8	
Density (calculated)	1.321 Mg/m^3	
Absorption coefficient	2.454 mm^{-1}	
F(000)	1184	
Crystal size	0.230 × 0.200 × 0.080 mm^3	
Theta range for data collection	3.774 to 73.497°.	
Index ranges	-17<=h<=16, -10<=k<=7, -28<=l<=28	
Reflections collected	26135	
Independent reflections	2777 [R(int) = 0.0769]	
Completeness to theta = 67.684°	100.0 %	
Absorption correction	Semi-empirical from equivalents	
Max. and min. transmission	1.00000 and 0.78003	

Refinement method	Full-matrix least-squares on F^2
Data / restraints / parameters	2777 / 0 / 169
Goodness-of-fit on F^2	1.065
Final R indices [I>2sigma(I)]	R1 = 0.0449, wR2 = 0.1170
R indices (all data)	R1 = 0.0465, wR2 = 0.1185
Extinction coefficient	n/a
Largest diff. peak and hole	0.450 and -0.353 e.Å^{-3}

Table S7. Crystal data and structure refinement for ((1*R*,5*S*,9*s*)-9-Hydroxy-3-oxabicyclo[3.3.1]nonane-1,5-diyl) dimethanol (**36**) (**s18phar11**).

Identification code	s18phar11	
Empirical formula	C10 H18 O4	
Formula weight	202.24	
Temperature	150.01(10) K	
Wavelength	1.54184 Å	
Crystal system	Monoclinic	
Space group	$P2_1$	
Unit cell dimensions	a = 5.9795(2) Å	α= 90°.
	b = 10.4226(5) Å	β= 95.080(4)°.
	c = 8.2748(3) Å	γ = 90°.
Volume	513.68(4) Å^3	
Z	2	
Density (calculated)	1.308 Mg/m^3	
Absorption coefficient	0.828 mm^{-1}	
F(000)	220	
Crystal size	0.160 × 0.120 × 0.080 mm^3	
Theta range for data collection	5.367 to 73.299°.	
Index ranges	-7<=h<=7, -12<=k<=12, -10<=l<=10	
Reflections collected	7894	
Independent reflections	1990 [R(int) = 0.0342]	
Completeness to theta = 67.684°	100.0 %	
Absorption correction	Semi-empirical from equivalents	
Max. and min. transmission	1.00000 and 0.85223	
Refinement method	Full-matrix least-squares on F^2	
Data / restraints / parameters	1990 / 1 / 139	
Goodness-of-fit on F^2	1.052	
Final R indices [I>2sigma(I)]	R1 = 0.0297, wR2 = 0.0764	
R indices (all data)	R1 = 0.0306, wR2 = 0.0773	
Absolute structure parameter	0.12(10)	
Extinction coefficient	n/a	
Largest diff. peak and hole	0.220 and -0.153 e.Å^{-3}	

4. Key ^{1}H NMR data related to steric compression

Table S8. $\delta_{7\text{-H}}$ (ppm) of [3.3.1]azabicycle (**8**) in 3 solvents

Solvent	δ (7-H_a)	δ (7-H_e)	$\Delta\delta_{7\text{-H}}$
$CDCl_3$	2.86	1.53	1.33
CD_3OD	2.88	1.52	1.36
d$_6$-DMSO	2.77	1.45	1.32

Table S9. $\delta_{7\text{-H}}$ (ppm) of [3.3.1]azabicycles (**8**), (**12**), and (**17**) and their DNP derivatives (**18**−**20**) ($CDCl_3$, ppm)

(**8**)		7-iPr (**12**)	7-Me (**17**)
δ (7-Ha)	2.86	3.02	3.42
δ (7-He)	1.53		
(**18**)		7-iPr (**19**)	7-Me (**20**)
δ (7-Ha)	2.89	2.97	3.28
δ (7-He)	1.58		

Table S10. Key ^{1}H NMR of [4.3.1]- and [3.2.1]azabicycle (**23**) and (**27**) and their DNP derivatives (**24**) and (**28**) ($CDCl_3$, ppm)

[4.3.1]azabicycle (**23**)				[3.2.1]azabicycle (**27**)			
δ (3-$H_{a'}$)	2.04	δ (4-$H_{a'}$)	1.96	δ (6-$H_{e'}$)	1.95	δ (7-$H_{e'}$)	2.38
δ (3-$H_{e'}$)	1.36	δ (4-$H_{e'}$)	1.51	δ (6-$H_{a'}$)	1.95	δ (7-$H_{a'}$)	2.25
$\Delta\delta_{3\text{-H}}$	0.68	$\Delta\delta_{4\text{-H}}$	0.45	$\Delta\delta_{6\text{-H}}$	0.00	$\Delta\delta_{7\text{-H}}$	0.13
[4.3.1]azabicyclic DNP derivative (**24**)				[4.3.1]azabicyclic DNP derivative (**28**)			
δ (3-$H_{a'}$)	2.05	δ (4-$H_{a'}$)	1.95	δ (6-$H_{e'}$)	2.07	δ (7-$H_{e'}$)	2.37
δ (3-$H_{e'}$)	1.42	δ (4-$H_{e'}$)	1.39	δ (6-$H_{a'}$)	1.94	δ (7-$H_{a'}$)	2.19
$\Delta\delta_{3\text{-H}}$	0.63	$\Delta\delta_{4\text{-H}}$	0.56	$\Delta\delta_{6\text{-H}}$	0.13	$\Delta\delta_{7\text{-H}}$	0.18

Table S11. Key NMR data comparison between compounds (**6**) and (**29**) ($CDCl_3$, ppm)

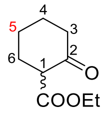

(**6**)

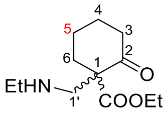

(**29**)

(**6**)	δ_{C3}	δ_{C4}	δ_{C5}
	41.57	27.12	23.31
(**29**)	δ_{C3}	δ_{C4}	δ_{C5}
	41.12	27.26	22.38
	$\delta_{5\text{-H}}{}^{a}$= 1.77, 1.68; $\Delta\delta_{5\text{-H}}$ = 0.09		

[a] no evidence was obtained to identify the orientation of 5-H

Table S12. Key ^{1}H NMR data of diol (**30**) in 4 solvents (ppm)

Solvent	δ (7-H_a)	δ (7-H_e)	$\Delta\delta_{7\text{-}H}$
$CDCl_3$	2.59	1.48	1.11
CD_3OD	2.58	1.42	1.16
d$_6$-DMSO	2.47	1.29	1.18
D_2O[a]	2.04	1.59	0.45

[a] with an additional 2 drops of *d*$_6$-DMSO

Table S13. Key ^{1}H NMR data of the salt (**31**–**33**) (ppm)

Compound	Solvent	$\delta_{7\text{-}H}$		$\Delta\delta_{7\text{-}H}$
ketone acetate salt (**31**)	*d*$_4$-acetic acid	2.42 (7-H_a)	1.72 (7-H_e)	0.70
ketone chloride salt (**32**)	*d*$_4$-acetic acid	2.60 (7-H_a)	1.73 (7-H_e)	0.87
ketal chloride salt (**33**)	D_2O[a]	1.72; 1.83[b]		0.11
	CD_3OD	1.78; 1.89[b]		0.11
	d$_6$-DMSO	1.55 ; 1.90[b]		0.35

[a] with an additional 2 drops of *d*$_6$-DMSO

[b] no reliable evidence was obtained to identify the orientation of these protons unambiguously

Table S14. $\delta_{7\text{-}H}$ (ppm) of oxabicycle (**36**) in different solvents

Solvent	δ (7-H_a)	δ (7-H_e)	$\Delta\delta_{7\text{-}H}$
CD_3OD	2.32	1.51	0.81
d$_6$-DMSO	2.18	1.38	0.80
d$_6$-acetone	2.35	1.43	0.92
D_2O	2.16	1.57	0.59

5. NMR spectra and MS data of synthetic compounds

Tautomers of ethyl 2-oxocyclohexane-1-carboxylate (**6**, keto) and ethyl 2-hydroxycyclohex-1-ene-1-carboxylate (**7**, enol)

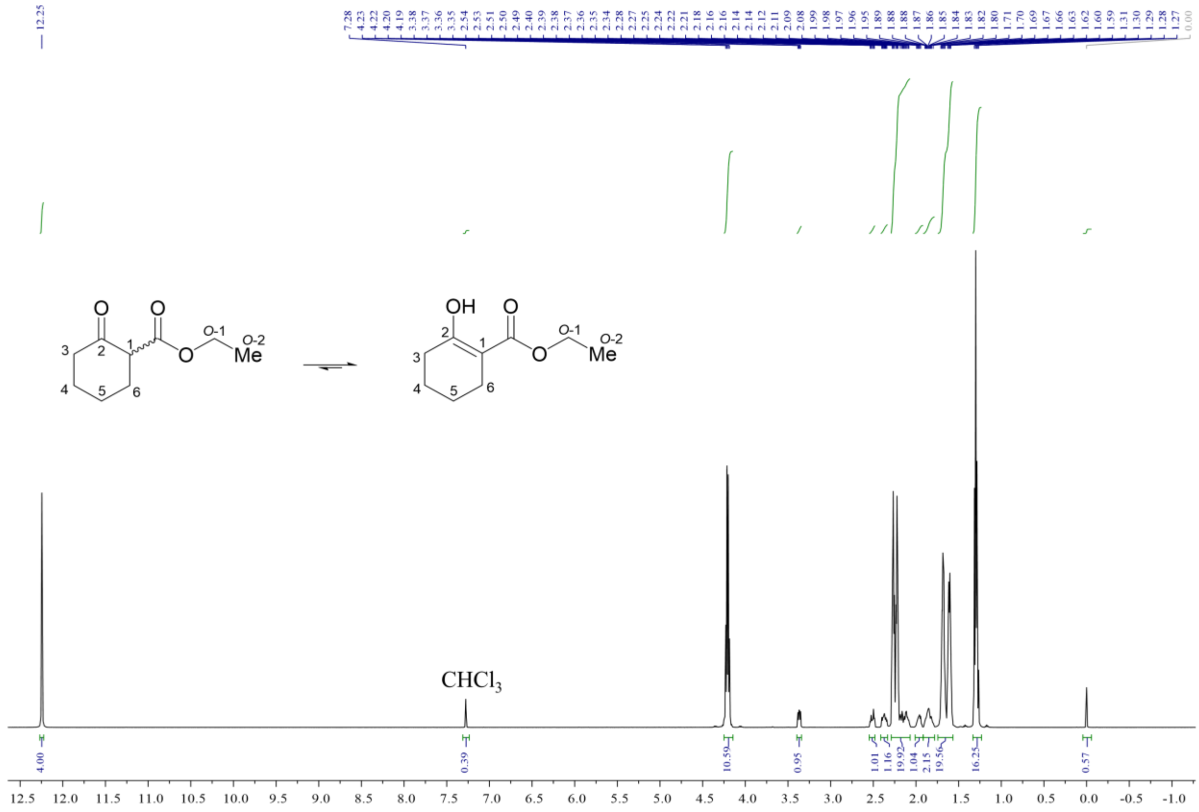

Figure S2. ^{1}H NMR spectrum of tautomers (**6**) and (**7**) in $CDCl_3$

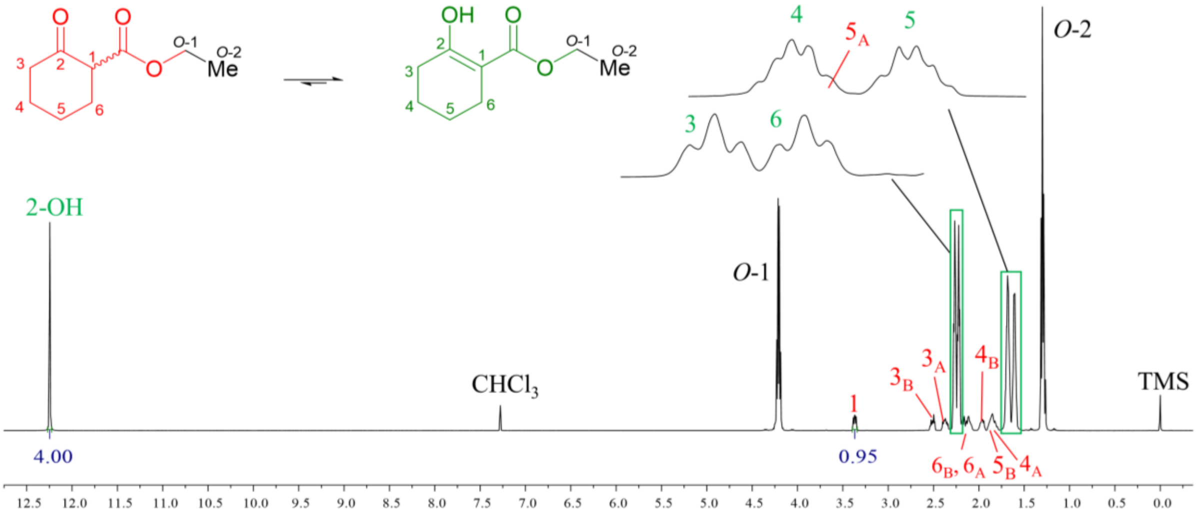

Figure S3. ^{1}H NMR expansion of tautomers (**6**) and (**7**) in $CDCl_3$ with assignments

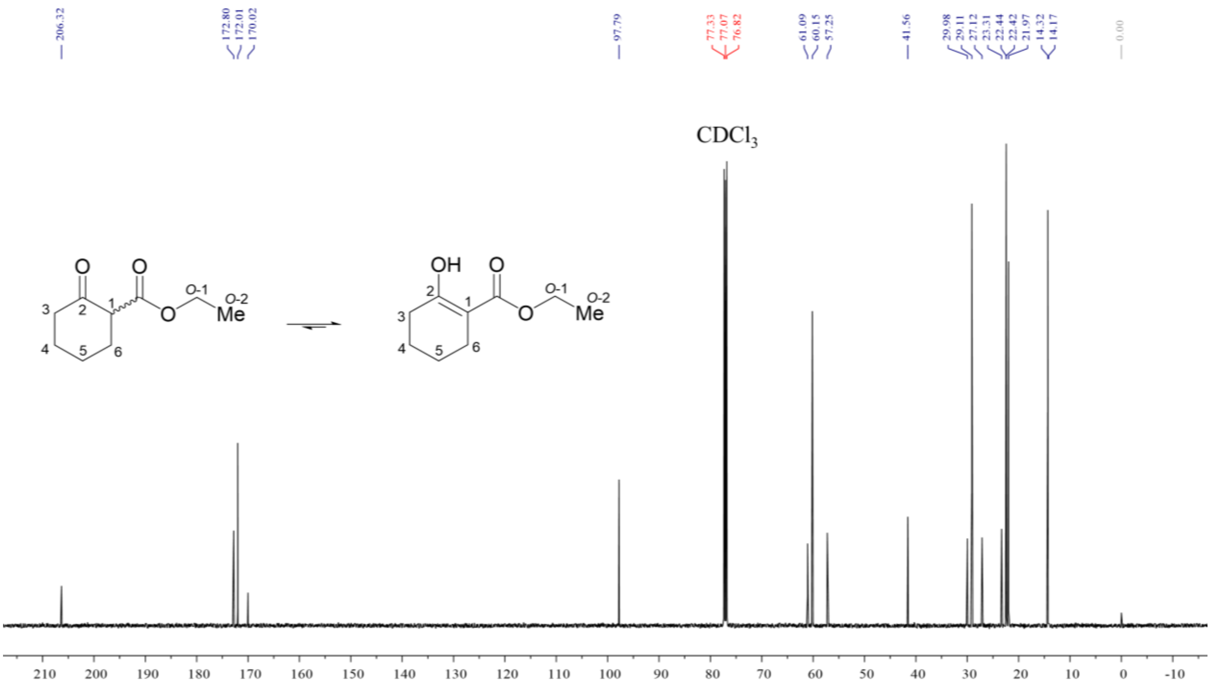

Figure S4. ^{13}C NMR spectrum of tautomers (**6**) and (**7**) in $CDCl_3$

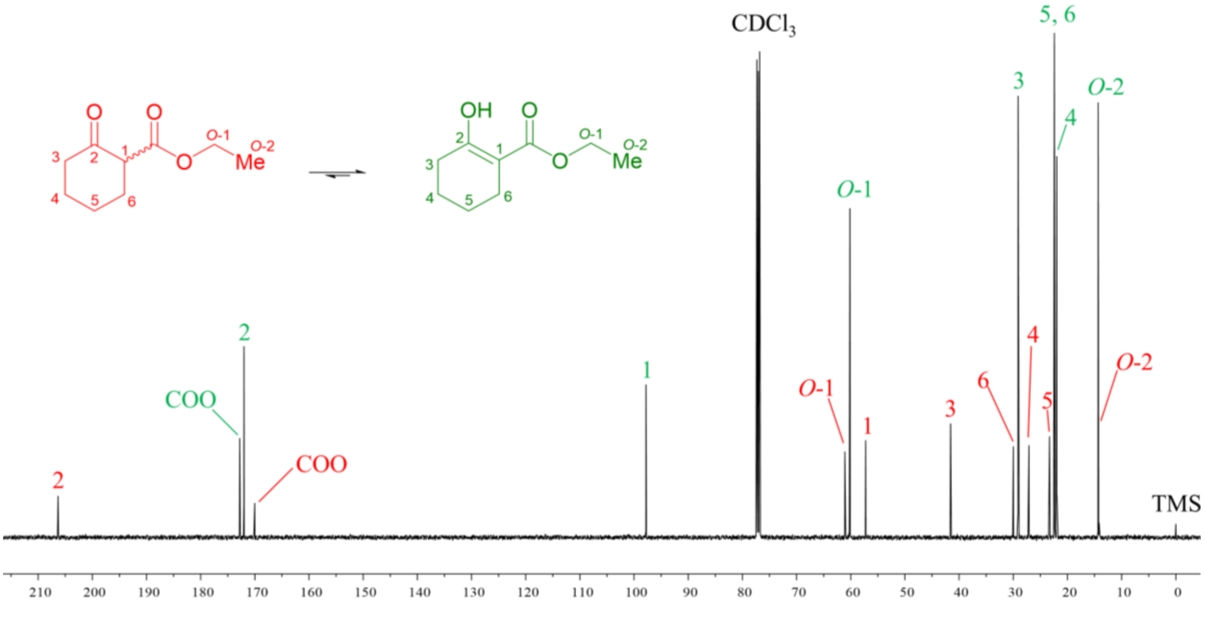

Figure S5. ^{13}C NMR expansion of tautomers (**6**) and (**7**) in $CDCl_3$ with assignments

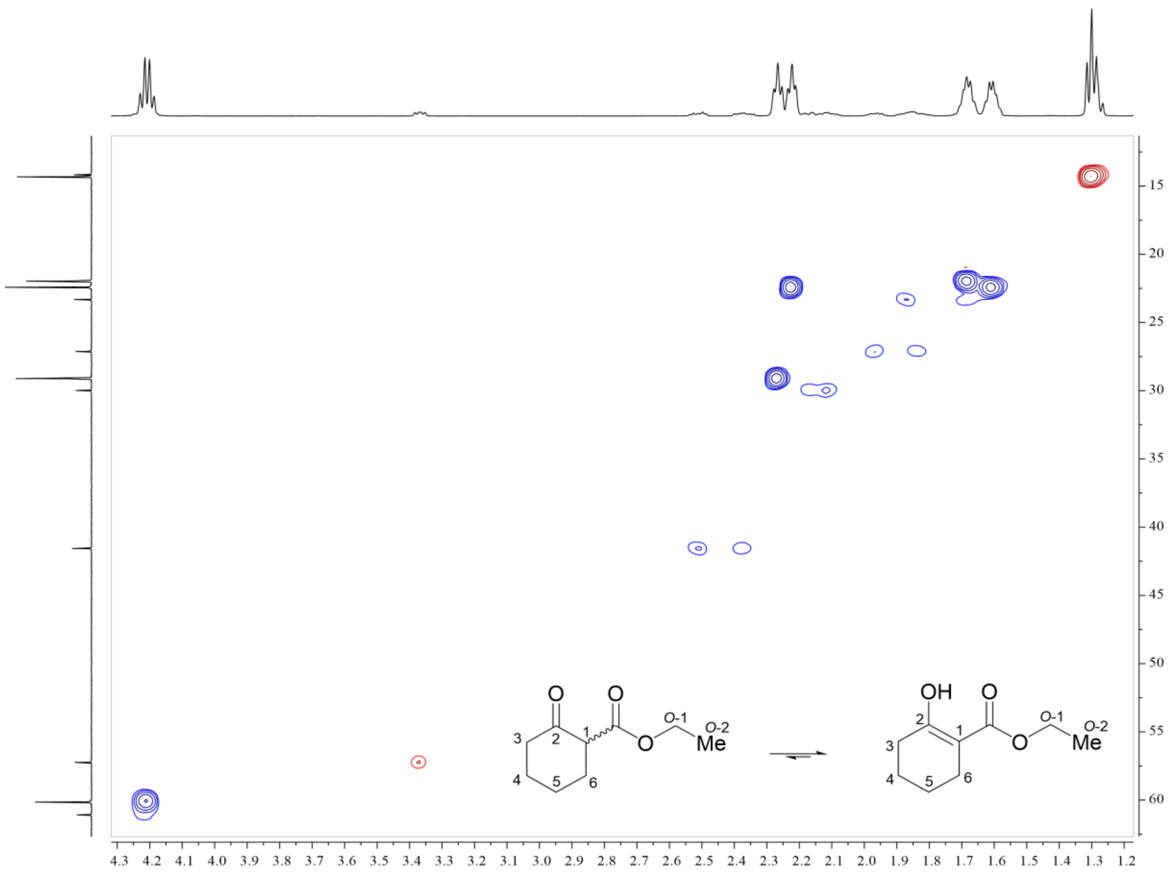

Figure S6. HSQC spectrum of tautomers (**6**) and (**7**) in $CDCl_3$

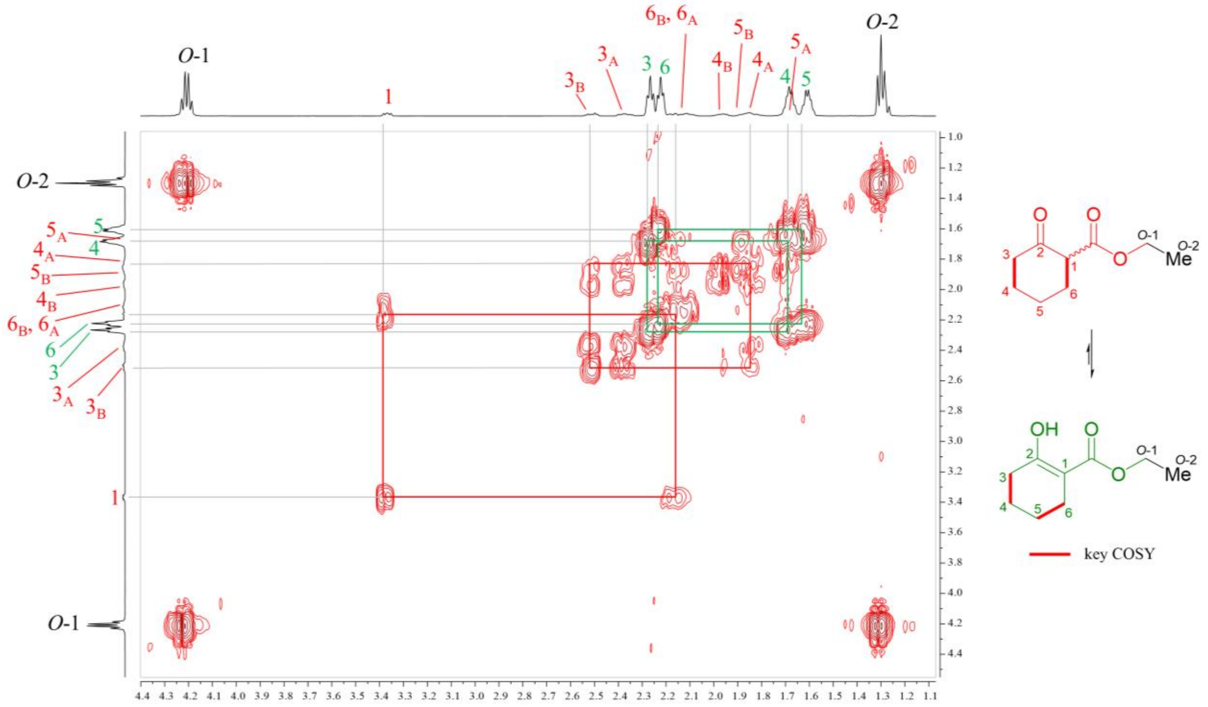

Figure S7. COSY spectrum of tautomers (**6**) and (**7**) in $CDCl_3$

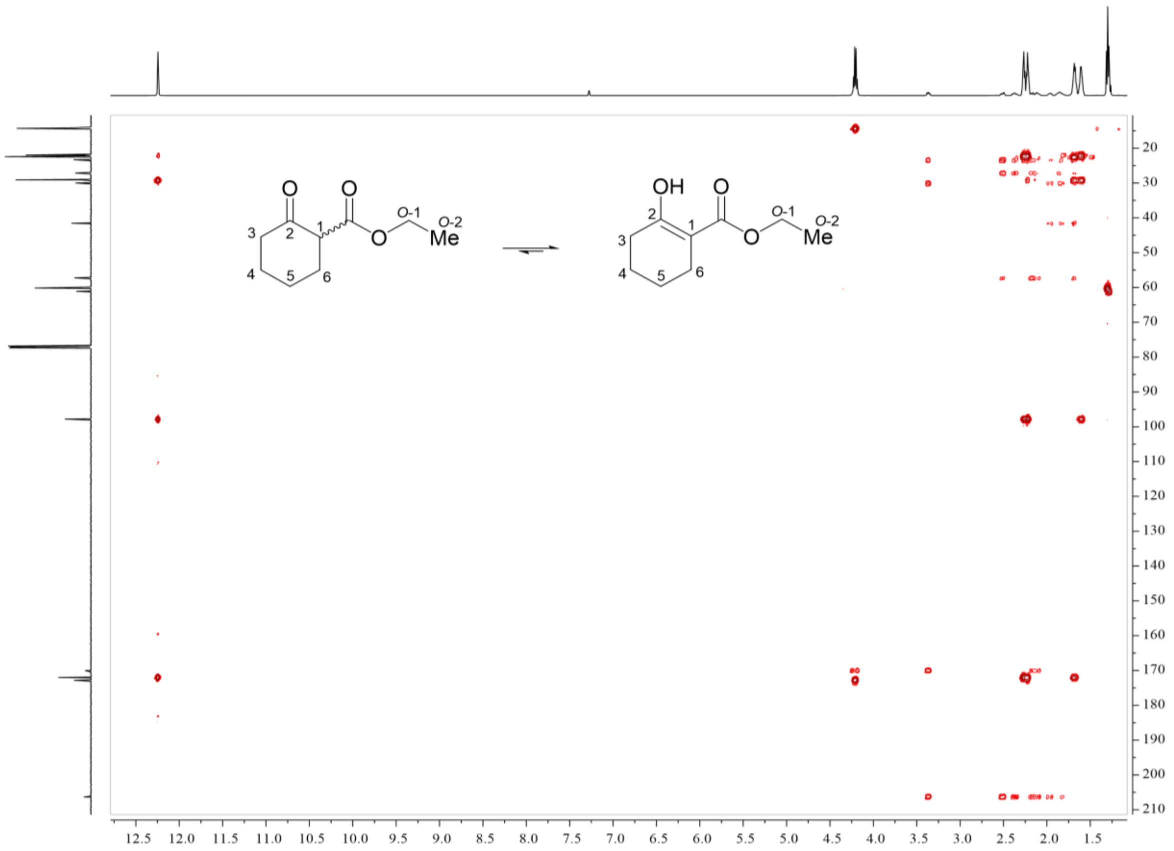

Figure S8. HMBC spectrum of tautomers (**6**) and (**7**) in $CDCl_3$

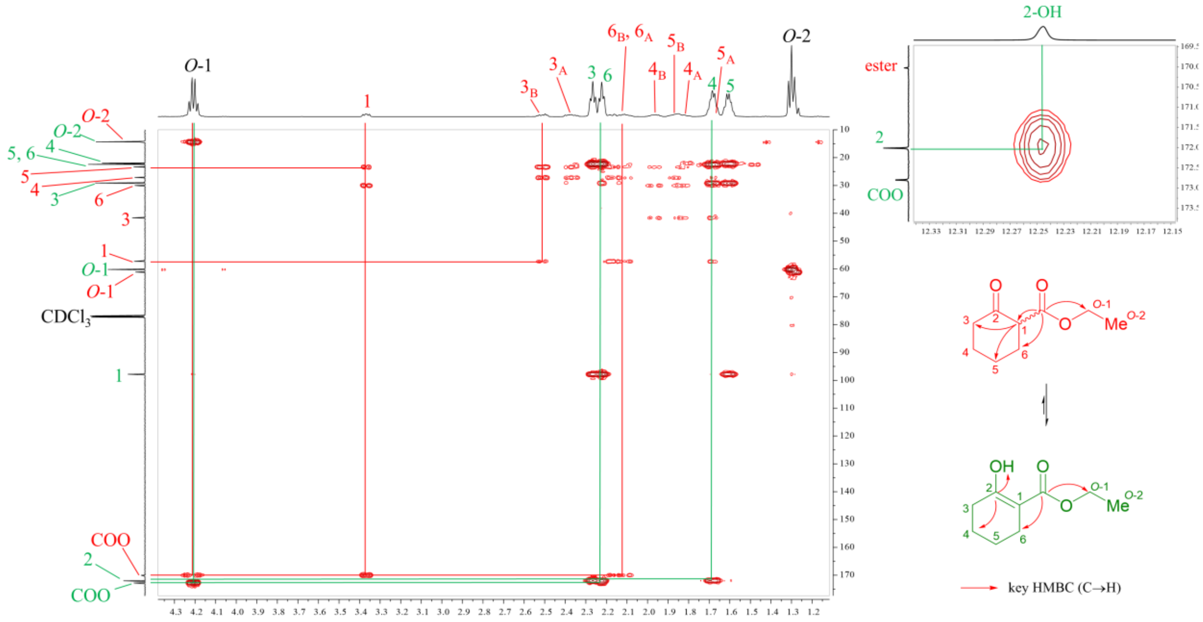

Figure S9. HMBC expansions of tautomers (**6**) and (**7**) in $CDCl_3$

Ethyl 3-ethyl-9-oxo-3-azabicyclo[3.3.1]nonane-1-carboxylate (**8**)

Confirmation of Expected Formula

Sample-ID	bl_zz_AE-MIX-2	Submitter	zz615 Ziyu Zeng
Analysis Name	bl_zz_AE-MIX-2_347529_97_01_52288.d	Supervisor	- Ian Blagborough
Method used	Confirm Formula Positive 50to500 loop inj.m	Acquisition Date	29/04/2016 10:20:03
Ionisation Mode	positive electrospray (ESI)		

+MS, 1.0-1.3min #(60-77), -Spectral Bkgrnd

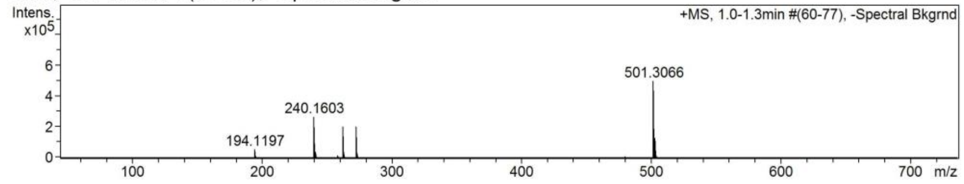

#	m/z	I	I %	Area	S/N
1	194.1197	55664	11.2	2065	21904.2
2	240.1603	265128	53.6	10180	5967.7
3	241.1622	37783	7.6	1875	828.0
4	262.1410	198756	40.2	8588	4278.8
5	263.1439	30185	6.1	1490	667.1
6	272.1847	200682	40.5	9085	5832.7
7	273.1871	28976	5.9	1706	872.6
8	501.3066	494924	100.0	21849	12814.5
9	502.3012	126778	25.6	11405	3398.2
10	503.3034	16644	3.4	1868	462.6

Generate Molecular Formula Parameters

Charge	Tolerance	SearchRadius	H/C Ratio min.	H/C Ratio max.	Electron Conf.	Nitrogen Rule	sigma limit
positive	10 ppm	0.05 m/z	0	3	both	true	0.05

Expected Formula C13 H21 N1 O3 **Adduct(s):** H, Na

#	meas. m/z	theo. m/z	Err[ppm]	Sigma	Formula
1	240.1603	240.159969	-3.80	0.0015	C 13 H 22 N 1 O 3
1	262.1410	262.141913	1.40	0.0045	C 13 H 21 N 1 Na 1 O 3

Note: Sigma fits < 0.05 indicates high probability of correct MF, and mass accuracy of 5ppm or better is generally acceptable for publication

Figure S10. MS data of [3.3.1]azabicycle (**8**)

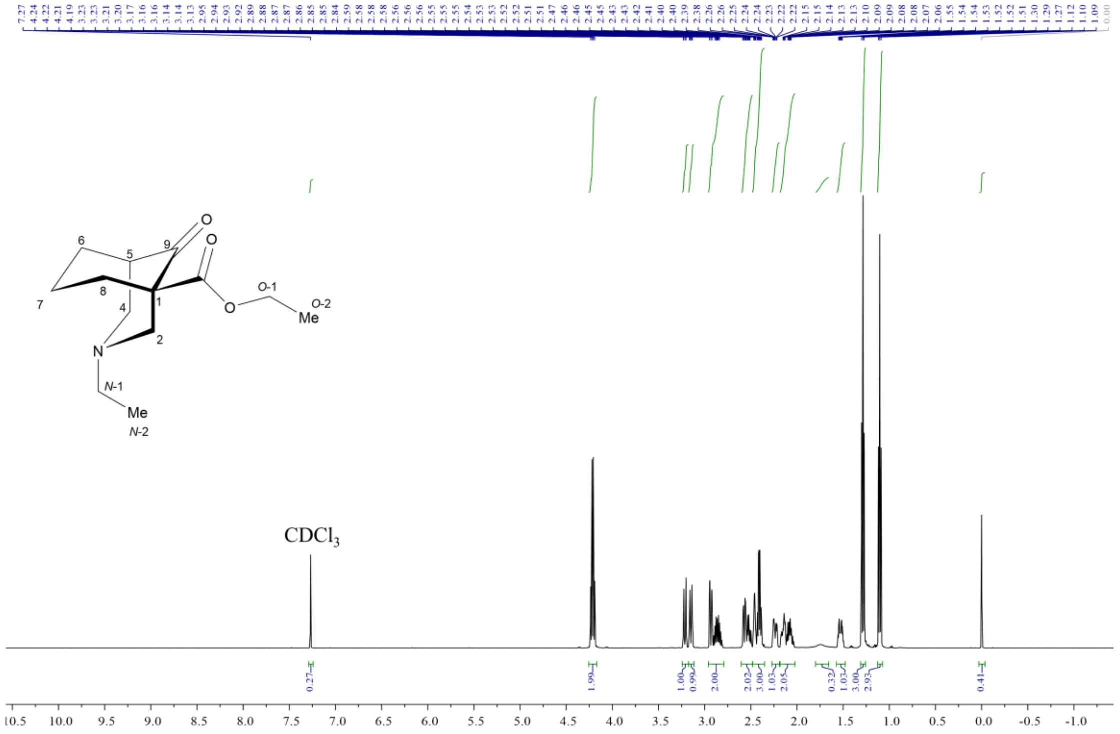

Figure S11. ^{1}H NMR spectrum of [3.3.1]azabicycle (**8**) in $CDCl_3$

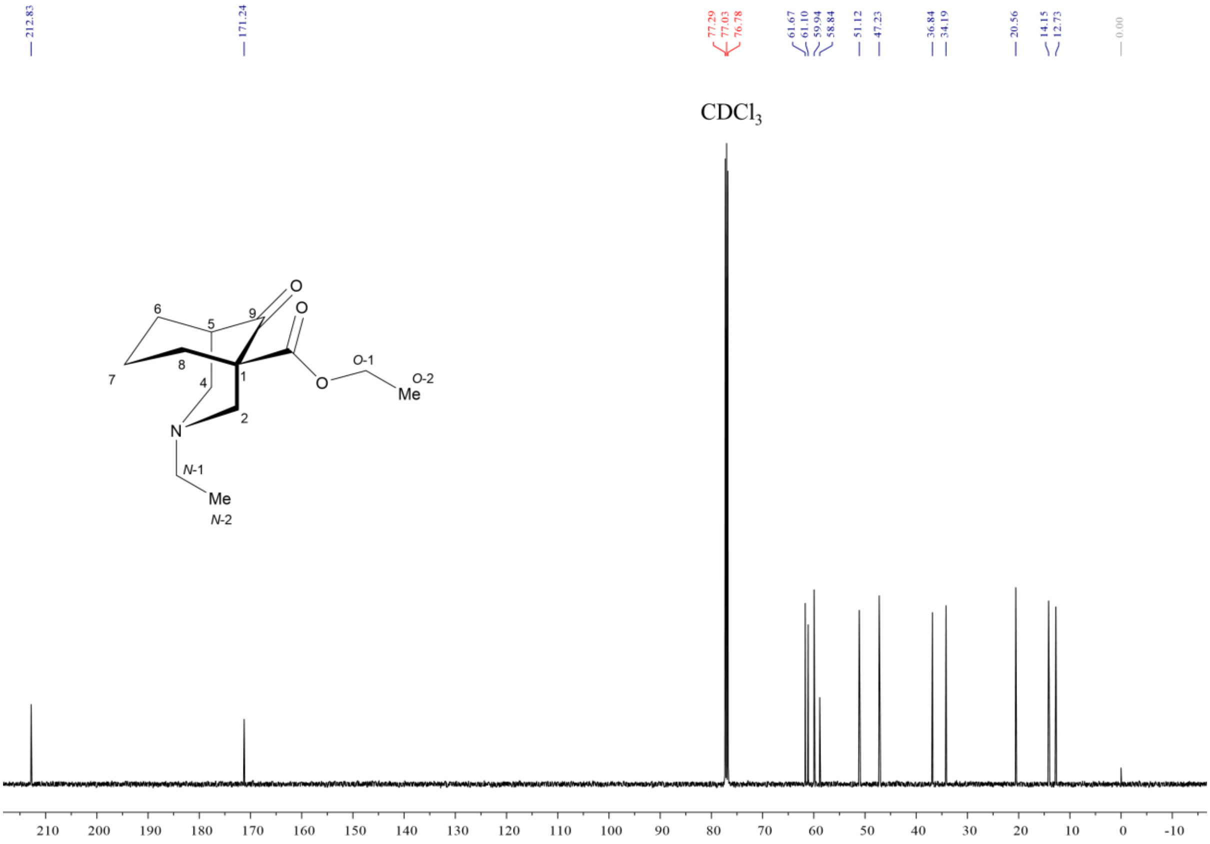

Figure S12. ^{13}C NMR spectrum of [3.3.1]azabicycle (**8**) in $CDCl_3$

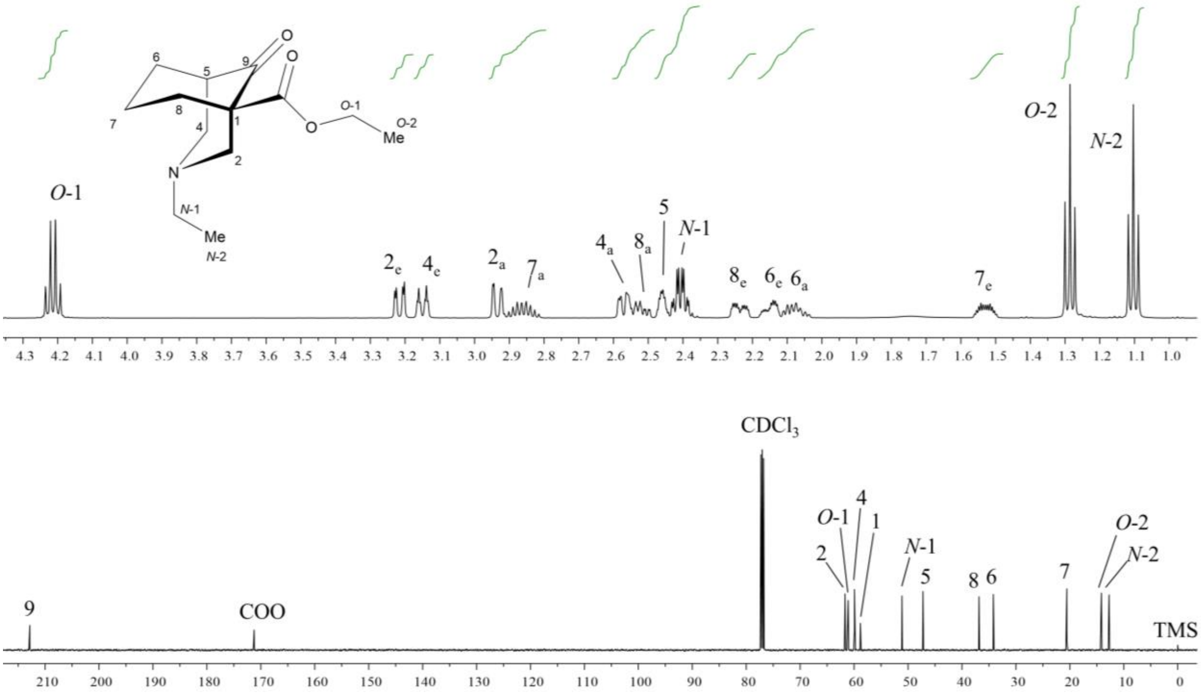

Figure S13. ^{1}H and ^{13}C NMR expansions of [3.3.1]azabicycle (**8**) in $CDCl_3$ with assignments

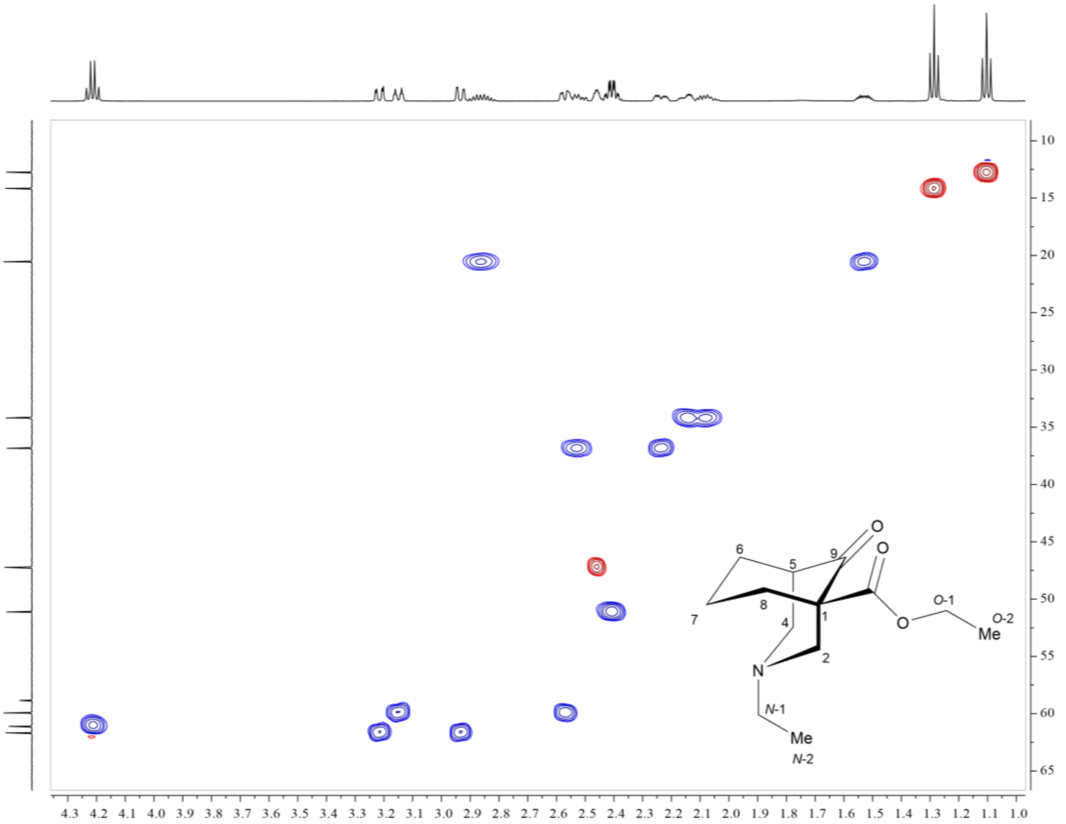

Figure S14. HSQC spectrum of [3.3.1]azabicycle (**8**) in $CDCl_3$

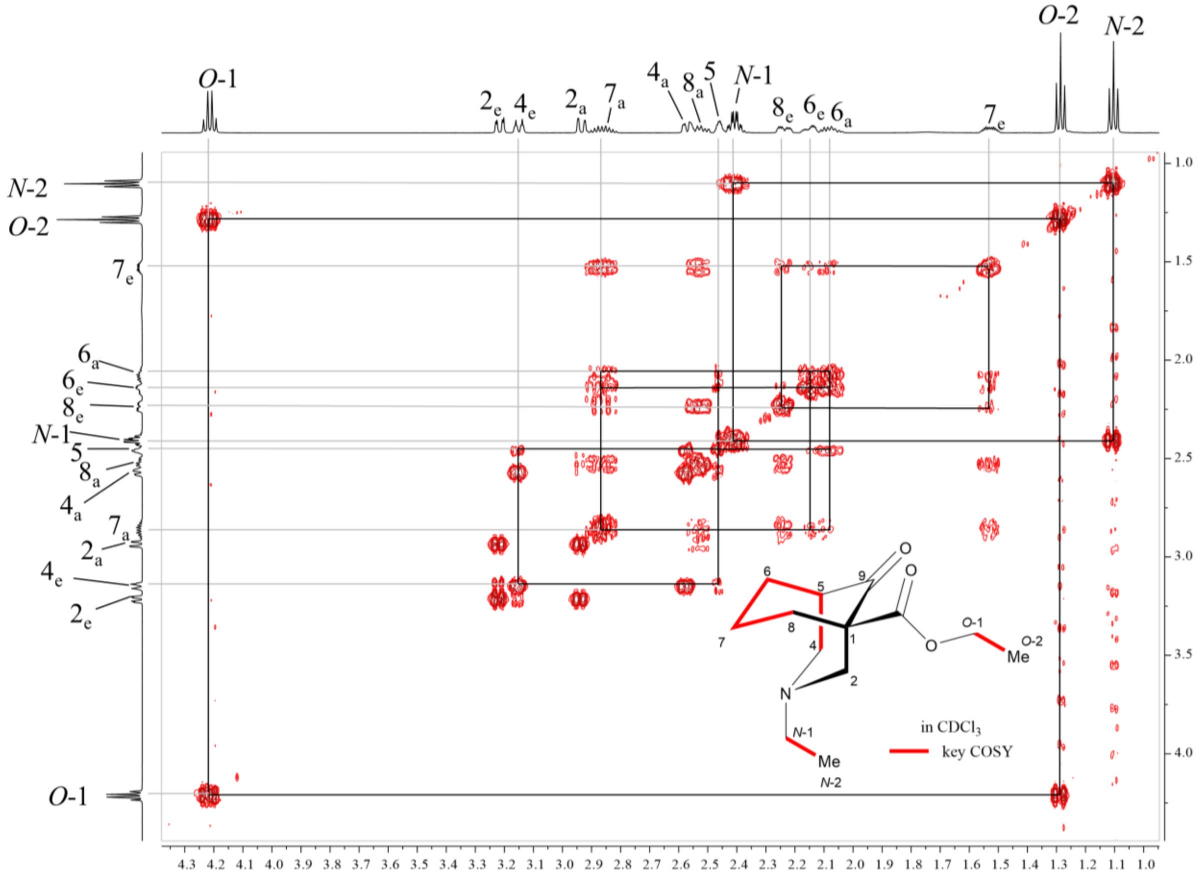

Figure S15. COSY spectrum of [3.3.1]azabicycle (**8**) in $CDCl_3$ with assingments

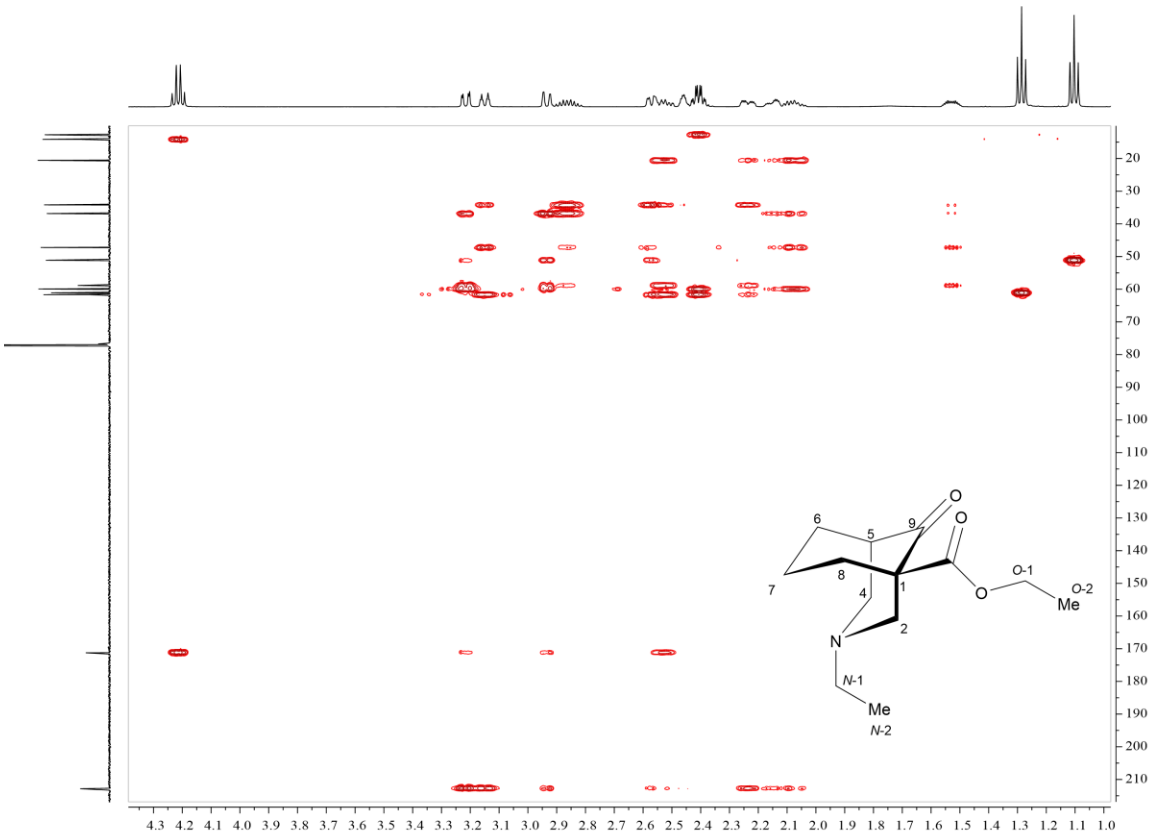

Figure S16. HMBC spectrum of [3.3.1]azabicycle (**8**) in $CDCl_3$

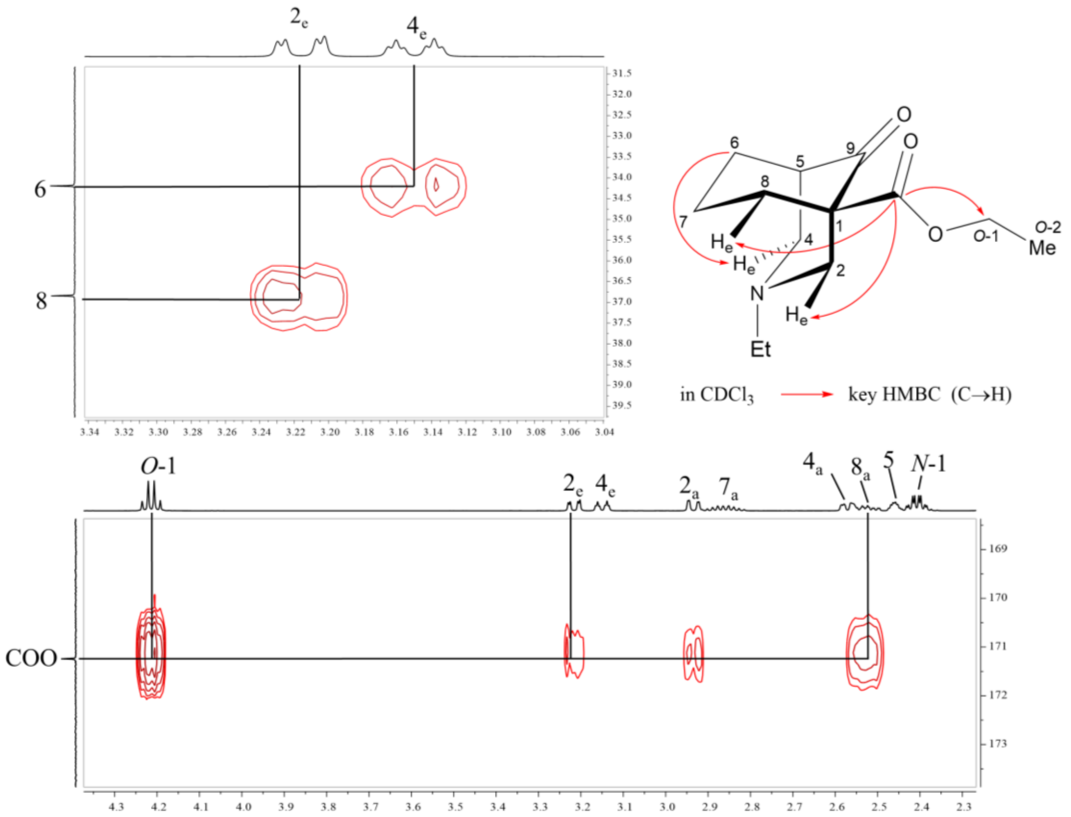

Figure S17. HMBC expansions of [3.3.1]azabicycle (**8**) in $CDCl_3$

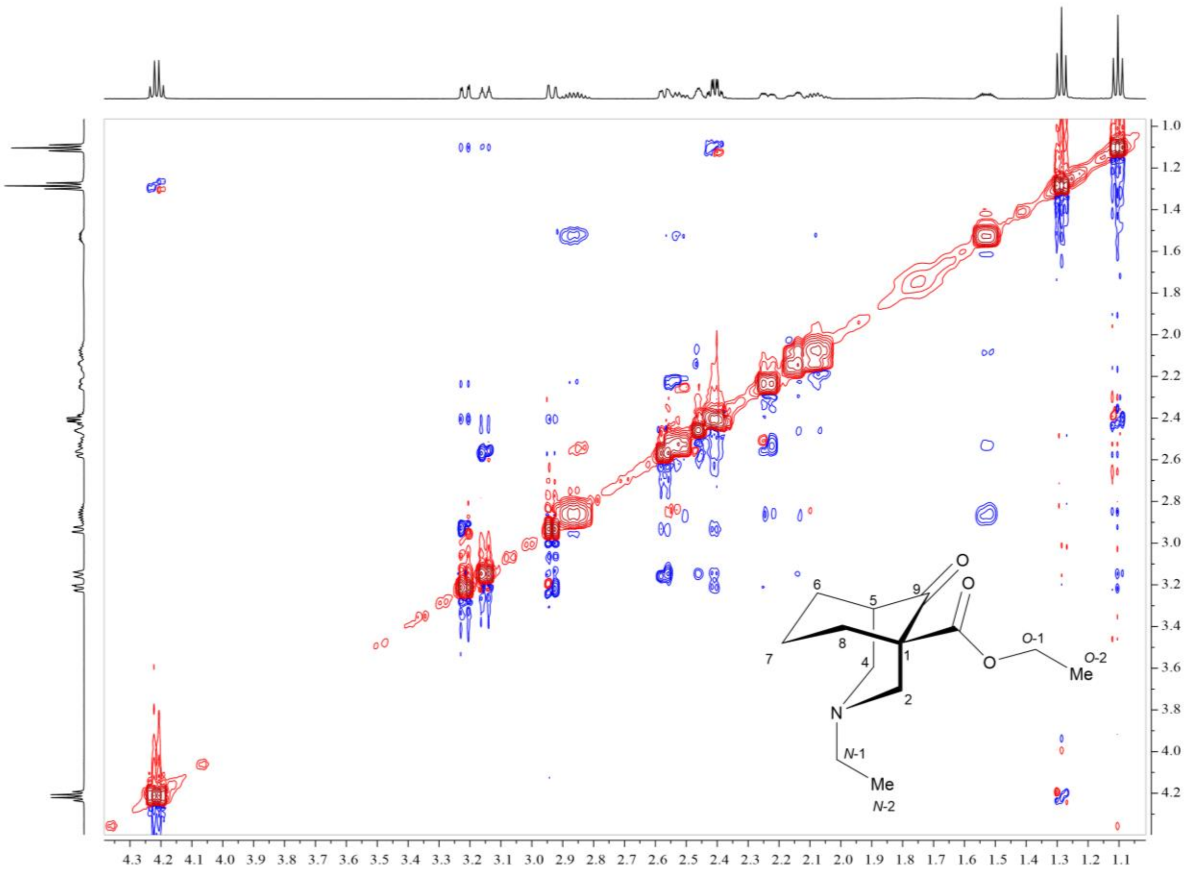

Figure S18. NOESY spectrum of [3.3.1]azabicycle (**8**) in $CDCl_3$

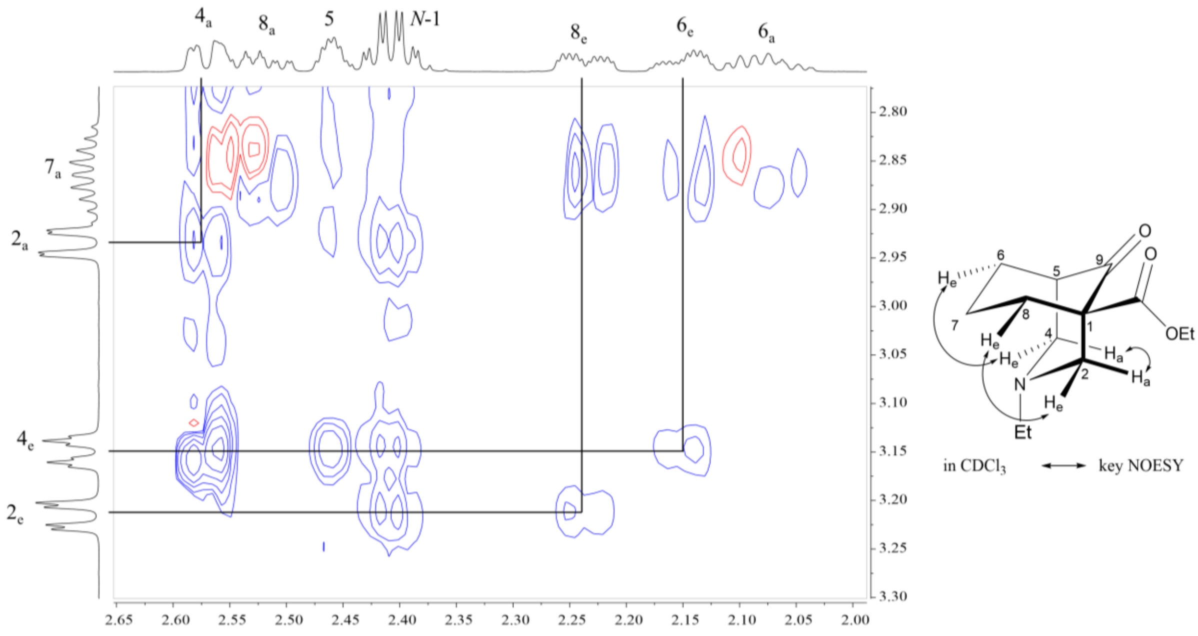

Figure S19. NOESY expasion of [3.3.1]azabicycle (**8**) in $CDCl_3$ with assingments

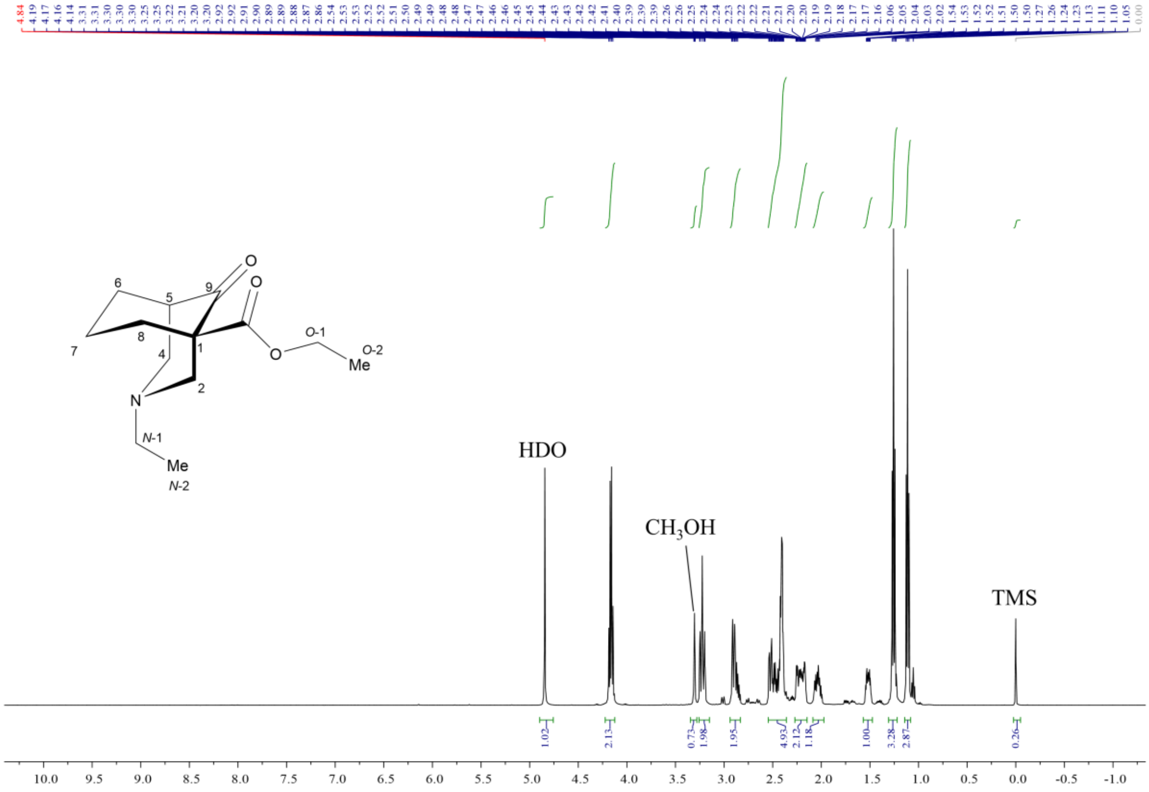

Figure S20. ^{1}H NMR spectrum of [3.3.1]azabicycle (**8**) in CD_3OD

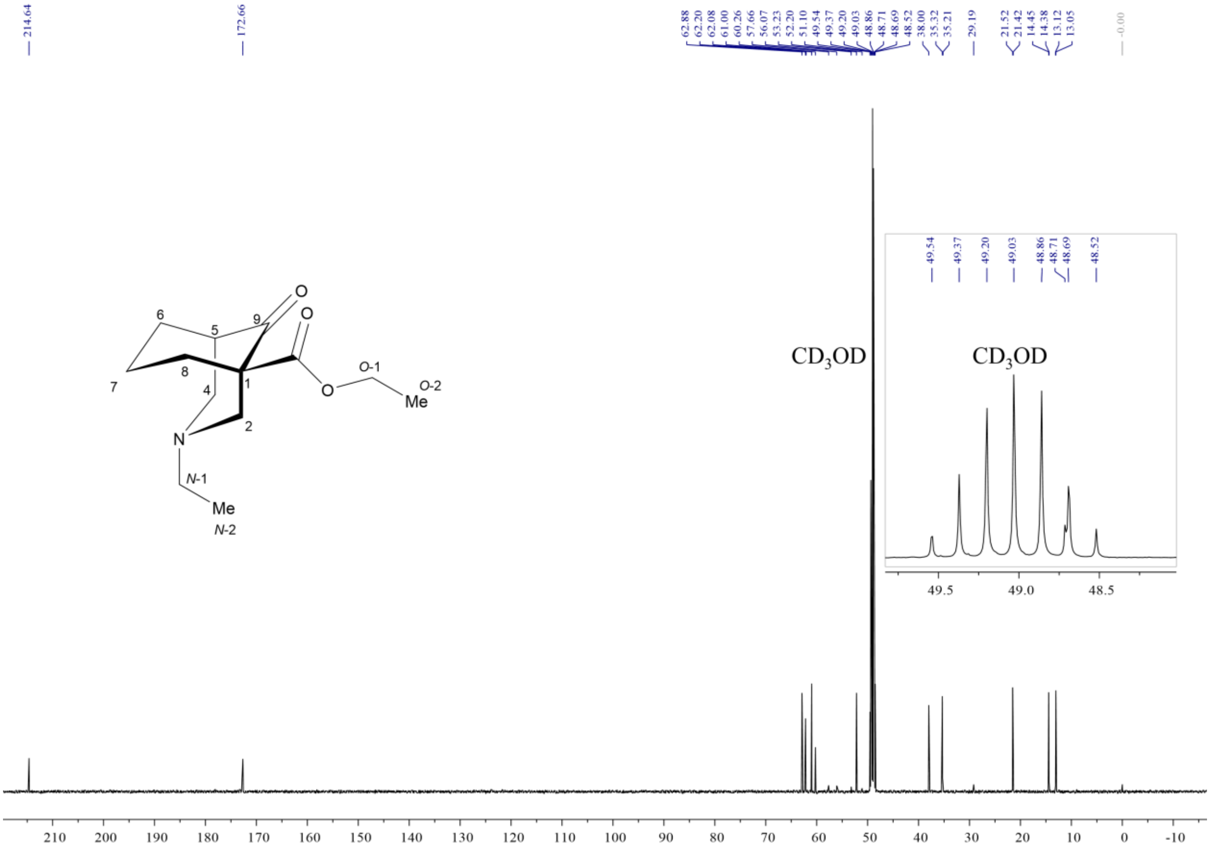

Figure S21. ^{13}C NMR spectrum of [3.3.1]azabicycle (**8**) in CD_3OD

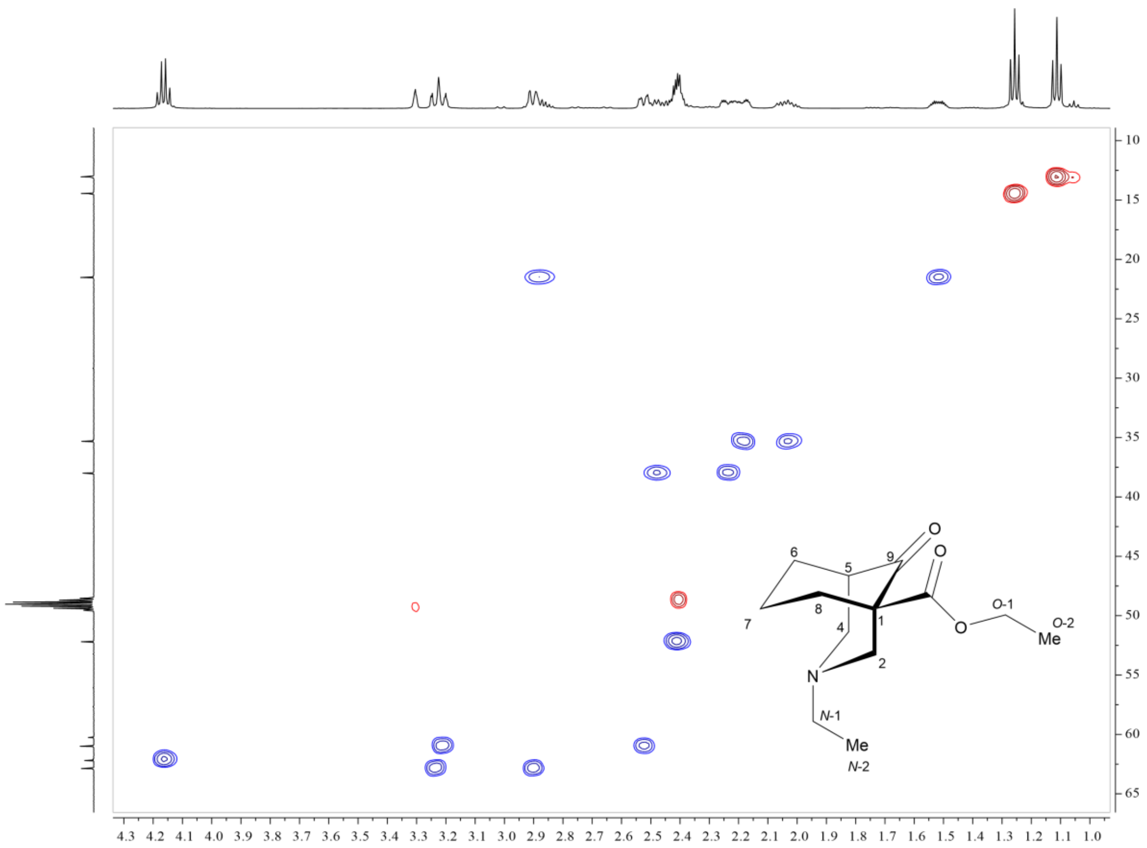

Figure S22. HSQC spectrum of [3.3.1]azabicycle (**8**) in CD_3OD

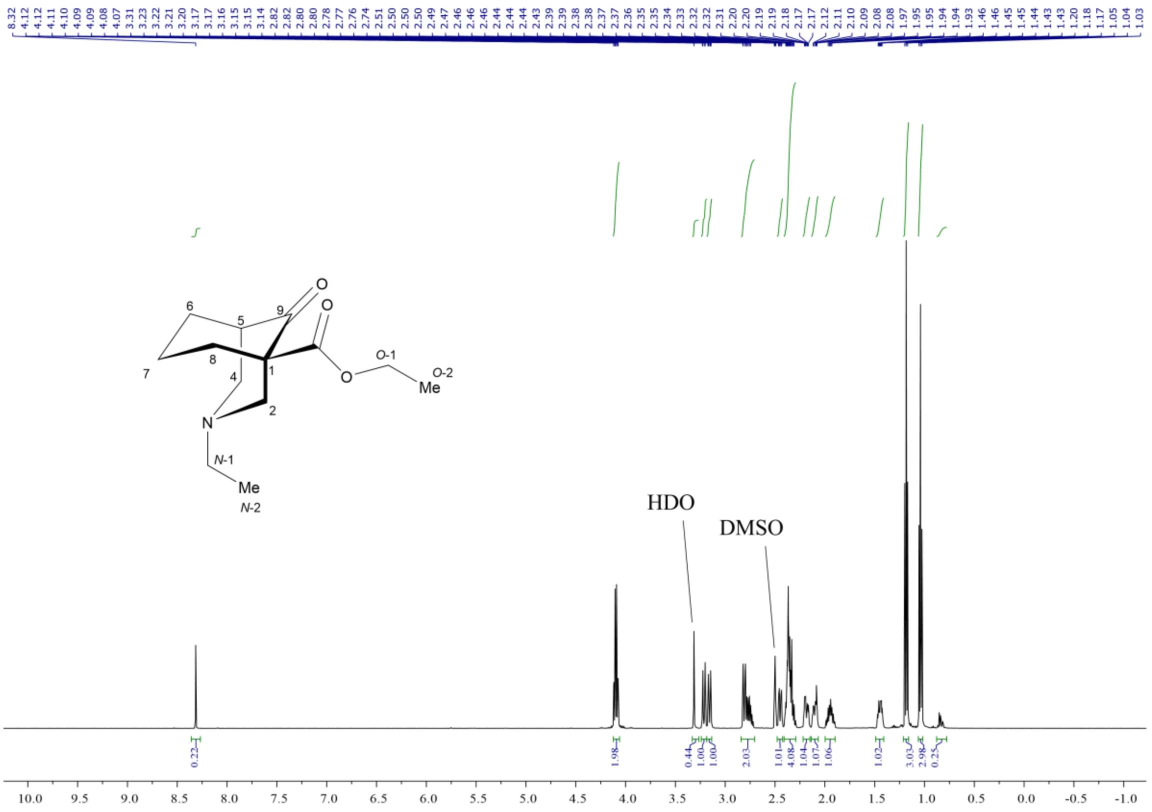

Figure S23. ^{1}H NMR spectrum of [3.3.1]azabicycle (8) in d_6-DMSO

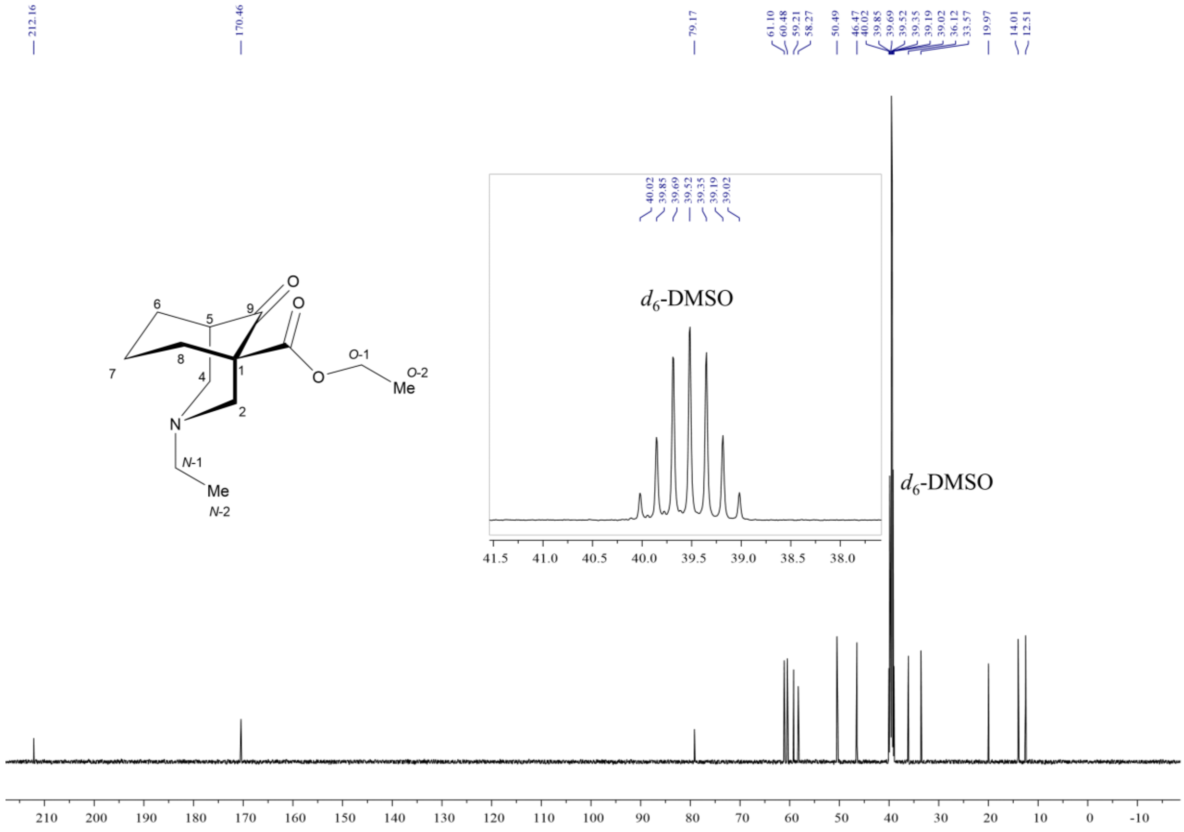

Figure S24. ^{13}C NMR spectrum of [3.3.1]azabicycle (**8**) in d_6-DMSO

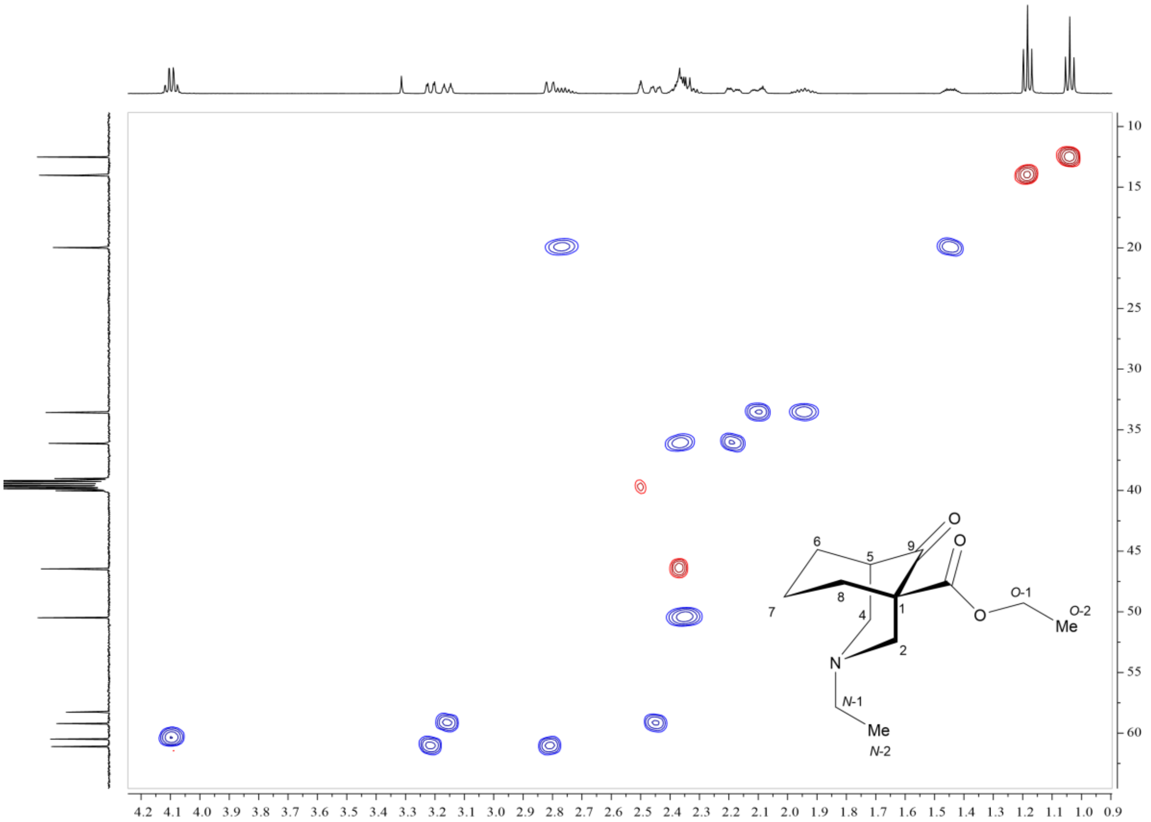

Figure S25. HSQC spectrum of [3.3.1]azabicycle (**8**) in d_6-DMSO

Ethyl 3-ethyl-7,7-dimethyl-9-oxo-3-azabicyclo[3.3.1]nonane-1-carboxylate (**10**)

Confirmation of Expected Formula

Sample-ID	bl_zz_4,4-diMe-4	Submitter	zz615 Ziyu Zeng
Analysis Name	bl_zz_4,4-diMe-4_348263_32_01_53138.d	Supervisor	- Ian Blagbrough
Method used	Confirm Formula Positive 50to500 loop inj.m	Acquisition Date	04/07/2016 14:51:02
Ionisation Mode	positive electrospray (ESI)		

+MS, 1.0-1.3min #(60-77), -Spectral Bkgrnd

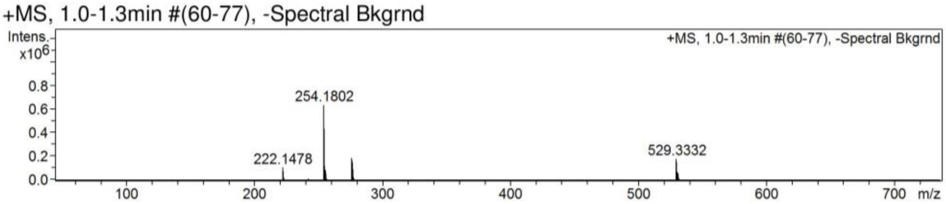

#	m/z	I	I %	Area	S/N
1	222.1478	105860	16.6	4565	5500.2
2	223.1519	13042	2.0	637	658.4
3	254.1802	637041	100.0	17920	18260.0
4	255.1763	85204	13.4	4143	2474.4
5	256.1803	7836	1.2	453	230.6
6	276.1559	188721	29.6	8456	7580.6
7	277.1572	27669	4.3	1471	1132.1
8	529.3332	179292	28.1	14024	8356.0
9	530.3293	60324	9.5	6345	2849.8
10	531.3310	8760	1.4	1043	419.6

Generate Molecular Formula Parameters

Charge	Tolerance	SearchRadius	H/C Ratio min.	H/C Ratio max.	Electron Conf.	Nitrogen Rule	sigma limit
positive	10 ppm	0.05 m/z	0	3	both	true	0.05

Expected Formula C14H23NO3 **Adduct(s):** H, Na

#	meas. m/z	theo. m/z	Err[ppm]	Sigma	Formula
1	276.1559	276.157563	3.90	0.0062	C 14 H 23 N 1 Na 1 O 3

Note: Sigma fits < 0.05 indicates high probability of correct MF, and mass accuracy of 5ppm or better is generally acceptable for publication

Figure S26. MS data of 7,7-dimethyl [3.3.1]azabicycle (**10**)

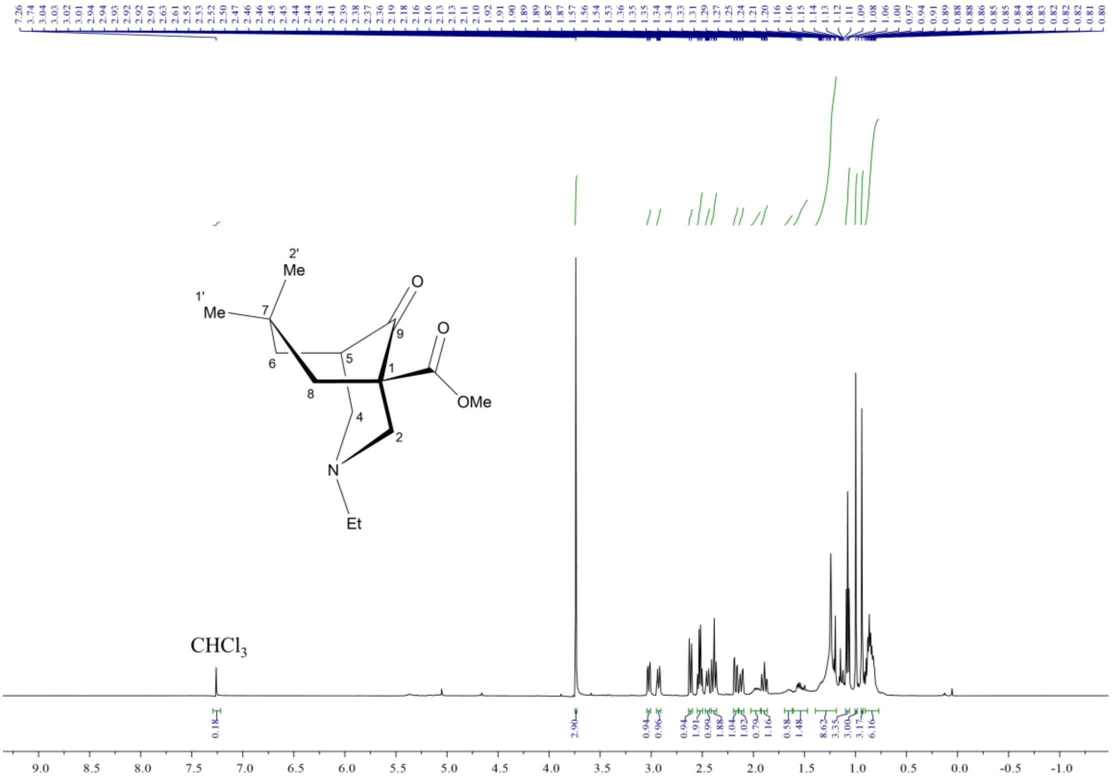

Figure S27. ^{1}H NMR spectrum of 7,7-dimethyl [3.3.1]azabicycle (**10**) in $CDCl_3$

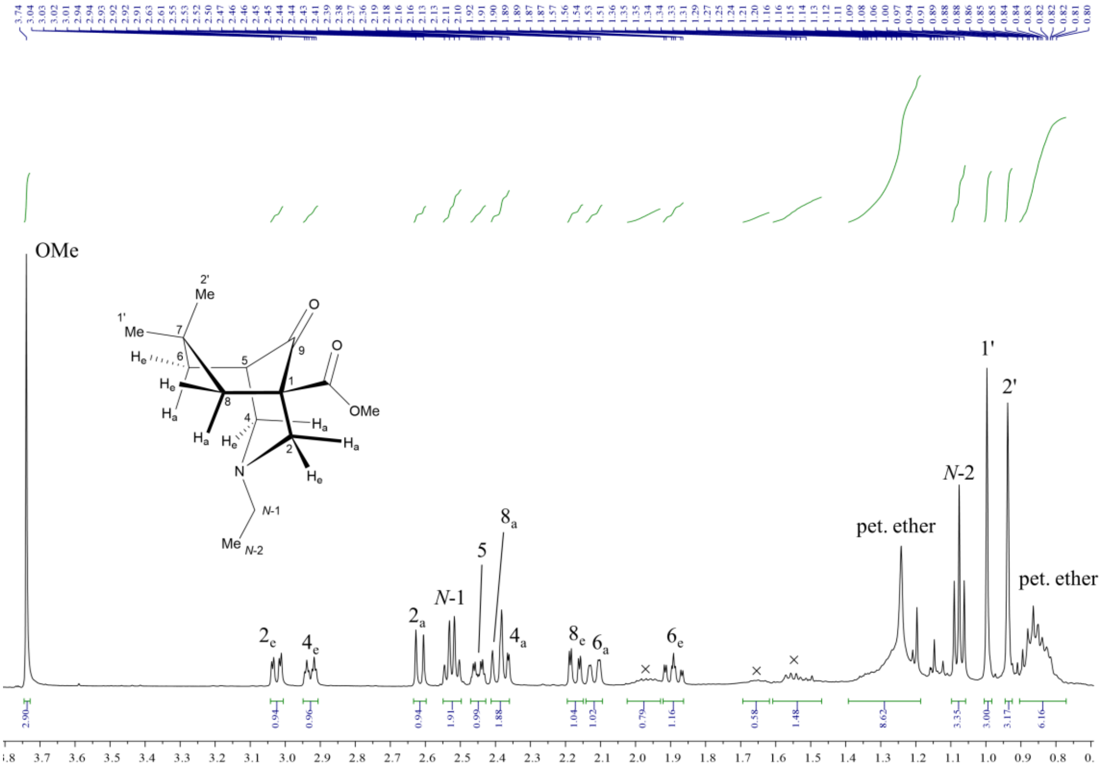

Figure S28. ^{1}H NMR expansion of 7,7-dimethyl [3.3.1]azabicycle (**10**) in $CDCl_3$ with assignments (residual petroleum ether is displayed)[1]

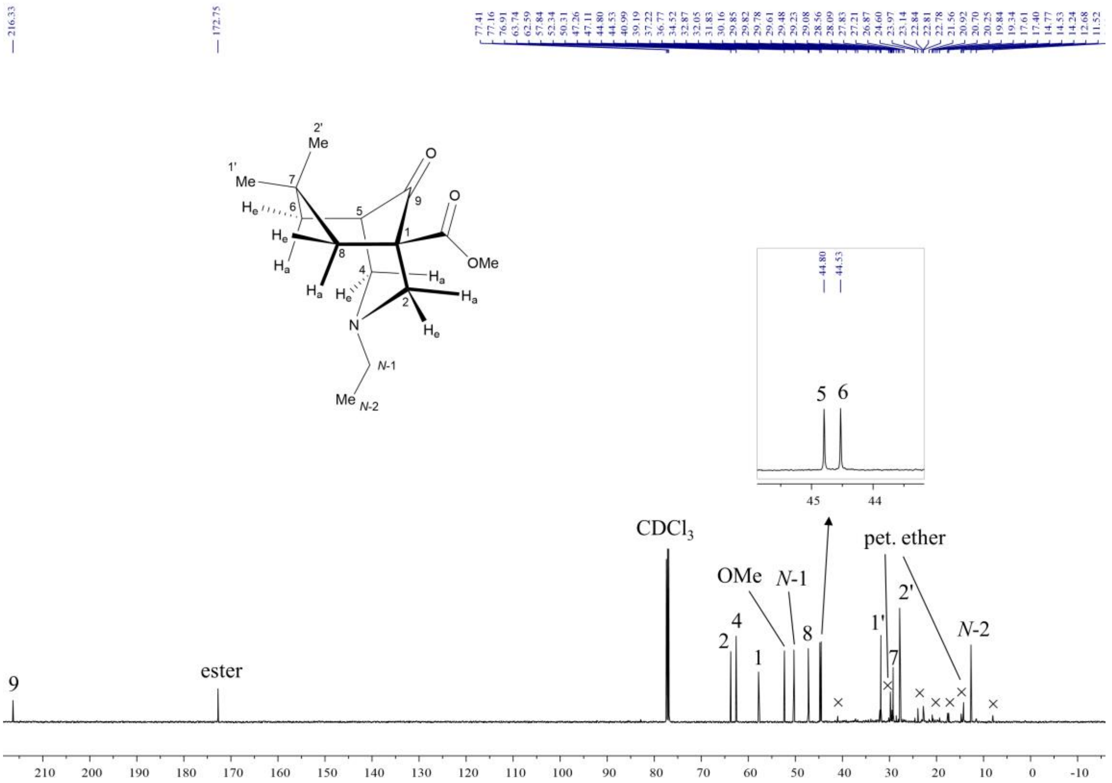

Figure S29. ^{13}C NMR spectrum of 7,7-dimethyl [3.3.1]azabicycle (**10**) in $CDCl_3$ with assignments (residual petroleum ether is displayed)[1]

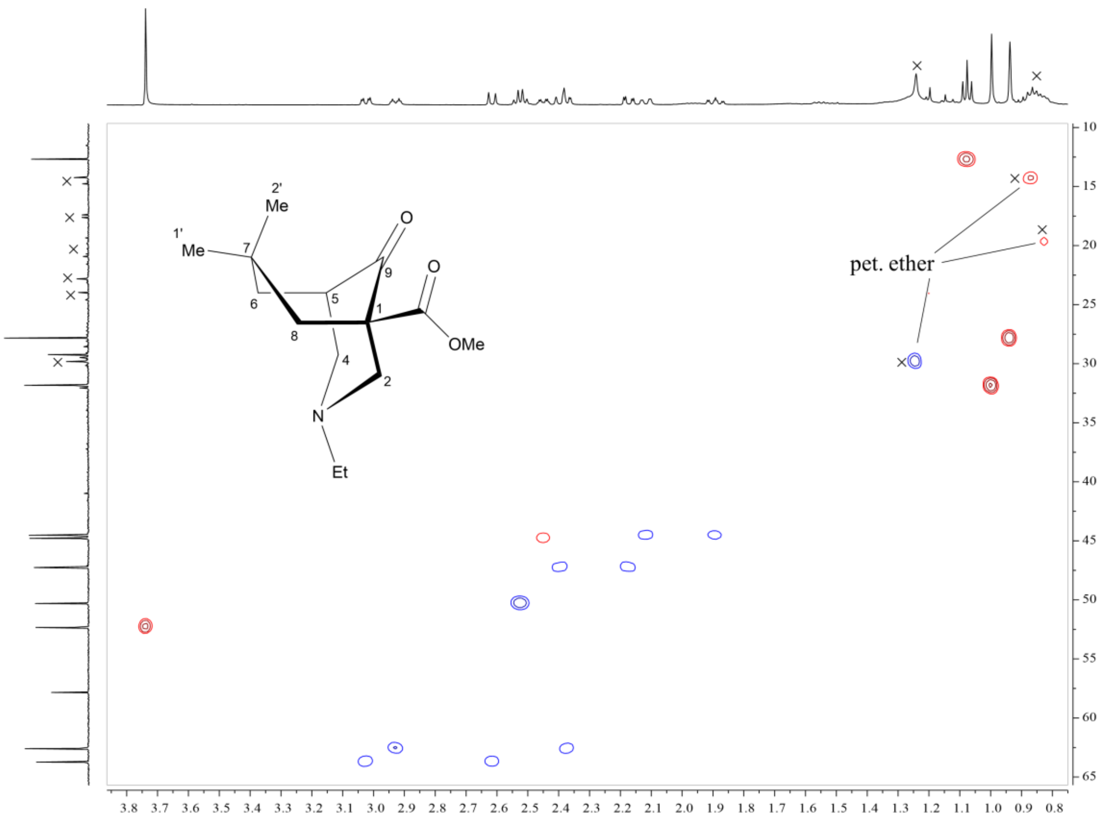

Figure S30. HSQC spectrum of 7,7-dimethyl [3.3.1]azabicycle (**10**) in $CDCl_3$

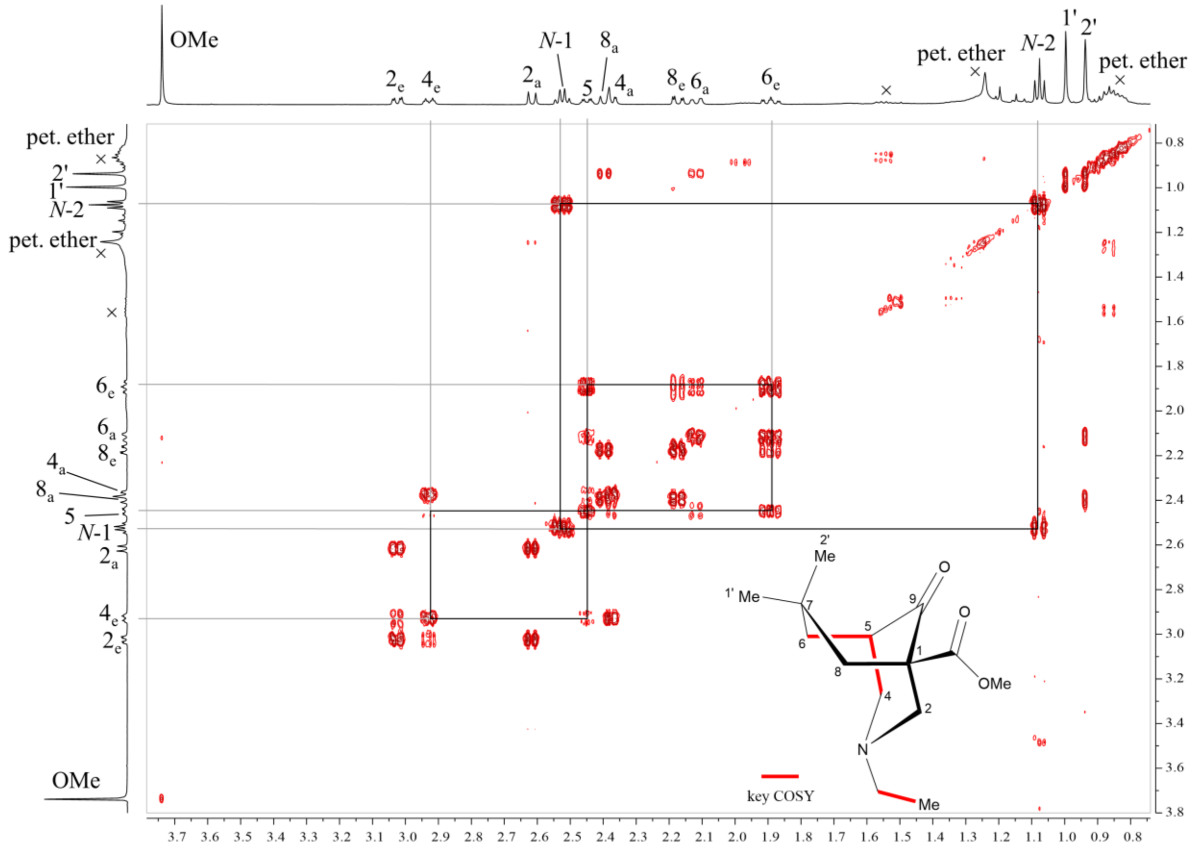

Figure S31. COSY spectrum of 7,7-dimethyl [3.3.1]azabicycle (**10**) in $CDCl_3$

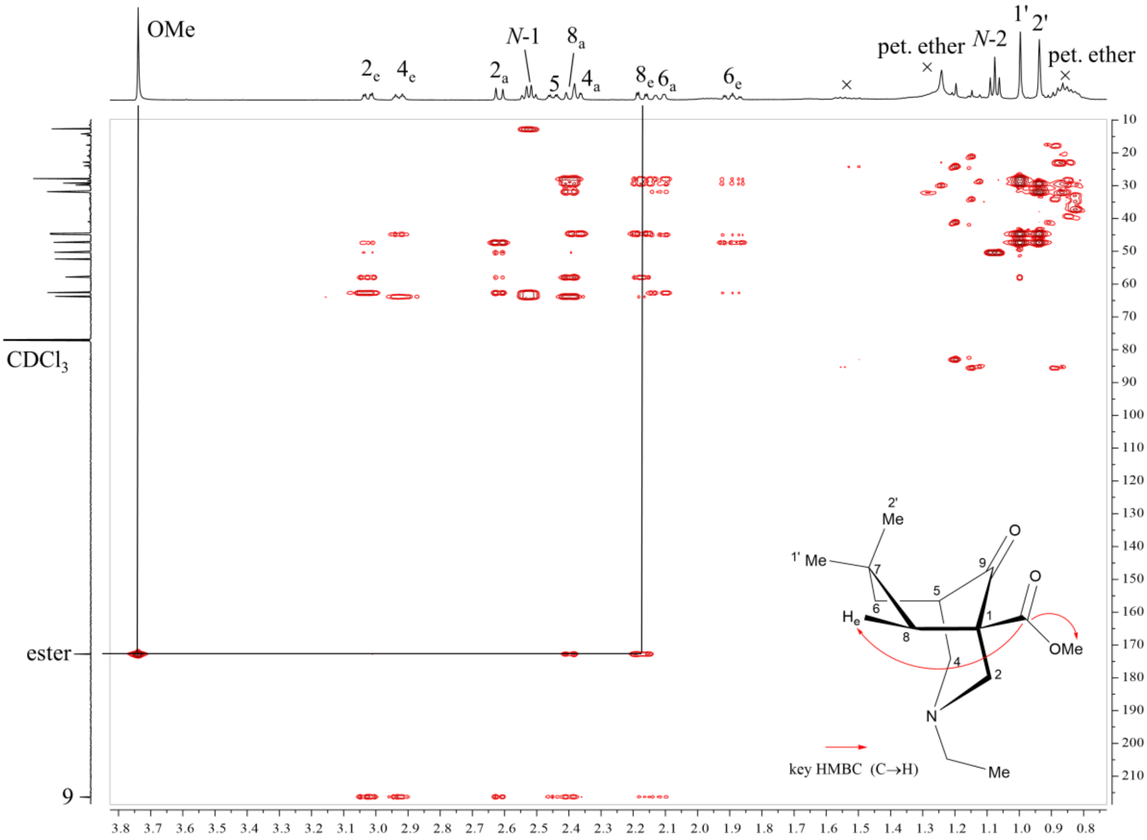

Figure S32. HMBC spectrum of 7,7-dimethyl [3.3.1]azabicycle (**10**) in $CDCl_3$

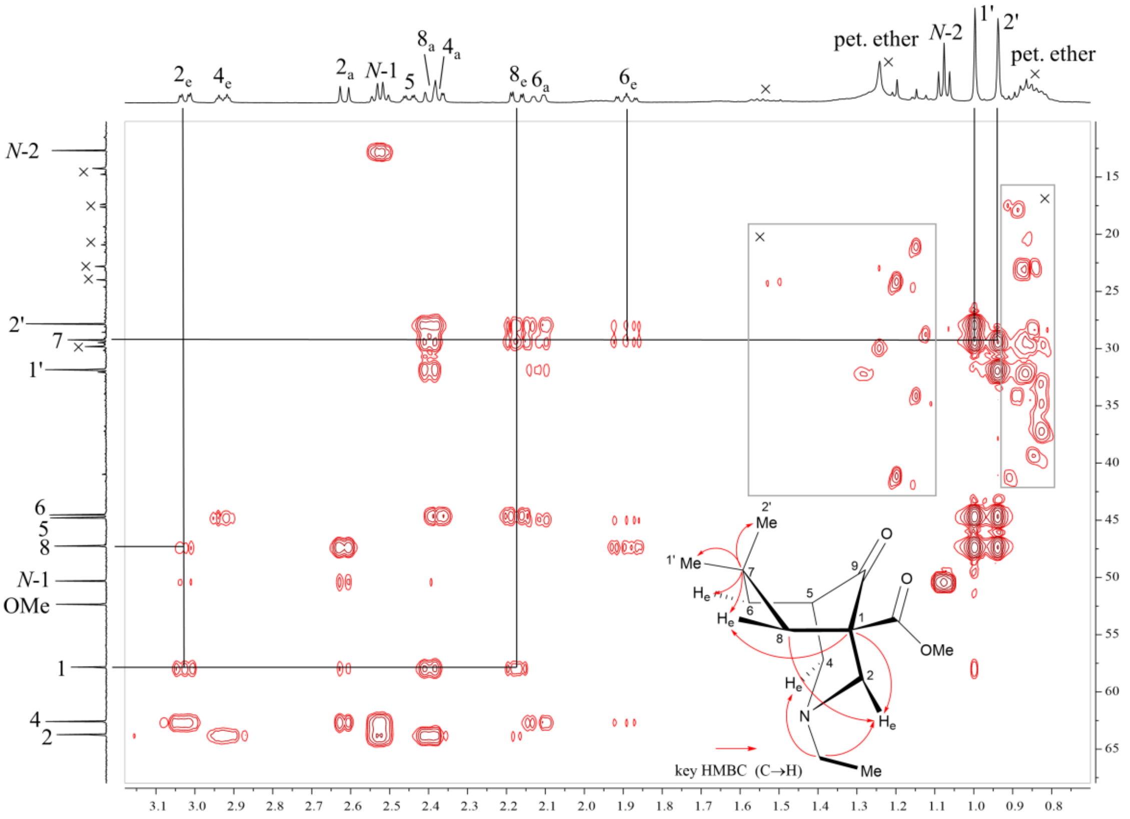

Figure S33. HMBC expansion of 7,7-dimethyl [3.3.1]azabicycle (**10**) in $CDCl_3$

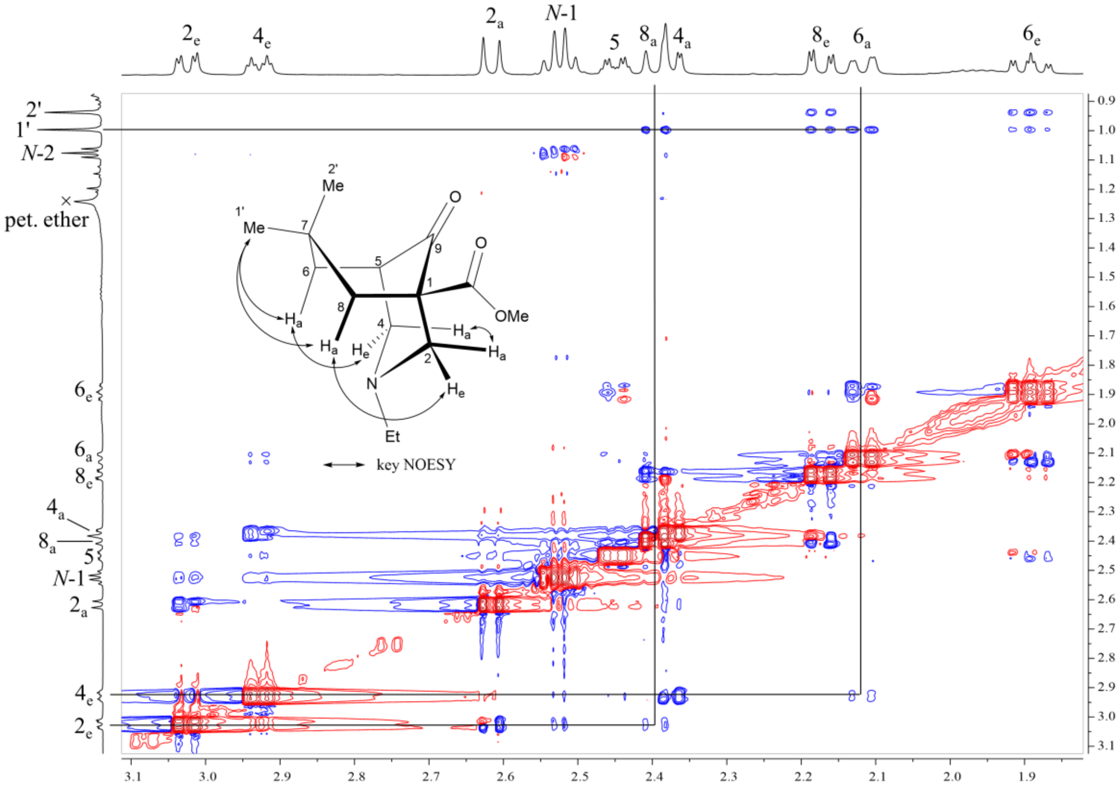

Figure S34. NOESY spectrum of 7,7-dimethyl [3.3.1]azabicycle (**10**) in $CDCl_3$

Methyl 3-ethyl-7-isopropyl-9-oxo-3-azabicyclo[3.3.1]nonane-1-carboxylate (**12**)

Confirmation of Expected Formula

Sample-ID	bl_zz_iPr up	Submitter	zz615 Ziyu Zeng
Analysis Name	bl_zz_iPr up_349401_79_01_54471.d	Supervisor	- Ian Blagbrough
Method used	Confirm Formula Positive 50to500 loop inj.m	Acquisition Date	12/10/2016 12:43:17
Ionisation Mode	positive electrospray (ESI)		

+MS, 1.0-1.3min #(60-77), -Spectral Bkgrnd

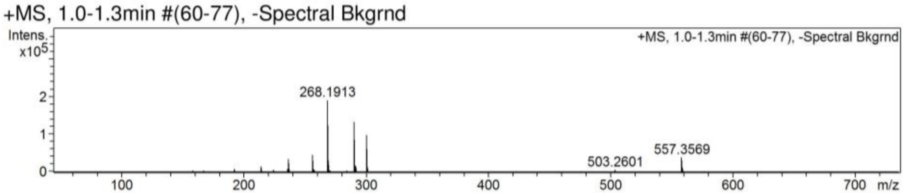

#	m/z	I	I %	Area	S/N
1	236.1661	32742	17.2	1603	1724.1
2	256.1896	43746	23.0	2007	1668.5
3	268.1913	190471	100.0	7372	7104.6
4	269.1923	29304	15.4	1385	1091.0
5	290.1723	131540	69.1	5570	4716.2
6	291.1759	14927	7.8	890	534.2
7	300.2155	96915	50.9	4686	3578.5
8	301.2188	13460	7.1	790	506.9
9	557.3569	38791	20.4	4344	3942.9
10	558.3612	11500	6.0	1278	1155.5

Generate Molecular Formula Parameters

Charge	Tolerance	SearchRadius	H/C Ratio min.	H/C Ratio max.	Electron Conf.	Nitrogen Rule	sigma limit
positive	10 ppm	0.05 m/z	0	3	both	true	0.05

Expected Formula C15H25NO3 **Adduct(s):** H, Na

#	meas. m/z	theo. m/z	Err[ppm]	Sigma	Formula
1	268.1913	268.191269	-2.00	0.0078	C 15 H 26 N 1 O 3
1	290.1723	290.173213	1.30	0.0307	C 15 H 25 N 1 Na 1 O 3

Note: Sigma fits < 0.05 indicates high probability of correct MF, and mass accuracy of 5ppm or better is generally acceptable for publication

Figure S35. MS data of 7-iPr [3.3.1]azabicycle (**12**)

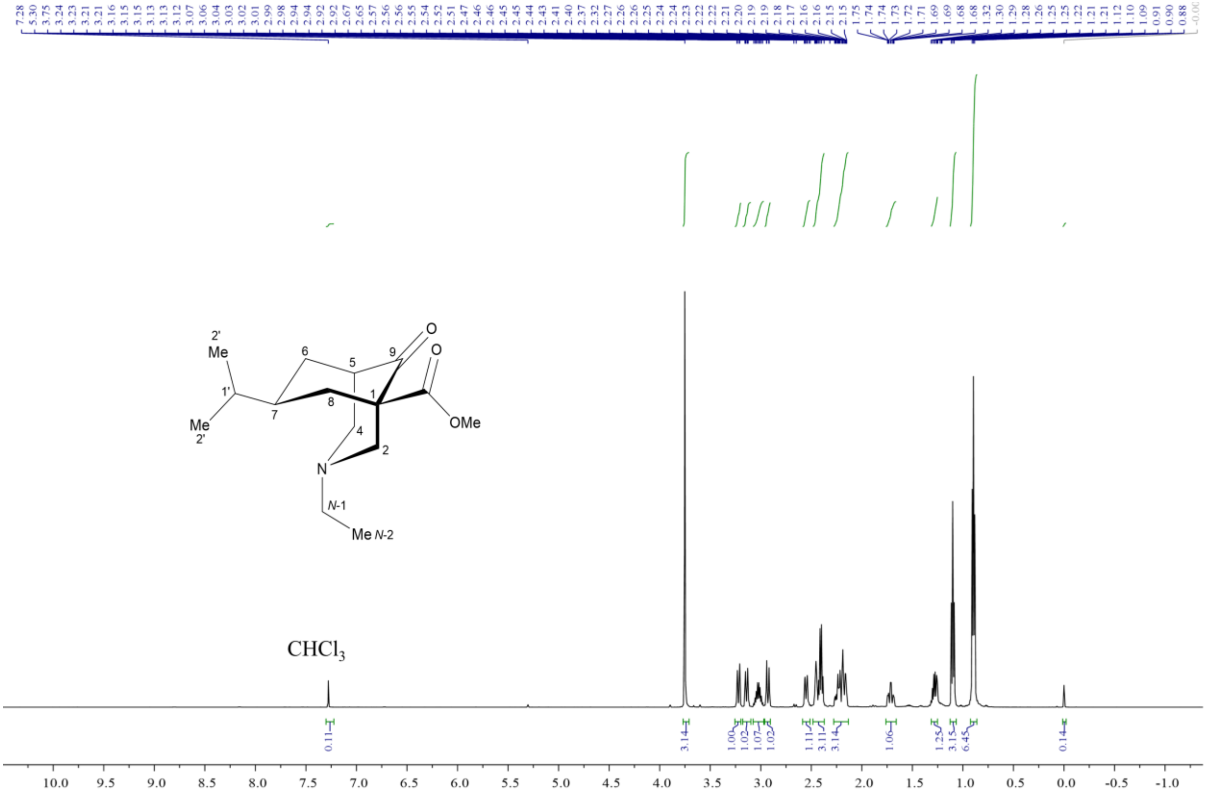

Figure S36. ^{1}H NMR spectrum of 7-iPr [3.3.1]azabicycle (**12**) in $CDCl_3$

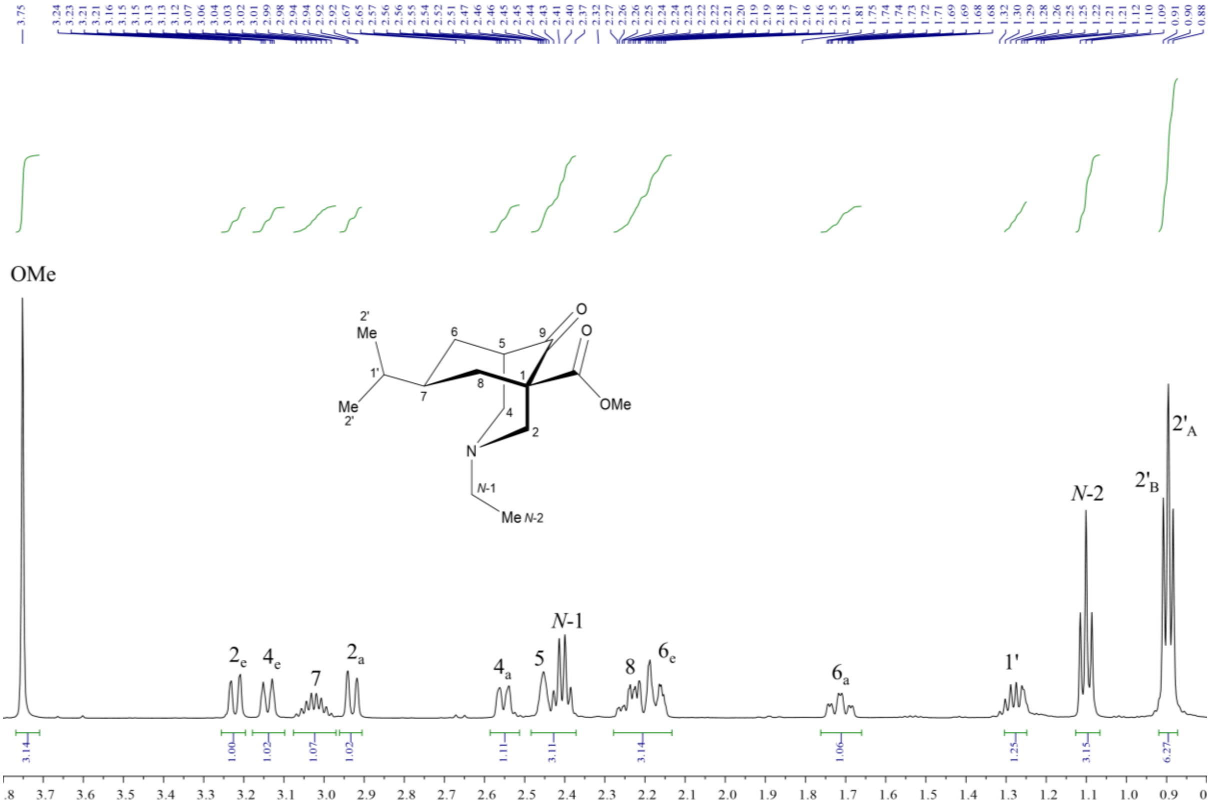

Figure S37. ^{1}H NMR expansion of 7-iPr [3.3.1]azabicycle (**12**) in $CDCl_3$ with assignments

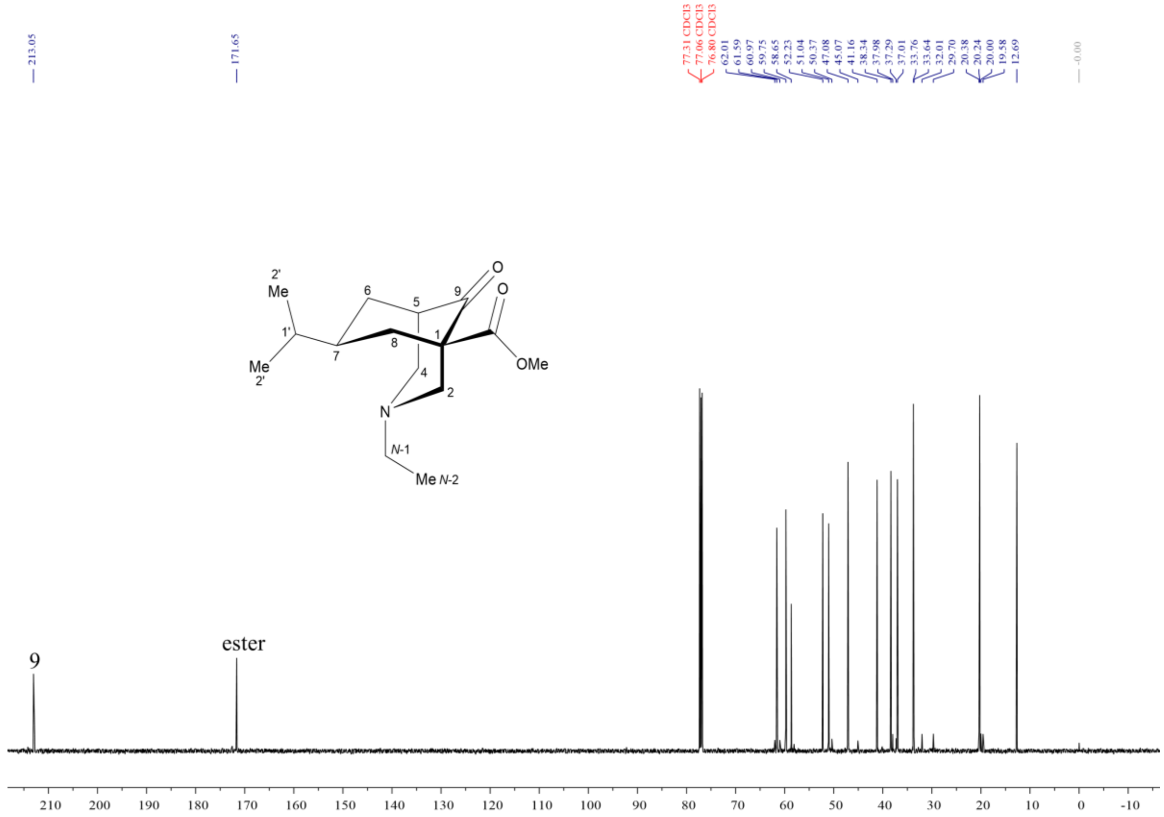

Figure S38. ^{13}C NMR spectrum of 7-iPr [3.3.1]azabicycle (**12**) in $CDCl_3$

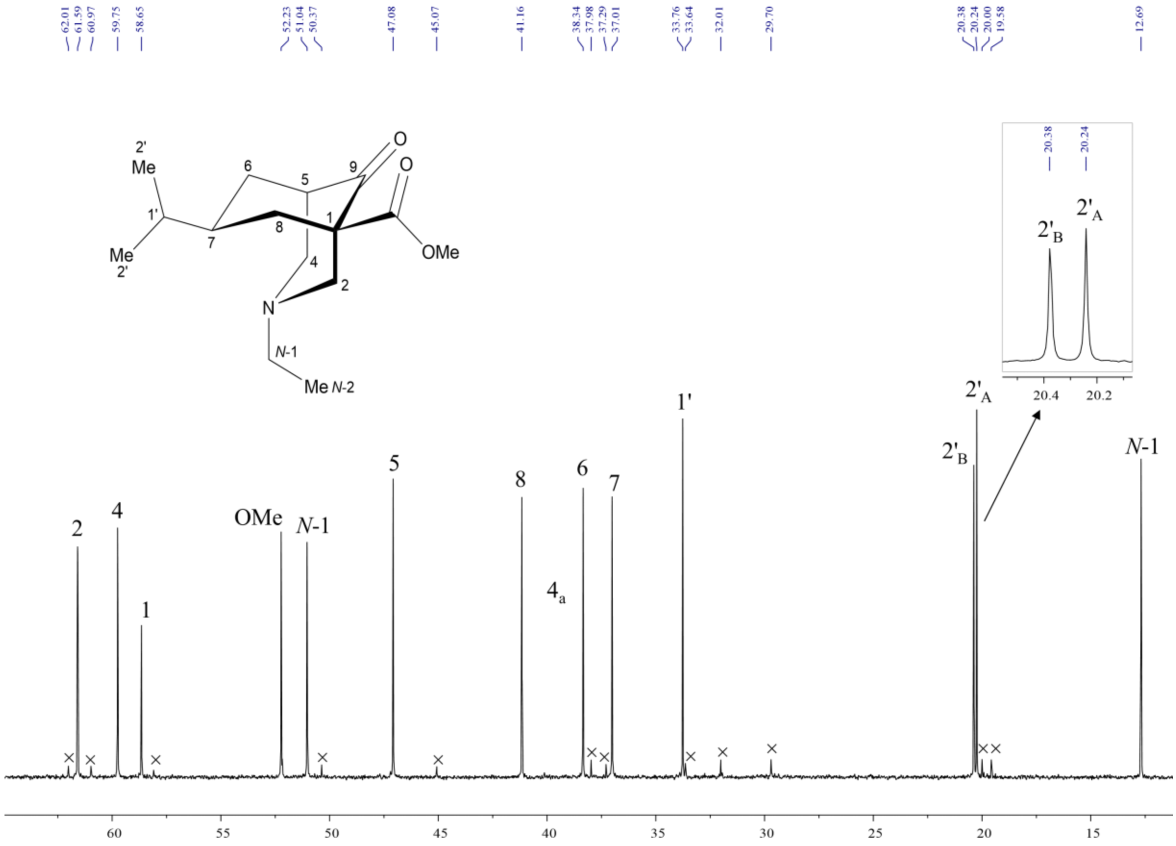

Figure S39. ^{13}C NMR expansion of 7-iPr [3.3.1]azabicycle (**12**) in $CDCl_3$ with assignments

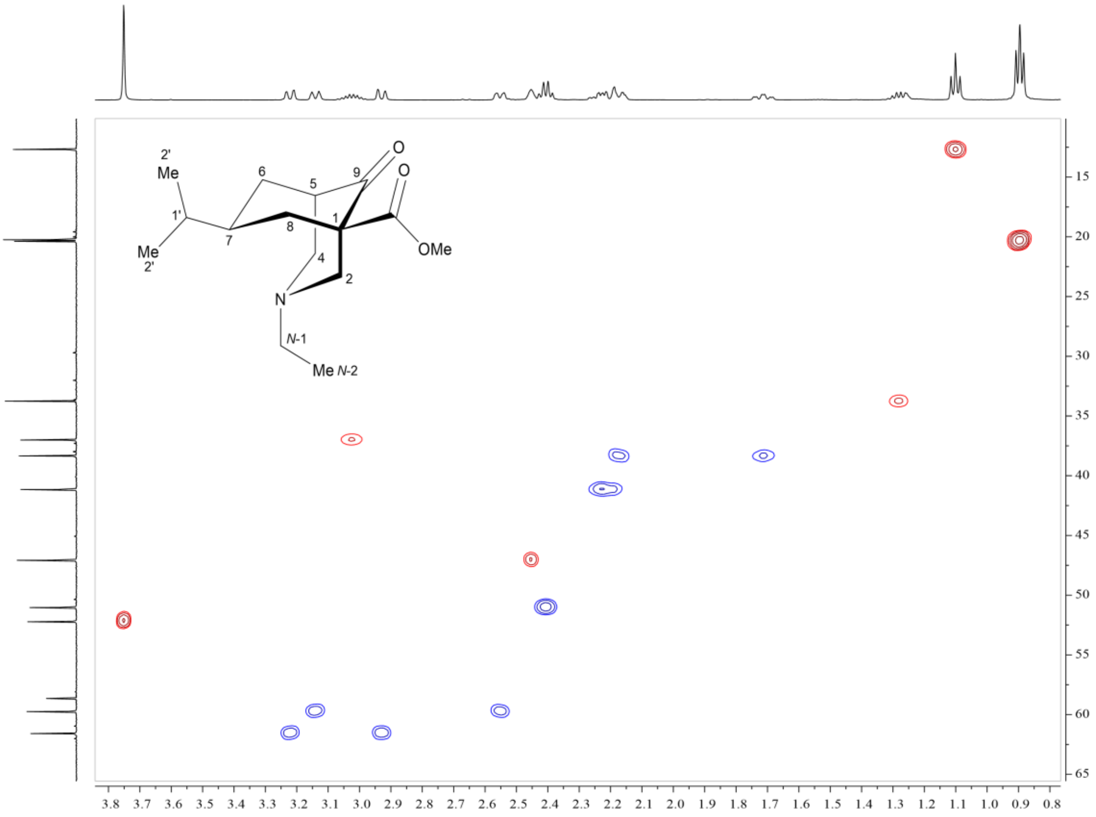

Figure S40. HSQC spectrum of 7-iPr [3.3.1]azabicycle (**12**) in $CDCl_3$

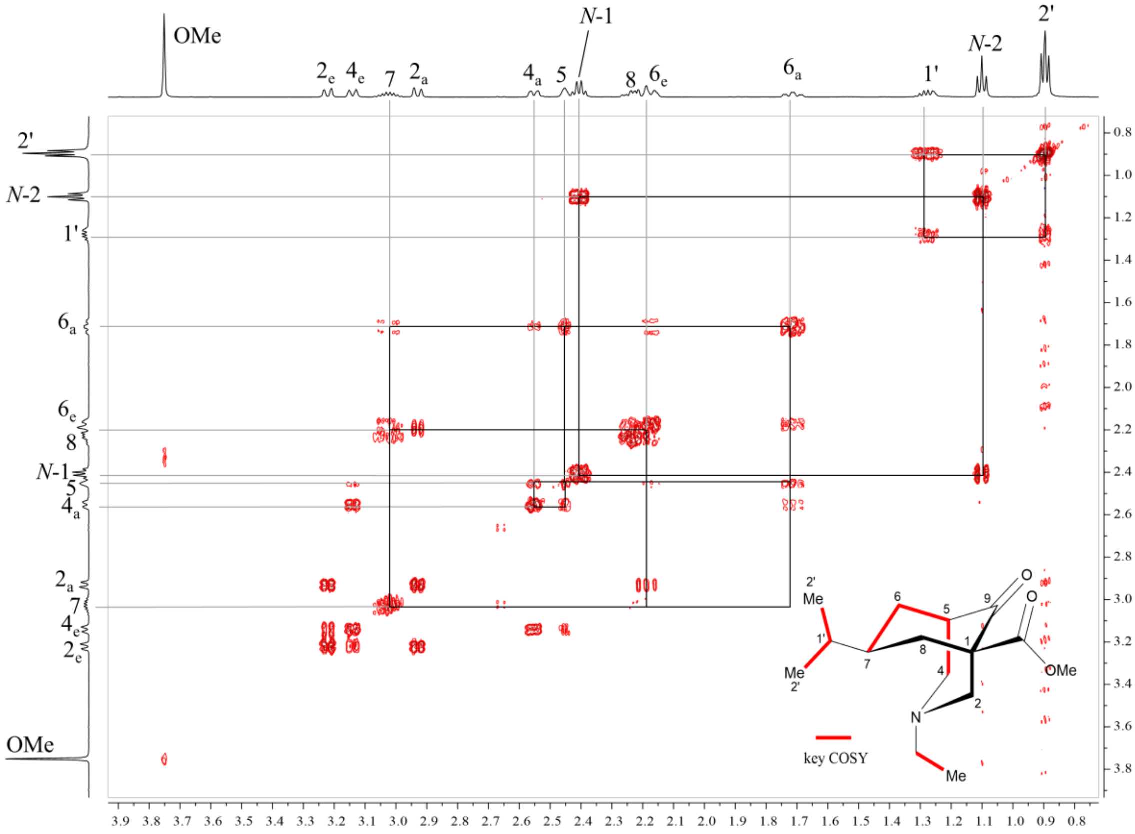

Figure S41. COSY spectrum of 7-iPr [3.3.1]azabicycle (**12**) in $CDCl_3$

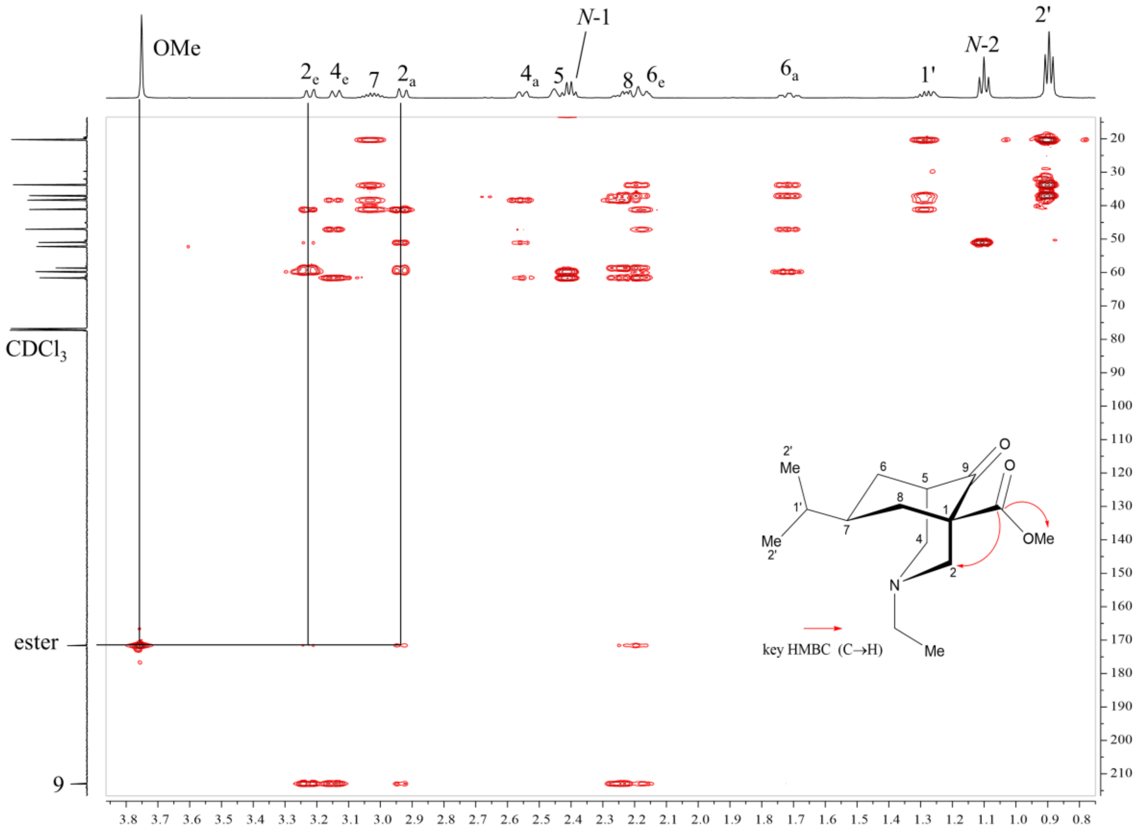

Figure S42. HMBC spectrum of 7-iPr [3.3.1]azabicycle (**12**) in $CDCl_3$

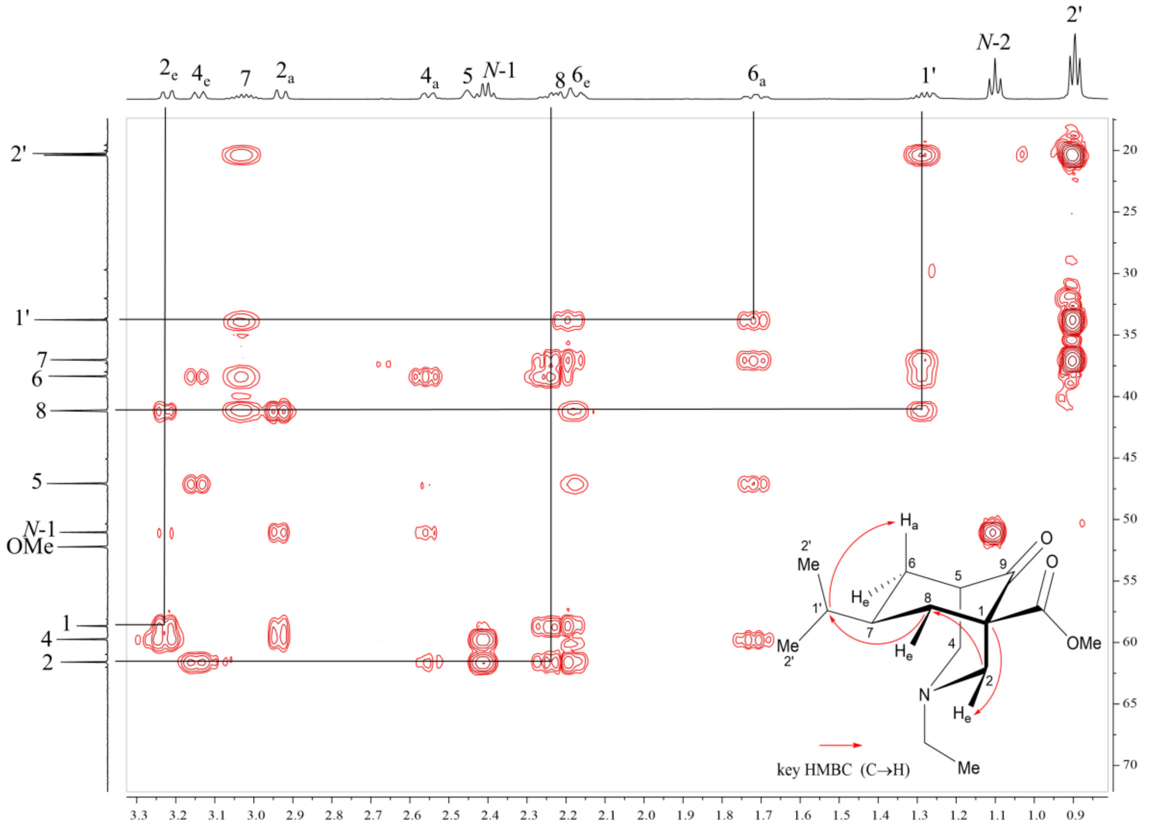

Figure S43. HMBC expansion of 7-iPr [3.3.1]azabicycle (**12**) in $CDCl_3$

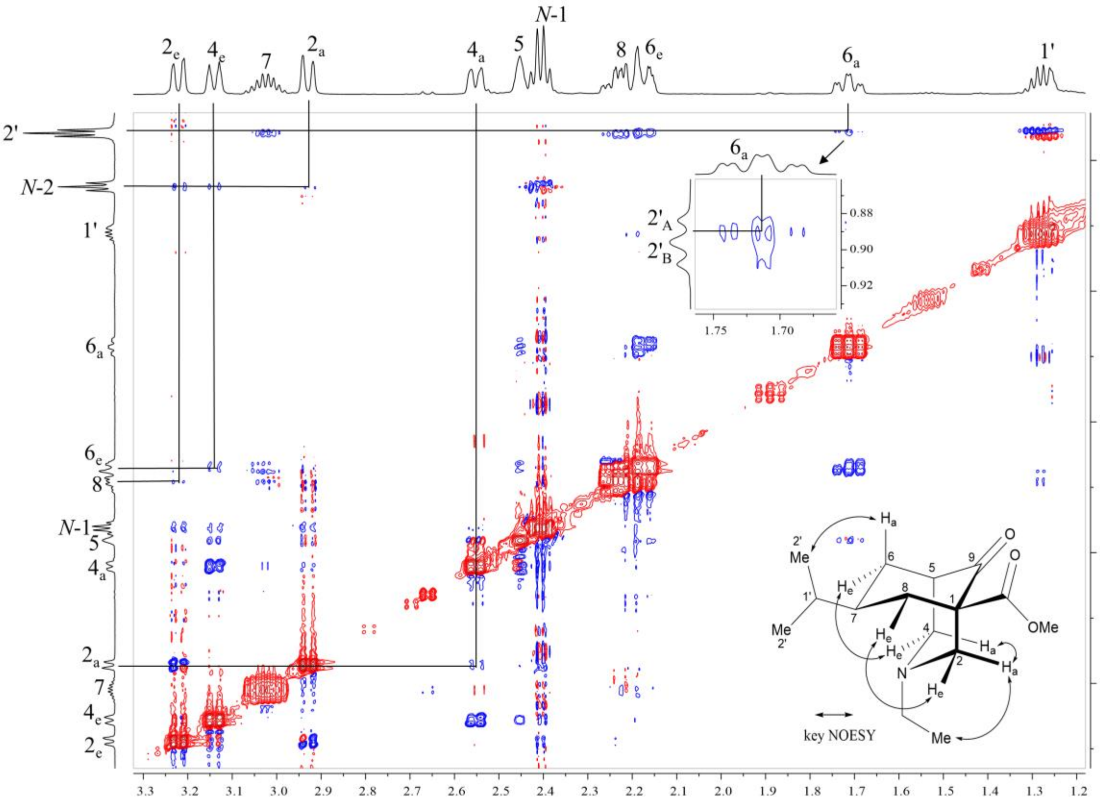

Figure S44. NOESY spectrum of 7-iPr [3.3.1]azabicycle (**12**) in $CDCl_3$

Ethyl 3-ethyl-7-methyl-9-oxo-3-azabicyclo[3.3.1]nonane-1-carboxylate (**14**)

Confirmation of Expected Formula

Sample-ID	bl_zz_7-Me all	Submitter	zz615 Ziyu Zeng
Analysis Name	bl_zz_7-Me all_350133_20_01_55334.d	Supervisor	- Ian Blagbrough
Method used	Confirm Formula Positive 50to500 loop inj.m	Acquisition Date	01/12/2016 15:04:44
Ionisation Mode	positive electrospray (ESI)		

+MS, 1.0-1.3min #(60-77), -Spectral Bkgrnd

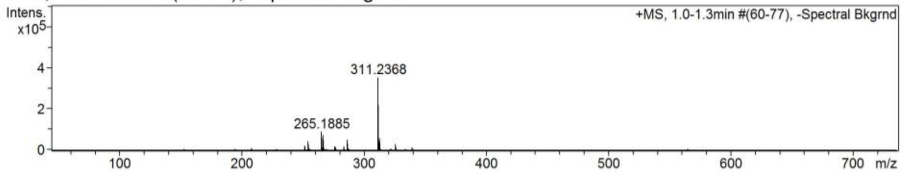

#	m/z	I	I %	Area	S/N
1	251.2109	23056	6.6	1147	605.8
2	254.1752	40846	11.6	1637	1009.9
3	265.1885	89860	25.6	4011	1814.4
4	266.1769	74369	21.2	3782	1477.3
5	276.1573	17452	5.0	757	298.0
6	283.2353	19368	5.5	1054	300.7
7	286.2001	50256	14.3	2354	751.8
8	311.2368	351593	100.0	13234	5579.3
9	312.2357	57036	16.2	3224	919.9
10	325.2215	29805	8.5	2344	610.9

Generate Molecular Formula Parameters

Charge	Tolerance	SearchRadius	H/C Ratio min.	H/C Ratio max.	Electron Conf.	Nitrogen Rule	sigma limit
positive	10 ppm	0.05 m/z	0	3	both	true	0.05

Expected Formula C14H23NO3 **Adduct(s):** H, Na

#	meas. m/z	theo. m/z	Err[ppm]	Sigma	Formula
1	254.1752	254.175619	-0.50	0.0234	C 14 H 24 N 1 O 3
1	276.1573	276.157563	-1.00	0.0390	C 14 H 23 N 1 Na 1 O 3

Note: Sigma fits < 0.05 indicates high probability of correct MF, and mass accuracy of 5ppm or better is generally acceptable for publication

Figure S45. MS data of 7-Me [3.3.1]azabicycle (**14**, ethyl ester)

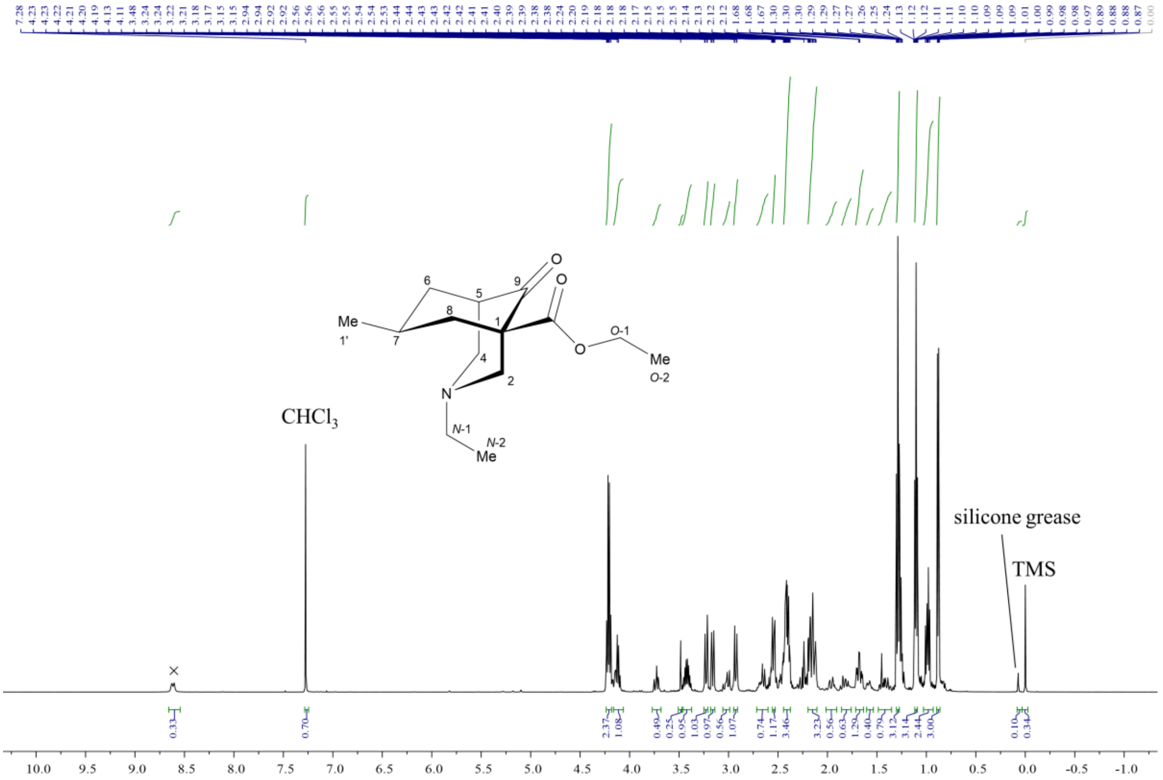

Figure S46. ^{1}H NMR spectrum of 7-Me [3.3.1]azabicycle (**14**, ethyl ester) in $CDCl_3$ (residual silicone grease is displayed)[1]

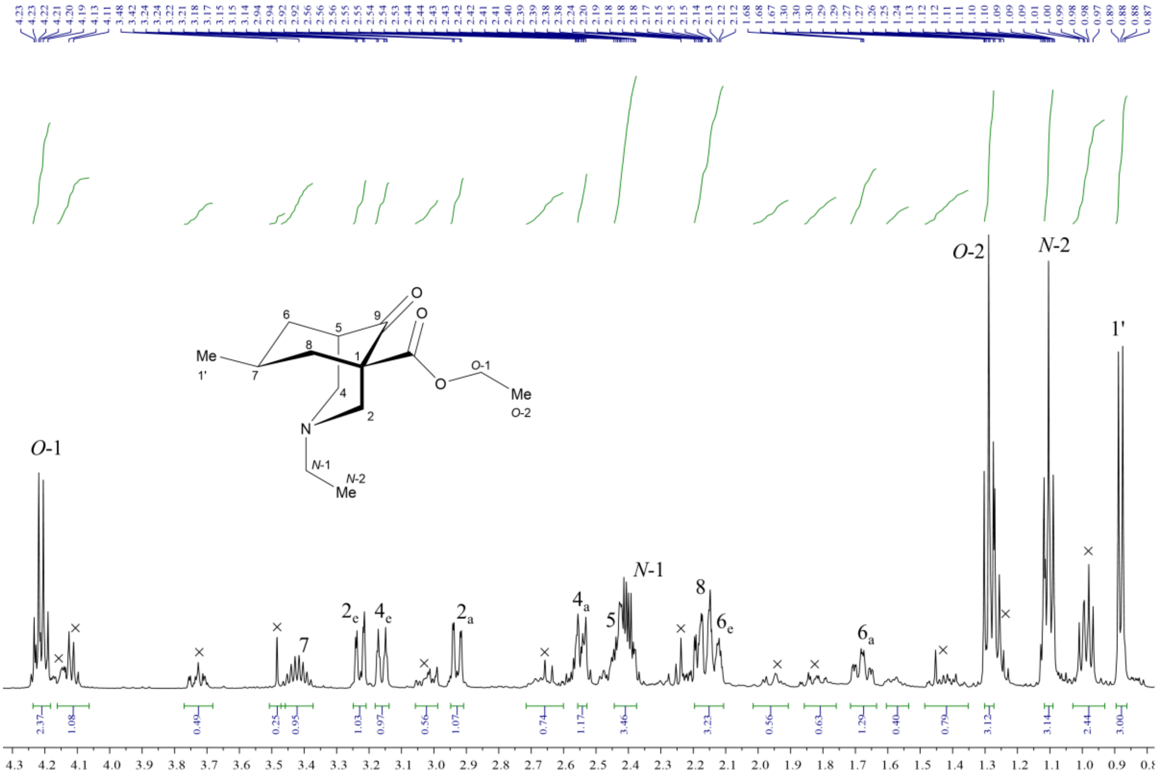

Figure S47. ^{1}H NMR expansion of 7-Me [3.3.1]azabicycle (**14**, ethyl ester) in $CDCl_3$ with assignments

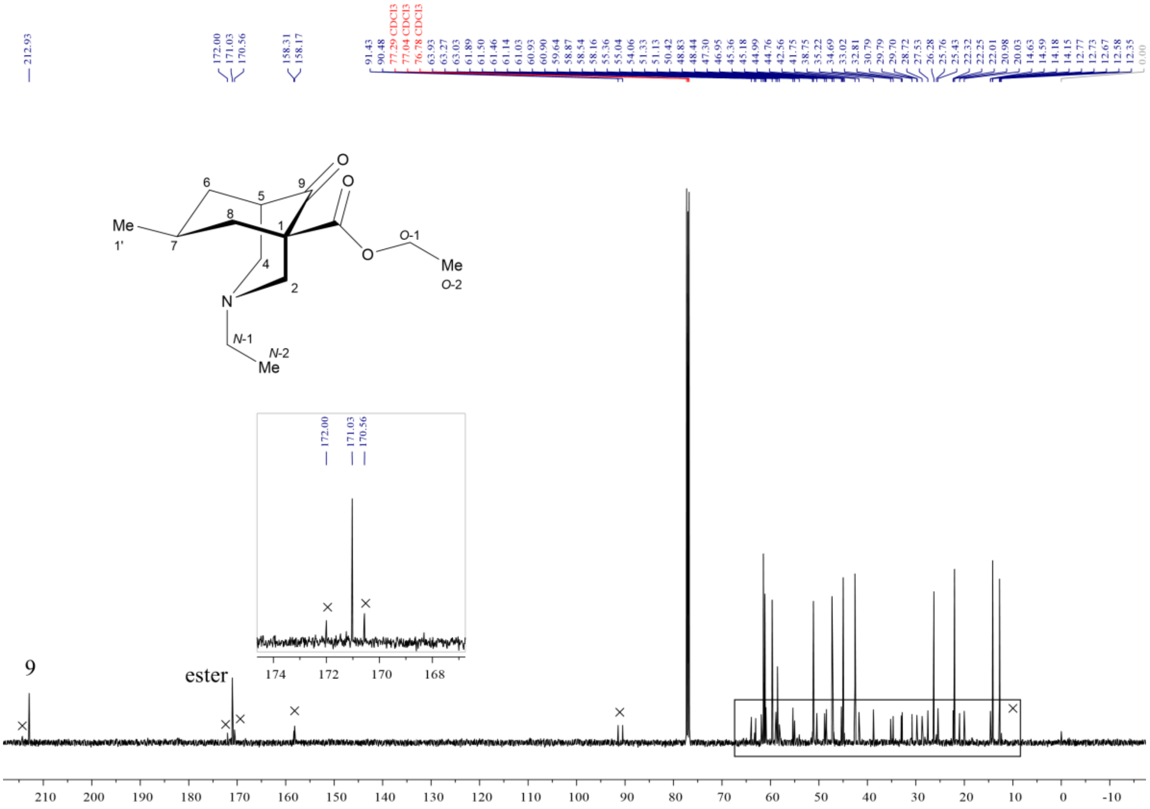

Figure S48. ^{13}C NMR spectrum of 7-Me [3.3.1]azabicycle (**14**, ethyl ester) in $CDCl_3$

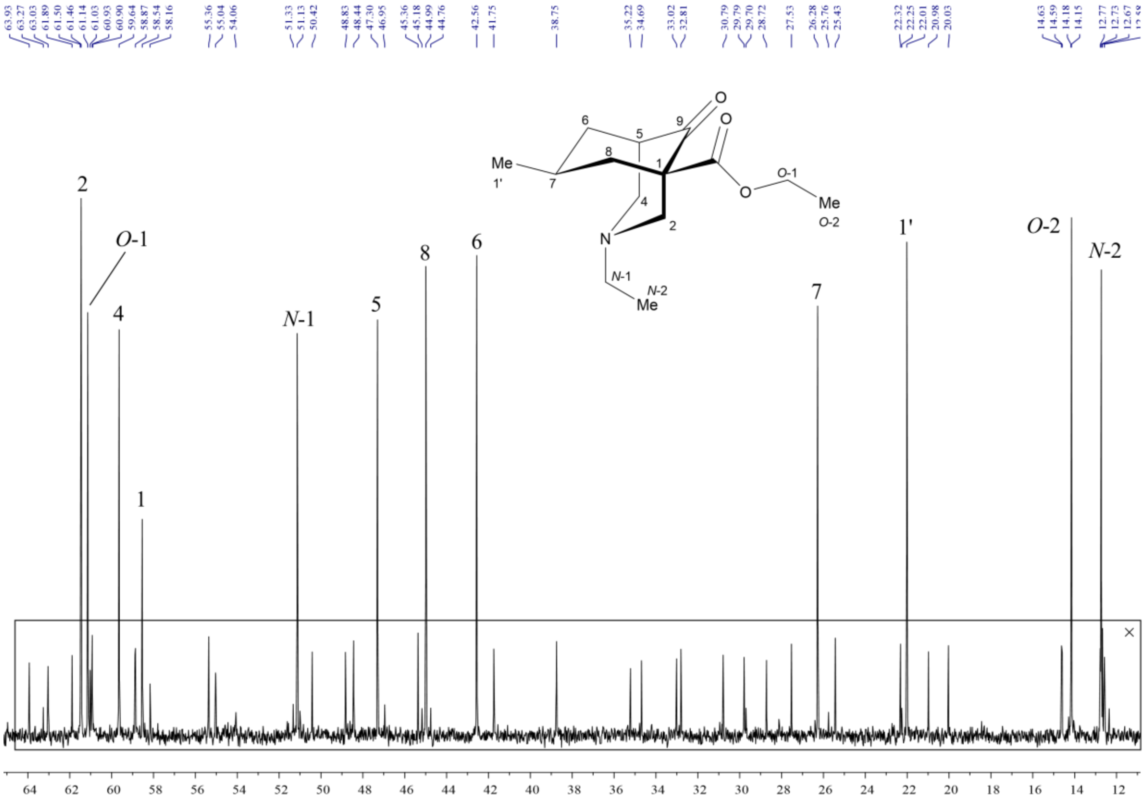

Figure S49. ^{13}C NMR expansion of 7-Me [3.3.1]azabicycle (**14**, ethyl ester) in $CDCl_3$ with assingments

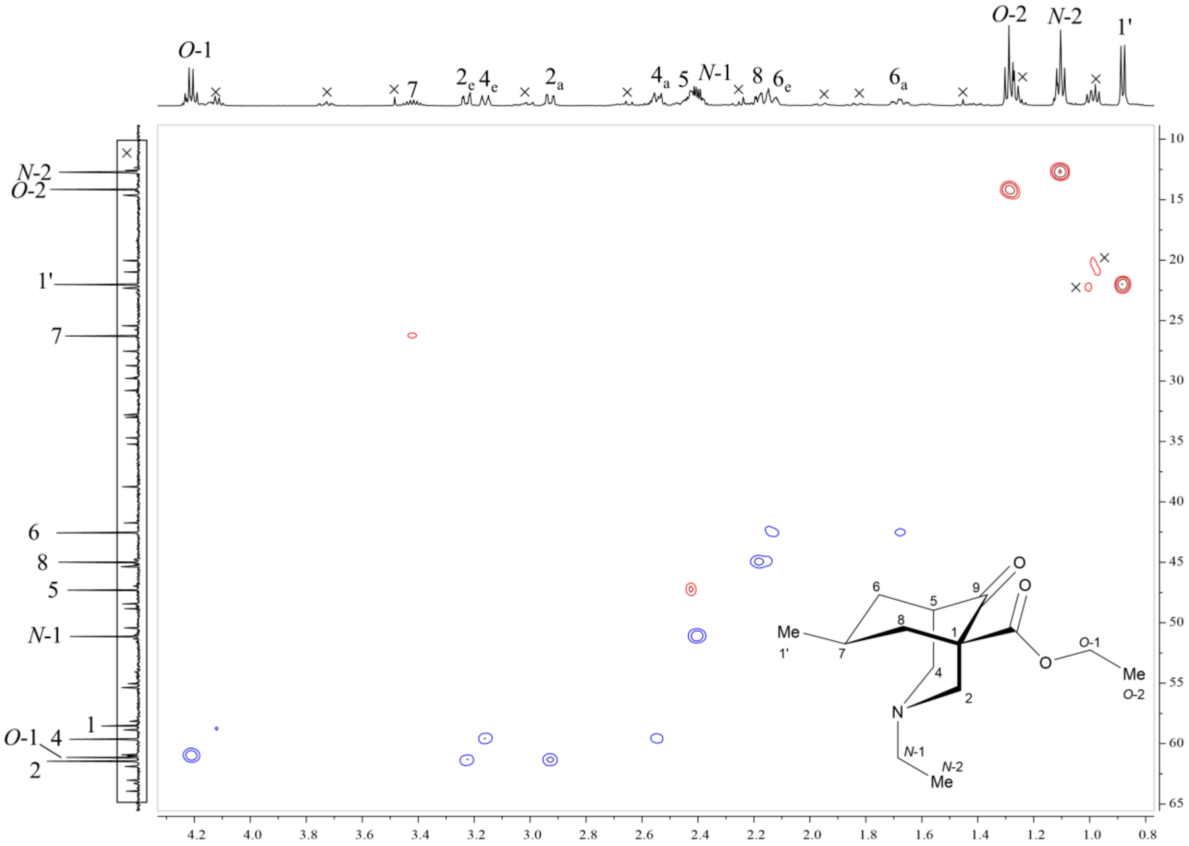

Figure S50. HSQC spectrum of 7-Me [3.3.1]azabicycle (**14**, ethyl ester) in $CDCl_3$

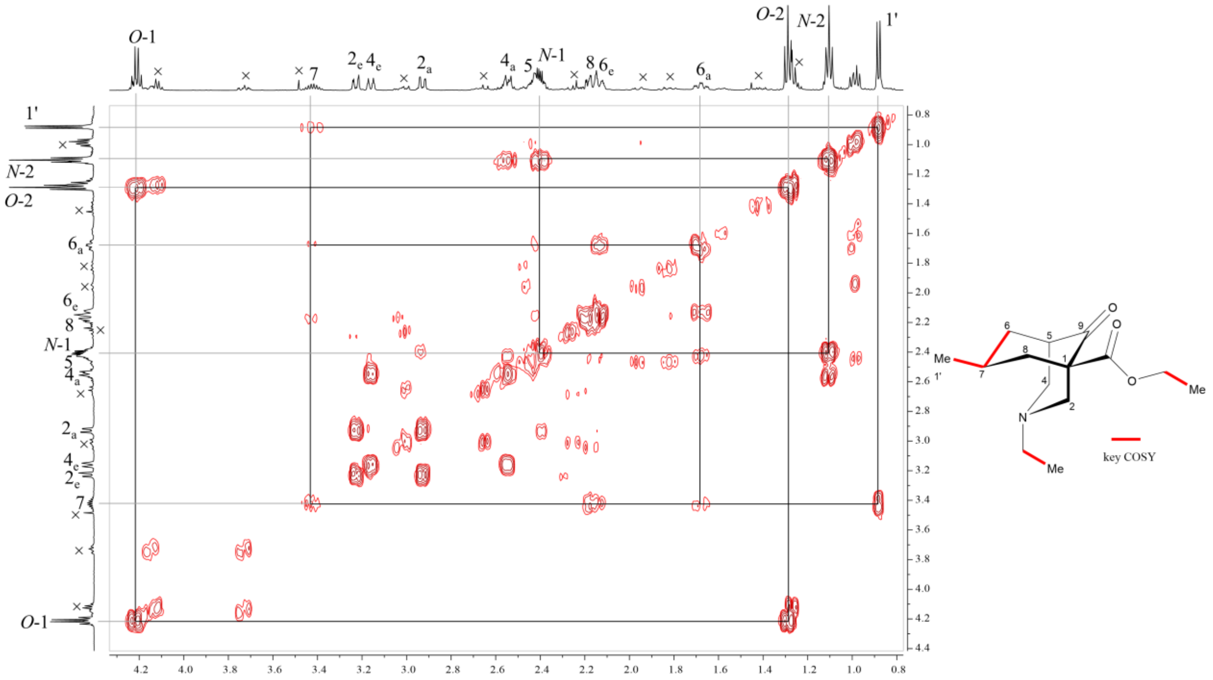

Figure S51. COSY spectrum of 7-Me [3.3.1]azabicycle (**14**, ethyl ester) in $CDCl_3$

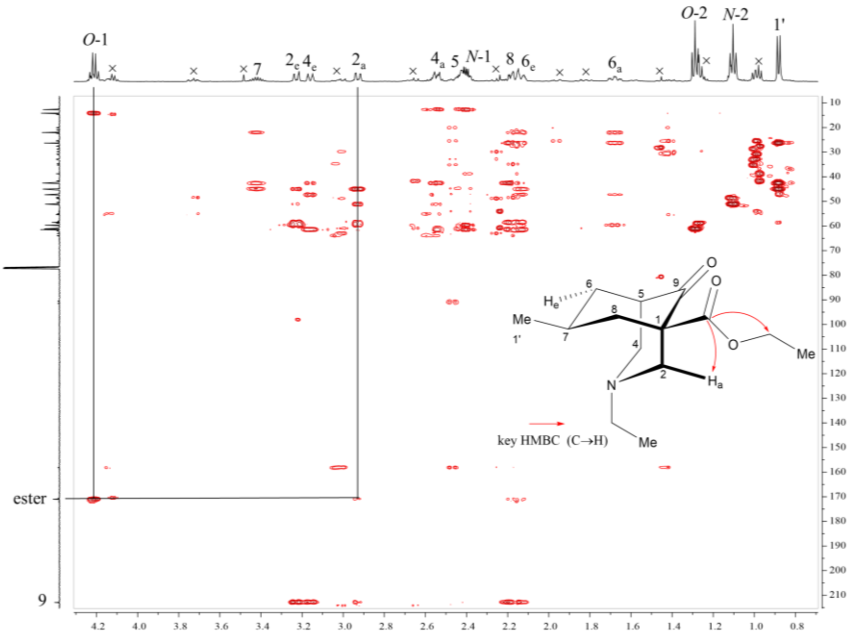

Figure S52. HMBC spectrum of 7-Me [3.3.1]azabicycle (**14**, ethyl ester) in $CDCl_3$

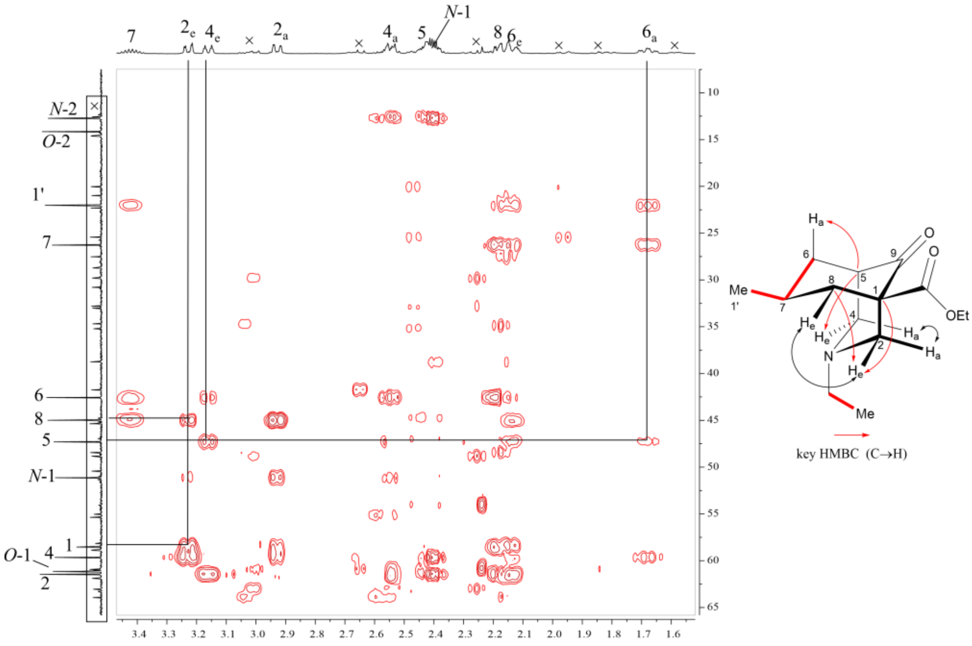

Figure S53. HMBC expansion of 7-Me [3.3.1]azabicycle (**14**, ethyl ester) in $CDCl_3$

The major component of 7-Me [3.3.1]azabicycle (**14**) is identified by HSQC, COSY, HMBC. The remaining signals (in both ^{1}H and ^{13}C) are due to the two conformational isomers (three ester carbonyl signals around 170.00 ppm are displayed, shown in Figure S48) rather than due to impurities as TLC of the purified product showed only a single spot (UV, λ = 254 nm; staining with iodine vapour, *p*-anisaldehyde solution and Dragendorff's reagent).

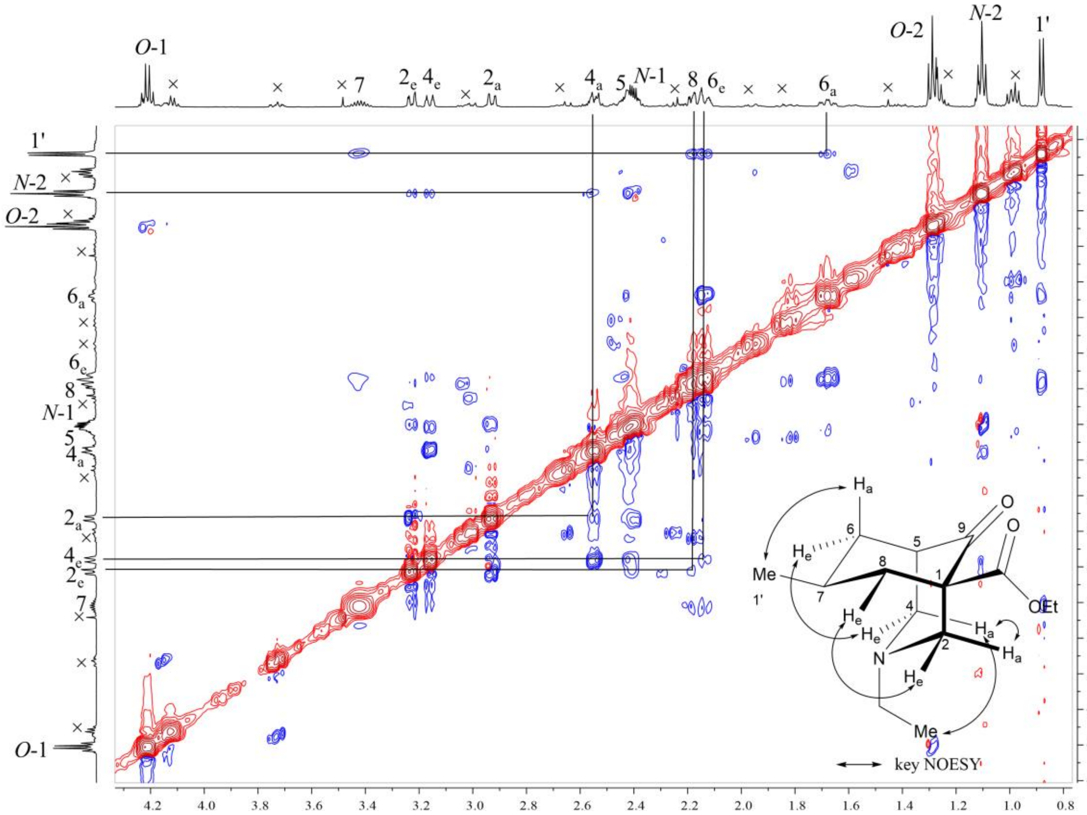

Figure S54. NOESY spectrum of 7-Me [3.3.1]azabicycle (**14**, ethyl ester) in $CDCl_3$

N,*N*-bis(ethoxymethyl)ethanamine (**16**)

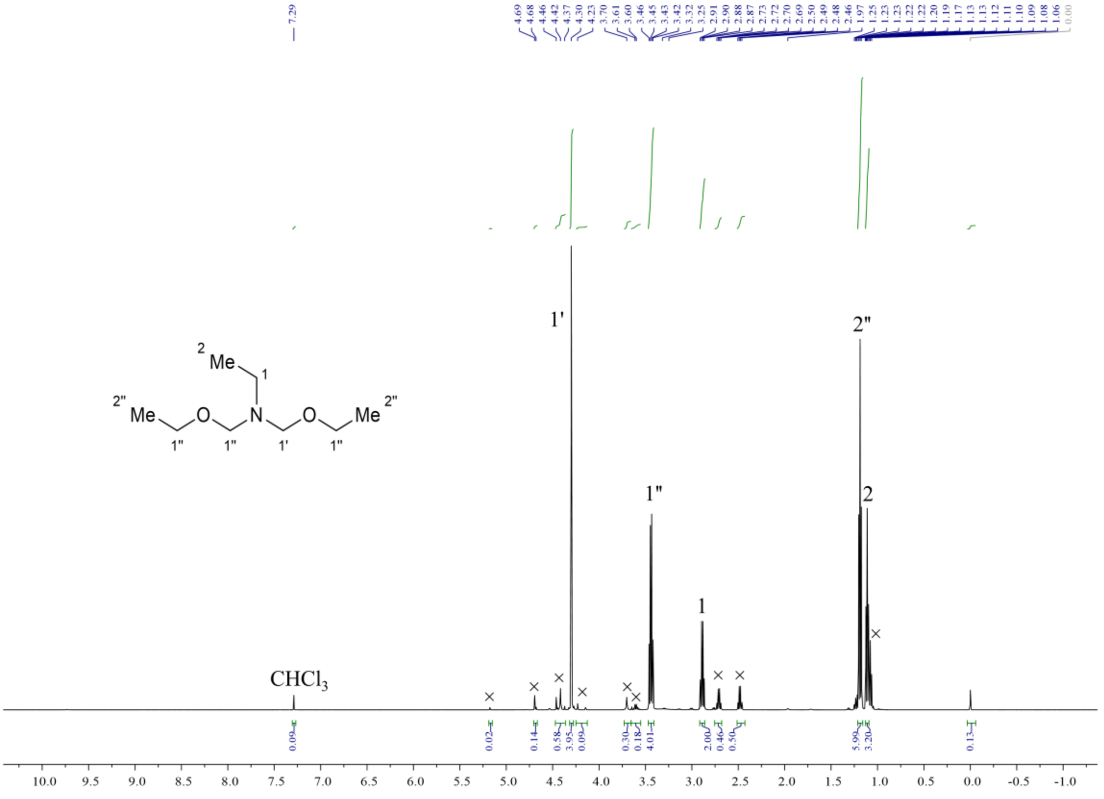

Figure S55. ^1H NMR spectrum of *N*,*N*-bis(ethoxymethyl)ethanamine (**16**) in $CDCl_3$

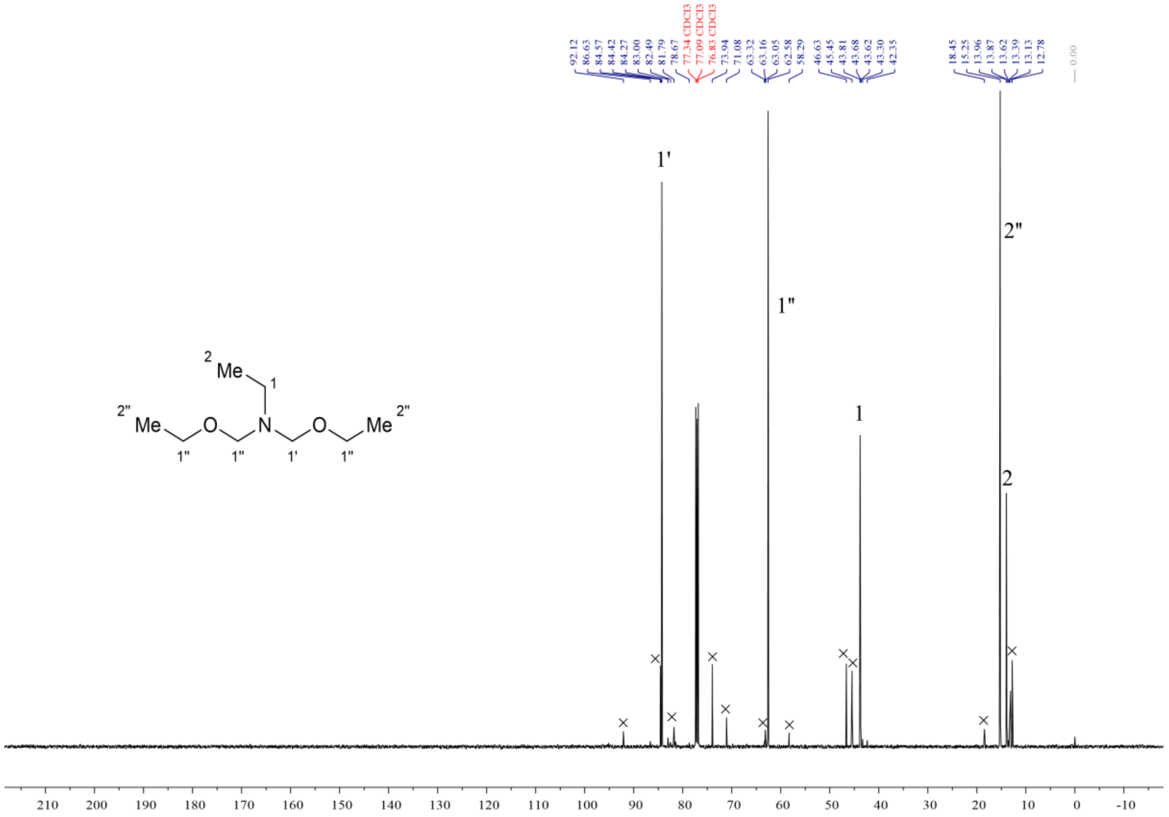

Figure S56. ^{13}C NMR spectrum of *N*,*N*-bis(ethoxymethyl)ethanamine (**16**) in $CDCl_3$

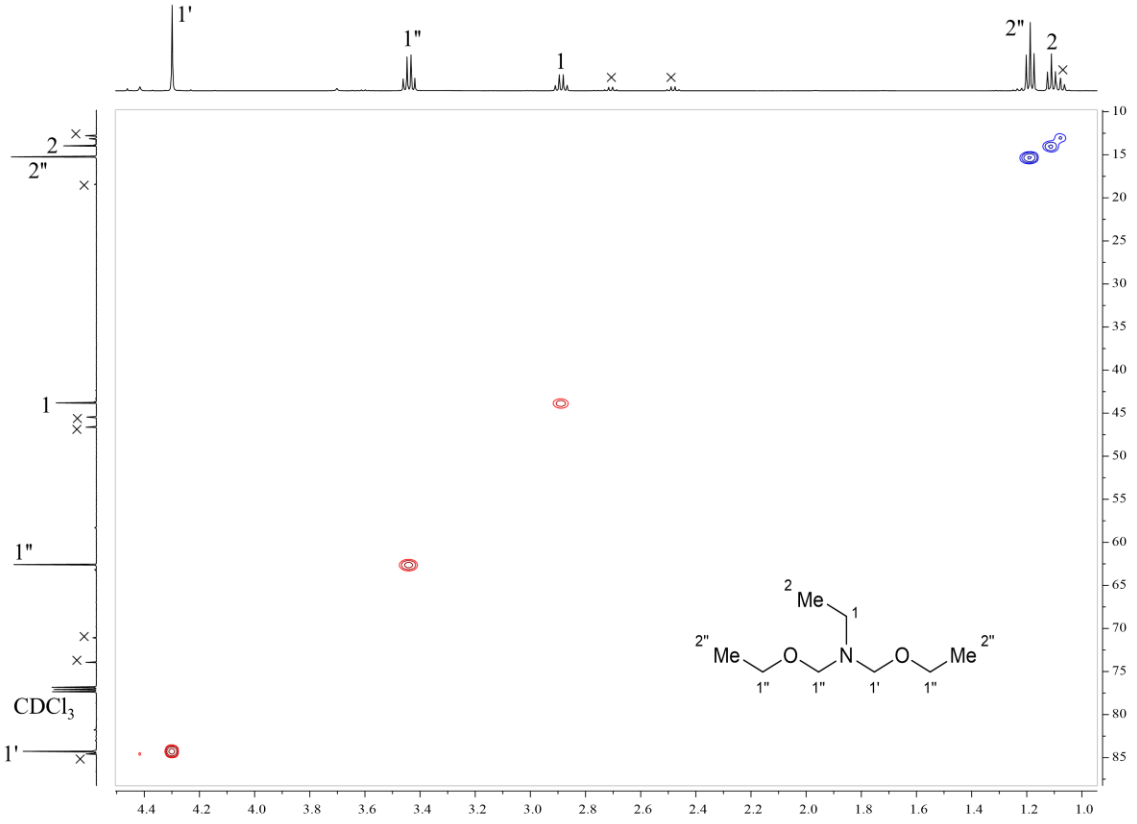

Figure S57. HSQC spectrum of *N*,*N*-bis(ethoxymethyl)ethanamine (**16**) in $CDCl_3$

Methyl 3-ethyl-7-methyl-9-oxo-3-azabicyclo[3.3.1]nonane-1-carboxylate (**17**)

Confirmation of Expected Formula

Sample-ID	bl_zz_7-Me AE bis final	Submitter	zz615 Ziyu Zeng
Analysis Name	bl_zz_7-Me AE bis final_356623_7_01_62718.d	Supervisor	- Ian Blagbrough
Method used	Confirm Formula Positive 50to500 loop inj.m	Acquisition Date	09/04/2018 22:03:31
Ionisation Mode	positive electrospray (ESI)		

+MS, 1.0-1.3min #(119-154), -Spectral Bkgrnd

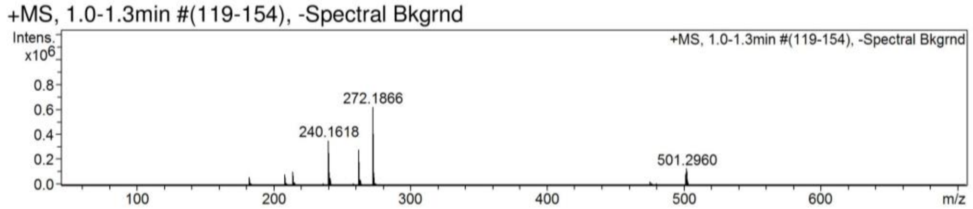

#	m/z	I	I %	Area	S/N
1	182.1545	58626	9.5	2207	1860.3
2	208.1342	78048	12.6	3190	1469.3
3	214.1805	102841	16.6	4393	1692.8
4	240.1618	353448	57.2	16975	3777.9
5	241.1644	52629	8.5	2289	554.8
6	262.1448	282101	45.7	13729	3533.3
7	263.1460	41539	6.7	1939	526.1
8	272.1866	617775	100.0	32659	8734.6
9	273.1893	88545	14.3	4643	1268.1
10	501.2960	129729	21.0	12656	4487.3

Generate Molecular Formula Parameters

Charge	Tolerance	SearchRadius	H/C Ratio min.	H/C Ratio max.	Electron Conf.	Nitrogen Rule	sigma limit
positive	25 ppm	0.05 m/z	0	3	both	true	0.05

Expected Formula C13H21NO3 **Adduct(s):** H, Na

#	meas. m/z	theo. m/z	Err[ppm]	Sigma	Formula
1	240.1618	240.1594	9.90	0.0028	C 13 H 22 N 1 O 3
1	262.1448	262.1414	13.10	0.0017	C 13 H 21 N 1 Na 1 O 3

Note: Sigma fits < 0.05 indicates high probability of correct MF.

For formula confirmation the mass error / accuracy at 200 Da should be better than 25 ppm, for 500 Da better than 10 ppm and for 1000 Da better than 5 ppm

Figure S58. MS data of 7-Me [3.3.1]azabicycle (**17**, methyl ester)

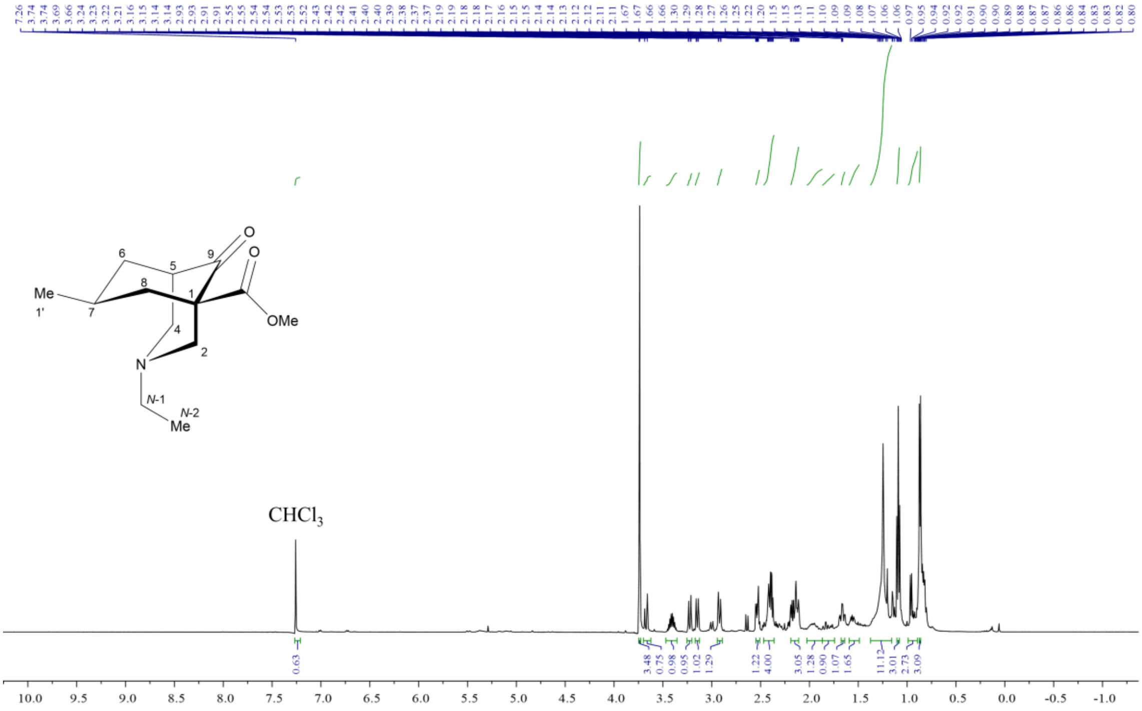

Figure S59. ^{1}H NMR spectrum of 7-Me [3.3.1]azabicycle (**17**, methyl ester) in $CDCl_3$

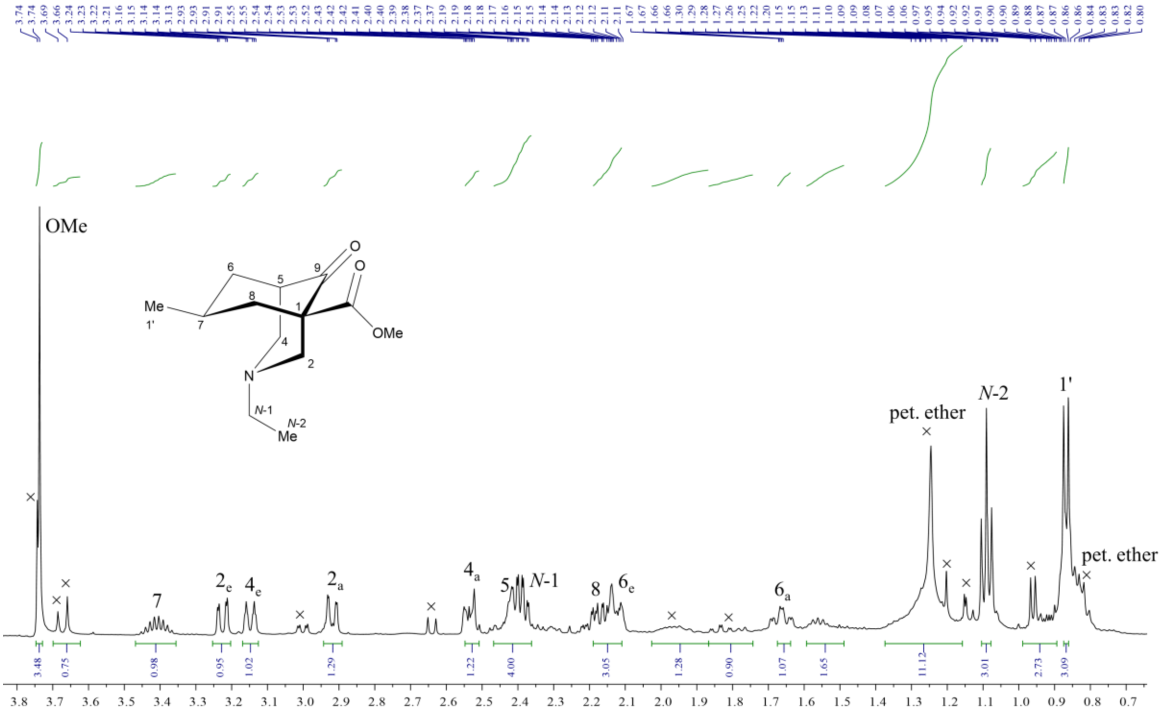

Figure S60. ^1H NMR expansion of 7-Me [3.3.1]azabicycle (**17**, methyl ester) in $CDCl_3$ assignments (residual petroleum ether is displayed)[1]

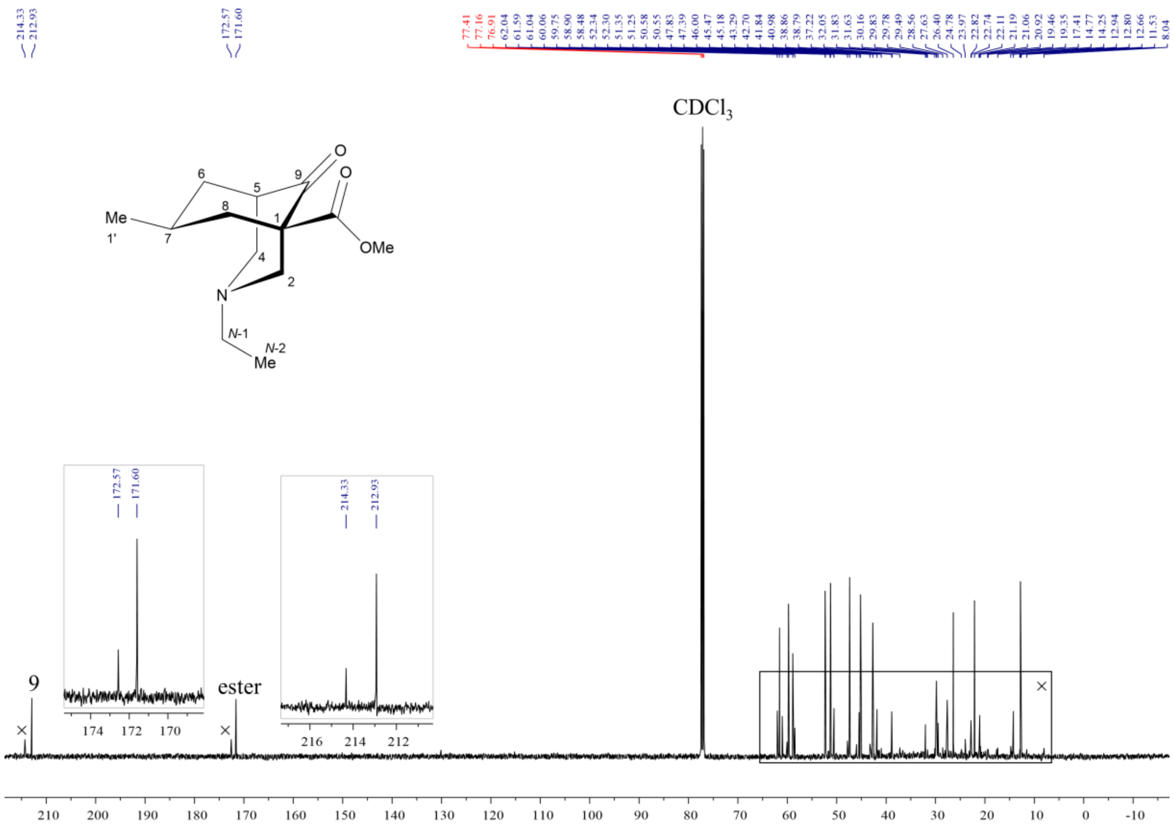

Figure S61. ^{13}C NMR spectrum of 7-Me [3.3.1]azabicycle (**17**, methyl ester) in $CDCl_3$

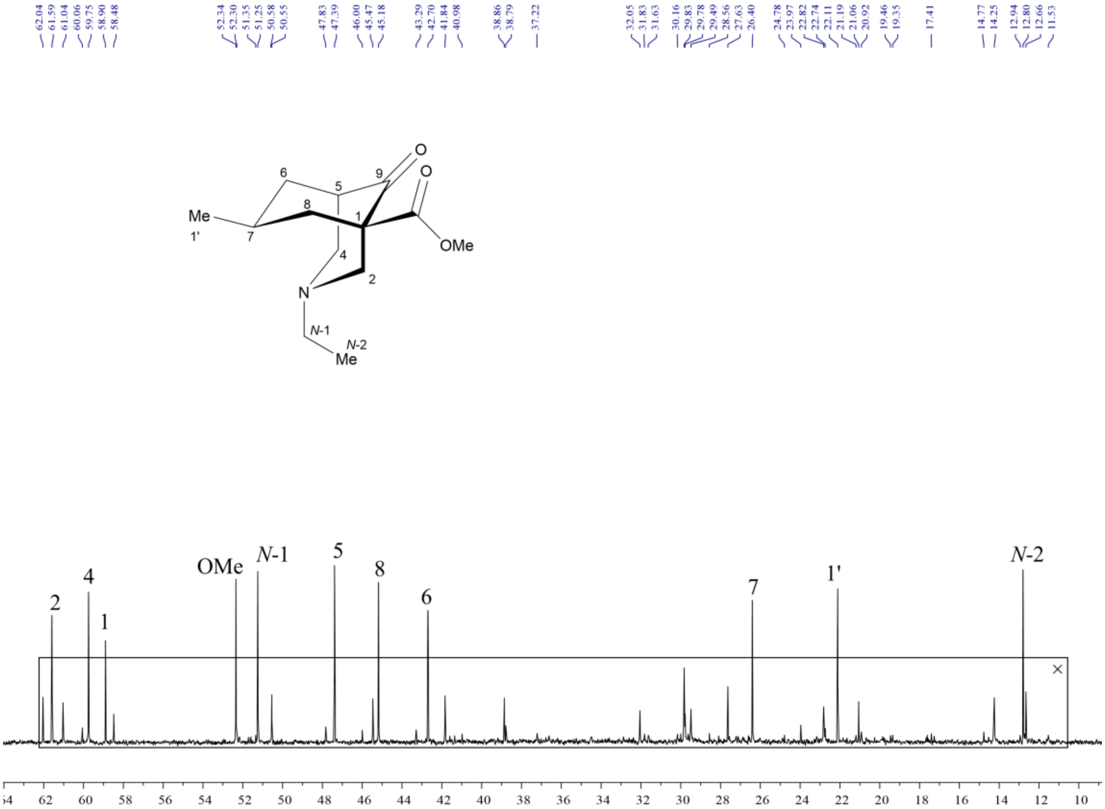

Figure S62. ^{13}C NMR expansion of 7-Me [3.3.1]azabicycle (**17**, methyl ester) in $CDCl_3$ with assignments

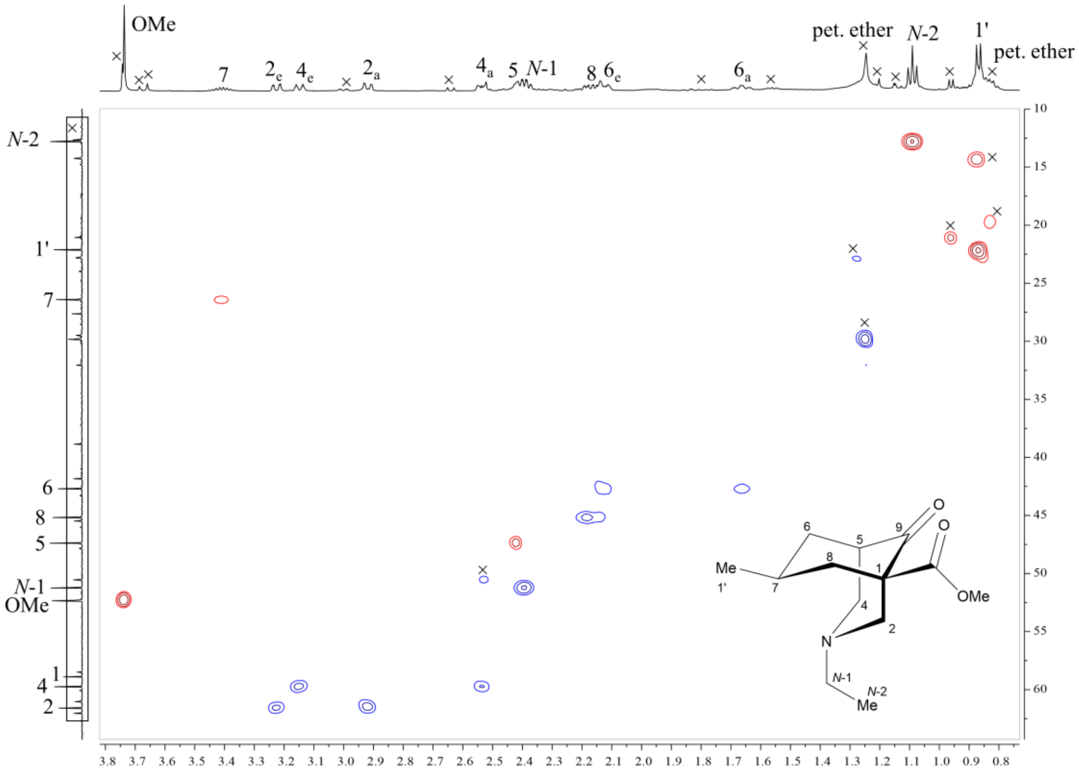

Figure S63. HSQC spectrum of 7-Me [3.3.1]azabicycle (**17**, methyl ester) in $CDCl_3$

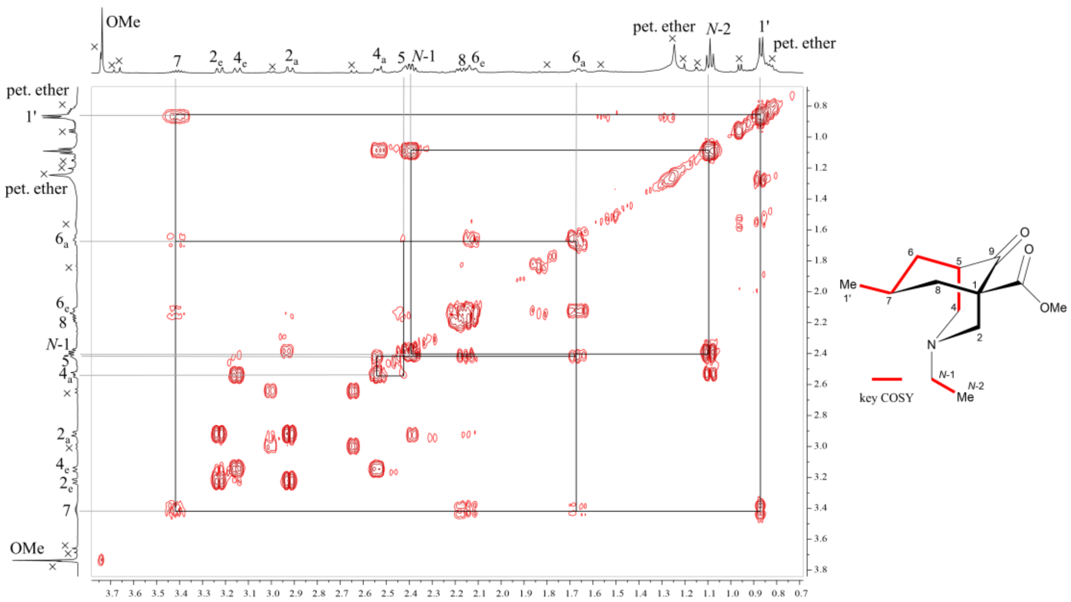

Figure S64. COSY spectrum of 7-Me [3.3.1]azabicycle (**17**, methyl ester) in $CDCl_3$

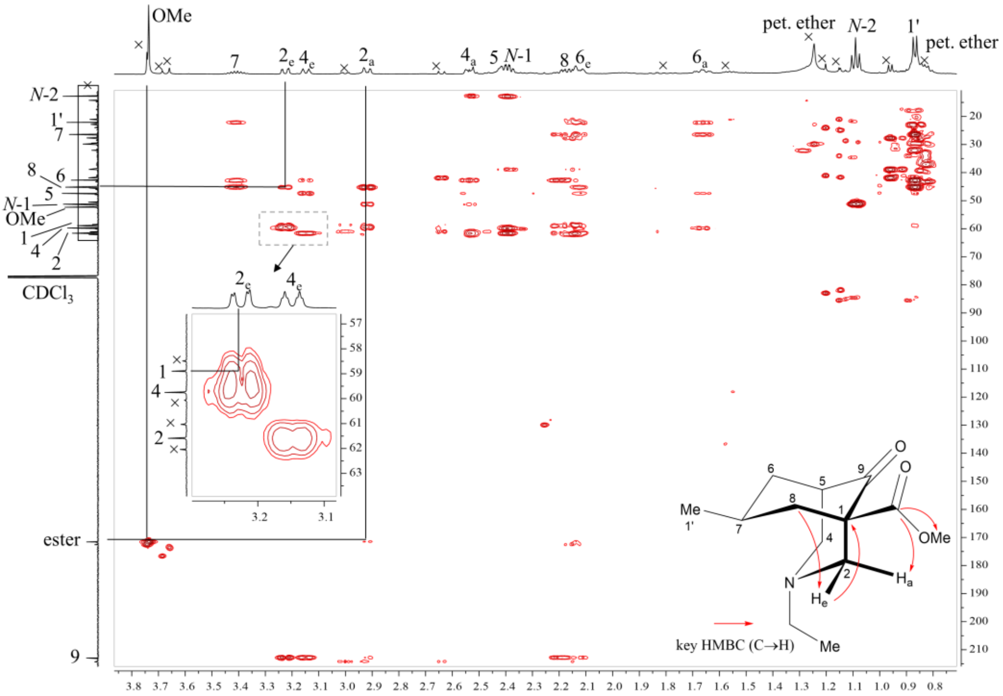

Figure S65. HMBC spectrum of 7-Me [3.3.1]azabicycle (**17**, methyl ester) in $CDCl_3$

Similar to 7-Me [3.3.1]azabicycle (**14**), the major component of 7-Me [3.3.1]azabicycle (**17**) is identified by HSQC, COSY, HMBC. The remaining signals (in both ^1H and ^{13}C) are due to the two conformational isomers rather than due to impurities as TLC of the purified product showed only a single spot (UV, λ = 254 nm; staining with iodine vapour, *p*-anisaldehyde solution and Dragendorff's reagent).

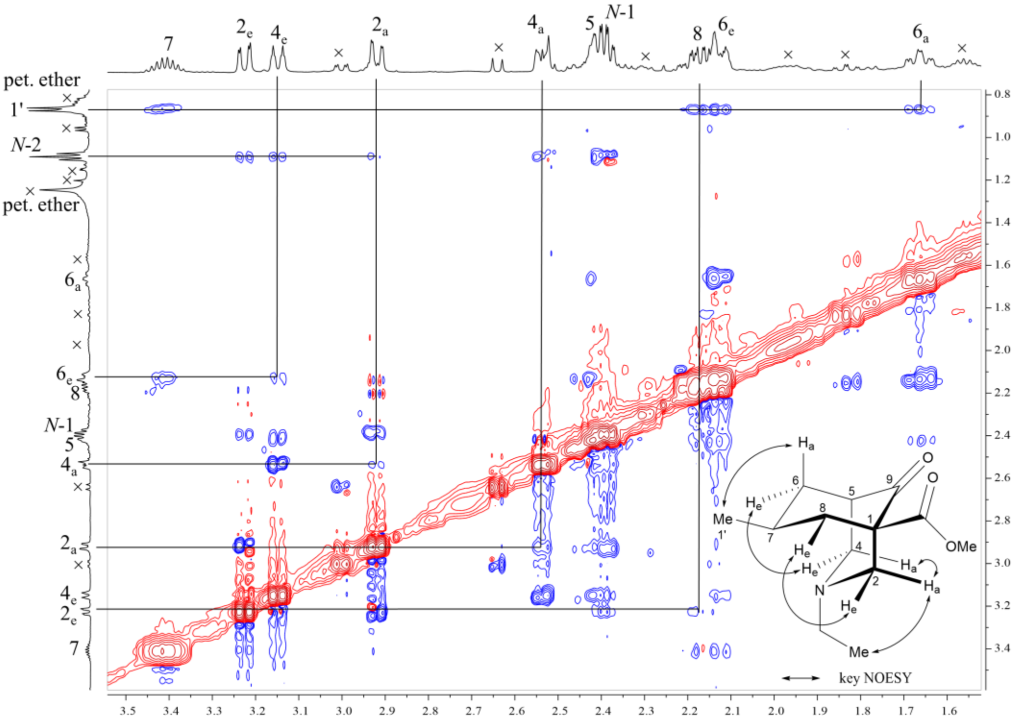

Figure S66. NOESY spectrum of 7-Me [3.3.1]azabicycle (**17**, methyl ester) in $CDCl_3$

Ethyl (*E*)-9-(2-(2,4-dinitrophenyl)hydrazinylidene)-3-ethyl-3-azabicyclo[3.3.1]nonane-1-carboxylate (**18**)

Confirmation of Expected Formula

Sample-ID	bl_zz_6-DNP BEST	Submitter	zz615 Ziyu Zeng
Analysis Name	bl_zz_6-DNP BEST_352760_93_01_58320.d	Supervisor	- Ian Blagbrough
Method used	Confirm Formula Positive 50to500 loop inj.m	Acquisition Date	09/08/2017 20:14:29
Ionisation Mode	positive electrospray (ESI)		

+MS, 1.0-1.3min #(119-154), -Spectral Bkgrnd

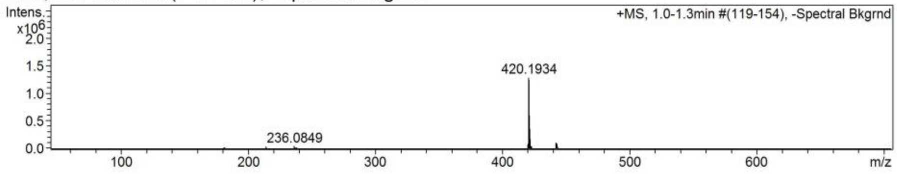

#	m/z	I	I %	Area	S/N
1	181.1112	14731	1.1	569	1090.3
2	214.0902	32964	2.5	1187	1497.8
3	236.0849	50648	3.9	863	1707.4
4	237.1624	21274	1.6	968	707.0
5	238.1694	24510	1.9	1146	805.4
6	420.1934	1296912	100.0	131918	9722.8
7	421.1930	374850	28.9	29747	2863.8
8	422.1959	53632	4.1	4395	417.0
9	442.1729	105804	8.2	9154	1359.5
10	443.1751	23708	1.8	1997	314.3

Generate Molecular Formula Parameters

Charge	Tolerance	SearchRadius	H/C Ratio min.	H/C Ratio max.	Electron Conf.	Nitrogen Rule	sigma limit
positive	20 ppm	0.05 m/z	0	3	both	true	0.05

Expected Formula C19H25N5O6 **Adduct(s):** H, Na

#	meas. m/z	theo. m/z	Err[ppm]	Sigma	Formula
1	420.1934	420.1878	13.50	0.0377	C 19 H 26 N 5 O 6
1	442.1729	442.1697	7.30	0.0006	C 19 H 25 N 5 Na 1 O 6

Note: Sigma fits < 0.05 indicates high probability of correct MF, and mass accuracy of 5ppm or better is generally acceptable for publication

Figure S67. MS data of [3.3.1]azabicyclic DNP derivative (**18**)

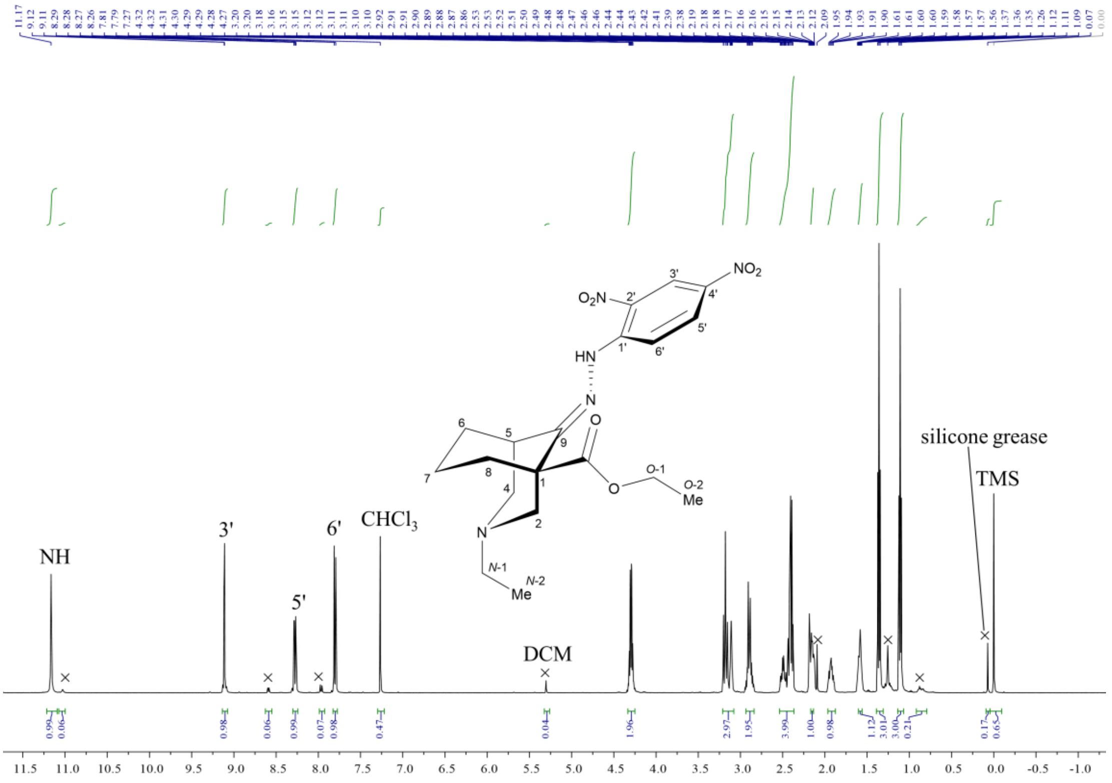

Figure S68. ^{1}H NMR spectrum of [3.3.1]azabicyclic DNP derivative (**18**) in $CDCl_3$ (residual DCM and residual silicone grease are displayed)[1]

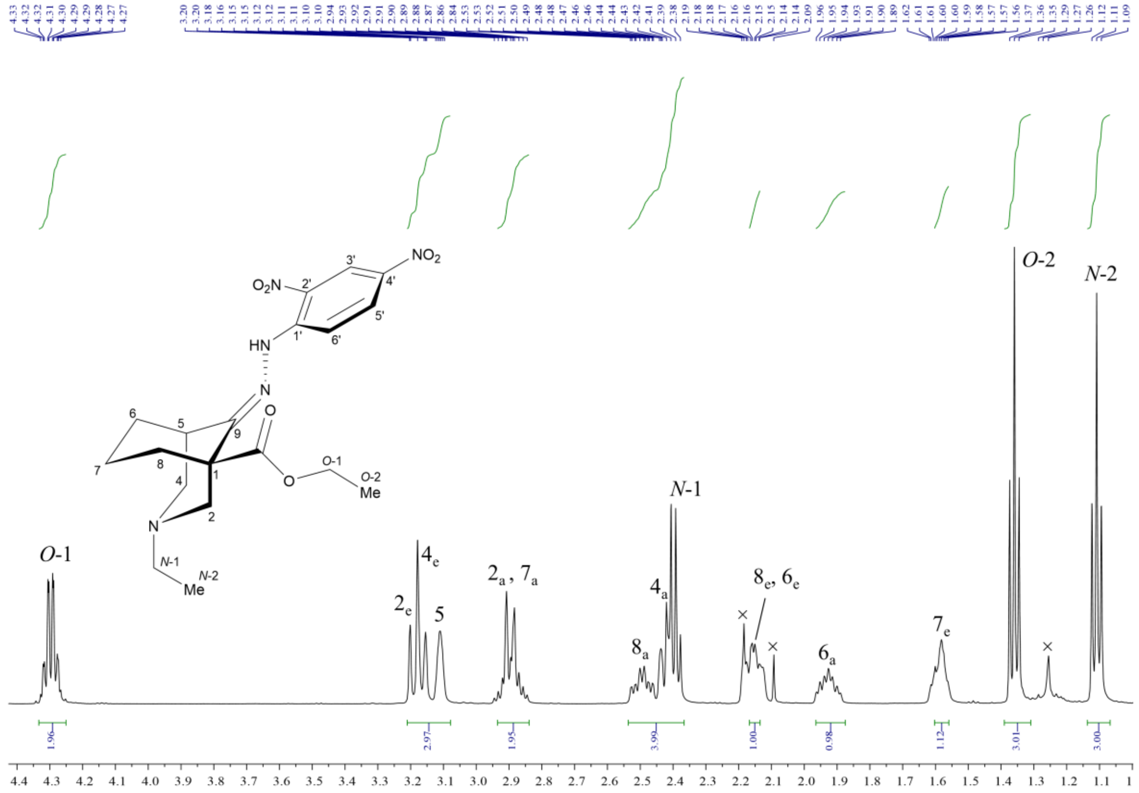

Figure S69. ^{1}H NMR expansion of [3.3.1]azabicyclic DNP derivative (**18**) in $CDCl_3$ with assignments

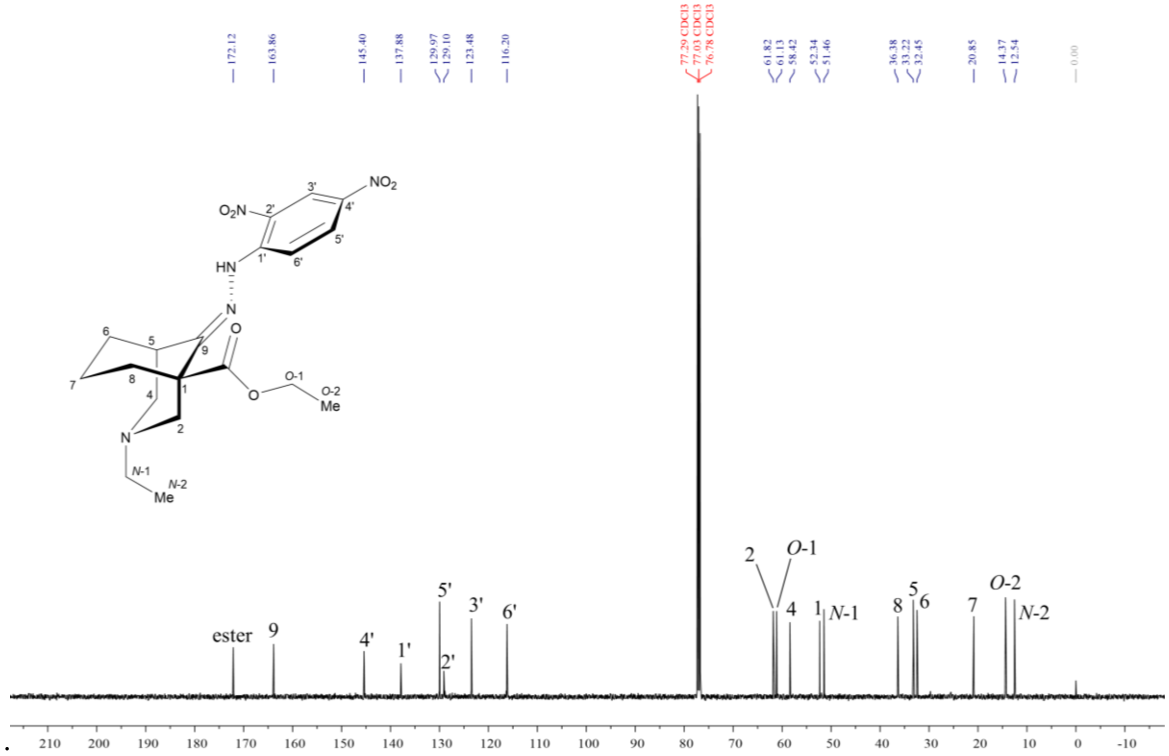

Figure S70. ^{13}C NMR spectrum of [3.3.1]azabicyclic DNP derivative (**18**) in $CDCl_3$ with assignments

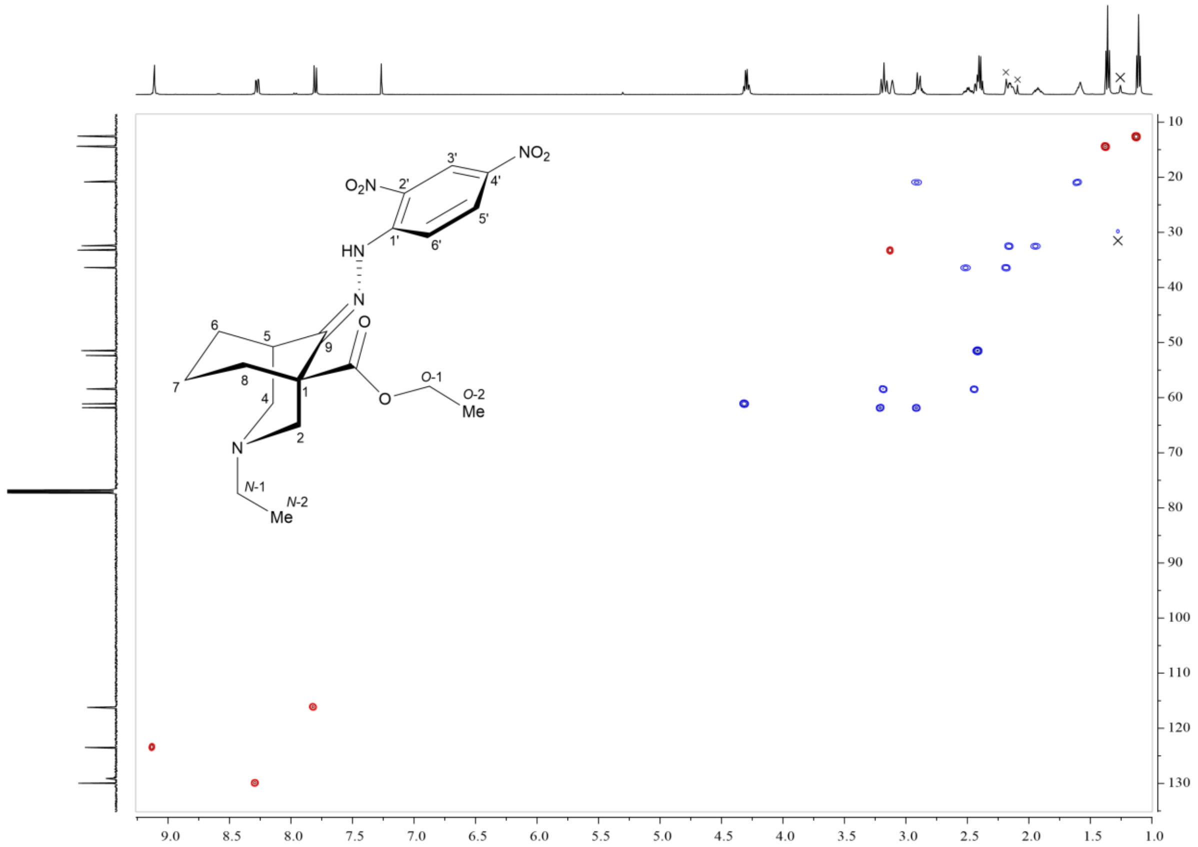

Figure S71. HSQC spectrum of [3.3.1]azabicyclic DNP derivative (**18**) in $CDCl_3$

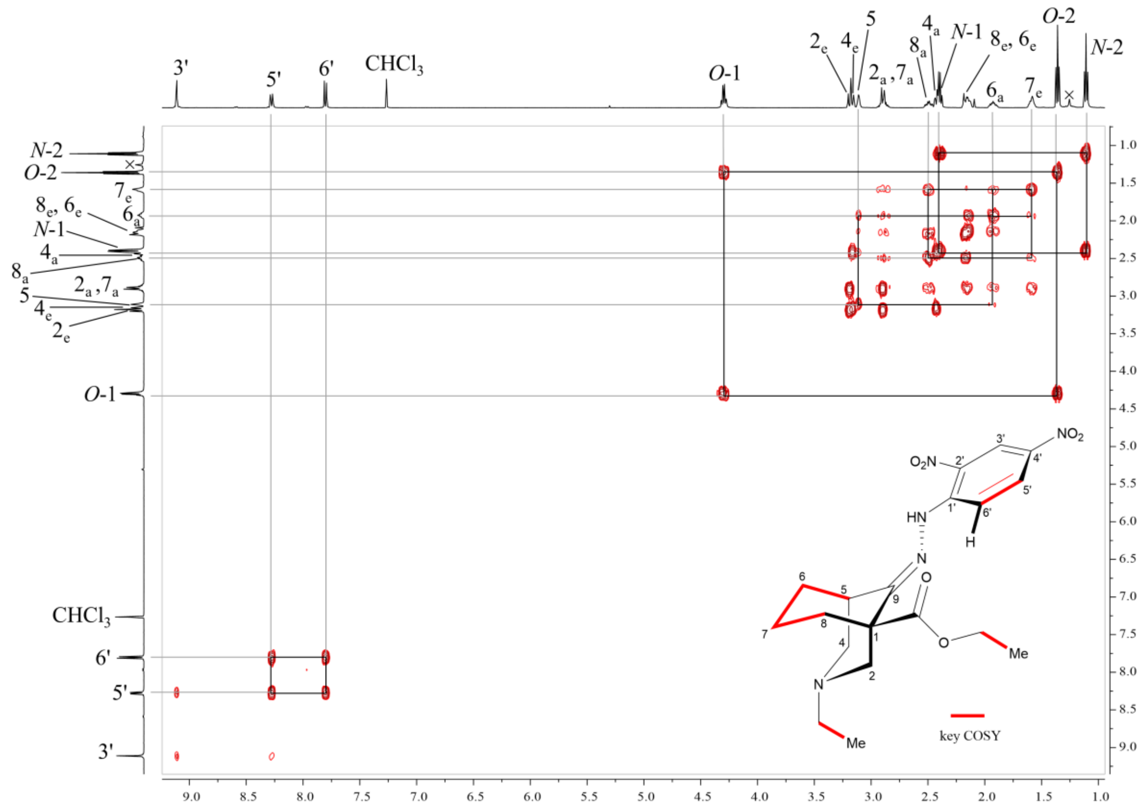

Figure S72. COSY spectrum of [3.3.1]azabicyclic DNP derivative (**18**) in $CDCl_3$

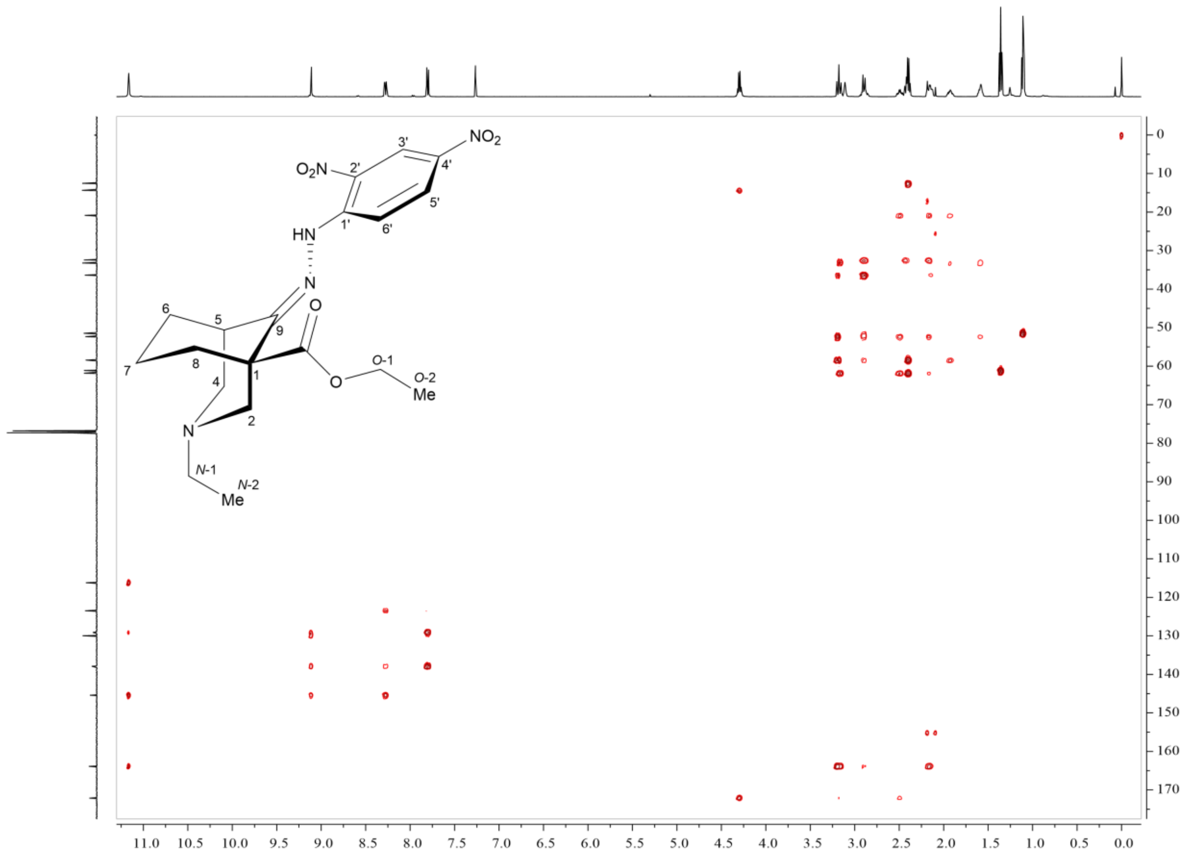

Figure S73. HMBC spectrum of [3.3.1]azabicyclic DNP derivative (**18**) in $CDCl_3$

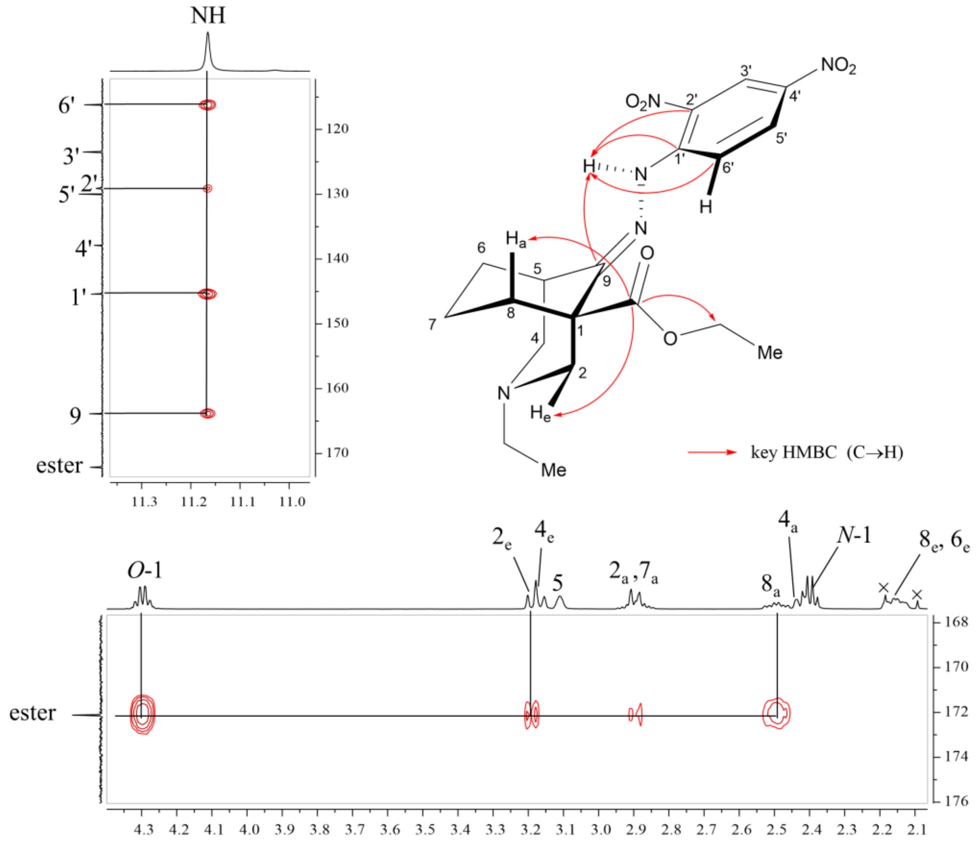

.))

Figure S74. HMBC expansions of [3.3.1]azabicyclic DNP derivative (**18**) in $CDCl_3$

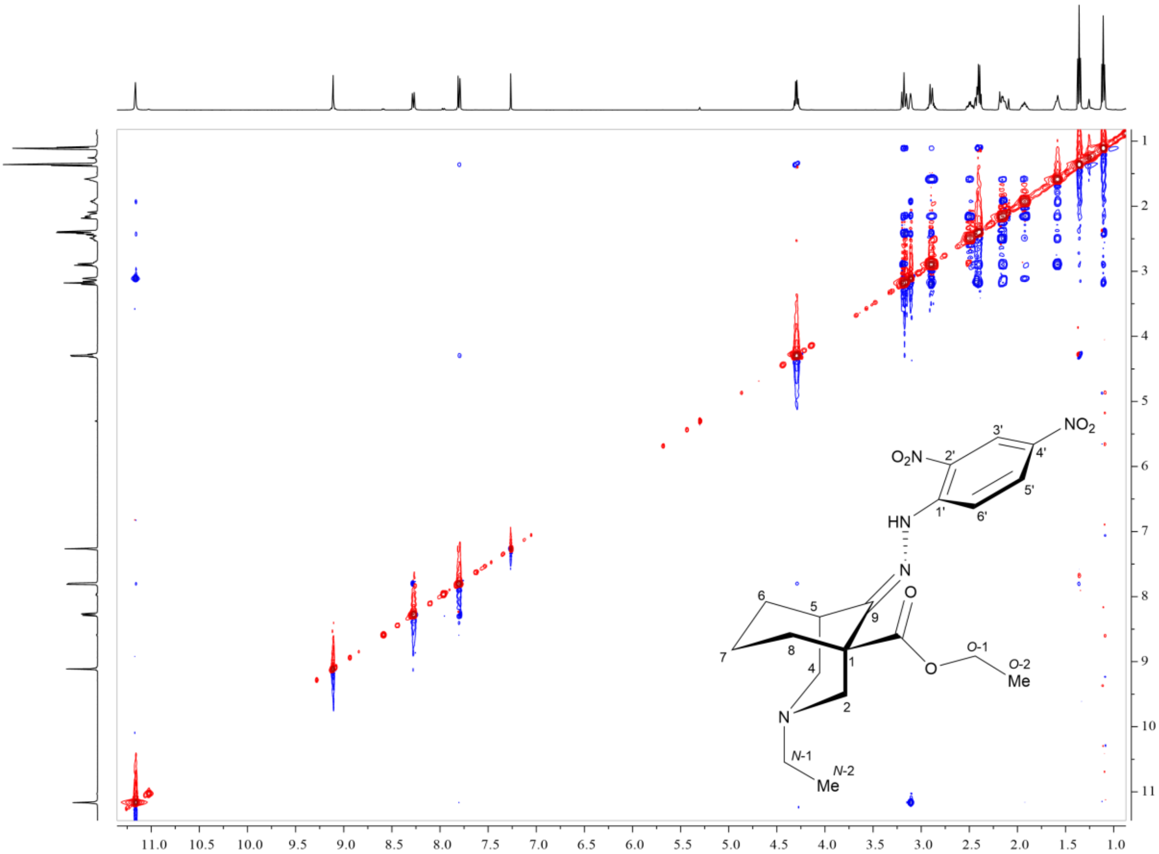

Figure S75. NOESY spectrum of [3.3.1]azabicyclic DNP derivative (**18**) in $CDCl_3$

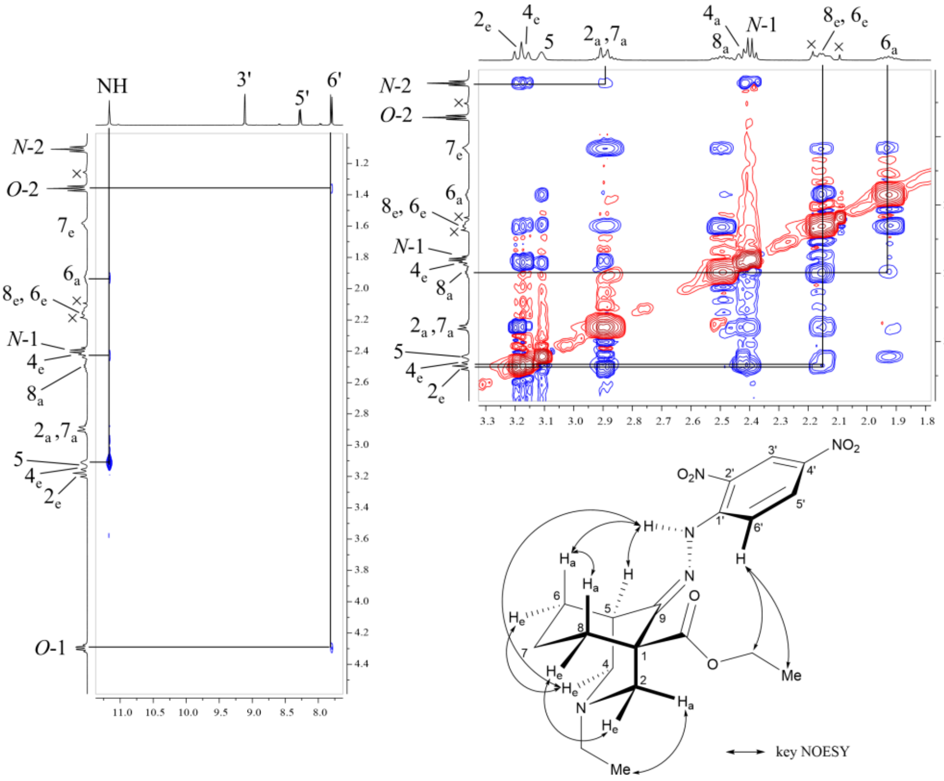

Figure S76. NOESY expansions of [3.3.1]azabicyclic DNP derivative (**18**) in $CDCl_3$

Methyl (*E*)-9-(2-(2,4-dinitrophenyl)hydrazinylidene)-3-ethyl-7-isopropyl-3-azabicyclo[3.3.1]nonane-1-carboxylate (**19**)

Confirmation of Expected Formula

Sample-ID	bl_zz_7-iso + DNP	Submitter	zz615 Ziyu Zeng
Analysis Name	bl_zz_7-iso + DNP_351992_56_01_57453.d	Supervisor	- Ian Blagbrough
Method used	Confirm Formula Positive 50to500 loop inj.m	Acquisition Date	26/04/2017 22:12:32
Ionisation Mode	positive electrospray (ESI)		

+MS, 1.0-1.3min #(60-77), -Spectral Bkgrnd

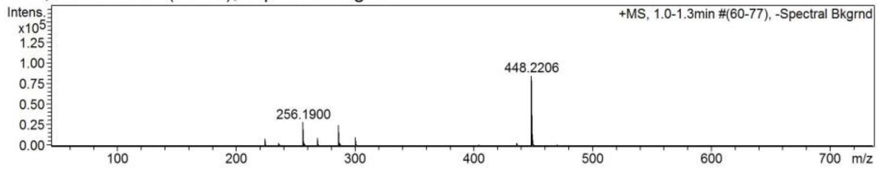

#	m/z	I	I %	Area	S/N
1	224.1641	8271	9.8	353	2412.2
2	236.0730	3355	4.0	102	775.1
3	256.1900	28208	33.3	1101	5159.2
4	257.1939	2446	2.9	123	448.4
5	268.1910	9303	11.0	420	1752.4
6	286.1996	24897	29.4	1131	4910.5
7	300.2167	10051	11.9	522	2115.0
8	436.2193	3542	4.2	271	300.7
9	448.2206	84794	100.0	5057	8995.6
10	449.2224	14688	17.3	1049	1591.3

Generate Molecular Formula Parameters

Charge	Tolerance	SearchRadius	H/C Ratio min.	H/C Ratio max.	Electron Conf.	Nitrogen Rule	sigma limit
positive	10 ppm	0.05 m/z	0	3	both	true	0.05

Expected Formula C21H29N5O6 **Adduct(s):** H, Na

#	meas. m/z	theo. m/z	Err[ppm]	Sigma	Formula
1	448.2206	448.2191	3.40	0.0444	C 21 H 30 N 5 O 6
1	470.2026	470.2010	3.50	0.0453	C 21 H 29 N 5 Na 1 O 6

Note: Sigma fits < 0.05 indicates high probability of correct MF, and mass accuracy of 5ppm or better is generally acceptable for publication

Figure S77. MS data of 7-iPr [3.3.1]azabicyclic DNP derivative (**19**)

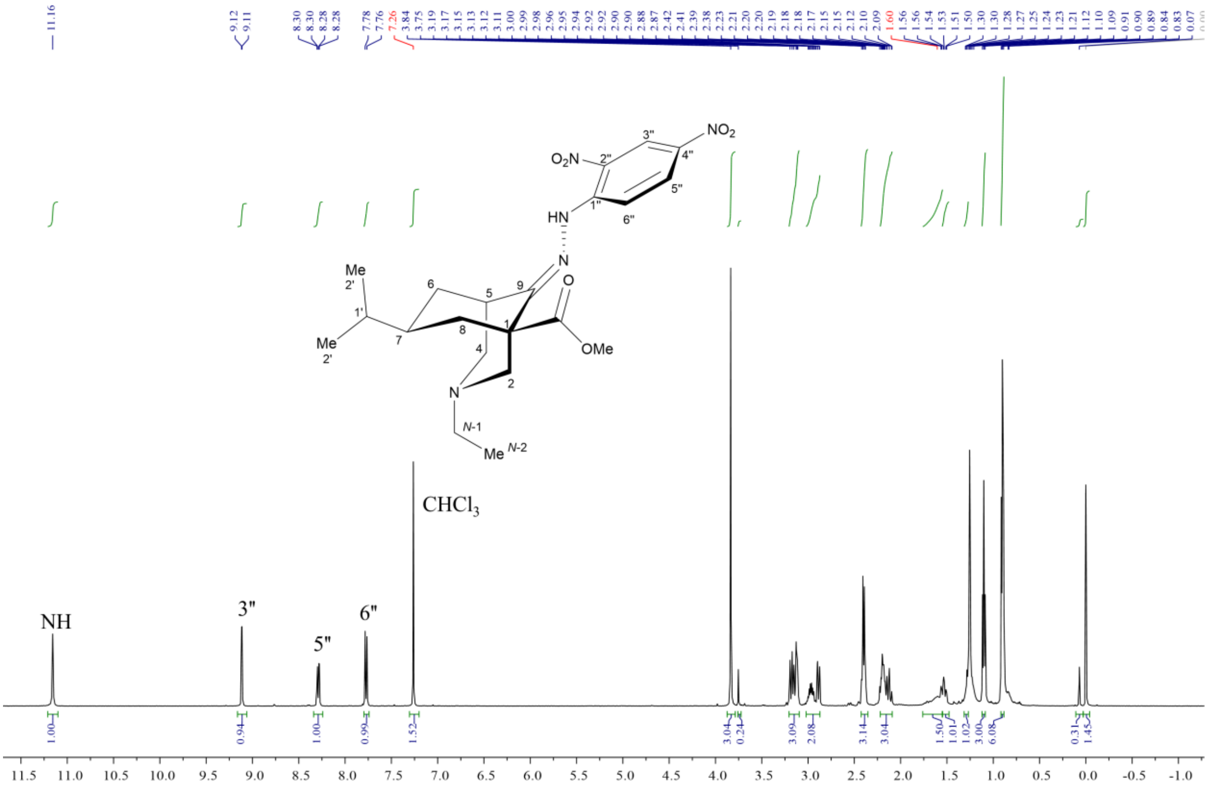

Figure S78. ^{1}H NMR spectrum of 7-iPr [3.3.1]azabicyclic DNP derivative (**19**) in $CDCl_3$

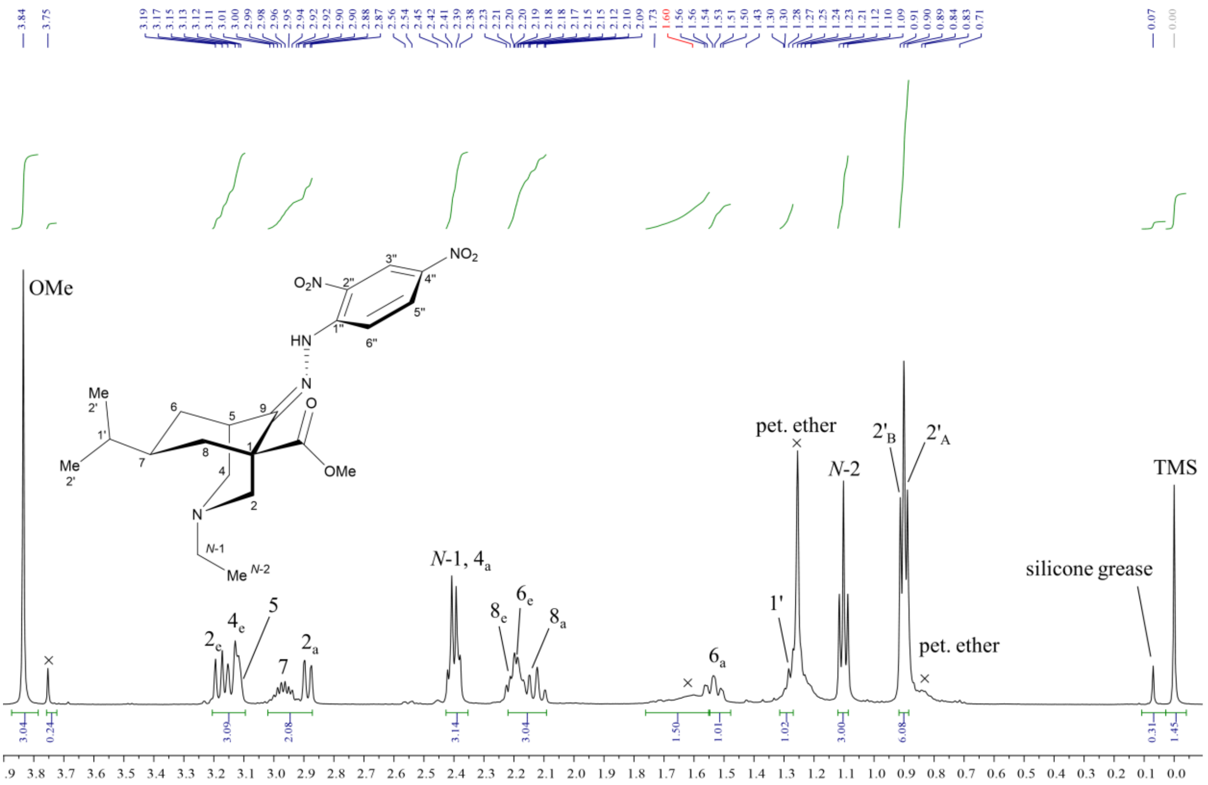

Figure S79. ^{1}H NMR expansion of 7-iPr [3.3.1]azabicyclic DNP derivative (**19**) in $CDCl_3$ with assignments (residual petroleum ether and silicone grease are displayed)[1]

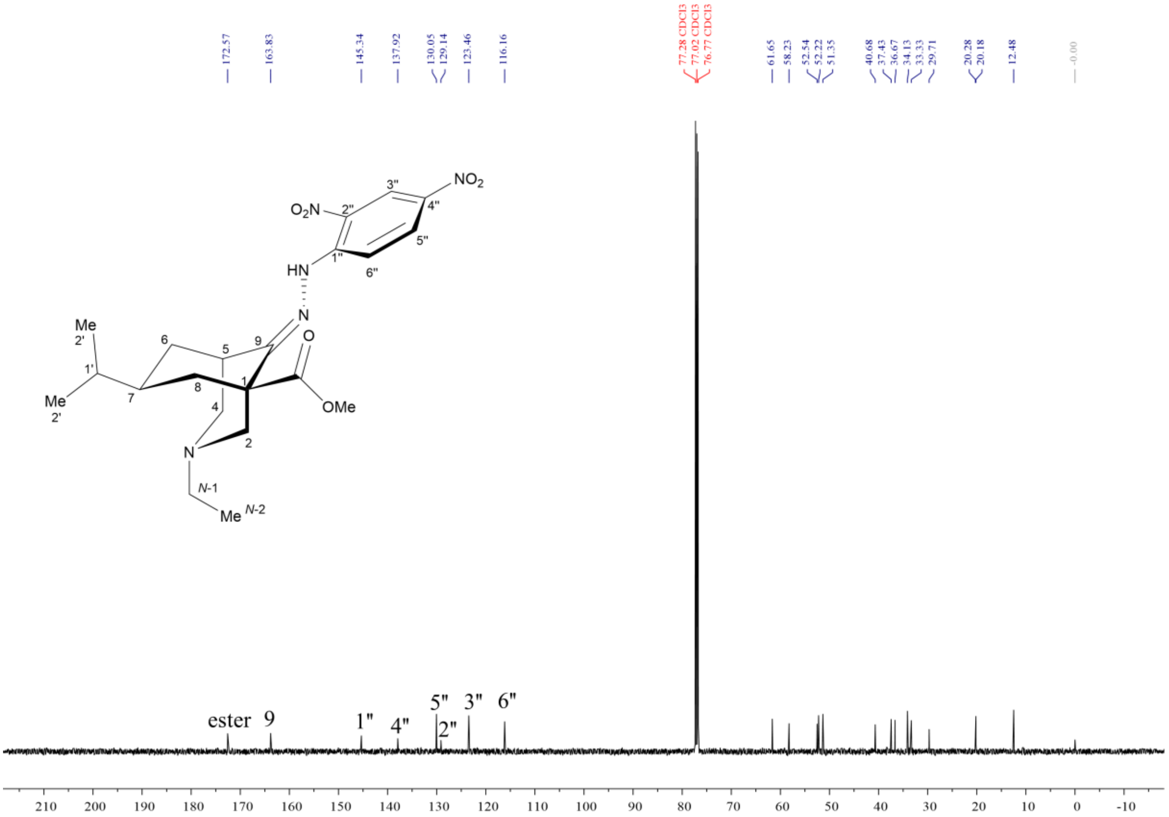

Figure S80. ^{13}C NMR spectrum of 7-iPr [3.3.1]azabicyclic DNP derivative (**19**) in $CDCl_3$

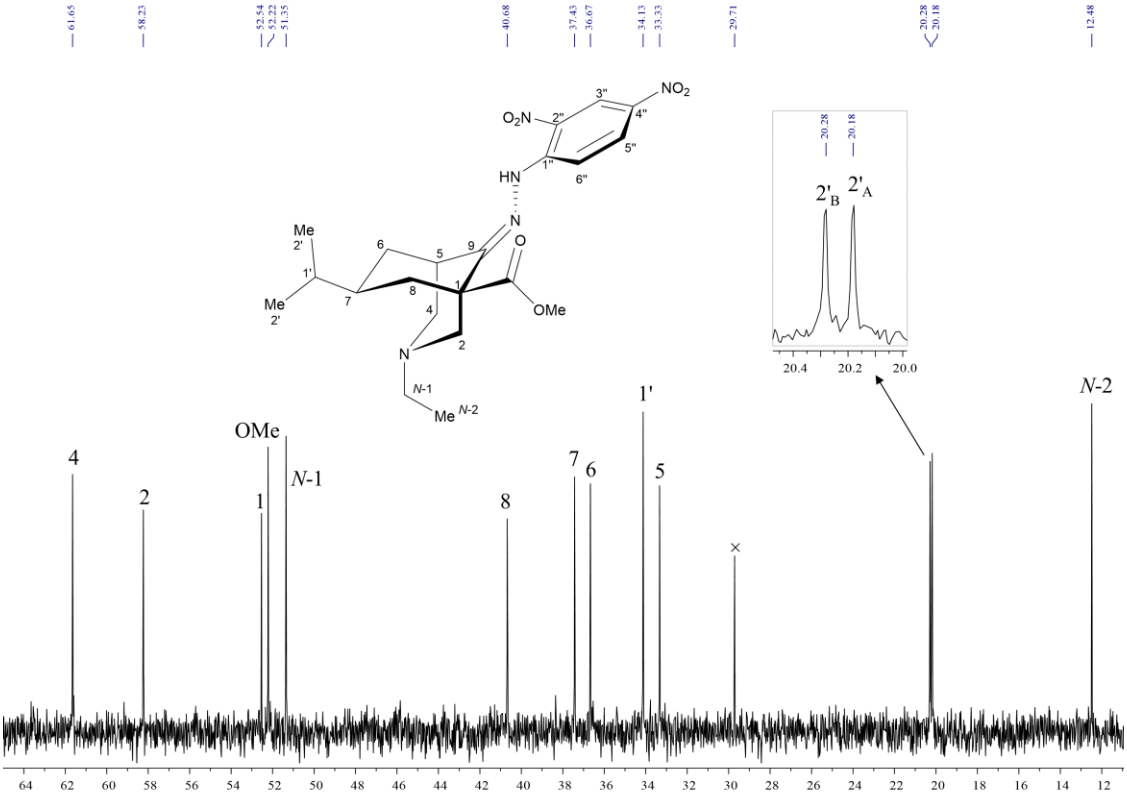

Figure S81. ^{13}C NMR expansion of 7-iPr [3.3.1]azabicyclic DNP derivative (**19**) in $CDCl_3$ with assignments (residual petroleum ether is displayed at 29.71 ppm)[1]

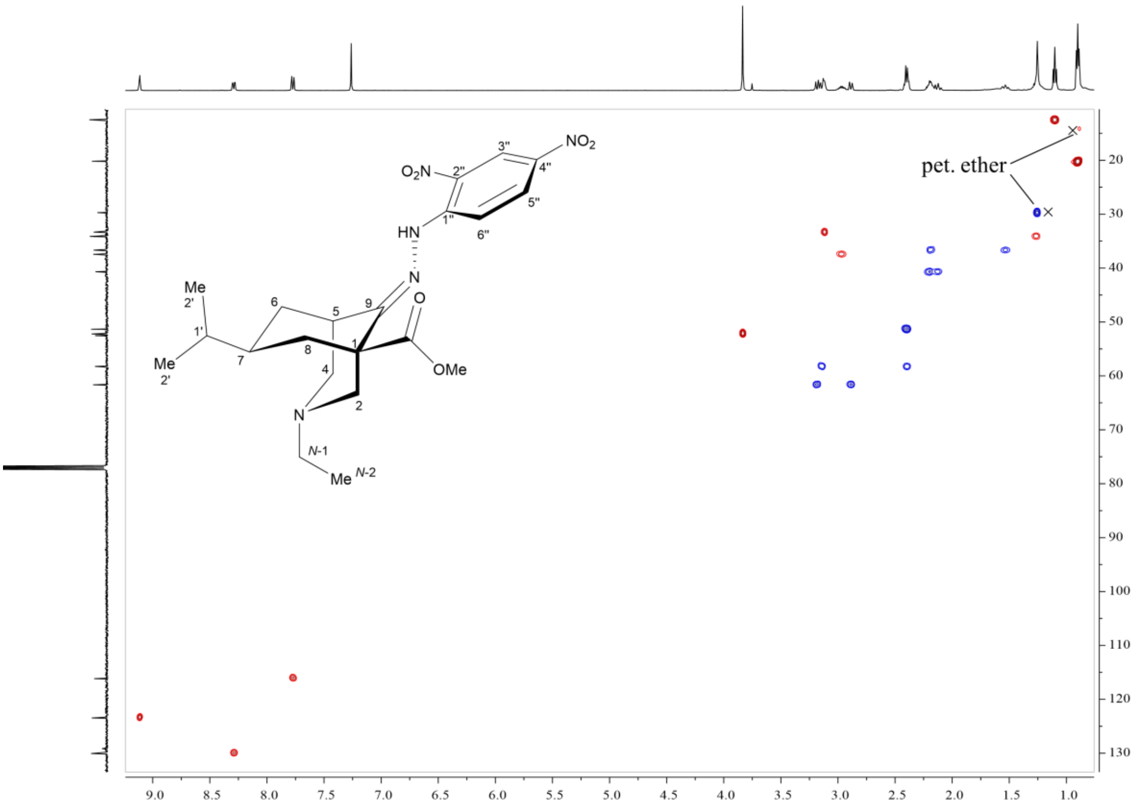

Figure S82. HSQC spectrum of 7-iPr [3.3.1]azabicyclic DNP derivative (**19**) in $CDCl_3$

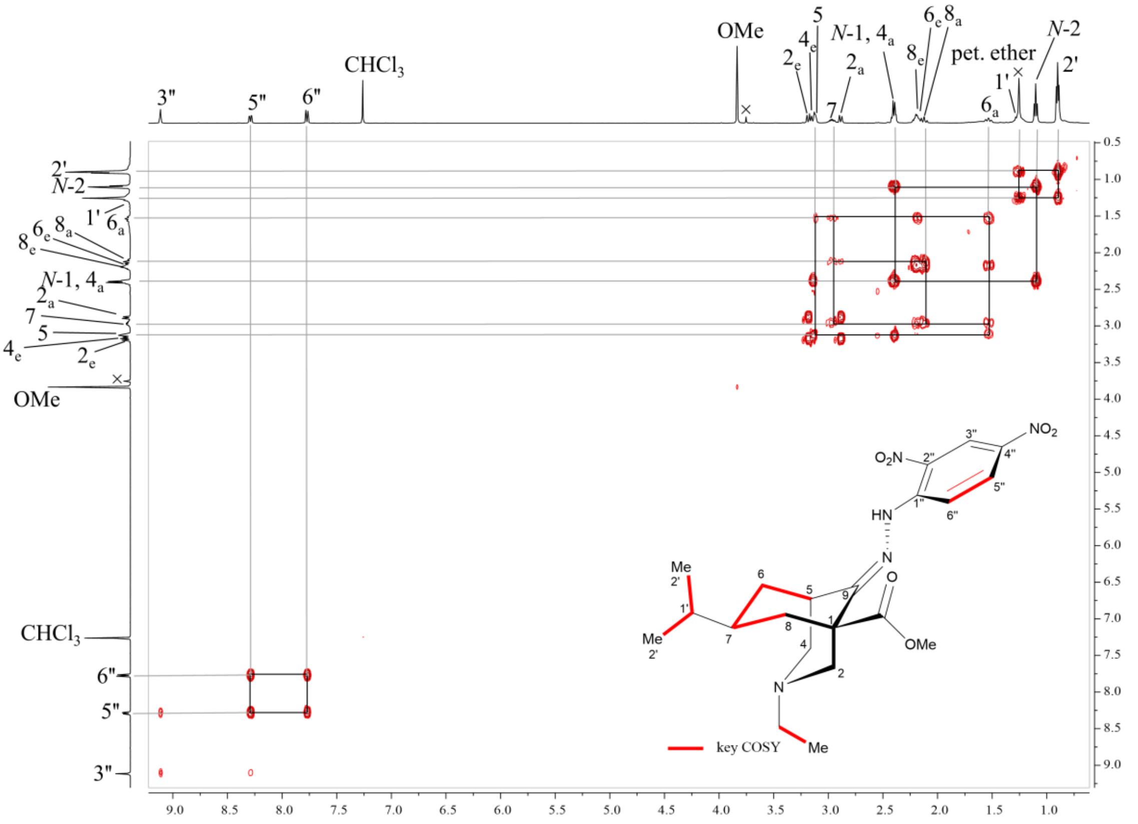

Figure S83. COSY spectrum of 7-iPr [3.3.1]azabicyclic DNP derivative (**19**) in $CDCl_3$

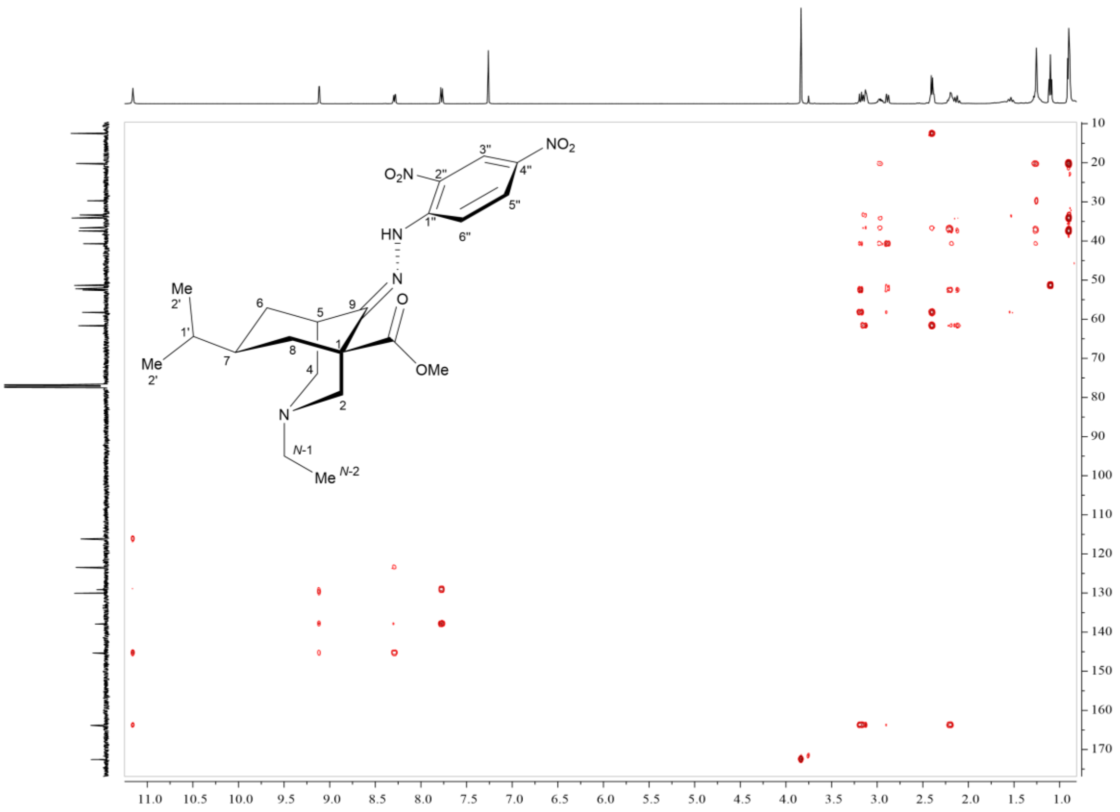

Figure S84. HMBC spectrum of 7-iPr [3.3.1]azabicyclic DNP derivative (**19**) in $CDCl_3$

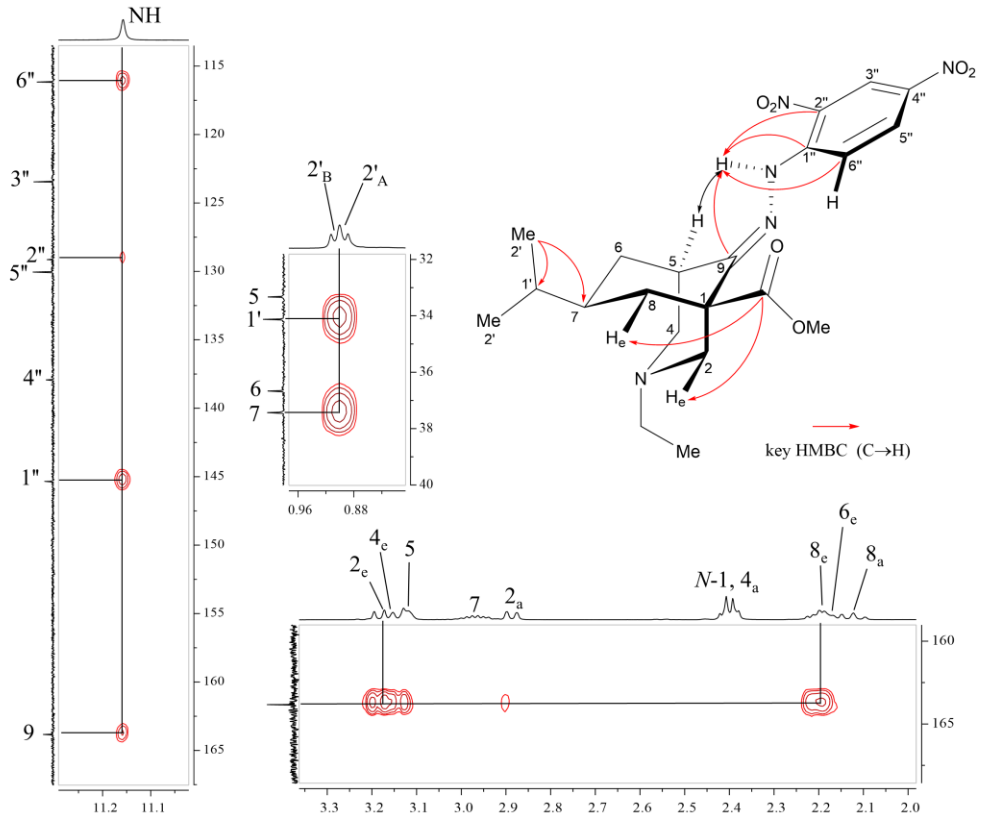

Figure S85. HMBC expansions of 7-iPr [3.3.1]azabicyclic DNP derivative (**19**) in $CDCl_3$

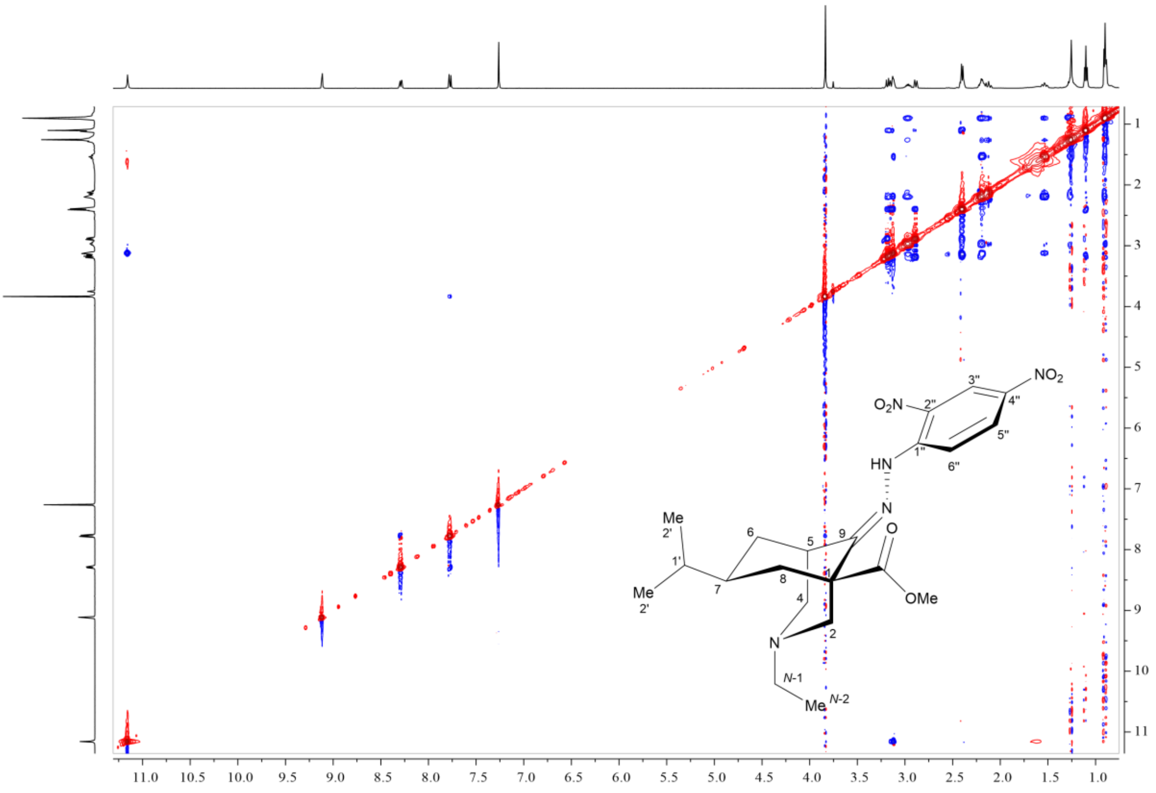

Figure S86. NOESY spectrum of 7-iPr [3.3.1]azabicyclic DNP derivative (**19**) in $CDCl_3$

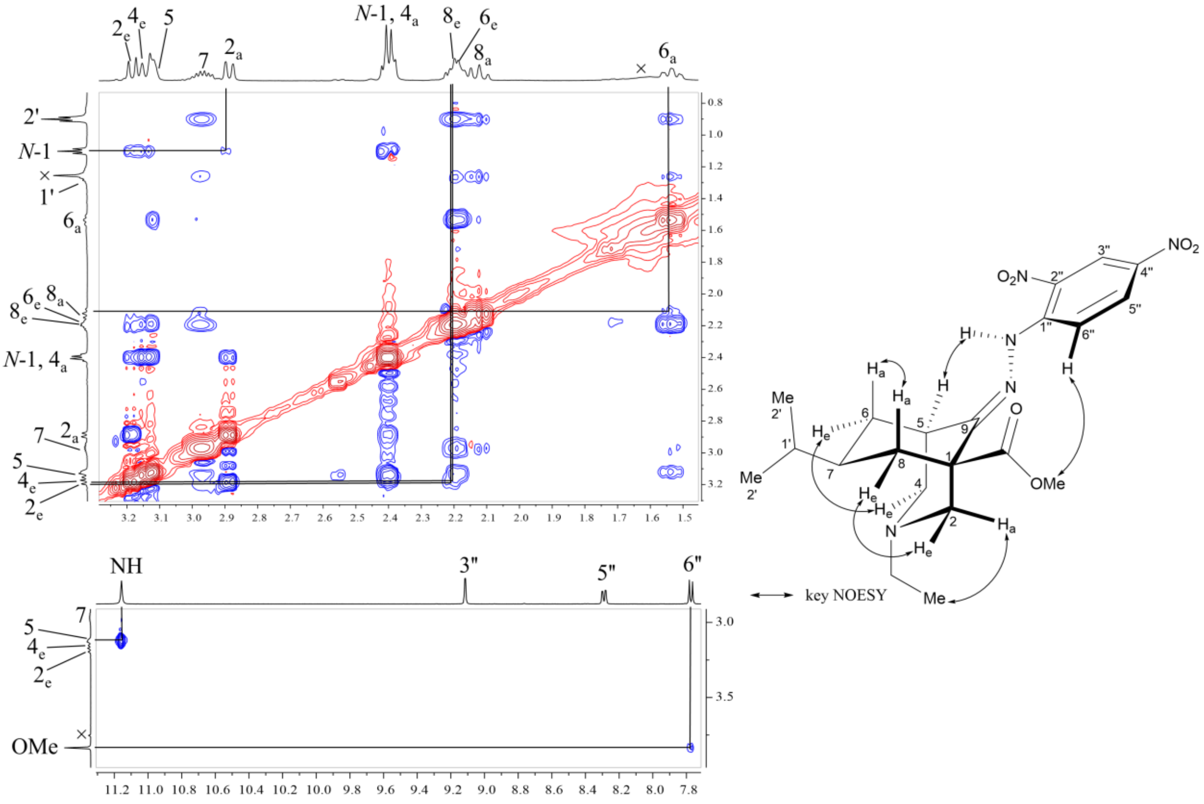

Figure S87. NOESY expansions of 7-iPr [3.3.1]azabicyclic DNP derivative (**19**) in $CDCl_3$

Methyl (*E*)-9-(2-(2,4-dinitrophenyl)hydrazinylidene)-3-ethyl-7-methyl-3-azabicyclo[3.3.1]nonane-1-carboxylate (**20**) and 1-(2,4-dinitrophenyl)-2-(propan-2-ylidene)hydrazine

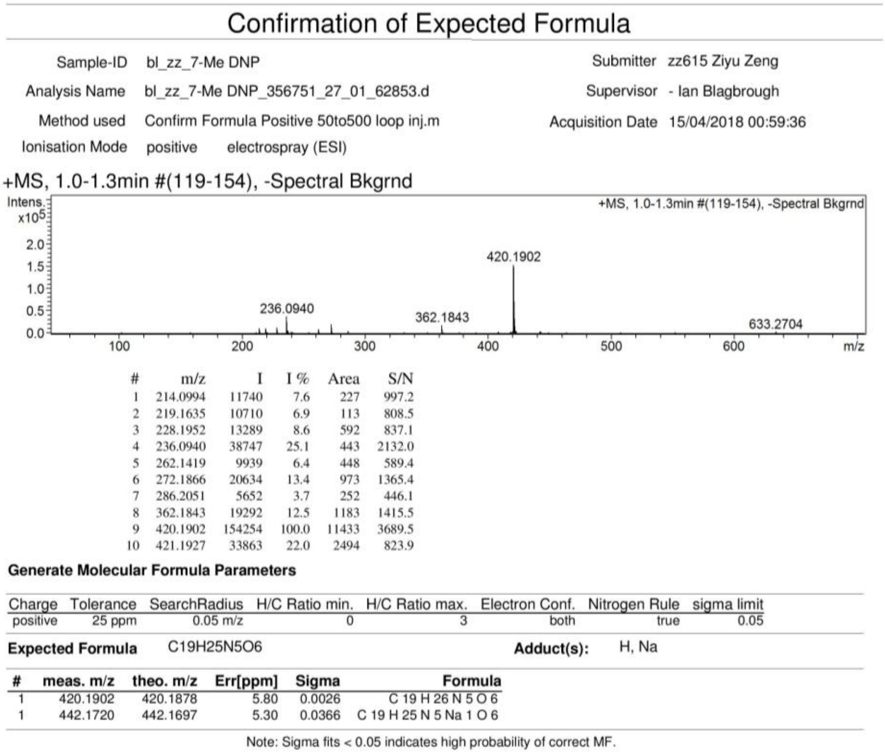

Confirmation of Expected Formula

Sample-ID	bl_zz_7-Me DNP	Submitter	zz615 Ziyu Zeng
Analysis Name	bl_zz_7-Me DNP_356751_27_01_62853.d	Supervisor	- Ian Blagbrough
Method used	Confirm Formula Positive 50to500 loop inj.m	Acquisition Date	15/04/2018 00:59:36
Ionisation Mode	positive electrospray (ESI)		

#	m/z	I	I %	Area	S/N
1	214.0994	11740	7.6	227	997.2
2	219.1635	10710	6.9	113	808.5
3	228.1952	13289	8.6	592	837.1
4	236.0940	38747	25.1	443	2132.0
5	262.1419	9939	6.4	448	589.4
6	272.1866	20634	13.4	973	1365.4
7	286.2051	5652	3.7	252	446.1
8	362.1843	19292	12.5	1183	1415.5
9	420.1902	154254	100.0	11433	3689.5
10	421.1927	33863	22.0	2494	823.9

Generate Molecular Formula Parameters

Charge	Tolerance	SearchRadius	H/C Ratio min.	H/C Ratio max.	Electron Conf.	Nitrogen Rule	sigma limit
positive	25 ppm	0.05 m/z	0	3	both	true	0.05

Expected Formula C19H25N5O6 **Adduct(s):** H, Na

#	meas. m/z	theo. m/z	Err[ppm]	Sigma	Formula
1	420.1902	420.1878	5.80	0.0026	C 19 H 26 N 5 O 6
1	442.1720	442.1697	5.30	0.0366	C 19 H 25 N 5 Na 1 O 6

Note: Sigma fits < 0.05 indicates high probability of correct MF.

Figure S88. MS data of 7-Me [3.3.1]azabicyclic DNP derivative (**20**, methyl ester)

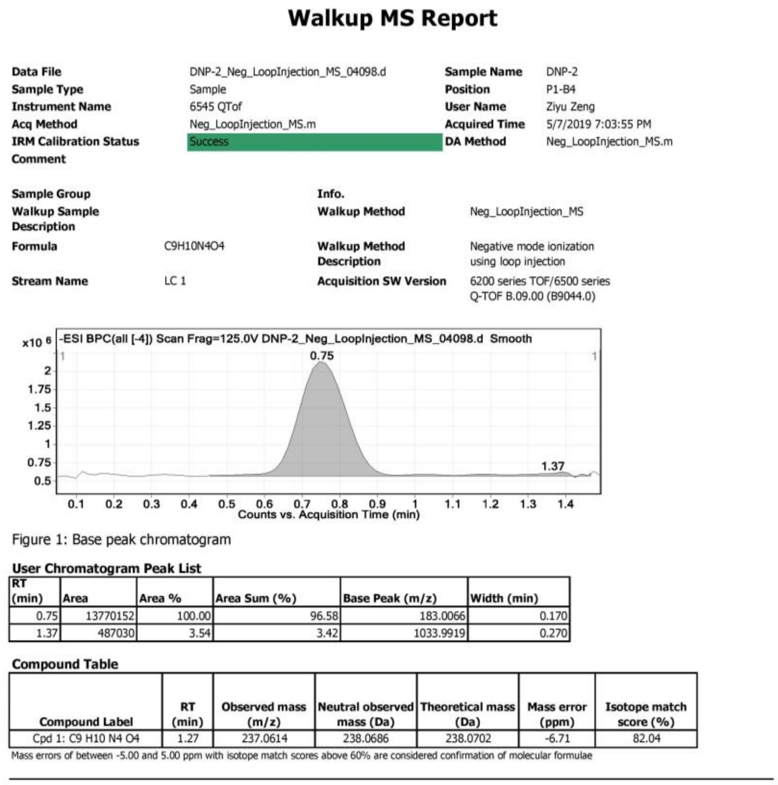

Walkup MS Report

Data File	DNP-2_Neg_LoopInjection_MS_04098.d	Sample Name	DNP-2
Sample Type	Sample	Position	P1-B4
Instrument Name	6545 QTof	User Name	Ziyu Zeng
Acq Method	Neg_LoopInjection_MS.m	Acquired Time	5/7/2019 7:03:55 PM
IRM Calibration Status	Success	DA Method	Neg_LoopInjection_MS.m
Comment			

Sample Group		Info.	
Walkup Sample Description		Walkup Method	Neg_LoopInjection_MS
Formula	C9H10N4O4	Walkup Method Description	Negative mode ionization using loop injection
Stream Name	LC 1	Acquisition SW Version	6200 series TOF/6500 series Q-TOF B.09.00 (B9044.0)

Figure 1: Base peak chromatogram

User Chromatogram Peak List

RT (min)	Area	Area %	Area Sum (%)	Base Peak (m/z)	Width (min)
0.75	13770152	100.00	96.58	183.0066	0.170
1.37	487030	3.54	3.42	1033.9919	0.270

Compound Table

Compound Label	RT (min)	Observed mass (m/z)	Neutral observed mass (Da)	Theoretical mass (Da)	Mass error (ppm)	Isotope match score (%)
Cpd 1: C9 H10 N4 O4	1.27	237.0614	238.0686	238.0702	-6.71	82.04

Mass errors of between -5.00 and 5.00 ppm with isotope match scores above 60% are considered confirmation of molecular formulae

Figure S89. MS data of 1-(2,4-dinitrophenyl)-2-(propan-2-ylidene)hydrazine

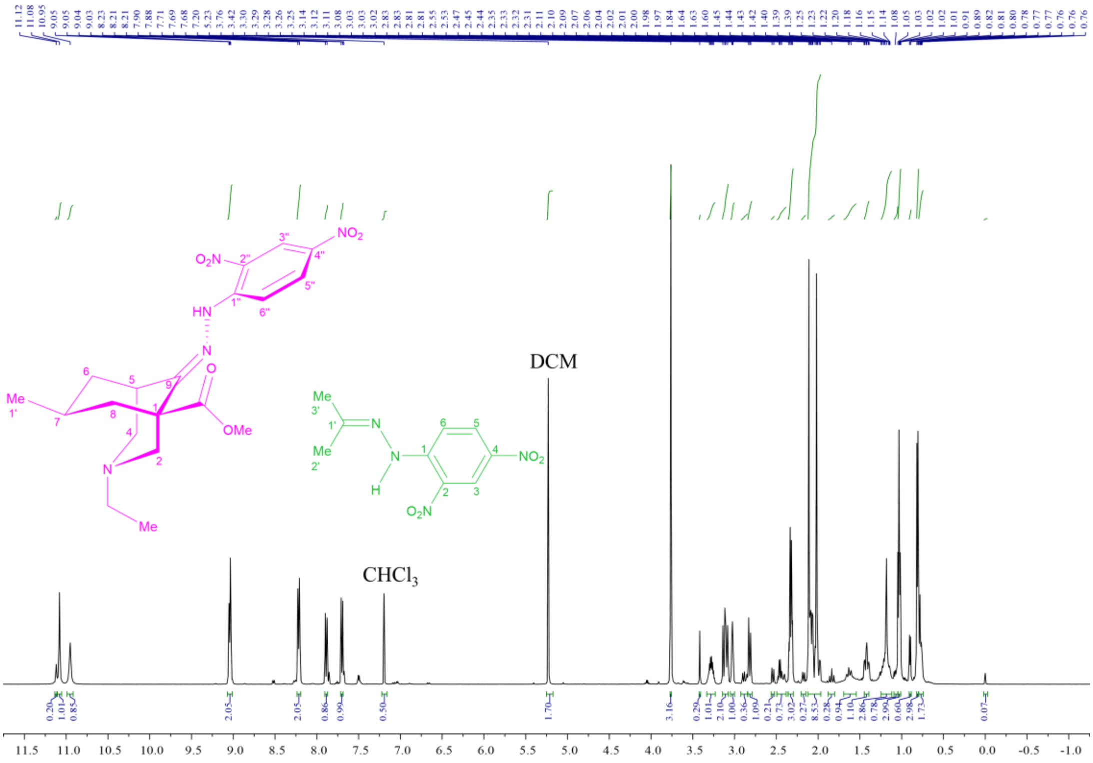

Figure S90. ^{1}H NMR spectrum of 7-Me [3.3.1]azabicyclic DNP derivative (**20**, methyl ester) and 1-(2,4-dinitrophenyl)-2-(propan-2-ylidene)hydrazine in $CDCl_3$ (residual DCM and petroleum ether are displayed)[1]

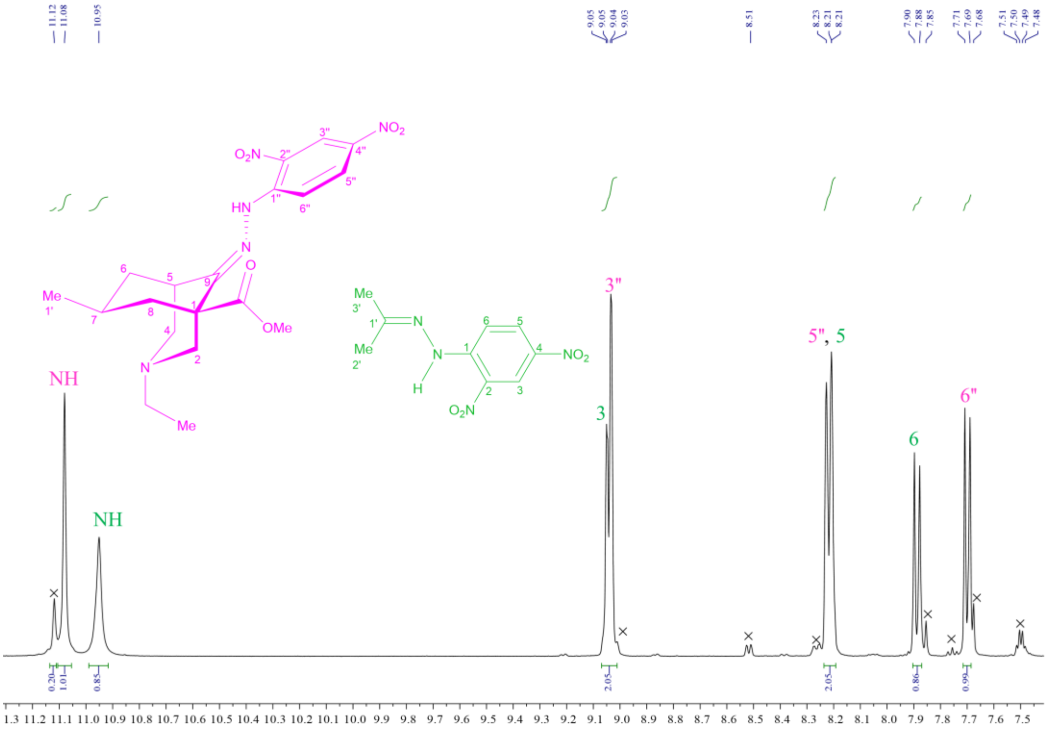

Figure S91. ^{1}H NMR expansion of 7-Me [3.3.1]azabicyclic DNP derivative (**20**, methyl ester) and 1-(2,4-dinitrophenyl)-2-(propan-2-ylidene)hydrazine in $CDCl_3$ with assignments

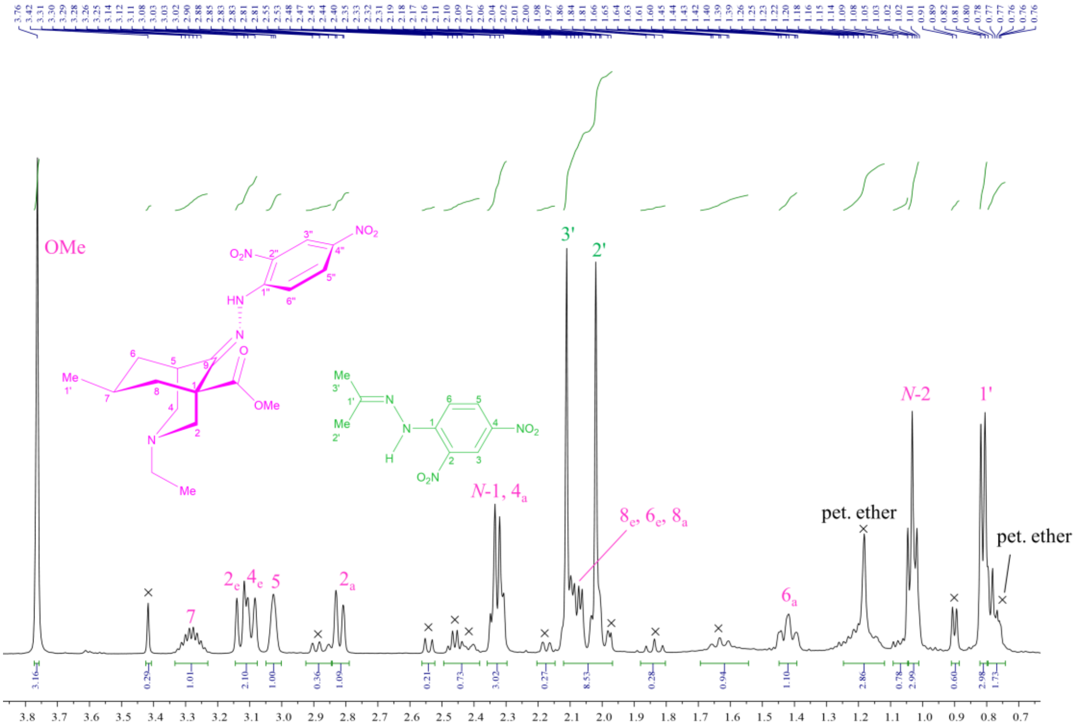

Figure S92. ^{1}H NMR expansion of 7-Me [3.3.1]azabicyclic DNP derivative (**20**, methyl ester) and 1-(2,4-dinitrophenyl)-2-(propan-2-ylidene)hydrazine in $CDCl_3$ with assingments (residual petroleum ether is displayed)[1]

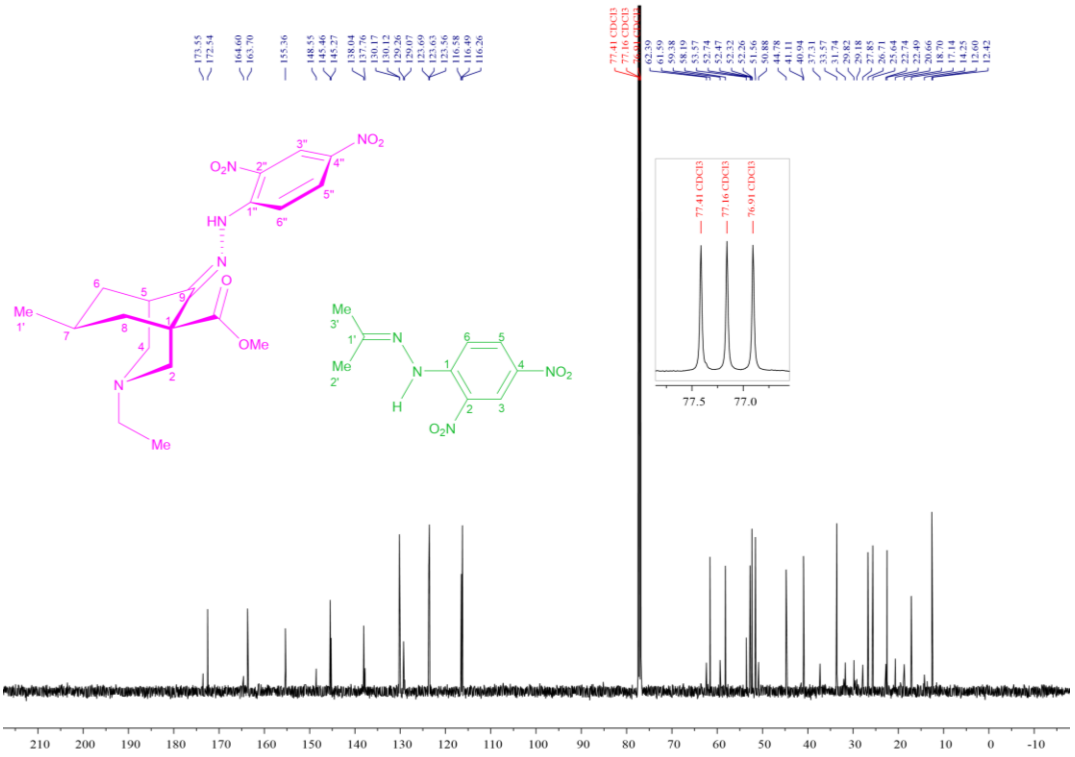

Figure S93. ^{13}C NMR spectrum of 7-Me [3.3.1]azabicyclic DNP derivative (**20**, methyl ester) and 1-(2,4-dinitrophenyl)-2-(propan-2-ylidene)hydrazine in $CDCl_3$

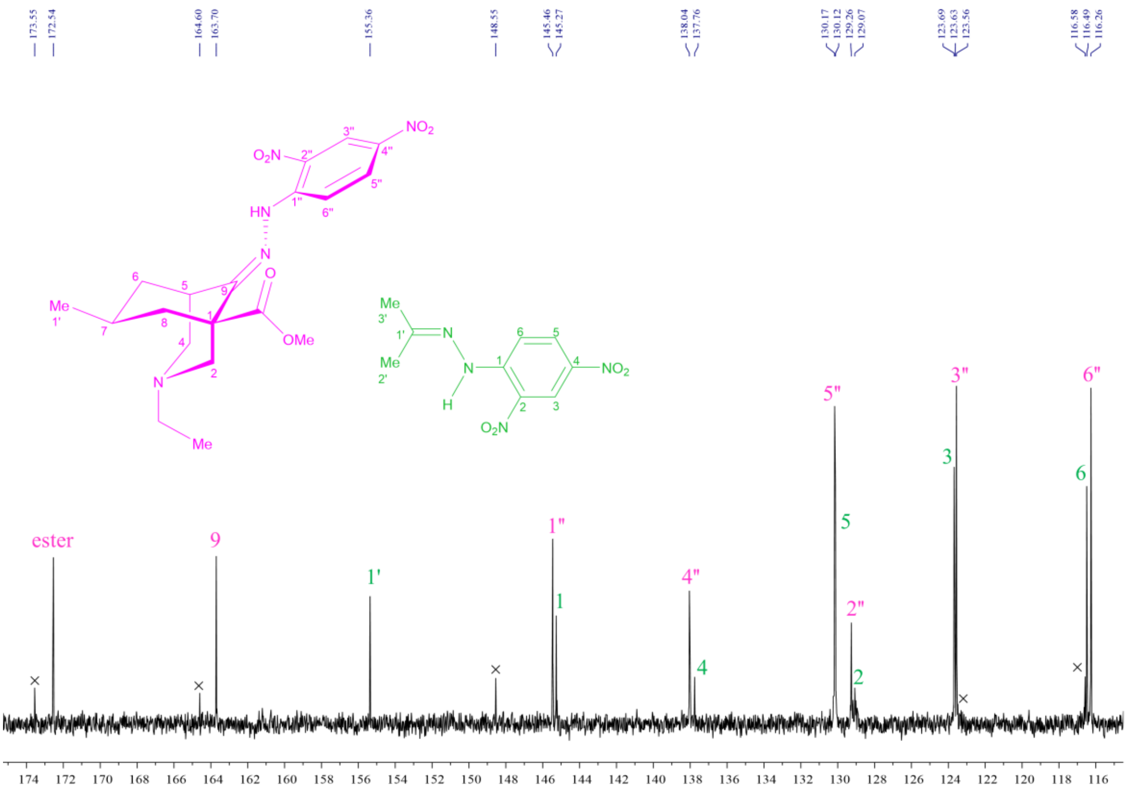

Figure S94. ^{13}C NMR espansion of 7-Me [3.3.1]azabicyclic DNP derivative (**20**, methyl ester) and 1-(2,4-dinitrophenyl)-2-(propan-2-ylidene)hydrazine in $CDCl_3$

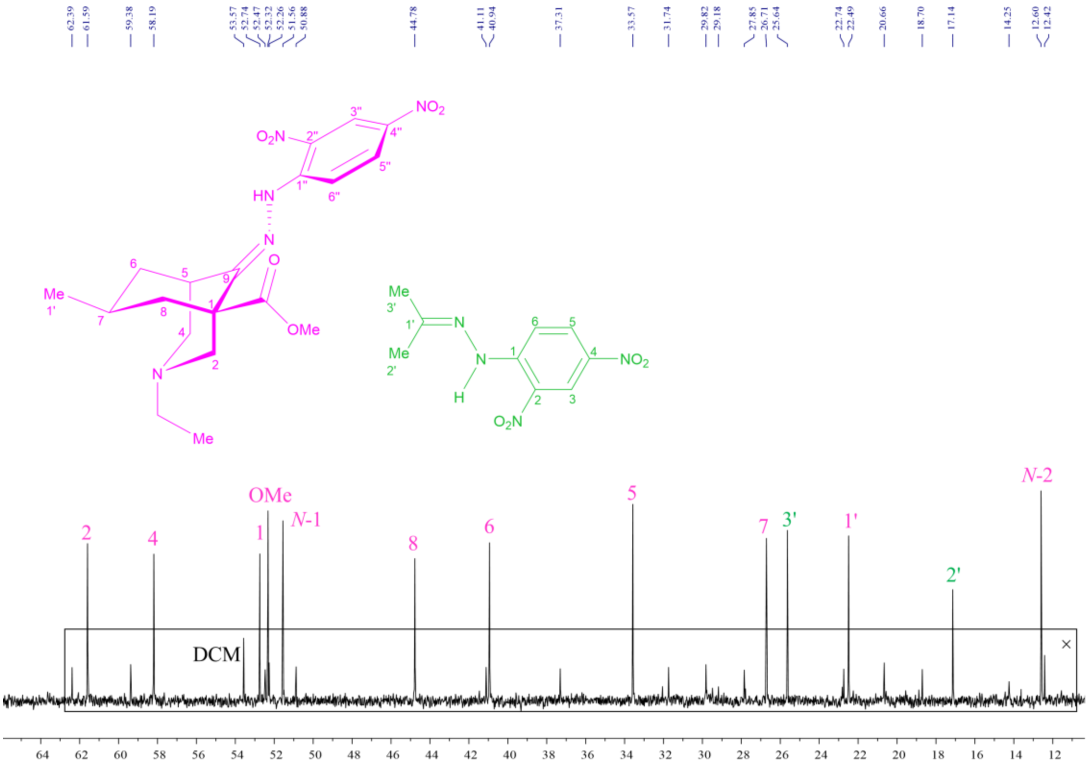

Figure S95. ^{13}C NMR espansion of 7-Me [3.3.1]azabicyclic DNP derivative (**20**, methyl ester) and 1-(2,4-dinitrophenyl)-2-(propan-2-ylidene)hydrazine in $CDCl_3$ (residual DCM is shown)[1]

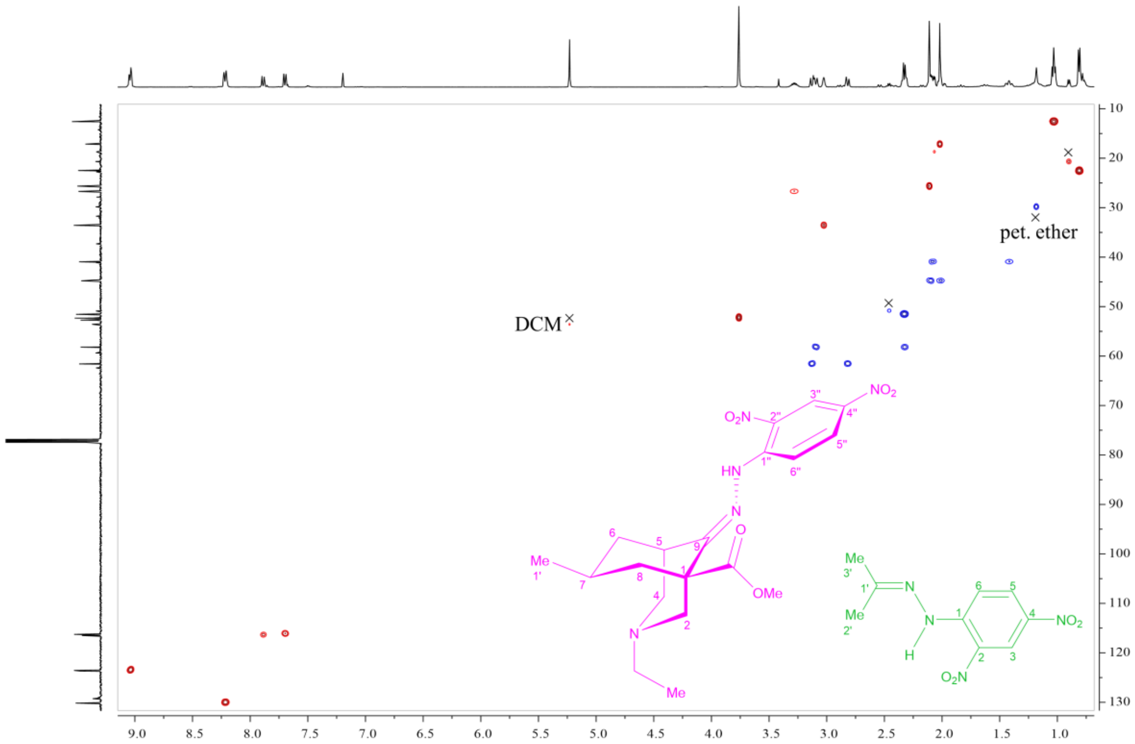

Figure S96. HSQC spectrum of 7-Me [3.3.1]azabicyclic DNP derivative (**20**, methyl ester) and 1-(2,4-dinitrophenyl)-2-(propan-2-ylidene)hydrazine in $CDCl_3$

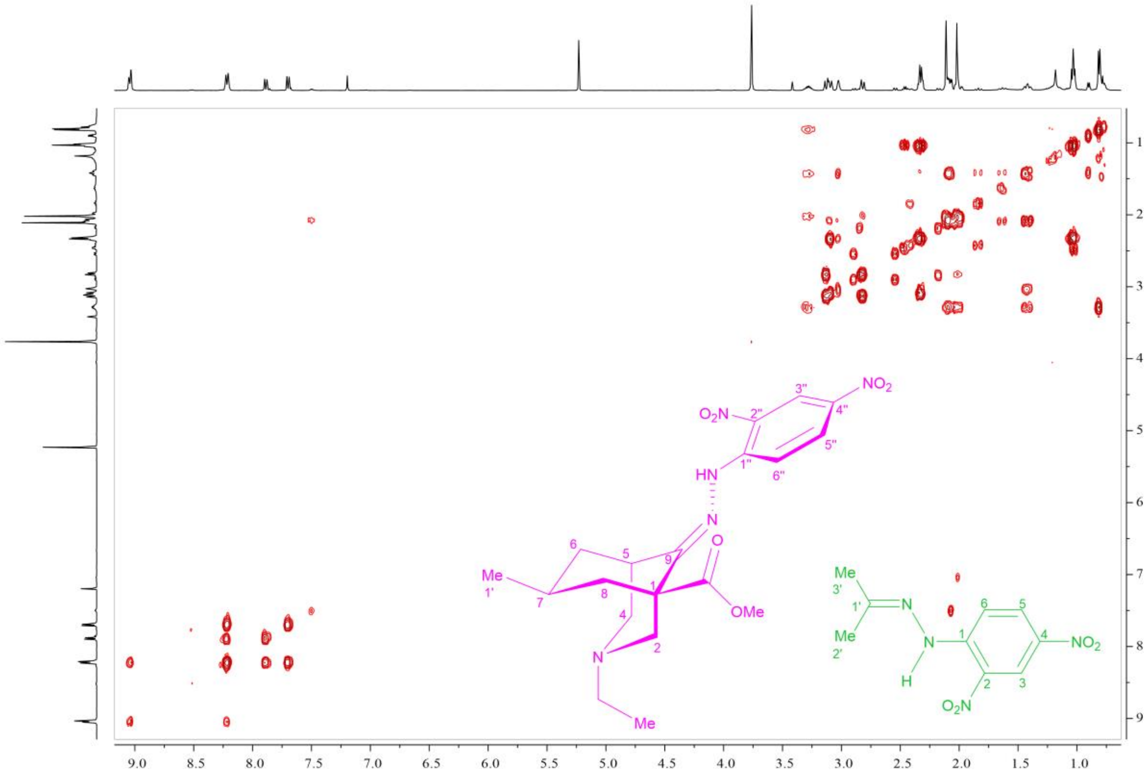

Figure S97. COSY spectrum of 7-Me [3.3.1]azabicyclic DNP derivative (**20**, methyl ester) and 1-(2,4-dinitrophenyl)-2-(propan-2-ylidene)hydrazine in $CDCl_3$

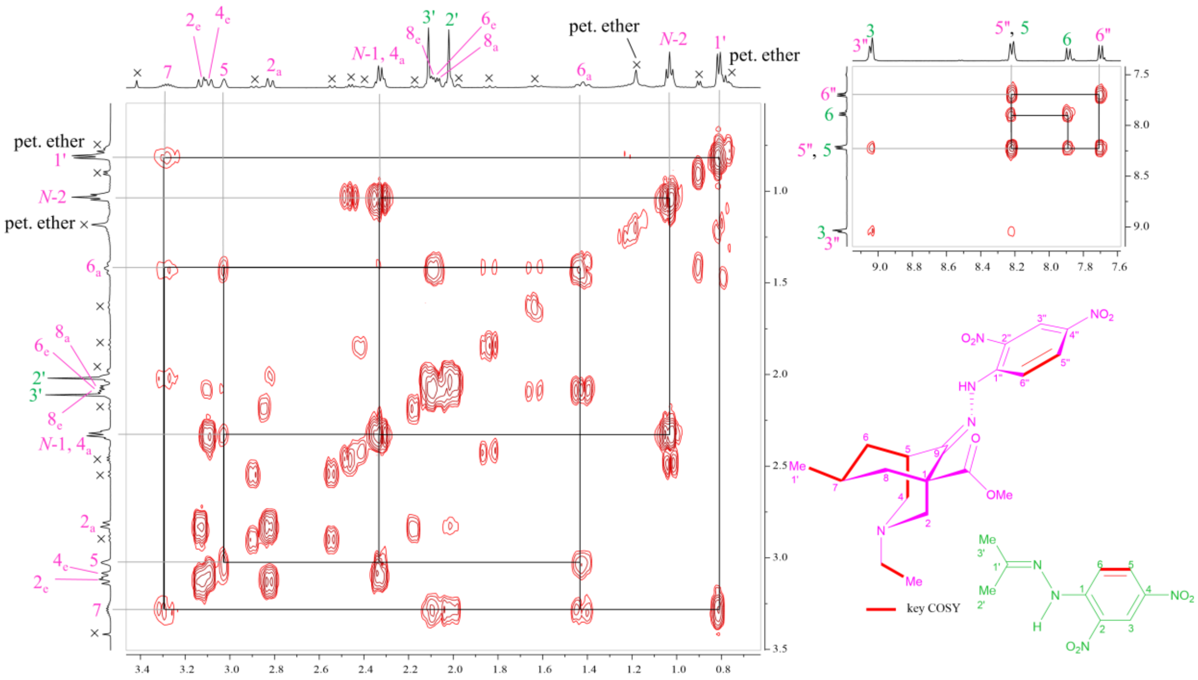

Figure S98. COSY expansions of 7-Me [3.3.1]azabicyclic DNP derivative (**20**, methyl ester) and 1-(2,4-dinitrophenyl)-2-(propan-2-ylidene)hydrazine in $CDCl_3$

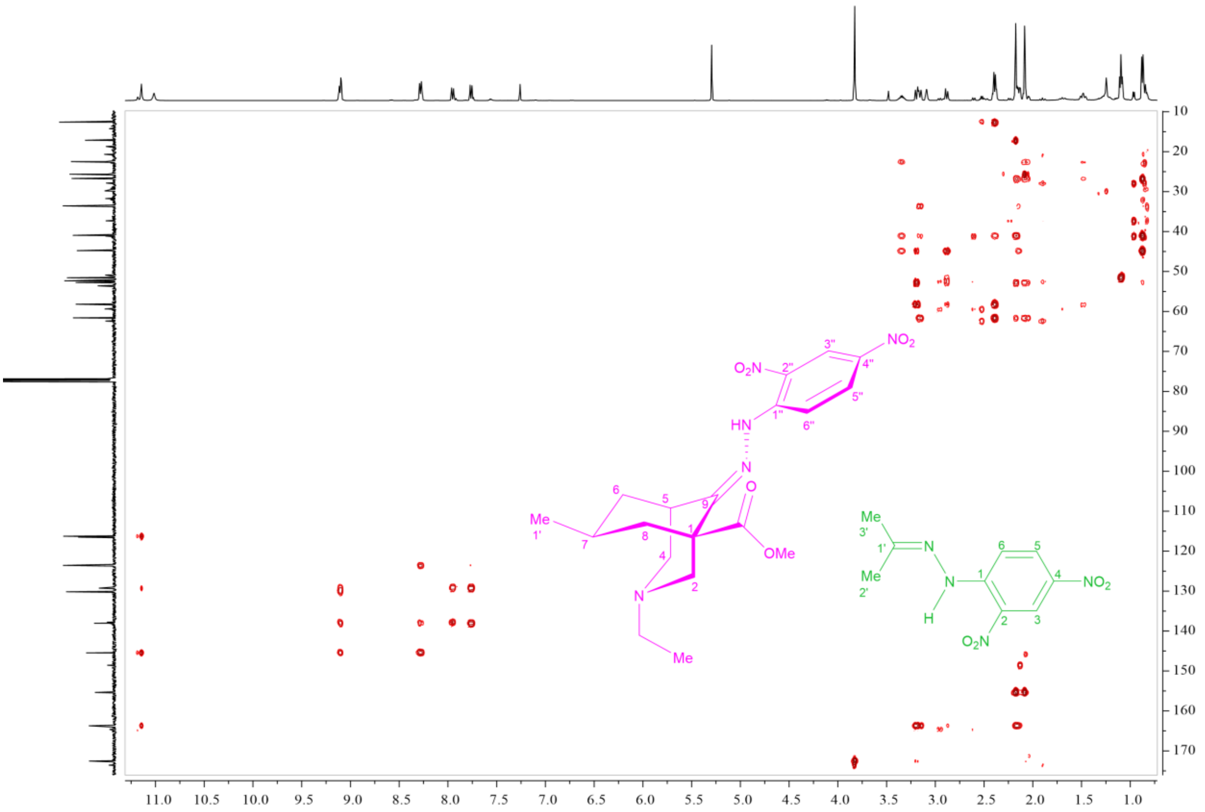

Figure S99. HMBC spectrum of 7-Me [3.3.1]azabicyclic DNP derivative (**20**, methyl ester) and 1-(2,4-dinitrophenyl)-2-(propan-2-ylidene)hydrazine in $CDCl_3$

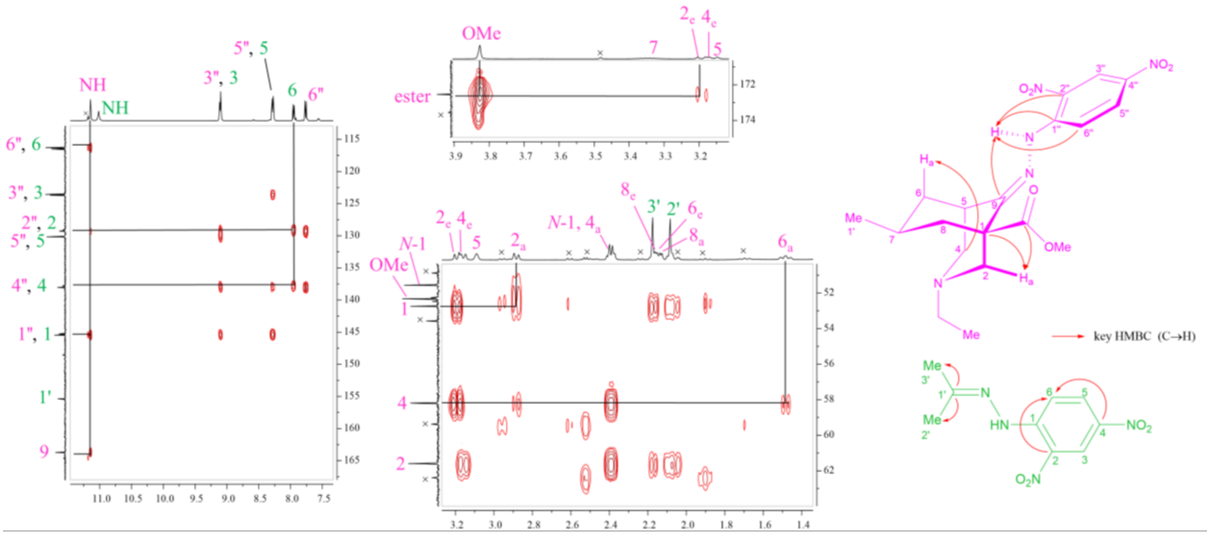

Figure S100. HMBC expansion of 7-Me [3.3.1]azabicyclic DNP derivative (**20**, methyl ester) and 1-(2,4-dinitrophenyl)-2-(propan-2-ylidene)hydrazine in $CDCl_3$

The major components are identified. The remaining signals (in both ^{1}H and ^{13}C) are due to the two conformational isomers of compound (**20**) rather than due to impurities as TLC of the purified product showed only a single spot (UV, λ = 254 nm; staining with iodine vapour, *p*-anisaldehyde solution and Dragendorff's reagent).

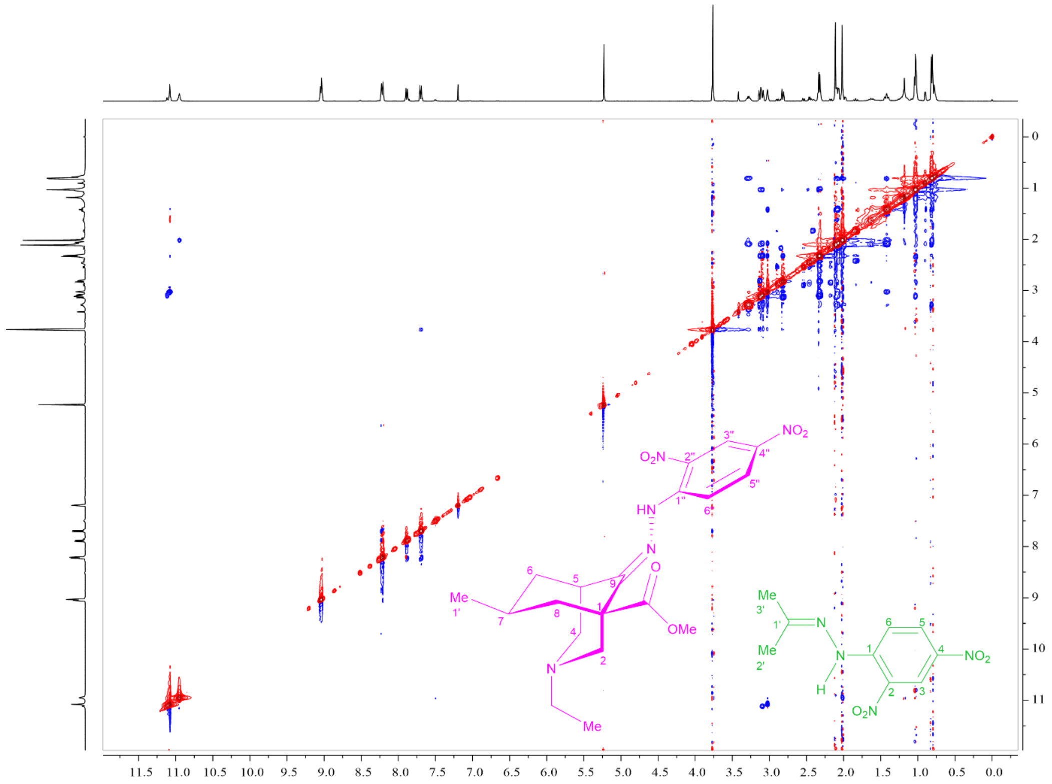

Figure S101. NOESY spectrum of 7-Me [3.3.1]azabicyclic DNP derivative (**20**, methyl ester) and 1-(2,4-dinitrophenyl)-2-(propan-2-ylidene)hydrazine in $CDCl_3$

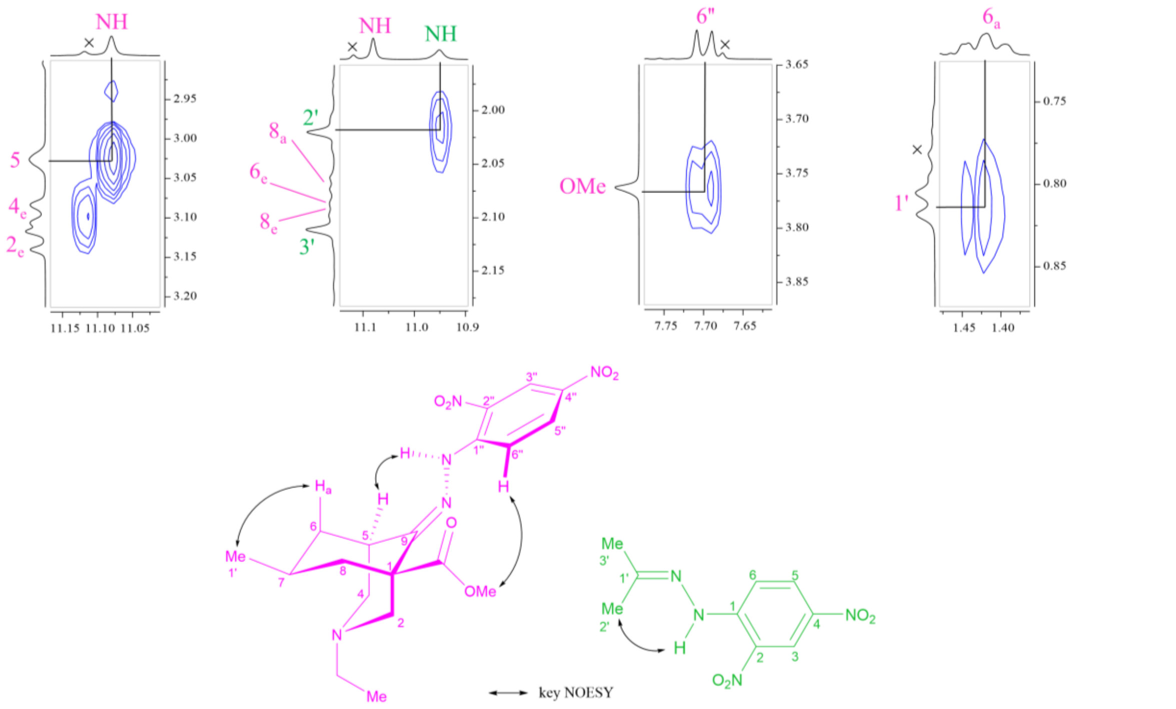

Figure S102. NOESY expansions of 7-Me [3.3.1]azabicyclic DNP derivative (**20**, methyl ester) and 1-(2,4-dinitrophenyl)-2-(propan-2-ylidene)hydrazine in $CDCl_3$

Methyl 8-ethyl-10-oxo-8-azabicyclo[4.3.1]decane-1-carboxylate (**23**)

Confirmation of Expected Formula

Sample-ID	bl_zz_7-AE	Submitter	zz615 Ziyu Zeng
Analysis Name	bl_zz_7-AE_352512_36_01_58030.d	Supervisor	- Ian Blagbrough
Method used	Confirm Formula Positive 50to500 loop inj.m	Acquisition Date	24/07/2017 12:47:43
Ionisation Mode	positive electrospray (ESI)		

+MS, 1.0-1.3min #(119-154), -Spectral Bkgrnd

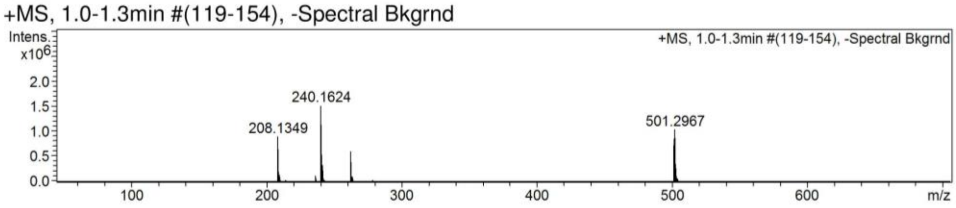

#	m/z	I	I %	Area	S/N
1	208.1349	888926	58.7	36961	7011.2
2	209.1373	120611	8.0	4662	922.4
3	236.0750	114015	7.5	3344	482.4
4	240.1624	1515422	100.0	95810	6005.6
5	241.1643	320997	21.2	13915	1252.5
6	262.1428	600891	39.7	29964	3858.9
7	263.1457	91715	6.1	4150	611.1
8	501.2967	1031520	68.1	105159	8869.3
9	502.2987	317968	21.0	30745	2689.3
10	503.3011	57671	3.8	5068	479.7

Generate Molecular Formula Parameters

Charge	Tolerance	SearchRadius	H/C Ratio min.	H/C Ratio max.	Electron Conf.	Nitrogen Rule	sigma limit
positive	20 ppm	0.05 m/z	0	3	both	true	0.05

Expected Formula C13H21NO3 **Adduct(s):** H, Na

#	meas. m/z	theo. m/z	Err[ppm]	Sigma	Formula
1	240.1624	240.1594	12.50	0.0391	C 13 H 22 N 1 O 3
1	262.1428	262.1414	5.50	0.0048	C 13 H 21 N 1 Na 1 O 3

Note: Sigma fits < 0.05 indicates high probability of correct MF, and mass accuracy of 5ppm or better is generally acceptable for publication

Figure S103. MS data of [4.3.1]azabicycle (**23**)

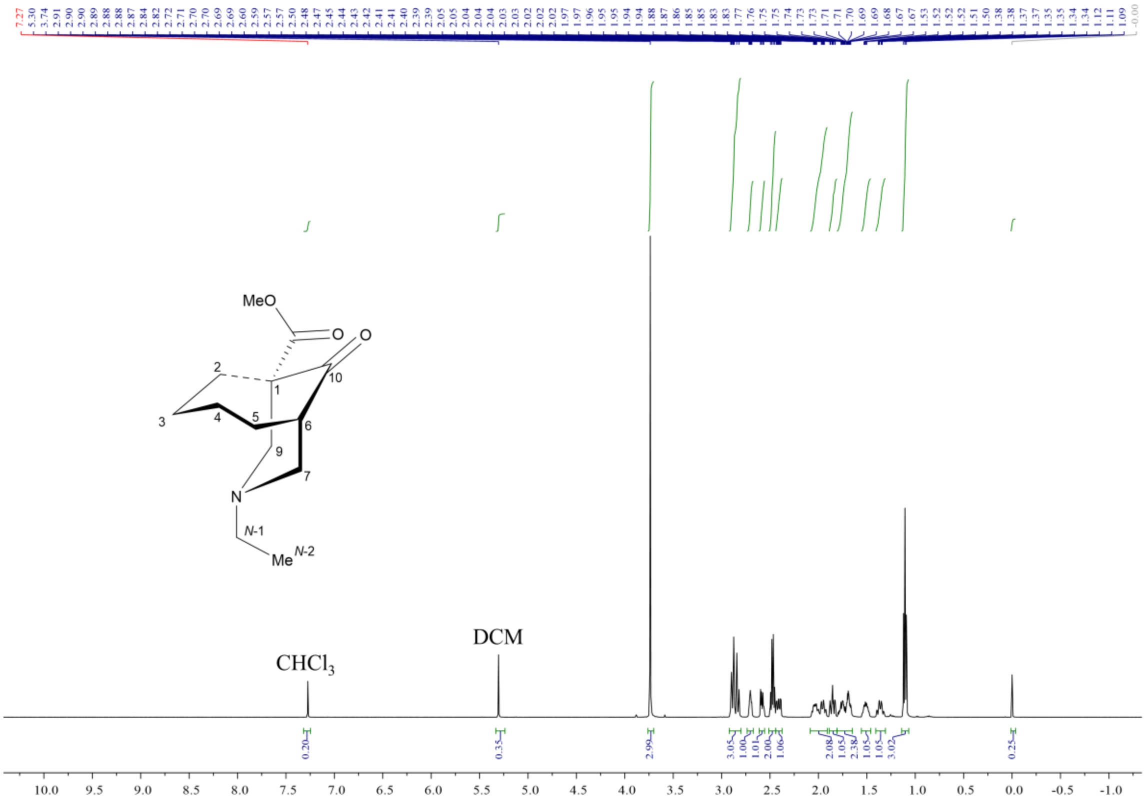

Figure S104. ^{1}H NMR spectrum of [4.3.1]azabicycle (**23**) in $CDCl_3$ (residual DCM is shown)[1]

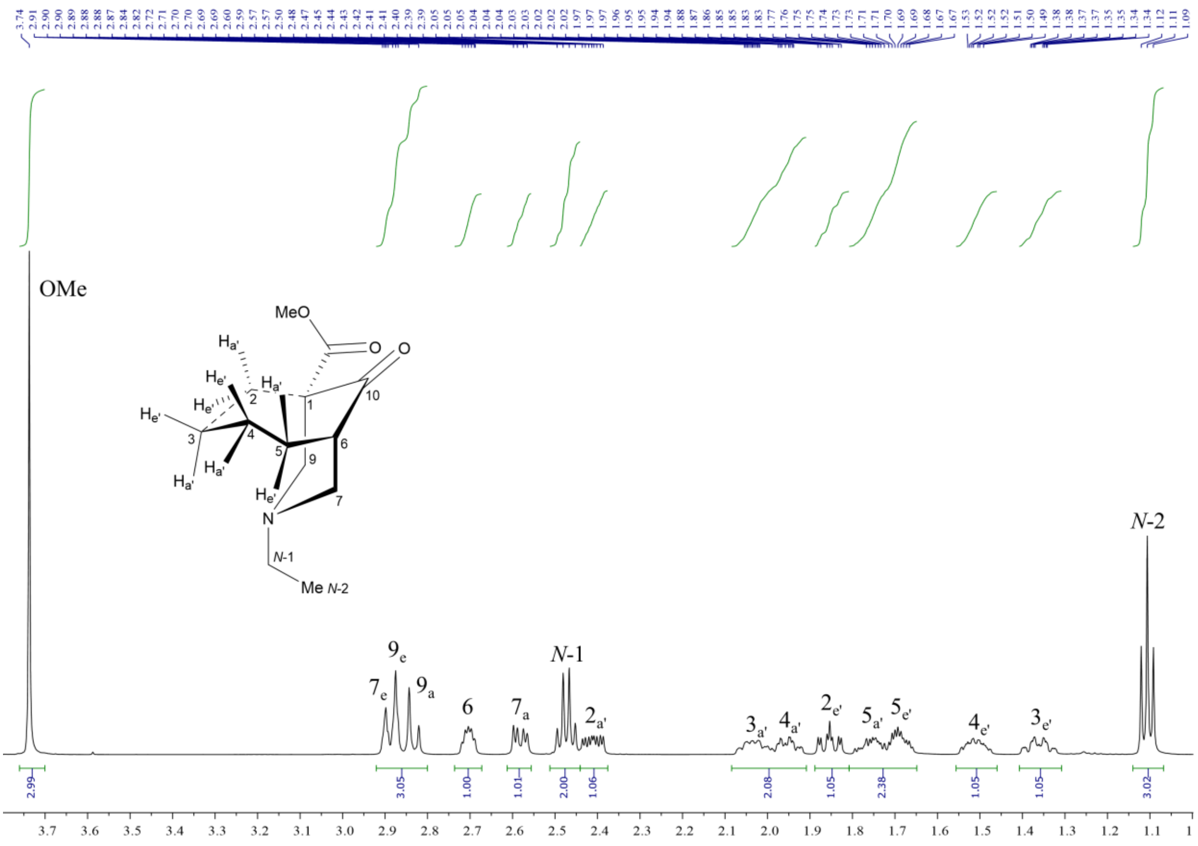

Figure S105. ^{1}H NMR expansion of [4.3.1]azabicycle (**23**) in $CDCl_3$ with assignments

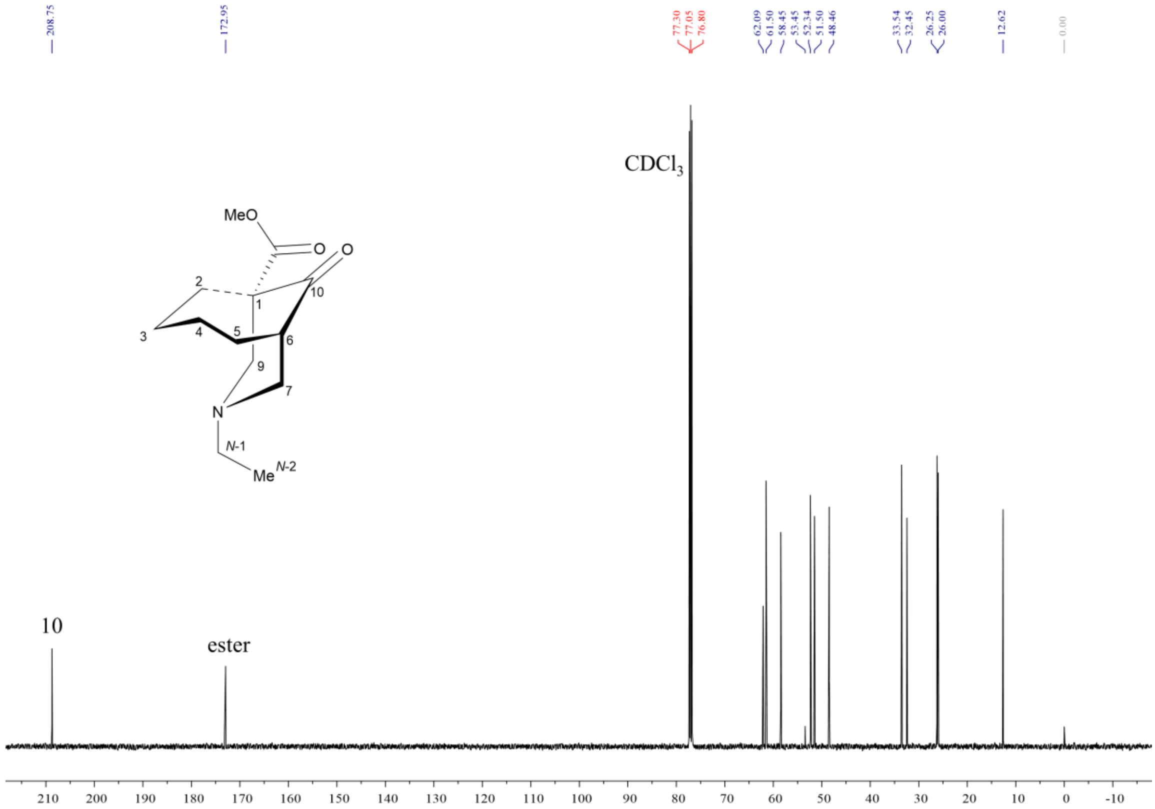

Figure S106. ^{13}C NMR spectrum of [4.3.1]azabicycle (**23**) in $CDCl_3$

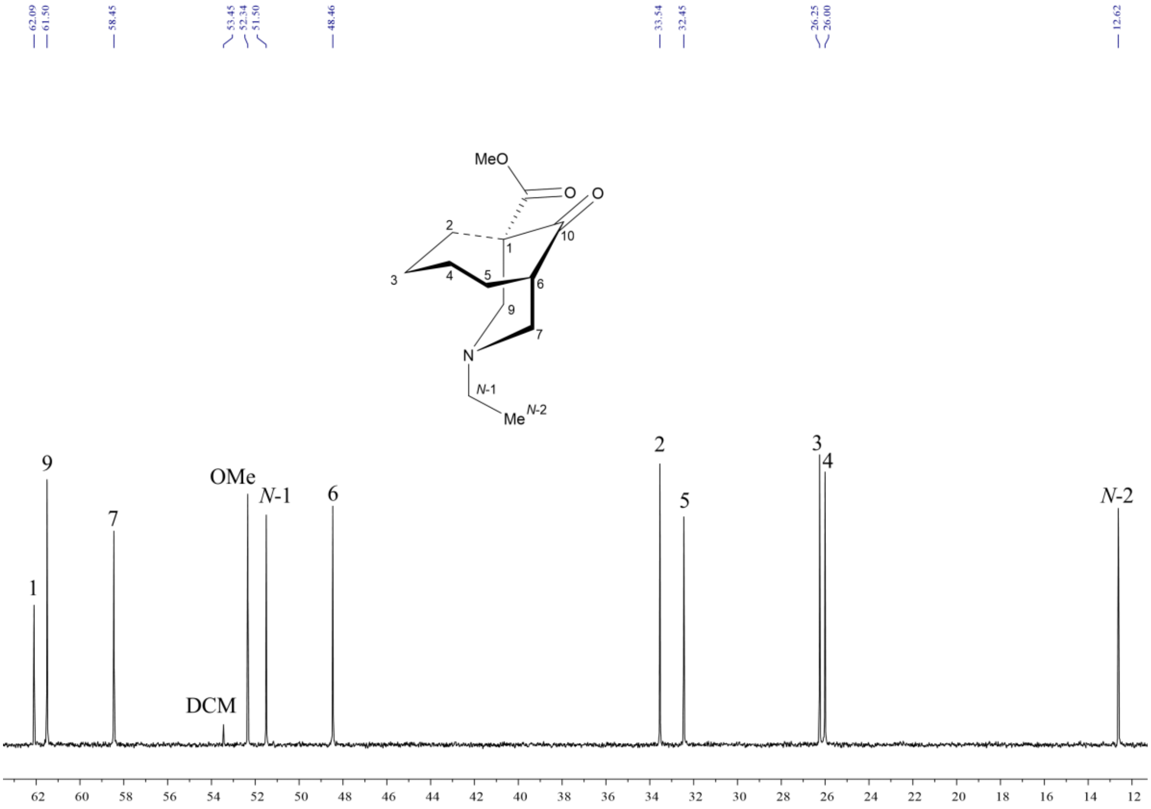

Figure S107. ^{13}C NMR expansion of azabicycle (**23**) in $CDCl_3$ with assignments (residual DCM is shown)[1]

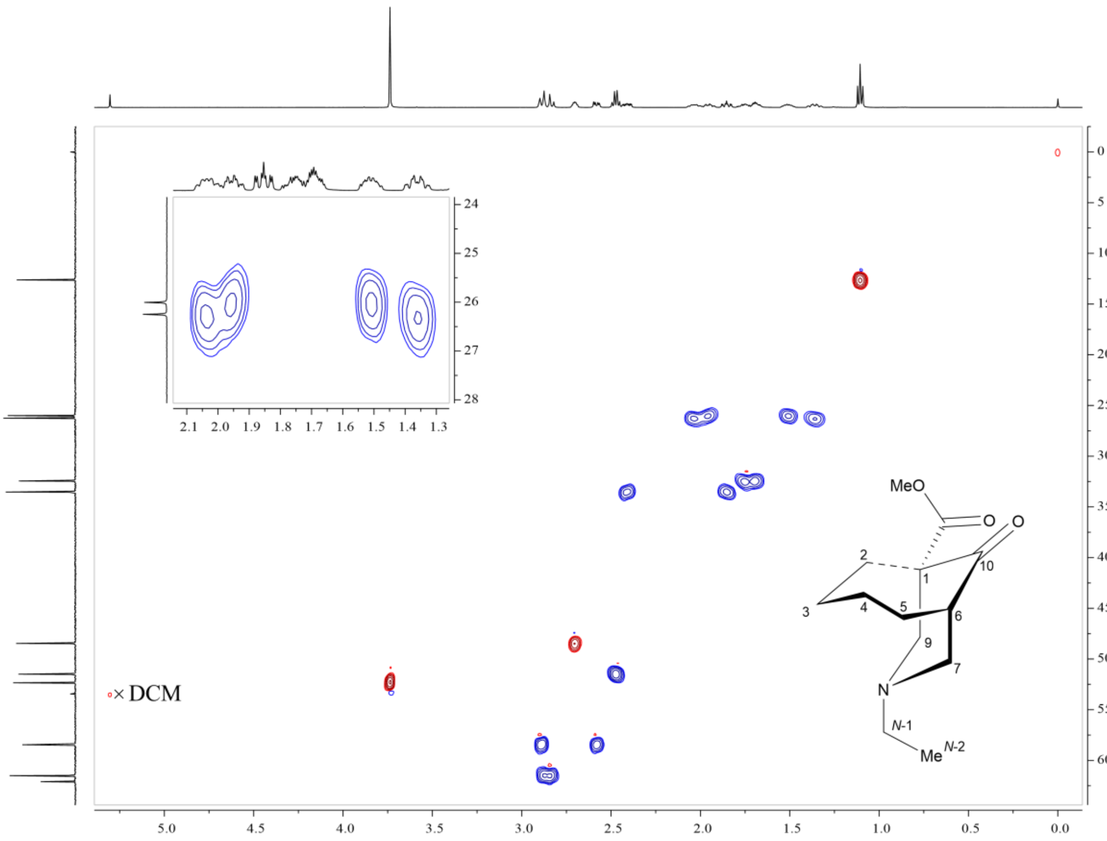

Figure S108. HSQC spectrum of [4.3.1]azabicycle (**23**) in $CDCl_3$

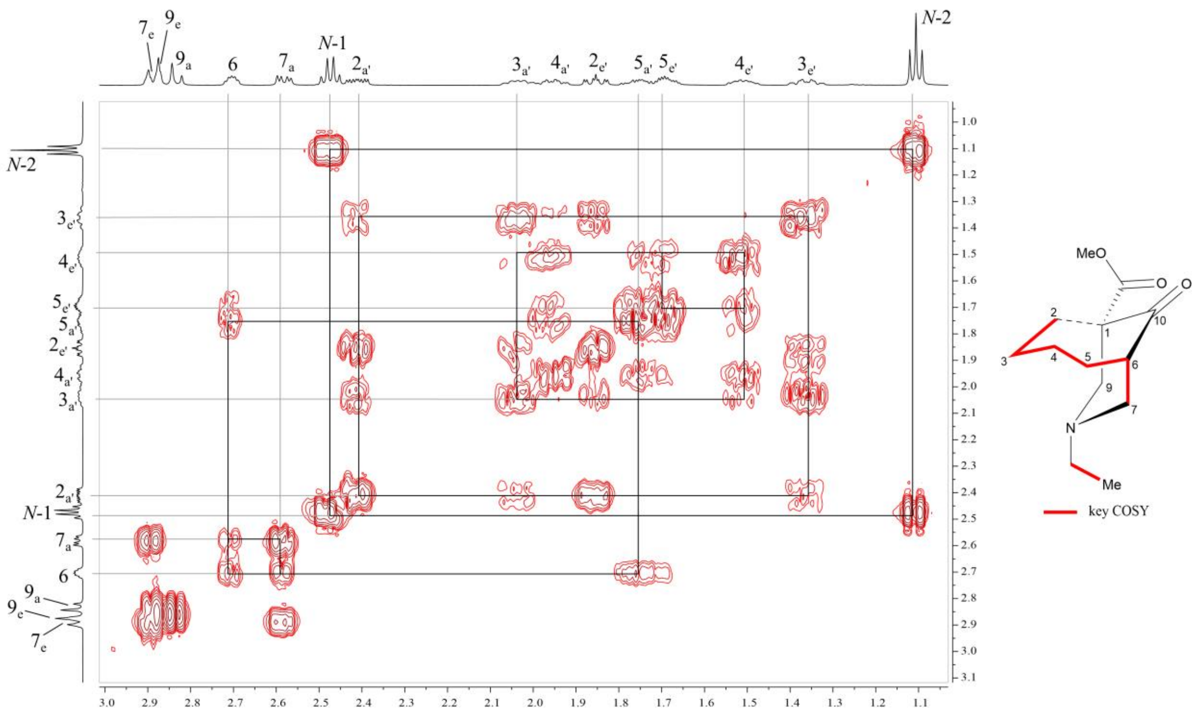

Figure S109. COSY spectrum of [4.3.1]azabicycle (**23**) in $CDCl_3$

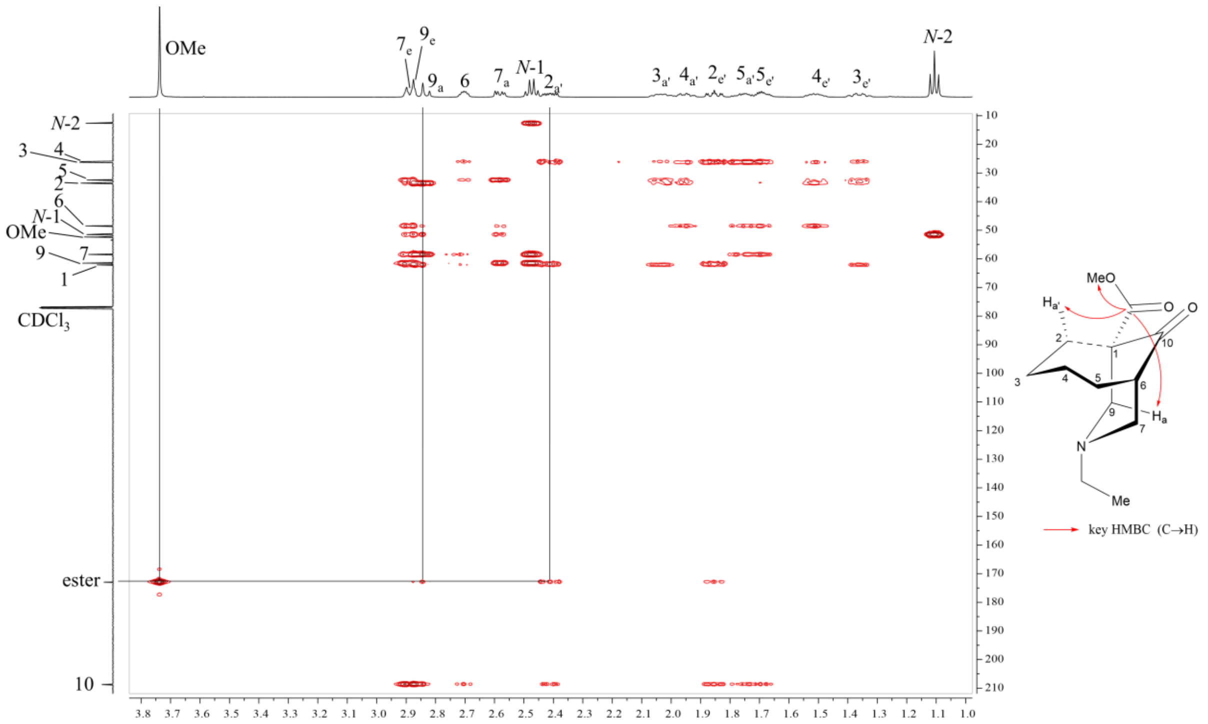

Figure S110. HMBC spectrum of [4.3.1]azabicycle (**23**) in $CDCl_3$

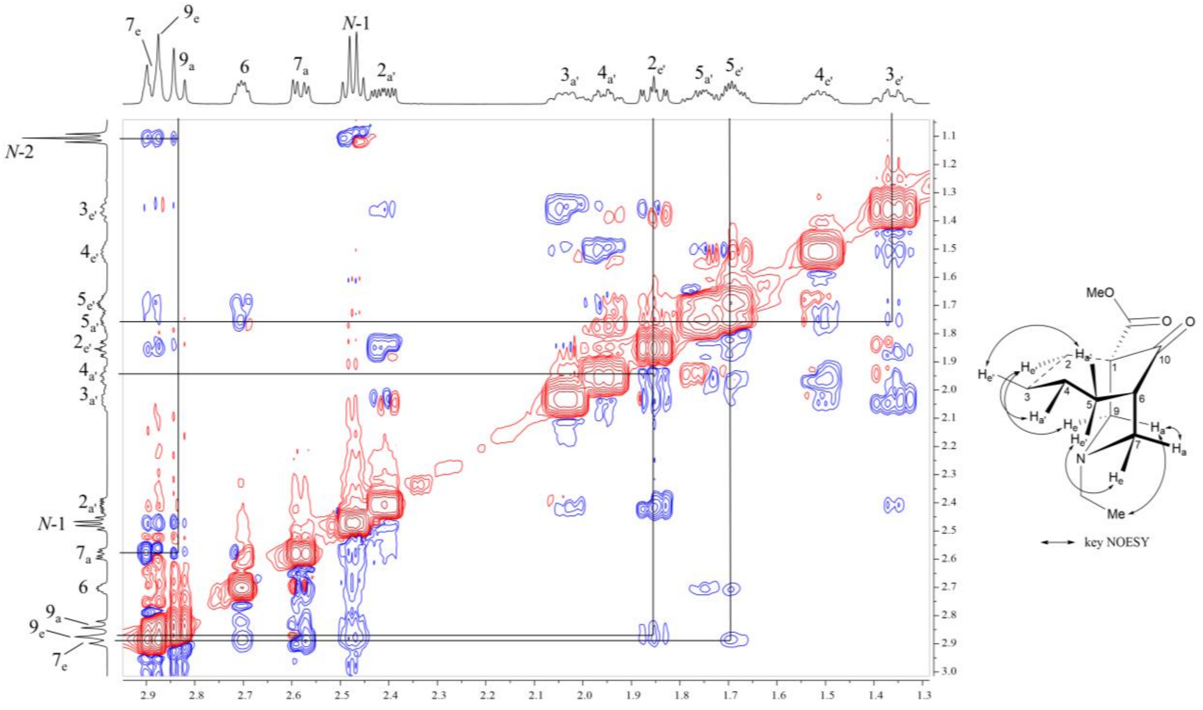

Figure S111. NOESY spectrum of [4.3.1]azabicycle (**23**) in $CDCl_3$

Methyl (*E*)-10-(2-(2,4-dinitrophenyl)hydrazinylidene)-8-ethyl-8-azabicyclo[4.3.1]decane-1-carboxylate (**24**)

Confirmation of Expected Formula

Sample-ID	bl_zz_8-9	Submitter	zz615 Ziyu Zeng
Analysis Name	bl_zz_8-9_353634_87_01_59304.d	Supervisor	- Ian Blagbrough
Method used	Confirm Formula Positive 50to500 loop inj.m	Acquisition Date	04/10/2017 18:05:28
Ionisation Mode	positive electrospray (ESI)		

+MS, 1.0-1.3min #(119-154), -Spectral Bkgrnd

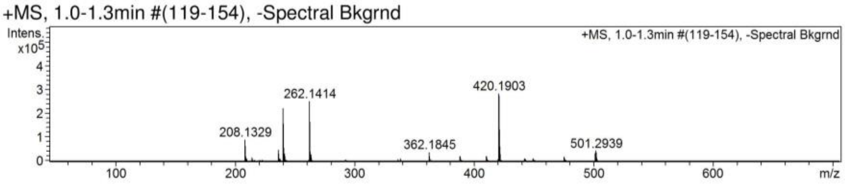

#	m/z	I	I %	Area	S/N
1	208.1329	90513	32.0	3827	2137.8
2	236.0946	45685	16.2	859	538.7
3	240.1595	220907	78.2	10574	2428.7
4	241.1623	30865	10.9	1444	333.7
5	262.1414	250061	88.5	12756	4062.4
6	263.1470	28424	10.1	1191	475.3
7	362.1845	37651	13.3	2398	1085.4
8	420.1903	282567	100.0	21798	3663.6
9	421.1931	63598	22.5	4794	835.4
10	501.2939	42703	15.1	4130	1777.0

Generate Molecular Formula Parameters

Charge	Tolerance	SearchRadius	H/C Ratio min.	H/C Ratio max.	Electron Conf.	Nitrogen Rule	sigma limit
positive	25 ppm	0.05 m/z	0	3	both	true	0.05

Expected Formula C19H25N5O6 **Adduct(s):** H, Na

#	meas. m/z	theo. m/z	Err[ppm]	Sigma	Formula
1	420.1903	420.1878	6.10	0.0094	C 19 H 26 N 5 O 6
1	442.1706	442.1697	2.00	0.0212	C 19 H 25 N 5 Na 1 O 6

Note: Sigma fits < 0.05 indicates high probability of correct MF.

For formula confirmation the mass error / accuracy at 200 Da should be better than 25 ppm, for 500 Da better than 10 ppm and for 1000 Da better than 5 ppm

Figure S112. MS data of [4.3.1]azabicyclic DNP derivative (**24**)

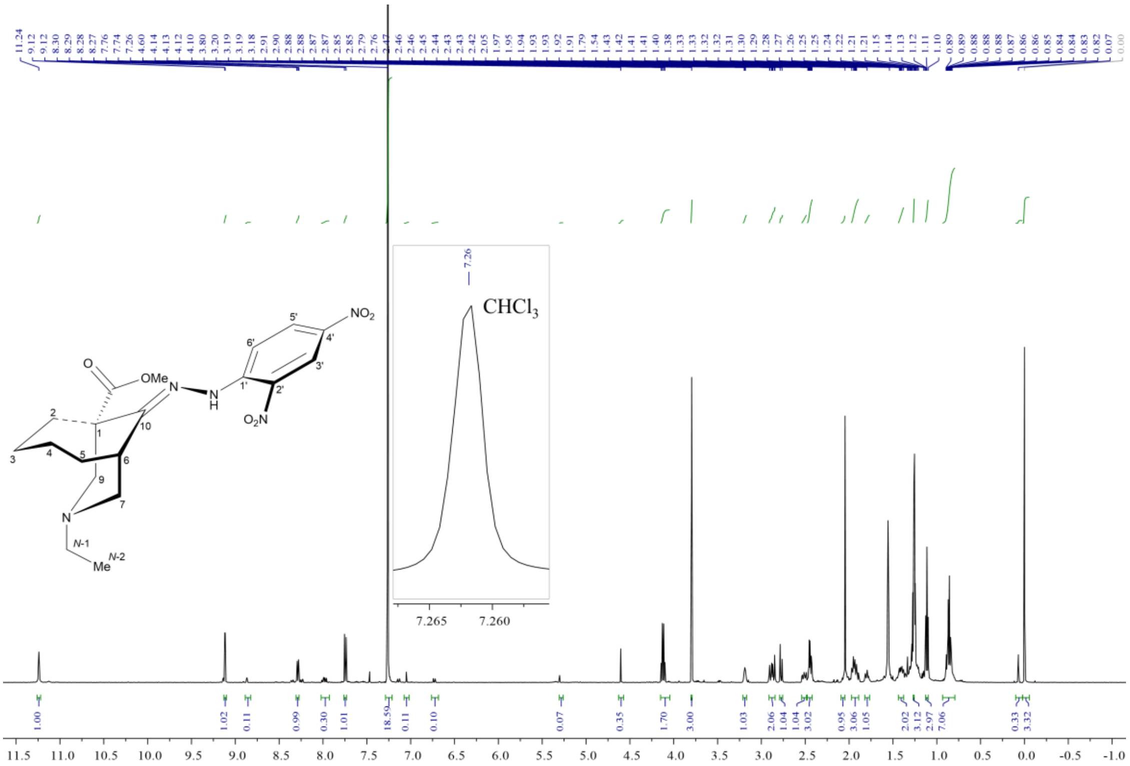

Figure S113. ^{1}H NMR spectrum of [4.3.1]azabicyclic DNP derivative (**24**) in $CDCl_3$

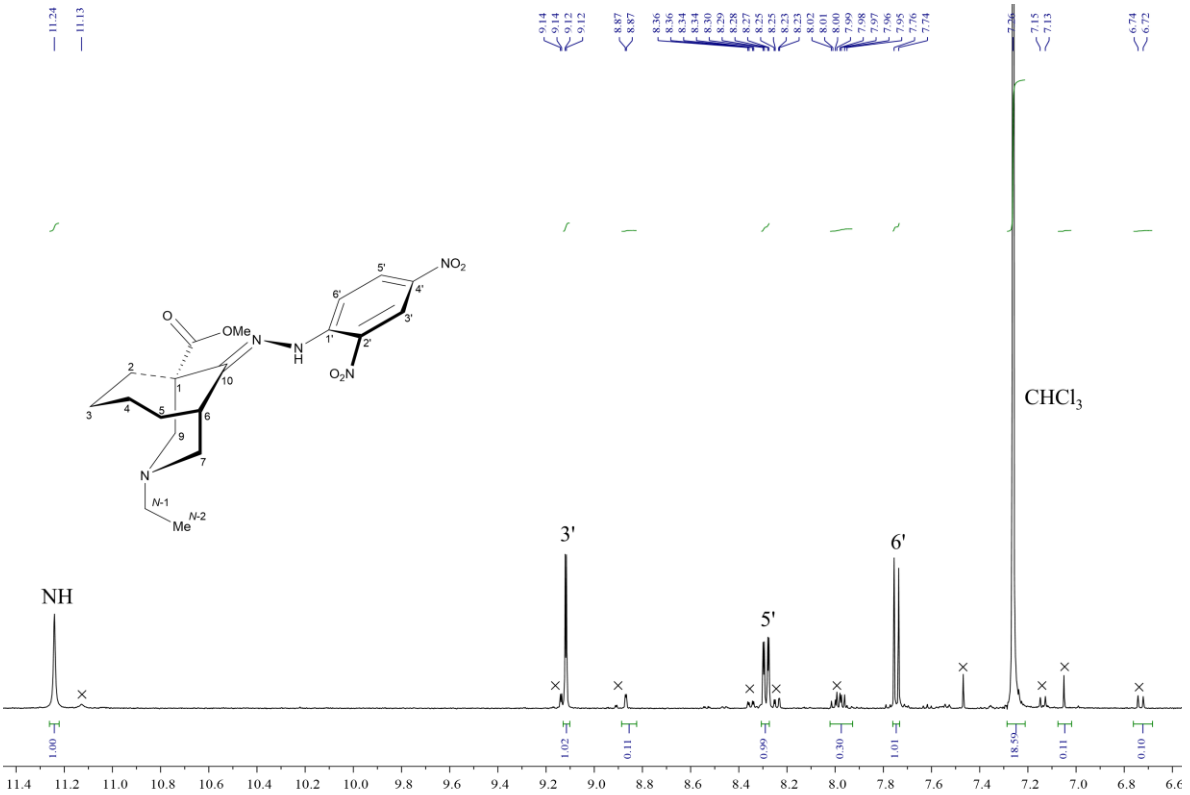

Figure S114. ^{1}H NMR expansion of [4.3.1]azabicyclic DNP derivative (**24**) in $CDCl_3$ with assignments

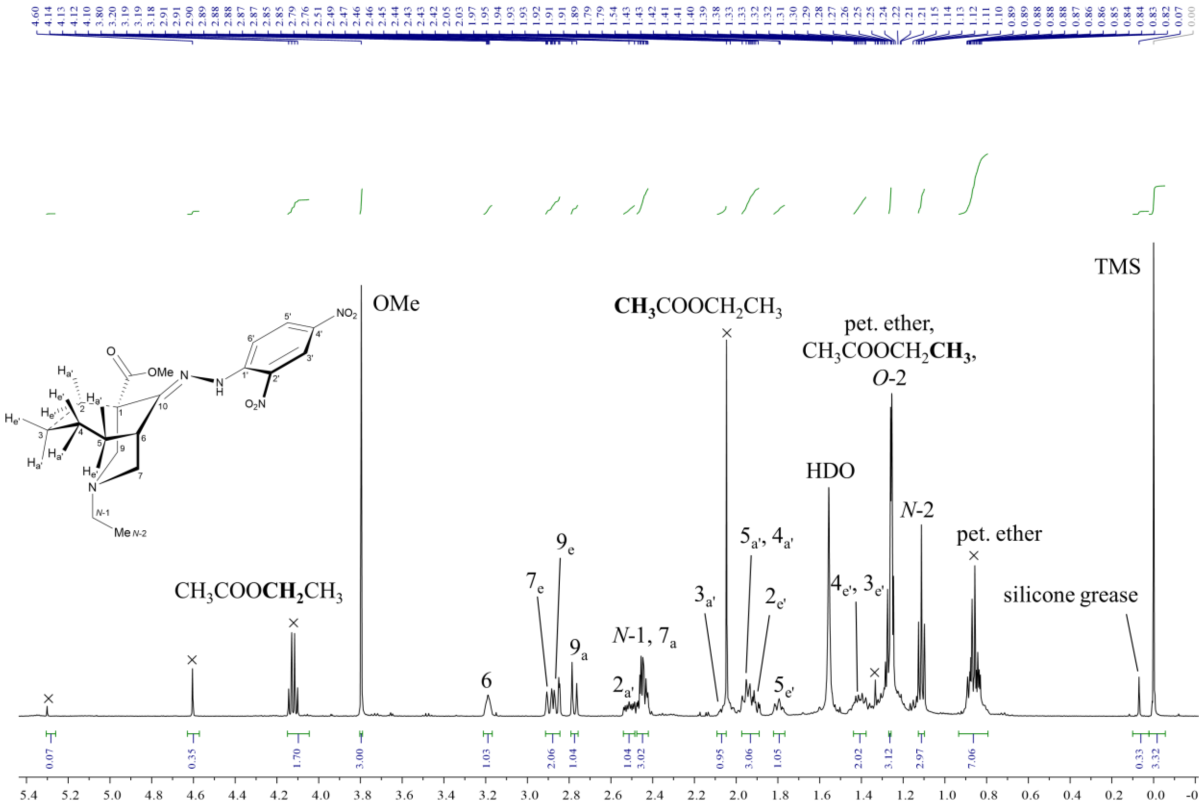

Figure S115. ^{1}H NMR expansion of [4.3.1]azabicyclic DNP derivative (**24**) in $CDCl_3$ with assignments (residual EtOAc, HDO, petroleum ether, and silicone grease are displayed)[1]

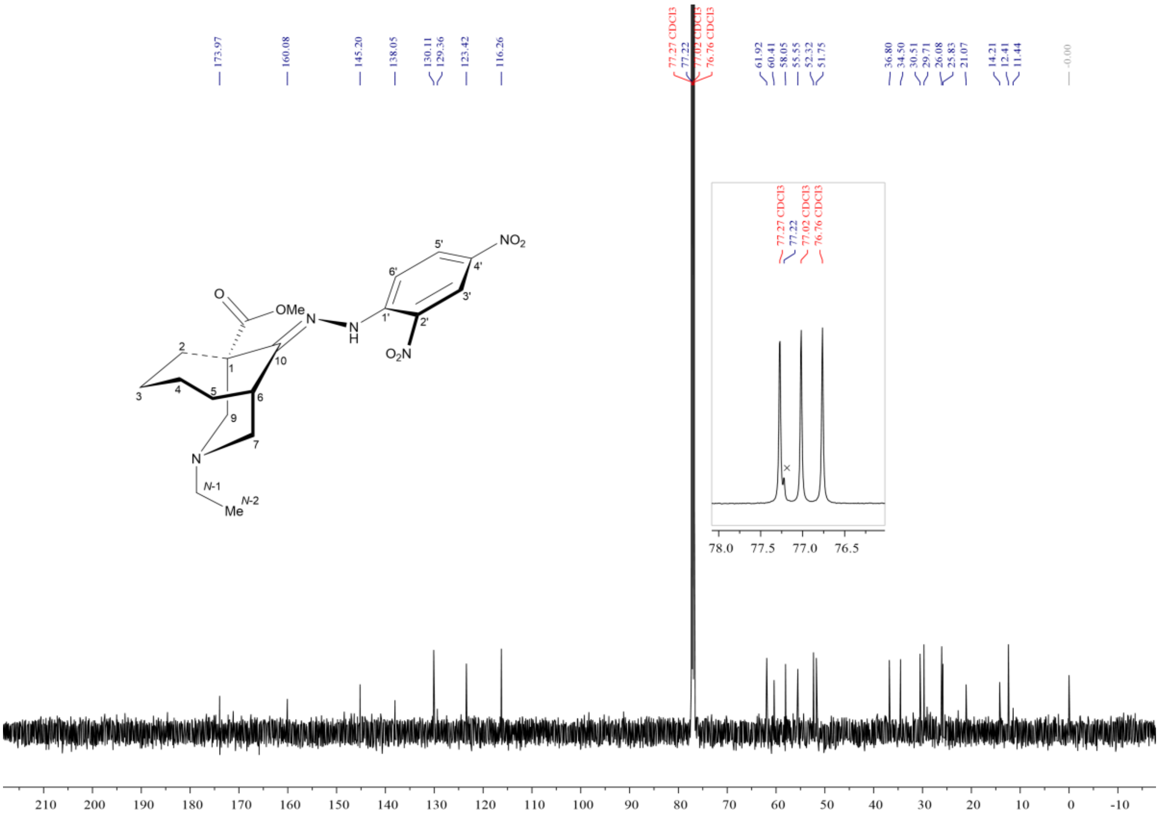

Figure S116. ^{13}C NMR spectrum of [4.3.1]azabicyclic DNP derivative (**24**) in $CDCl_3$

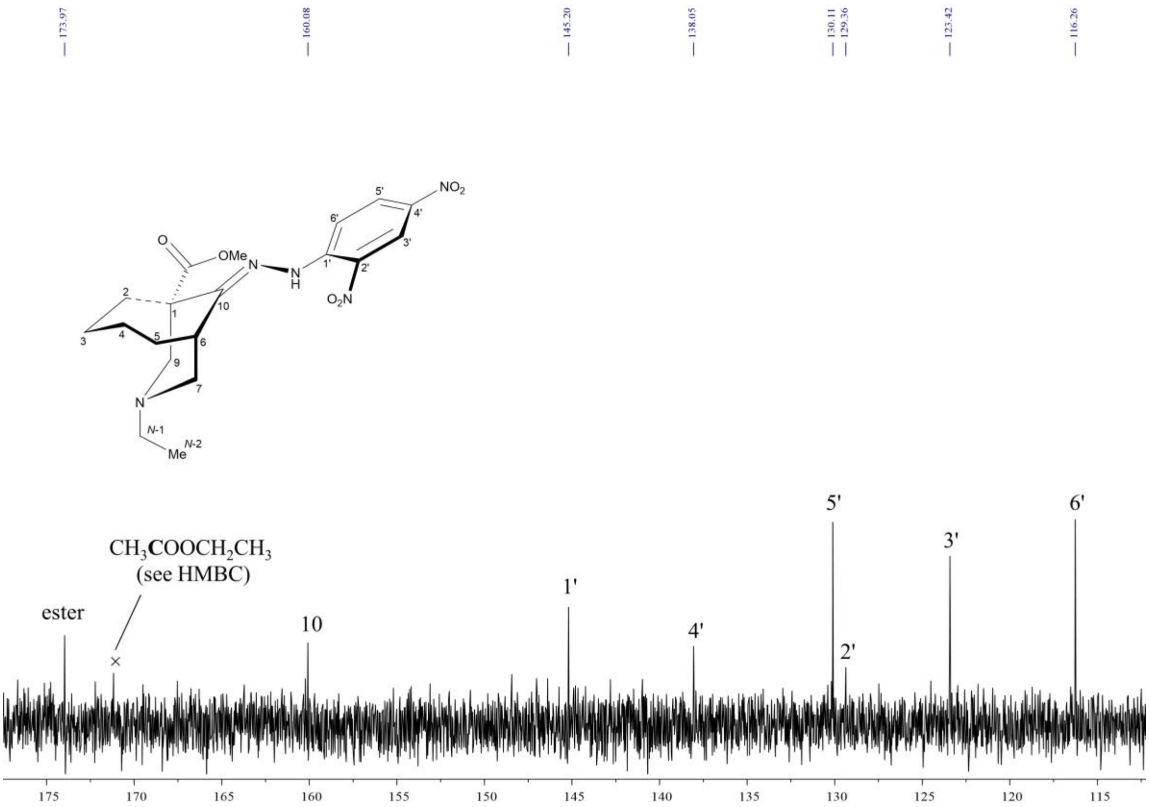

Figure S117. ^{13}C NMR expansion of [4.3.1]azabicyclic DNP derivative (**24**) in $CDCl_3$ with assignments (residual EtOAc is shown)[1]

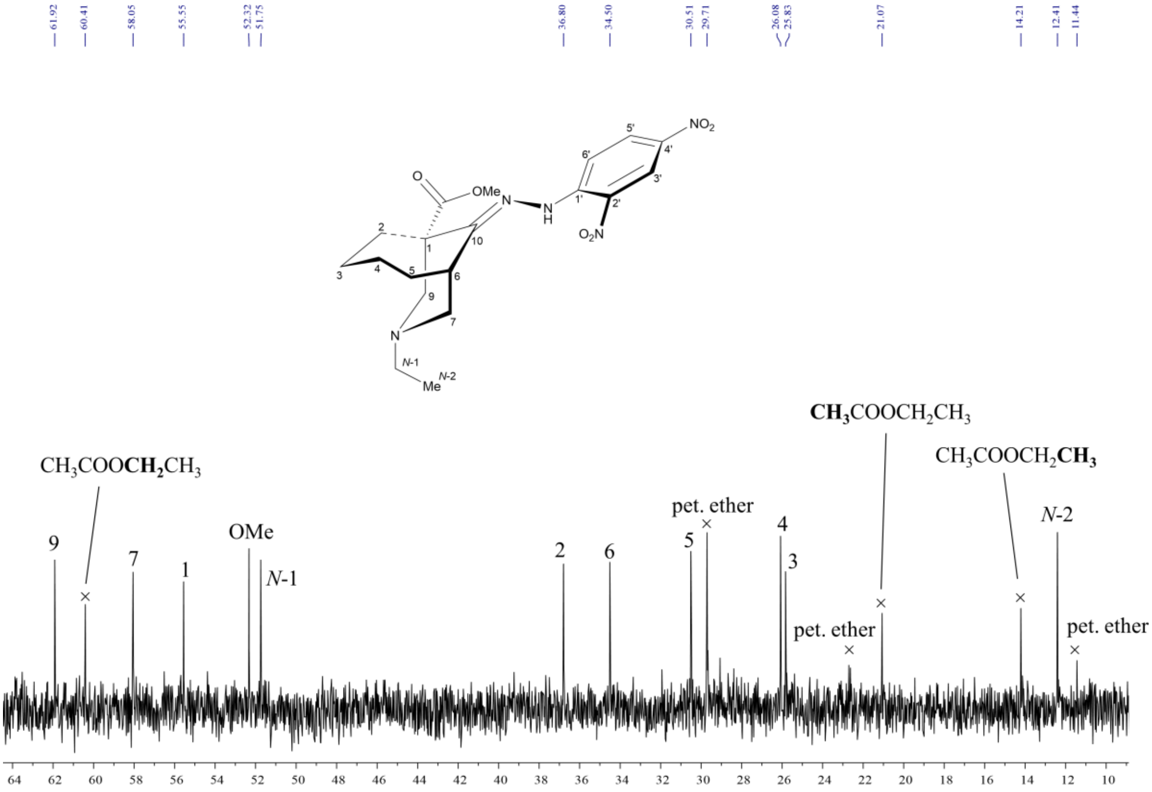

Figure S118. ^{13}C NMR expansion of [4.3.1]azabicyclic DNP derivative (**24**) in $CDCl_3$ with assignments (residual EtOAc and petroleum ether are displayed)[1]

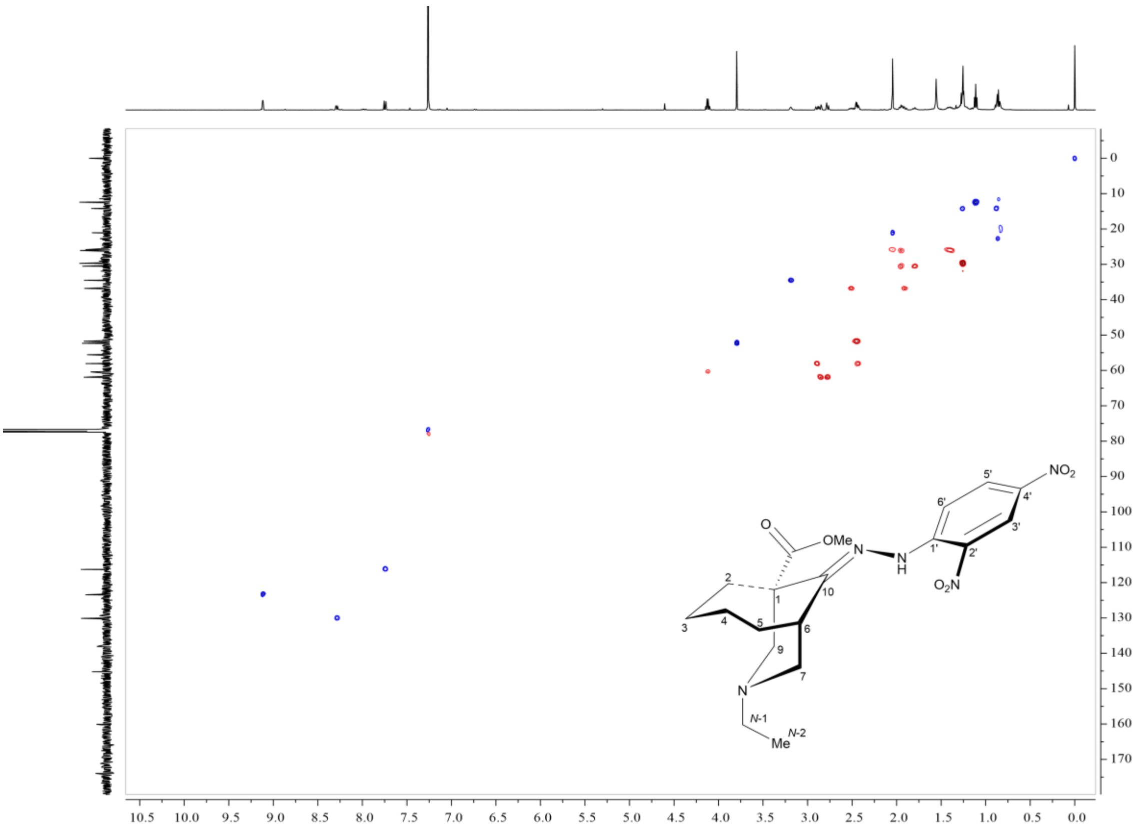

Figure S119. HSQC spectrum of [4.3.1]azabicyclic DNP derivative (**24**) in $CDCl_3$

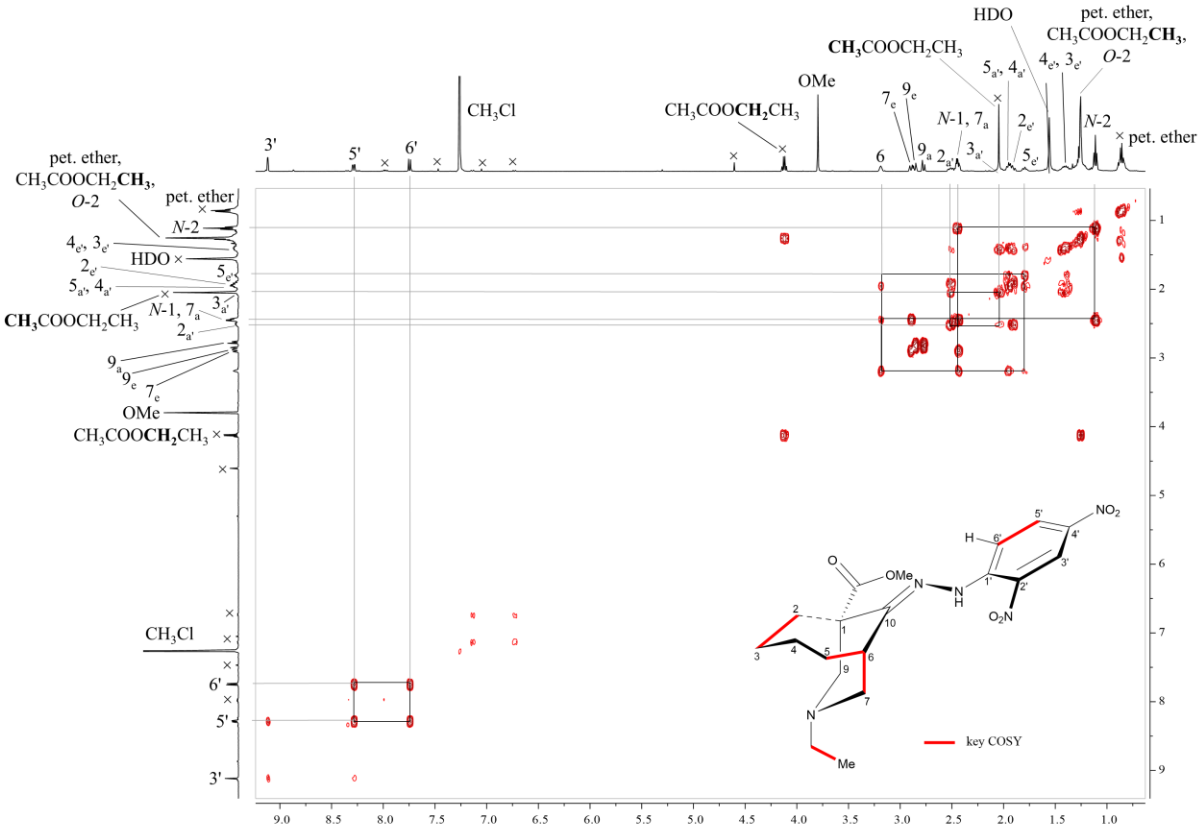

Figure S120. COSY spectrum of [4.3.1]azabicyclic DNP derivative (**24**) in $CDCl_3$

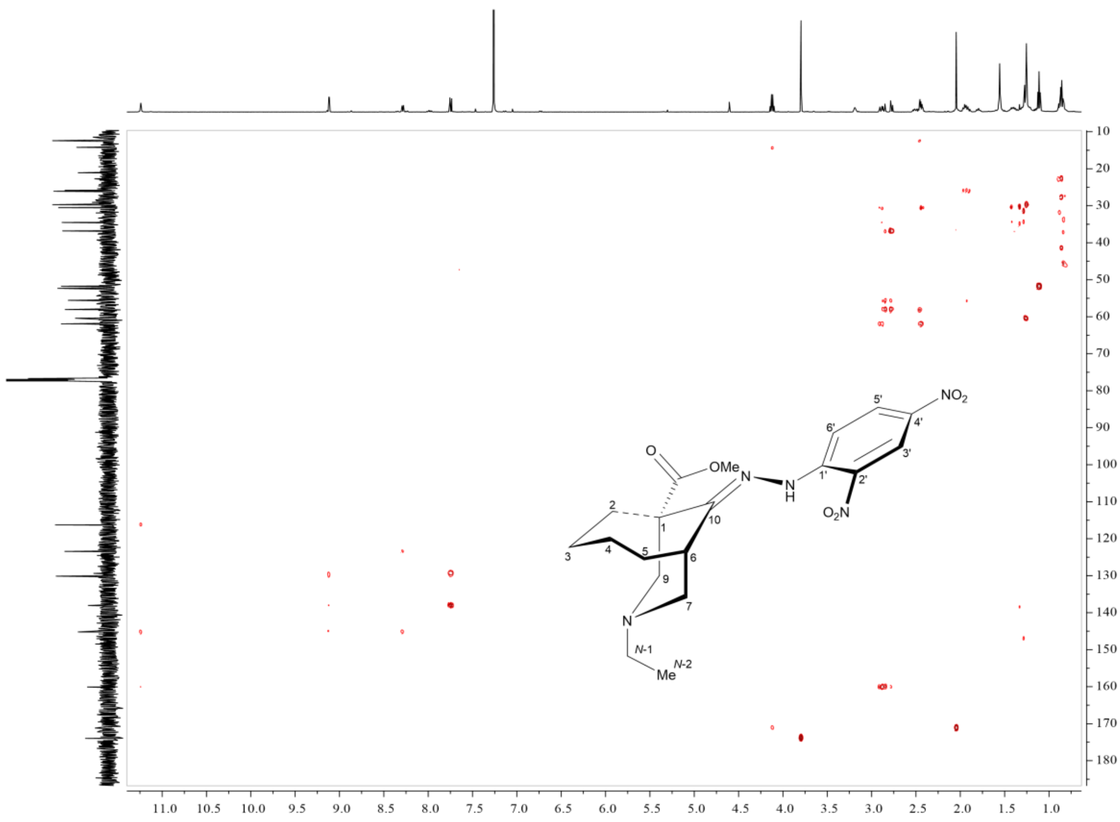

Figure S121. HMBC spectrum of [4.3.1]azabicyclic DNP derivative (**24**) in $CDCl_3$

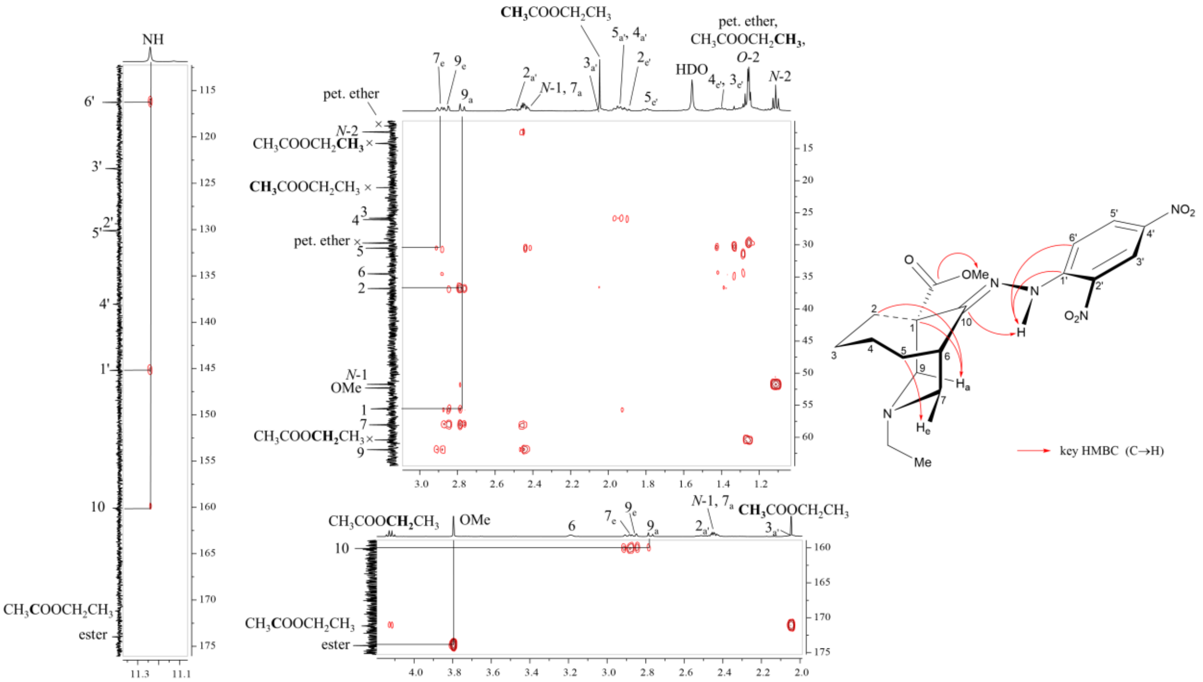

Figure S122. HMBC expansions of [4.3.1]azabicyclic DNP derivative (**24**) in $CDCl_3$

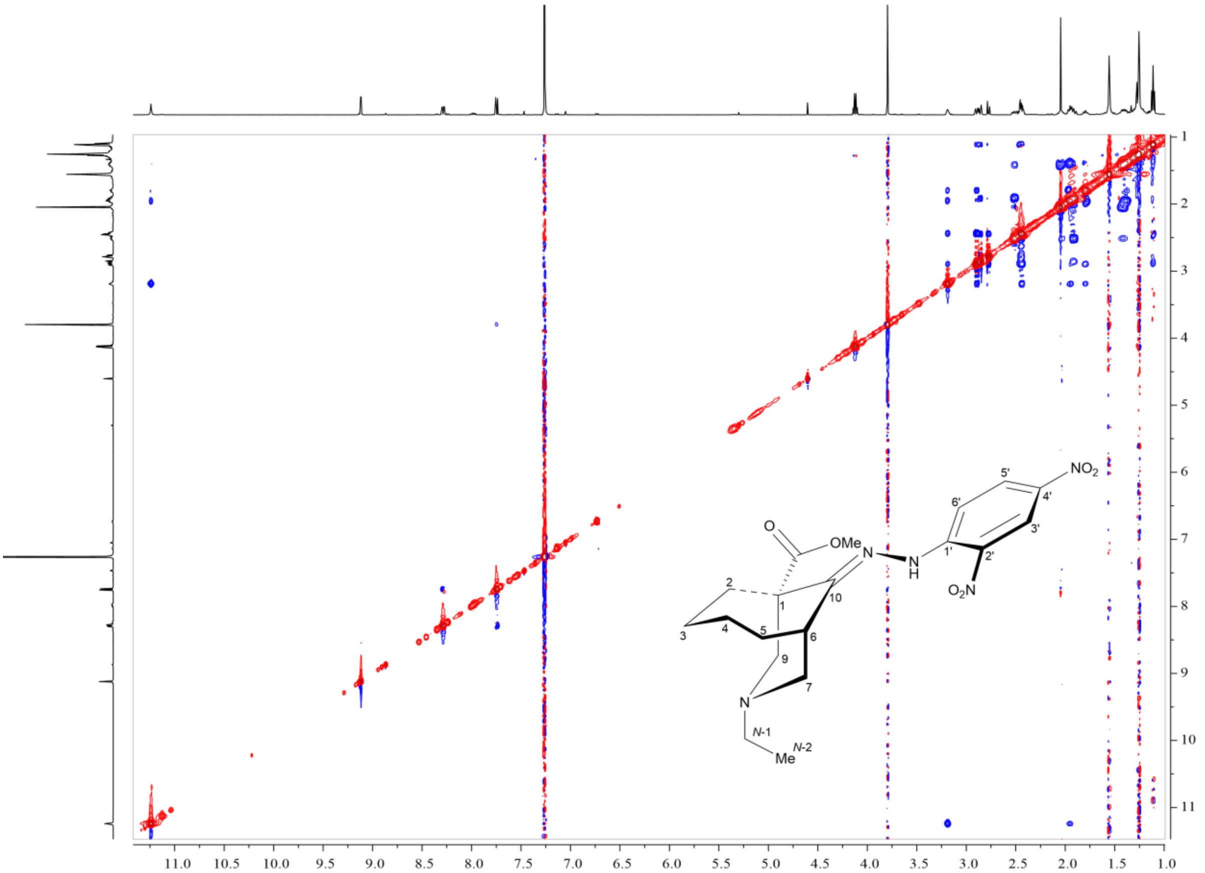

Figure S123. NOESY spectrum of [4.3.1]azabicyclic DNP derivative (**24**) in $CDCl_3$

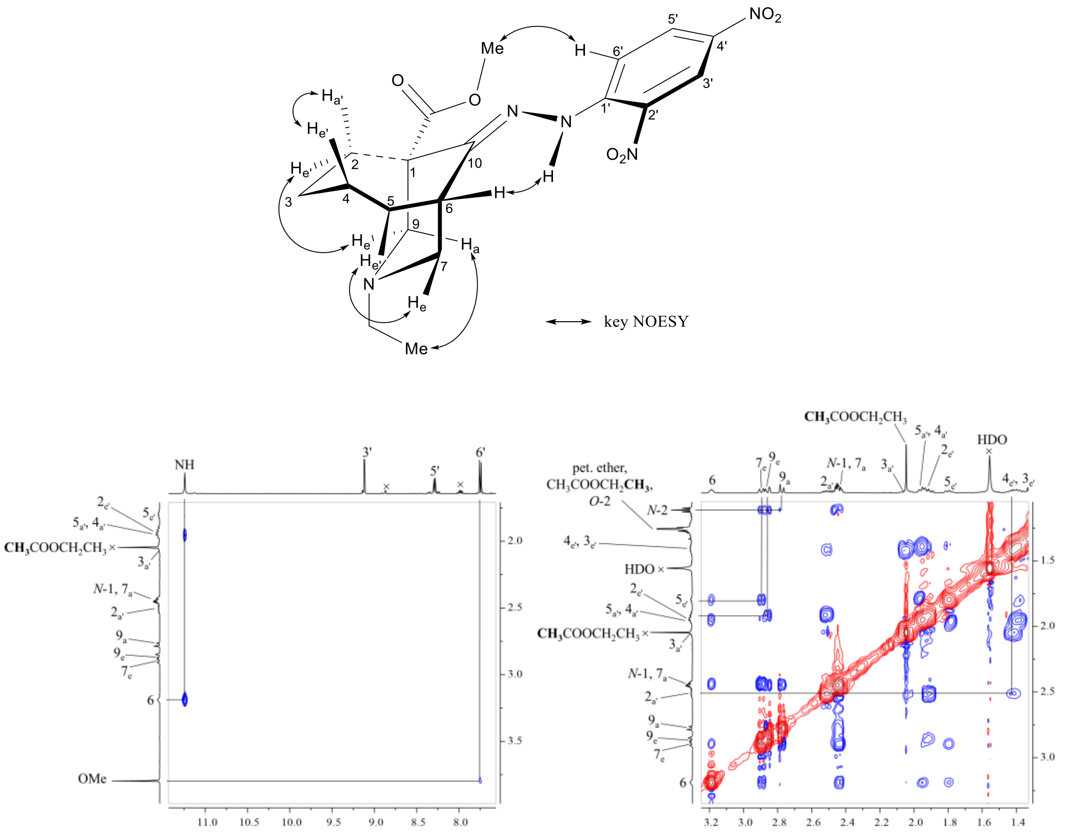

Figure S124. NOESY expansions of [4.3.1]azabicyclic DNP derivative (**24**) in $CDCl_3$

Ethyl 3-ethyl-8-oxo-3-azabicyclo[3.2.1]octane-1-carboxylate (**27**)

Confirmation of Expected Formula

Sample-ID	bl_zz_5-AE final	Submitter	zz615 Ziyu Zeng
Analysis Name	bl_zz_5-AE final_355528_31_01_61384.d	Supervisor	- Ian Blagbrough
Method used	Confirm Formula Positive 50to500 loop inj.m	Acquisition Date	23/01/2018 17:26:09
Ionisation Mode	positive electrospray (ESI)		

+MS, 1.0-1.3min #(119-153), -Spectral Bkgrnd

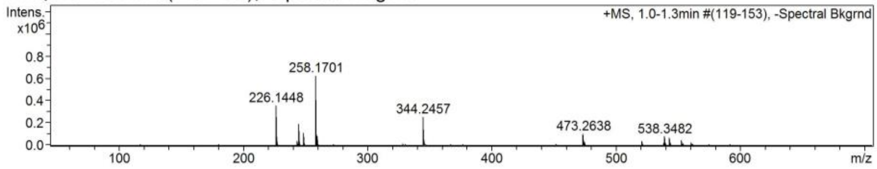

#	m/z	I	I %	Area	S/N
1	226.1448	360109	57.6	16100	2754.3
2	244.1543	192784	30.9	9850	1014.6
3	248.1265	107748	17.2	5097	615.4
4	258.1701	624801	100.0	34699	4554.7
5	259.1745	83958	13.4	4161	629.0
6	344.2457	256002	41.0	16460	6604.7
7	345.2481	50236	8.0	3187	1322.2
8	473.2638	106224	17.0	9613	3329.2
9	538.3482	79782	12.8	8618	833.7
10	542.3213	67403	10.8	7087	649.8

Generate Molecular Formula Parameters

Charge	Tolerance	SearchRadius	H/C Ratio min.	H/C Ratio max.	Electron Conf.	Nitrogen Rule	sigma limit
positive	25 ppm	0.05 m/z	0	3	both	true	0.05

Expected Formula C12H19NO3 **Adduct(s):** H, Na

#	meas. m/z	theo. m/z	Err[ppm]	Sigma	Formula
1	226.1448	226.1438	4.50	0.0021	C 12 H 20 N 1 O 3
1	248.1265	248.1257	3.20	0.0024	C 12 H 19 N 1 Na 1 O 3

Note: Sigma fits < 0.05 indicates high probability of correct MF.

Figure S125. MS data of [3.2.1]azabicycle (**27**)

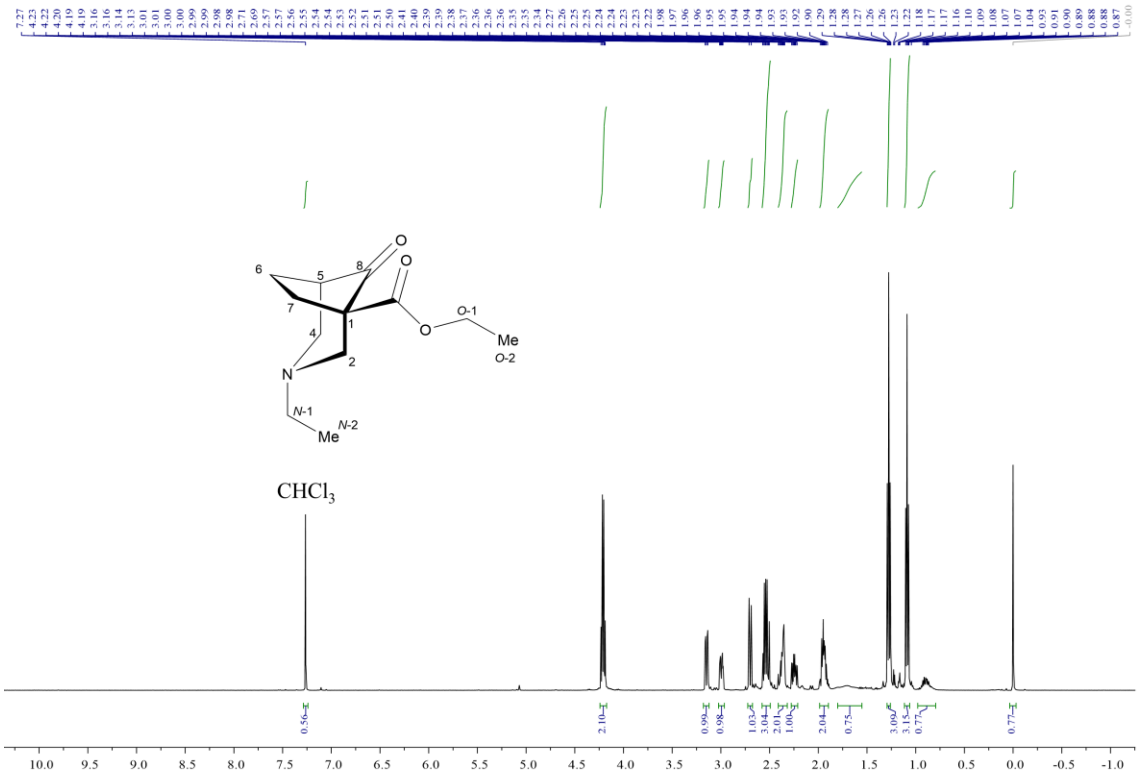

Figure S126. ^{1}H NMR spectrum of [3.2.1]azabicycle (27) in $CDCl_3$

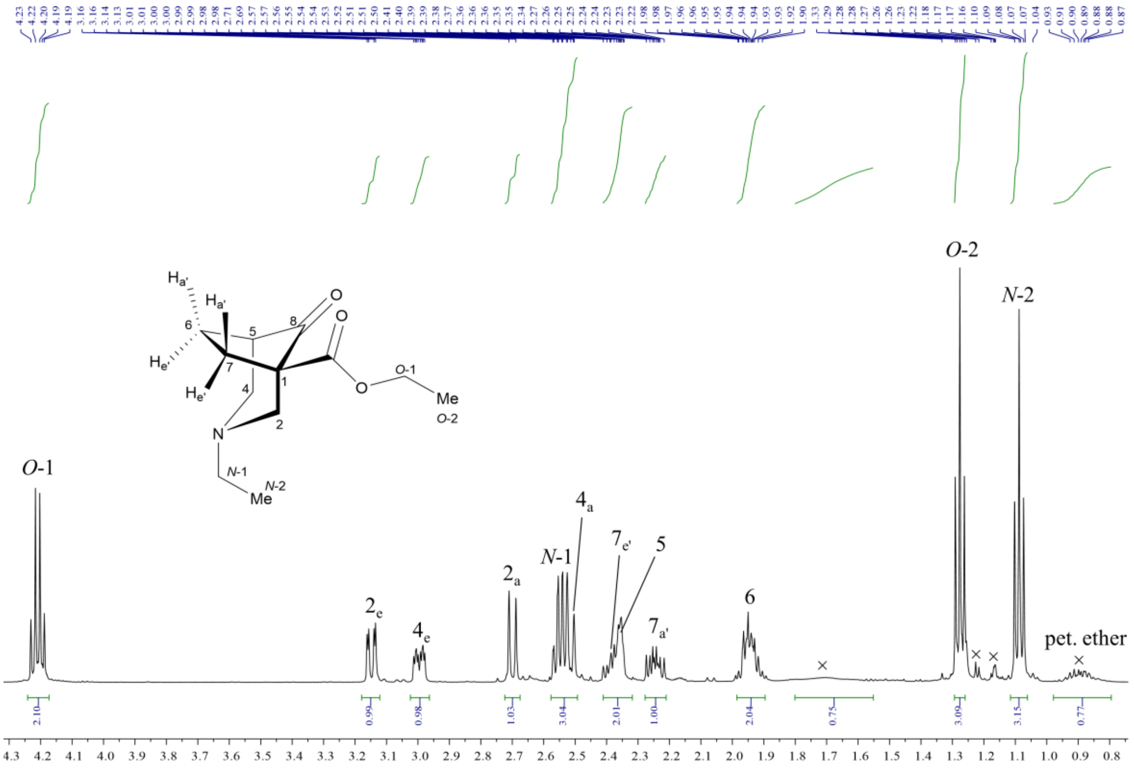

Figure S127. ^{1}H NMR spectrum expansion of [3.2.1]azabicycle (**27**) in $CDCl_3$ with assignments (residual petroleum ether is displayed)[1]

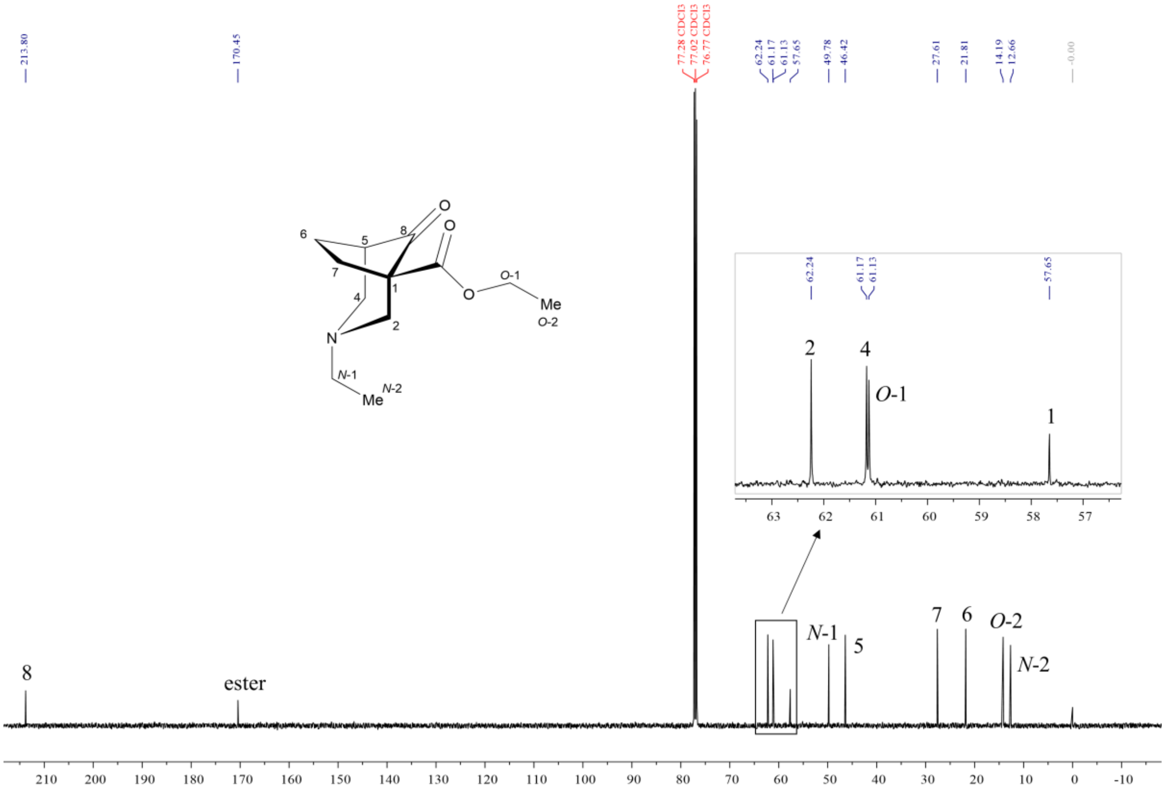

Figure S128. ^{13}C NMR spectrum of [3.2.1]azabicycle (**27**) in $CDCl_3$ with assignments

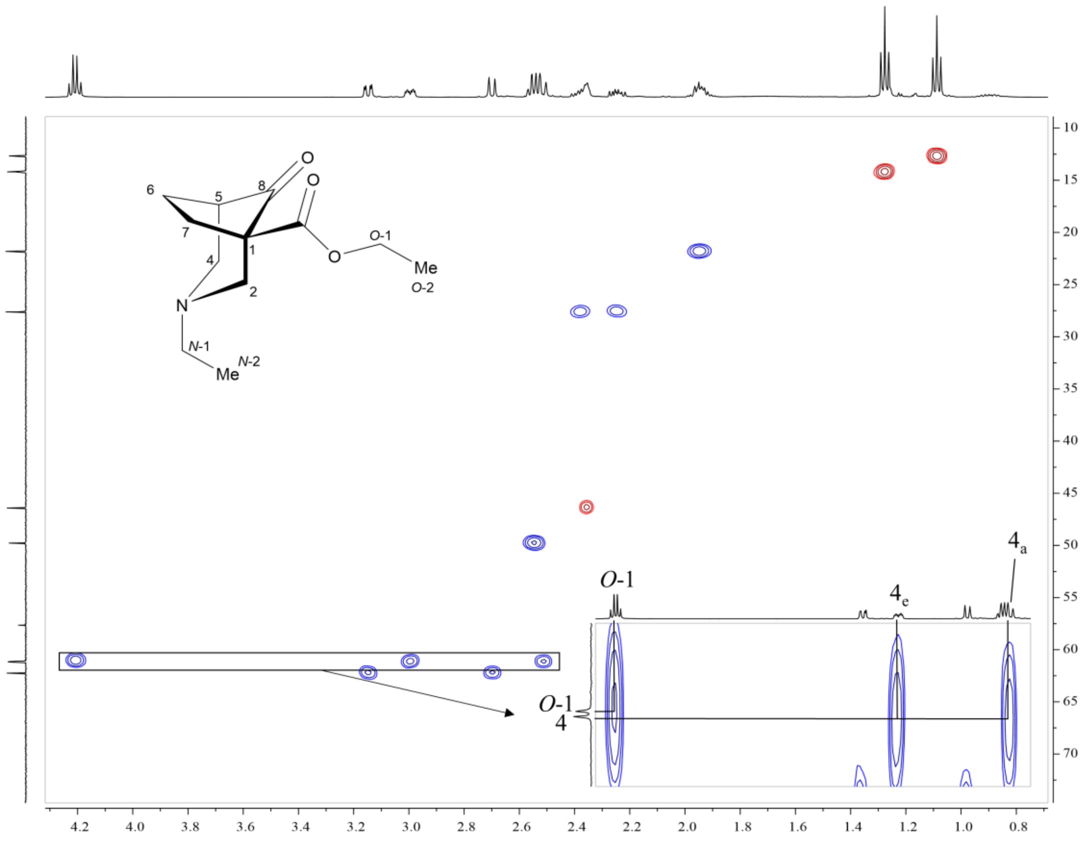

Figure S129. HSQC spectrum of [3.2.1]azabicycle (**27**) in $CDCl_3$

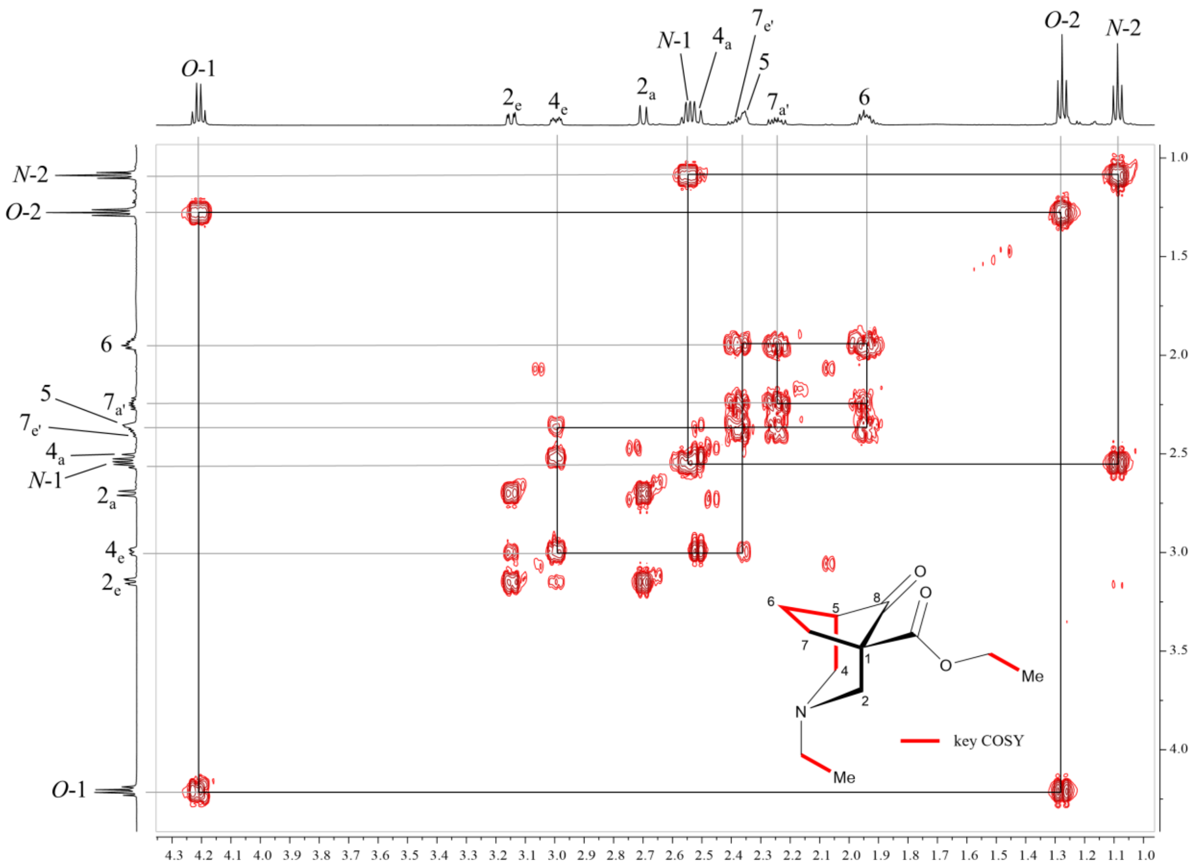

Figure S130. COSY spectrum of [3.2.1]azabicycle (**27**) in $CDCl_3$

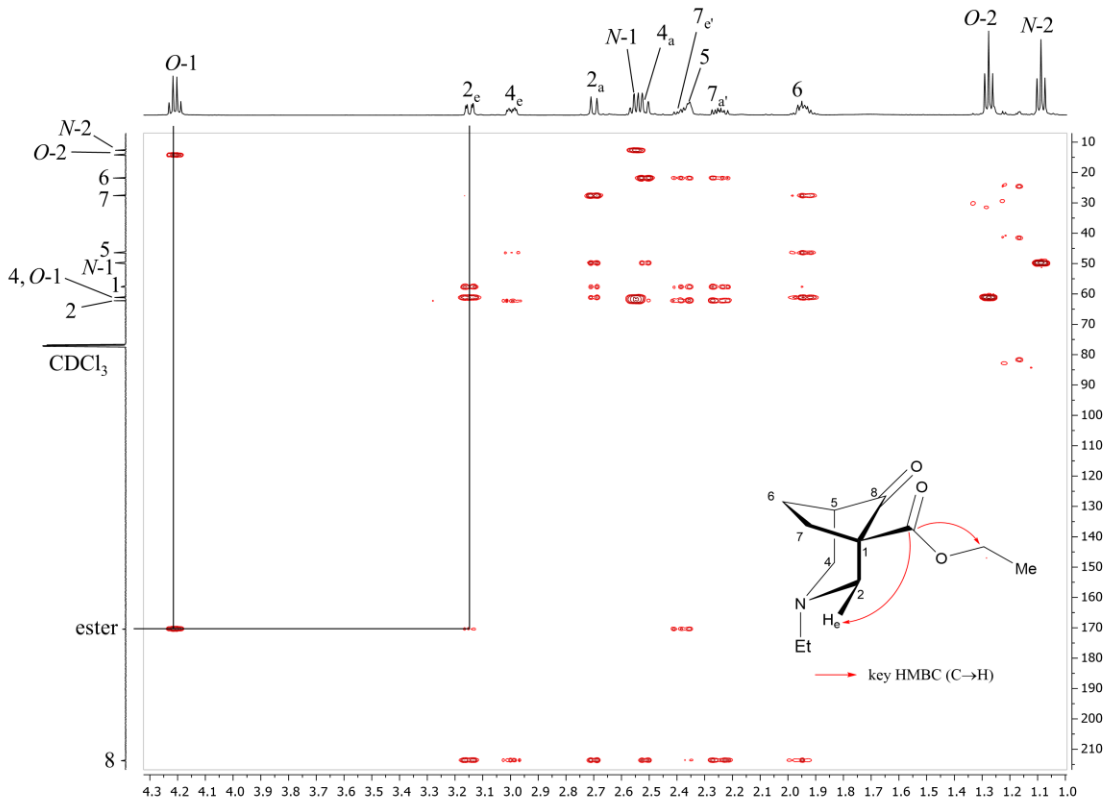

Figure S131. HMBC spectrum of [3.2.1]azabicycle (**27**) in $CDCl_3$

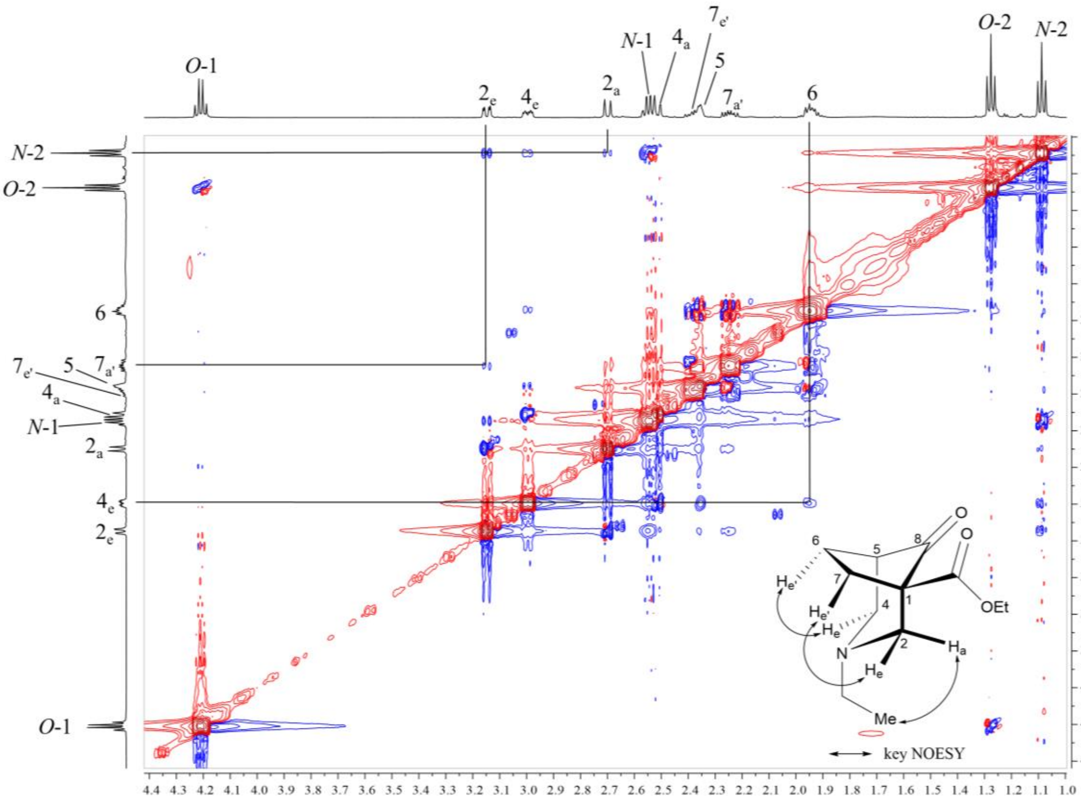

Figure S132. NOESY spectrum of [3.2.1]azabicycle (**27**) in $CDCl_3$

Ethyl (*E*)-8-(2-(2,4-dinitrophenyl)hydrazinylidene)-3-ethyl-3-azabicyclo[3.2.1]octane-1-carboxylate (**28**) [1-(2,4-dinitrophenyl)-2-(propan-2-ylidene)hydrazine is also shown]

Confirmation of Expected Formula

Sample-ID	bl_zz_5-AE DNP final	Submitter	zz615 Ziyu Zeng
Analysis Name	bl_zz_5-AE DNP final_355580_80_01_61452.d	Supervisor	- Ian Blagbrough
Method used	Confirm Formula Positive 50to500 loop inj.m	Acquisition Date	25/01/2018 21:33:07
Ionisation Mode	positive electrospray (ESI)		

+MS, 1.0-1.3min #(119-154), -Spectral Bkgrnd

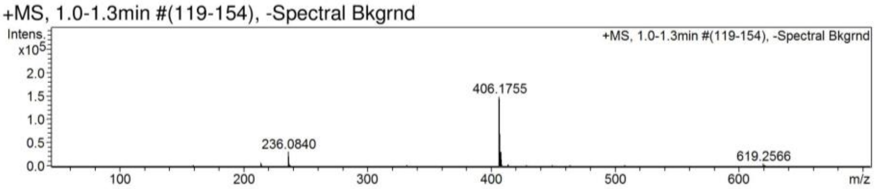

#	m/z	I	I %	Area	S/N
1	159.0975	2588	1.7	22	3059.1
2	214.0962	6872	4.6	204	1276.6
3	236.0840	30911	20.9	475	2969.4
4	237.0978	2696	1.8	30	253.3
5	406.1755	148077	100.0	11269	5441.4
6	407.1773	31070	21.0	2284	1117.2
7	408.1796	4649	3.1	319	163.6
8	413.2821	3566	2.4	196	113.3
9	449.1811	2678	1.8	109	168.2
10	619.2566	5115	3.5	597	1041.1

Generate Molecular Formula Parameters

Charge	Tolerance	SearchRadius	H/C Ratio min.	H/C Ratio max.	Electron Conf.	Nitrogen Rule	sigma limit
positive	25 ppm	0.05 m/z	0	3	both	true	0.05

Expected Formula C18H23N5O6 **Adduct(s):** H, Na

#	meas. m/z	theo. m/z	Err[ppm]	Sigma	Formula
1	406.1755	406.1721	8.30	0.0024	C 18 H 24 N 5 O 6

Note: Sigma fits < 0.05 indicates high probability of correct MF.

Figure S133. MS data of [3.2.1]azabicyclic DNP derivative (**28**)

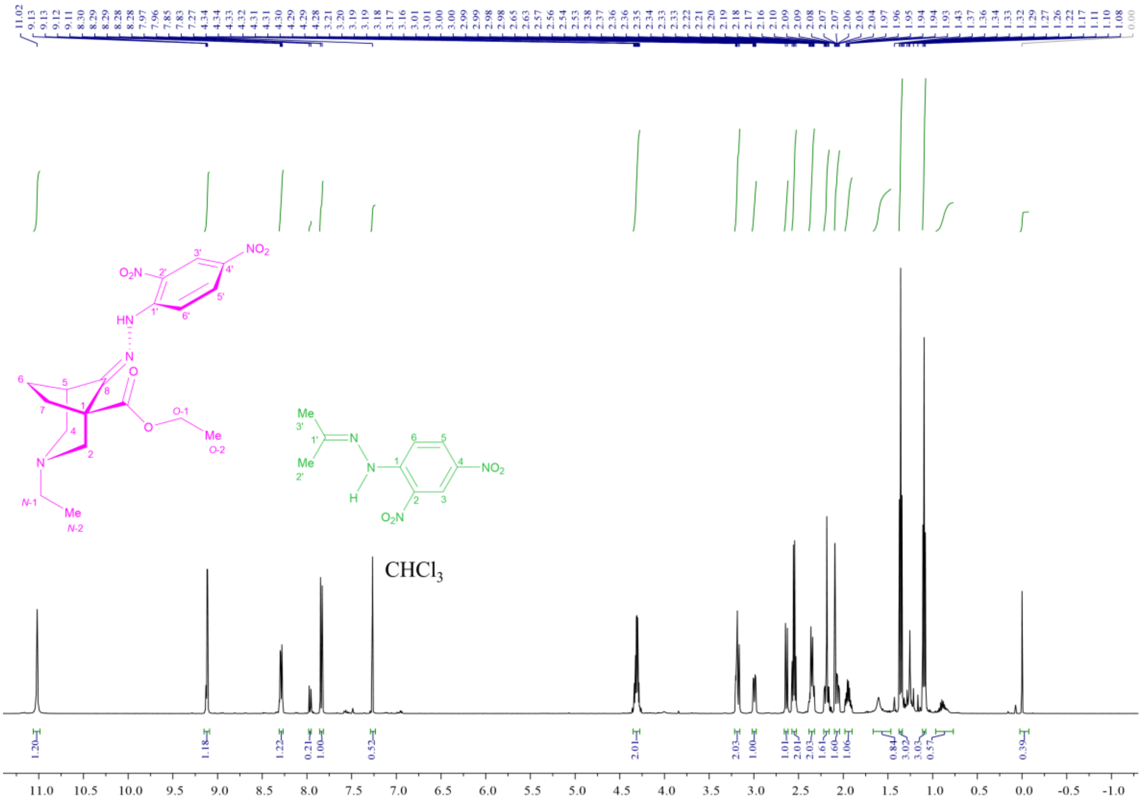

Figure S134. ^{1}H NMR spectrum of [3.2.1]azabicyclic DNP derivative (**28**) in $CDCl_3$

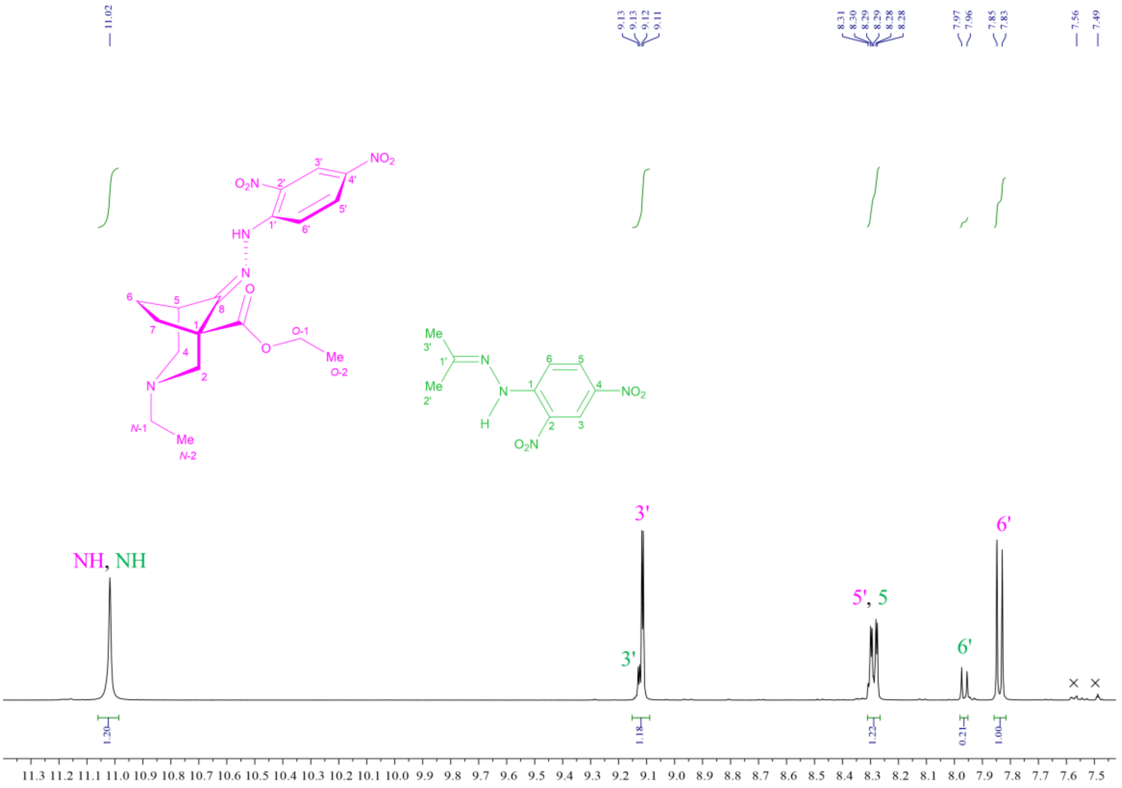

Figure S135. ^{1}H expansion spectrum of [3.2.1]azabicyclic DNP derivative (**28**) in $CDCl_3$ with assignments

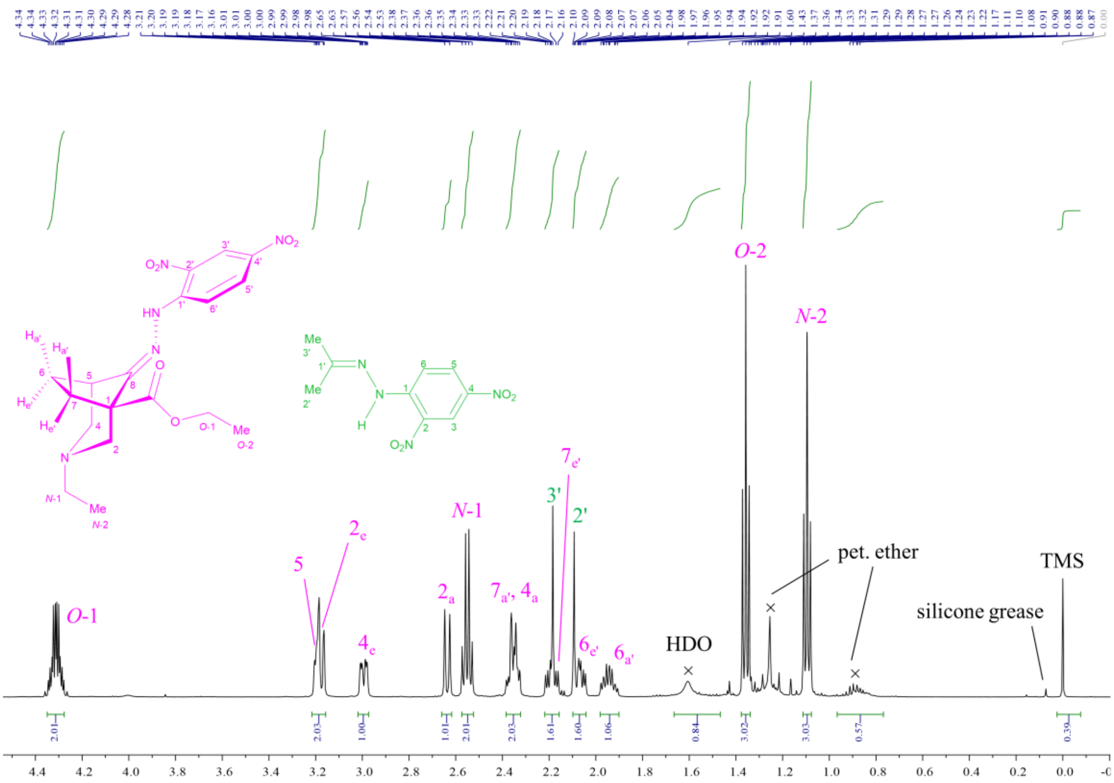

Figure S136. ^{1}H expansion spectrum of [3.2.1]azabicyclic DNP derivative (**28**) in $CDCl_3$ with assignments (residual petroleum ether and silicone grease are displayed)[1]

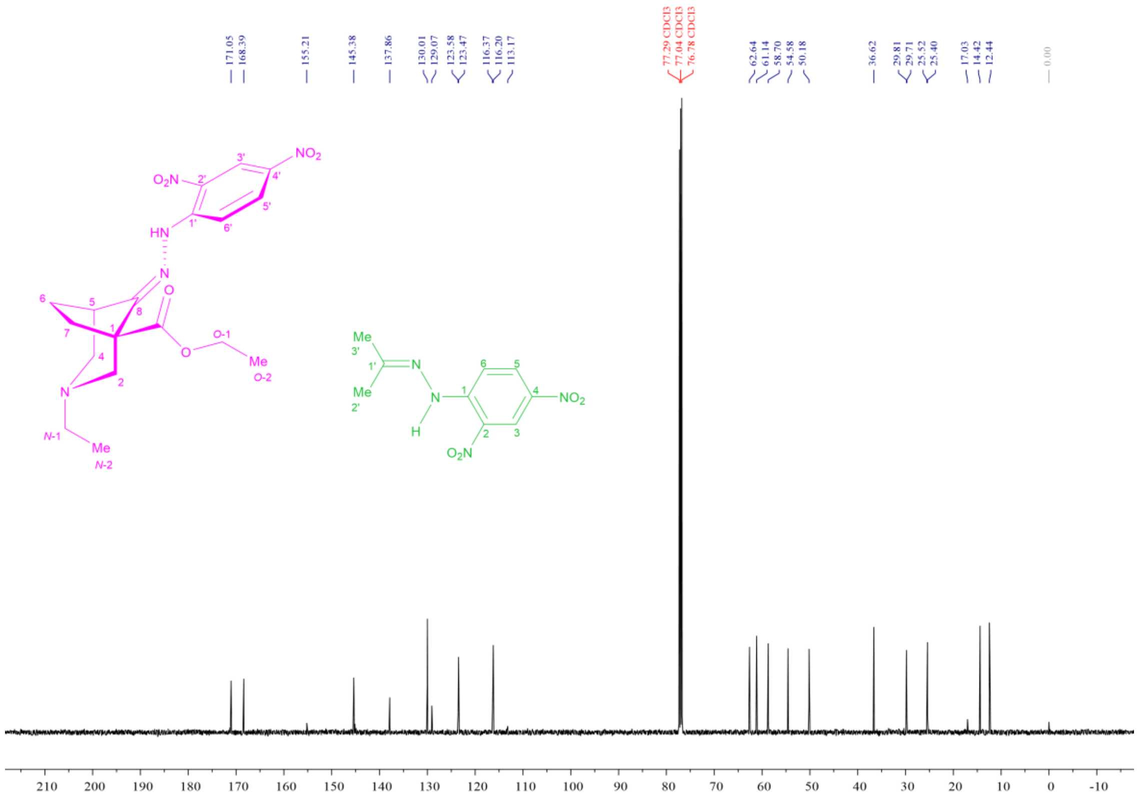

Figure S137. ^{13}C NMR spectrum of [3.2.1]azabicyclic DNP derivative (**28**) in $CDCl_3$

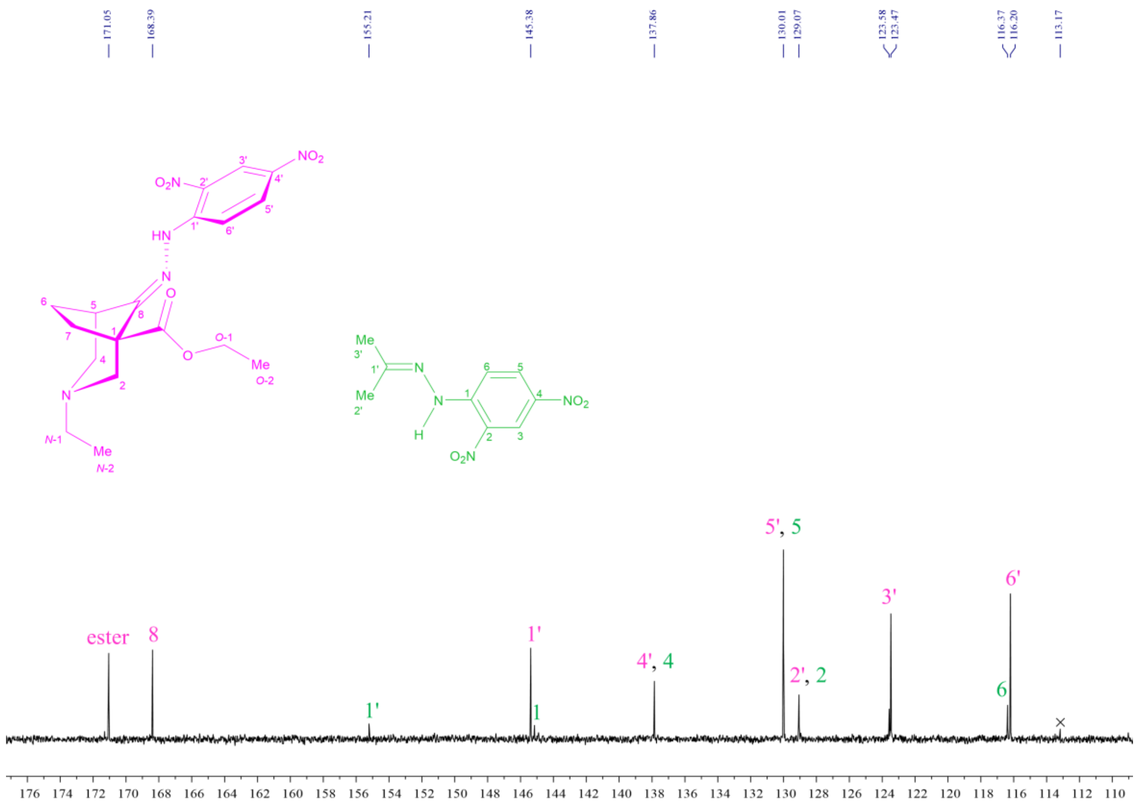

Figure S138. ^{13}C NMR expansion of [3.2.1]azabicyclic DNP derivative (**28**) in $CDCl_3$

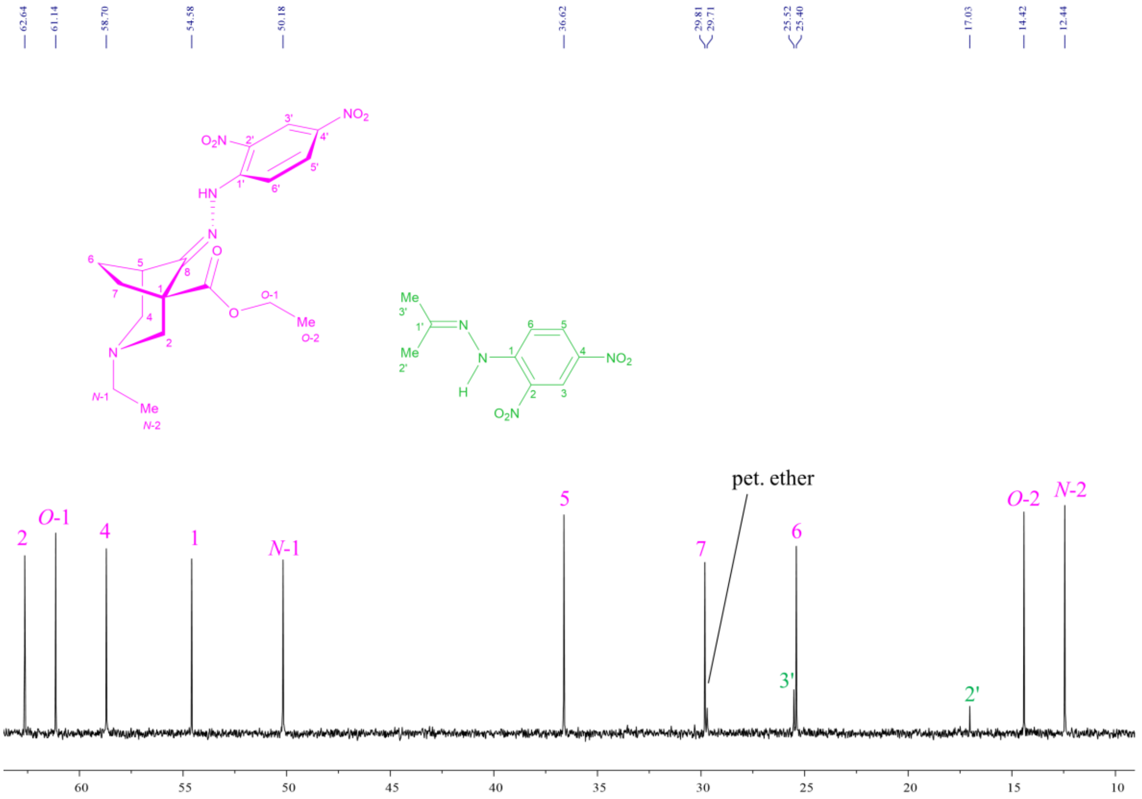

Figure S139. ^{13}C NMR expansion of [3.2.1]azabicyclic DNP derivative (**28**) in $CDCl_3$ (residual petroleum ether is displayed)[1]

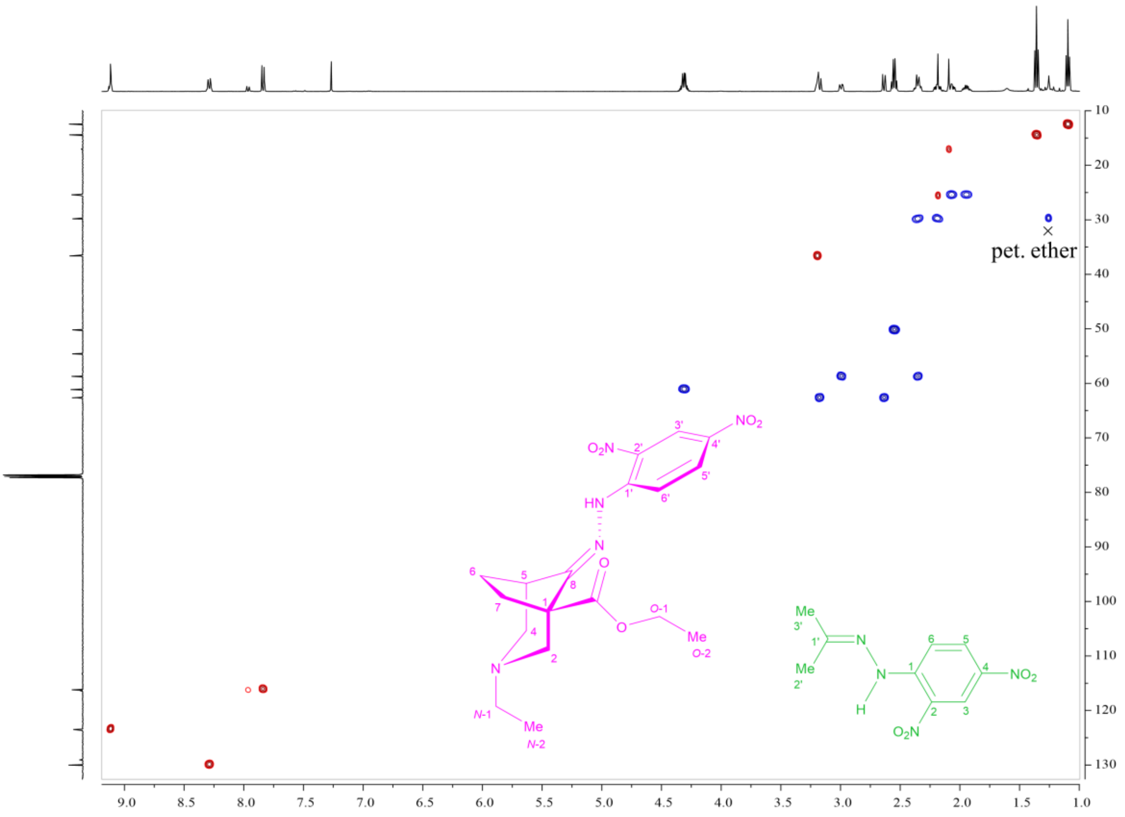

Figure S140. HSQC spectrum of [3.2.1]azabicyclic DNP derivative (**28**) in $CDCl_3$

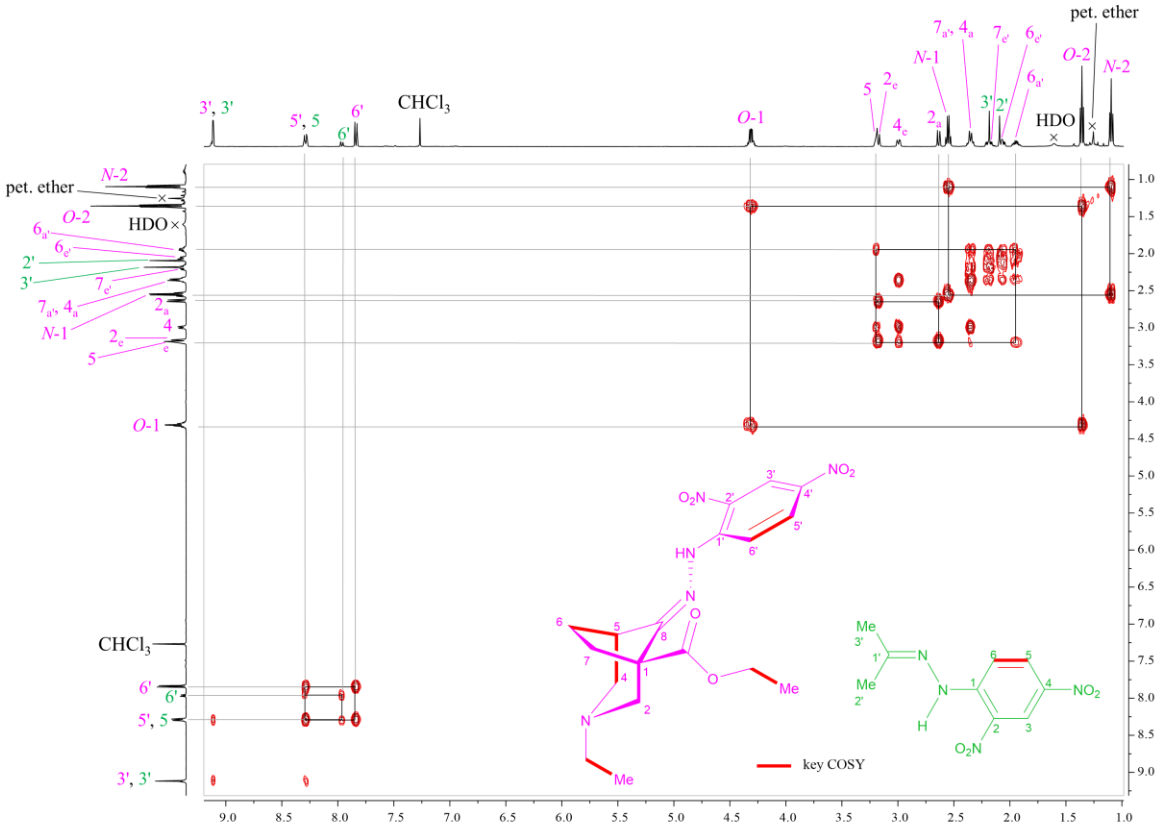

Figure S141. COSY spectrum of [3.2.1]azabicyclic DNP derivative (**28**) in $CDCl_3$

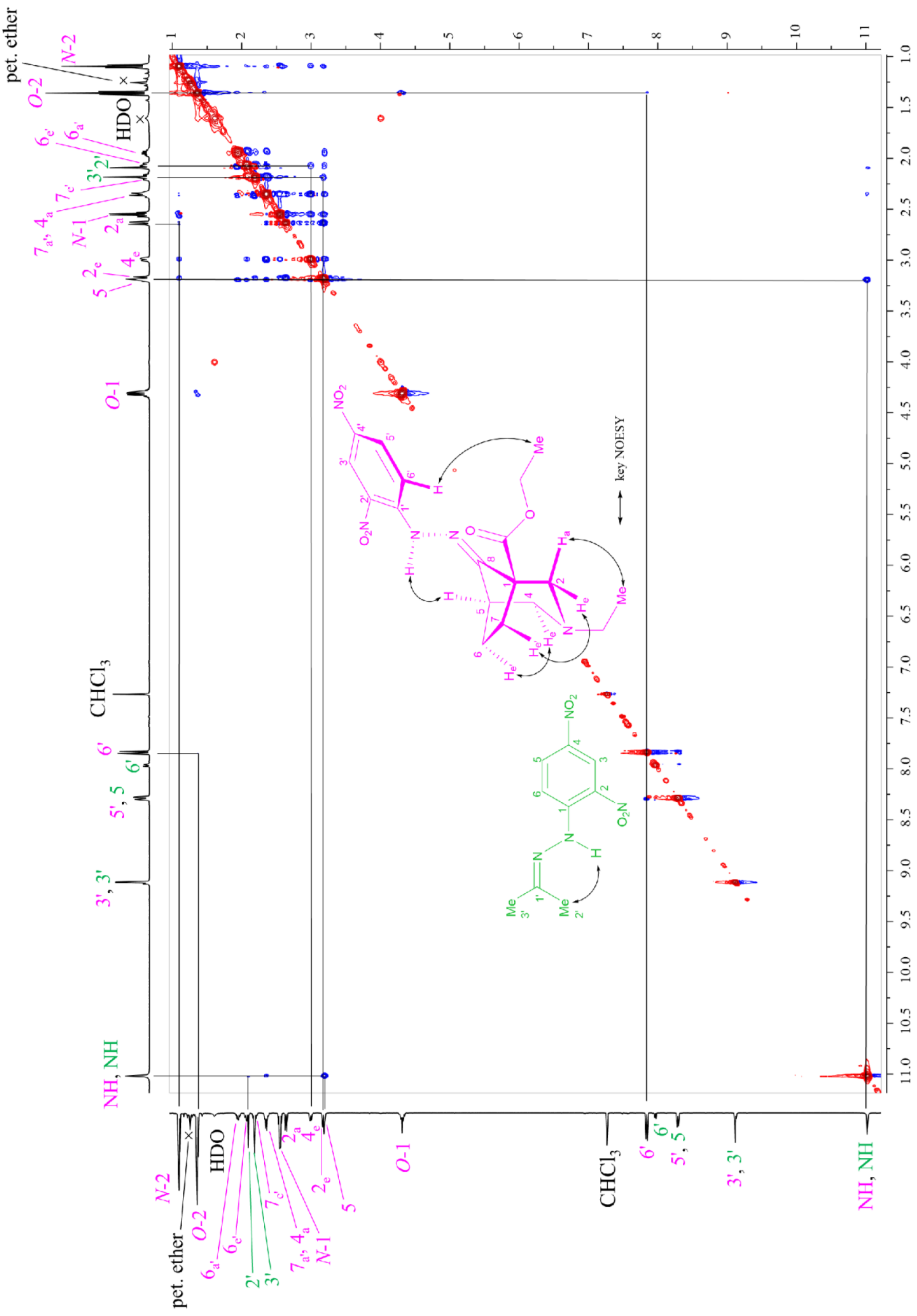

Figure S142. NOESY spectrum of [3.2.1]azabicyclic DNP derivative (**28**) in $CDCl_3$

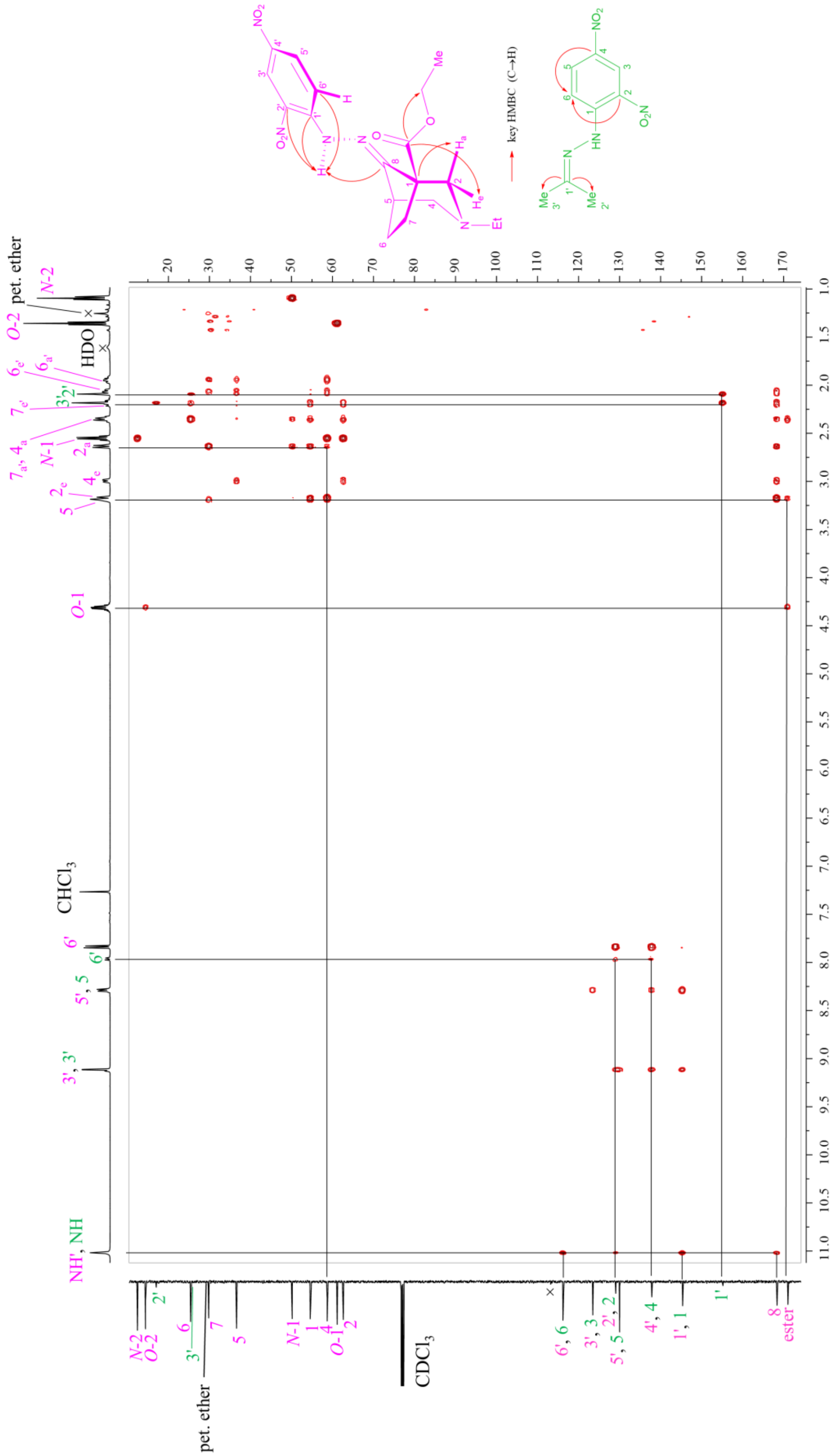

Figure S143. HMBC spectrum of [3.2.1]azabicyclic DNP derivative (**28**) in $CDCl_3$

Ethyl 1-[(ethylamino)methyl]-2-oxocyclohexane-1-carboxylate (**29**)

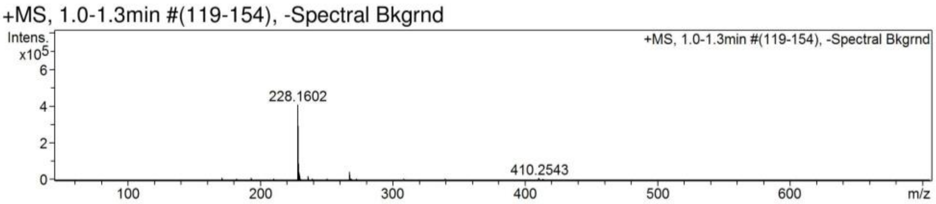

Confirmation of Expected Formula

Sample-ID	bl_zz_mono final	Submitter	zz615 Ziyu Zeng
Analysis Name	bl_zz_mono final _353346_82_01_58974.d	Supervisor	- Ian Blagbrough
Method used	Confirm Formula Positive 50to500 loop inj.m	Acquisition Date	15/09/2017 16:32:23
Ionisation Mode	positive electrospray (ESI)		

+MS, 1.0-1.3min #(119-154), -Spectral Bkgrnd

#	m/z	I	I %	Area	S/N
1	171.1017	8885	2.2	301	4300.6
2	193.0836	11849	2.9	451	3522.2
3	228.1602	406846	100.0	19282	12536.9
4	229.1623	46397	11.4	1810	1387.5
5	236.0741	21085	5.2	342	523.9
6	239.1742	7899	1.9	207	182.5
7	267.2067	45092	11.1	2236	1626.7
8	308.2367	7574	1.9	298	984.7
9	339.3370	7913	1.9	440	2969.5
10	410.2543	8889	2.2	625	1999.8

Generate Molecular Formula Parameters

Charge	Tolerance	SearchRadius	H/C Ratio min.	H/C Ratio max.	Electron Conf.	Nitrogen Rule	sigma limit
positive	10 ppm	0.05 m/z	0	3	both	true	0.05

Expected Formula C12H21NO3 **Adduct(s):** H, Na

#	meas. m/z	theo. m/z	Err[ppm]	Sigma	Formula
1	228.1602	228.1594	3.40	0.0112	C 12 H 22 N 1 O 3

Note: Sigma fits < 0.05 indicates high probability of correct MF, and mass accuracy of 5ppm or better is generally acceptable for publication

Figure S144. MS data of mono-Mannich product (**29**)

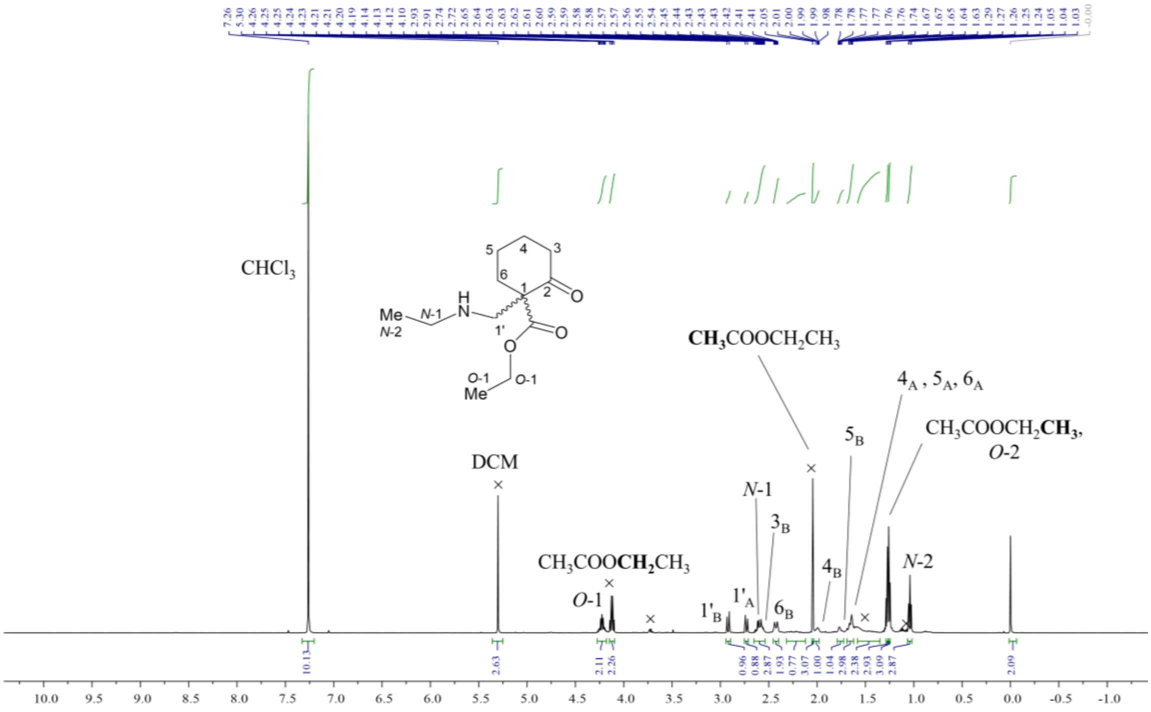

Figure S145. ^{1}H NMR spectrum of mono-Mannich product (**29**) in $CDCl_3$ with assignments (residual EtOAc is displayed)[1]

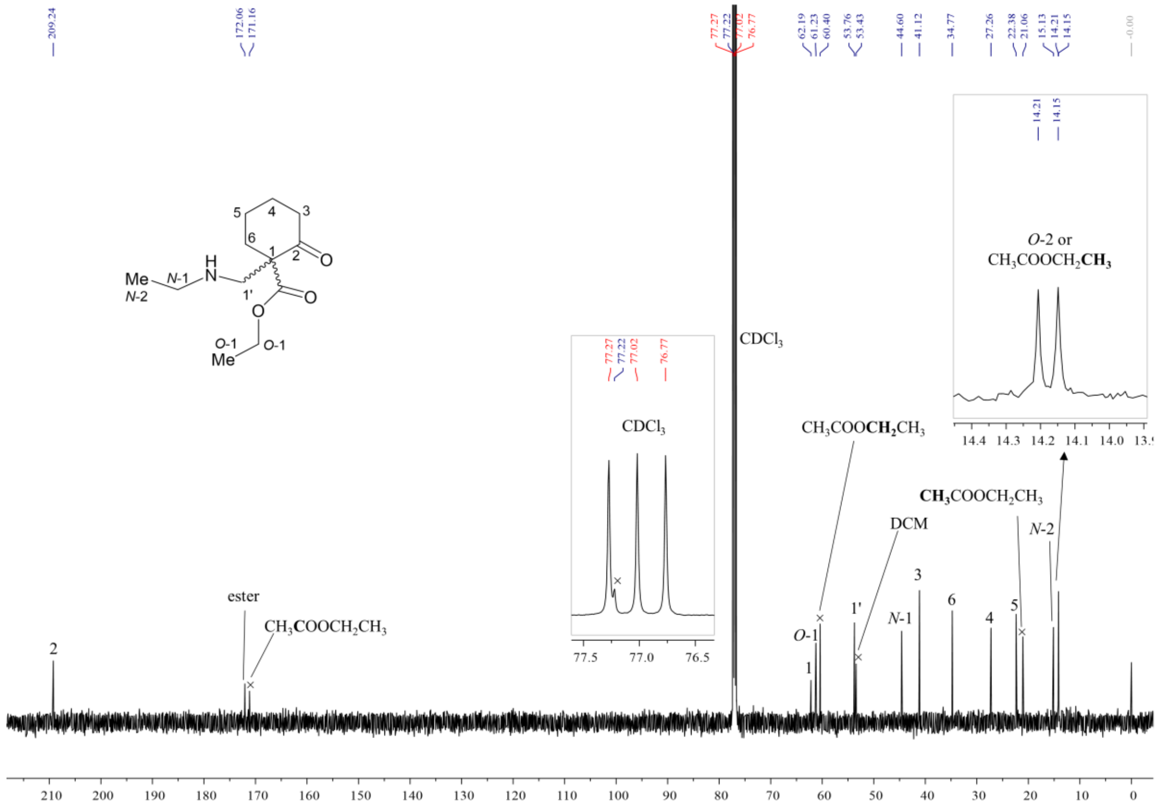

Figure S146. ^{13}C NMR spectrum of mono-Mannich product (**29**) in $CDCl_3$ with assignments (residual EtOAc is displayed)[1]

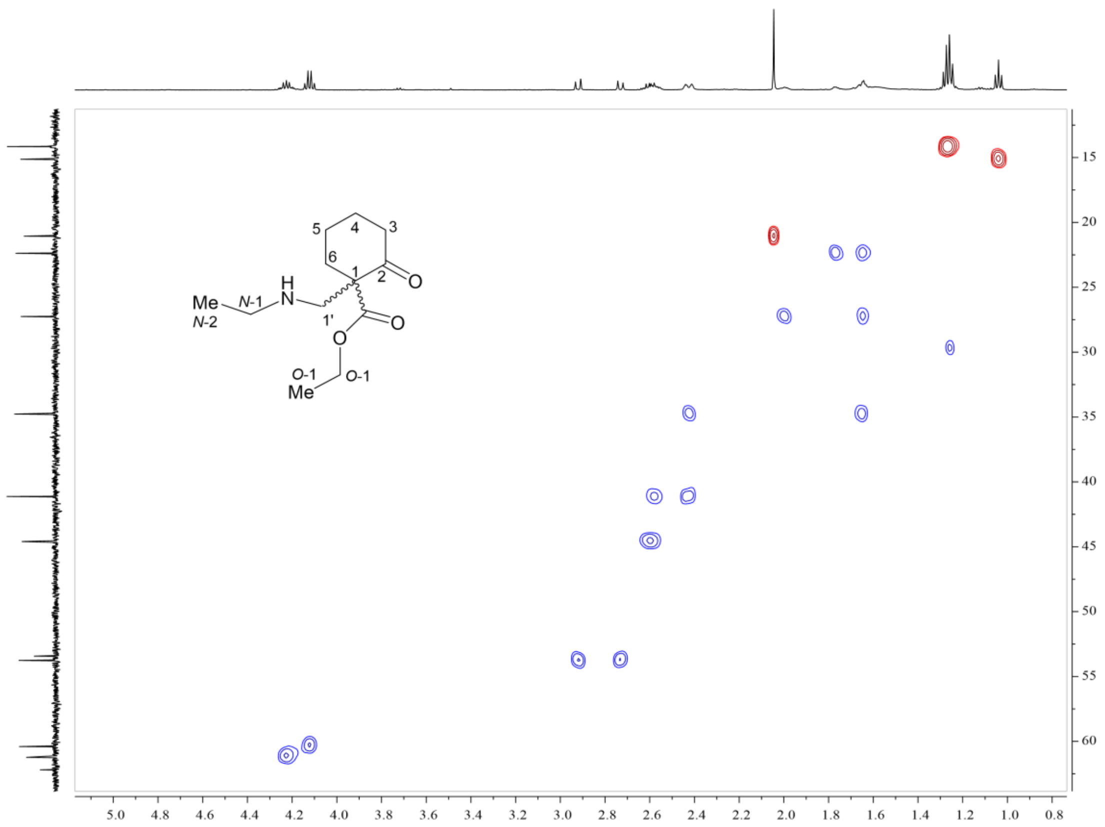

Figure S147. HSQC spectrum of mono-Mannich product (**29**) in $CDCl_3$

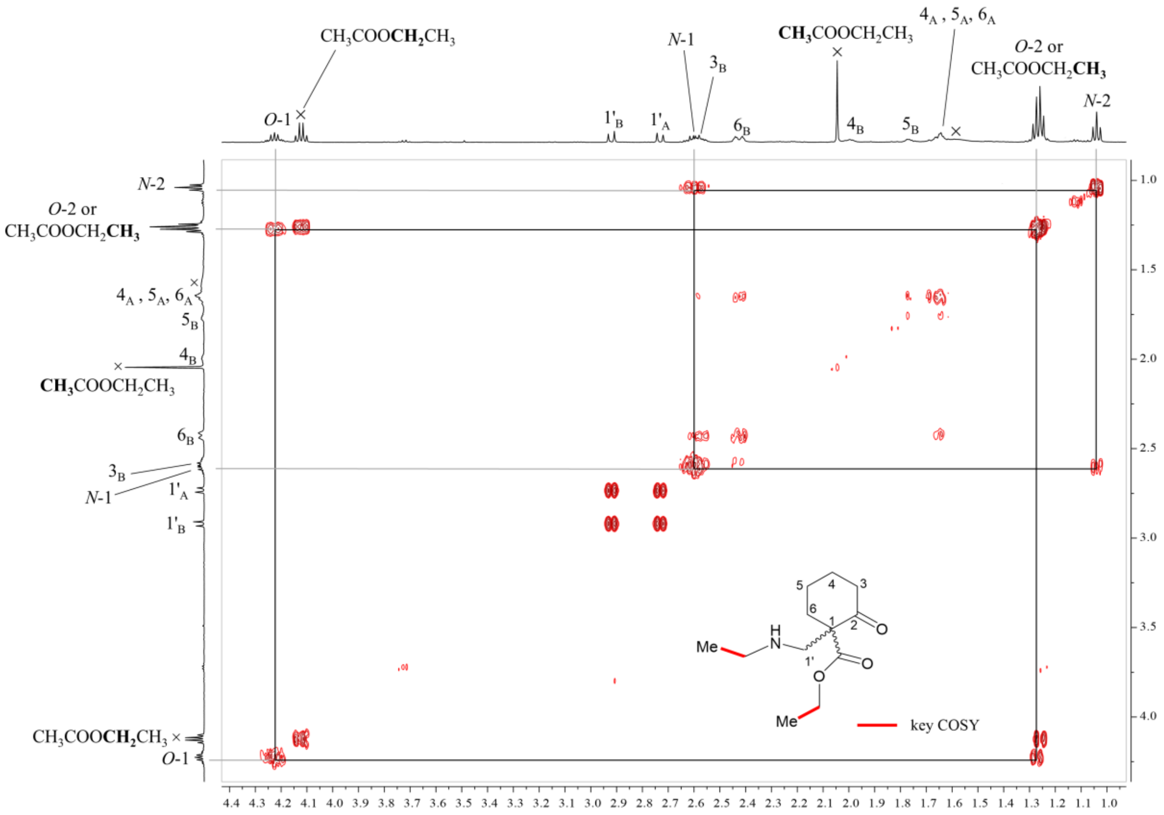

Figure S148. COSY spectrum of mono-Mannich product (**29**) in $CDCl_3$

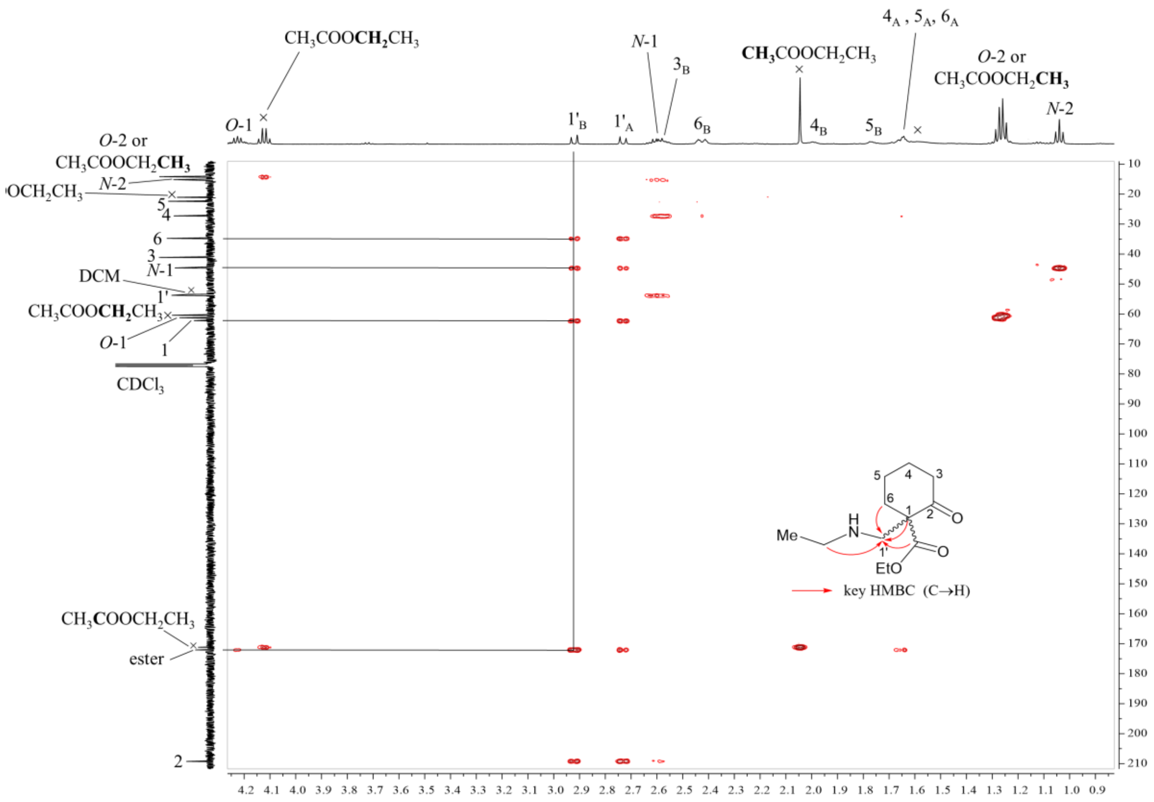

Figure S149. HMBC spectrum of mono-Mannich product (**29**) in $CDCl_3$

3-Ethyl-1-(hydroxymethyl)-3-azabicyclo[3.3.1]nonan-9-ol (**30**)

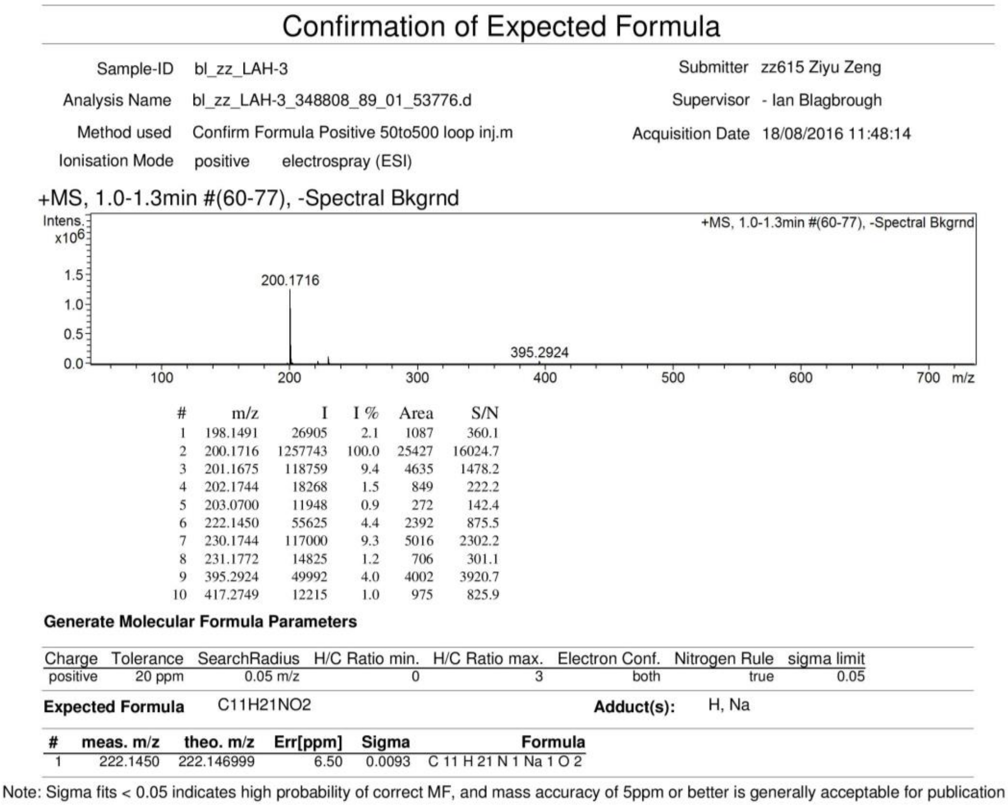

Confirmation of Expected Formula

Sample-ID	bl_zz_LAH-3	Submitter	zz615 Ziyu Zeng
Analysis Name	bl_zz_LAH-3_348808_89_01_53776.d	Supervisor	- Ian Blagbrough
Method used	Confirm Formula Positive 50to500 loop inj.m	Acquisition Date	18/08/2016 11:48:14
Ionisation Mode	positive electrospray (ESI)		

#	m/z	I	I %	Area	S/N
1	198.1491	26905	2.1	1087	360.1
2	200.1716	1257743	100.0	25427	16024.7
3	201.1675	118759	9.4	4635	1478.2
4	202.1744	18268	1.5	849	222.2
5	203.0700	11948	0.9	272	142.4
6	222.1450	55625	4.4	2392	875.5
7	230.1744	117000	9.3	5016	2302.2
8	231.1772	14825	1.2	706	301.1
9	395.2924	49992	4.0	4002	3920.7
10	417.2749	12215	1.0	975	825.9

Generate Molecular Formula Parameters

Charge	Tolerance	SearchRadius	H/C Ratio min.	H/C Ratio max.	Electron Conf.	Nitrogen Rule	sigma limit
positive	20 ppm	0.05 m/z	0	3	both	true	0.05

Expected Formula C11H21NO2 **Adduct(s):** H, Na

#	meas. m/z	theo. m/z	Err[ppm]	Sigma	Formula
1	222.1450	222.146999	6.50	0.0093	C 11 H 21 N 1 Na 1 O 2

Note: Sigma fits < 0.05 indicates high probability of correct MF, and mass accuracy of 5ppm or better is generally acceptable for publication

Figure S150. MS data of diol (**30**)

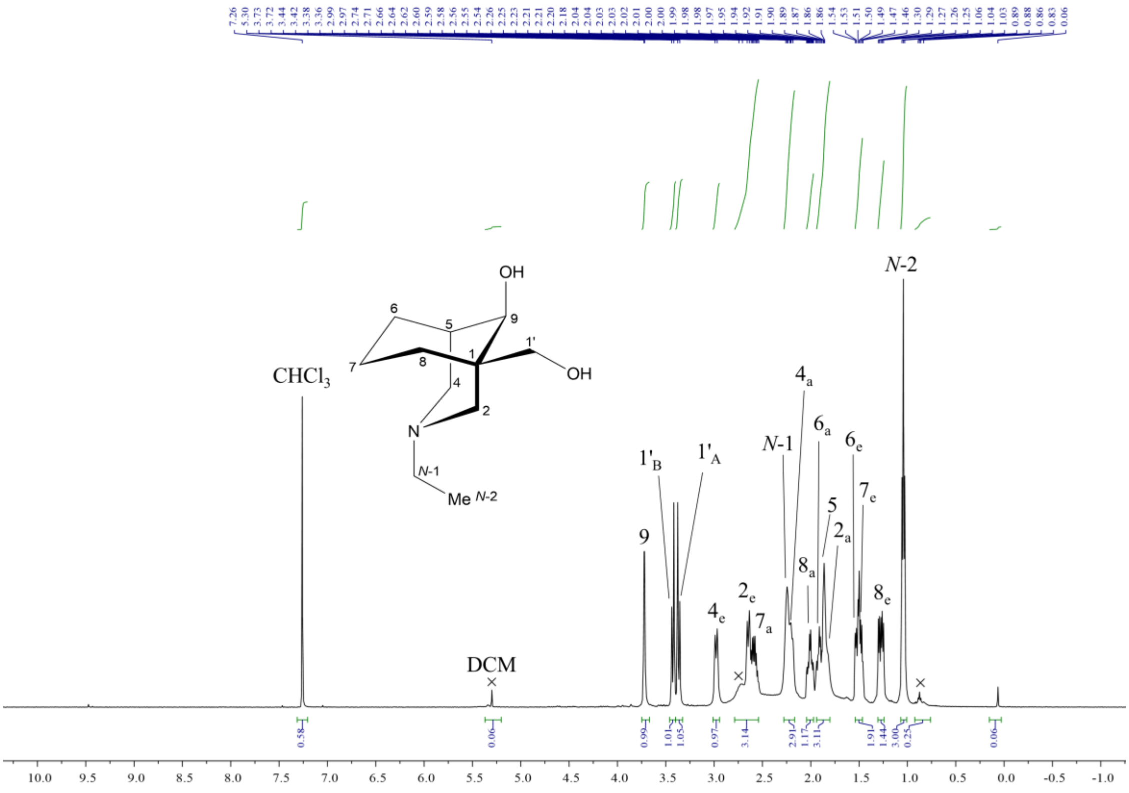

Figure S151. ^{1}H NMR spectrum of diol (**30**) in $CDCl_3$ with assignments (residual DCM is displayed)[1]

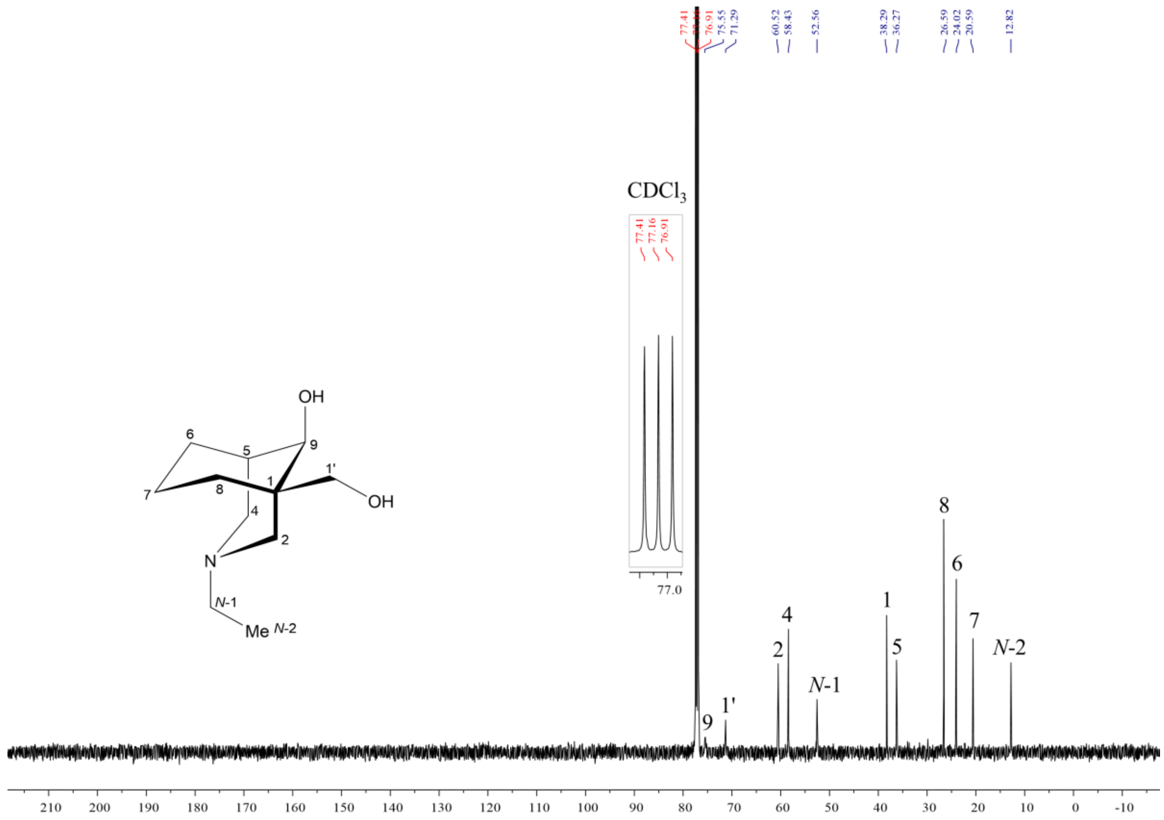

Figure S152. ^{13}C NMR spectrum of diol (**30**) in $CDCl_3$ with assignments

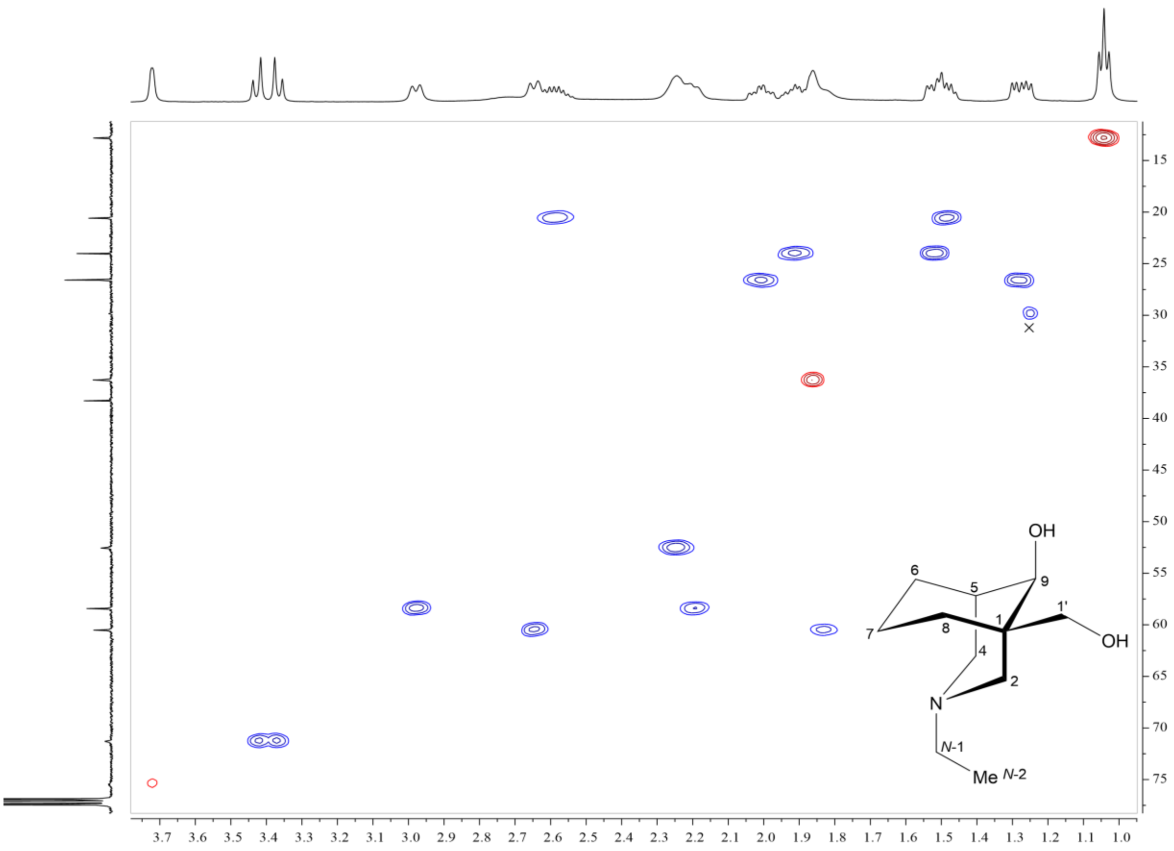

Figure S153. HSQC spectrum of diol (**30**) in $CDCl_3$

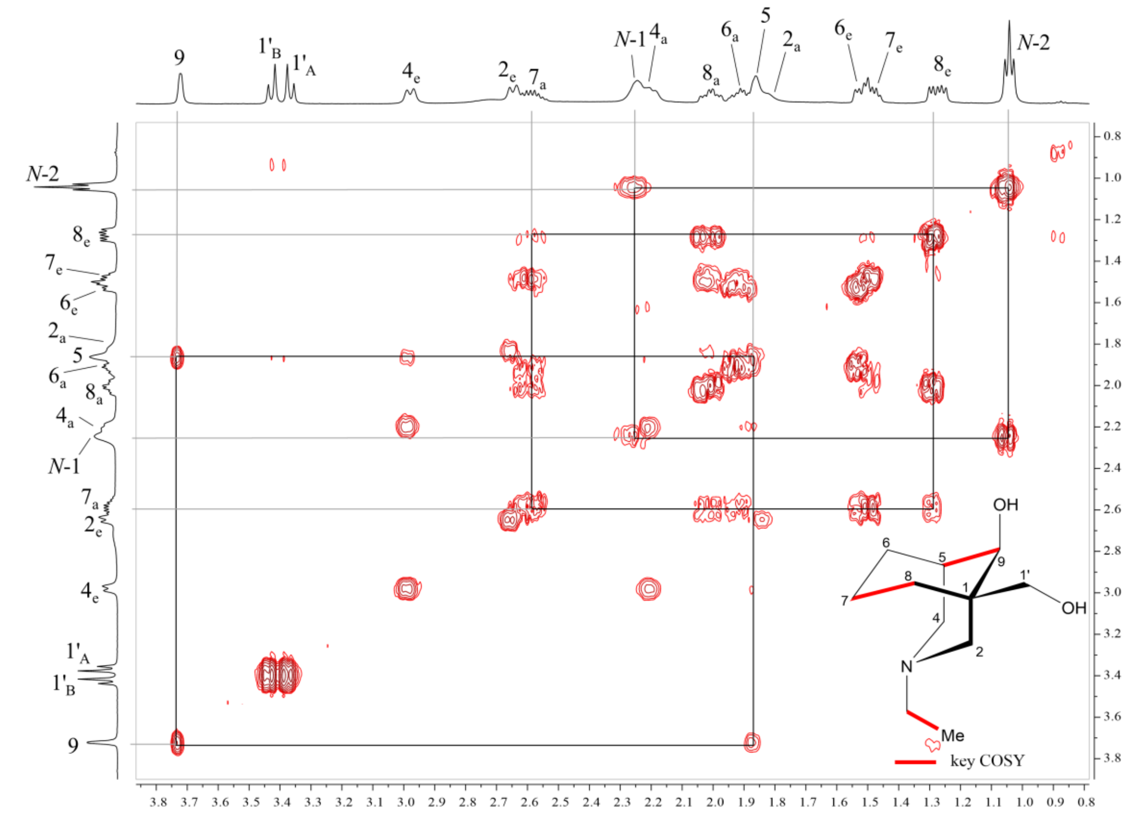

Figure S154. COSY spectrum of diol (**30**) in $CDCl_3$

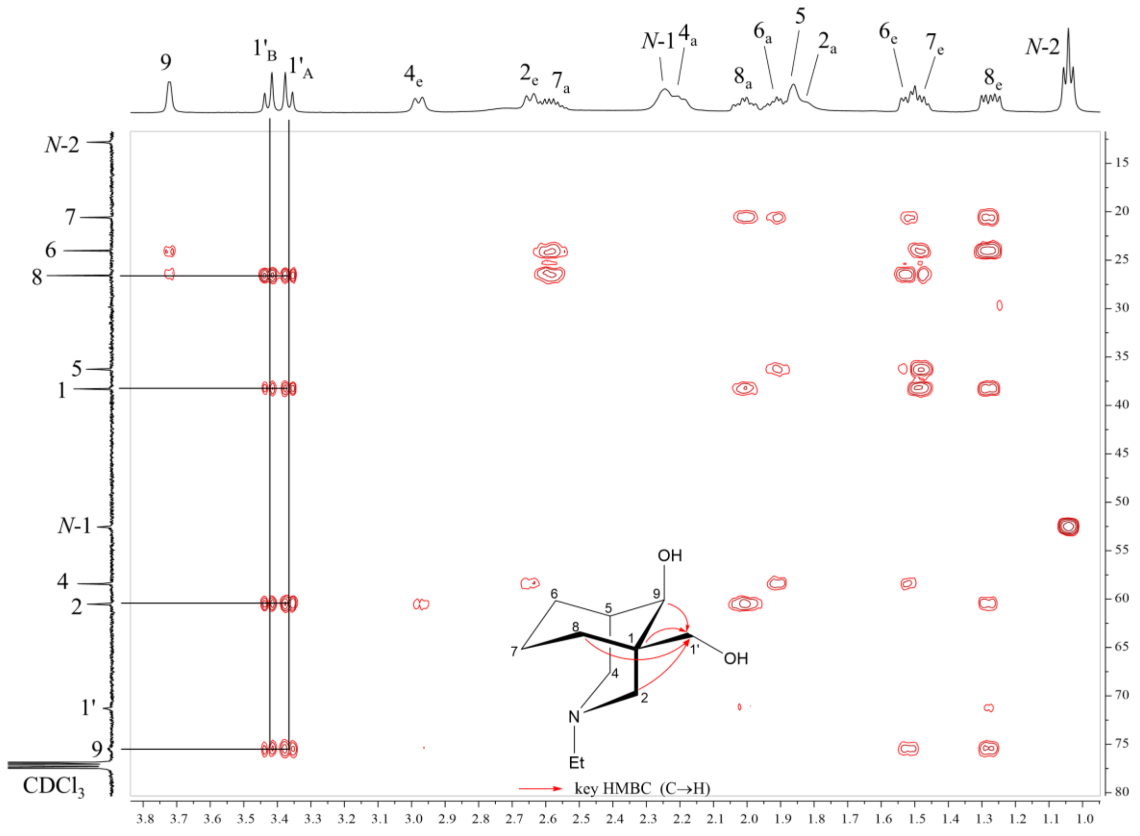

Figure S155. HMBC spectrum of diol (**30**) in $CDCl_3$

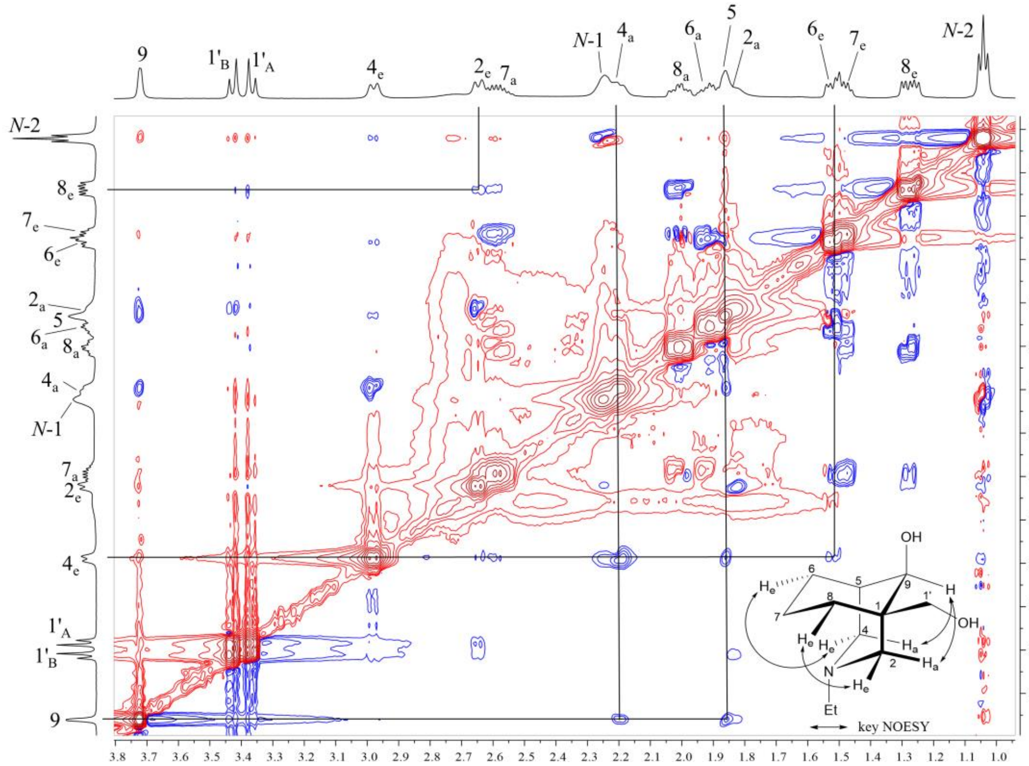

Figure S156. NOESY spectrum of diol (**30**) in $CDCl_3$

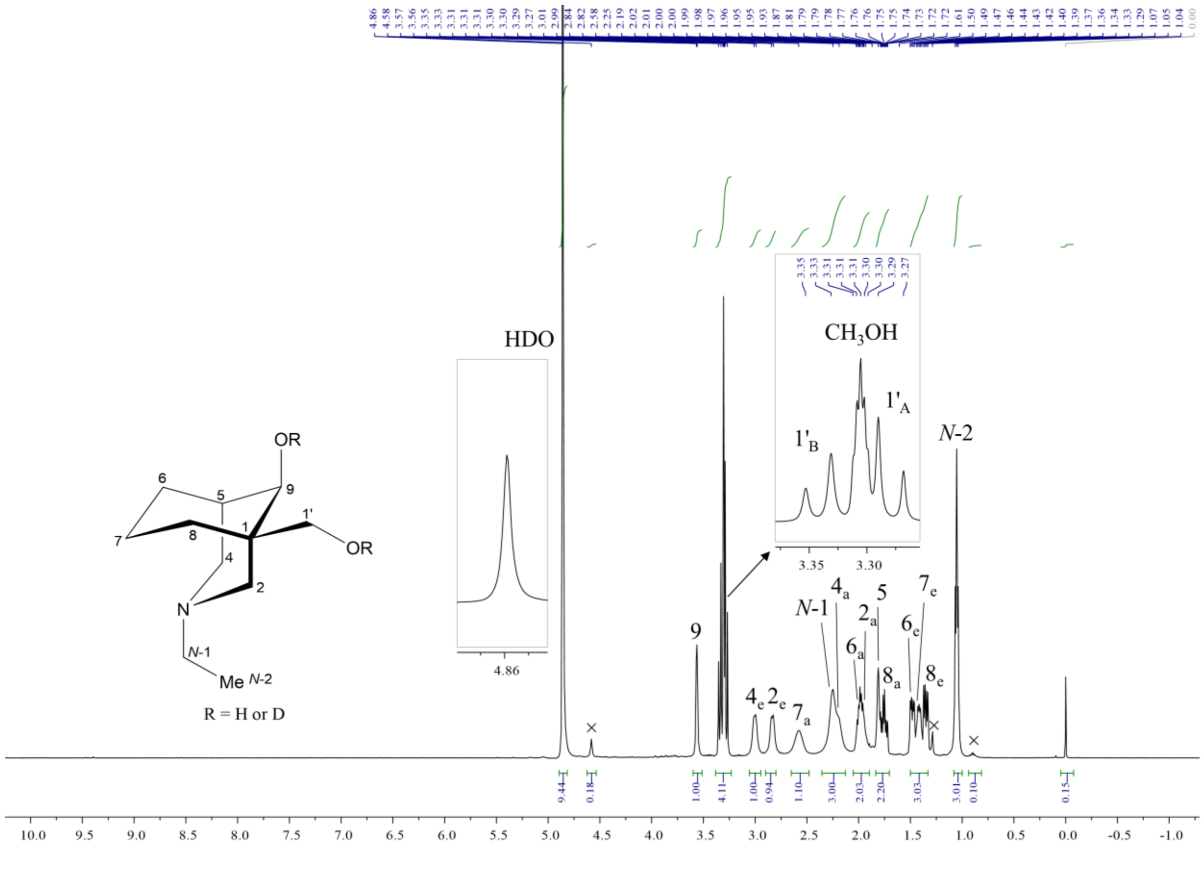

Figure S157. ^1H NMR spectrum of diol (**30**) in CD_3OD with assignments

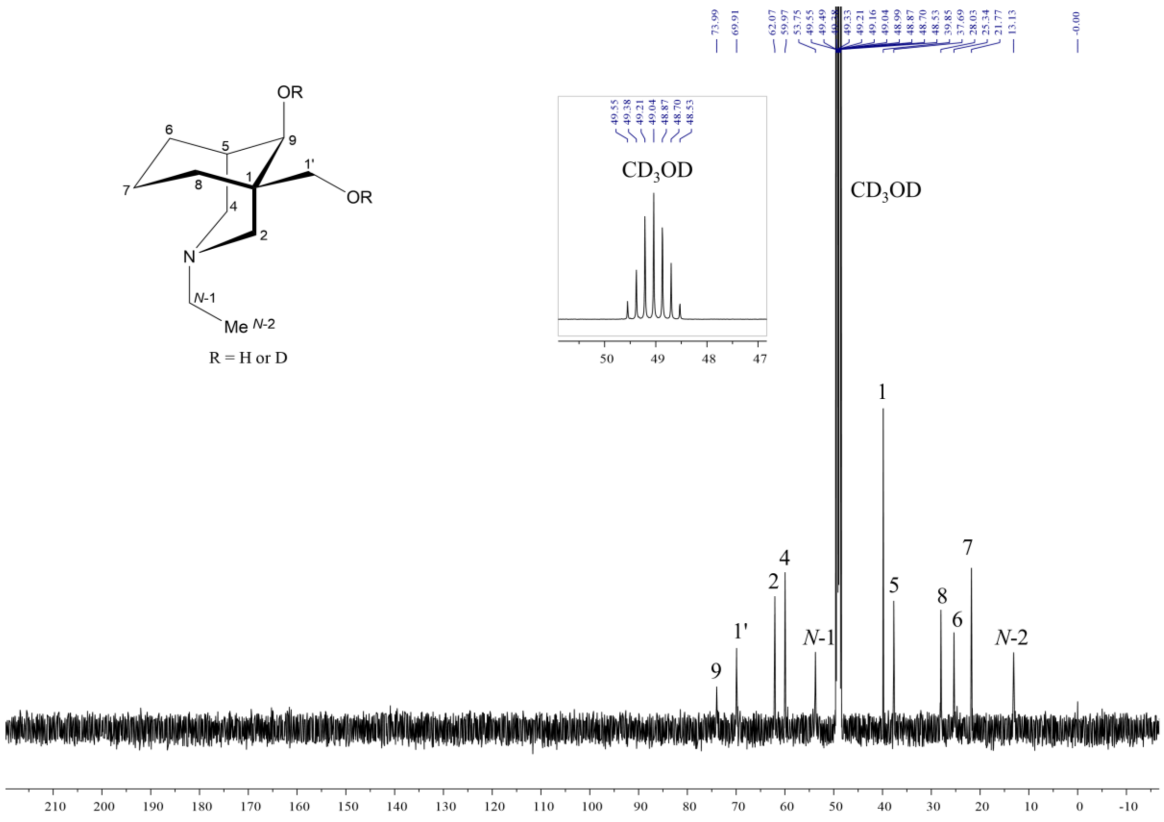

Figure S158. ^{13}C NMR spectrum of diol (**30**) in CD_3OD with assignments

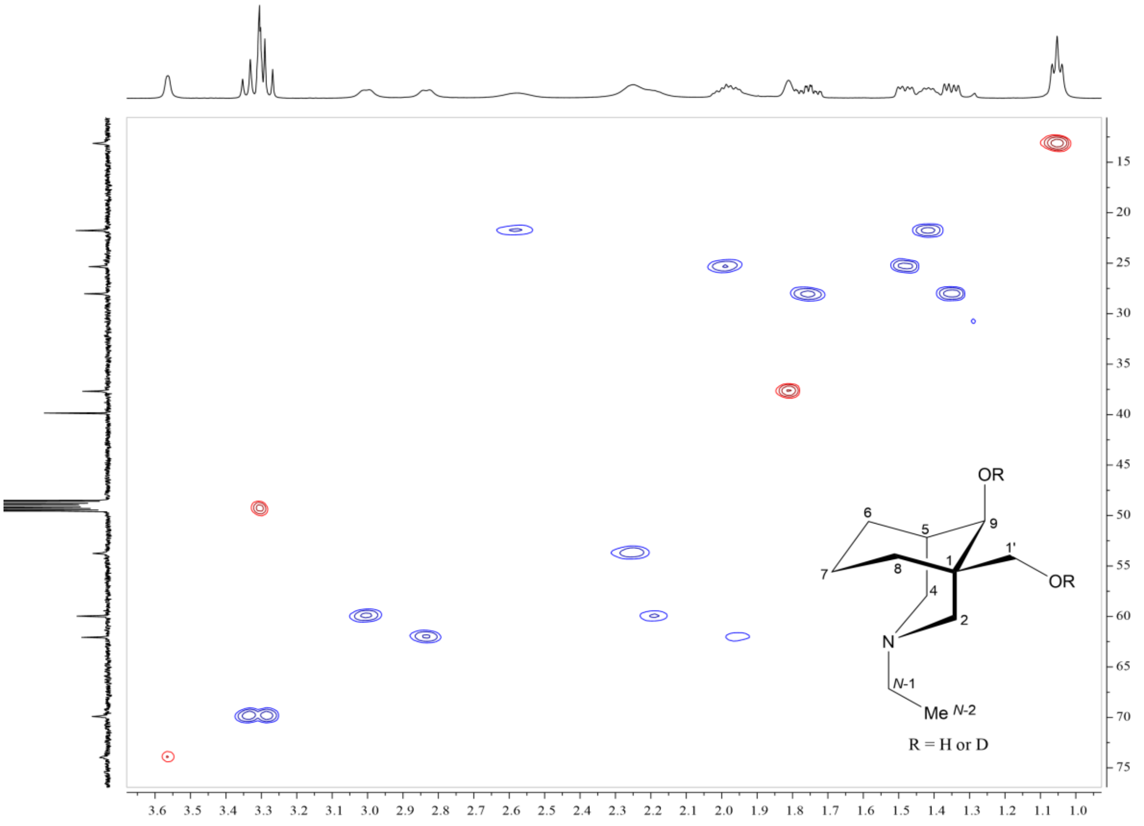

Figure S159. HSQC spectrum of diol (**30**) in CD_3OD

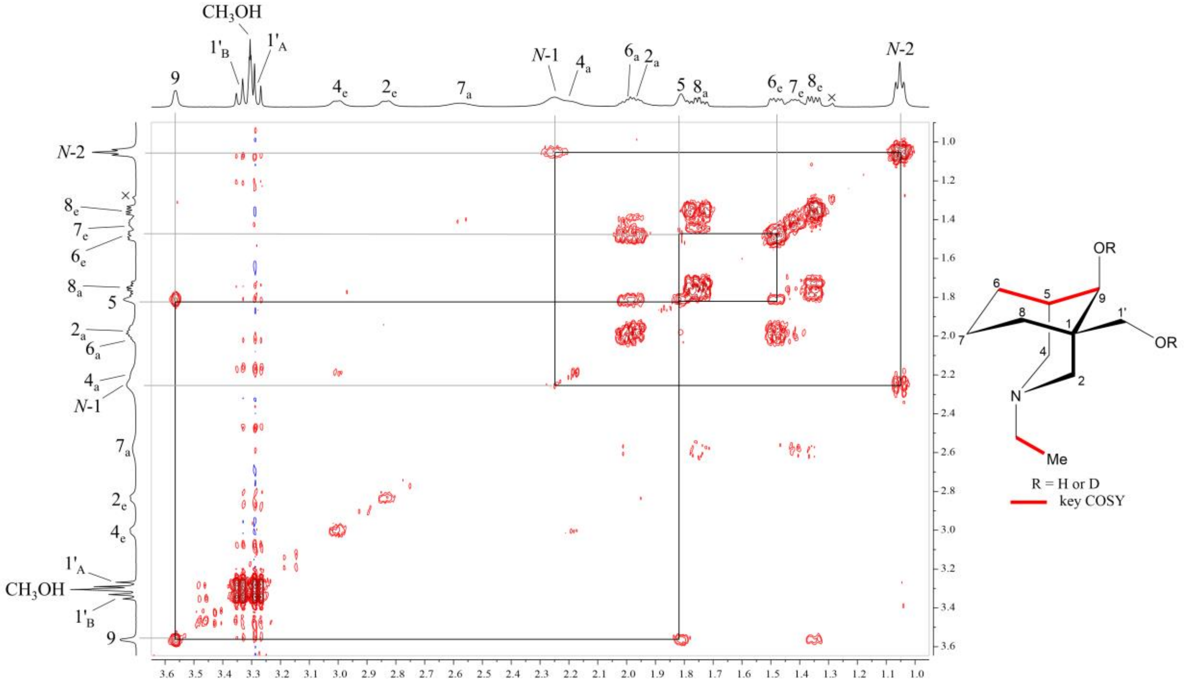

Figure S160. COSY spectrum of diol (**30**) in CD_3OD

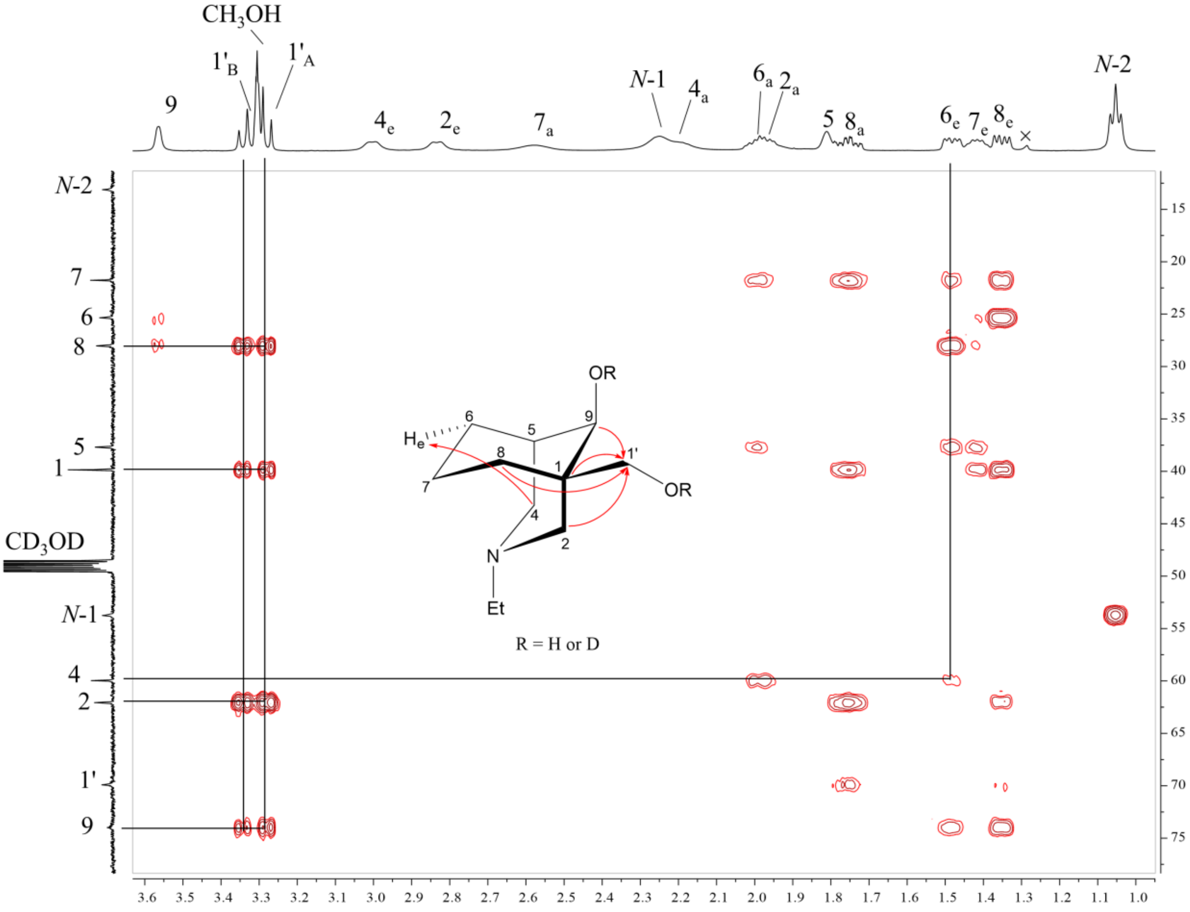

Figure S161. HMBC spectrum of diol (**30**) in CD_3OD

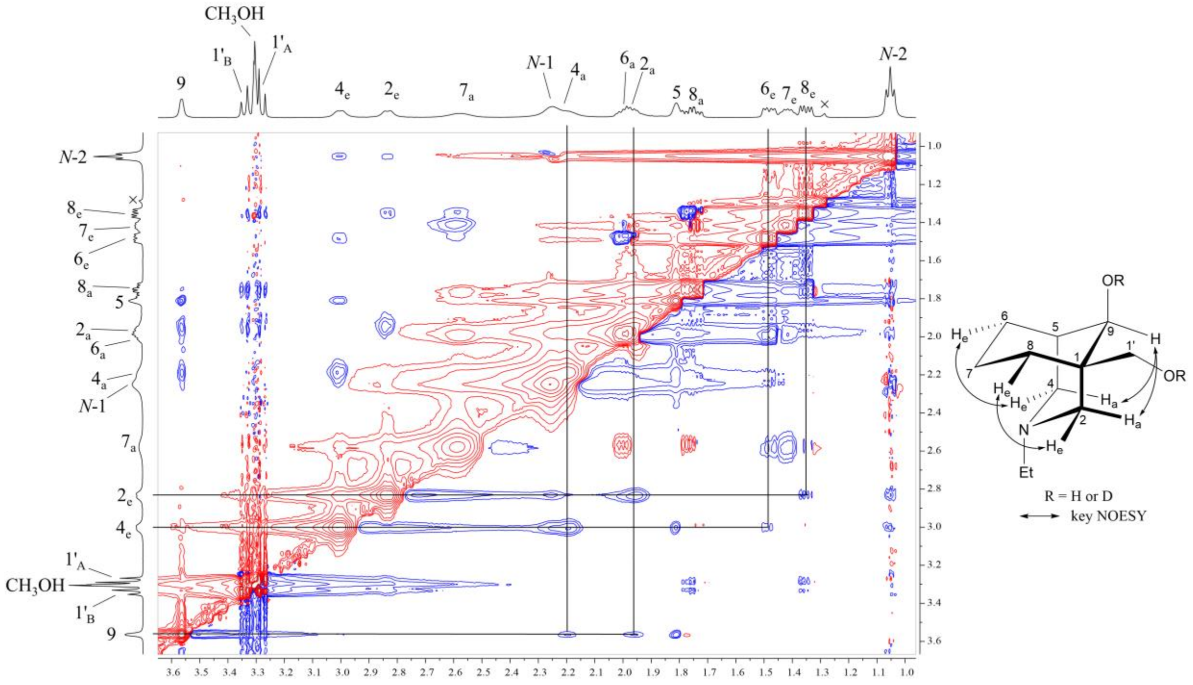

Figure S162. NOESY spectrum of diol (**30**) in CD_3OD

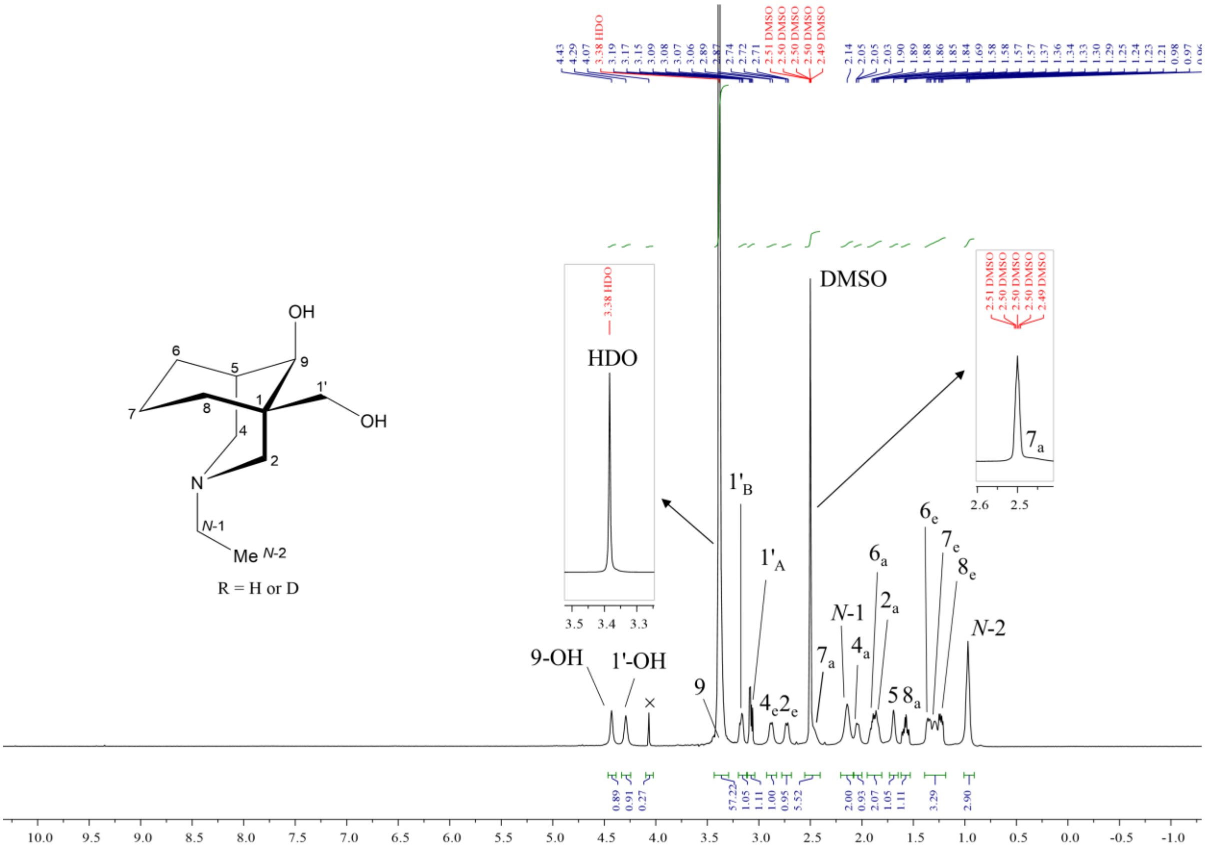

Figure S163. ^{1}H NMR spectrum of diol (**30**) in d_6-DMSO (with 2 drops of D_2O) with assignments

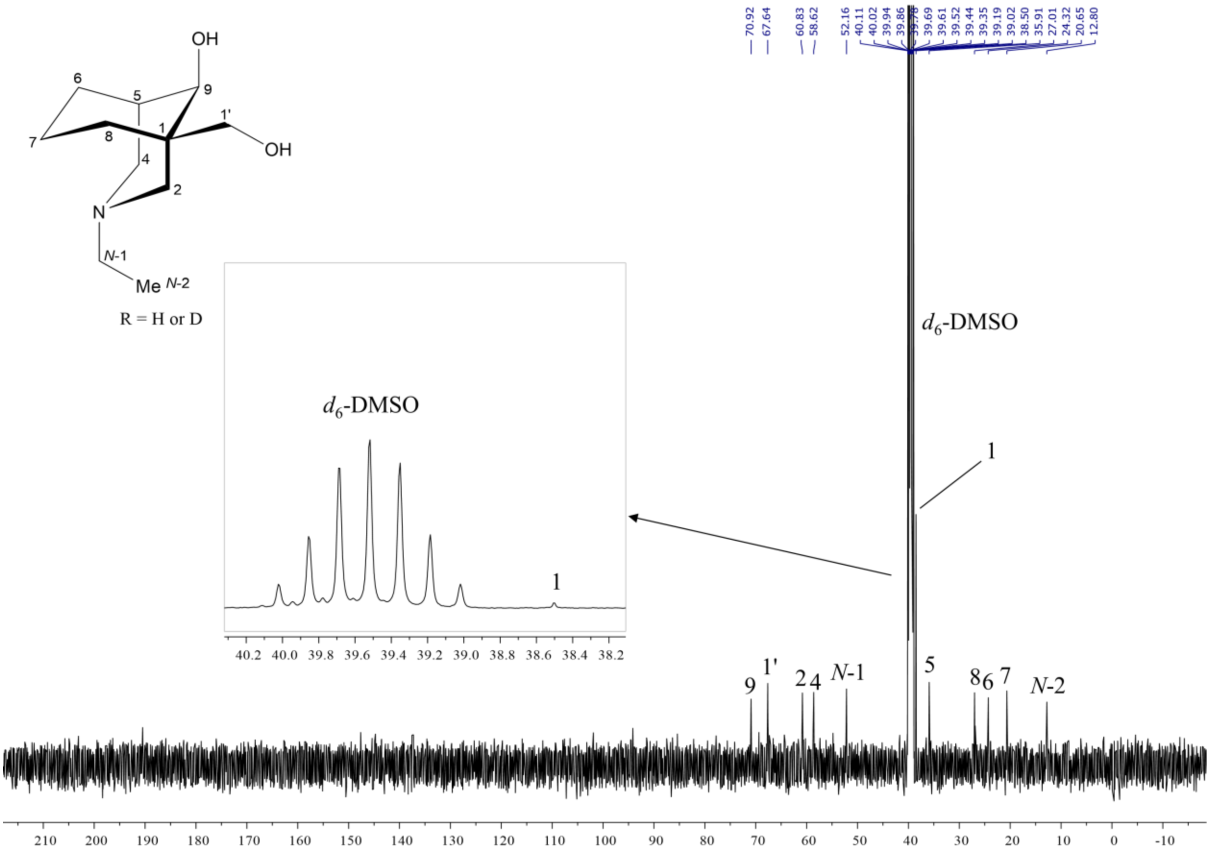

Figure S164. ^{13}C NMR spectrum of diol (**30**) in d_6-DMSO (with 2 drops of D_2O) with assignments

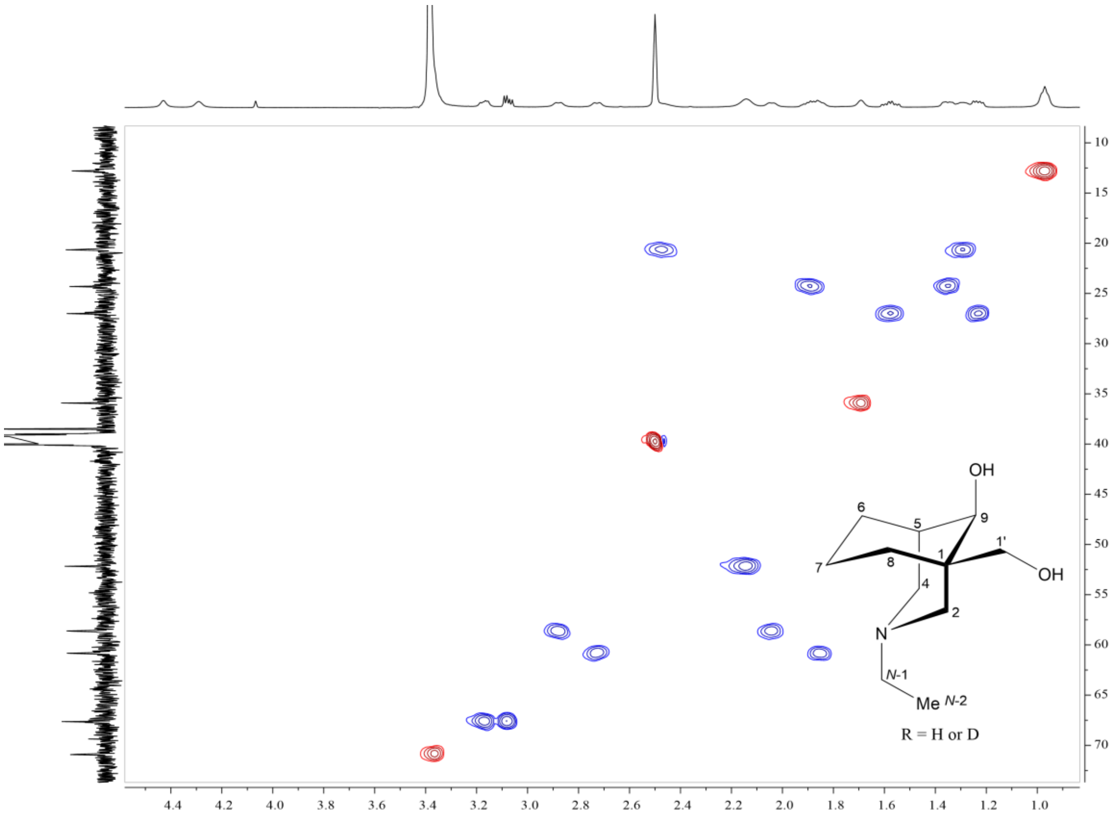

Figure S165. HSQC spectrum of diol (**30**) in d_6-DMSO (with 2 drops of D_2O)

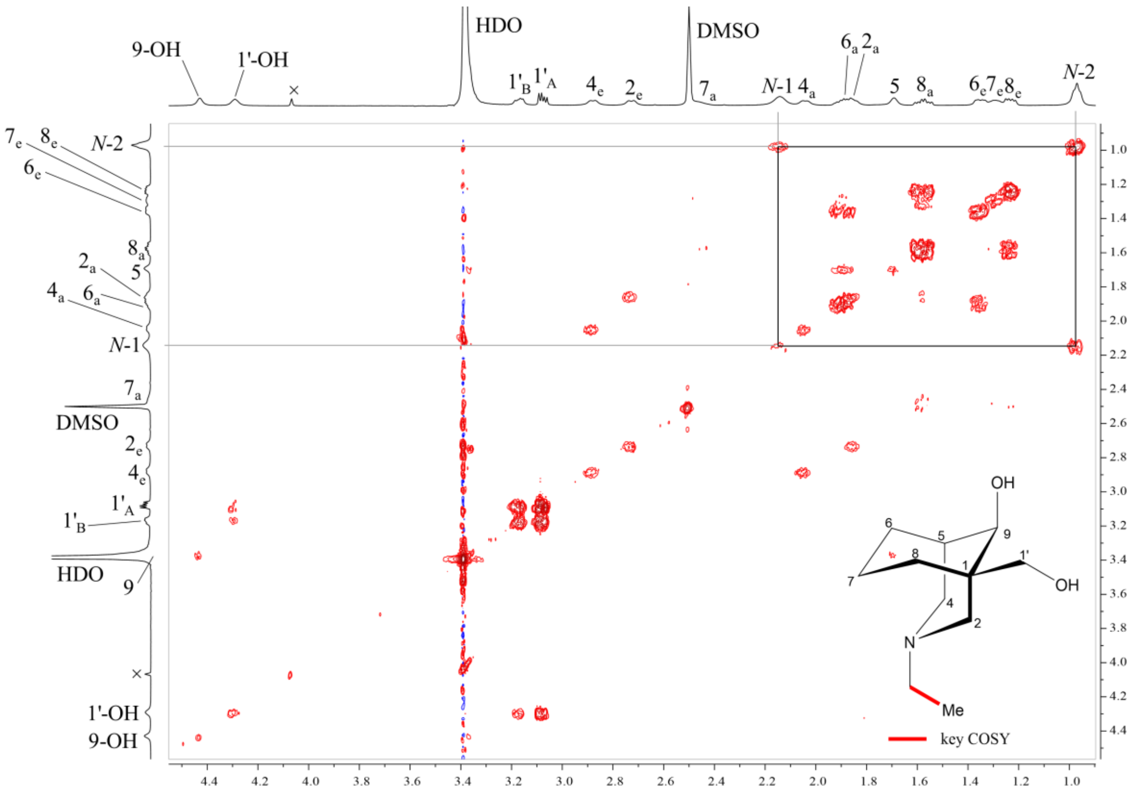

Figure S166. COSY spectrum of diol (**30**) in d_6-DMSO (with 2 drops of D_2O)

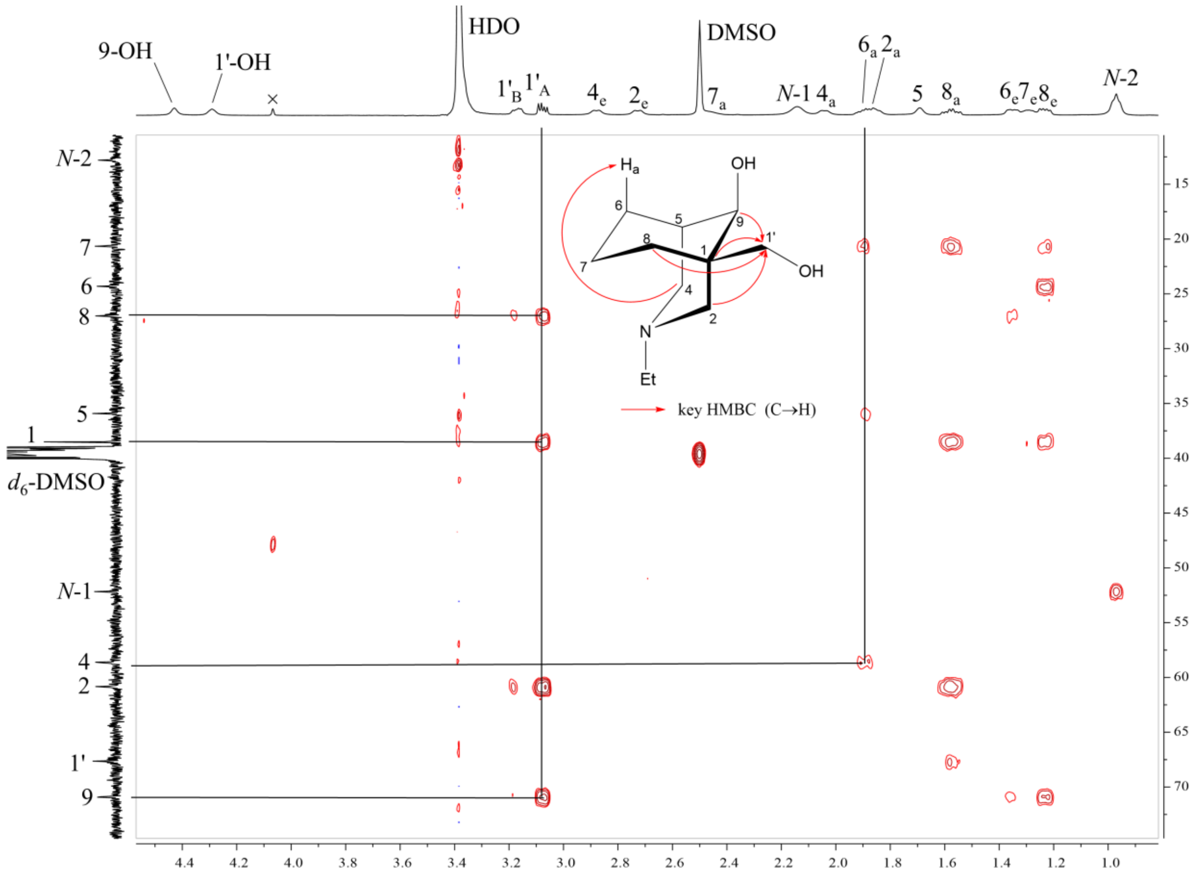

Figure S167. HMBC spectrum of diol (**30**) in d_6-DMSO (with 2 drops of D_2O)

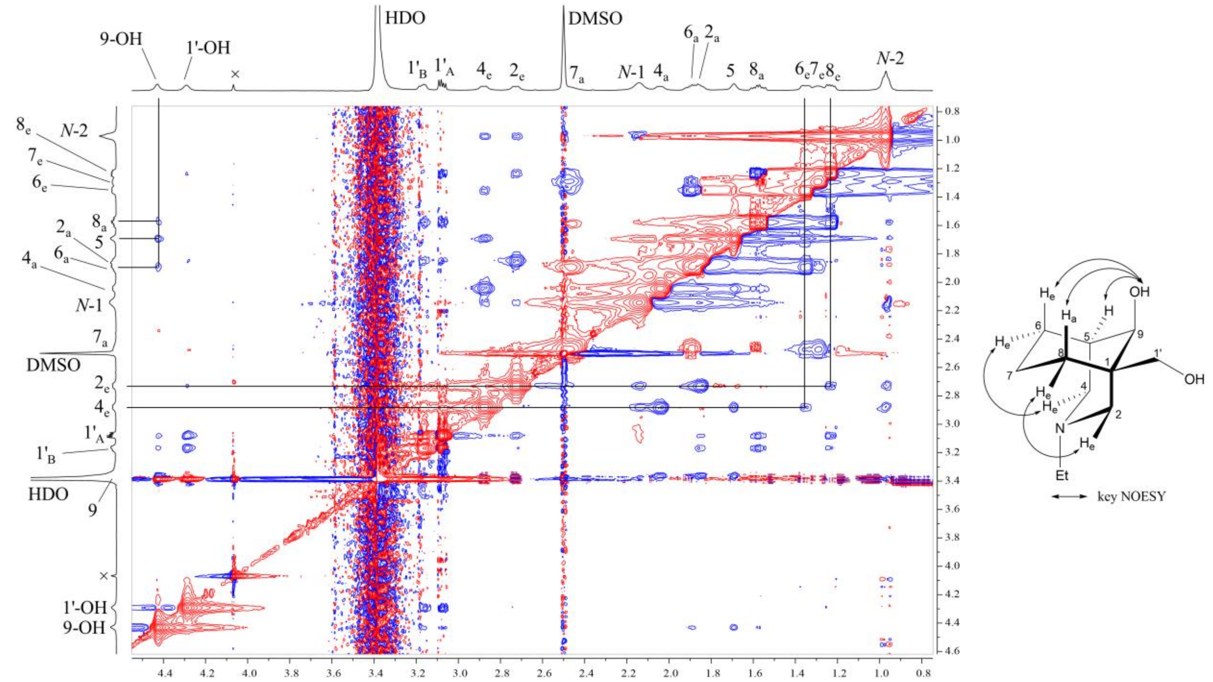

Figure S168. NOESY spectrum of diol (**30**) in d_6-DMSO (with 2 drops of D_2O)

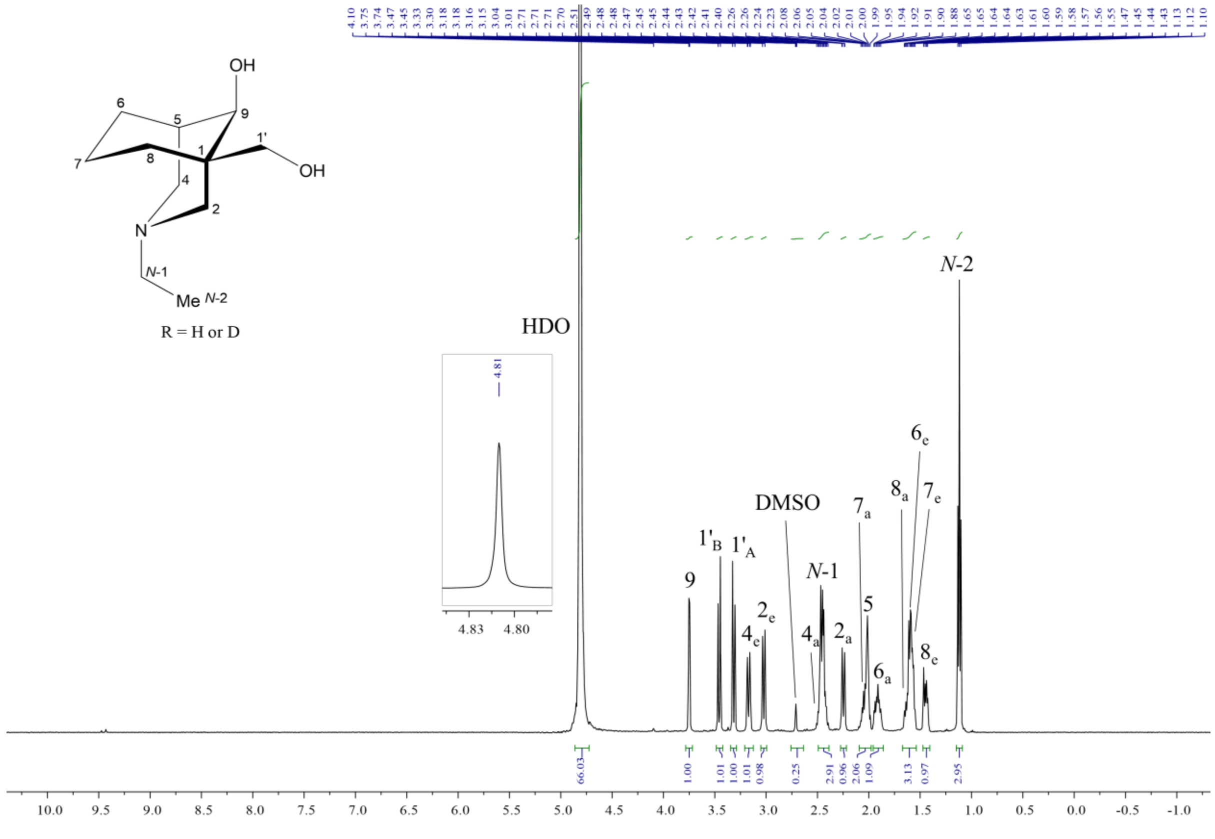

Figure S169. ^{1}H NMR spectrum of diol (**30**) in D_2O with assignments (with 2 drops of d_6-DMSO, residual DMSO is shown at 2.71 ppm, HDO residual is shown at 4.81 ppm)[1,2]

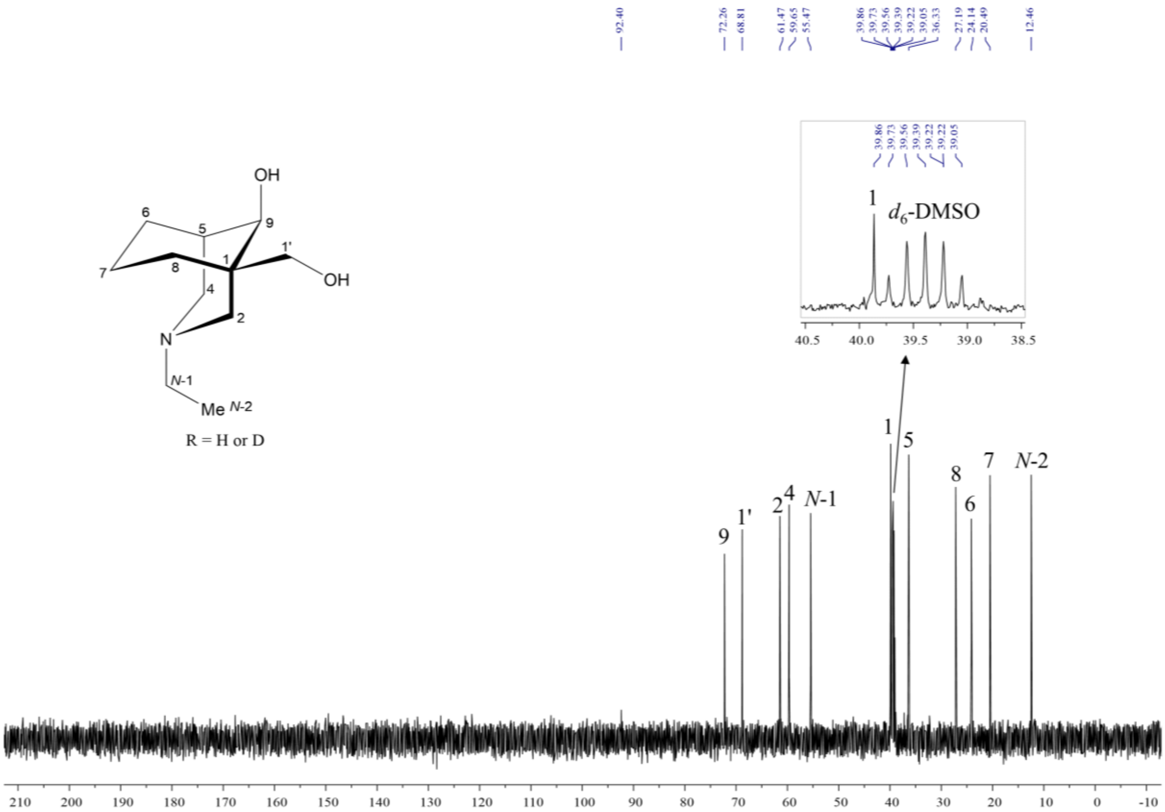

Figure S170. ^{13}C NMR spectrum of diol (**30**) in D_2O with assignments (with 2 drops of d_6-DMSO, d_6-DMSO solvent peak is shown at 39.39 ppm)[1]

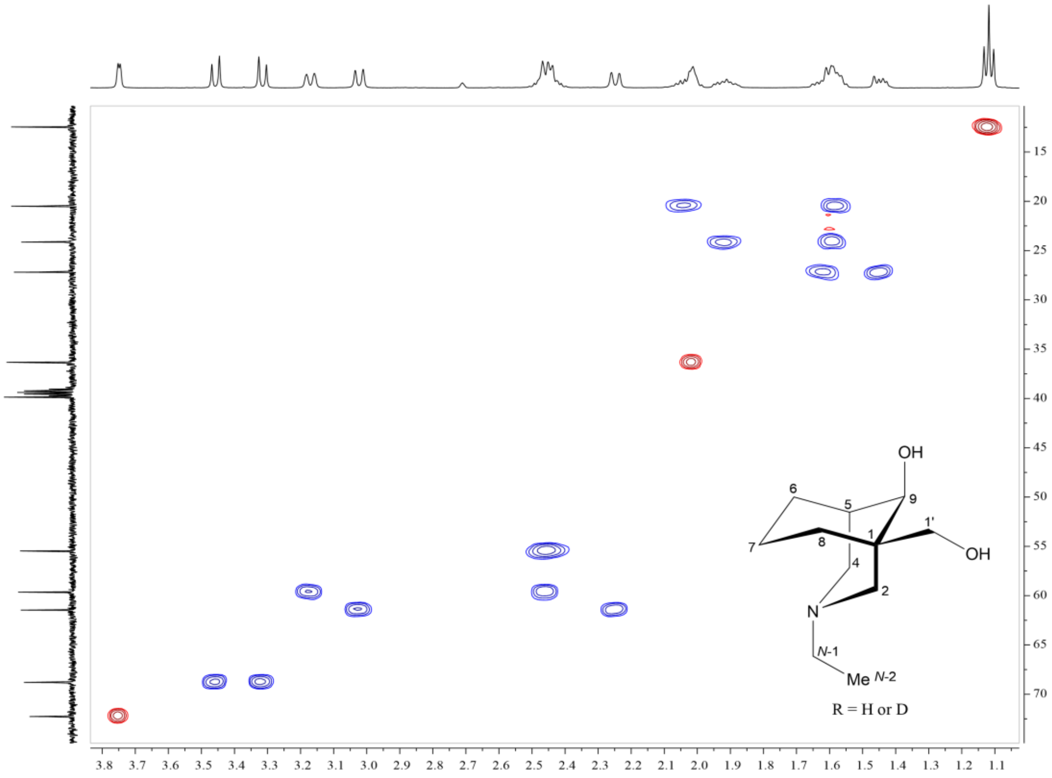

Figure S171. HSQC spectrum of diol (**30**) in D_2O (with 2 drops of d_6-DMSO)

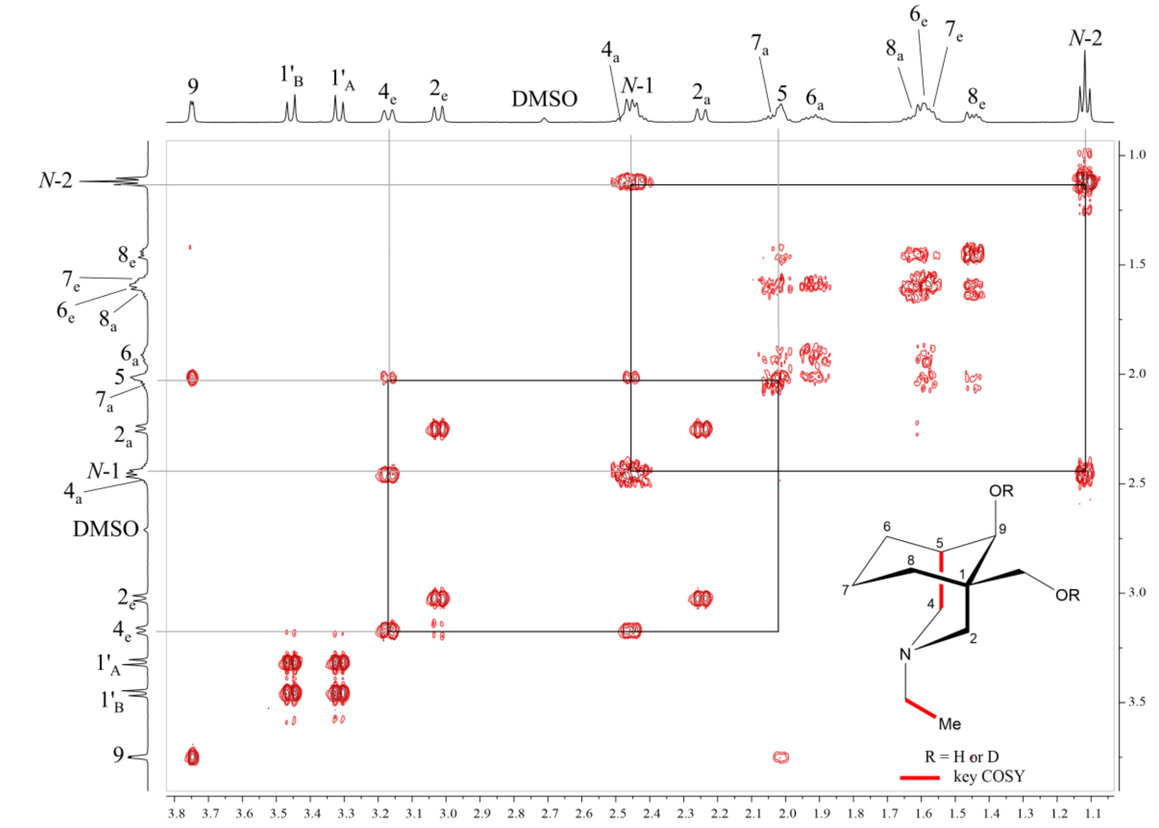

Figure S172. COSY spectrum of diol (**30**) in D_2O (with 2 drops of d_6-DMSO)

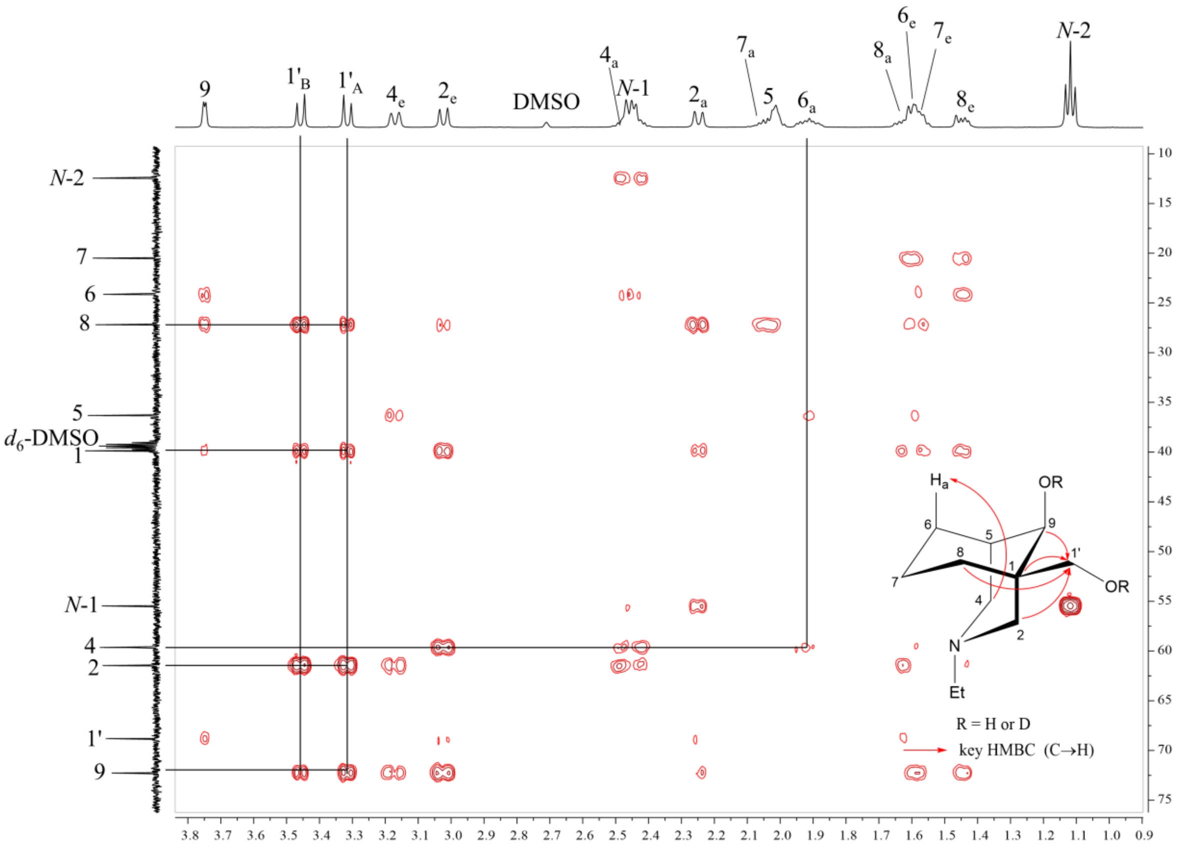

Figure S173. HMBC spectrum of diol (**30**) in D_2O (with 2 drops of d_6-DMSO)

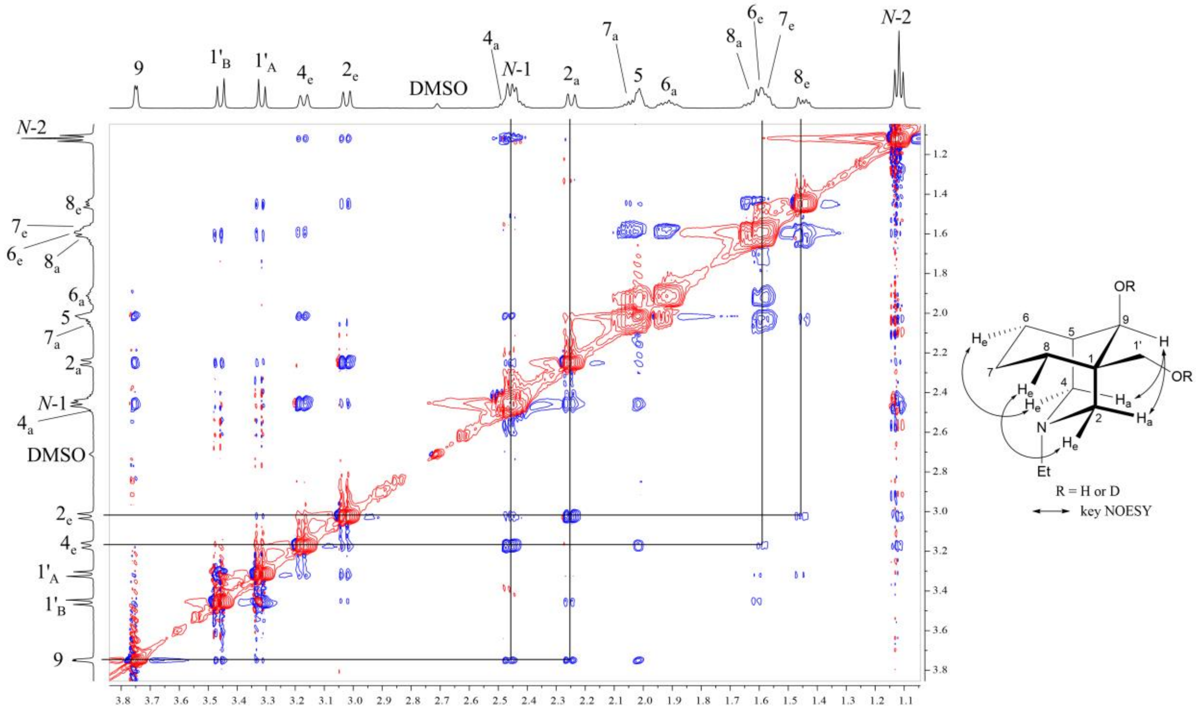

Figure S174. NOESY spectrum of diol (**30**) in D_2O (with 2 drops of d_6-DMSO)

1-(Ethoxycarbonyl)-3-ethyl-9-oxo-3-azabicyclo[3.3.1]nonan-3-ium acetate salt (**31**)

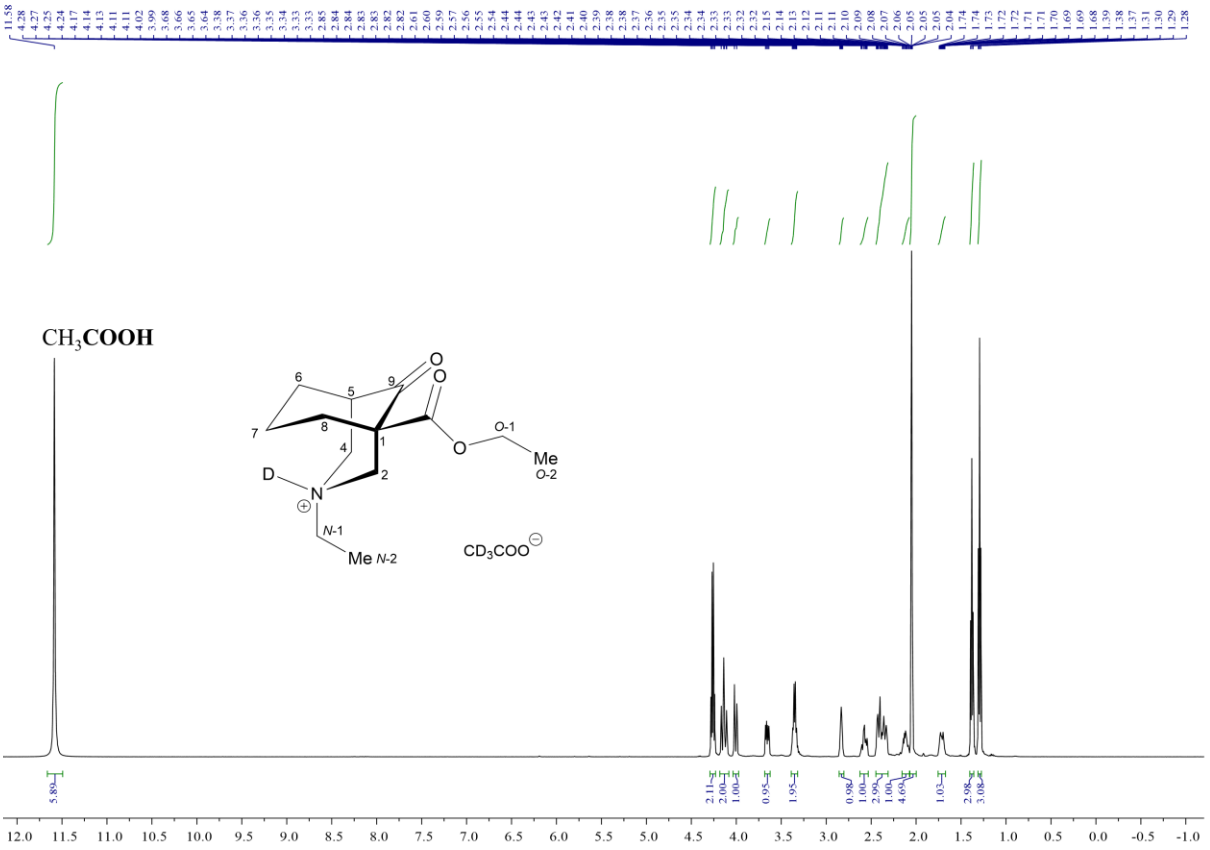

Figure S175. ^{1}H NMR spectrum of ketone acetate-salt (**31**) in d_4-acetic acid

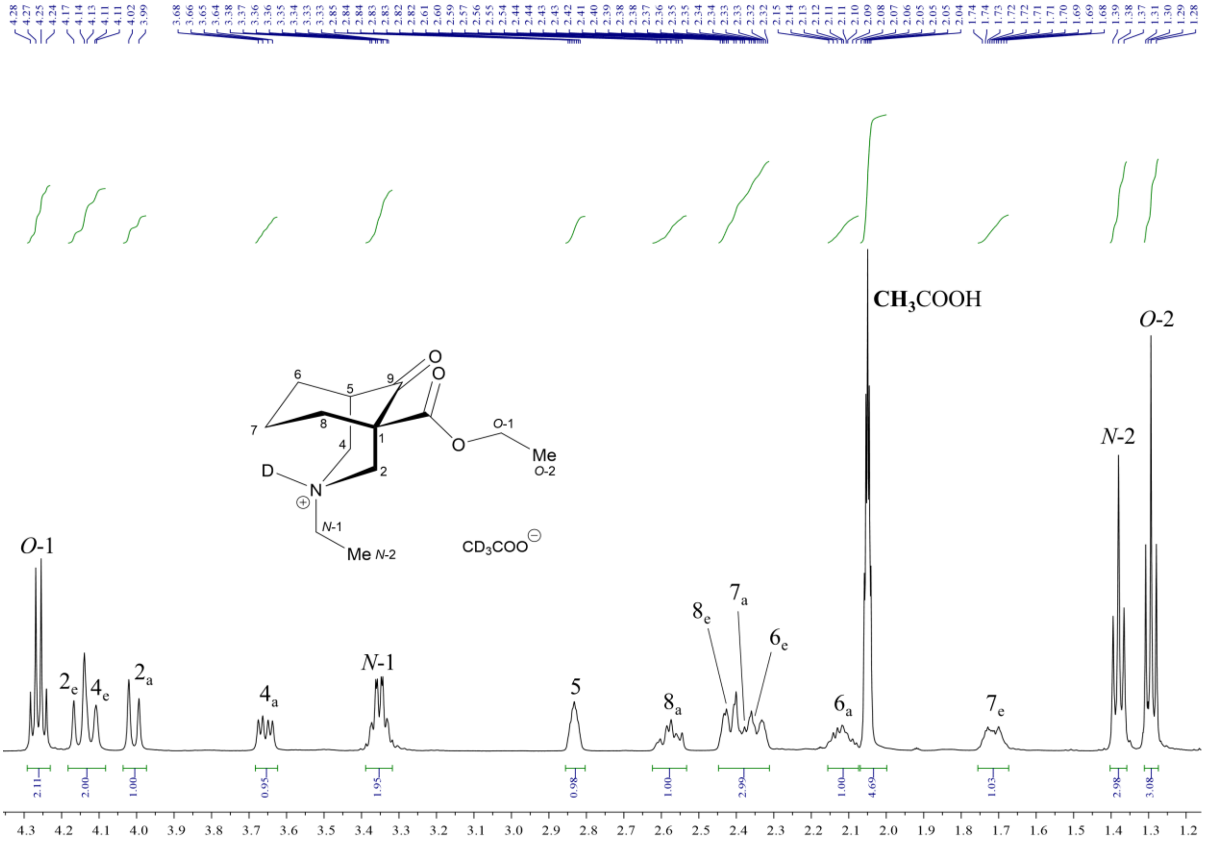

Figure S176. ^{1}H NMR expansion of ketone acetate-salt (**31**) in d_4-acetic acid with assignments

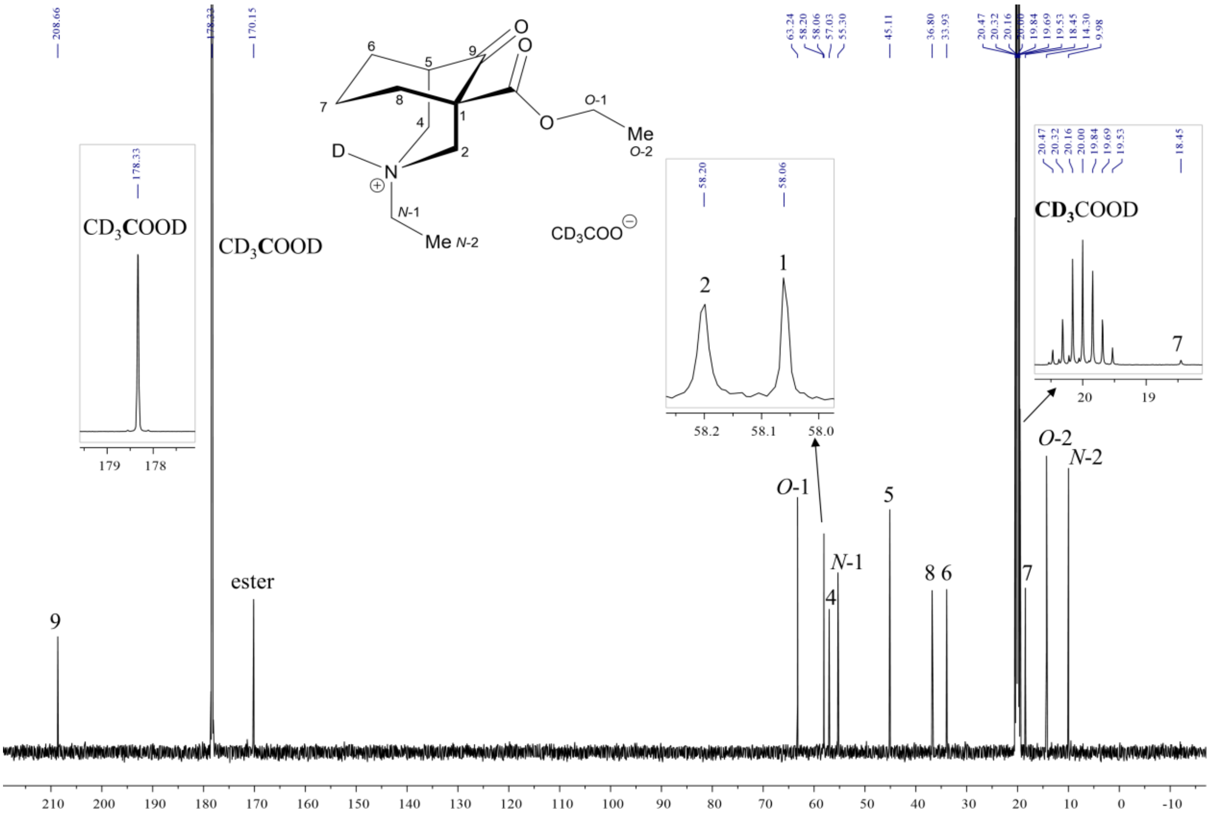

Figure S177. ^{13}C NMR spectrum of ketone acetate-salt (**31**) in d_4-acetic acid

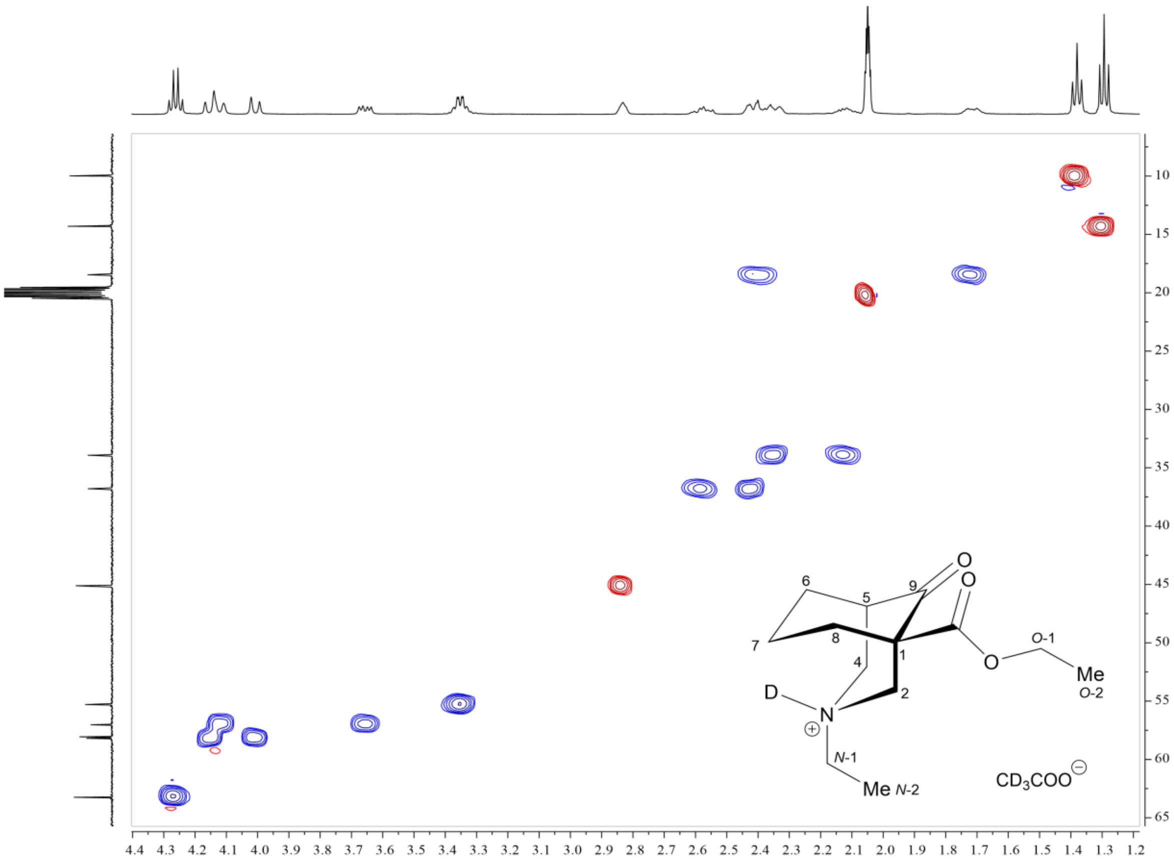

Figure S178. HSQC spectrum of ketone acetate-salt (**31**) in d_4-acetic acid

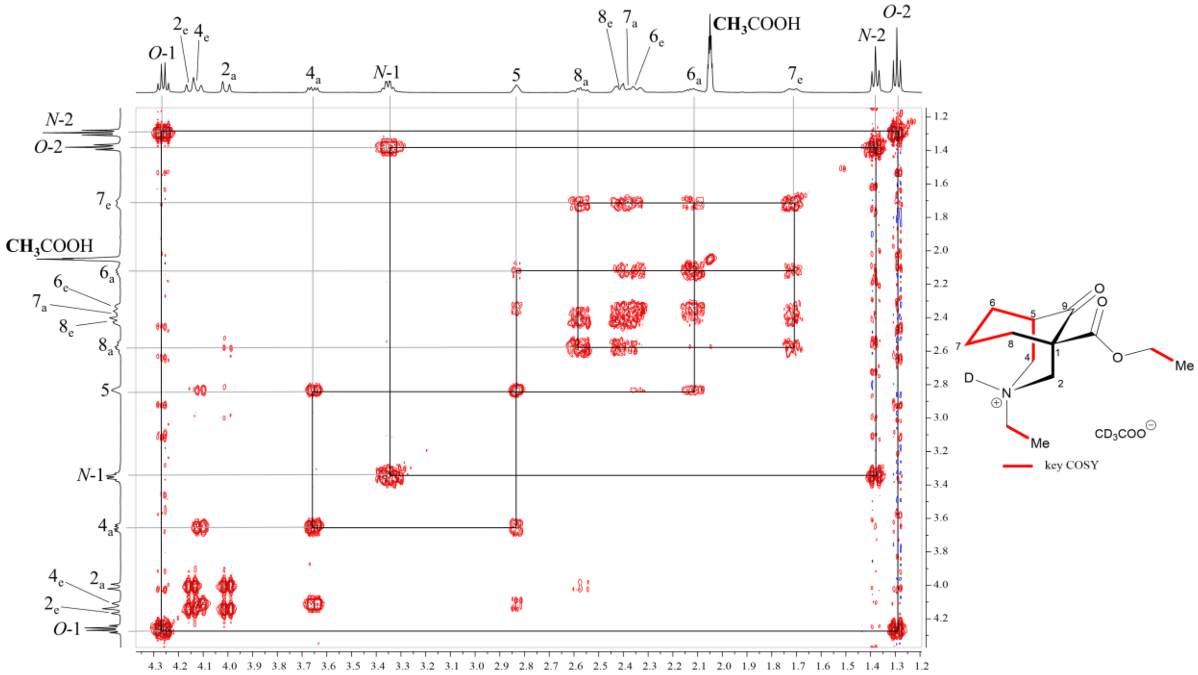

Figure S179. COSY spectrum of ketone acetate-salt (**31**) in d_4-acetic acid

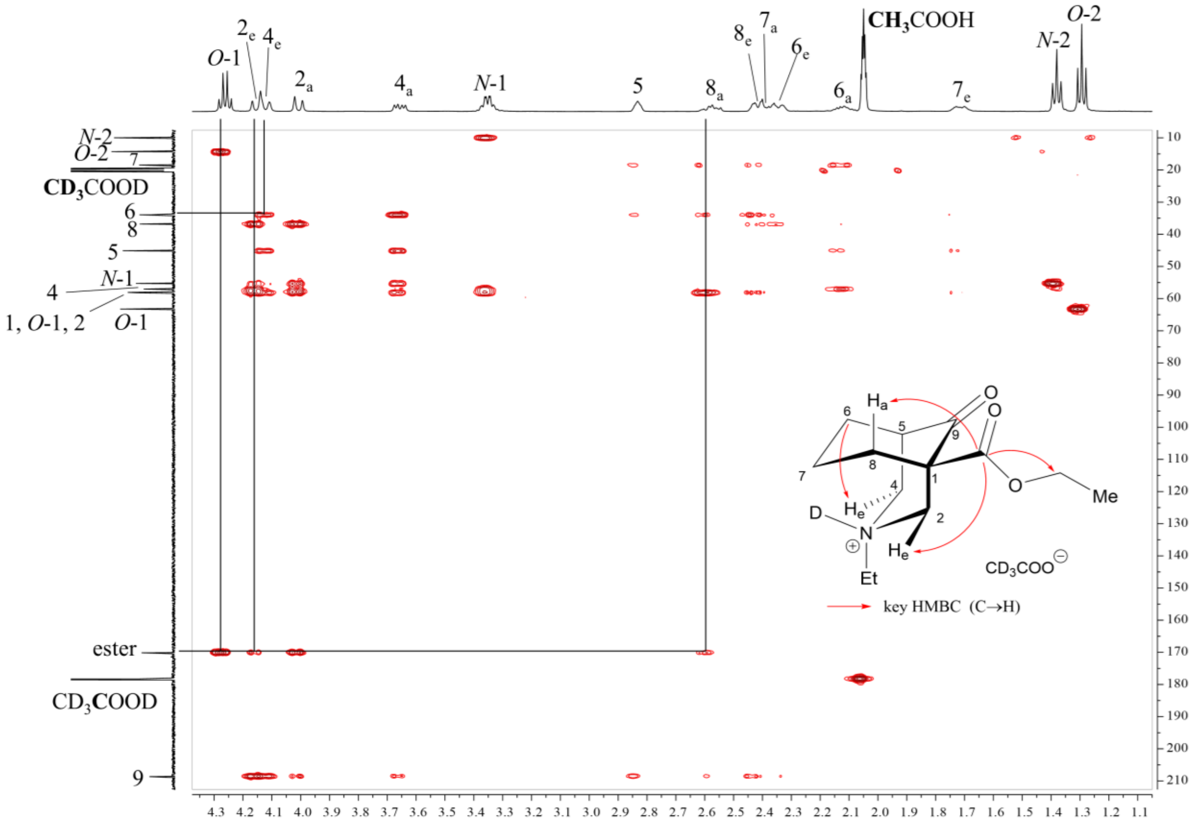

Figure S180. HMBC spectrum of ketone acetate-salt (**31**) in d_4-acetic acid

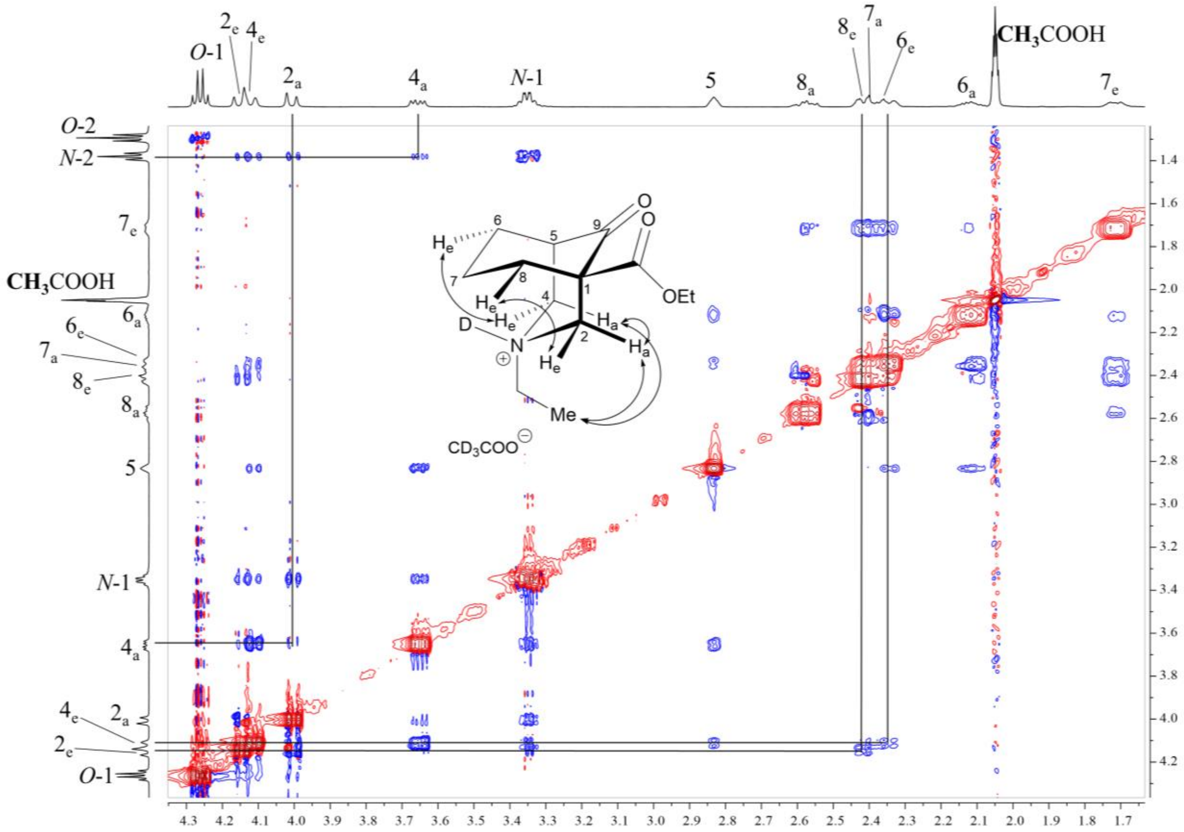

Figure S181. NOESY spectrum of ketone acetate-salt (**31**) in d_4-acetic acid

1-(Ethoxycarbonyl)-3-ethyl-9-oxo-3-azabicyclo[3.3.1]nonan-3-ium chloride (**32**)

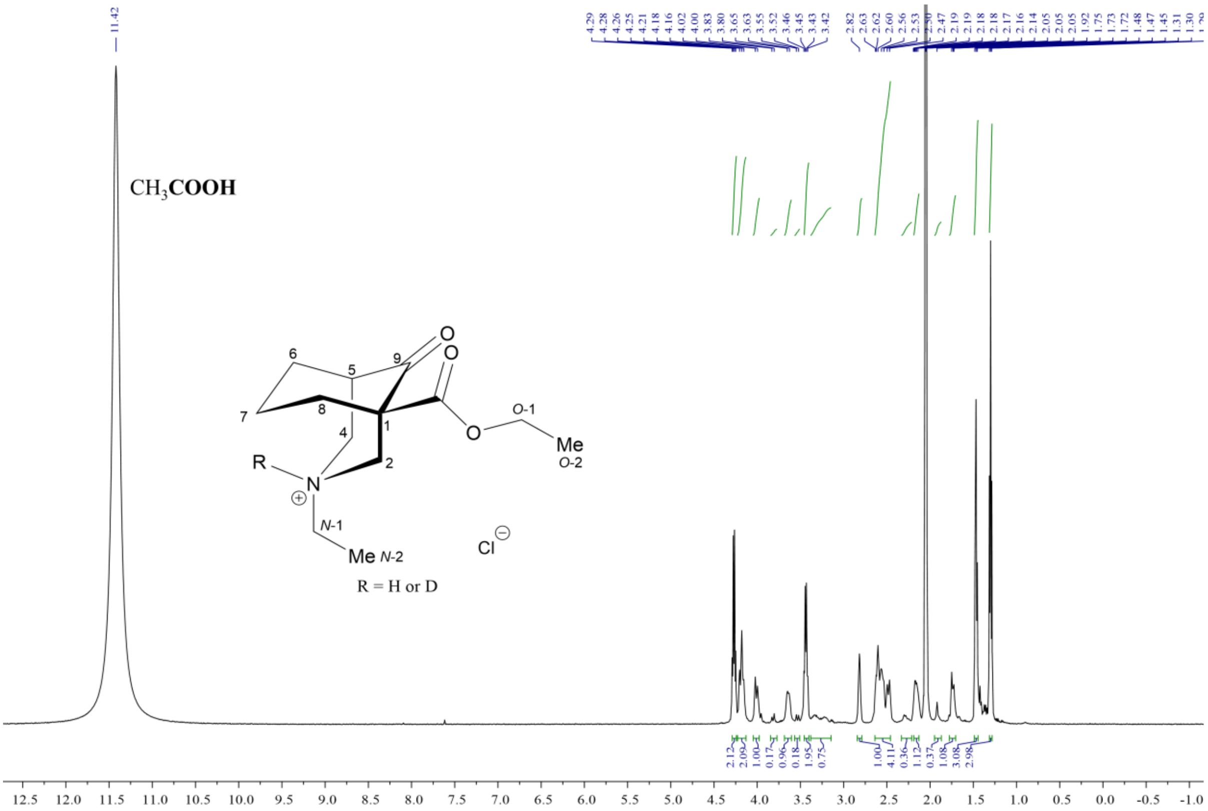

Figure S182. ^{1}H NMR spectrum of ketone chloride-salt (**32**) in d_4-acetic acid

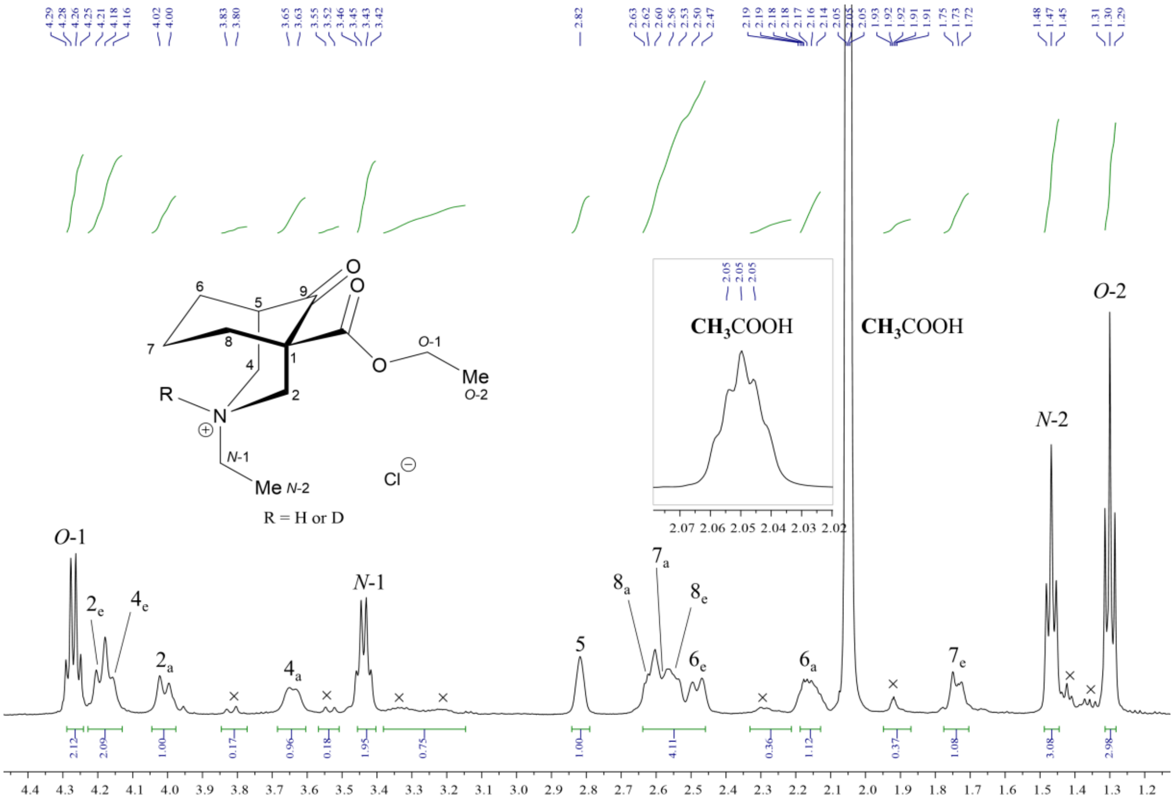

Figure S183. ^{1}H expansion spectrum of ketone chloride-salt (**32**) in d_4-acetic acid with assignments

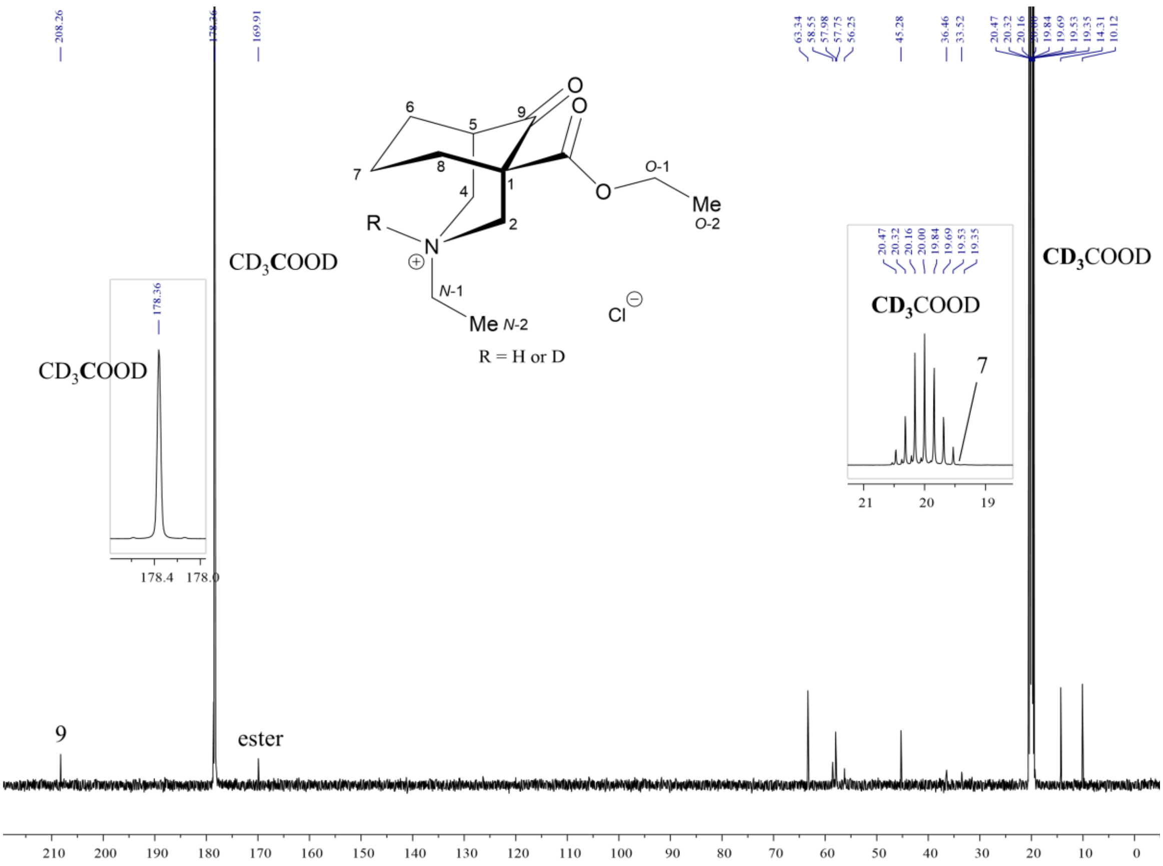

Figure S184. ^{13}C NMR spectrum of ketone chloride-salt (**32**) in d_4-acetic acid

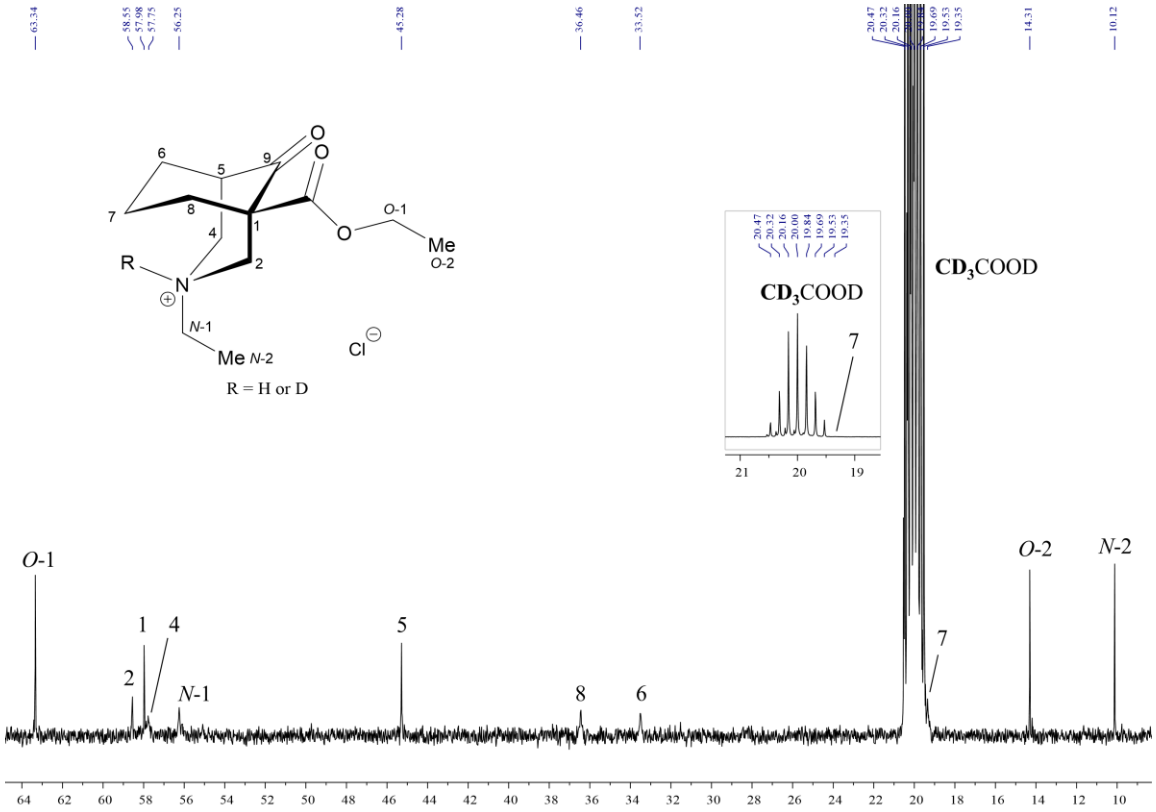

Figure S185. ^{13}C NMR expansion of ketone chloride-salt (**32**) in d_4-acetic acid with assignments

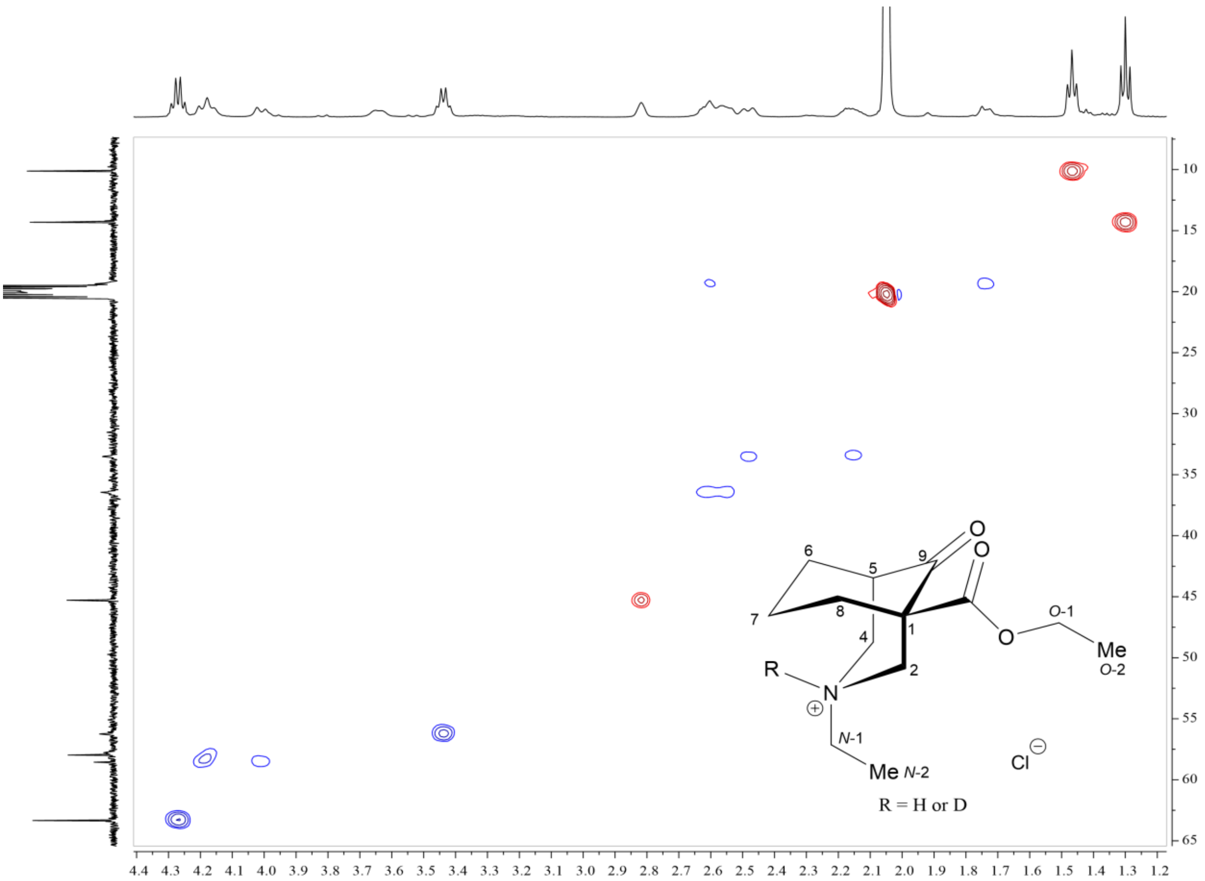

Figure S186. HSQC spectrum of ketone chloride-salt (**32**) in d_4-acetic acid

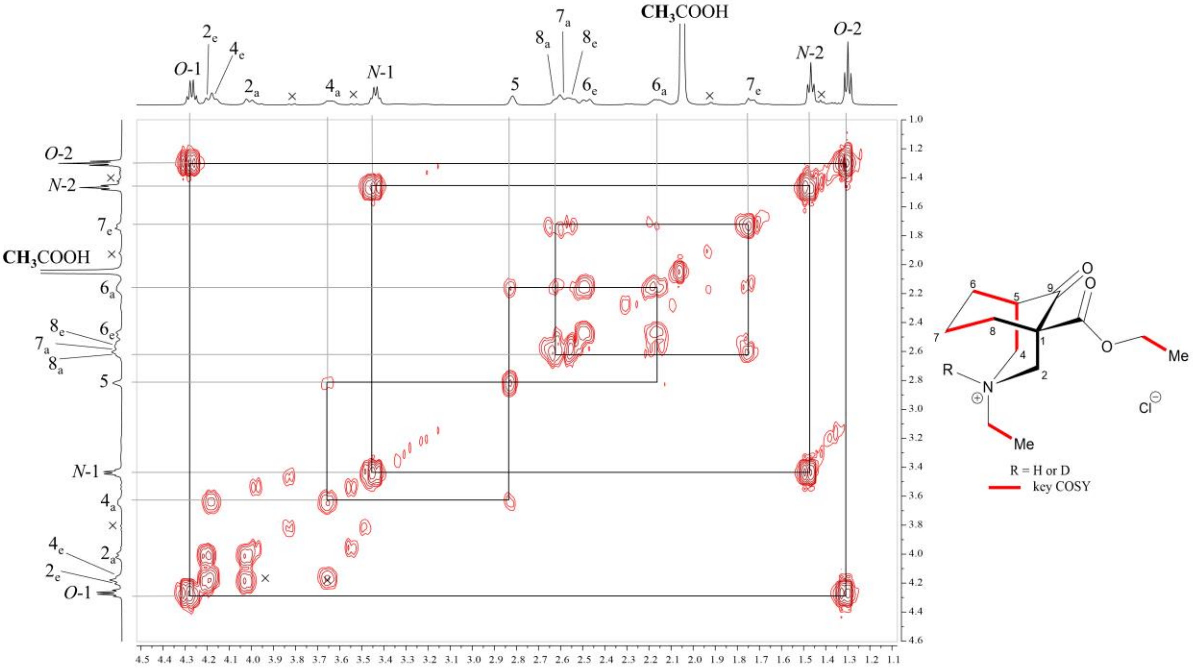

Figure S187. COSY spectrum of ketone chloride-salt (**32**) in d_4-acetic acid

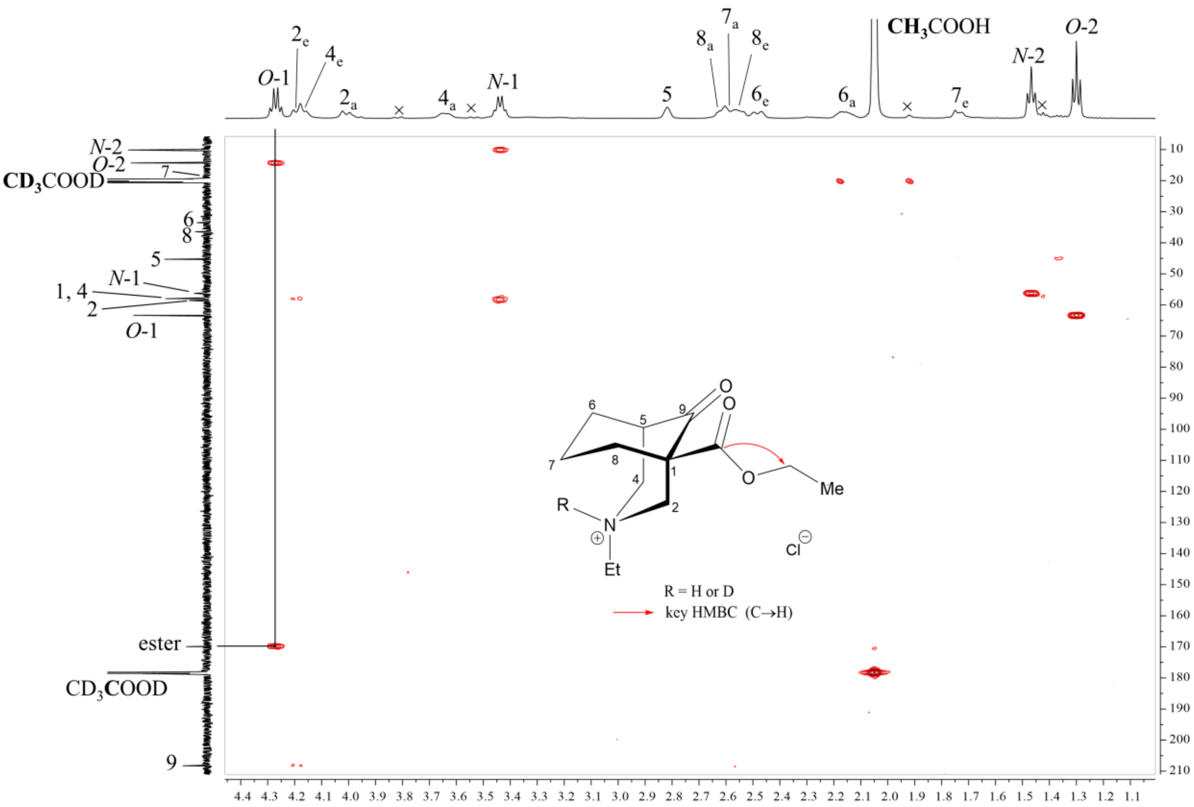

Figure S188. HMBC spectrum of ketone chloride-salt (**32**) in d_4-acetic acid

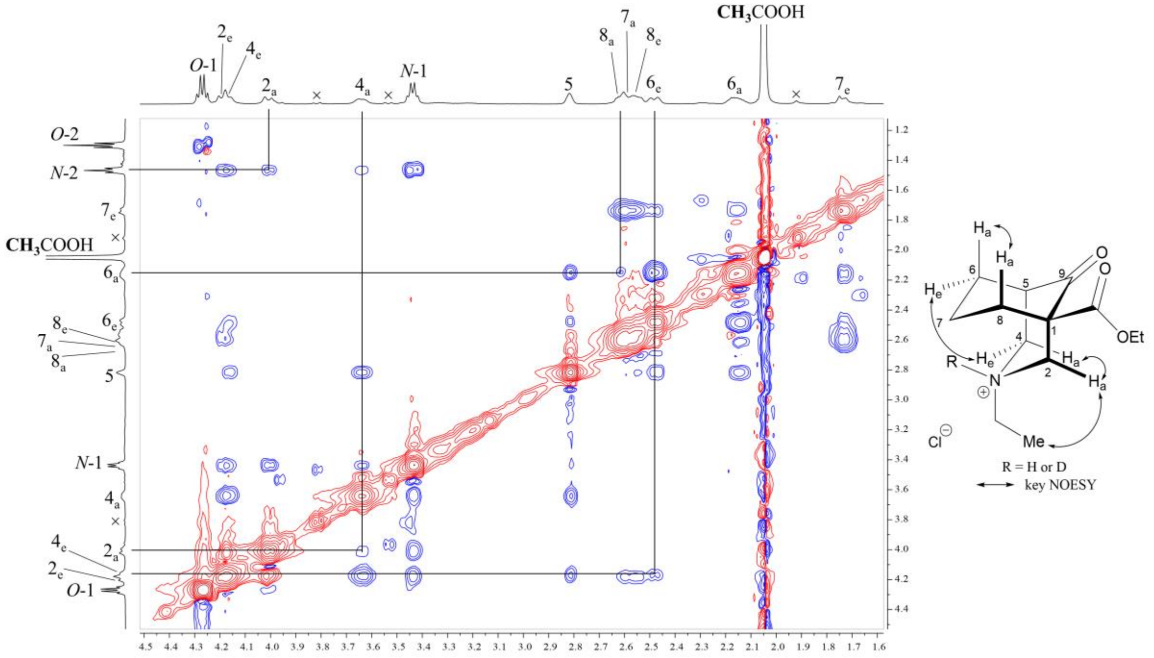

Figure S189. NOESY spectrum of ketone chloride-salt (**32**) in d_4-acetic acid

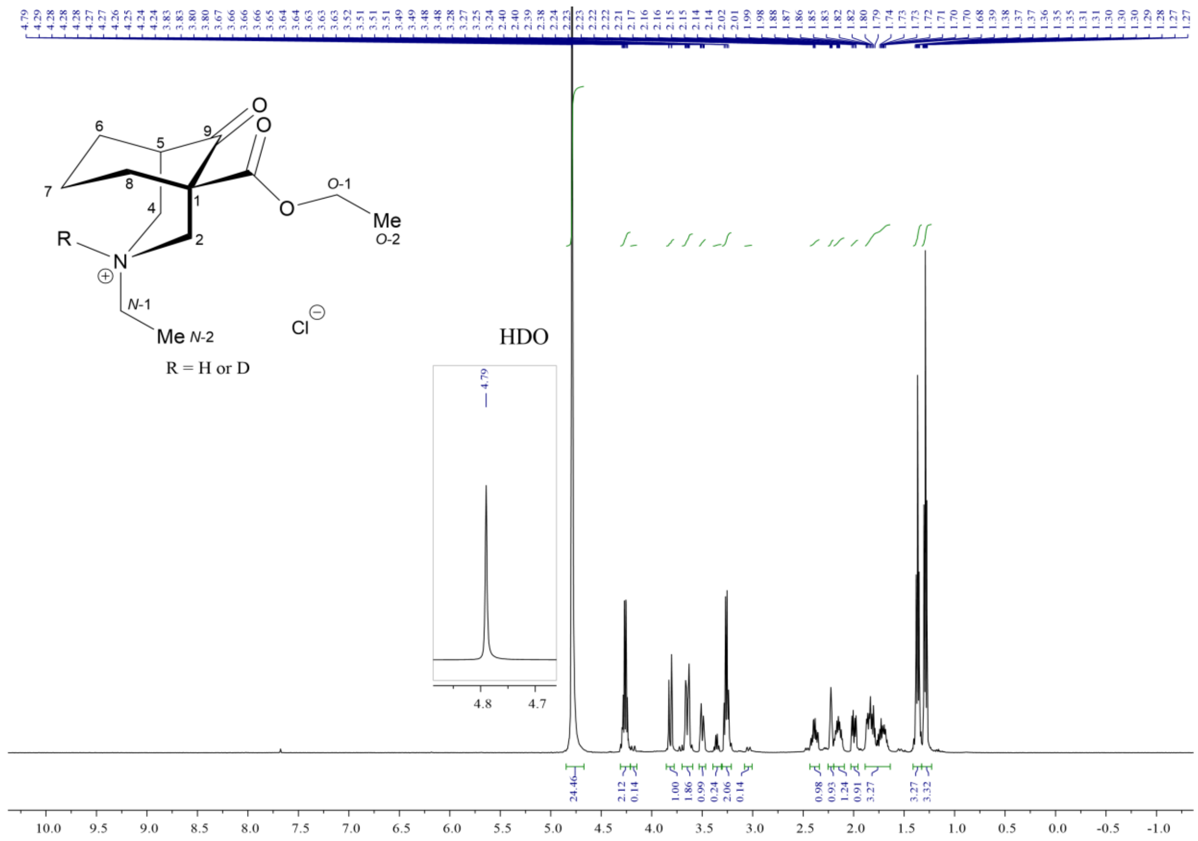

Figure S190. ^{1}H NMR spectrum of ketal chloride-salt (**33**) in D_2O

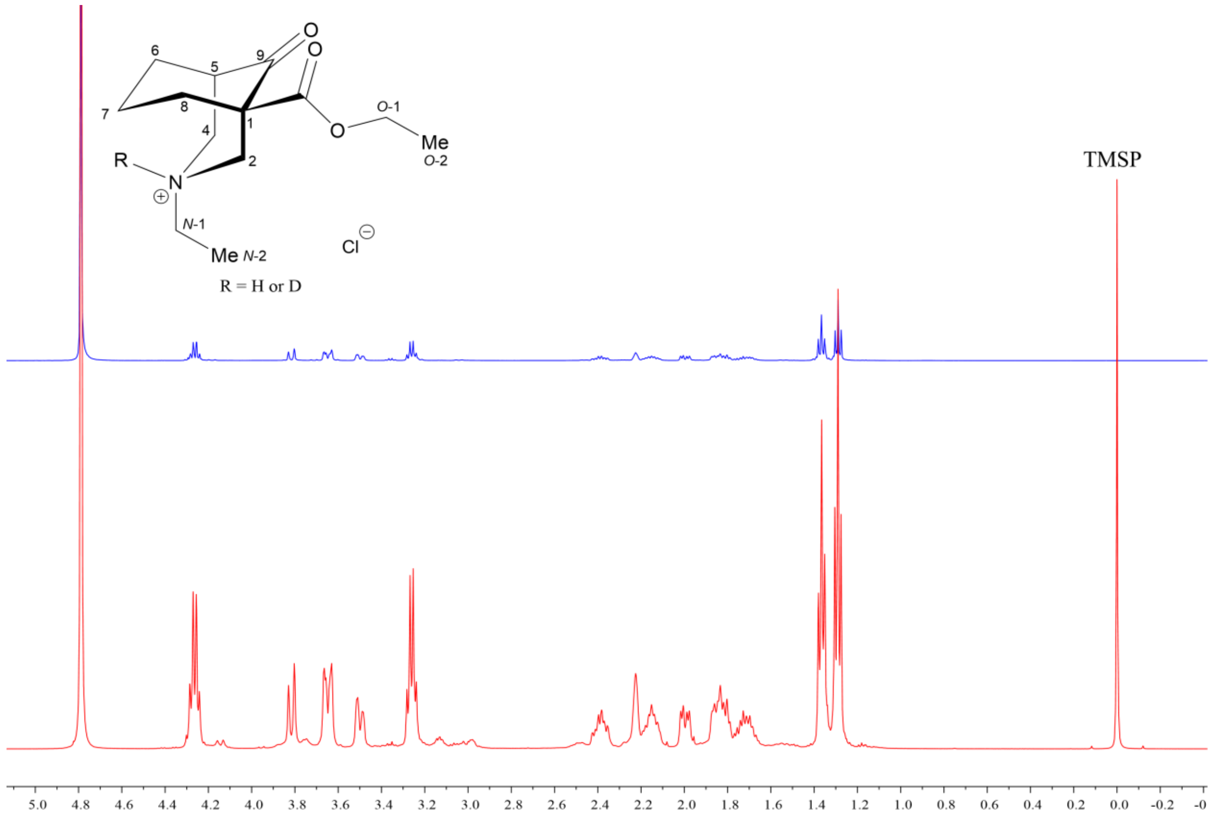

Figure S191. Externally calibrate ^{1}H NMR spectrum of ketal chloride-salt (**33**) in D_2O with TMSP

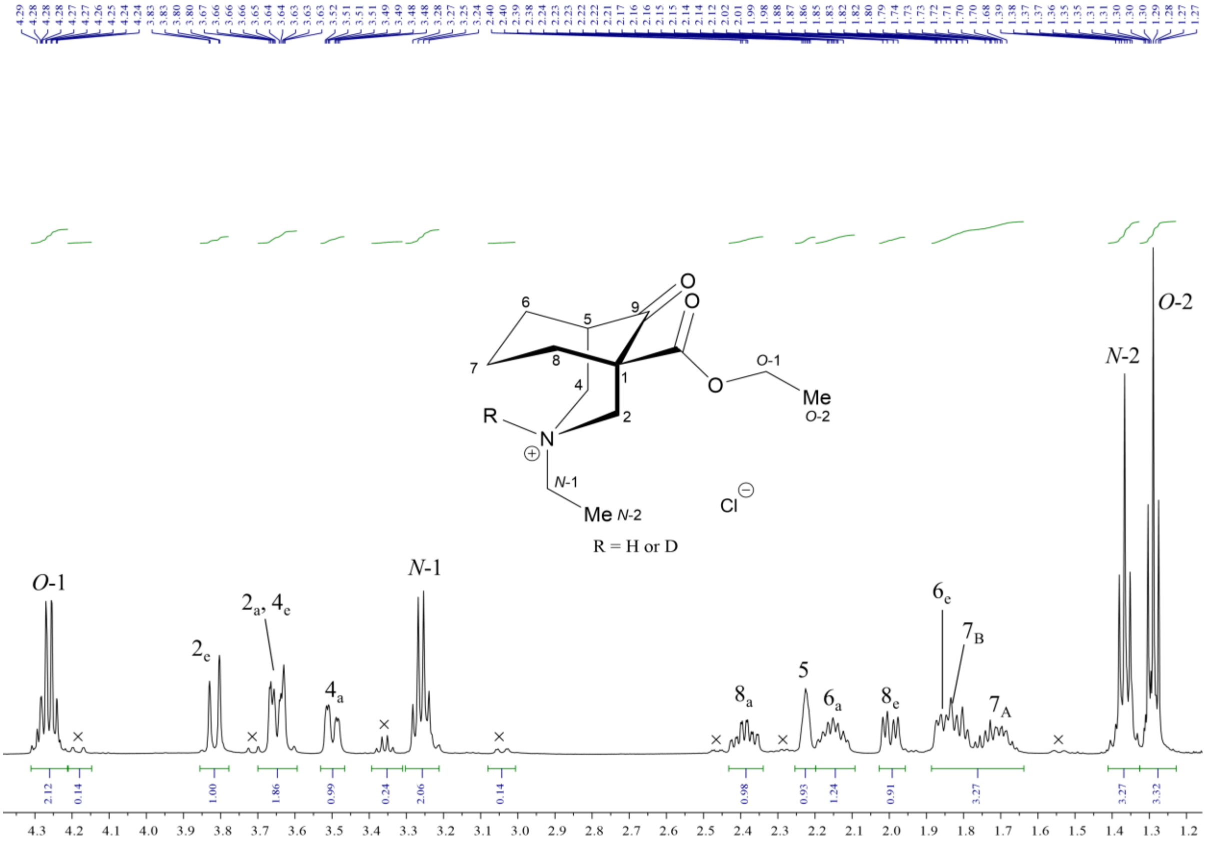

Figure S192. ^{1}H NMR expansion of ketal chloride-salt (**33**) in D_2O with assignments

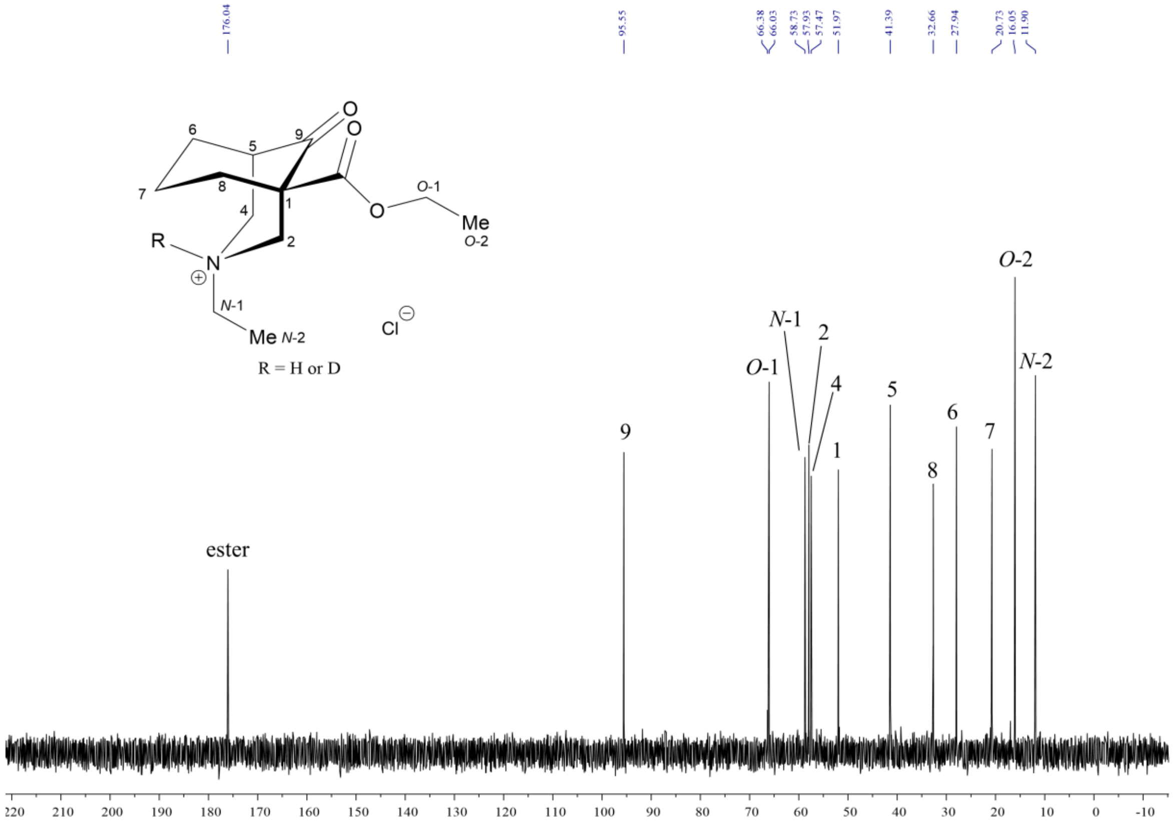

Figure S193. ^{13}C NMR spectrum of ketal chloride-salt (**33**) in D_2O with assignments

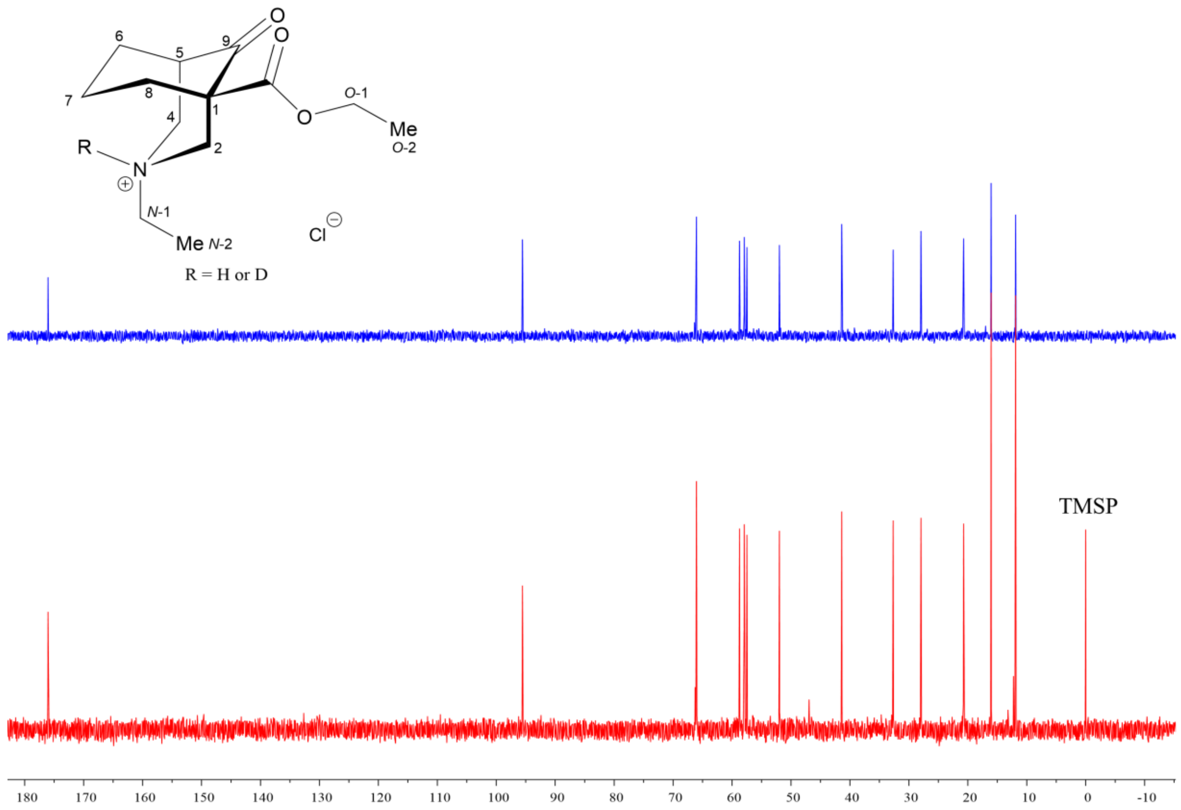

Figure S194. Externally calabrate ^{13}C NMR spectrum of ketal chloride-salt (**33**) in D_2O with TMSP

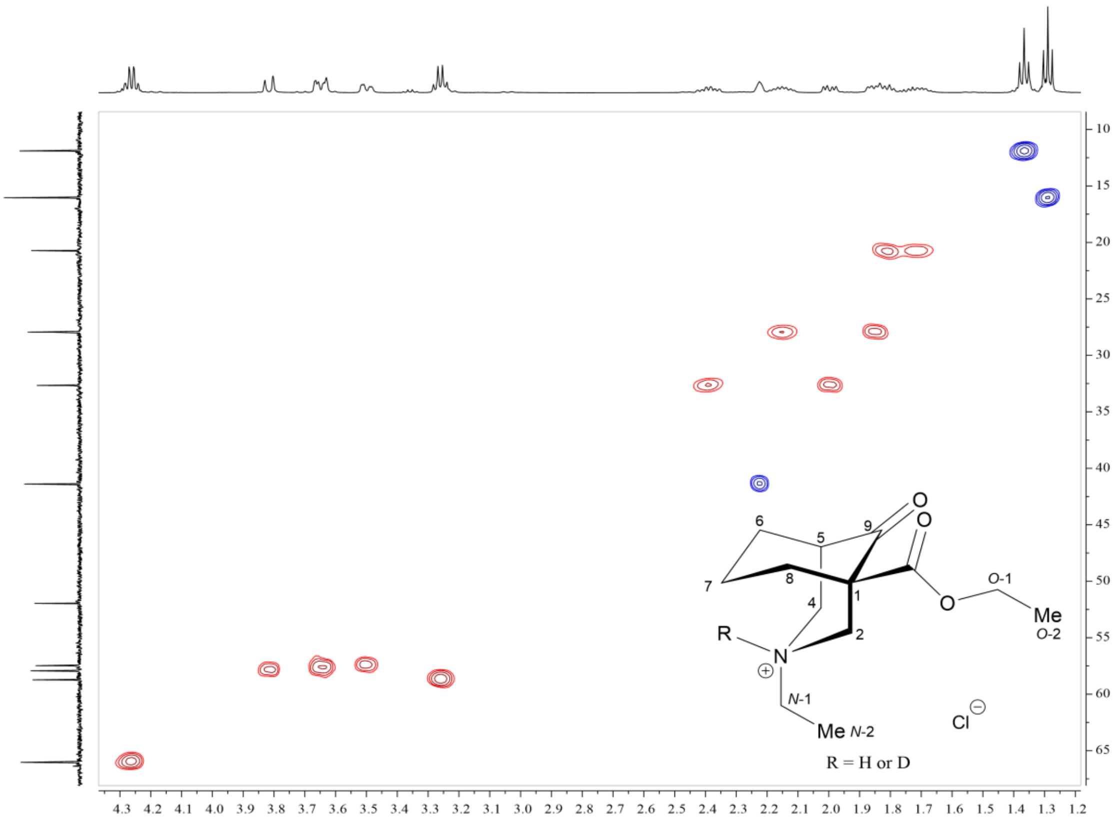

Figure S195. HSQC spectrum of ketal chloride-salt (**33**) in D_2O

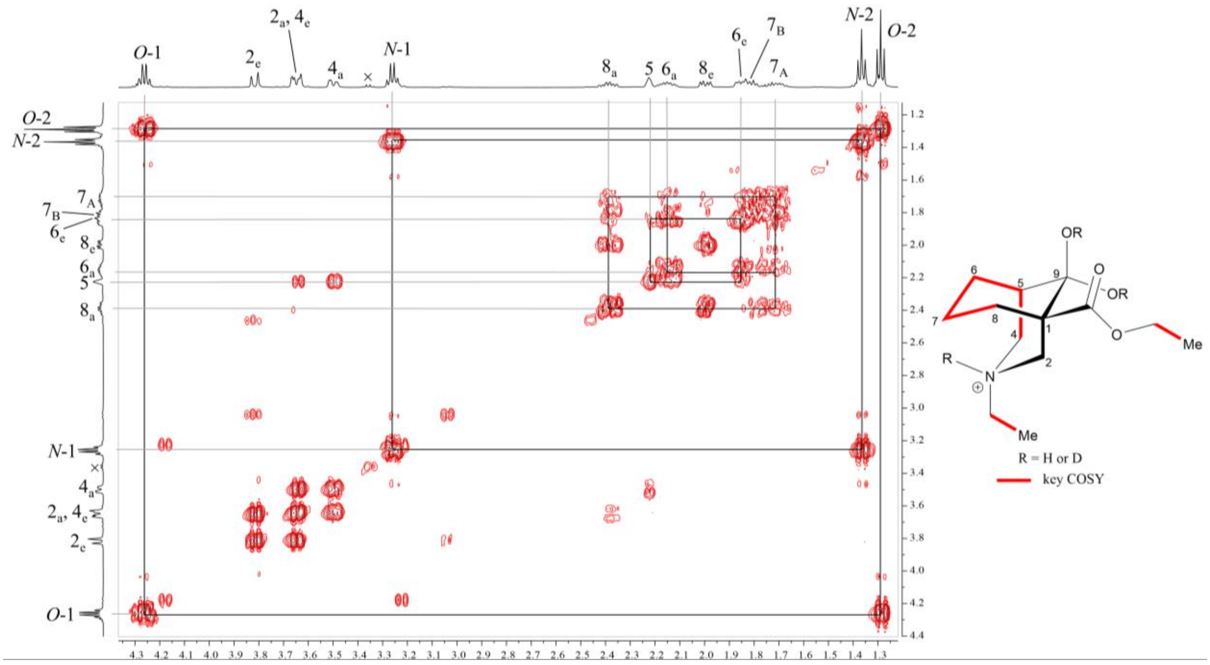

Figure S196. COSY spectrum of ketal chloride-salt (**33**) in D_2O

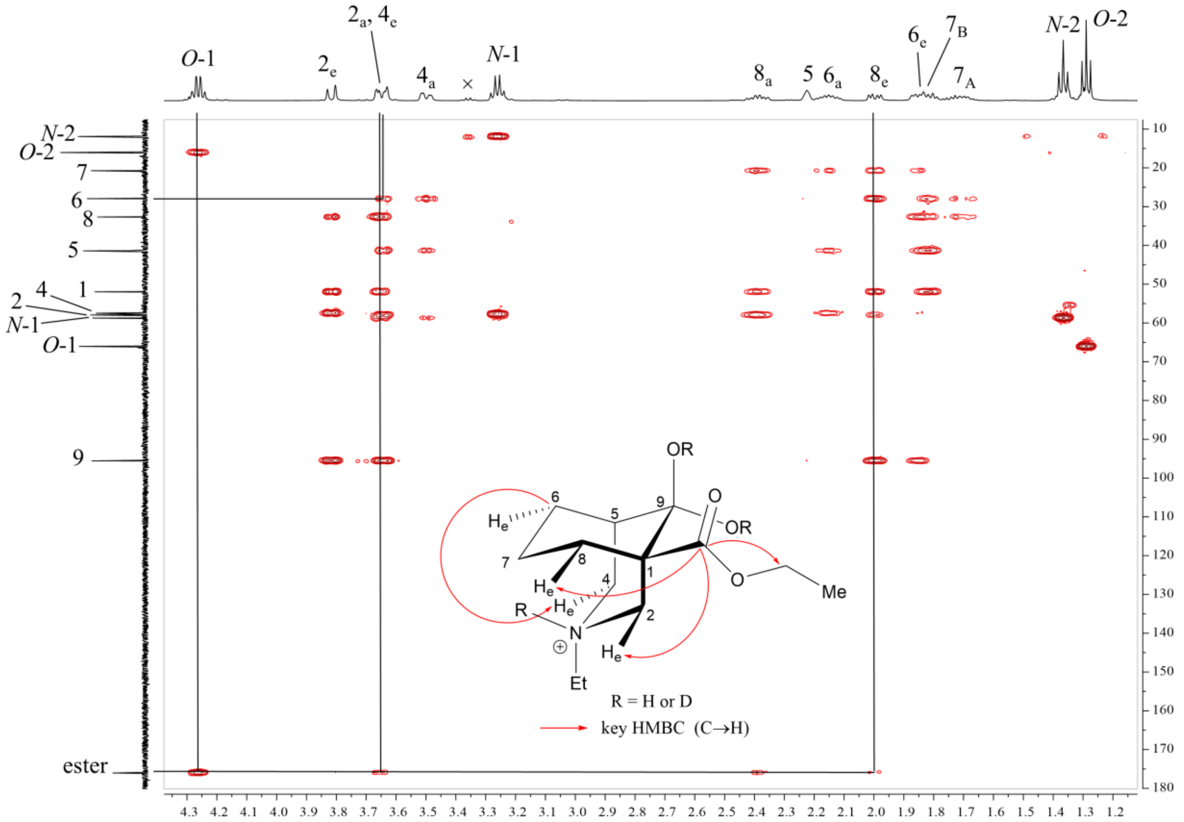

Figure S197. HMBC spectrum of ketal chloride-salt (**33**) in D_2O

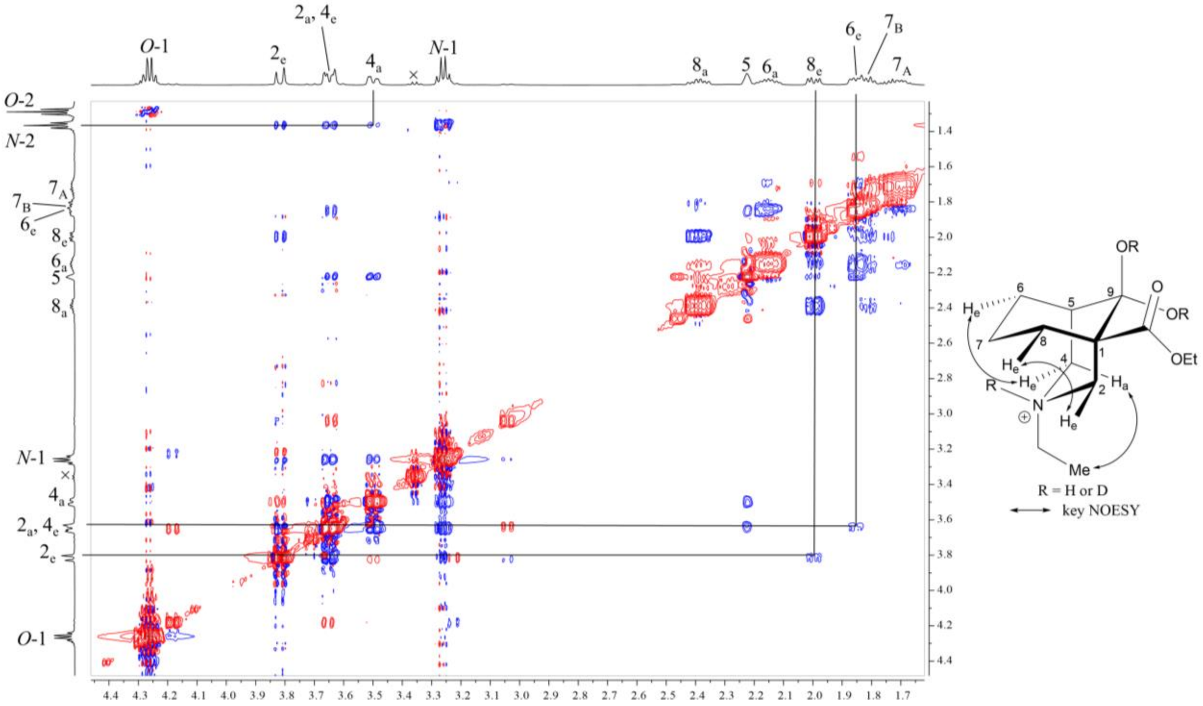

Figure S198. NOESY spectrum of ketal chloride-salt (**33**) in D_2O

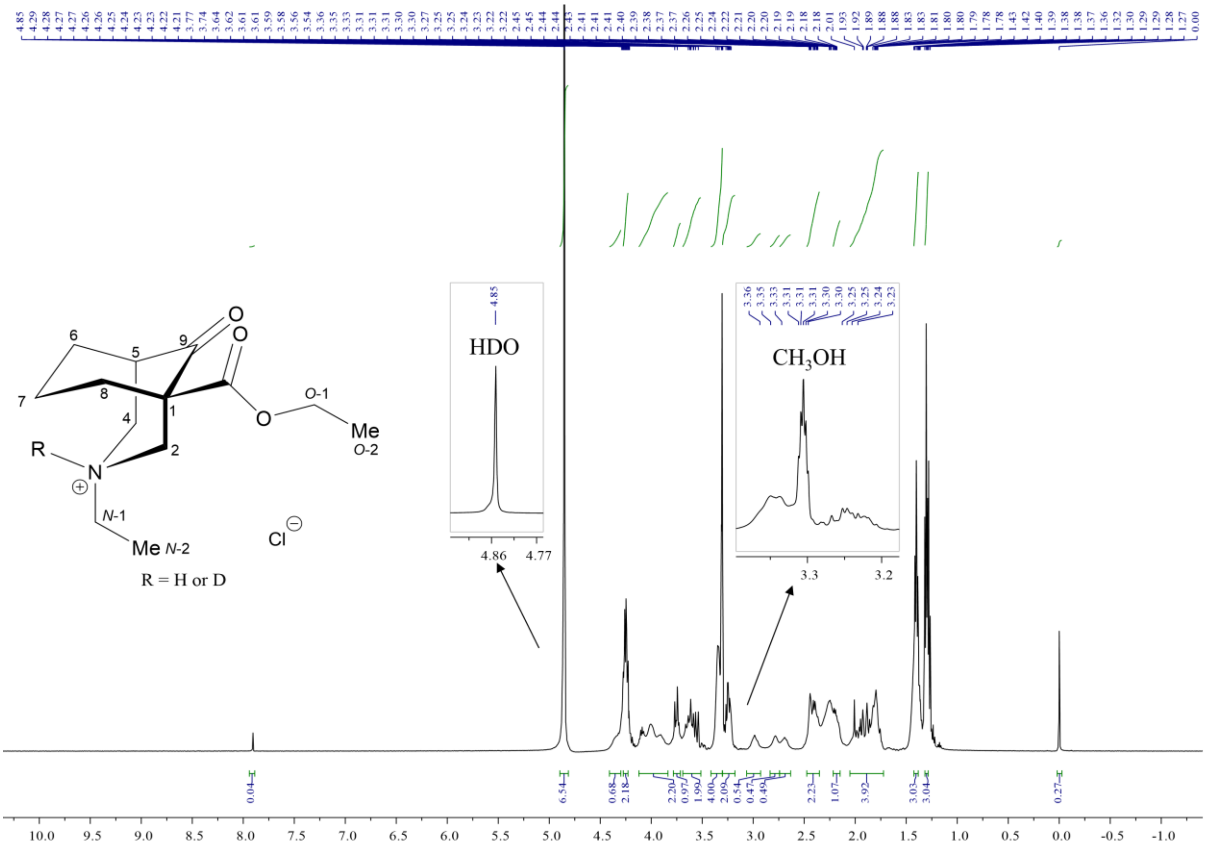

Figure S199. ^{1}H NMR spectrum of ketal chloride-salt (**33**) in CD_3OD

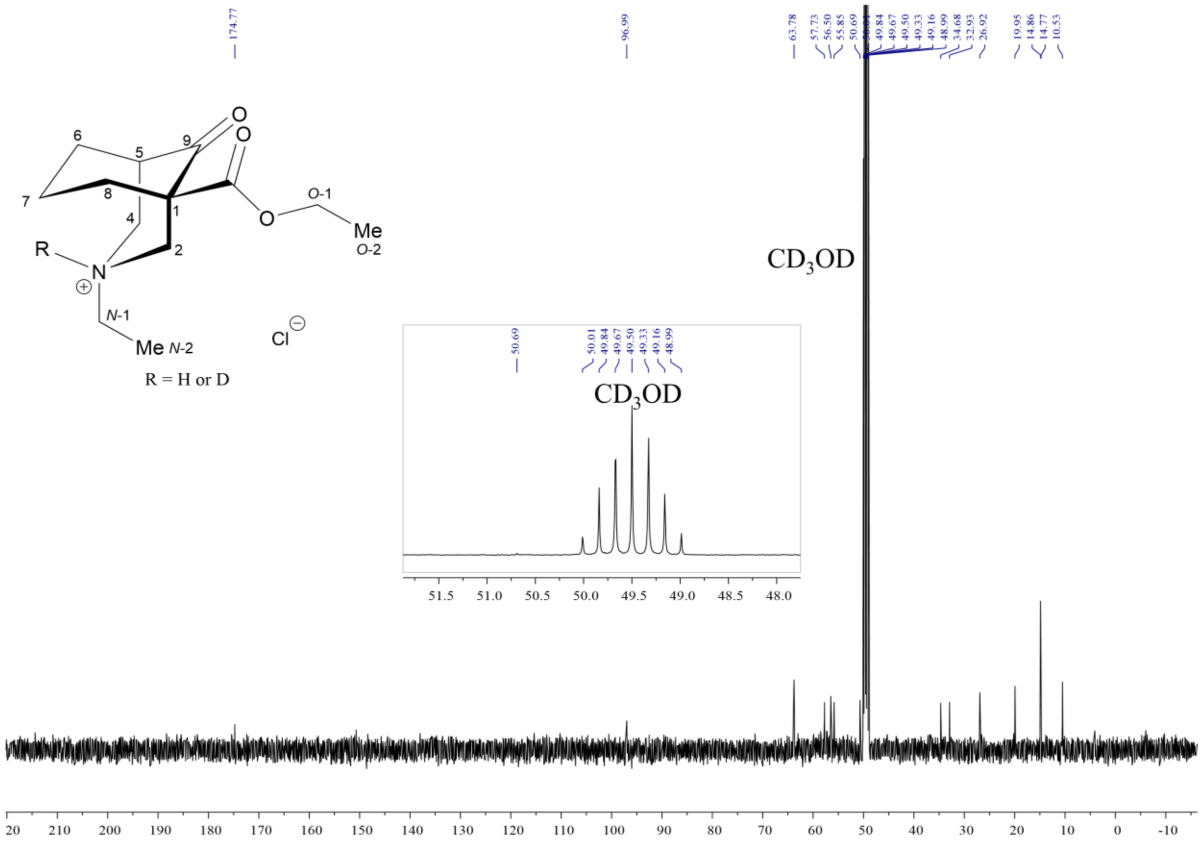

Figure S200. ^{13}C NMR spectrum of ketal chloride-salt (**33**) in CD_3OD

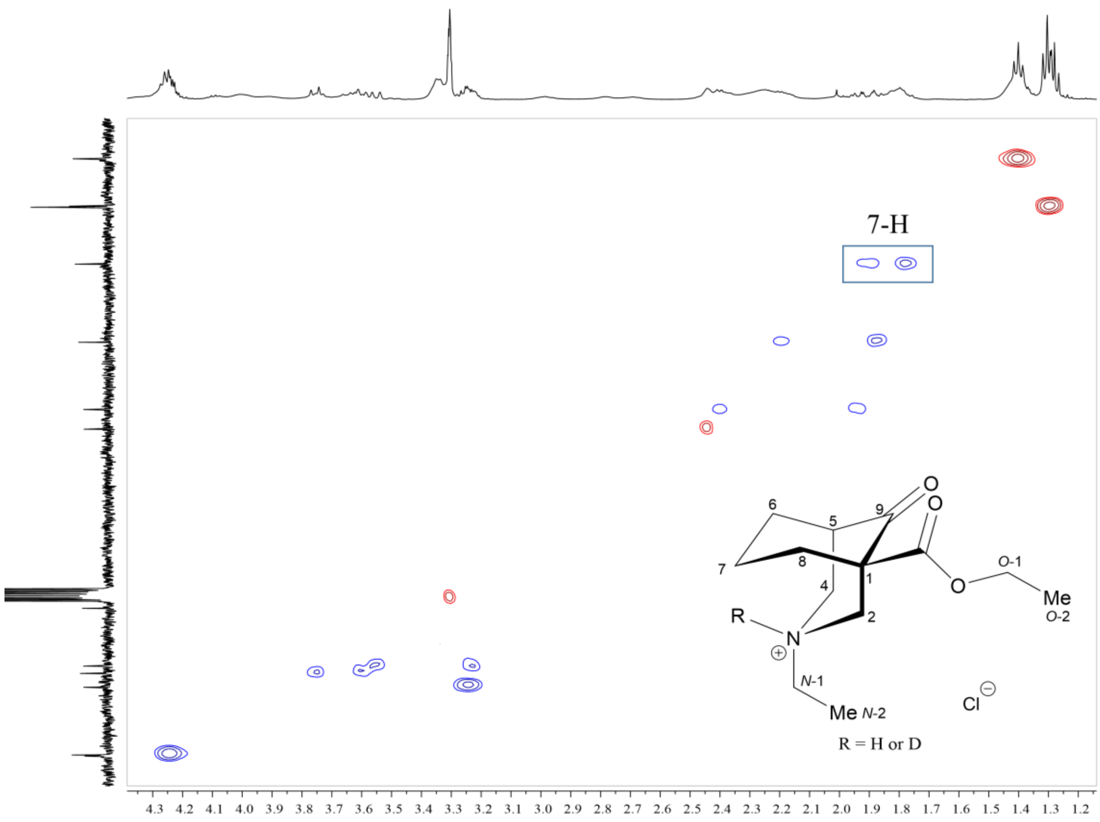

Figure S201. HSQC spectrum of ketal chloride-salt (**33**) in CD_3OD

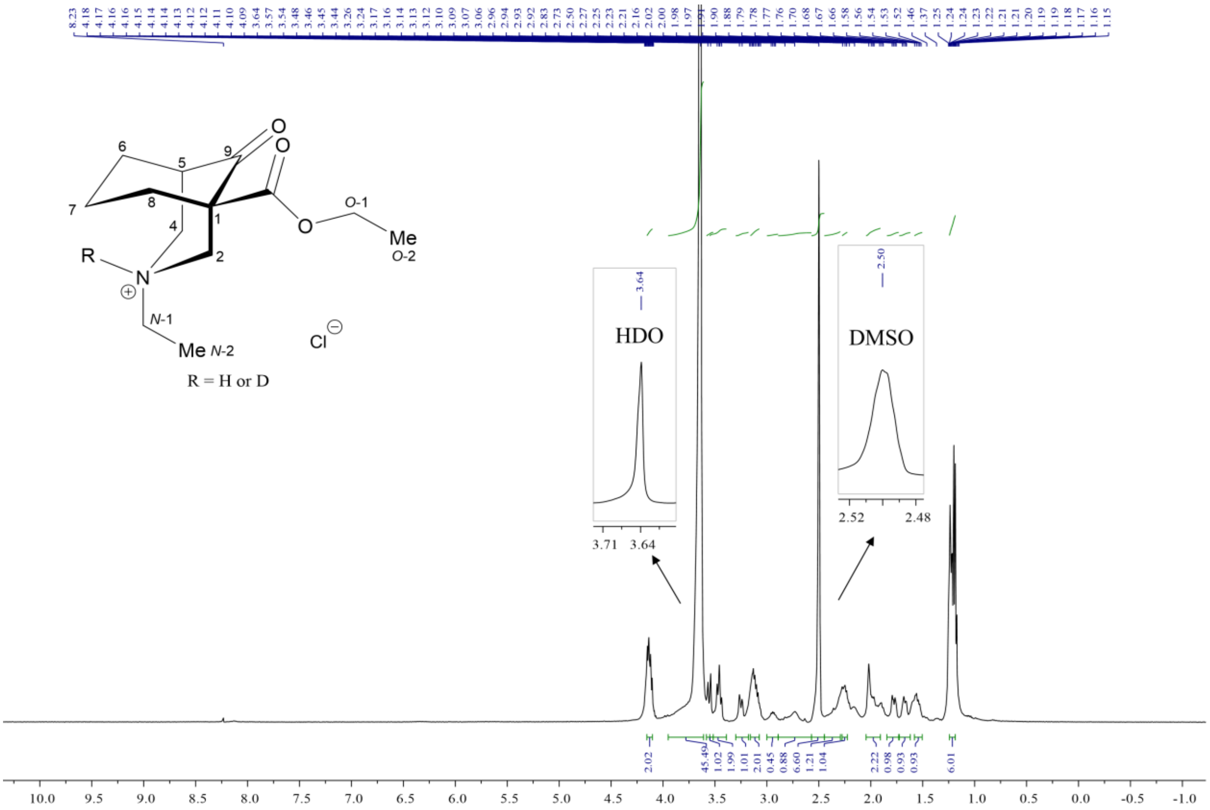

Figure S202. ^{1}H NMR spectrum of ketal chloride-salt (**33**) in d_6-DMSO (with 2 drops of D_2O)

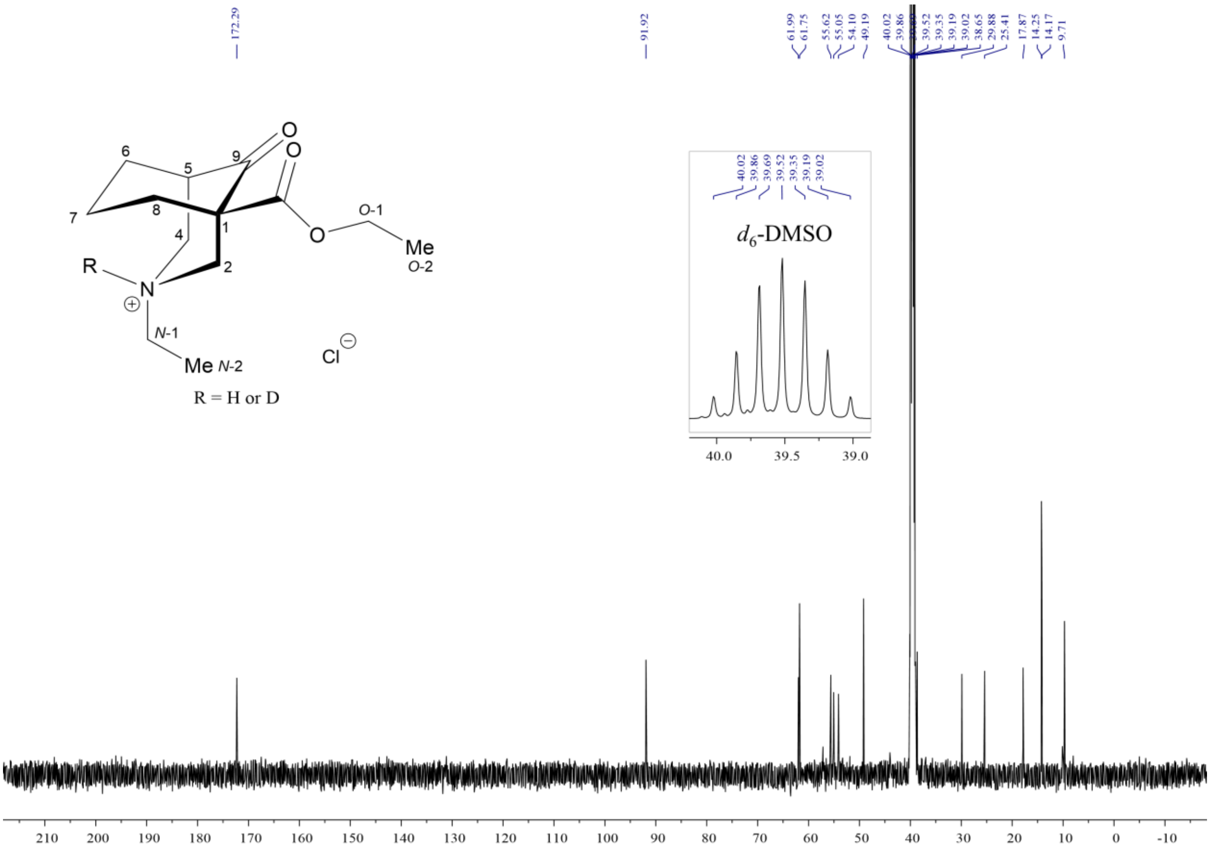

Figure S203. ^{13}C NMR spectrum of ketal chloride-salt (**33**) in d_6-DMSO (with 2 drops of D_2O)

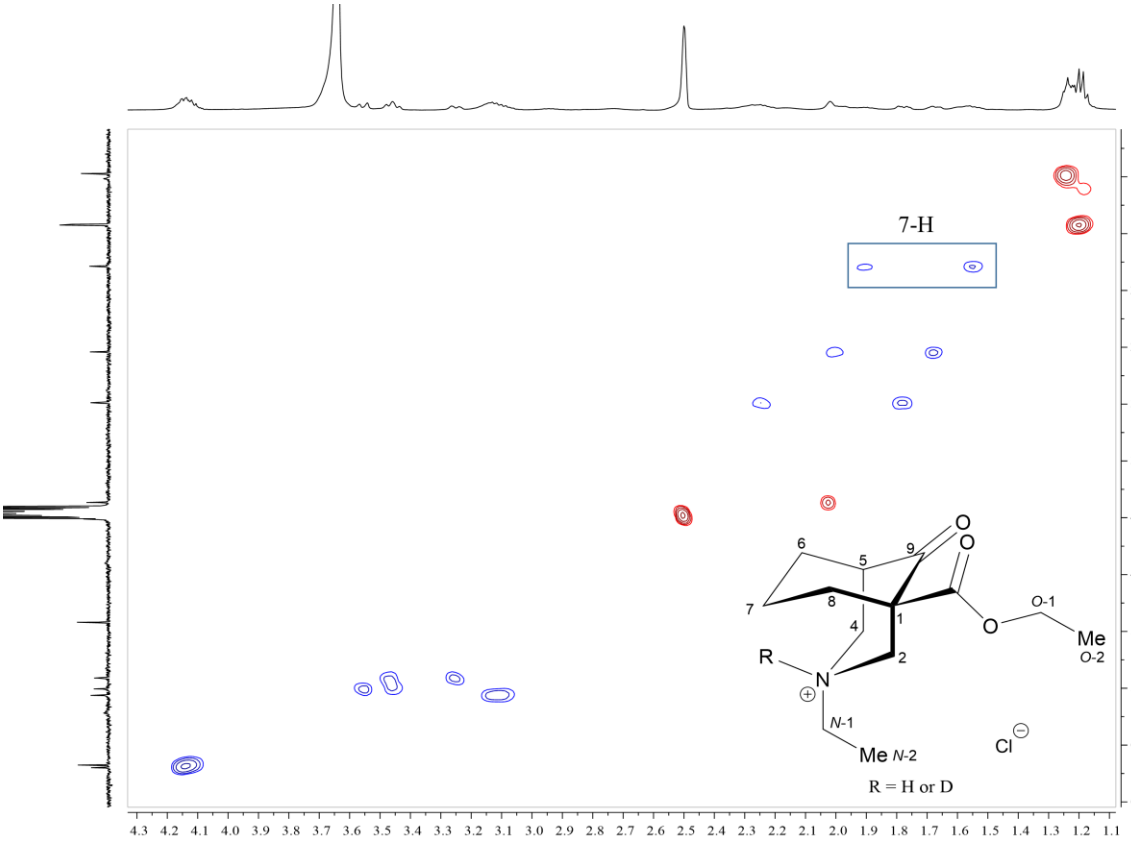

Figure S204. HSQC spectrum of ketal chloride-salt (**33**) in d_6-DMSO (with 2 drops of D_2O)

1-(Ethoxycarbonyl)-3-ethyl-3-methyl-9-oxo-3-azabicyclo[3.3.1]nonan-3-ium iodide (**34**)

Confirmation of Expected Formula

Sample-ID	bl_zz_MeI all	Submitter	zz615 Ziyu Zeng
Analysis Name	bl_zz_MeI all_351122_15_01_56505.d	Supervisor	- Ian Blagbrough
Method used	Confirm Formula Positive 50to500 loop inj.m	Acquisition Date	08/03/2017 19:19:29
Ionisation Mode	positive electrospray (ESI)		

+MS, 1.0-1.3min #(59-76), -Spectral Bkgrnd

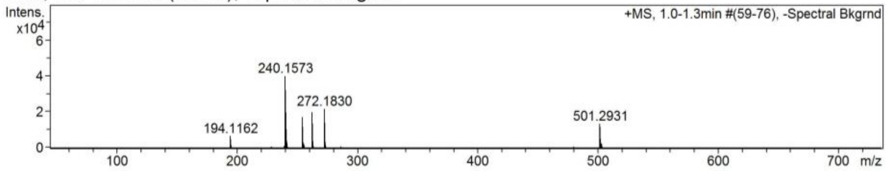

#	m/z	I	I %	Area	S/N
1	194.1162	6522	16.4	245	5630.5
2	240.1573	39695	100.0	1563	4325.4
3	241.1613	3865	9.7	164	410.8
4	254.1733	17268	43.5	739	1516.3
5	262.1390	19608	49.4	867	2032.0
6	263.1428	2034	5.1	96	215.7
7	272.1830	21401	53.9	1023	2869.5
8	273.1872	2407	6.1	123	332.5
9	501.2931	13300	33.5	1189	2883.2
10	502.2967	2547	6.4	243	556.6

Generate Molecular Formula Parameters

Charge	Tolerance	SearchRadius	H/C Ratio min.	H/C Ratio max.	Electron Conf.	Nitrogen Rule	sigma limit
positive	10 ppm	0.05 m/z	0	3	both	true	0.05

Expected Formula C14H24NO3 **Adduct(s):** H, Na

#	meas. m/z	theo. m/z	Err[ppm]	Sigma	Formula

Note: Sigma fits < 0.05 indicates high probability of correct MF, and mass accuracy of 5ppm or better is generally acceptable for publication

Figure S205. MS data of methylated [3.3.1]azabicycle (**34**)

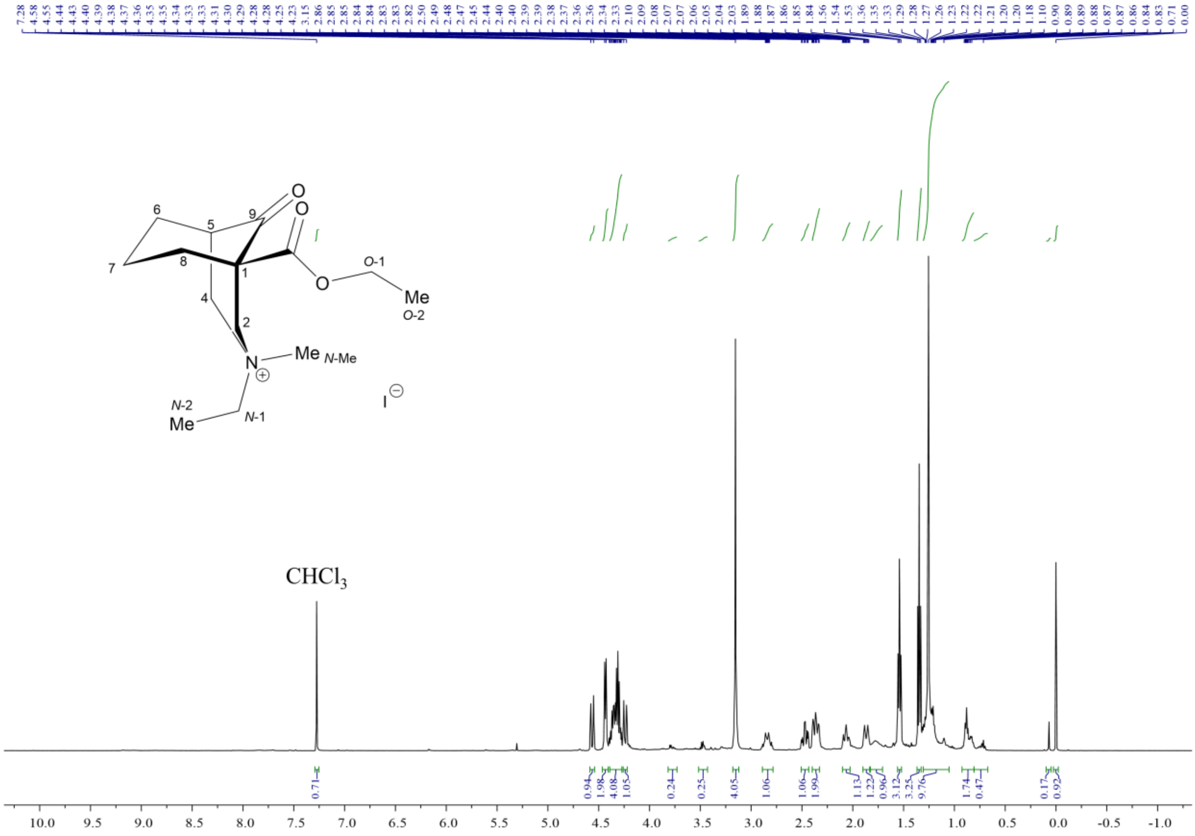

Figure S206. ^{1}H NMR spectrum of methylated [3.3.1]azabicycle (**34**) in $CDCl_3$

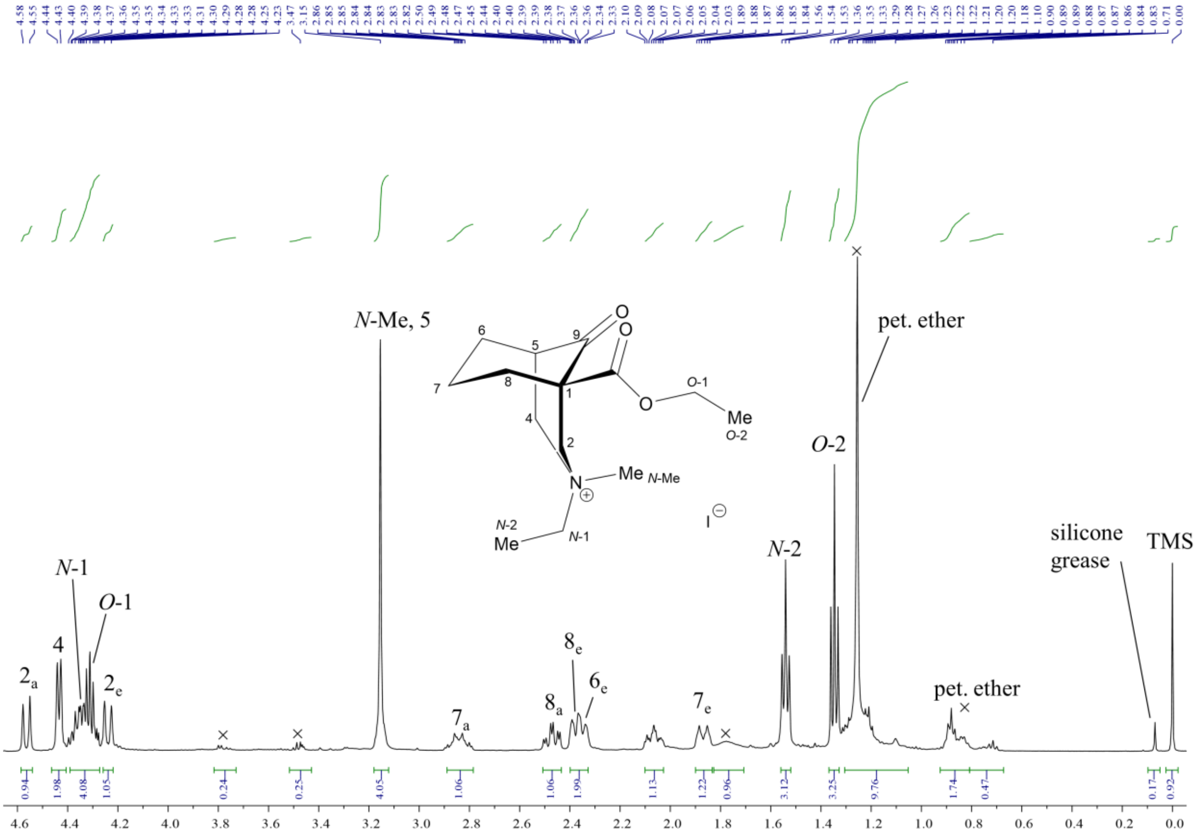

Figure S207. ^{1}H NMR expansion of methylated [3.3.1]azabicycle (**34**) in $CDCl_3$ with assignments

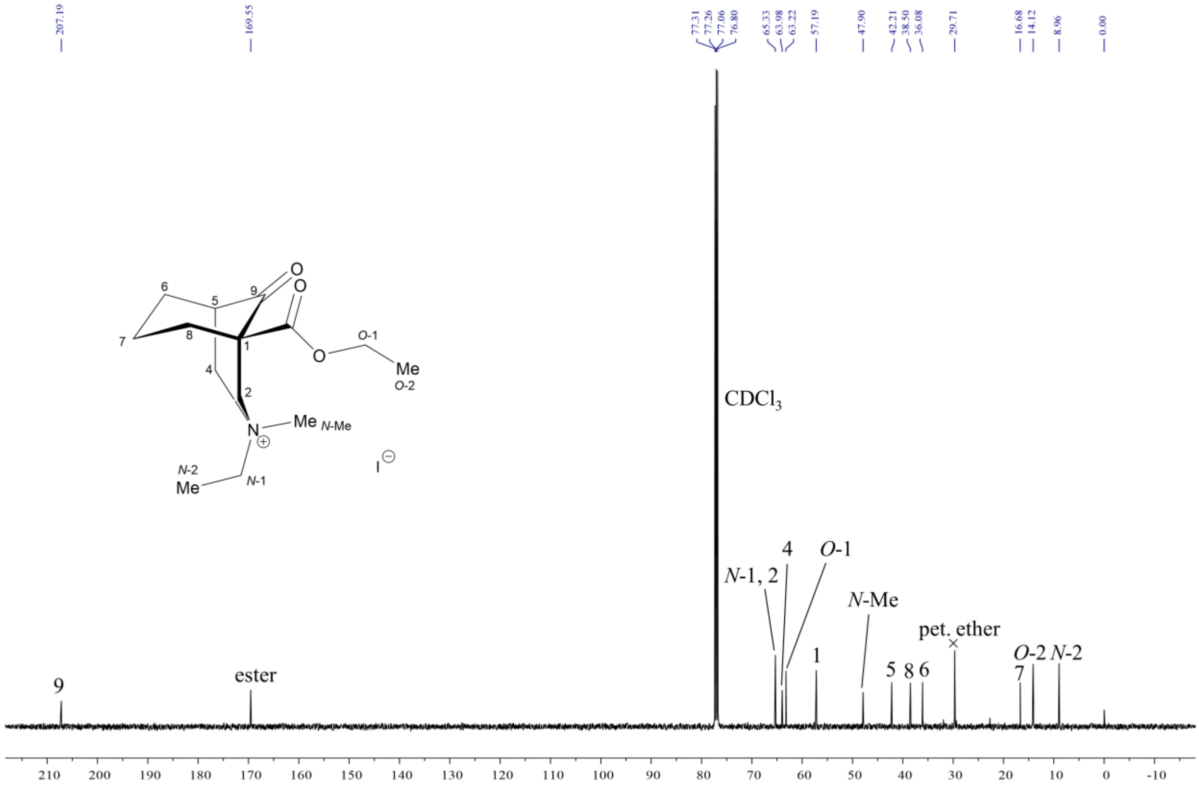

Figure S208. ^{13}C NMR spectrum of methylated [3.3.1]azabicycle (**34**) in $CDCl_3$ with assignments

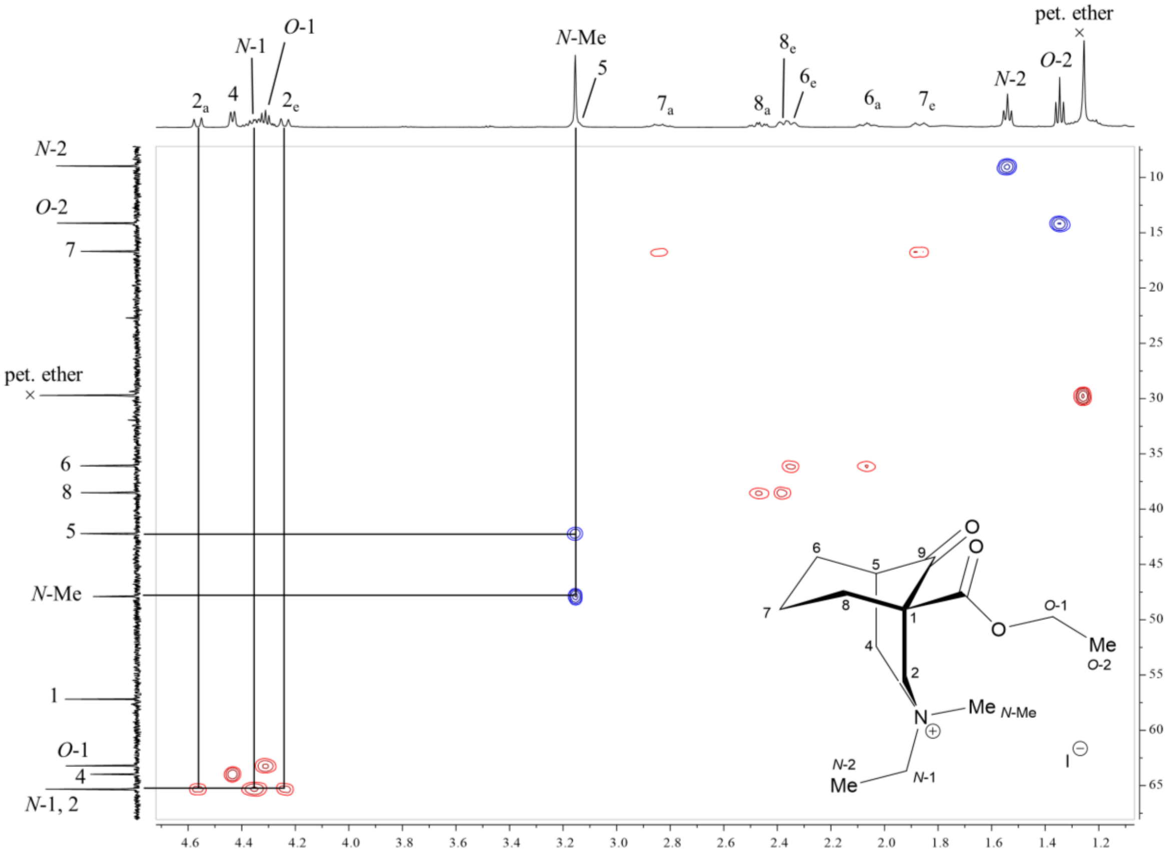

Figure S209. HSQC spectrum of methylated [3.3.1]azabicycle (**34**) in $CDCl_3$

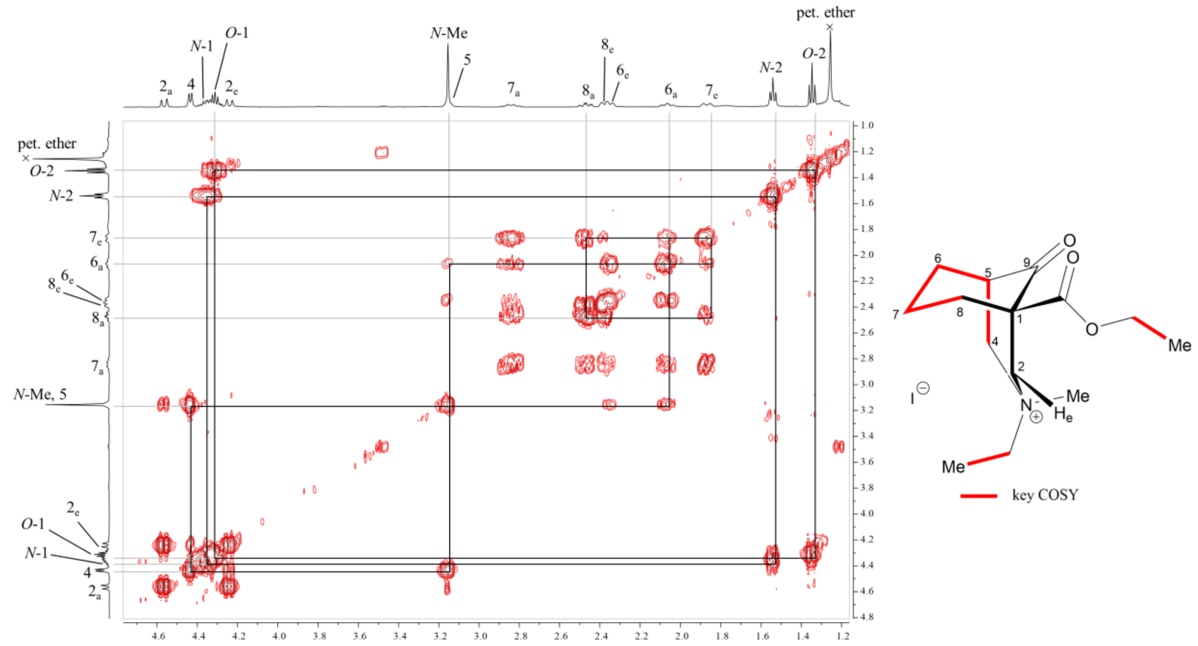

Figure S210. COSY spectrum of methylated [3.3.1]azabicycle (**34**) in $CDCl_3$

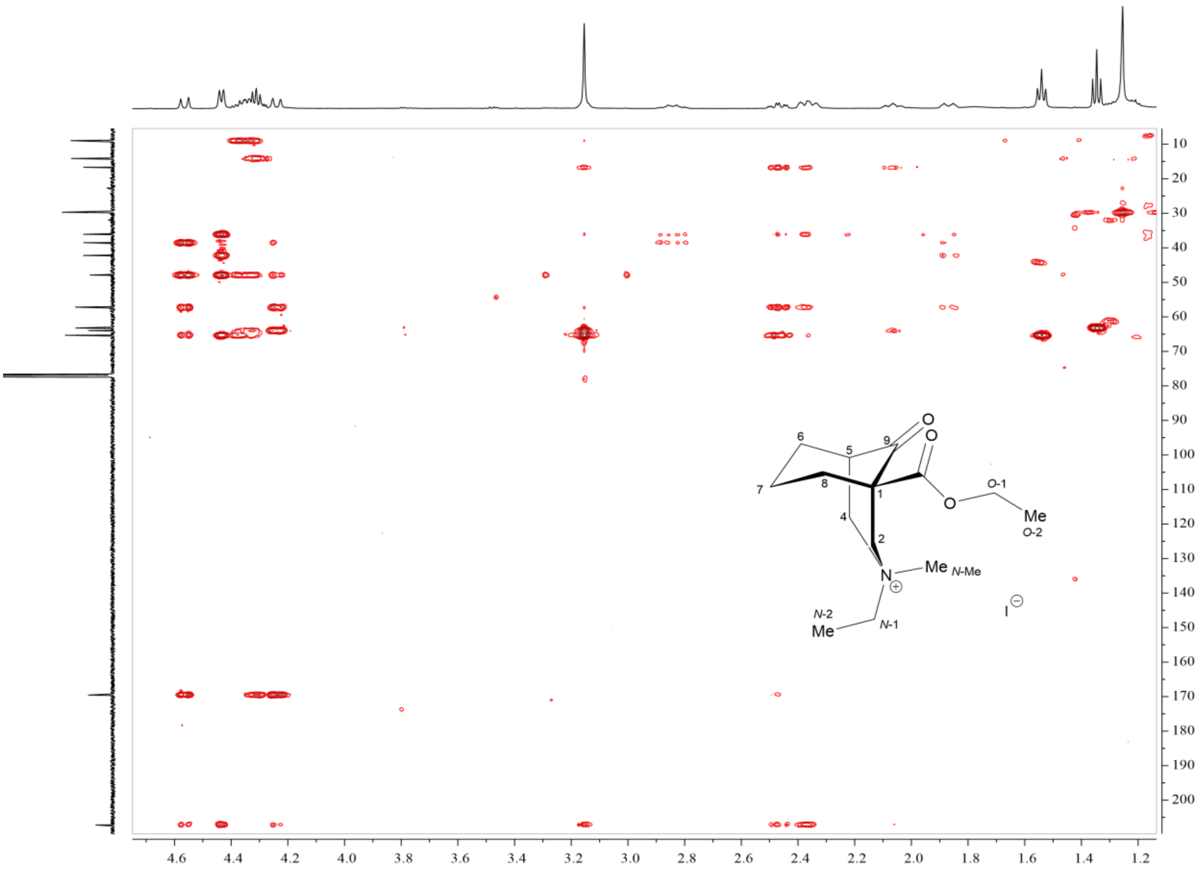

Figure S211. HMBC spectrum of methylated [3.3.1]azabicycle (**34**) in $CDCl_3$

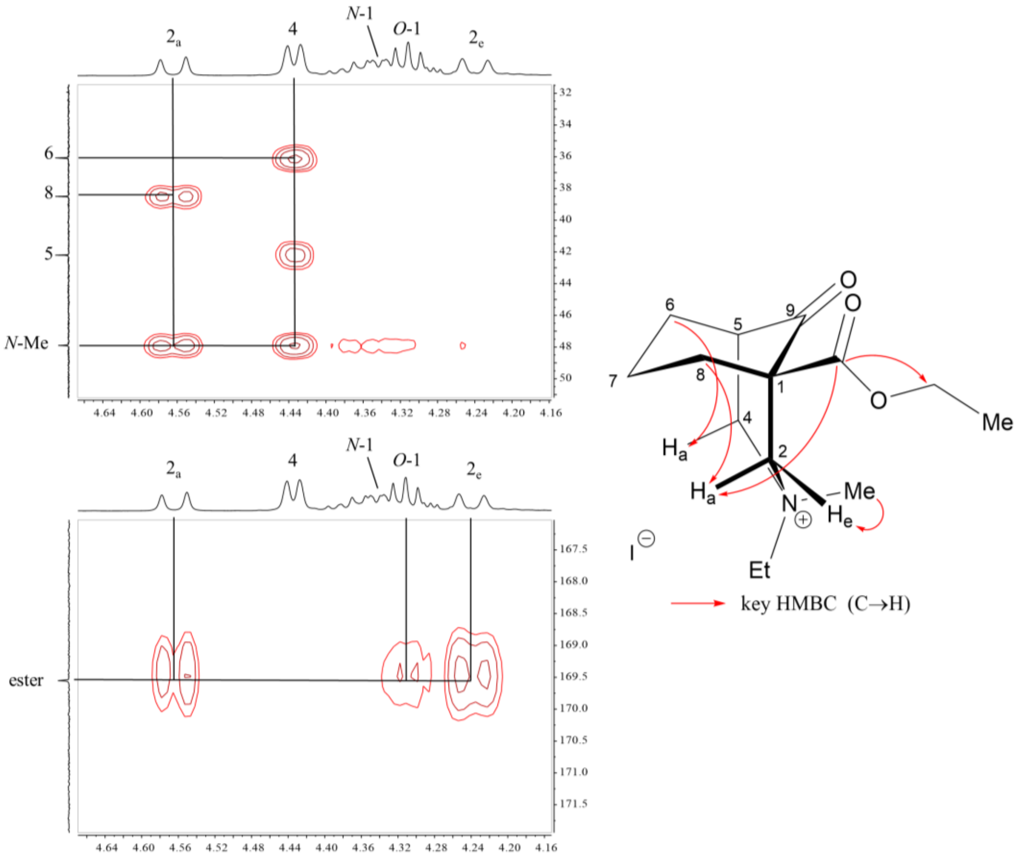

Figure S212. HMBC expansions of methylated [3.3.1]azabicycle (**34**) in $CDCl_3$

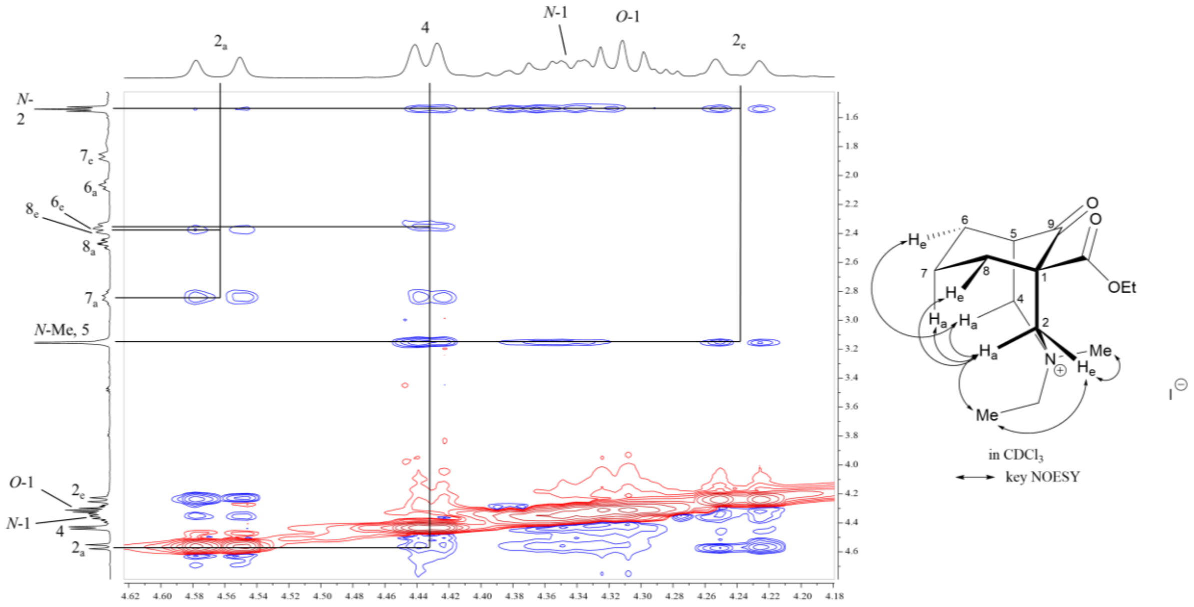

Figure S213. NOESY spectrum of methylated [3.3.1]azabicycle (**34**) in $CDCl_3$

((1*R*,5*S*,9*s*))-9-Hydroxy-3-oxabicyclo[3.3.1]nonane-1,5-diyl) dimethanol (**36**)

Walkup MS Report

**Data File**	oxa_Pos_LoopInjection_MS_04091.d	**Sample Name**	oxa
**Sample Type**	Sample	**Position**	P1-B1
**Instrument Name**	6545 QTof	**User Name**	Ziyu Zeng
**Acq Method**	Pos_LoopInjection_MS.m	**Acquired Time**	5/7/2019 6:49:58 PM
**IRM Calibration Status**	Success	**DA Method**	Pos_LoopInjection_MS.m
**Comment**			

**Sample Group**		**Info.**	
**Walkup Sample Description**		**Walkup Method**	Pos_LoopInjection_MS
**Formula**	C10H18O4	**Walkup Method Description**	Positive mode ionization using loop injection
**Stream Name**	LC 1	**Acquisition SW Version**	6200 series TOF/6500 series Q-TOF B.09.00 (B9044.0)

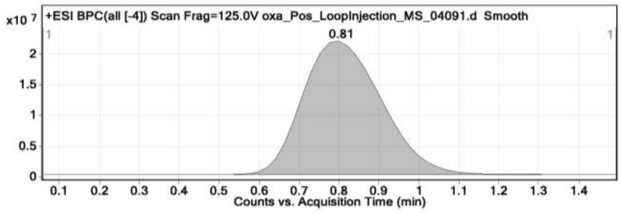

Figure 1: Base peak chromatogram

User Chromatogram Peak List

RT (min)	Area	Area %	Area Sum (%)	Base Peak (m/z)	Width (min)
0.81	298373756	100.00	100.00	425.2855	0.240

Compound Table

Compound Label	RT (min)	Observed mass (m/z)	Neutral observed mass (Da)	Theoretical mass (Da)	Mass error (ppm)	Isotope match score (%)
Cpd 1: C10 H18 O4	0.81	203.1281	202.1209	202.1205	2.01	99.12

Mass errors of between -5.00 and 5.00 ppm with isotope match scores above 60% are considered confirmation of molecular formulae

Figure S214. MS data of [3.3.1]oxabicycle (**36**)

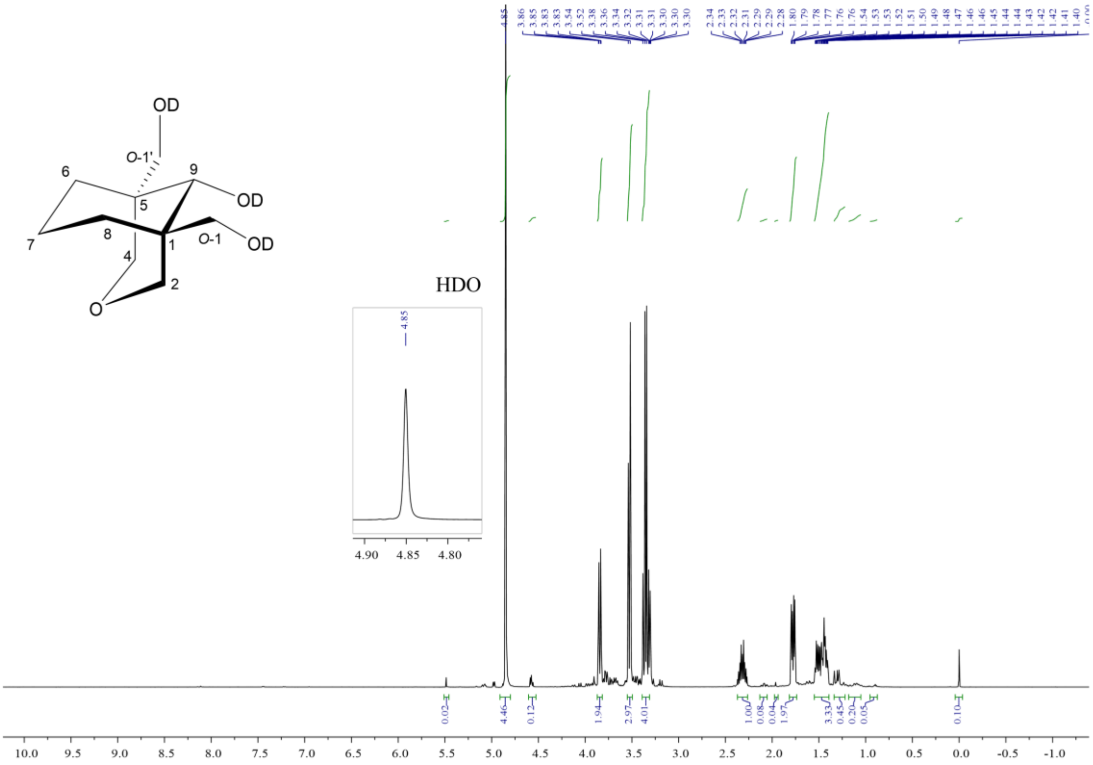

Figure S215. ^{1}H NMR spectrum of [3.3.1]oxabicycle (**36**) in CD_3OD

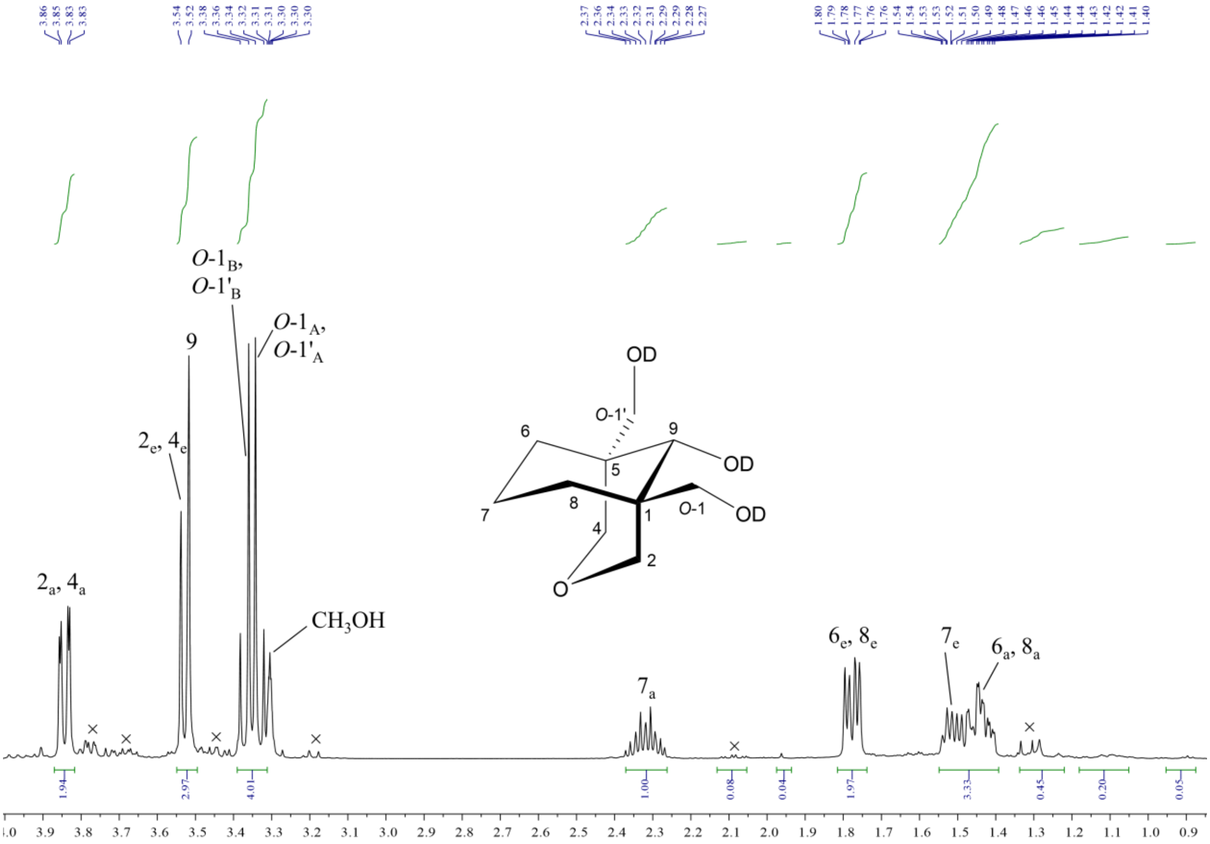

Figure S216. ^{1}H expansion spectrum of [3.3.1]oxabicycle (**36**) in CD_3OD with assignments

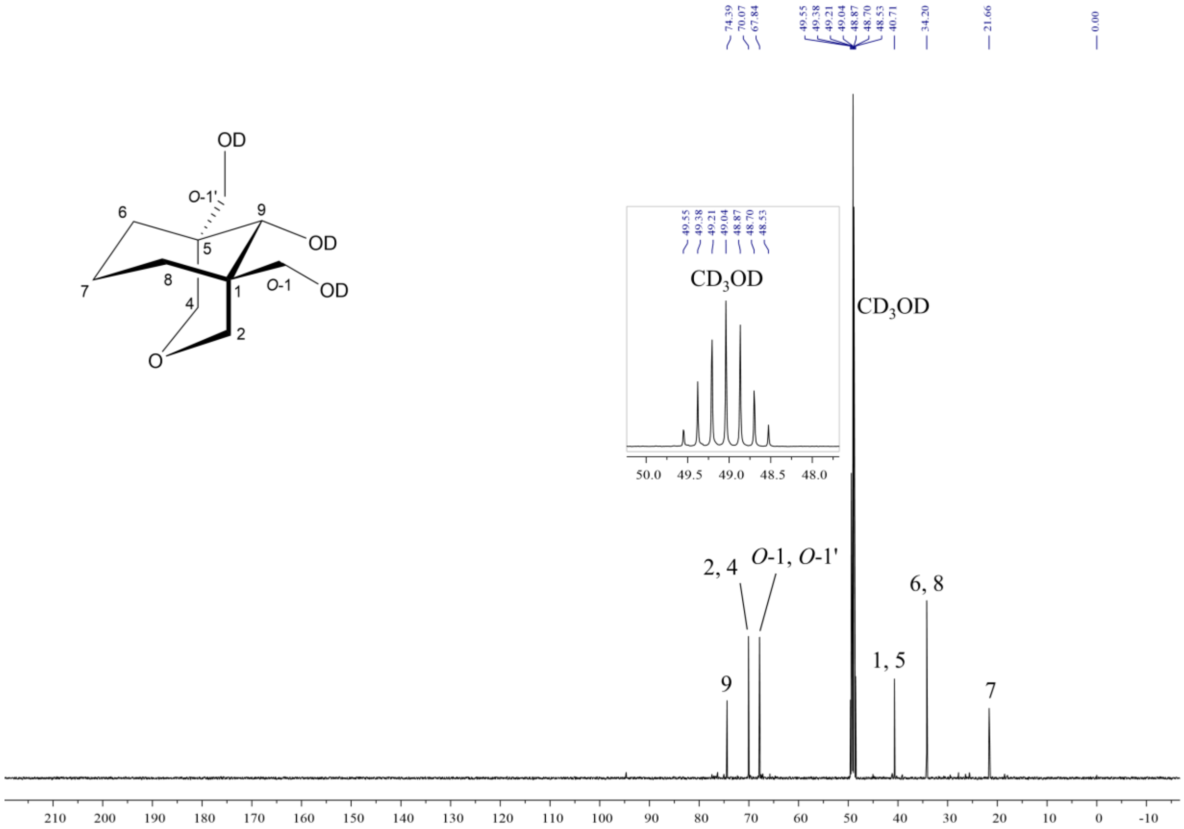

Figure S217. ^{13}C NMR spectrum of [3.3.1]oxabicycle (**36**) in CD_3OD with assignments

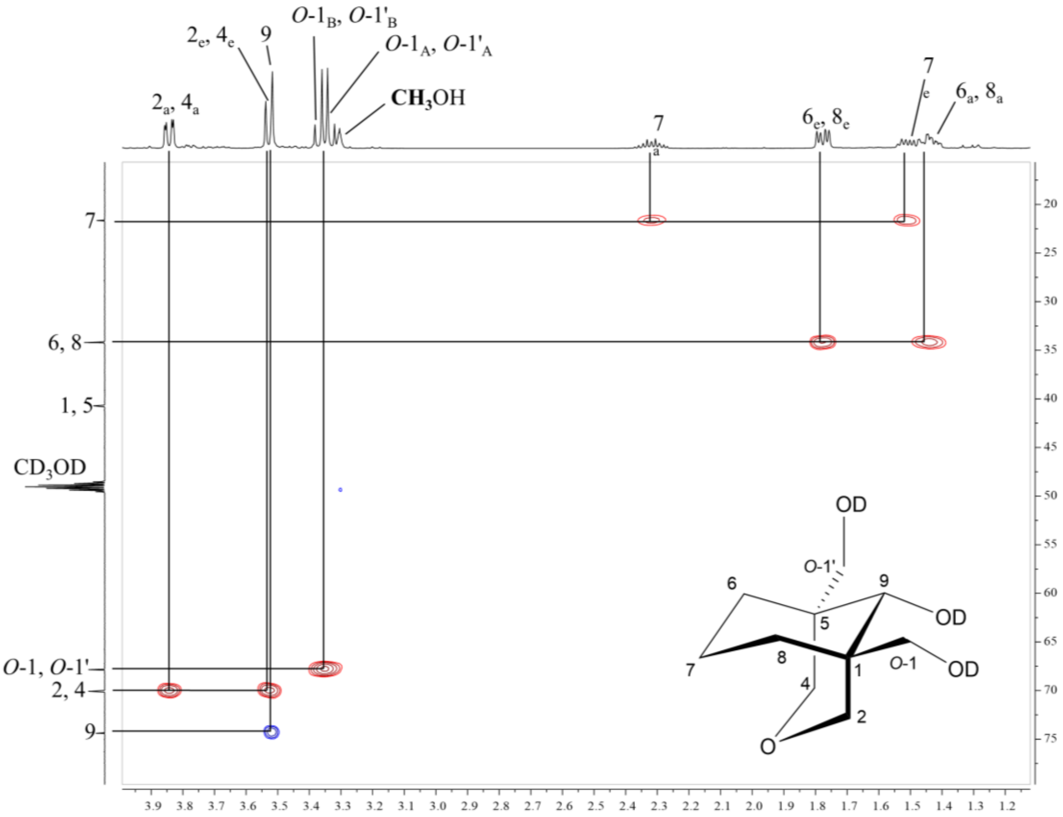

Figure S218. HSQC spectrum of [3.3.1]oxabicycle (**36**) in CD_3OD

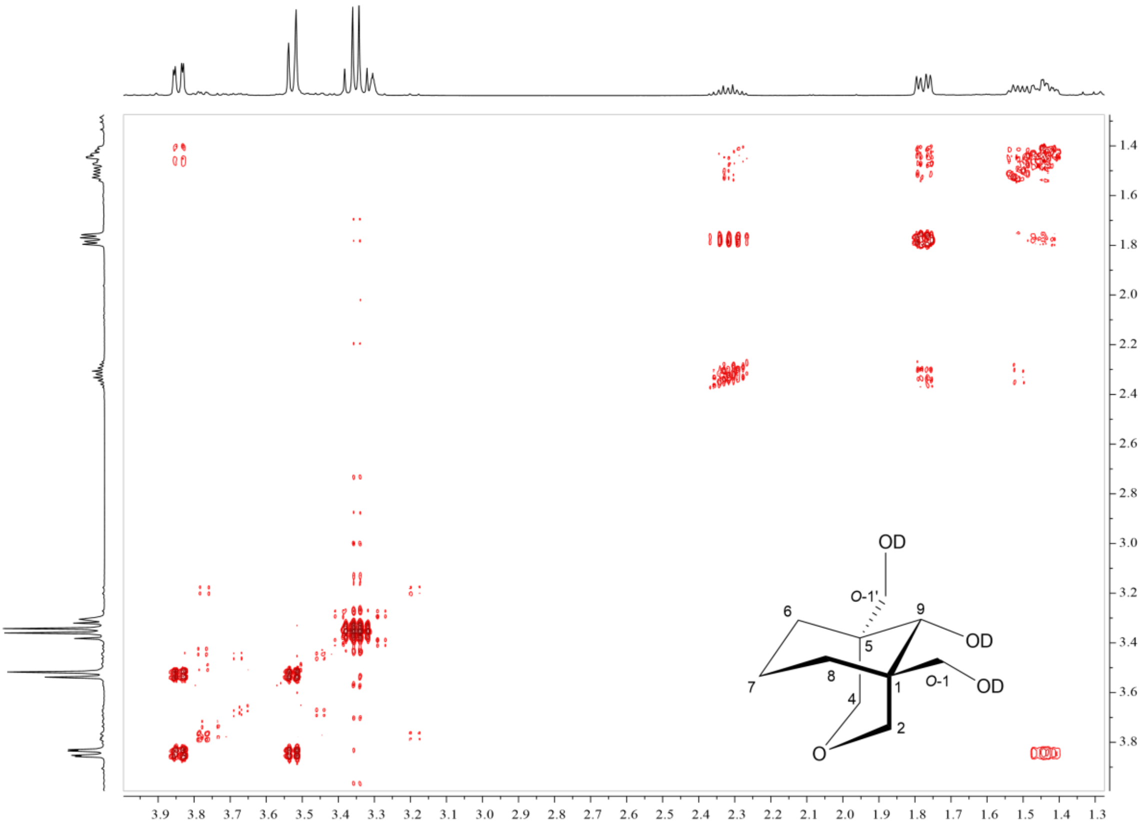

Figure S219. COSY spectrum of [3.3.1]oxabicycle (**36**) in CD_3OD

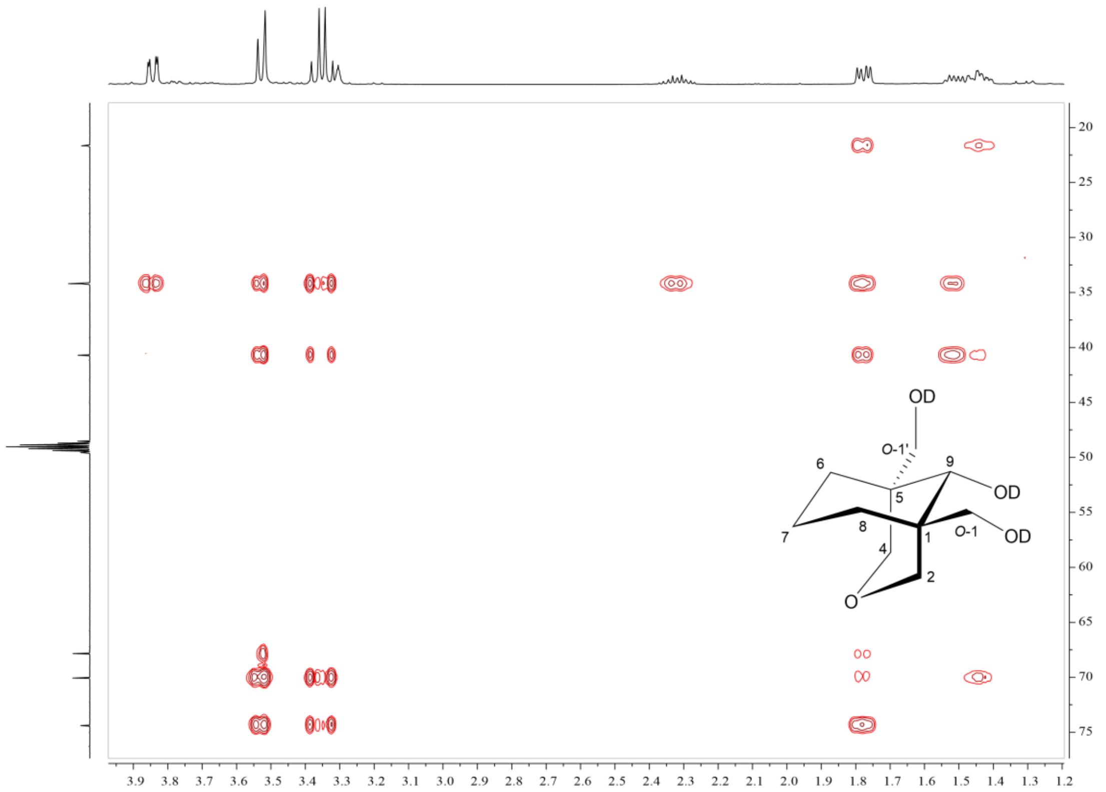

Figure S220. HMBC spectrum of [3.3.1]oxabicycle (**36**) in CD_3OD

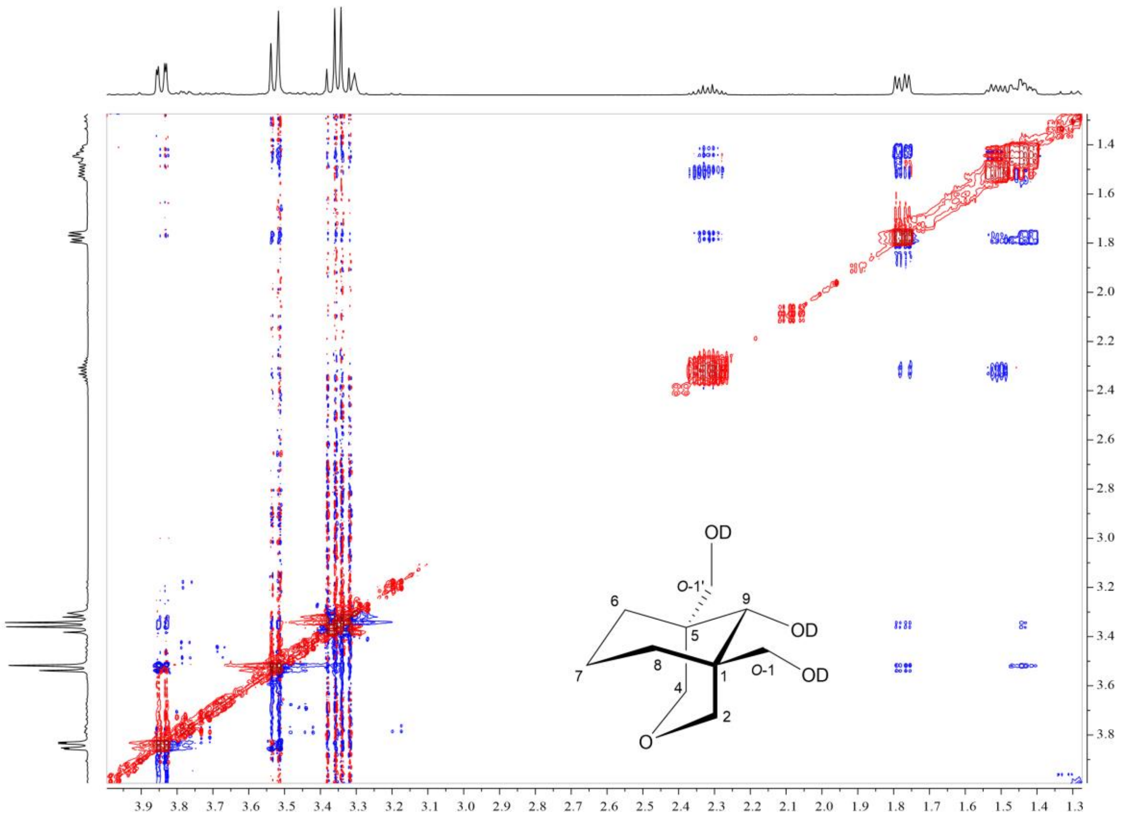

Figure S221. NOESY spectrum of [3.3.1]oxabicycle (**36**) in CD_3OD

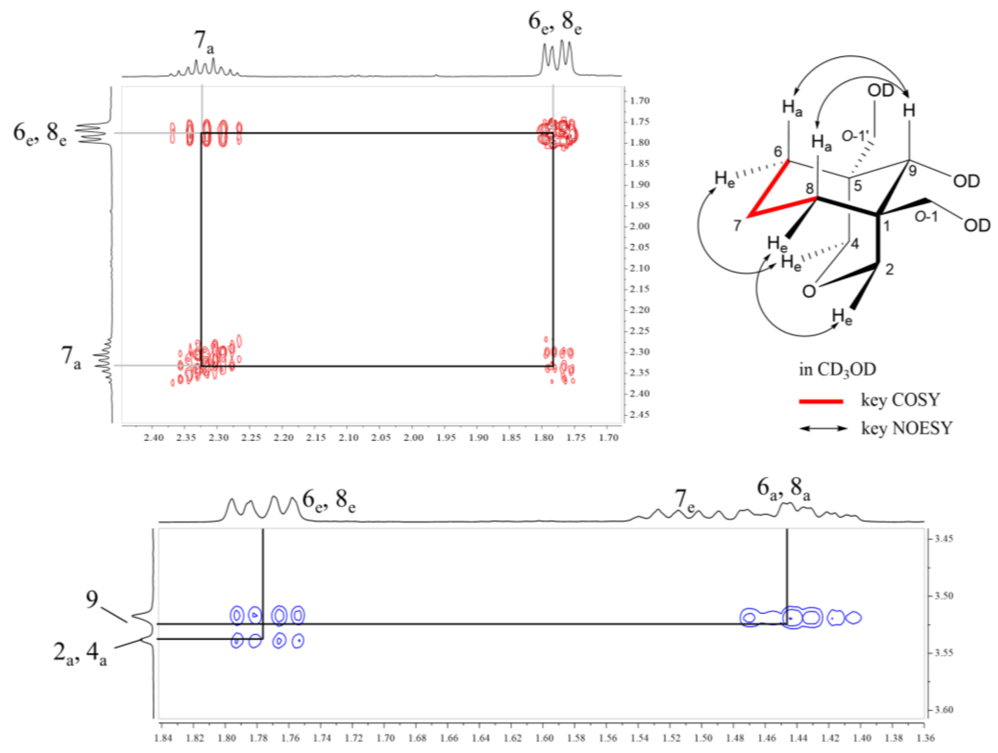

Figure S222. Key 2D correlations of [3.3.1]oxabicycle (**36**) in CD_3OD

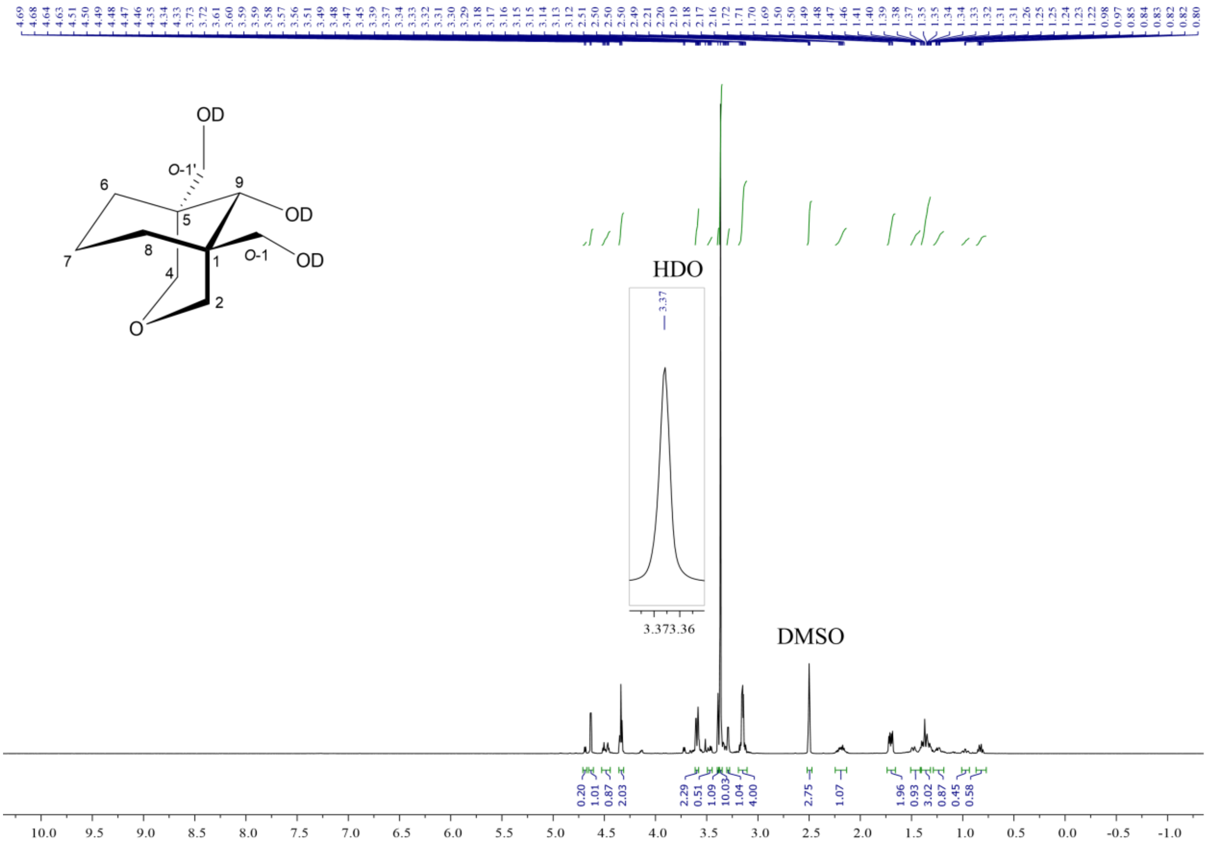

Figure S223. ^{1}H NMR spectrum of [3.3.1]oxabicycle (**36**) in d_6-DMSO

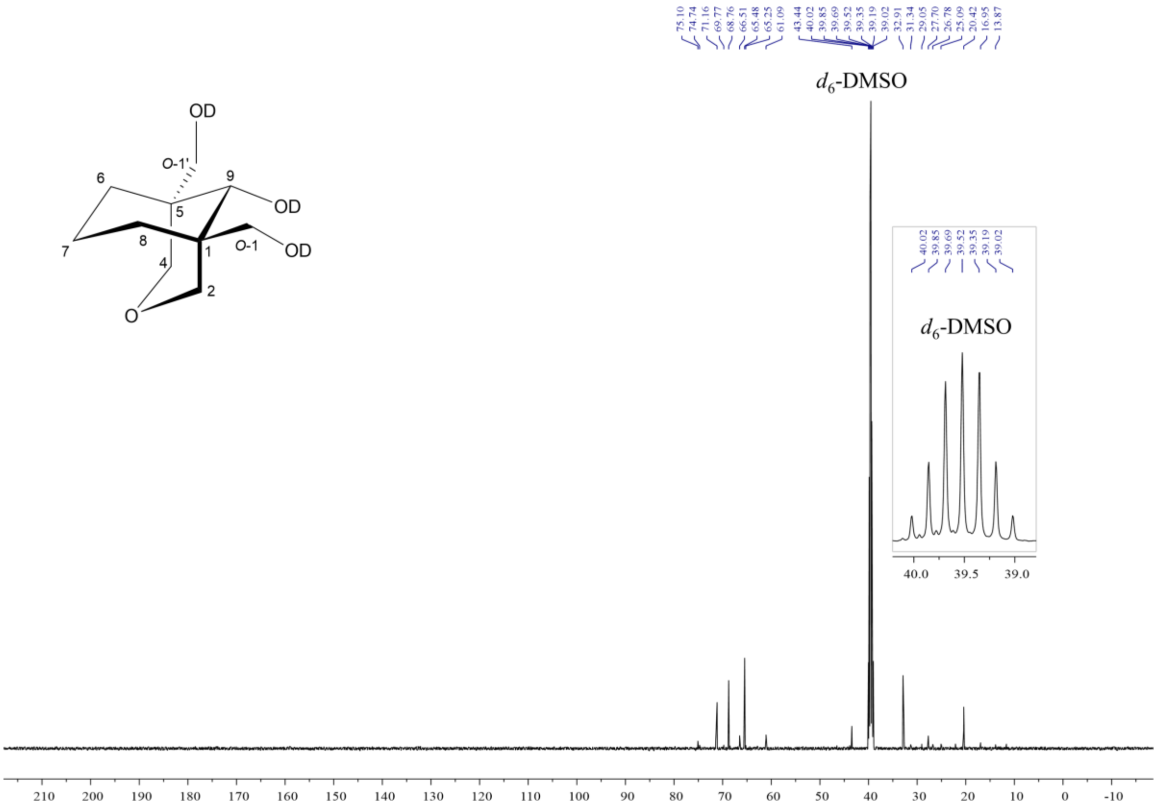

Figure S224. ^{13}C NMR spectrum of [3.3.1]oxabicycle (**36**) in d_6-DMSO

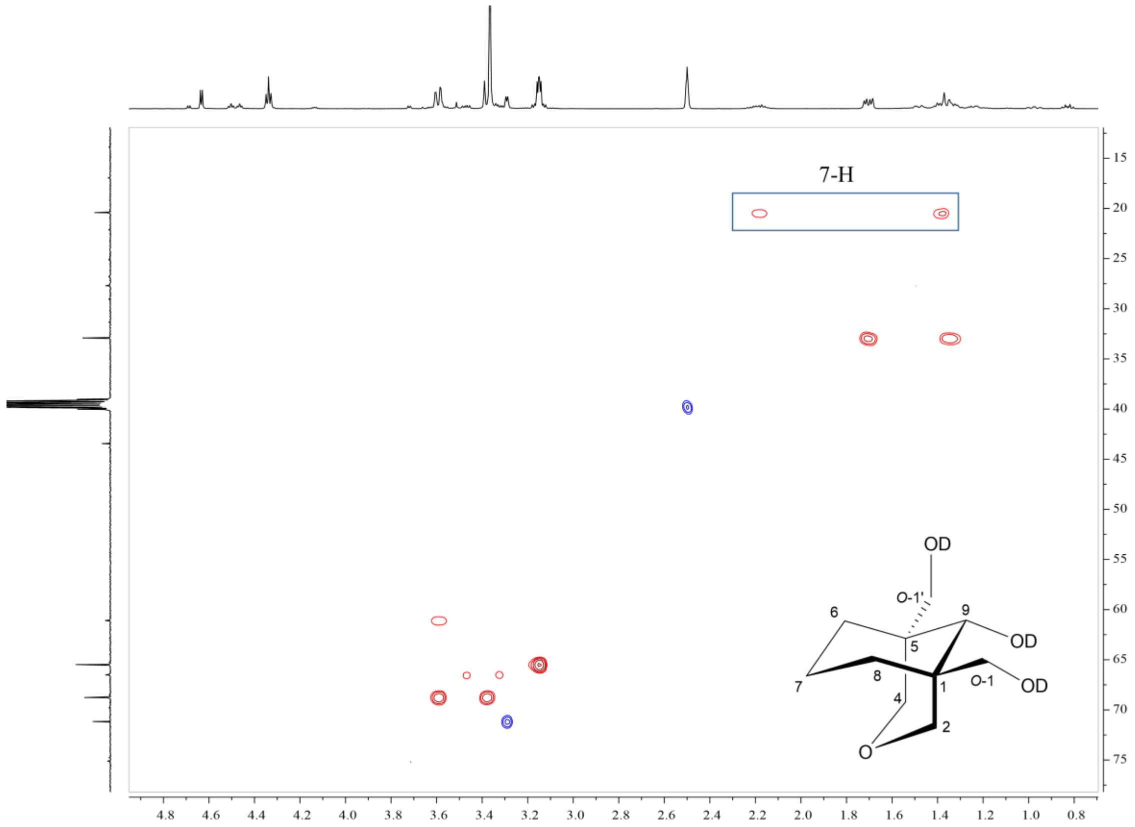

Figure S225. HSQC spectrum of [3.3.1]oxabicycle (**36**) in d_6-DMSO

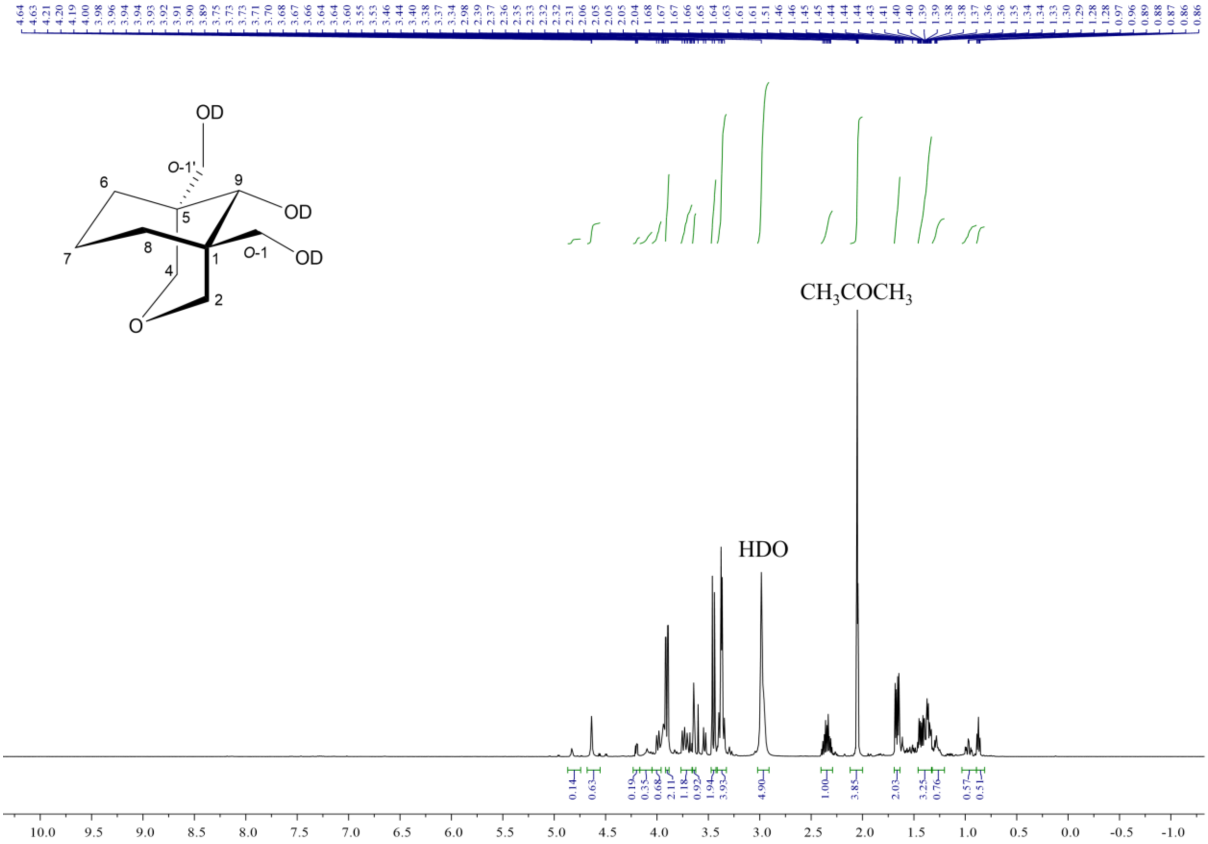

Figure S226. ^{1}H NMR spectrum of [3.3.1]oxabicycle (**36**) in d_6-acetone

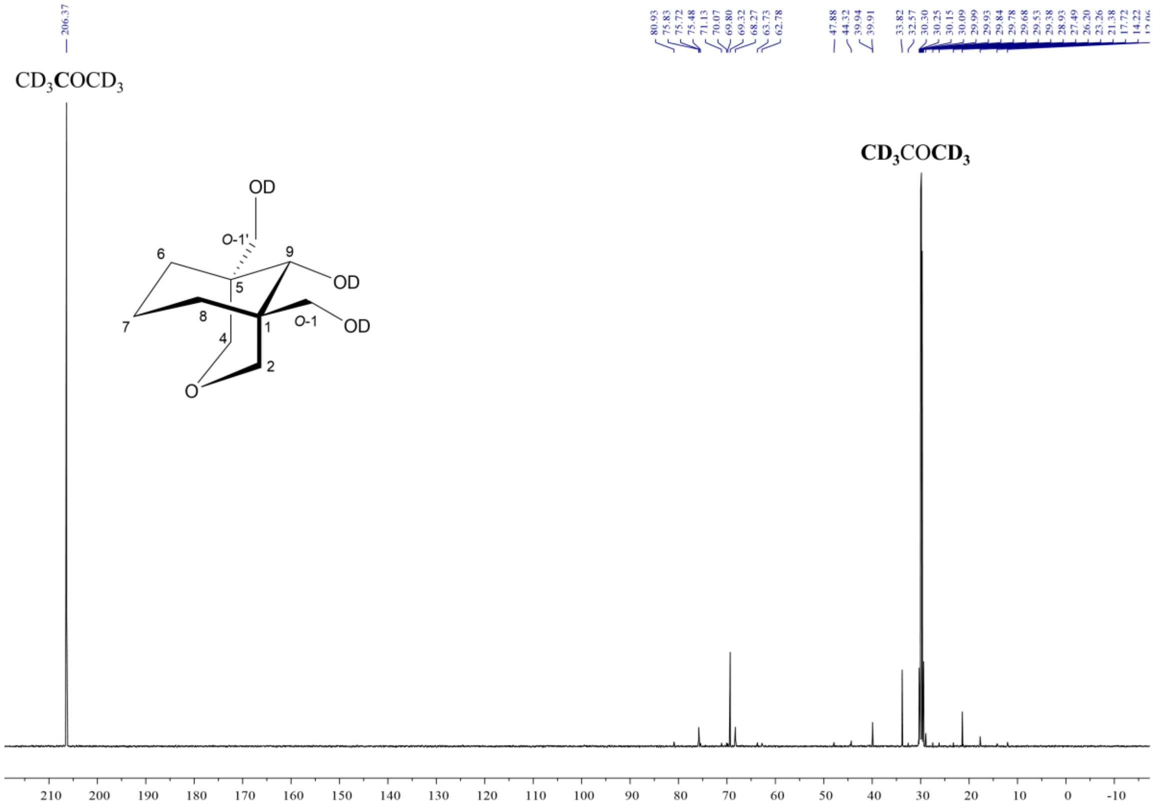

Figure S227. ^{13}C NMR spectrum of [3.3.1]oxabicycle (**36**) in d_6-acetone

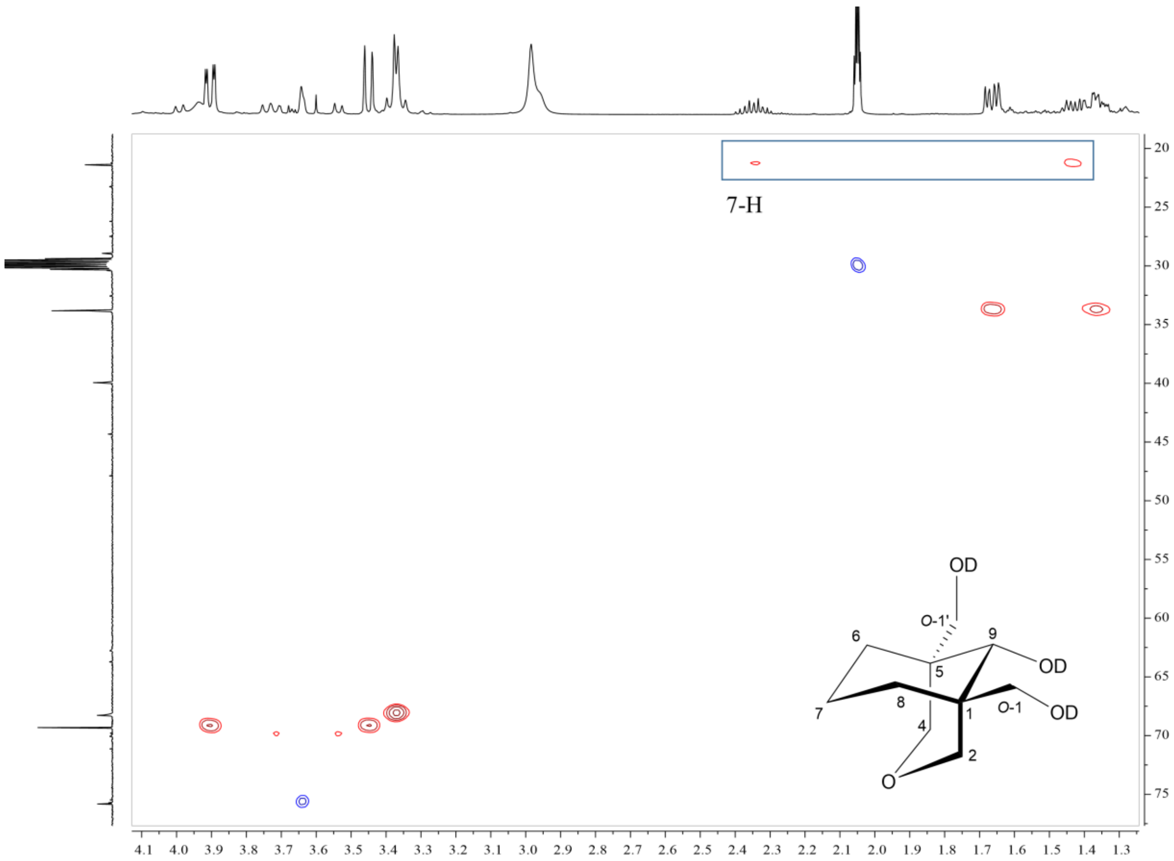

Figure S228. HSQC spectrum of [3.3.1]oxabicycle (**36**) in d_6-acetone

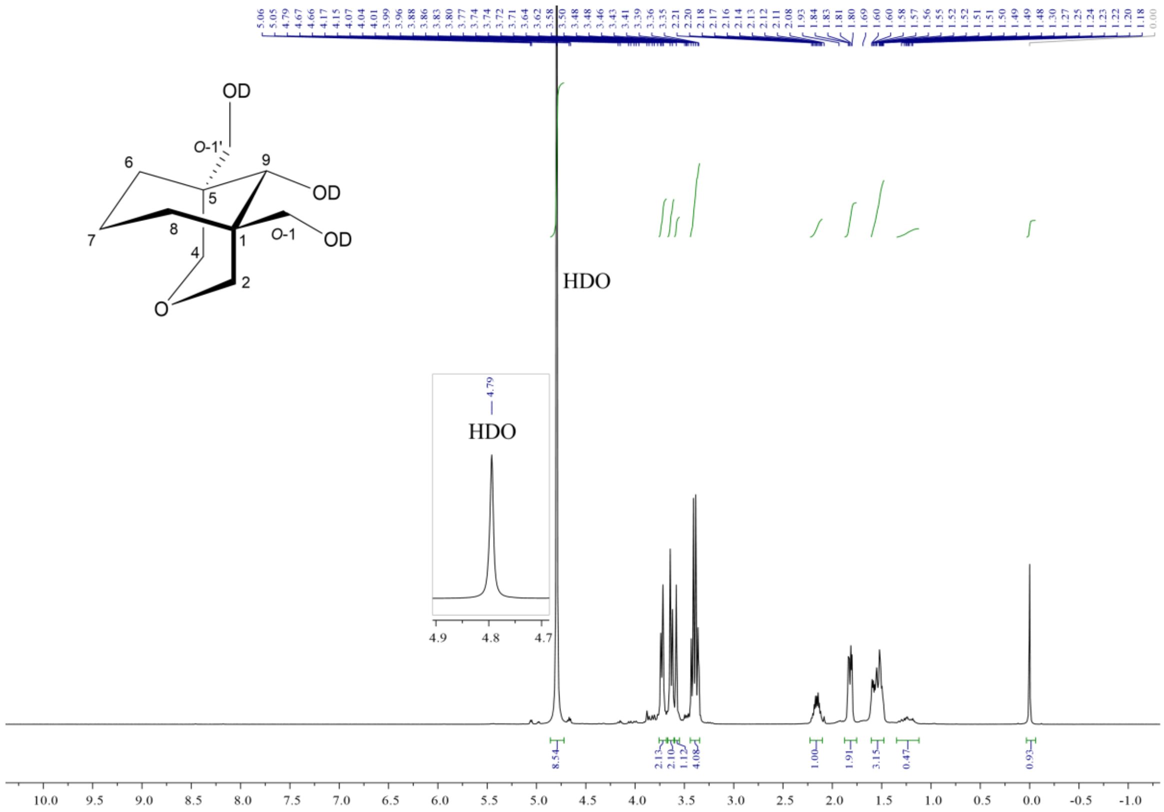

Figure S229. ^{1}H NMR spectrum of [3.3.1]oxabicycle (**36**) in D_2O

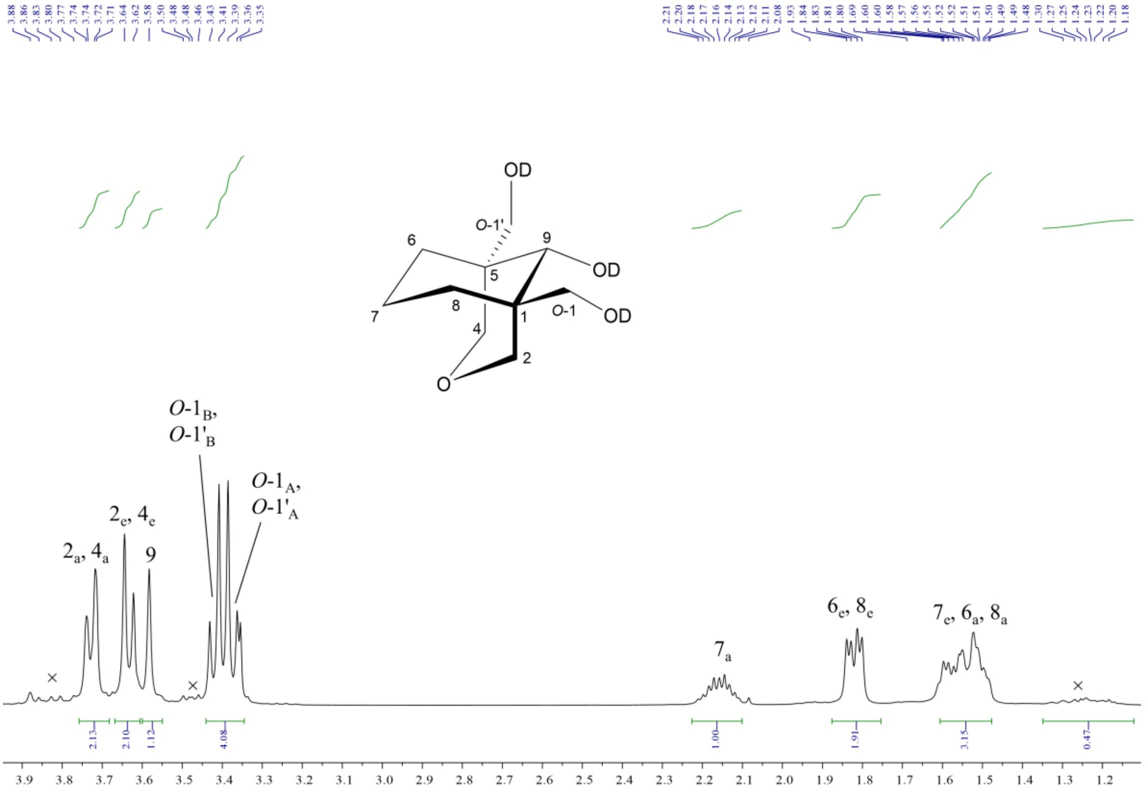

Figure S230. ^{1}H NMR expansion of [3.3.1]oxabicycle (**36**) in D_2O with assignments

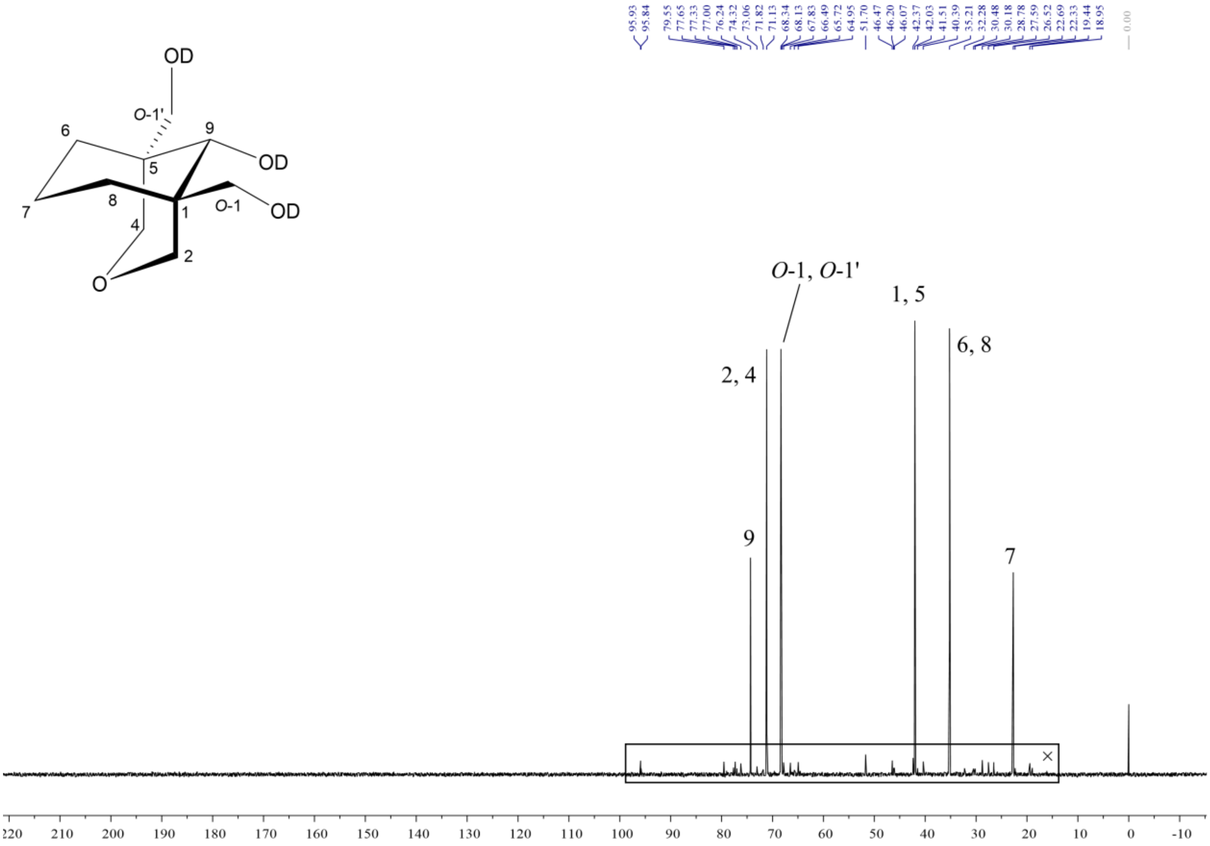

Figure S231. ^{13}C NMR spectrum of [3.3.1]oxabicycle (**36**) in D_2O

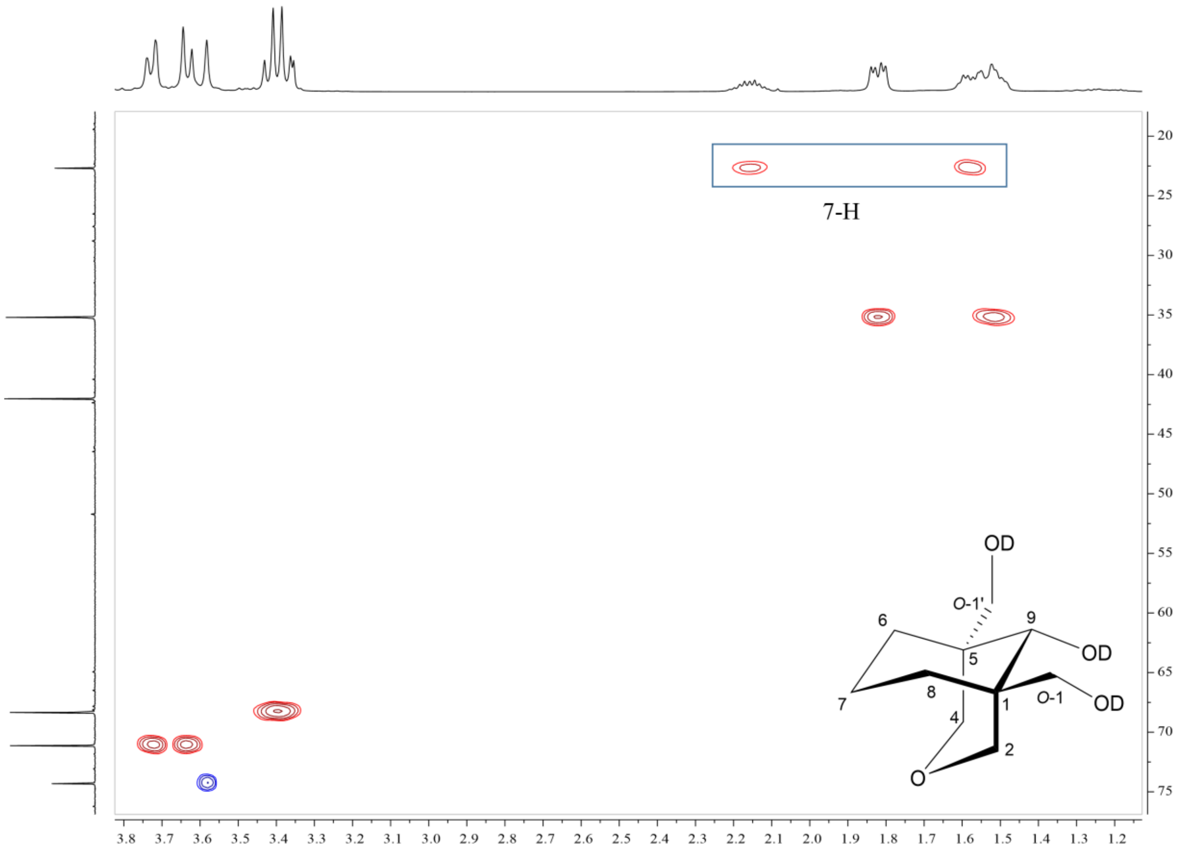

Figure S232. HSQC spectrum of [3.3.1]oxabicycle (**36**) in D_2O

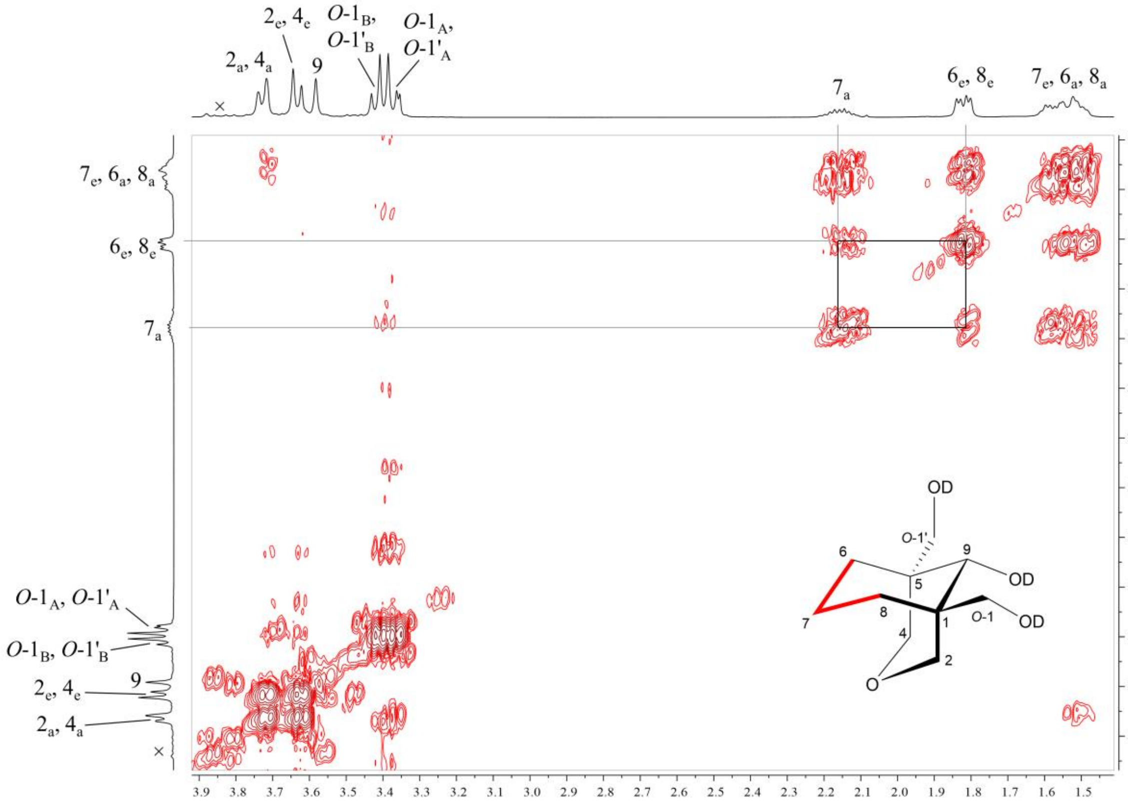

Figure S233. COSY spectrum of [3.3.1]oxabicycle (**36**) in D_2O

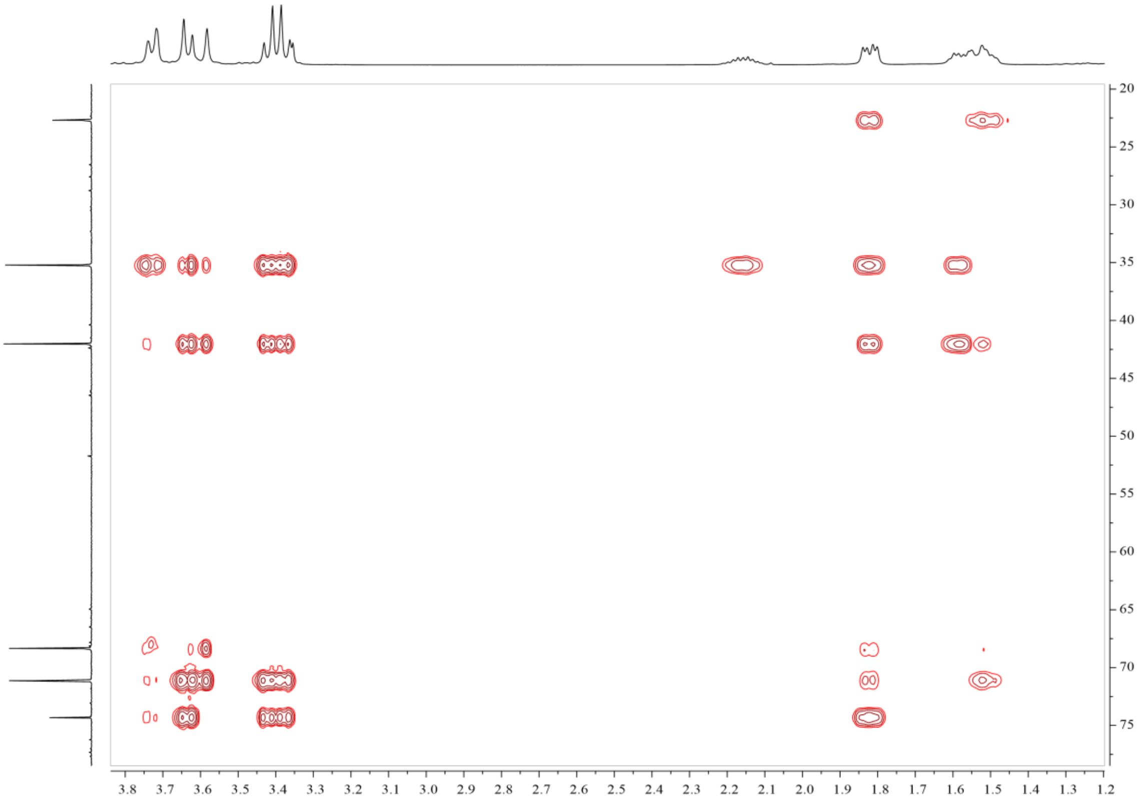

Figure S234. HMBC spectrum of [3.3.1]oxabicycle (**36**) in D_2O

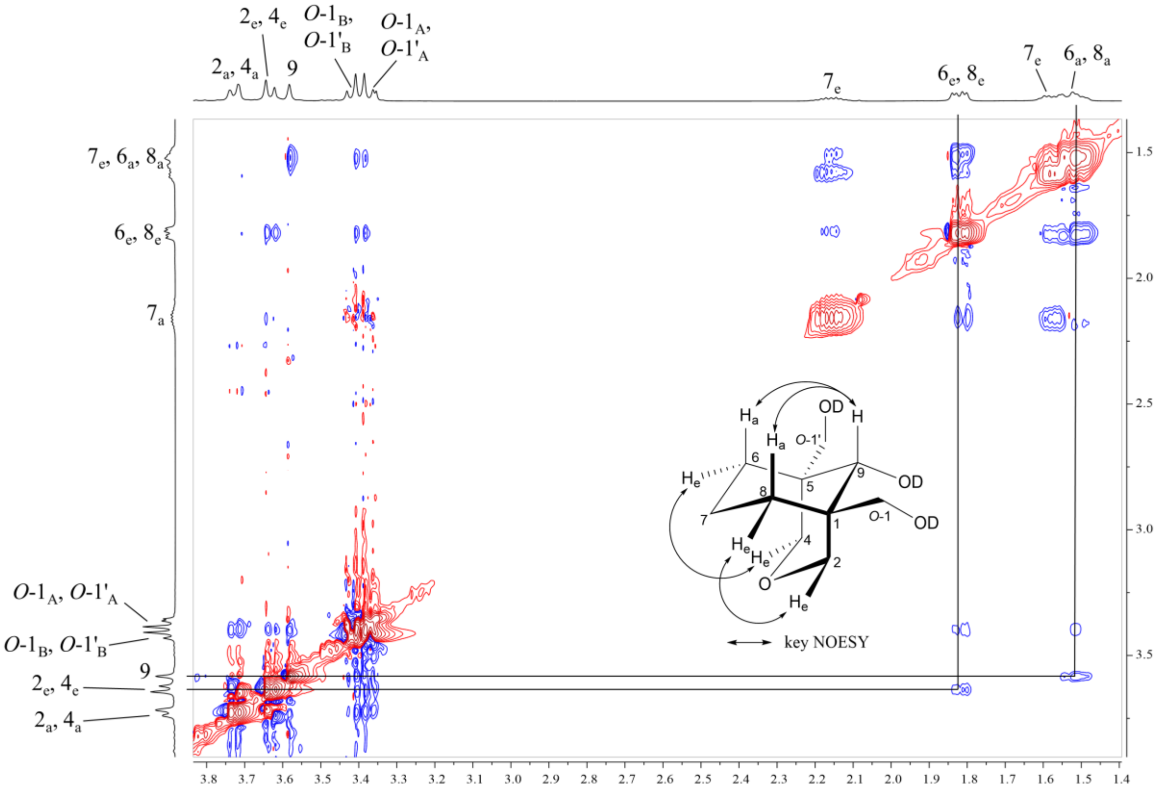

Figure S235. NOESY spectrum of [3.3.1]oxabicycle (**36**) in D_2O

References

(1) Gottlieb, H. E.; Kotlyar, V.; Nudelman, A. NMR chemical shifts of common laboratory solvents as trace impurities. *J. Org. Chem.* **1997**, 62, 7512-7515.

(2) Babij, N. R.; McCusker, E. O.; Whiteker, G. T.; Canturk, B.; Choy, N.; Creemer, L. C.; De Amicis, C. V.; Hewlett, N. M.; Johnson, P. L.; Knobelsdorf, J. A.; Li, F. Z.; Lorsbach, B. A.; Nugent, B. M.; Ryan, S. J.; Smith, M. R.; Yang, Q. NMR chemical shifts of trace impurities: Industrially preferred solvents used in process and green chemistry. *Org. Process. Res. Dev.* **2016**, *20*, 661-667.
